# Structure and Function of the TIR Domain from the Grape NLR Protein RPV1

**DOI:** 10.3389/fpls.2016.01850

**Published:** 2016-12-08

**Authors:** Simon J. Williams, Ling Yin, Gabriel Foley, Lachlan W. Casey, Megan A. Outram, Daniel J. Ericsson, Jiang Lu, Mikael Boden, Ian B. Dry, Bostjan Kobe

**Affiliations:** ^1^School of Chemistry and Molecular Biosciences, Institute for Molecular Bioscience and Australian Infectious Diseases Research Centre, University of Queensland, BrisbaneQLD, Australia; ^2^Research School of Biology, The Australian National University, CanberraACT, Australia; ^3^Guangxi Crop Genetic Improvement and Biotechnology Key Lab, Guangxi Academy of Agricultural SciencesNanning, China; ^4^Commonwealth Scientific and Industrial Research Organisation, UrrbraeSA, Australia; ^5^College of Food Science and Nutritional Engineering, China Agricultural UniversityBeijing, China; ^6^Australian Synchrotron, ClaytonVIC, Australia; ^7^Department of Plant Science, Shanghai Jiao Tong UniversityShanghai, China

**Keywords:** nucleotide-binding oligomerisation domain (NOD)-like receptor (NLR), toll/interleukin-1 receptor (TIR), *Muscadinia rotundifolia*, *Plasmopara viticola*, grapevine downy mildew, plant disease resistance, X-ray crystallography

## Abstract

The N-terminal Toll/interleukin-1 receptor/resistance protein (TIR) domain has been shown to be both necessary and sufficient for defense signaling in the model plants flax and *Arabidopsis*. In examples from these organisms, TIR domain self-association is required for signaling function, albeit through distinct interfaces. Here, we investigate these properties in the TIR domain containing resistance protein RPV1 from the wild grapevine *Muscadinia rotundifolia*. The RPV1 TIR domain, without additional flanking sequence present, is autoactive when transiently expressed in tobacco, demonstrating that the TIR domain alone is capable of cell-death signaling. We determined the crystal structure of the RPV1 TIR domain at 2.3 Å resolution. In the crystals, the RPV1 TIR domain forms a dimer, mediated predominantly through residues in the αA and αE helices (“AE” interface). This interface is shared with the interface discovered in the dimeric complex of the TIR domains from the *Arabidopsis* RPS4/RRS1 resistance protein pair. We show that surface-exposed residues in the AE interface that mediate the dimer interaction in the crystals are highly conserved among plant TIR domain-containing proteins. While we were unable to demonstrate self-association of the RPV1 TIR domain in solution or using yeast 2-hybrid, mutations of surface-exposed residues in the AE interface prevent the cell-death autoactive phenotype. In addition, mutation of residues known to be important in the cell-death signaling function of the flax L6 TIR domain were also shown to be required for RPV1 TIR domain mediated cell-death. Our data demonstrate that multiple TIR domain surfaces control the cell-death function of the RPV1 TIR domain and we suggest that the conserved AE interface may have a general function in TIR-NLR signaling.

## Introduction

To detect pathogens and activate defense responses, plants utilize multi-domain receptor proteins that resemble mammalian innate immunity NLRs [nucleotide-oligomerisation (NOD)-like receptors] (Dangl et al., 2013). In plants, NLRs can interact directly with effector proteins secreted by the invading pathogen, or perceive the presence of effector proteins by monitoring host proteins that are targeted and modified during infection (Dodds et al., 2006; Jones and Dangl, 2006; van Der Hoorn and Kamoun, 2008). This process generally occurs within the plant cell, whereby effector-recognition and subsequent activation of the NLR stimulates an immune response known as the hypersensitive response (HR). The activation of a HR generally culminates in programmed cell death of the infected cell and immunity at the whole plant level in a process commonly referred to as effector-triggered immunity (Dodds and Rathjen, 2010).

The multi-domain architecture of plant NLRs generally involves a C-terminal leucine-rich repeat (LRR) domain, a central nucleotide-binding (NB) domain and either a coiled-coil (CC) domain or Toll-interleukin receptor (TIR) domain at the N-terminus. The LRR domain was originally defined as the effector recognition domain, but this has only been demonstrated for a handful of NLR-effector pairs (Dodds et al., 2006; Krasileva et al., 2010). Subsequent evidence from a number of plant NLR systems suggests a more general regulatory role (Moffett et al., 2002; Ade et al., 2007; Slootweg et al., 2010). While no plant NLR structure is currently available, the crystal structure of the autoinhibited mammalian NLR protein NLRC4 supports an inhibitory role for the LRR domain (Hu et al., 2013). The central NB domain controls the activation of NLR proteins through the binding of adenosine nucleotide di- or tri-phosphate (ADP/ATP; Tameling et al., 2002; Williams et al., 2011). Mutations within conserved motifs that mediate nucleotide binding prevent proper NLR function in most cases. A number of autoactive mutations locate to the NB domain and change the dynamics of, or preference for, ADP/ATP binding (Tameling et al., 2002; Williams et al., 2011). The N-terminal CC and TIR domains have both been implicated in effector-independent activation of cell-death pathways and are therefore generally implicated in NLR signaling (Frost et al., 2004; Swiderski et al., 2009; Krasileva et al., 2010; Bernoux et al., 2011; Collier et al., 2011; Maekawa et al., 2011). However, it has been shown that this cell-death function is not universal. Collier et al. (2011) observed CC domain-dependent cell death from the helper-NLR protein NRG1 but not the canonical solanaceous CC-NLR resistance proteins they tested.

Our current understanding of the molecular and structural basis of plant NLR protein activation and function comes from analyses of the N-terminal domains, and lacks reference to a full-length structure. To date, structures of the CC domains from the barley NLR MLA10, potato NLR Rx and wheat NLR Sr33 have been solved (Maekawa et al., 2011; Hao et al., 2013; Casey et al., 2016), and five crystal structures of plant TIR domains have been published (Chan et al., 2010; Bernoux et al., 2011; Williams et al., 2014). Four TIR domain structures originate from *Arabidopsis* proteins, and include AtTIR, a protein of unknown function, and the TIR domains from the NLRs RRS1 (RRS1^TIR^), RPS4 (RPS4^TIR^) and a heterodimer complex between the two (Chan et al., 2010; Williams et al., 2014). The remaining TIR domain structure is from the flax NLR L6 (L6^TIR^; Bernoux et al., 2011). The known plant TIR domains all share a common fold. They also appear to share functional features, as both L6 and RPS4 TIR domains require self-association for cell-death signaling, albeit through distinct interfaces (Bernoux et al., 2011; Williams et al., 2014). However, in the crystal structures of AtTIR, RRS1^TIR^, RPS4^TIR^ and the RRS1^TIR^:RPS4^TIR^ complex, a common TIR:TIR domain interface has been observed. This interface was shown to control heterodimerisation between the TIR domains from RPS4 and RRS1, which has significant functional consequences for the activation of dual NLR protein resistance provided by these proteins in *Arabidopsis* (Williams et al., 2014). We previously reported that residues within the dimerisation interface of RPS4 and RRS1 that facilitate the interaction are conserved in other plant TIR-NLR proteins but that their function in other NLR proteins was not yet known.

In an effort to understand TIR domain function further, we investigated the TIR domain from the *Muscadinia rotundifolia* TIR-NLR protein RPV1 (resistance to *Plasmopara viticola* 1). *M. rotundifolia* is a wild North American grape species closely related to the cultivated grapevine species *Vitis vinifera*, and RPV1 confers resistance to the oomycete *Plasmopara viticola*, the casual agent of downy mildew in cultivated grapevines (Feechan et al., 2013). Here we report the crystal structure of the TIR domain of RPV1 (residues 20–193; RPV1^TIR20-193^) at 2.3 Å resolution. In the crystal structure we observe a molecular interface within the asymmetric unit involving residues within the αA and αE helices that resembles the interface previously observed for AtTIR, RRS1^TIR^, RPS4^TIR^ and RRS1^TIR^:RPS4^TIR^ structures. A thorough assessment of sequencing data from the plant Phytozome resource (Goodstein et al., 2012) reveals that surface-exposed residues within the αA and αE helices involved in molecular contacts in the RPV1 TIR domain crystal structures are well conserved among plant species. We demonstrate that the integrity of this interface is important for the autoactive signaling function of RPV1 TIR domain in a *Nicotiana tabacum* transient expression system. Additionally, we show that mutations in other distinct protein surfaces, including a region analogous to that required for L6^TIR^ self-association and signaling, also disrupts cell-death signaling. In light of these observations, we suggest that in addition to its role in the dual-NLR protein function in *Arabidopsis*, the AE interface may play a more general functional role in TIR-NLR protein function and that multiple, distinct protein surfaces in plant TIR domains influence TIR domain signaling.

## Materials and Methods

### Vectors and Constructs

Truncation constructs coding for the RPV1 TIR domain were prepared by amplification of the corresponding fragments from a plasmid template of *MrRPV1* full-length cDNA (Feechan et al., 2013) with primers containing Gateway attB sites, using polymerase chain reaction (PCR). The PCR products were cloned into the donor vector pDONR223 (Invitrogen) using BP clonase. To create protein fusions with yellow fluorescent protein (YFP) tags, the desired entry clone and destination vector (pEarlyGate100-L-YFP) containing attR1 and attR2 sites were recombined by LR reaction. The vector pEarlygate100-L-YFP was derived from pEarlyGate100 (Earley et al., 2006) by the introduction of an AvrII-SpeI GA linker-vYFP fragment from ER082 (Tucker et al., 2012). All MrRPV1 TIR domain mutants were created from the plasmid of the entry clone with the QuikChange Site-Directed Mutagenesis Kit (Agilent Technologies) according to the manufacturer’s instructions. The desired mutations were recombined into pEarlygate100-L-YFP by LR reaction. For yeast-two-hybrid (Y2H) studies, RPV1^TIR1-193^ was recombined into the Gateway-compatible Y2H vectors based on pGADT7 and pGBKT7 (Clontech) kindly provided by Dr Maud Bernoux (CSIRO Agriculture, Canberra). All constructs were sequenced for verification. All primers used to generate the above constructs are listed in Supplementary Table S1.

### Transient Expression and Yeast-2-Hybrid Assays

Tobacco (*N. tabacum* cv. White Burley) plants were grown in a greenhouse at the Commonwealth Scientific and Industrial Research Organisation (CSIRO), Adelaide, Australia. Agrobacterium (strain EHA105) cells were in grown in yeast extract peptone (YEP) media supplemented with 25 μg/mL rifampicin and 50 μg/mL kanamycin at 28°C for 2 days. Approximately 0.5 ml culture was inoculated into 50 ml of YM+MES (pH 5.6) media supplemented with 20 μM acetosyringone and 50 μg/mL kanamycin and grown at 28°C until the OD_600 nm_ was >0.5. Cells were pelleted and resuspended in infiltration medium (10 mM MgCl_2_, 10 mM MES pH 5.6, 200 μM acetosyringone) to give a final OD_600 nm_ of 0.5 and incubated at room temperature for 2–3 h. Infiltration of rapidly expanding tobacco leaves was carried out with a blunt needleless syringe. Plants were maintained at a constant temperate of 23°C for 48 h before being transferred to the glasshouse.

Yeast transformation and growth assays were performed essentially as described in the Yeast Protocols Handbook (Clontech). Yeast cells (AH109) were co-transformed with the prey and bait vectors using a lithium acetate-based protocol. Transformants were first spread on CSM-Trp-Leu plates, and then co-transformed positive colonies were grown on CSM-Trp-Leu-His + 5 mM 3-AT plates to detect activation of the *HIS3* reporter gene. Prey and bait vectors containing the L6^TIR29-233^ (Bernoux et al., 2011) were used as a positive control. Empty prey and bait vectors were used as a negative control.

### Immunoblot Analysis

Leaf tissue (∼1 cm diameter disk) was collected from the middle of an infiltrated area 48 h after agroinfiltration. Tissue was ground up in 100 μl of 2X Laemmli extraction buffer, centrifuged at 23,000 x *g* for 10 min at 4°C and the supernatant fraction collected. Proteins were separated by SDS-PAGE and transferred to nitrocellulose membranes (Pall). Membranes were blocked in 5% skim milk and probed with anti-GFP (Roche) followed by goat anti-mouse antibodies conjugated with horseradish peroxidase (Thermo Scientific). Labeling was detected with a SuperSignal West Pico chemiluminescence kit (Thermo Scientific) following the manufacturer’s instructions. Membranes were stained with Ponceau S staining solution for protein loading.

### Cloning, Expression and Protein Purification

RPV1^TIR20-193^ and RPV1^TIR20-193^ ^H42A^ were amplified from plasmid templates described above using the primer combinations RPV1^TIR^20-F and RPV1^TIR^193-R (Supplementary Table S1). The PCR product was cloned into the *Escherichia coli* expression vector pMCSG7 (Stols et al., 2002) by ligation-independent cloning and verified by sequencing. The proteins were expressed in *E. coli* BL21 (DE3) cells using the autoinduction method (Studier, 2005). All media was supplemented with 100 μg/mL ampicillin for plasmid selection. An overnight culture was used to inoculate large-scale cultures. Cells were grown by continuous shaking at 37°C in 2 L flasks containing 500 mL of media until the OD_600 nm_ reached 0.6–0.8. At this point, the temperature was reduced to 20°C and the cells were grown for an additional 18 h before harvesting by centrifugation.

Cells expressing the protein of interest were resuspended in a lysis buffer containing 50 mM HEPES (pH 8.0), 300 mM NaCl and 1 mM DTT. The cells were lysed using sonication, clarified by centrifugation and the resulting supernatant was applied to a 5 mL HisTrap FF column (GE Healthcare). The column was washed with the lysis buffer supplemented with 30 mM imidazole to remove non-specifically bound proteins. The bound protein was eluted using a linear gradient of imidazole from 30 to 250 mM. Fractions containing the protein of interest were pooled, concentrated and buffer-exchanged (to remove imidazole) into 50 mM Tris pH 8.0, 250 mM NaCl, 1 mM DTT and 0.1 mM EDTA for overnight treatment with His-tagged TEV protease at 4°C. The cleaved protein was reapplied to the HisTrap FF column to remove the histidine tag, TEV protease and other contaminants and the flow-through was concentrated and separated further on a Superdex 75 HiLoad 26/60 gel-filtration column (GE Healthcare) pre-equilibrated with the gel filtration buffer containing 10 mM HEPES (pH 7.5), 150 mM NaCl and 1 mM DTT. The peak fractions were pooled and concentrated to 10 mg/mL. Protein was stored in aliquots at -80°C for future biophysical and crystallization studies.

### Crystallization and Crystallography

Initial screening was prepared using a Mosquito robot (TTP LabTech, UK) in a 96-well format. The hanging drop vapor-diffusion method of crystallization was used, and drops consisting of 100 nl protein solution and 100 nl reservoir solution were equilibrated against 80 μl reservoir solution. Eight commercial screens were utilized: Index, PEG/Ion and PEGRx (Hampton Research), Morpheus, ProPlex, JCSG Plus, PACT Premier (Molecular Dimensions) and Precipitant Synergy (Jena Biosciences). A number of single crystals were observed in the screening plates and these were harvested directly from the screens and after cryo-protection with 20% glycerol in well solution the crystals were vitrified in liquid nitrogen. The crystals were subjected to X-ray radiation at the Australian Synchrotron MX1 beamline. X-ray diffraction data to approximately 2.3 Å resolution was obtained from a crystal (approximate dimensions 200 μm × 30 μm × 30 μm) harvested from the PEG/Ion screen, grown in condition 16% PEG3350, 0.1 M Tris pH 8.5 and 2% Tacsimate. Data was collected using a wavelength of 0.9537 Å with a detector distance of 200 mm. The resulting dataset was indexed and integrated with XDS (Kabsch, 2010) and scaled with Aimless (Evans and Murshudov, 2013). Molecular replacement was performed using the program phaser (McCoy et al., 2007) using the RPS4^TIR^ structure (PDB ID 4c6r) as a template. Automated model building was performed in the Phenix package using autobuild, while phenix refine combined with manual inspection and corrections using coot (Emsley et al., 2010) were combined to produce the final atomic model.

### Biophysical Studies

Multi-angle laser light scattering (MALS) and small-angle x-ray scattering (SAXS) were used in conjunction with size-exclusion chromatography (SEC) to assess the molecular mass of RPV1^TIR20-193^ in solution. SEC-SAXS was performed at the SAXS/WAXS beamline of the Australian Synchrotron. A Pilatus 1M detector at a sample-to-detector distance of 1.6 m and an energy of 12 keV yielded data over a *q*-range of 0.007–0.361 Å^-1^, where *q*=4π.sin (θ)/λ. 1 mg of both RPV1^TIR^ and RPV1^TIR20-193^H34A were separated over an inline 3 mL Superdex S200 5/150 GL Increase column (GE Healthcare) at 16°C, at a flow rate of 0.2 mL/min in 10 mM HEPES (pH 7.5), 150 mM NaCl buffer with 1 mM DTT. Frames were collected in 2 s exposures. Data reduction and subtraction was performed using scatterBrain^1^. 100 frames immediately preceding each peak were summed and normalized to obtain buffer blanks, which were subtracted from each individual frame across the peak. Values of *R*_g_ and *I*(0) were calculated from each frame via Autorg in the PRIMUS suite (Petoukhov et al., 2012), and molecular masses were calculated from the volume of correlation for points where 0 < *q* < 0.3 Å^-1^ (Rambo and Tainer, 2013).

### Sequence-Level Analysis of Conservation

To gain insight into whether the AE interface is conserved across a wider sample of plant species, TIR domain-annotated sequences from Pfam version 29.0 (Finn et al., 2016) were aligned and used to construct profile Hidden Markov Models (profile HMMs). These profile HMMs were then used to search plant genomes from the more extensive Phytozome database (**Supplementary Figure S1**). Pfam sequences were mapped to four clades and four individual sequences within Phytozome in order to include representative sequences from a greater number of plant species than present in Pfam (**Supplementary Figure S2**). We were able to uncover a larger set of TIR domains by expanding the search from Pfam to Phytozome, except in the cases of *Arabidopsis thaliana* and *V. vinifera*, in which mapping to the Phytozome database resulted in fewer proteins than originally identified within Pfam (Supplementary Table S2 and Supplementary Data Sheet 1).

The Pfam description of TIR domains (PF01582) does not always account for the entire sequence of the domain, judging from the available structures, with sequences often truncated at the C-terminus of the domain (*i.e.*, missing the αE helix). To address these errors, the full-length protein sequences for each Pfam entry were retrieved from UniProt (UniProt, 2015).

When an individual sequence had multiple annotated TIR domains, it was divided into regions that maximized the length of each region. If an individual region’s new length was below 100 amino-acids, it was deemed unlikely to represent a true TIR domain and was re-joined with the neighboring region in the sequence. If two or more neighboring region’s new lengths were greater than 100 amino-acids the original sequence was split at the boundary and the regions were included as separate sequences within the set.

The previously reported AE interface within RPS4 (Williams et al., 2014) was used as a reference to exclude sequences that did not contain the αA and αE regions. As RPS4 was only present within the Malvids clade and Pentapetalae clade, for all other sets RPS4 was manually added and aligned to identify a new sequence that aligned to RPS4 in the AE interface regions and could serve as a reference sequence for its set. After identification of this reference sequence, RPS4 was removed from the alignments for subsequent analysis.

The sequences were clustered using CD-HIT (Li and Godzik, 2006) at 100% identity to identify any duplicate sequences and remove them. Sequences were aligned using MAFFT (Katoh and Standley, 2013) with the default FFT-NS-2 strategy. Any sequence in the alignment that had less than 50% of positions containing an amino-acid in either the αA or αE regions defined by the reference sequence were tentatively excluded. The remaining sequences were realigned and this exclusion process was repeated until no sequences were present in the alignment that had less than 50% of positions containing an amino-acid in either of these regions.

Excluded sequences were checked for the possibility of truncation of the UniProt record. A BLAST search was conducted that only accepted hits longer than the query sequence but that otherwise exactly matched the query sequence. The results from this BLAST search were realigned to the set of sequences without 50% or higher gaps in the αA or αE regions, and all sequences with 50% or higher gaps were definitively excluded.

The remaining sequences within these clades were aligned and used to build four profile HMMs specific to their containing sequences. To identify TIR domains in species not annotated by Pfam, the characteristic profile HMMs were used to query sets of predicted proteins from primary transcripts of genomes found in the Phytozome database. The primary transcripts were from clades containing the original sets of species used to derive each profile.

A profile HMM was constructed using HMMER 3.1b2 (Eddy, 2011) and used to search the relevant subset of the Phytozome database. Hits from the profile HMM search of the Phytozome databases with an *E*-value cut-off of 1*e*-5 were used to construct a multiple sequence alignment following the previous protocol of clustering at 100% identity with CD-HIT, identification of a reference sequence, alignment with MAFFT FFT-NS-2 strategy, and exclusion of sequences missing characters at over 50% of the reference sequence’s designated αA or αE regions. The resulting multiple sequence alignments were trimmed of columns that had gaps in 75% or more of the sequences, and visualized as sequence logos. Note that residues at positions 18–20 representing the start of the αE region are presented here in the sequence logos for completeness; however, there is a higher presence of gaps in the alignments at positions 18–20 and alignment error could account for the differences of amino-acids at these positions.

In order to highlight patterns occurring at the species level, individual species from the four clades were chosen—*A. thaliana*, *Glycine max*, *P. persica*, *Populus trichocarpa*, and *V. vinifera*. The same search procedure as for the larger sets was repeated for each of these species. Alignments and profile HMMs were constructed specific to each individual species and the subsets of the Phytozome database used for the more extensive search were restricted to sequences from each individual species.

## Results

### The RPV1 TIR Domain Causes Cell Death in Tobacco

**FIGURE 1:**
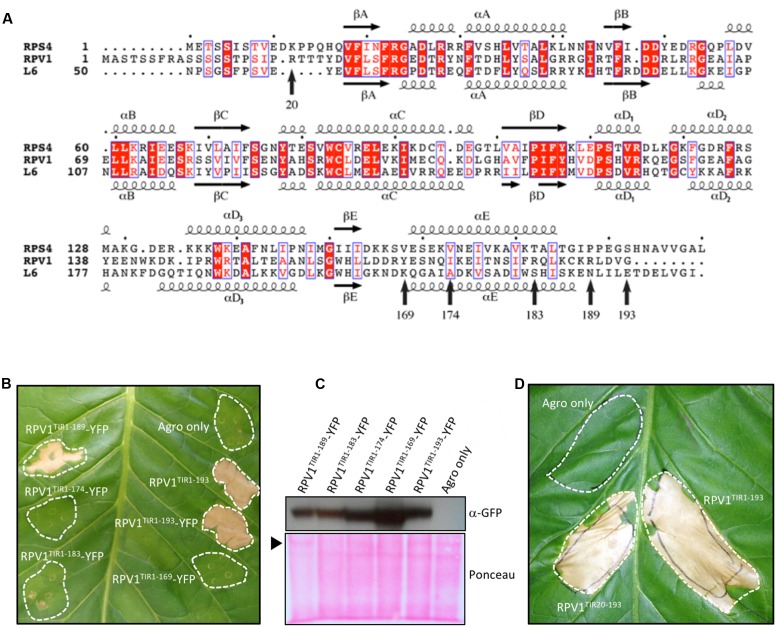
**RPV1^TIR^ cell-death signaling.** **(A)** Multiple sequence alignment containing RPS4 (residues 1–193), RPV1 (1–193) and L6 (50–240). The alignment was formatted using the program ESPript (Robert and Gouet, 2014). The secondary structure shown above and below the alignment is derived from the RPS4^TIR^ (PDB ID 4c6r) and L6^TIR^ (PDB ID 3ozi) structures, respectively. Arrows indicate residue positions corresponding to the various RPV1 constructs tested in **(B,D)**. **(B,D)** *Nicotiana tabacum* leaves 5 days after infiltration with *A. tumefaciens* alone or *A. tumefaciens* expressing RPV1^TIR^ constructs. **(B)** Comparison of the RPV1^TIR^ truncations fused to YFP. **(C)** Protein extracts of tobacco-leaf tissue **(B)**, sampled from agroinfiltrated areas 2 days after agroinfiltration, were immunoblotted with anti-GFP antibodies, demonstrating RPV1^TIR^-YFP protein expression. The arrow indicates Ponceau staining of the large RuBisCO subunit. **(D)** Autoactivity is observed with both RPV1^TIR1-193^ and RPV1^TIR20-193^ constructs without YFP. Agro only, corresponds to agrobacterium transformed with a vector without an insert.

The *RPV1* gene from *M. rotundifolia* encodes a TIR-NB-LRR protein that confers resistance to the oomycte pathogen *P. viticola* (Feechan et al., 2013). Based on the protein structures of RPS4^TIR^ and L6^TIR^ (Bernoux et al., 2011; Williams et al., 2014), the TIR domain is predicted to be located between residues 20 and 188 (**Figure 1A**). As TIR-domain autoactivity has been demonstrated for a number of plant NLR proteins (Frost et al., 2004; Swiderski et al., 2009; Krasileva et al., 2010; Bernoux et al., 2011), we tested whether the RPV1 TIR domain was also autoactive. Agroinfiltration experiments confirmed that RPV1^TIR1-193^ and RPV1^TIR20-193^ were capable of causing rapid cell death in *N. tabacum* (**Figures 1B,D**). To determine the minimal functional region of RPV1 required for autoactivity, we tested a series of RPV1^TIR^ truncated fragments fused to YFP for autoactivity in tobacco. The addition of the YFP tag had no effect on RPV1^TIR1-193^ autoactivity (**Figure 1B**). Truncation of the αE helix led to a significant decrease in RPV1^TIR^ autoactivity (**Figure 1B**), in agreement with similar results obtained with L6^TIR^ (Bernoux et al., 2011). However, whereas corresponding truncations in the αE helix of L6^TIR^ were found to affect protein stability, this was not the case for the truncated RPV1^TIR^ proteins (**Figure 1C**). These results strongly support the secondary-structure predictions, suggesting that RPV1^TIR^ is located between residues 20–188.

### Crystal Structure of RPV1 TIR Domain Reveals a Conserved Dimeric Interface

**FIGURE 2:**
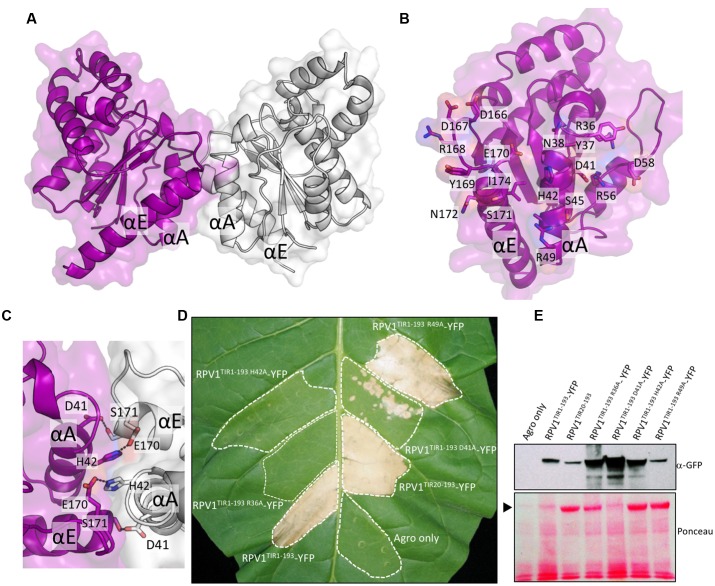
**RPV1^TIR^ cell-death signaling is dependent on the integrity of the conserved interface revealed by the RPV1^TIR20-193^ crystal structure.** **(A)** Structure of the dimer of RPV1 observed in the asymmetric unit of the crystals, shown in cartoon representation with transparent surface (chain A and B are colored purple and gray, respectively). **(B)** The dimer interface with chain A facing the plane of the page. Buried residues are displayed in stick representation and labeled. **(C)** The conserved histidine stacking within the interface. **(D)** *N. tabacum* plants 5 days after infiltration with *A. tumefaciens* strains expressing the RPV1^TIR1-193^ and mutants fused to YFP, and RPV1^TIR20-193^ analogous to the protein used for structural analysis. **(E)** Immunoblot detection of RPV1^TIR^-YFP fusions with anti-GFP antibodies, 2 days after agroinfiltration into *N. tabacum* leaves. Ponceau staining of the membrane used for western analysis, with the large RuBisCO subunit identified with an arrow. Agro only, corresponds to agrobacterium transformed with a vector without an insert.

On the basis of these results, we expressed the N-terminal residues 20–193 of RPV1 (designated RPV1^TIR20-193^) in *E. coli* and purified it to homogeneity. Crystals of RPV1^TIR20-193^ diffracted x-rays to 2.3 Å resolution and the structure was solved by molecular replacement (**Table 1**, **Figure 2**). The RPV1^TIR20-193^ structure resembles closely the AtTIR (PDB ID 3jrn), L6^TIR^ (3ozi) and RPS4^TIR^ (4c6r; Chan et al., 2010; Bernoux et al., 2011; Williams et al., 2014) structures with an overall Cα RMSD (root-mean-square-distance) value of ∼1.2, ∼1.3, ∼1.6 Å (for 141, 147, 148 superimposed residues), respectively. Within the asymmetric unit, we observe a dimer (**Figure 2A**) that resembles the heterodimer of the TIR domains from *Arabidopsis* RRS1 and RPS4 (Williams et al., 2014). This interaction involves the αA and αE helices and the loop regions that precede both helices (**Figure 2A**); we consequently define this protein-protein interface as the AE interface. Analysis of the RPV1^TIR^ structure using the program PISA (Krissinel and Henrick, 2007) identified that the AE interface was the largest crystal lattice contact, contributing a combined buried surface area of ∼1340 Å^2^ (∼670 Å^2^ from each molecule). This is similar to the combined ∼1300 Å^2^ buried surface in the RRS1^TIR^:RPS4^TIR^ heterodimer (Williams et al., 2014). Within the AE interface, 17 surface-exposed amino-acids from each RPV1^TIR20-193^ monomer are buried within the dimer and contribute to the interaction (**Figure 2B**). At the core of the interface, His42 forms an important stacking interaction between the two monomers and hydrogen bonds with Glu170 from the opposing monomer (**Figure 2C**). Hydrogen bonding between Asp41 and Ser171 is also prominent and these equivalent residues were also important in the RPS4^TIR^:RRS1^TIR^ interaction (Williams et al., 2014).

**Table 1:** Crystallographic data.

	RPV1^TIR20-193^
**Data collection**	
Space group	P 2 2_1_ 2_1_
a, b, c (Å)	41.855 89.117 113.858
α, β, γ (°)	90 90 90
Resolution (Å)	37.95-2.3 (2.38-2.3)^a^
Rmeas (%)^b^	0.098 (0.997)
Rpim (%)^c^	0.036 (0.366)
<I/σ(I)>	19.6 (2.2)
CC_1/2_^d^	0.99 (0.78)
Completeness (%)	100 (100)
Multiplicity	7.2 (7.4)
Wilson plot B (Å^2^)	27.5
Observations	142338 (13863)
Unique reflections	19672 (1881)
**Refinement**	
Rwork (%)	17.9
Rfree (%)	23.5
Average B-factor (Å^2^)	48.9
R.m.s deviations
Bond lengths (Å)	0.008
Bond angles (°)	1.03
Ramachandran plot (%)^e^
Favored	99.1
Allowed	0.9
Outliers	0


*^a^Values within parentheses indicate the highest resolution bin.*

*^b^$R_{meas}=\Sigma_{hkl}{\left\{N(hkl)/[N(hkl)-1\right\}}^{1/2}\,\;\Sigma_{i}|l_{i}(hkl)-<l(hkl)>|/\Sigma_{hkl}\,\;\Sigma_{i}l_{i}\,(hkl),$, where I_i_(hkl) is the intensity of the ith measurement of an equivalent reflection with indices hkl.*

*^c^$R_{pim}=\Sigma_{hkl}{\left\{1/\left[N(hkl)-1\right]\right\}}^{1/2}\,\;\Sigma_{i}|l_{i}(hkl)-<l(hkl)>|/\Sigma_{hkl}\,\;\Sigma_{i}l_{i}\,\left(hkl\right)$.*

*^d^Calculated with the program Aimless (Evans and Murshudov, 2013). ^e^As calculated by MolProbity (Chen et al., 2010).*

### RPV1 TIR Domain-Mediated Cell Death Is Dependent on the Conserved AE Interface

Based on the analysis of the crystal structure of RPV1^TIR20-193^, we were interested to understand further the potential role of the AE interface in RPV1 function. To do this, we generated mutations to specific residues in the core and peripheral regions of the AE interface of RPV1^TIR1-193^ (fused to YFP) and assessed the impact of these mutations on autoactivity. Mutations of residues His42 and Asp41 to alanine within the core of the AE interface, were found to abolish and markedly reduce, RPV1^TIR1-193^-YFP mediated cell death in tobacco. Mutation of R49 to alanine at the periphery of the interface had no effect on RPV1^TIR1-193^ autoactivity (**Figure 2D**), while mutation of R36 to alanine, which is also on the periphery of the interface, abolished cell death.

Importantly, these results could not be explained by differences in protein stability. Indeed, levels of RPV1^TIR1-193^-YFP protein recovered from tobacco-leaf tissue agroinfiltrated for the loss-of-function mutants (R36A, D41A and H42A) appeared higher, 48 h post-infiltration, than from the constructs that displayed strong necrosis at day 5 (**Figure 2E**), potentially due to the impact of cell death on protein yield from agroinfiltrated sectors. These results demonstrate that residues R36, D41, and H42 within the AE interface play a key role in the RPV1^TIR^ signaling.

### RPV1 TIR Domain-Mediated Cell Death Is Dependent on Regions Outside the AE Interface

**FIGURE 3:**
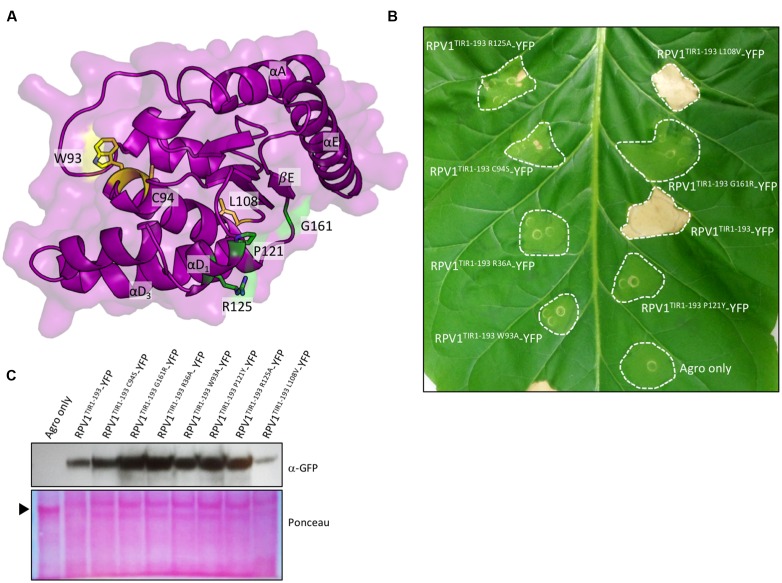
**RPV1^TIR^ cell-death signaling is dependent on regions outside of the AE interface.** **(A)** Structure of the RPV1^TIR20-193^, highlighting residues mutated in regions outside of the AE interface. Residues colored green represent residues important in L6^TIR^ self-association and autoactivity, while residues colored yellow represent residues outside of this region and the AE interface. **(B)** *N. tabacum* plants 5 days after infiltration with *A. tumefaciens* strains expressing the RPV1^TIR1-193^ and mutants fused to YFP. RPV1^TIR1-193^ and RPV1^TIR1-193R36A^ are included as positive and negative controls. **(C)** Immunoblot detection of RPV1^TIR^-YFP fusions with anti-GFP antibodies, 2 days after agroinfiltration into *N. tabacum* leaves. The arrow indicates Ponceau staining of the large RuBisCO subunit.

The crystal structure of L6^TIR^ revealed an interface that is spatially distinct to the AE interface, involving the αD and αE helices and the βE strand. In L6^TIR^, this region was shown to be important in mediating L6^TIR^ self-association, which is a requirement for the cell death signaling function of the L6^TIR^ (Bernoux et al., 2011). To investigate if residues in this region affect RPV1^TIR^ signaling, we generated mutations P121Y, R125A and G161R (**Figure 3A**); equivalent to P160Y, R164A and G201R in L6^TIR^, and found that these mutants were compromised in their ability to mediate cell death in tobacco (**Figure 3B**). In the case of L6^TIR^, cell-death signaling was shown to be compromised when residues outside the L6^TIR^ self-association interface were mutated (Bernoux et al., 2011). We also observed disruption of cell-death signaling in W94A and C95S RPV1^TIR1-193^-YFP constructs (equivalent to W131A and C132S in L6^TIR^), consistent with findings for L6. Importantly not all mutations to surface-exposed residues disrupted cell-death signaling, as demonstrated by the fact that mutation of the non-conserved L108 to a valine did not affect the protein (**Figures 3A,B**). All mutated RPV1^TIR1-193^YFP proteins were detectable by western-blot analysis (**Figure 3C**), suggesting that these results are not influenced by *in planta* protein stability.

### Solution Studies of the RPV1 TIR Domain

**FIGURE 4:**
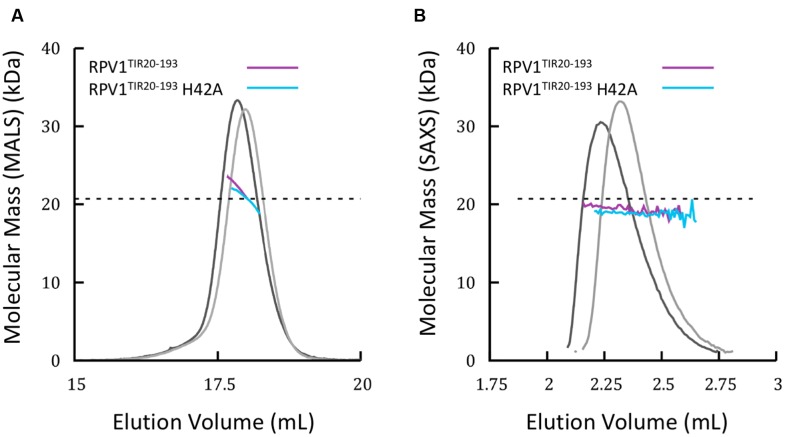
**Solution properties of RPV1^TIR20-193^** **(A)** Size-exclusion chromatography (SEC)/multi-angle laser light scattering (MALS) analysis of RPV1^TIR20-193^ (purple) and RPV1^TIR20-193^H42A (sky blue). Purified proteins were separated over an inline Superdex 200 10/300 GL column and the molecular mass (MM) was calculated across the elution peak by MALS. Colored lines under the peaks correspond to the averaged MM (*y*-axis) distributions across that peak, while gray lines indicate the normalized refractive index trace (nominal units). A dotted black line denotes the expected monomeric molecular mass of RPV1^TIR20-193^. **(B)** SEC/small-angle x-ray scattering (SAXS) analysis of RPV1^TIR20-193^ and RPV1^TIR20-193^H42A. Purified proteins were separated on an in-line Superdex Increase 200 5/150 GL SEC column. MM was calculated using the volume of correlation (Rambo and Tainer, 2013). Colored lines under the peak correspond to the molecular mass (*y*-axis) across the peak. Gray lines indicate the zero angle scattering, *I*(0), arbitrarily scaled onto the molecular mass for visualization.

Homo- and hetero-meric TIR:TIR domain interactions are responsible for the biological functions of TIR domains (Ve et al., 2014). Interactions have been observed in the TIR domains from RRS1, RPS4, and L6 by both Y2H assays and by studies in solution with recombinant proteins (Bernoux et al., 2011; Williams et al., 2014). For RPV1^TIR1-193^, we did not observe an interaction in Y2H assays (**Supplementary Figure S3**) under the conditions tested, despite observing self-association of L6^TIR29-233^, which was consistent with previous observations (Bernoux et al., 2011). Solution studies using SEC-coupled MALS and SAXS suggested that only limited self-interaction of RPV1^TIR20-193^ may be present (**Figure 4**). The average SEC-MALS-derived molecular mass of RPV1^TIR20-193^ was 22.3 kDa, while the theoretical molecular mass of RPV1^TIR20-193^ is 20.7 kDa. We also tested, by SEC-MALS, the recombinant RPV1^TIR20-193^ protein carrying an alanine mutation at position H42 (RPV1^TIR20-193H42A^). The equivalent mutation in RPS4^TIR^ had previously been shown to disrupt RPS4^TIR^:RRS1^TIR^ dimerisation and inhibited RPS4^TIR^ self-association. This analysis produced a molecular mass of 20.8 kDa, slightly lower than that of RPV1^TIR20-193^ (**Figure 4A**). Using SEC-SAXS, the averaged molecular masses observed for both proteins were lower than masses determined by SEC-MALS, 19.6 kDa for RPV1^TIR20-193^ and 18.7 kDa for RPV1^TIR20-193H42A^, and small shifts in elution time between RPV1^TIR20-193^ and RPV1^TIR20-193H42A^ were also observed (**Figure 4B**). Molecular masses higher than monomer were previously observed in L6, RPS4, and RRS1 by SEC-MALS (Bernoux et al., 2011; Williams et al., 2014) and in these cases, this behavior could be convincingly linked to self-association. However, in the case of RPV1^TIR^, the absolute difference in molecular mass is small and may be beyond the sensitivity of these scattering techniques. Therefore, while these data do not exclude the possibility that the RPV1^TIR^ domain can self-associate, a link to this function cannot be made based on the Y2H and in-solution experiments presented here.

### Conservation of a Functional Interface in Plants

**FIGURE 5:**
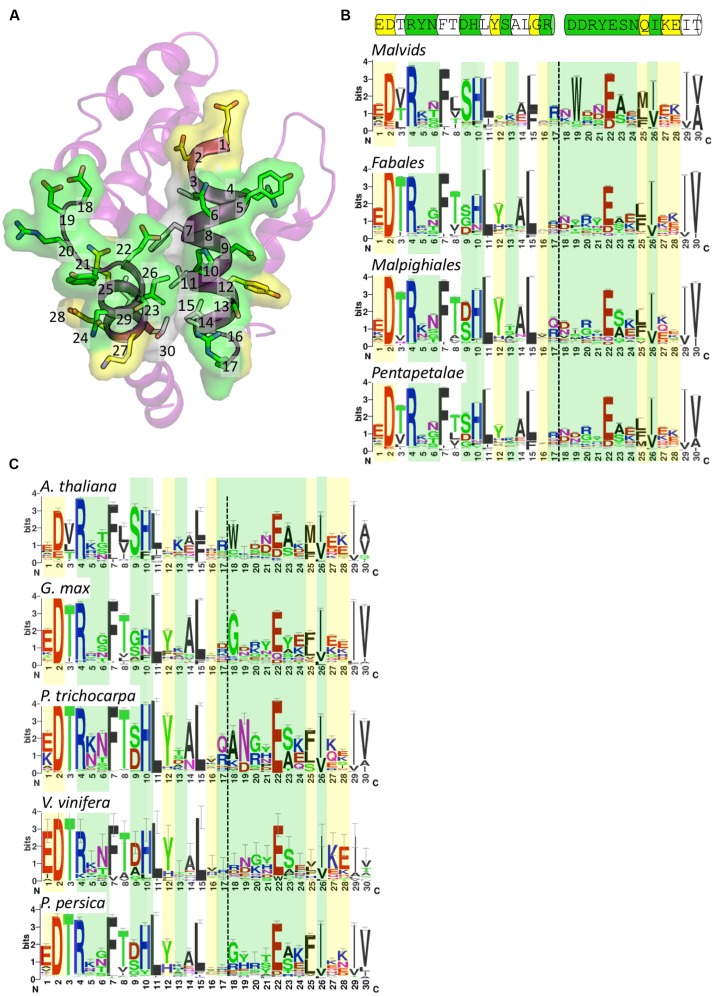
**Conservation of surface-exposed residues within the AE interface.** **(A)** Crystal structure of RPV1 shown in ribbon representation with the face of the AE interface oriented toward the page. The AE interface residues are presented as sticks and are included in the sequence logos presented in **(B,C)**. The numbering corresponds to position in the logos. Surface-exposed residues involved in interface interactions are colored green, surface-exposed residues not involved in interface interactions are colored yellow, and residues that are not surface-exposed are colored white. **(B)** Sequence logos of the AE interface in the families Malvids, Fabales, Malipighiales and Pentapetalae. Coloring as defined for **(A)**. Dotted lines represent the division between the αA and αE regions. Cartoon above the logos represents the sequence of this region in RPV1 as shown in **(A)**. **(C)** Sequence logos of the AE interface in the species *A. thaliana, G. max, P. trichocarpa, V. vinifera*, and *P. persica* colored and labeled as in **(B)**.

We previously highlighted the potential conservation of surface-exposed residues in the AE interface after defining its role in RPS4 and RRS1 defense signaling (Williams et al., 2014). In light of our findings presented here for RPV1, we embarked on a more extensive analysis to characterize the conservation of the AE interface across a broader range of plant species. We created species-specific profiles to capture >2000 TIR domain-containing sequences from across 29 plant species and used profile HMMs to create and inspect TIR-domain sequence logos across this wide species range with a focus on the AE interface (See Supplementary Table S2, Supplementary Data Sheet 1, and Materials and Methods). Sequences identified from Pfam were collated into four clades for comparison based on the species tree from Phytozome (**Supplementary Figure S2**): Malvids, containing *A. thaliana*, *A. lyrata*, *Brassica rapa*, and *Eutrema salsugineum*; Fabales, containing *G. max* and *P. persica*; Malpighiales, containing *P. trichocarpa* and *Ricinus communis*; and Pentapetalae, that contained all of the three previous sets, plus *Solanum lycopersicum*, *S. tuberosum*, and *V. vinifera*—representing all classified TIR domain sequences from Pfam within the Pentapetalae clade (**Figure 5B**). We also undertook a more specific analysis into five individual species to highlight residue differences occurring at the species level (**Figure 5C**).

Conservation of residues across a wide range of species in the αA and αE regions showed that the αA region (positions 1–17) was more conserved than the αE region (positions 18–30). A number of hydrophobic residues are highly conserved, including positions 7, 11, 15, 26, 29, and 30 (**Figures 5A–C**). These residues are mostly buried and appear to assist in stabilizing the positioning of the αA and αE helices. The well-conserved position 26 (RPV1 Ile174) is surface-exposed and contributes to a pocket that in the RPV1^TIR20-193^ structure is occupied by His42 from the interacting RPV1^TIR20-193^ protomer. The most important surface-exposed residues in the AE interface are at positions 9, 10, and 22, which form the core of the AE interface (**Figures 5A** and **2C**). This analysis reveals that at these positions, there is conservation of histidine and glutamate at position 10 and 22, respectively (RPV1 His42 and Glu170). The amino-acid distribution at position 9 (RPV1 Asp41) is more varied across the clades (**Figure 5B**), with evidence of species-specific conservation, for example the conservation of serine in *A. thaliana* (**Figure 5C**). There is also a general trend for position 23 to contain a small amino-acid such as alanine or serine. While histidine at position 10 is well conserved, there is minor representation of aromatic residues including phenylalanine and tyrosine. Interestingly, in the case of AtTIR, a phenylalanine occupies this position, indicating that phenylalanine is compatible with dimerisation through the AE interface, at least within the context of AtTIR crystals. At the species level, *G. max* (soybean) appeared to deviate most significantly of the species compared across the important AE interface residues. *G. max* appears to have a similar preference for histidine and asparagine at position 10 (**Figure 5C**) and at position 22, the negatively charged residue glutamate is replaced by aspartate and glutamine in some instances. In general, there is significant variation in other interface interacting residues at both the clade and species levels, with the exception of position 4, which is essentially an invariant arginine.

## Discussion

Toll-interleukin receptor domains are protein scaffolds that regulate pathogen defense-related pathways in plants and animals through TIR:TIR domain interactions (Ve et al., 2014). Of the five structures currently available for plant TIR domains, four originate from *Arabidopsis* and one from flax. All the crystal structures of TIR domains from *Arabidopsis* proteins show an analogous interaction interface (designated here the AE interface). We previously hypothesized that this interface would have broad functional relevance, beyond signaling in *Arabidopsis* (Williams et al., 2014). Here, our crystal structure of the *M. rotundifolia* RPV1^TIR20-193^ domain reveals a dimer interaction that is mediated by the AE interface. We demonstrate that the conserved surface-exposed residues in the AE interface are required for the autoactive cell-death phenotype exhibited by the TIR domain of RPV1 when transiently expressed in *N. tabacum*. In addition, we demonstrate that the integrity of residues that map to the L6^TIR^ self-association interface and residues that are outside of both interfaces are also required for the autoactive cell-death in *N. tabacum*. These observations combined with the conservation of this region across a broad range of plant species strongly suggest that this protein interface plays a general role in plant TIR-domain signaling.

The presence of an interface within a crystal structure does not, in itself, support a functional role for this interface (Kobe et al., 2008). However, the presence of a dimer mediated by the analogous AE interface in five of the six available plant TIR domain crystal structures is striking (**Supplementary Figure S4**). We have previously demonstrated that the AE interface is biologically relevant. The AE interface is responsible for the strong heterodimeric interaction between RPS4^TIR^ and RRS1^TIR^, and also that self-association of the RPS4^TIR^, suggesting that competition between these two interactions is important for the regulation in the full-length NLR (Williams et al., 2014). In the case of RPV1^TIR20-193^, biophysical characterisation suggests that self-association, is absent or very weak in solution. SEC-MALS yielded an averaged molecular mass only slightly higher than that predicted for a monomer and while the interface-disrupting mutant RPV1TIR^20-193^H42A did restore the theoretical monomeric molecular mass of RPV1 in SEC-MALS, the difference between the mutant and wild-type protein is less than 10% of the mass and therefore within potential error associated with this technique. Consequently, from the experiments used we were unable to confirm self-association of RPV1^TIR^. Despite this, it is clear that the AE interface plays a crucial role in autoactivity of the RPV1 TIR domain. Mutations that alter the surface characteristics in the core of the AE interface were found to significantly reduce cell-death signaling *in planta*, demonstrating that the interface is functionally relevant in RPV1 TIR-domain cell-death signaling. This disruption is unlikely to be due to misfolding, as these proteins were expressed *in planta*. We also show that regions outside the AE interface are important for this cell-death signaling. RPV1^TIR^ autoactivity is also prevented by mutations of conserved residues within the interface identified in L6^TIR^ (the DE interface) to be required for self-association and signaling. Interestingly, a similar observation was recently made in studies of the *Arabidopsis* NLR protein RPP1, whereby residues in both the AE and DE interfaces played were important for RPP1 TIR domain mediated cell death signaling (Schreiber et al., 2016).

An interesting feature of the cell death induced by RPV1 is that the region that encompasses the TIR domain structure is, itself, sufficient to induce cell death. To the best of our knowledge, this is the first demonstration that the TIR domain alone can signal autoactive cell death. In previous studies, amino-acid sequences that extended beyond the structural borders of the TIR domain were included when assaying for autoactivity *in planta*. For example, the available L6^TIR^ structure encompasses residues 59–228; however, residues 1–233 were used in functional experiments to demonstrate cell death (Bernoux et al., 2011). Similarly, the available RPS4^TIR^ structure includes residues 10–178; however, the minimal cell death-inducing construct used in functional experiments comprised residues 1–235 (Williams et al., 2014). Furthermore, in the case of NdA allele of RPP1, autoactive cell death was induced in *N. tabacum* when the first 254 residues were used, while the TIR domain alone (residues 90–254) did not induce this phenotype (Schreiber et al., 2016). In all these cases, the integrity of residues in the structured region of the TIR domain was established to be critical for the cell-death function, demonstrating that the TIR domain was required for cell death. Also, in all cases, a strong correlation between self-association (measured *in vitro*) and cell-death function was observed.

The RPV1 TIR domain represents the first instance of a lack of obvious correlation between self-association (measured *in vitro*) and *in planta* autoactive cell-death function. Previous studies involving the WsB allele of RPP1 (residues 1–266) demonstrated that the weak dimerisation propensity of the C-terminal GFP tag was required to cause autoactive cell death (Krasileva et al., 2010). In the case of RPV1^TIR^, the inclusion of the YFP had no consequence on the autoactive cell-death phenotype (**Figure 1**). It is plausible that RPV1^TIR^ self-association is stabilized by other proteins when overexpressed in *N. tabacum*; however, it is also possible that RPV1^TIR^ can interact with other TIR-only, TIR-NLR proteins in *N. tabacum* or a yet to be identified signaling protein in order to initiate cell-death. Regardless of the mechanisms of cell-death activation, the integrity of the AE interface is required.

Our comparison of the AE interfaces across plant species shows that surface-exposed residues that occupy the central region of the interface are highly conserved, while residues further away from the center are generally variable. We suggest that this gives the AE interface a conserved core to facilitate interactions, while the variable exterior residues control the specificity of TIR:TIR domain interactions. An exception to this is the highly conserved arginine at position 4 (**Figure 5**), which is at the periphery of the AE interface. Mutation of this residue to in alanine in RPV1^TIR^ prevented TIR domain dependent cell death, which was also observed previously for L6^TIR^ and RPS4^TIR^ (Swiderski et al., 2009; Bernoux et al., 2011). While this residue has only a minor involvement in the AE interface, it does appear to associate with a slight kinking of the αA helix in the known plant TIR-domain structures and likely influences the positing of the αA helix. The comparison between species also highlights that the functionally important histidine is highly preferred in *Arabidopsis* (*A. thaliana*), poplar (*P. trichocarpa*), grape (*V. vinifera*) and peach (*P. persica*) but less so in soybean (*G. max*). It will be interesting to characterize further these divergent residues at conserved positions in terms of TIR domain autoacitivity and NLR function in the future.

Overall, the data presented here highlights that the AE interface is likely to be functionally important across a broad range of plant species and is not limited to *Arabidopsis* TIR domains. Our work also confirms that the TIR domain is the minimal region required for cell-death signaling. We suggest the available data build a general paradigm of structure-function relationships in plant TIR domains, where the AE interface and other spatially distinct protein surfaces in plant TIR domains play key roles in the signaling mechanism of plant TIR-NLR proteins.

## Author Contributions

SW, BK, ID, MB, LY, and GF conceived and designed the experiments, SW, LY, GF, LC, MO, and DE performed the experiments, all authors analyzed the data and SW, LY, BK, ID, GF, and LC wrote the paper. All authors reviewed and edited the paper.

## Conflict of Interest Statement

The authors declare that the research was conducted in the absence of any commercial or financial relationships that could be construed as a potential conflict of interest.

## Footnotes

1 http://www.synchrotron.org.au/index.php/aussyncbeamlines/saxswaxs/software-saxswaxs

## Supplementary Material

**FIGURE S1 | Workflow diagram.** Key steps involved in the sequence-level analysis of conservation.

**FIGURE S2 | Species tree representing species and clades investigated in the sequence-level analysis of conservation.** Colored triangles indicate individual species retrieved from Pfam that comprised the four clades used to build the profile HMMs. Colored circles on the tree indicate the subtree from Phytozome that was queried with the profile HMM built from the equivalently colored triangle. Yellow squares represent the individual species used to build species-level profile HMMs to query the respective species from Phytozome.

**FIGURE S3 | Analysis of TIR dimerization in Y2H assays.** Growth of yeast cells co-expressing GAL4-BD and GAL4-AD empty vector (Negative), RPV1^TIR^ and L6^TIR^ domain fusions on synthetic media lacking tryptophan and leucine (-TL) or on selective media lacking tryptophan, leucine and histidine and containing 5 mM 3AT. Growth on media lacking tryptophan and leucine (-TL) confirms yeast viability, while growth on media lacking histidine (-HTL) indicates expression of the HIS3 reporter gene due to interaction between the fusion proteins.

**FIGURE S4 | Published TIR-domain structures that feature the AE interface.** **(A)** RPV1 (PDB ID 5KU7) chain A and B. **(B)** RRS1-RPS4 heterodimer (PDB ID 4c6t) chain A and B. **(C)** RPS4 (PDB ID 4c6r) chain A and D (symmetry operator *x*, *y*-1, *z*). **(D)** RRS1 (PDB ID 4c6s) chain A and A (symmetry operator *y*+1, -*x*+1, -*z*+5/2). **(E)** AtTIR (PDB ID 3jrn) chain A and A (symmetry operator -*y*+1, -*x*+1, -*z*+1/2). **(F)** L6 (PDB ID 3OZI) chain A (yellow) and B (wheat) mediated by αD and αE helices and orientated as in **(A–E)** for comparison.
