# Supplementary figures and images for: Structure and Function of the TIR Domain from the Grape NLR Protein RPV1
Source: Front Plant Sci. 2016 Dec 8;7:1850. doi: 10.3389/fpls.2016.01850 (PMC5143477; doi:10.3389/fpls.2016.01850)

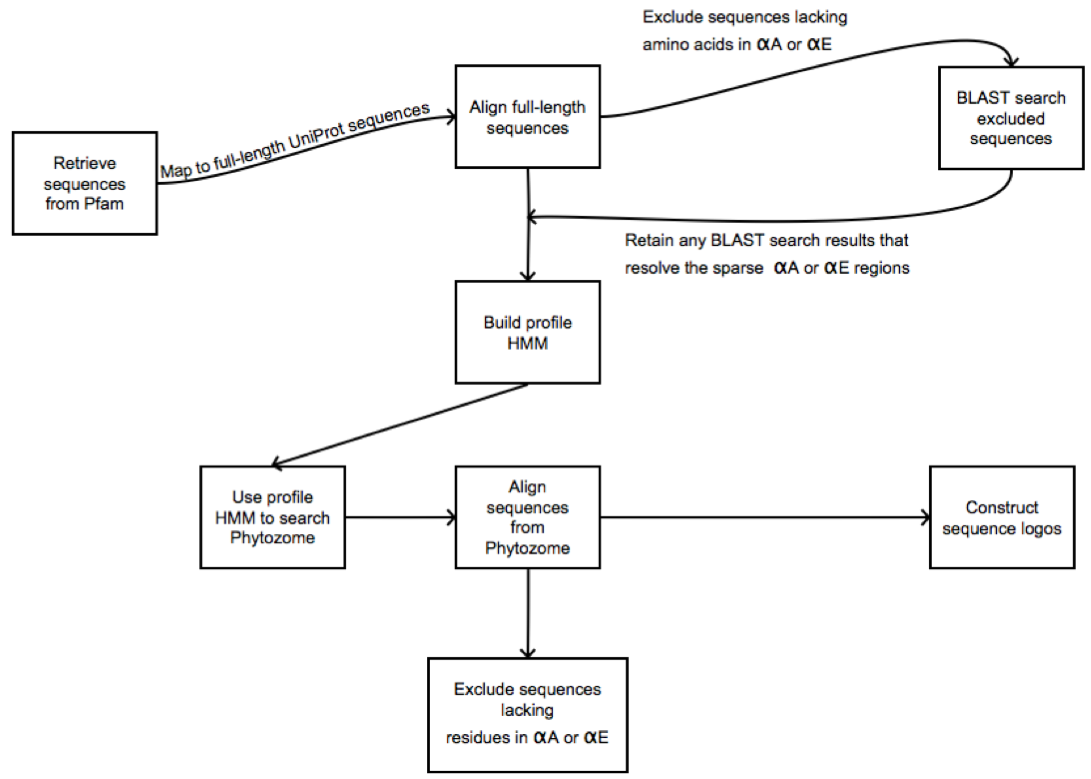

Supplement: Supplementary file 1 [file Image_1.TIFF]

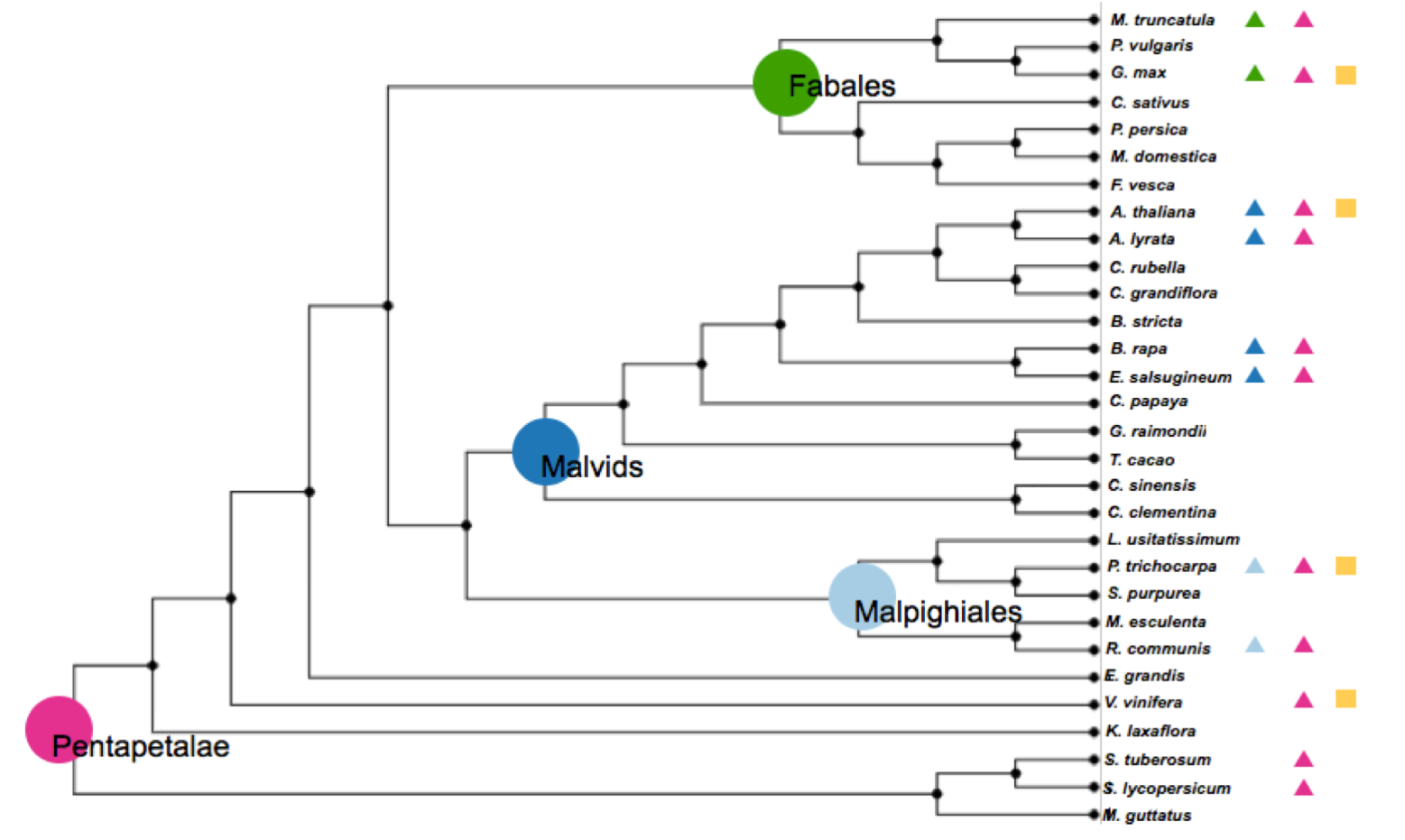

Supplement: Supplementary file 2 [file Image_2.TIFF]

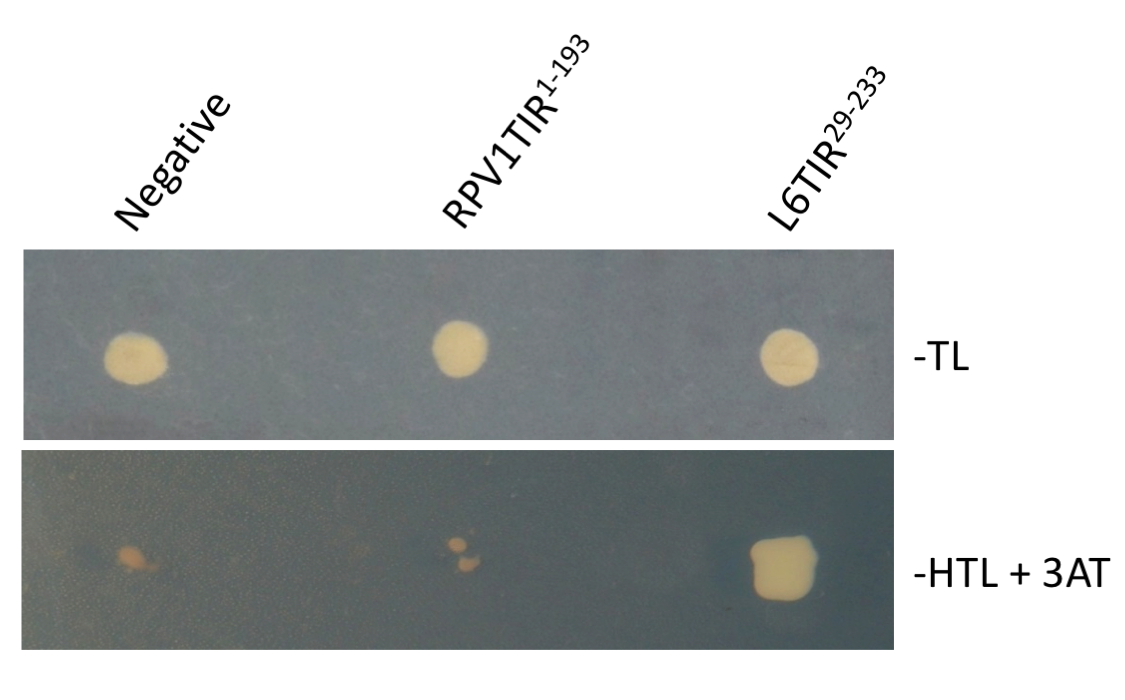

Supplement: Supplementary file 3 [file Image_3.TIFF]

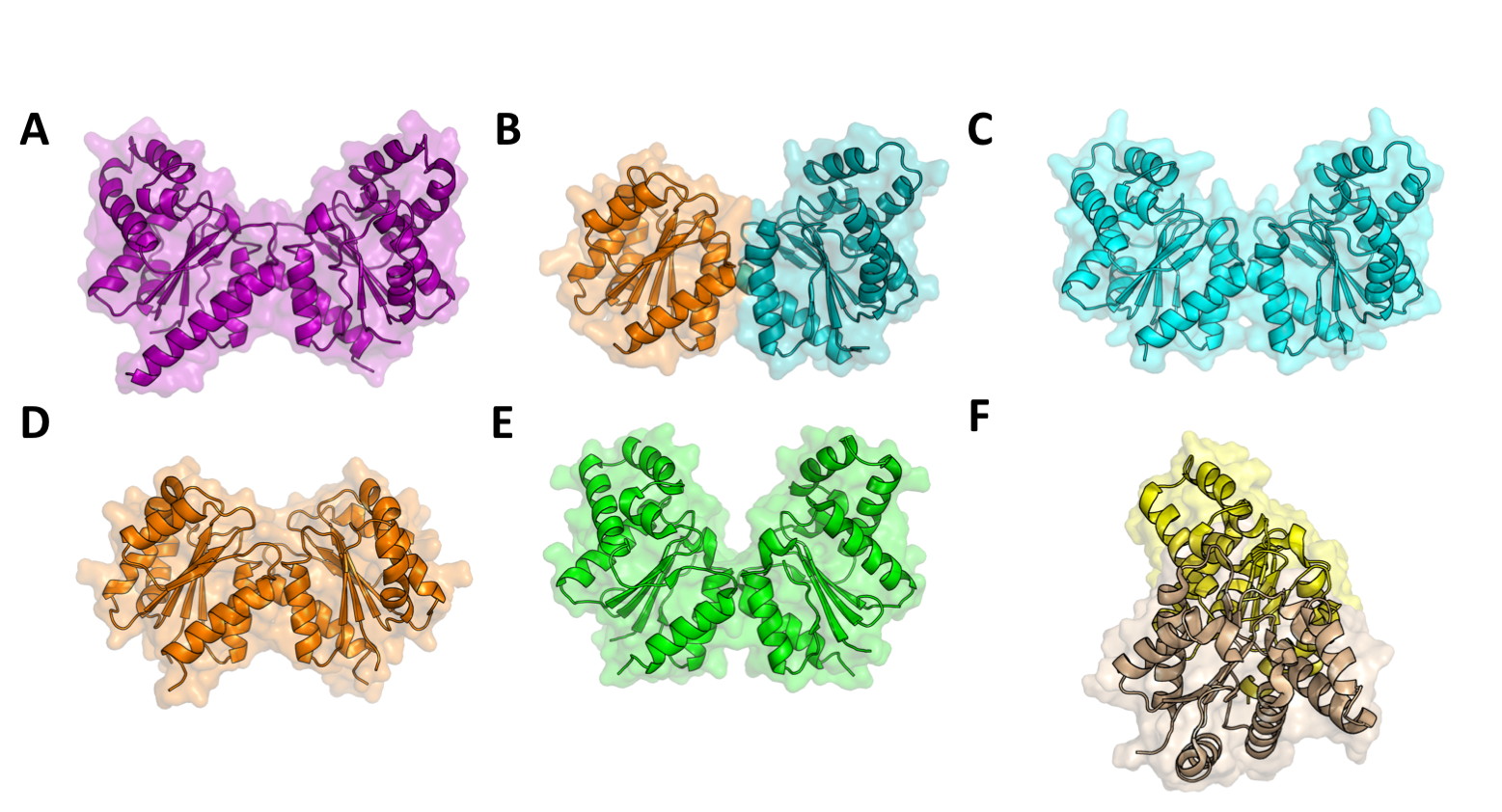

Supplement: Supplementary file 4 [file Image_4.TIFF]
