# Supplementary material for: Structure and Function of the TIR Domain from the Grape NLR Protein RPV1
Source: Front Plant Sci. 2016 Dec 8;7:1850. doi: 10.3389/fpls.2016.01850 (PMC5143477; doi:10.3389/fpls.2016.01850)
Supplement: Supplementary file 5 [file Table_1.DOCX]

**Supplementary Table 1:** Primer sequences

| **Primer** | **Application** | **5'-3' sequence (Gateway sequence underlined)** |
| --- | --- | --- |
| attB1-RPV1TIR1-F | Amplify from RPV1TIR start codon | GGGGACAAGTTTGTACAAAAAAGCAGGCTAGAATGGCTTCCACAAGCTCCTTC |
| attB1-RPV1TIR20-F | Amplify RPV1TIR - N-term with first 20 aa missing | GGGGACAAGTTTGTACAAAAAAGCAGGCTATGGCTCGAACAACTACTTATGATGTTTTC |
| attB1-RPV1TIR193-STOP-R | RPV1TIR1-193 with added stop codon | GGGGACCACTTTGTACAAGAAAGCTGGGTTCAACCAACATCAAGCCTCTTACA |
| attB1-RPV1TIR1-F-Y2H | In frame N-terminal fusion in Y2H construct | GGGGACAAGTTTGTACAAAAAAGCAGGCTTCATGGCTTCCACAAGCTCCTTC |
| attB1-RPV1TIR193-R | RPV1TIR^1-193^-YFP fusion construct | GGGGACCACTTTGTACAAGAAAGCTGGGTCACCAACATCAAGCCTCTTACATTT |
| attB1-RPV1TIR189-R | RPV1TIR^1-189^-YFP fusion construct | GGGGACCACTTTGTACAAGAAAGCTGGGTCCCTCTTACATTTCAATTGACGAAAT |
| attB1-RPV1TIR183-R | RPV1TIR^1-183^-YFP fusion construct | GGGGACCACTTTGTACAAGAAAGCTGGGTCACGAAATATGCTATTAGTTATTTCTTTAAT |
| attB1-RPV1TIR174-R | RPV1TIR^1-174^-YFP fusion construct | GGGGACCACTTTGTACAAGAAAGCTGGGTCAATTTGATTGGACTCGTACCGGT |
| attB1-RPV1TIR169-R | RPV1TIR^1-169^-YFP fusion construct | GGGGACCACTTTGTACAAGAAAGCTGGGTCGTACCGGTCATCTAGTAGATGC |
| RPV1TIR1-193_R36A-F | Introduce R36A mutation into RPV1TIR^1-193^ | GTTTTAGAGGCGAAGACACCGCCTATAATTTCACTGATCACCT |
| RPV1TIR1-193_R36A-R | Introduce R36A mutation into RPV1TIR^1-193^ | AGGTGATCAGTGAAATTATAGGCGGTGTCTTCGCCTCTAAAAC |
| RPV1TIR1-193_D41A-F | Introduce D41A mutation into RPV1TIR^1-193^ | CCGCTATAATTTCACTGCTCACCTCTACAGTGCC |
| RPV1TIR1-193_D41A-R | Introduce D41A mutation into RPV1TIR^1-193^ | GGCACTGTAGAGGTGAGCAGTGAAATTATAGCGG |
| RPV1TIR1-193_H42A-F | Introduce H42A mutation into RPV1TIR^1-193^ | GACACCCGCTATAATTTCACTGATGCATTGTACAGTGCCTTGGGTAGGAGAG |
| RPV1TIR1-193_H42A-R | Introduce H42A mutation into RPV1TIR^1-193^ | CTCTCCTACCCAAGGCACTGTACAATGCATCAGTGAAATTATAGCGGGTGTC |
| RPV1TIR1-193_R49A-F | Introduce R49A mutation into RPV1TIR^1-193^ | CCTCTACAGTGCCTTGGGTGCTAGAGGGATTCGCACCTTC |
| RPV1TIR1-193_R49A-R | Introduce R49A mutation into RPV1TIR^1-193^ | GAAGGTGCGAATCCCTCTAGCACCCAAGGCACTGTAGAGG |
| RPV1TIR1-193_W93A-F | Introduce W93A mutation into RPV1TIR^1-193^ | GAAAACTATGCTCATTCGAGAGCGTGTTTGGATGAGTTGGTAAAG |
| RPV1TIR1-193_W93A-R | Introduce W93 mutation into RPV1TIR^1-193^ | CTTTACCAACTCATCCAAACACGCTCTCGAATGAGCATAGTTTTC |
| RPV1TIR1-193_C94S-F | Introduce C94S mutation into RPV1TIR1-193 | ACTATGCTCATTCGAGATGGTCTTTGGATGAGTTGGTAAAGATC |
| RPV1TIR1-193_C94S-R | Introduce C94S mutation into RPV1TIR1-193 | GATCTTTACCAACTCATCCAAAGACCATCTCGAATGAGCATAGT |
| RPV1TIR1-193_L108V-F | Introduce L108V mutation into RPV1TIR1-193 | GGAGTGCCAGAAAGATGTGGGACATGCTGTTTTCC |
| RPV1TIR1-193_L108V-R | Introduce L108V mutation into RPV1TIR1-193 | GGAAAACAGCATGTCCCACATCTTTCTGGCACTCC |
| RPV1TIR1-193_P121Y-F | Introduce P121Y mutation into RPV1TIR1-193 | TCCCAATTTTCTATCATGTGGATTACTCCCATGTACGAAAGCAAGAAGG |
| RPV1TIR1-193_P121Y-R | Introduce P121Y mutation into RPV1TIR1-193 | CCTTCTTGCTTTCGTACATGGGAGTAATCCACATGATAGAAAATTGGGA |
| RPV1TIR1-193_R125A-F | Introduce R125A mutation into RPV1TIR1-193 | ATGTGGATCCATCCCATGTAGCAAAGCAAGAAGGAAGTTTTG |
| RPV1TIR1-193_R125A-R | Introduce R125A mutation into RPV1TIR1-193 | CAAAACTTCCTTCTTGCTTTGCTACATGGGATGGATCCACAT |
| RPV1TIR1-193_G161R-F | Introduce G161R mutation into RPV1TIR1-193 | GAAGCAGCCAATCTTTCTCGATGGCATCTACTAGATG |
| RPV1TIR1-193_G161R-R | Introduce G161R mutation into RPV1TIR1-193 | CATCTAGTAGATGCCATCGAGAAAGATTGGCTGCTTC |
| RPV1TIR20-F | Amplify RPV1TIR - N-term with first 20 aa missing for *E.coli* expression | TACTTCCAATCCAATGCGCGAACAACTACTTATGATGTTTTCTTGAGTTTTAGAG |
| RPV1TIR193-R | Amplify RPV1TIR - combined with RPV1_FW | TATTCCACTTCCAATGTTAACCAACATCAAGCCTCTTACATTTCAATTGACG |
