# Supplementary material for: Structure and Function of the TIR Domain from the Grape NLR Protein RPV1
Source: Front Plant Sci. 2016 Dec 8;7:1850. doi: 10.3389/fpls.2016.01850 (PMC5143477; doi:10.3389/fpls.2016.01850)
Supplement: Supplementary file 6 [file Table_2.DOCX]

**Supplementary Table 2:** Number of species at each stage of profile HMM search

| **Species from Pfam annotated with TIR domains. Numbers in brackets indicate number of protein sequences.** | **Number of sequences in multiple sequence alignment used to build profile HMM after alignment pruning and exclusion.** | **Species from Phytozome database. Numbers in brackets indicate number of protein sequences derived from translation of primary transcripts.** | **Number of sequences in multiple sequence alignment used to build logo after alignment pruning and exclusion.** |
| --- | --- | --- | --- |
| Set 1: Malvids - *A. lyrata* (169), *A. thaliana* (164), *B. rapa* (166), *E. salsugineum* (103) | 483 | *A. lyrata (32 670),* ***A. thaliana (27 416)****, B. rapa (40 492), Boechera*  *stricta (27 416), Citrus clementine (24 533), Capsella grandiflora (24 805), Carica. papaya (27 751), Capsella rubella (26 521), Citrus sinensis (25 379), E. salsugineum (26 351), Gossypium raimondii (37 505), Theobroma cacao (29 452)* | 931 |
| Set 2: Fabales - *G. max* (266), *M. truncatula* (256) | 388 | *Cucumis sativus (21 503), Fragaria vesca (32 831),* ***G. max (56 044)****, Malus domestica (63 514), M. truncatula (50 894), Prunus persica (26 873), Phaseolus vulgaris (27 197)* | 926 |
| Set 3: Malpighiales - *P. trichocarpa* (186), *R. communis* (42) | 130 | *Linum usitatissimum (43 471), Manihot esculenta (33 033),* ***P. trichocarpa (41 335)****, R. communis (31 221), Salix purpurea (37 865)* | 376 |
| Set 4: Pentapetalae - *A. lyrata* (169), *A. thaliana* (164), *B. rapa* (166), *E. salsugineum* (103), *G. max* (266), *M. truncatula* (256), *P. trichocarpa* (186), *R. communis* (42), *S. lycopersicum* (38), *S. tuberosum* (93), *V. vinifera* (77) | 1102 | *A. lyrata (32 670),* ***A. thaliana (27 416),*** *B. rapa (40 492), B. stricta (27 416), C. clementine (24 533), C. grandiflora (24 805), C. papaya (27 751), C. rubella (26 521), C. sativus (21 503), C. sinensis (25 379), Eucalyptus grandis (36 349), E. salsugineum (26 351), F.vesca (32 831),* ***G. max (56 044****), G. raimondii (37 505), L. usitatissimum (43 471), M. domestica (63 514), M. esculenta (33 033), Mimulus guttatus (28 140), M. truncatula (50 894), P.persica (26 873),* ***P. trichocarpa (41 335),*** *P. vulgaris (27 197), R. communis (31 221), S. lycopersicum (34 727), S. purpurea (37 865), S. tuberosum (35 119), T. cacao (29 452),* ***V. vinifera (26 346)*** | 2469 |
| Set 5: *A. thaliana* (164) | 141 | *A. thaliana (27 416)* | 120 |
| Set 6: *G. max* (266) | 197 | *G. max (56 044)* | 240 |
| Set 7: *P. trichocarpa* (186) | 93 | *P. trichocarpa (41 335)* | 161 |
| Set 8: *V. vinifera* (77) | 43 | *V. vinifera (26 346)* | 21 |
