# Supplementary material for: Structure and Function of the TIR Domain from the Grape NLR Protein RPV1
Source: Front Plant Sci. 2016 Dec 8;7:1850. doi: 10.3389/fpls.2016.01850 (PMC5143477; doi:10.3389/fpls.2016.01850)
Supplement: Supplementary file 7 [file Data_Sheet_1.DOCX]

**Supplementary Data 1: Aligned sequences from Pfam used to build profile HMMs and aligned sequences from Phytozome identified from profile HMMS.** Sequences retrieved from Pfam after alignment pruning and exclusion cropped to the αA and αE regions, including the residues between the two regions. Sequences identified from Phytozome cropped to the αA and αE regions, without the residues between the two regions.

Aligned sequences appear in the following order:

**Aligned Malvid sequences from Pfam used to build profile HMM after alignment pruning and exclusion.**

**Aligned Fabale sequences from Pfam used to build profile HMM after alignment pruning and exclusion.**

**Aligned Malpighiale sequences from Pfam used to build profile HMM after alignment pruning and exclusion.**

**Aligned Pentapetalae sequences from Pfam used to build profile HMM after alignment pruning and exclusion.**

**Aligned *A. thaliana* sequences from Pfam used to build profile HMM after alignment pruning and exclusion.**

**Aligned *G. max* sequences from Pfam used to build profile HMM after alignment pruning and exclusion.**

**Aligned *P. trichocarpa* sequences from Pfam used to build profile HMM after alignment pruning and exclusion.**

**Aligned *V. vinifera* sequences from Pfam used to build profile HMM after alignment pruning and exclusion.**

**Aligned Malvid sequences from Phytozome primary transcript database.**

**Aligned Fabale sequences from Phytozome primary transcript database.**

**Aligned Malpighiale sequences from Phytozome primary transcript database.**

**Aligned Pentapetalae sequences from Phytozome primary transcript database.**

**Aligned *A. thaliana* sequences from Phytozome primary transcript database.**

**Aligned *G. max* sequences from Phytozome primary transcript database.**

**Aligned *P. trichocarpa* sequences from Phytozome primary transcript database.**

**Aligned *V. vinifera* sequences from Phytozome primary transcript database.**

**Aligned Malvid sequences from Pfam used to build profile HMM after alignment pruning and exclusion.**

>D7LQF8

GEQLRYSFVSHLVDAFERNEVNFFVDKY‑‑EQRGKDL‑‑KNLFLRIQESRIALAIFSTRYTESSWCMDELVKMKKLAD‑KR‑KLHVIPIFYKVKVDDVRKQTGEFGDNFWTLAKVS‑‑‑‑SGDQ‑IKKWKEALECISNK‑MGL‑SLGDK‑SSEADFVKEVV

>D7MW48

GADVRKHFISFLDPALRKANINVFIDED‑‑EYLGSDL‑‑VNLLKRIEESEIALVIFSEEFTSSYWCLEELAKIKDCKD‑Q‑‑‑‑‑‑‑‑‑‑‑‑‑‑‑‑‑‑‑‑‑‑GKFGDHFRDQIRNL‑R‑HQPER‑TQKWEDALLSIPES‑IGM‑PLAAQ‑SDDKDFITSMV

>D7MKY7

GEDLRLGFVSHLVEALENDNIKVFIDNYA‑D‑KGEPL‑‑ETLLTKIQESRIALAIFSGKYTESTWCLRELAMIKDCVEK‑GNLV‑AIPIFYKLDPSTVRGVRGKFGDAFRDLEER‑‑‑‑‑D‑VLKKKEWKKALKWIPD‑LIGIT‑VHDK‑SPESEILNEIV

>D7KPK0

GADTRDNFGGRLYEALKK‑‑VRVFRDEGM‑K‑RGDEIGS‑SLQASMEDSAASVIVLSPNYANSHWCLDELAMLCDLKSSLD‑RR‑MLPVFYMVDPSHVRKQSGDF‑KDFQKLAKTF‑‑‑SE‑AE‑IKRWKDAMKLVGN‑LAGYV‑CHK‑‑SKEDDIIELVV

>D7MKA7

SSKDTRSFVSHLHAAFGRRGISVFLAEHC‑T‑LSATL‑‑‑‑KPLAIERSKIYVVVFSKNYASSPLCLETLMTFMDLQRKDG‑PV‑VIPVFYGVTRSIVEQQTERFKEDFSKHRG‑‑‑‑‑FK‑DR‑VERWRKGLTEAAK‑LHGHE‑‑SIE‑QNDSELVEDIV

>D7MBP1

GKQLRKGFVSHLEKALKRDGINAFIDEDE‑T‑RGNDL‑‑SILFSRIDESRIALAIFSSMYTESNWCLDELVKIKECVDL‑GKLV‑VIPIFYKVETDDVKNLKGVFGDKFWELVKT‑‑‑‑CN‑GEKLDKWKEALKVVTN‑KMGFT‑LGEM‑SNEGEYVEKIV

>D7MLF8

GDELRDNFIRYLVWGLRDERVNVFIDRA‑‑EANRRDI‑‑RNISTKIEESNIAVIIFSKRYTESEMCLNEHQKMHEHVK‑QS‑NLKVIPVFYDVSISDVKNLEGEFGNHFEEMKMKY‑A‑NDPLK‑ILNWENSLSSIVE‑‑‑GL‑TSEEH‑GTGLGLVMAIV

>D7MKX8

GAGVRKHFISFLDPALRKANINVFIDED‑‑EYLGSDL‑‑VNLLKRIEESEIALVIFSEDFTSSYWCLEELAKIKECKD‑QG‑RLKVIPIFYKVKPSVVKYLKGKFGDHFRDQIRNL‑R‑HQPER‑TQKWEDALLSIPES‑IGM‑PLAAQ‑‑‑DKDFITSMV

>D7MKX0

GEELRRGFISFLEPTLKNENINVFIDEL‑‑ELRGRDL‑‑QNLFVRIKESKIALVIFSKDYANSEWCLDELAMIKECMD‑QG‑NLDVIPIFYKVEPSVVKYLLGYFGENFMNLKNRY‑E‑NDPER‑TRKWEEALASVSQK‑FGL‑PFPEK‑SDDREFINSIV

>D7KQ34

GPDVRKSFLSHLRKQFNYNGITMFDDQGI‑E‑RSETIAP‑SLIQAIRESRILIVILSTNYASSSWCLNELVEIMECKKVMG‑QI‑VMTIFYGVDPTHVRKQIGDFGKAFSETCS‑‑RN‑TD‑VE‑MRKWSKALTDVSN‑ILGEH‑‑LLNWDNEANMIEKVA

>D7MUT0

RQDIQINFLDYLIAALRK‑‑‑NVIPSSK‑‑‑‑‑‑RYG‑‑IKITQEVPESRSTISIFTINYASSSWLLNELVETAKSNE‑DV‑‑‑MVIPIFHPDLPLDVREQTGEFGRRFEETCKNK‑‑‑‑TEDE‑KQQWRRALTDISSE‑YNI‑TY‑‑‑‑SSDEEFIKSMV

>D7MGR3

GADTRNSFTSYLVQFLQRKGIDTFFDGKL‑R‑RGKDI‑‑SVVFDRIEQSKMSIVVFSENYANSTWCLEELWKIIQCREK‑FGHG‑VLPVFYKVRKSDVENQKGTFGVPFLSPKESFG‑‑DG‑Q‑KVGAWKEALKIASN‑ILGYV‑LPEE‑RPESEFVEKIA

>D7KFH4

G‑‑‑‑‑KIALDVNYDLSRNGIKAFKSESWKS‑SFKPIDQ‑RTLEALTESKVAVVMTSDEEASSVGFLEELIVIVEFQ‑EKRSLT‑VIPIFLTKHPLDV‑EQVSQF‑‑‑‑‑‑‑‑‑‑‑‑‑‑‑P‑ER‑AIIWRTAIAKLDI‑IAAQY‑FSRN‑MHGTHRIKQIA

>D7MQS4

GEDVRRDFLSHIQMEFQRMGITPFIDNEI‑E‑RGQSIGP‑ELIRAIRESKIAIILLSRNYASSSWCLDELAEIMKCREELG‑QT‑VLAVFYKVDPSDVKKLTGDFGKVFKKTCA‑‑GK‑TK‑EH‑VGRWRQALANVAT‑IAGYH‑‑STNWDNEATMIRNIA

>D7MM05

GEDVRKGFLSHIQKEFQRMGITPFIDNEM‑K‑RGGSIGP‑ELLQAIRGSKIAIILLSRNYGSSKWCLDELVEIMKCREELG‑QT‑VMTVFYDVDPSDVRKQKGDFGKVFRKTCV‑‑GR‑PE‑EV‑KQKWKQALTSAAN‑ILGED‑‑SRNWENEADMIIKIA

>D7MK04

GADVRKTFLAHILKEFKGKGIVPFIDNDI‑E‑RSKSIGP‑ELIEAIKGSKIAIVLLSRNYASSSWCLNELVEIMNCREELG‑QT‑VMTIFYDVDPTDVKKQTGDFGKVFKKTCK‑‑GK‑TK‑ED‑IKRWQNVLEAVAT‑IAGEH‑‑SRNWDNEAAMTKKIA

>D7MXE9

GEELRNNFISHLHDALHRMGIKAFIDSDE‑P‑PGEDL‑‑DIFFKRIEQSKVALAVLSSRYTESHWCLEELAKIKECVDR‑SSLR‑VIPIFYNVDPTTVKELDGDFGLKLWDLWRK‑G‑‑‑R‑DNRILKWDAALQDVVD‑KIGMV‑L‑GI‑RNESEF‑‑‑‑‑

>D7KR50

GKDTRKNFISFLHKELESKGIRTFKDDTELE‑RGRPISP‑ELLQAIKGSRIAVVVVSVNYPDSFWCLEELKEILKL‑ENQGLLT‑VIPIFYEIDPSAVRRQIGVVAKQFKKHE‑‑‑KRESK‑ER‑VKSWKEALNKLAN‑LSGEC‑‑SKDWEDDSKLVDGIT

>D7LMJ8

GQDVRVDFLSHIQKEFRRKGIIPFIDNEI‑R‑RGESIGP‑ELIKAIRESKIAVVLFSRNYGSSKWCLDELVEIMKCREEFG‑QT‑VIPIFYKVDPSNVKKLTGDFGSVFRNTCA‑‑GK‑TK‑EV‑IGRWRQALAKLAT‑IAGYD‑‑SHNWYNEAAMIEKIV

>D7LI65

GKDMRRHFVSYLTHALKMNGVSFFLDEME‑V‑KGVDL‑‑GYLFKRIEESKLALVIISSRYTESAWCLNELVKIKELRDE‑GKLV‑AIPIFYKVEPSQVKKLKGVFGDNFRSLCRM‑‑‑‑NQ‑DHHITKWMEALMSMAS‑TMGFY‑LDEY‑SSESEFIKHIV

>D7MW45

EEDVSKGLINFLEPILKNENINVFIDEE‑‑TVRGKDL‑‑KNLFKRIQDTRISLAIFSESKCD‑‑‑‑FNELRKIKEPVD‑E‑‑‑‑‑AIPIFYKVDAI‑‑‑‑‑‑‑‑‑GD‑‑‑‑‑‑‑‑‑‑‑‑‑‑‑‑‑‑‑‑‑‑‑‑‑‑‑‑‑‑‑‑‑‑‑‑‑‑LADL‑QNKKDLINSAV

>D7KC67

GKDVRKTFLSHQLKEFGRKAINFFVDNEI‑K‑RGEFIGP‑ELKRAIKGSKIAVVLLSKNYASSSWCLDELVEIMK‑‑KESG‑QT‑VITIFYEVDPTDVKKQKGDFGKVFKKTCK‑‑GK‑GK‑EK‑VQTWKKALEGVAT‑IAGYH‑‑SSNWVDESTMIENIA

>D7KFH5

GKDTRRTFISFLYKELIGMSIRTFKDDVELK‑SGRRIAS‑DLLMAIENSKIAVVIVSKNYPASPWCLQELEMIMDV‑EKKGSLI‑VMPIFYNVEPAHVRRQIEKVAQQFRKHE‑‑‑NRENY‑ET‑VVSWRQALTNLAS‑ISGHC‑‑SRDCEDDSKLLDEIT

>D7MK93

GKDLRNGFVSHLVEALIRNKINVFMDKFE‑D‑RGKSL‑‑ESLLTRIEESRIALAIFSENYTESDWCVKEADKMNDCMKE‑GTLV‑VIPIFYKVKPSTVRDLEGRFGNKFWSLVKG‑‑‑‑‑D‑E‑RKKKWEEVWKSIPN‑LFGIT‑VDEK‑SDENRTVNEIV

>D7MK94

GEELRRPFVSHLHEALRNVGINAFIDSDE‑D‑PGEDL‑‑ENLFKRIEESEIALAILSSKYTESQWCLDELVKIMECSSK‑KKLW‑VIPIFYKLDTSIVKGLDGDFGVNLWKLWTK‑‑‑‑VR‑DDRIVKWNAALQDARN‑KTALI‑LKES‑SEEMAFLAKIV

>D7LXN7

CEETVRSFVSHLSSALHREGISVCVFA‑‑‑‑‑‑‑‑‑‑‑‑‑‑‑‑DQNQGARVTVVVFSENYAFPHPMLDNFAKILQLRSNSG‑HE‑VIPVFYGVDPSAVNPNH‑‑‑‑‑‑‑‑‑‑‑‑‑‑‑‑‑‑‑‑‑‑‑‑‑DWLPL‑‑HMEG‑HSMNS‑‑SNV‑SSDSQLVEDIV

>D7MCA2

GEDVRQSLISHLRKELDGKLVNTFNDTRI‑E‑RSRKINP‑ELLLAIEGSRISLVVFSKNYASSTWCLDELVKIQECHEQLD‑QM‑VIPIFYNVDPSHVRKQTGEFGKVFVETCK‑‑GR‑TE‑NE‑KRKWMRALTEVAN‑LAGED‑‑LRNGRSEAEMLENIA

>D7MRE0

GEDVRITFLSHFLKELDRKLIIAFKDNEI‑E‑RSQSLDP‑ELKQAIRSSRIAVVVFSEKYPSSSWCLDELLEIVRCKEELG‑QL‑VIPVFYGLDPSHVRKQTGQFGEAFAKTCQ‑‑RK‑TE‑DE‑TKLWRQSLTDVAN‑VLGYH‑‑SQNWPSEAKMIEAIA

>D7MIU1

GEDVRRGFLSHLHYHFASKGIMTFNDQKI‑E‑RGHTIGP‑ELVRAIRESRVSIVVLSKRYASSSWCLDELLEILKCKEDDG‑QI‑VLTIFYQVDPSDVRKQRGDFGSAFEITCQ‑‑GK‑PE‑EV‑KLRWSNALAHVAT‑IAGEH‑‑SLHWPNETEMIQKIA

>D7KYM9

GAELRYSFVSHLSDAFERHGINFFLDNH‑‑ELRGKDL‑‑ANLFVRIEESRIALAIFSTRYAESSWCMDELVKMKKCVD‑KG‑KLKVIPIFYKIRARDVRGQTGKFGDKFWELAKVS‑‑‑‑RGDK‑IKKWKDALECITGK‑MGL‑SLGKK‑CSEAGFIMEII

>D7MKB3

SEERLRSFVPHLSAAFGRKGISVSTSM‑‑‑‑‑‑‑‑‑‑‑‑‑‑‑‑DEF‑‑‑VASLLVFSEKYVSSKESLDEVVKTIQQRHDKG‑HV‑VATVFYGVSRSDVQELKGNFGKVLLENGA‑‑‑‑‑‑S‑DQ‑VTQWHNALAEIAS‑LPGYE‑‑ASN‑QSDYEFVEKIT

>D7MG62

GEDLSDGFIRHLASALRDEGFNVFIDDERR‑‑RGEHI‑‑‑‑‑‑RAIDNSKVALVIFSDRYTASELCLHEAVRIYDRRR‑EG‑KV‑LIPVFYRVS‑DDVN‑‑‑GRFGESFLETIQGIDH‑PA‑EH‑‑‑‑WMRNVNFICTD‑TGYI‑‑SEDYSSDTSLVVEIV

>D7MRE5

GEDVRNTFLSHFLKELDSKLIISFKDNEI‑E‑RSQSLDP‑ELKHGIRNSRIAVVVFSKNYASSSWCLNELLEIVKCKKEFG‑QL‑VIPIFYHLDPSHVRKQTGDFGKIFEKTCR‑‑NK‑TV‑DE‑KIRWKEALTDVAN‑ILGYH‑‑IVTWDNEASMIKEIA

>D7KVG7

GPDVRKTFLSHLRKQFNYNGITMFDDQGI‑E‑RSQTIAP‑ALTRAINESRIAIVVLSKNYASSSWCLDELVQILKCKEDRG‑QI‑VMTVFYGVDPHDVRKQTGDFGRAFNETCA‑‑RK‑TE‑EE‑RRKWSQALNYVGN‑IAGEH‑‑FRNWDNEAKMIEKIA

>D7LI64

GADLRYGFIDHLKKAFMANNIRYYIDEIE‑P‑RGENL‑‑GILFQRIRESRIALVFFSNRYPESEWCLDELVEIMKNMEN‑DTLR‑VIPIFFKVKPEDVRGQKKEFGVALYGEGRR‑‑‑‑‑R‑RPRMPQWEDALEAIPS‑NMGLV‑FQEQ‑SSEADFLAKLI

>D7MKY4

GAELRNGFVSHLVTALQSKDINVFIDKLE‑D‑RGKPI‑‑EILLDRIQKSRIALVIFSGKYTESVWCMREVAKIKDCMDE‑GTLE‑VIPIFYKVEPSTVKYLMGDFGDTFRSLAMN‑‑‑‑YD‑E‑GKEKWEDALKAVSG‑IMGTV‑VDEK‑SEESEIVKKTV

>D7MGR4

NHDVDESFIEAISKELHKREVTPLMYN‑‑‑‑‑‑‑‑‑‑‑‑‑‑LLKMLYRSSVGIMILSHSYACSRQALDHLVEIMEHGKARN‑LV‑IIPIYFKATLSDICGLEGRFEPIYLQYMD‑‑‑‑‑SL‑SR‑VQKWKAAMAEIAS‑IDGHE‑‑WEK‑EKQVLLAEEVV

>D7MKB6

GADVRYNFISHLEKALKDAGINVFVDEDE‑K‑RGKDL‑‑TVLFHRIEGSNMAIVVFSERYMESEWCLNELAKIKERVDE‑GKLV‑AIPIFFKVGADELKELLDVACETHGNVPGT‑‑‑‑‑‑‑‑‑‑‑QKWKVALECTTL‑KMGLT‑LGKK‑SDEANFVKMVV

>D7KR56

GLDTRRNFISFLYNELVRRNIRTFKDDNELK‑NGRRITP‑ELVRAIEGSKFAVVVVSVNYAASRWCLEELVKIMDF‑ENKGSLK‑VMPIFYGVDPCHVRRQIGVVAEQFKKHE‑‑‑AREDH‑EK‑VLSWRQALTNLAS‑ISGDC‑‑SWKWEDDSKMVDEIT

>D7LXP5

GQDVRRSFLSHFLEGLKGKGIKTFVDHGI‑M‑RSDSINS‑ELVRAIRESRIAVVILSKNYASSSWCLNELQLILECRVTLG‑QT‑VMTIFYDVDPSDVRKQTGDFGKVFEETCD‑‑GK‑TE‑EE‑KQRWRKALTEVAV‑IAGEH‑‑SVSWASEAAMISKIV

>D7MIU4

GPDVRRTFLSHLQHHFASKGITVFKDQEI‑K‑RGQTIGL‑ELKQAIRESRVSIVVLSKKYASSSWCLDELVEILKCREACG‑KI‑VMTIFYEIDPFHVRKQIGDFGRAFRETCF‑‑SK‑TK‑KV‑RLKWSKALTDVAN‑IAGEH‑‑SLRWEDEAKMIEKIA

>D7MKB2

GDQLHYNFVSYLVDALRRSEINVFIDNE‑‑EQRGEDL‑‑NTLFKRIEESGIAIVVLLTHITN‑‑‑‑FGNIVTLYHKV‑‑‑‑‑‑‑‑‑LPIFYKVTPTNVKRLKGEFGDHFRDKEYMY‑K‑SDEPM‑IKQWKEAIVSVSHK‑FAL‑ALDEK‑SSEIDFVETIV

>D7MRZ0

GGDVRVTFRSHFLKELDRKLITAFRDNEI‑E‑RSHSLWP‑DLEQAIKESRIAVVLFSKNYASSSWCLNELLEIVNCND‑‑‑‑KI‑VIPVFYGVDPSQVRHQIGDFGSIFEKTCR‑‑RH‑SE‑EV‑KNQWKKALTDVAN‑MLGFD‑‑SATWDDEAKMIEEIA

>D7LX32

GEDVRKTFVSHLFCEFDRMGINAFRDDLDLQ‑RGKSISP‑ELIDAIKGSRFAIVVVSRNYAASSWCLDELLKIMEC‑KDTISQT‑ILPIFYEVDPSDVRRQRGSFGEDVESHS‑‑‑‑‑‑DK‑EK‑VRKWKEALKKLAA‑ISGED‑‑SRNWRDESKLIKKIV

>D7KR43

GKDTRRTVVSFLYKDLIRQGILTYKDDQGIG‑AGSEIKE‑RLIEAIKTSQVAVVFISENYATSQWCLEELRLIMEL‑HSVNRIH‑VVPIFYRVDPSDVRHQKGRFAAAFQKHE‑‑‑DRE‑P‑NR‑ASQWRRALNQISH‑ISGIH‑‑STEWDDDSAMIDEVV

>D7LMK1

GADVRRTLLSHIMESFRRKGIDTFIDNNI‑E‑RSKPIGP‑ELKEAIKGSKIAIVLLSKNYASSSWCLDELAEIMKCREVLG‑QI‑VMTIFYEVDPTDIKKQTGDFGKAFRKTCK‑‑GK‑TK‑EH‑IERWRKALKDVAI‑IAGEH‑‑SRNWSNEAEMIEKIS

>D7MUS0

GADLRLRFVSHLVTALKLNNINVFIDDYE‑D‑RGQPL‑‑DVLLKRIEESKIVLAIFSGNYTESIWCVRELEKIKDCTDE‑GTLV‑AIPIFYKLEPSTVRDLKGKFGDRFRSMAKG‑‑‑‑‑D‑E‑RKKKWKEAFNLIPN‑IMGIT‑IDKK‑SVESEKVNEIV

>D7KRU8

GKDLRKGFLSFLSPALKKEKINVFIDEQ‑‑EERGKYL‑‑ISLFNTIGESKIALVIFSEGYCESHWCMDELVTIKEYMD‑KN‑RLKIIPIFYRLELDVVKDLTGKFGDNFWNMVDNY‑Q‑PEPEK‑LHKWTEALFSVCQL‑FAL‑ILPKH‑SDDRDFVKVIV

>D7MKY8

CVSEVRSFVSHLSEALRRKGISSVIID‑V‑D‑SDLL‑‑‑‑‑‑‑AKIEISRVSVMVLSRICEPTRVC‑QNFVNVIECQRNKN‑QV‑VVPVLYGESPLLG‑‑‑‑‑‑‑‑‑‑‑‑‑‑‑‑‑‑‑‑‑‑‑‑‑‑‑‑‑EWLSVL‑DLRD‑LPVHQ‑‑SRK‑CSDSQFVKEIV

>D7MKX9

GKDVRNGFLSFLEPAMREANINVFIDKH‑‑EVVGTDL‑‑VNLFVRIQESRVVVVIFSKDYTSSEWCLDELAQIKDCID‑QG‑GLNVIPIFYKLAPSSVEELKGGFGDSFRVLKCKY‑K‑DEPER‑TQKWEEALKSIPKI‑KGL‑TLSEK‑SDEREFMNETI

>D7MKA4

N‑DSSVSFISYLIAAFNRQGIISAFVDGK‑S‑SHEAV‑‑E‑‑‑EEFSKLRVVVVVFSKNYALHVSFLEKQI‑‑LEYSRNNN‑DFVVVPVFYGVSISSVNQHMERFGEEFDAIQR‑‑‑‑‑S‑‑‑‑‑‑IKWRP‑‑‑‑‑‑‑‑‑‑GHE‑‑YDK‑YSESEFLEEIA

>D7MUS9

GADIRFGFVSHLVEAFKKHKINFVYDDYE‑D‑RGQPI‑‑EILLTRIEQSRIALAIFSGKYTESFWCLEELTKIRNCEKE‑GKLV‑AIPIFYKVEPSTVRYLMGEFGDSFRSLPKD‑‑‑‑‑D‑E‑KKKEWEEALNVIPG‑IMGII‑VNER‑SSESEIIKKIV

>D7MKY3

CVKQVRSFVSHLSEALRRKGIIDVFI‑‑‑‑D‑TDFL‑‑‑‑‑‑‑SKVERARVSVVVLS‑‑‑GNSTVCLDKLVNVLGCQRNID‑QV‑VVPVLYGEIPLQV‑‑‑‑‑‑‑‑‑‑‑‑‑‑‑‑‑‑‑‑‑‑‑‑‑‑‑‑‑EWDKAL‑NSRG‑LSVHQ‑‑SRN‑CTDSELVEEIT

>D7KR54

GLDTRRNFISFLYKELVRRNIRTFKDDKELE‑NGRRISP‑ELKRAIEESKFAVVVVSVNYAASPWCLDELVKIMDF‑ENKGSIT‑VMPIFYGVDPCHLRRQSGDVAEQFKKHE‑‑‑AREDL‑DK‑VASWRRALTSLAS‑ISGDC‑‑SLKCEDESKLVDEIA

>D7MRE1

GEDVRVSFRSHFLKELDRKLITAFKDNEI‑K‑KSHSLWP‑ELVQAIKESRIAVVVFSKNYASSSWCLNELLEIVNCND‑‑‑‑KI‑VIPVFYGVDPSHVRNQTGDFGRIFEETCE‑‑KN‑TE‑QV‑KNRWKKALSDVAN‑MFGFH‑‑SATWDDEAKMIEEIA

>D7KR46

GKEMRKTFVSHLLSSFTTKNITSFIVDPF‑E‑‑‑‑‑‑DP‑DL‑KAMEQSLVAIPVISKNYVSNLW‑MDDLRKIIECEK‑IGTLT‑AIPIFFQVSPLDILHAT‑‑‑‑EKYADT‑‑‑‑DE‑T‑LEM‑VRKW‑‑‑‑‑‑VSR‑‑PSFH‑‑SNDWDDDSELVDKIT

>D7MVQ3

GEYVRRDFLSHIQMEFQRMGITPFIDNEI‑E‑RGQSIGP‑ELIRAIRESKIAIILLSRNYASSSWCLDELAEIMKCRDELG‑QT‑VLAVFYKVDPSDVKKLTGDFGKVFKKTCA‑‑GK‑TK‑EH‑VGRWRQALANVAT‑IAGYH‑‑STNWDNEAAMIKKIA

>D7MIS2

GPDVRRGFLSHLHNHFASKGITTFNDEKI‑D‑RGQTIGP‑ELVQAIRESRVSVVLLSKKYASSSWCLDELLEILKCNEAQG‑QI‑VMTIFYDVDPSDVKKQRGEFGKAFEKTCE‑‑GK‑TE‑EV‑KQRWIEALAHVAT‑IAGEH‑‑SLNWANEAEMIQKIA

>D7LXP7

GEDVRKNFLSHLQKELQLRGINAFKDHGI‑K‑RSRSIWP‑ELKQAIWESRISIVVLSSNYAGSSWCLDELLEIMECREAVG‑QT‑LLTVFYEVDPSDVRKQTGAFGKVFEKTCL‑‑GR‑TV‑EE‑TQRWKQALTDVAN‑VSGYC‑‑SEKWDNEASMIEKIV

>D7MJ12

GPDVRNGFLSHLHNHFESKGITTFNDQEI‑E‑RGHTIGP‑ELVQAIRESRVSIVVLSEKYASSGWCLDELVEILKCKEASG‑QA‑VLTIFYKVDPSDVRKQRGDFGNTFKKTCE‑‑GK‑TE‑EV‑KQRWIKALTDVAT‑IAGEH‑‑SLNWANEAEMIQKIA

>D7MRE2

GEDVRITFLSHFLKELDRKLIIAFKDNEI‑E‑RSQSLDP‑ELKQAIRTSRIAVVVFSEKYPSSSWCLDELLEIVRCKEELG‑QL‑VIPVFYGLDPSHARKQTGKFGEAFVKTCQ‑‑RK‑TE‑DE‑TKLWRQSLTDVAN‑VLGYH‑‑SQNWPNEAQMIEAIA

>D7LLZ1

GEDVRKDFLSHIQKGFERKGIRQFNDYEI‑E‑RGESISF‑QLIRAIRGSKIAVILFSRNYASSKWCLDELMEIMKCRRELG‑QI‑VIAIFYKVDPSDVRNQSGDFGKVFRKTCA‑‑GK‑TK‑EE‑IRRWRTALAEVAT‑IAGYH‑‑SSNWDNEAAMIENIA

>D7MRD3

GDDLRHNFLAHFRKELDRKLIRTFNDMEI‑E‑KGESLDP‑VLTQAIRGSKIAVVLFSKNYASSGWCLNELLEIVKCKKEIG‑QL‑VIPIFHGVDPSHVRHQIGDFGSIFEKTCR‑‑RH‑SE‑EV‑KNQWKKALTEVAN‑MVGTH‑‑LQNWDNEAKQIEYIV

>D7MJ13

GPDVRSGFLSHLHNHFESKGITTFNDQEI‑E‑RGHTIGP‑ELVQAIRESRVSIVVLSEKYASSGWCLDELVEILKCKEASG‑QA‑VMTIFYKVDPSDVRKQRGDFGYTFKKTCE‑‑GK‑TE‑EV‑KQRWIKALNDAAT‑IAGEN‑‑SLNWANEAEMIQKIA

>D7KSY0

GEDVRKTFLSHIRKQFICNGITMFDDQGI‑K‑RGKTITP‑ELIQGIRESRISIIVLSKNYASSSWCLDELLEILKCREDIG‑QI‑VMTVFYGVDTSDVRKQTGEFGIAFNKTCA‑‑GK‑TE‑EE‑SRRWSQALTDAAN‑IAGVD‑‑FKNCKNEAEMIEEIA

>D7KXD9

GPDVRVTFLSHLQKQFQHNGIITFNDEGI‑E‑RSQTISS‑ELTRAIRESRISIVVLSENYASSSWCLNELLEISKCQESAG‑QI‑VMTVFYKVDPSDVRKQMGEFGKAFKKTCQ‑‑GK‑TE‑AK‑IHRWTQSLTHVAN‑IAGEH‑‑SLNWDNEANMIEKIA

>D7MVQ6

GEDVRRDFLSHIHMEFQRMGITPFIDNEI‑E‑RGQSIGP‑ELIRAIRESKIAIILLSRNYASSSWCLDELAEIMKCREELG‑QT‑VLAVFYKVDPSDVKKLTGDFGKVFKKTCA‑‑GK‑TK‑EH‑VGRWRQALANVAT‑IAGYH‑‑STNWDNEATMIRNIA

>D7LM60

GEDVRVTFLSHFLKELDRKLIIAFKDNEI‑K‑KSESLDP‑VLKQAIKDSRIAVVVFSINYASSTWCLNELVEIVKCKEEFS‑QM‑VIPVFYRLDPSHVRKQTGDFGKIFEKTCH‑‑NK‑TE‑EV‑KIQWKEALTSVAN‑ILGYH‑‑STTWFNEAKMIEEIA

>D7MVI5

‑‑‑‑‑‑GFIDHLYINLKRSGIHTFKDEAL‑K‑RGENLSP‑TLLKAIKSSKVHLVVLTENYSSSMWCLDELMHIMECRRNPG‑HV‑VVPIFYDVEPRDVRRQRGSFGAYFSKHEARH‑K‑‑‑‑‑‑‑VQKWKDALTEVAN‑RLGHV‑‑RANYRSEVELIYEIT

>D7MEA2

GVDVRRDFLSHIQKEFQRKGITPFIDNEI‑K‑RGESIGP‑ELIHAIRGSKIAIILLSRNYASSKWCLDELVEIMKCREELG‑QS‑VMVIFYKVDPYDVKTLAGEFGKVFSKTCA‑‑GN‑TK‑ED‑IKRWRQALGKVAT‑IAGYH‑‑SSNWDNEAAMIERLA

>D7MCA5

GEDVRKTFLSHLLKALDGKSINTFMDHGI‑E‑RSRTIAP‑ELISAIREARISIVIFSKNYASSTWCLNELVEIHKCCKDLD‑QM‑VIPVFYYIDPSEVRKQIGEFGDVFKKTCE‑‑DK‑PE‑DQ‑KQRWVQALTDISN‑IAGED‑‑LRNGPDEAHMVEKIV

>D7KFH3

GEDTRKTIVSHLYAALDSRGIVTFKDDQRLE‑IGDHISD‑ELRRALGSSSFAVVVLSENYATSRWCLLELQLIMEL‑MKEGRLE‑VFPIFYGVDPSVVRHQLGSF‑‑ALEKYQG‑‑‑PE‑A‑DK‑VLRWREALNLIAN‑LSGVV‑‑SSHCVDEAIMVGEIA

>D7MW47

GKDVRNGFLSFLEPAMREANINVFIDKH‑‑EVVGTDL‑‑VNLFVRIQESRVVVVIFSKDYTSSEWCLDELAQIKDCID‑QG‑GLNVIPIFYKLAPSSVEELKGGFGDSFRVLKCKY‑K‑DEPER‑TQKWEEALKYIPKI‑KGL‑TLSEK‑SDEREFIYETI

>D7MKZ4

GDELRKSFLGFLVKAMRDANINVFTDEI‑‑EVKGKDL‑‑QNLFSRIEESRVAVAILSKRYTESSWCLDELVKMKERND‑QD‑KLVVIPIFYRLDANNCKRLEGPFGDNFRKLEREY‑R‑SEPER‑IKKWKEALIYIPQK‑IGL‑TSAGH‑RR‑TVVSSRIH

>D7KXG7

GEDVRRKILSYIQQEFQRKGIIPFIDNEI‑K‑RGESIGP‑ELIKAIRESKIAIVLLSRNYASSKWCLDELVEIMECKKKFG‑LT‑VFVVFYEVDPSHVKKLTGEFGAVFQKTCK‑‑GR‑TK‑EE‑IWRWRQAFEEVAT‑IAGYD‑‑SRDWENEAAMV‑‑‑‑

>D7L878

GPDVRKSFLSHFRKQFISNGITMFDDQKI‑V‑RSQTIAP‑SLTQGIRESRISIVILSKNYASSTWCLDELLEILKCREDIG‑QI‑VMTVFYGVDPSDVRKQTGEFGTVFNKTCA‑‑RR‑TK‑EE‑RRNWSQALNDVGN‑IAGEH‑‑FLNWDNEAEMIEKIA

>D7MKA8

GTELRNNFISHLEKALLNKKVNVFIDIRE‑R‑IGKDK‑‑DIFFQRIRESRITIAVISSKYTESKWCLNELAEIQKCVLA‑ETME‑VFPVFYKVDVGTVEKQTGEFGENFKKLLEQ‑‑‑‑HH‑SER‑EKWERALKFVTS‑KLGVR‑VDEK‑SFECDIVDHVV

>D7KC69

G‑NLRYGFVSHLTDALKRHNINFFIDTH‑‑EQKGRDL‑‑KHLFKRIEEATVALVILSTRYAESKWCLDELTKIMDQAE‑KM‑EMIVIPIFYKVKPKDVELQEGVFGDRFWSHADQS‑‑‑‑SREE‑MEKWQVALKAVCNK‑VGI‑TLYRK‑‑‑EAKFIKKVV

>D7KB25

GPDVRKTFLSHLRNQFNQNGITMFDDNGI‑P‑RSENIPS‑ALIQGIRESRISIIVLSKMYASSRWCLDELLEILKCKEDVG‑KI‑VMTVFYGVDPSDVRNQTGDFGIAFNKTCA‑‑RK‑TK‑EH‑GRKWSEALDYVGN‑IAGEH‑‑‑‑NWGNEAEMIAKIA

>D7KC63

GKDVRKAFLSHILKEFGRKAINFFVDNEI‑K‑RGEFIGP‑ELKRAIKGSKIALVLLSKNYASSSWCLDELAEIMK‑‑QESG‑QT‑VITIFYEVDPTDVKKQKGDFGKVFKKTCK‑‑GK‑DK‑EK‑IKTWRKALEDVAT‑IAGYH‑‑SSNWVDEAAMIENIA

>D7KT09

GEDVRRDFLSHIHKEFQRKGITPFIDSEI‑K‑RGESIGL‑EIVQAIRGSKIAIVLLSRNYASSSWCLDELVEIMKCKEELS‑QI‑VIPIFYKVDPSDVKKLTGSFGSVFEDRCA‑‑GK‑TN‑EL‑IRRWRQALAKVAT‑ITGYD‑‑SRCWDNEAAMIEKIA

>D7MK98

GRDLRYGFVSHLEKILKDHKIEVFVDSGE‑D‑RGEHL‑‑ENLLTRIEESRIALAIFSENYTESEWCLRELAKIKDCVDQ‑KRLV‑AIPIFYKVEPSTVKYLMGEFGDAFRKLAKN‑‑‑‑‑D‑K‑RKKEWKAALRAIPE‑FMGIP‑VHEK‑SPESEILKTIV

>D7MW46

GADVRSHFISHLDPALREANINVFIDDD‑‑EFLGTDL‑‑VNLLKRIEESEIALVIFSEDFTSSYRCLDELAKIKECKD‑QG‑RLIVIPIFYKVKPSVVKYLKGNFGDNFRELERNN‑L‑HMQQR‑TQKWKKALVSIPES‑KGM‑PRAEQ‑SEDKDFITSMV

>D7KTD8

GPDVRKTFLSHLRKQFICNGITMFDDQGI‑E‑RGQTISP‑ELTRGIRESRISIVVLSKNYASSSWCLDELLEILKCKEDIG‑QI‑VMTIFYGVYPSHVRKQTGEFGIRLSETCD‑‑GK‑TE‑EE‑RRRWSQALNDVGN‑IAGEH‑‑FLNWDKESKMVEKIA

>D7MVQ1

GEDVRRDFLSHIQMEFQRMGITPFIDNEI‑K‑RGQSIGP‑ELIRAIRESKIAIILLSRNYASSSWCLDELAEIMKCREELG‑QT‑VLAVFYKVDPSDVKKLTGDFGKVFKKTCA‑‑GK‑TK‑EH‑VGRWRQALANVAT‑IAGYH‑‑STNWDNEAAMIKKIA

>D7LTW2

GEELRNSFVSHLRSALVRHGVNIFIDTNE‑Q‑KGKPL‑‑HVFFERIEESRIALAIFSLRYTESKWCLNELVKMKECMDK‑GKLL‑IIPIFYKVKAYEVRYQKGRFGYVFKNL‑RN‑‑‑‑AD‑VHQKNQWSEALSSVAD‑RIGFP‑FDGK‑SDENNFINGIV

>D7L8B6

GEDVRRTFLSHLLLALDRKLITCFKDSEI‑Q‑RSQSIGL‑ELVHAIRGSRIAIVVFSKIYASSSWCLNELLEIVKCKEEKG‑QM‑VIPIFYALDPSHVRKQTGDFGKAFEMICE‑‑SK‑TD‑EL‑QIQWRRALTDVAN‑IHGYH‑‑SENWYNEAHLIEEIA

>D7MVQ4

GEDVRRDFLSHIQMEFQRMGITPFIDNEI‑E‑RGQSIGP‑ELIRAIRESKIAIILLSRNYASSSWCLDELAEIMKCREELG‑QT‑VLAVFYKVDPSDVNKLTGDFGKVFKKTCA‑‑GK‑TK‑EH‑VGRWRQALANVAT‑IAGYH‑‑STNWDNEATMIRNIA

>D7M8F3

GEDVRVTFLTHFFKELDRKMIIAFKDNEI‑E‑RGHSIGP‑KLIKAIKDSRIAVVVFSKNYSSSSWCLNELLEIVKCQ‑‑‑‑‑EI‑VIPIFYDLDPSDVRKQEGEFGESFKKTCK‑‑NR‑TK‑DE‑IQRWREALTNVAN‑IAGYH‑‑TGKPNDEAKLIEEIA

>D7MG64

GGDLSDYFVRELATALRDQGFNVFIGDERGR‑RGKYI‑‑‑‑‑‑RTIESSDVALVIFSDMYAASEWCLHEAVRIYDRRR‑EG‑KV‑LVPIYYRVS‑EDVN‑‑‑GRFGECFVKTTRGIGH‑SA‑DI‑‑‑‑WKANVNLICTE‑PGFT‑‑SKDFCHDVNFMVAMV

>D7KR45

GEDTRKGIVSHLHRAFLARGIKIFKDDQTLE‑IGDSISE‑EIKEAIHNSKFAILVISMNYASSTWCLDELQMIMEL‑HKEKQLT‑AVPIFYNVDPSDVRHQRGTF‑‑ALERYEC‑‑SRVMA‑AK‑IQKWREALREVAG‑TSGKD‑‑LSTCKDEATMVADIV

>D7KSY5

GPDVRKTLLSHMRKQFNRNGITMFDDEKI‑E‑RSATIAP‑SLIGGIRDSRISIVILSKKYASSSWCLDELVEILECKKVMG‑QI‑VMTIFYGADPSDVRKQLGEFGIAFDETCA‑‑HK‑TD‑EE‑RKKWSEALNEVGN‑IAGED‑‑FNRWDNEANMIKKIA

>D7MLF9

GEKLRDGFLGFLVDALLKENVNVFIDDH‑‑ELRGRDL‑‑DHLFSRIEESRVALTIFSKNFTESRWCLDELAKIRECVD‑QG‑SLTVIPIFFKMKTDDVKKLKGKFGDNFRDLKSTH‑R‑GEPEN‑FRRWKEALIFVSEK‑AGL‑SSSRY‑SR‑NDLVSTIV

>D7MRH7

GEDVRKTFLSHFLKELDRRLILAFKDNEI‑E‑RSRSLDP‑ELNHAFKGSKIAVVVFSRNYASSSWCLNELLEIVRCKEEFG‑QM‑VVPIFYHLDPSHVRNQTGDFGKMFEQTCQ‑‑HK‑TE‑DQ‑KIRWRRALTDVAN‑ILGYH‑‑SVAWDNEASMVEEFA

>D7LXN6

GVELRYNFVSHLKKGLKRNGINAFIDTDE‑D‑MGQEL‑‑NILLKRIEGSKIALAIFSPRYTESDWCLKELAKMKECREQ‑GKLV‑VIPIFYKVEPSTVKRQKGEFGDNFRDLVEF‑‑‑‑ID‑EETKNNWTEALKSIPL‑LTGFV‑LNEN‑SDEDDLIFKVV

>D7MJC1

GEDTRHSIVSHLYEALTSRGIATFKDDKRLE‑LGDHISE‑ELQRAIEGSDFAVVVLSENYPTSRWCLMELQSIMKL‑QVEGRLG‑VFPVFYSVEPSAVRYHLGSF‑‑DLEGYQR‑‑DPK‑A‑DV‑VPKWRQALKLIAD‑LSGVA‑‑SGQCIDEATMVRKIV

>D7MK10

GADVRKNFLAHILKEFKGKGIVPFIDNDI‑E‑RSKSIGP‑ELIEAIKGSKIAIVLLSRNYASSSWCLNELVEIMNCREELG‑QT‑VMTIFYDVDPTDVKKQTGDFGKVFKKTCK‑‑GK‑TK‑ED‑IKRWQNVLEAVAT‑IAGEH‑‑SCNWDNEAAMTEKIA

>D7LTW1

REDTVRSFVSHLSAAFHRRGVSSFTGE‑H‑G‑SDET‑‑‑‑‑‑‑‑‑LEKSRASVVVFSEKYPSSKSCMEELLKVSEHRRKNC‑LA‑VVPVFYPVTKSFVKKQICNLA‑‑‑‑‑‑‑‑‑‑‑‑‑‑‑‑DV‑RSDWRTALLETVD‑LPGHE‑‑LYD‑QSDSDFVVEIV

>D7LNZ5

GEDVRRNFLSHIQKEFQRKGITTFVDNEI‑K‑RGESIGP‑KLIHAIRGSKIALVLLSKNYASSSWCLDELVEIMKCKEELG‑QT‑VLPIFYKIDPSDVKKLTGKFGSAFKNICA‑‑CK‑TN‑EI‑IRKWRQALAKVAT‑TTGYS‑‑SRNWDNEADMIEKIS

>D7LMK5

GADVRRTLLSHIMESFRRKGIDTFIDNNI‑E‑RSKPIGP‑ELKEAIKGSKIAIVLLSKNYASSSWCLDELAEIMKCREVLG‑QI‑VMTIFYEVDPTDIKKQTGDFGKAFRKTCK‑‑GK‑TK‑EH‑IERWRKALKDVAI‑IAGEH‑‑SRNWSNEAEMIEKIS

>D7KZ23

GEDVRKNFVCHFIKELDRKLITAFKDNQI‑E‑RSRSLDP‑ELKQAIRDSRIAVVVFSKNYASSSWCLDELLEIVRCKKEYG‑QL‑VIPIFYRLDPSHVRKQTGEFGKIFEKTCQ‑‑HK‑TK‑QV‑QNRWSRALSHVSN‑ILGYH‑‑SVTWENEAKMIEEIT

>D7MKY5

GKELRKGFISFLVPALKDNNINVFIDDQ‑‑EERGKYL‑‑TSLFDRMGESKIALVIFSEDYTESKWCLDELVQIKECMD‑QN‑KLRVIPIFYKLDPAVVKRLQGKFGDQFRDLEYRY‑K‑HKPER‑PQKWKEAVISVCQT‑FAL‑FLPEH‑SDDKDFIMLIV

>D7MRE4

GEDVRLTFLSHLLKELDRKMIIAFKDNEI‑P‑RGQSLDP‑ELKQAIRDSRIAVVVFSKNYASSTWCLNELLEIVQYKEEFGRQM‑VIPVFYDLDPSHVRKQTGDFGKIFQETCK‑‑NK‑TE‑DV‑INRWKKALTDVAN‑ILGYH‑‑SVTQVNEAKMIEEIA

>D7LXU4

GEDVRRTFLSHLLKKFQLKGIRTFMDNDI‑E‑RGQMIGP‑ELIQAIRESRFAVVVLSKTYASSKWCLDELVEIKE‑‑‑‑AS‑KK‑VIPIFYNVEPSDVKNIGGEFGNEFEKACK‑‑EK‑P‑‑EK‑LDRWREALVYVAD‑IAGEC‑‑SQNWVSEADMIENIA

>D7MLG0

GAELRNSFISHLEGALALAGIKYYIDTKE‑V‑PSEDL‑‑SVLFERIEQSEIALSIFSSKYAESNWCLDELVKIMEQVKK‑EKLR‑IIPVFFNVKPEEVREQKGEFGLKLYGEGKR‑‑‑‑‑K‑RPNIPNWENALQSVPS‑KIGLN‑LSNY‑RNERELVEKIV

>D7KR44

AEDTTNIFVSDLHRSLSEKGITYQKDEKQEE‑KDSSVVS‑DLKKCIIESKLAVVVVSKSYPTSVLCLNQLQTIINF‑HDEGQLS‑VLPIFYGVDLSNIRNQTGEYTEAFRNLA‑‑‑‑‑‑SP‑EK‑VQAWRSALAKLTS‑VSSLD‑‑SRFWSKEETMVDLVT

>D7MK07

GADVRKTFLAHILKEFKGKGIVPFIDNDI‑E‑RSKSIGP‑ELVEAIRGSKIAIVLLSRNYASSSWCLNELVEIMKCREELG‑QT‑VMTIFYDVDPTDVKKQTGDFGKVFKKTCK‑‑GK‑TK‑ED‑IKRWQNVLEAVAT‑IAGEH‑‑SCNWDNEAAMTEKIA

>D7MKB5

SEETLRSFVPHLSAAFGRKGISVLTDK‑‑‑‑‑‑‑‑‑‑‑‑‑‑‑‑DQSYKSIASVLIFSENYVSSKESLDEFIKTIQRRHEKG‑HI‑VTAIFYGVSRSNVQELMGNFSKAFLEHRD‑‑‑‑‑‑S‑DQ‑VNQWRNALAEITS‑LPGYE‑‑TSN‑QSDYKSVEKIA

>D7MRH5

GEDIRKSFLSHFYKELDRKPILVFKDNEI‑K‑RGISLGP‑KLKRAIRDSRIAVVIFSRKYASSSWCLNELLEIVRCKKEFS‑QV‑VIPIFFHLDPTHVRKQTGVFGMNFEKTCH‑‑NK‑TE‑KM‑KIRLRRALTEVAN‑ITGYH‑‑SVTCKNEAKMIEAII

>D7MKA3

GVELRKTFISHLHTRLRRDGINAFIDSDE‑A‑PGREL‑‑KNLFKRIEDSKIALAVLSSRYTESHWCLQELVKMMECSPKNNKLL‑VIPIFYKLKISTVAELDGDFGRNLWDLWRL‑GR‑DR‑DNRIVKWNEALQDVLS‑RNALV‑LPET‑GKEDDFLSTIV

>D7LIG9

GPDVRRGFLSHLHNLFASKGITTFNDEKI‑D‑RGQPIGP‑ELVQAIRESRVSIVLLSKKYASSSWCLDELLEILKCKEDDG‑QI‑LMTIFYDVNPSHVKKQRGEFGKAFEKTCQ‑‑GK‑TE‑EL‑KQRWSKALAHVAT‑IAGEH‑‑SLNWPYEAEMIQKIA

>D7MKY0

GADVRSHFISYLDPALREANINVFIDDD‑‑ELLGSDL‑‑VHLLKRIEESEIALVIFSEDFTSSYWCLEELAKIKECKD‑QG‑RLKVIPIFYKVKPSVVKYLKGKFGDHFRDQIRNL‑R‑HQPER‑TQKWEDALLSIPES‑IGM‑PLAAQ‑SEDKDFITSMV

>D7KSY1

GPDVRKTFLSHLRKEFICNGITMFDDQGI‑E‑RGQTISP‑ELTQGIRESRISIVLLSKNYASSSWCLDELLEILKCKEDMG‑QI‑VMTVFYGVNPSDVRKQTGEFGMAFNETCA‑‑RK‑TE‑EE‑RRKWSQALNDVGN‑IAGEH‑‑FLNWDNESKMIEKIA

>D7M0D5

QINDD‑‑FISHLRAALCRRGISVF‑‑‑‑‑‑‑‑‑‑‑‑‑‑‑‑‑‑‑DAVPKCRVFIILLTSTYVPS‑‑‑‑‑NLLNILEHQQTEY‑QA‑VYPIFYRLSPYDLISNSKNYERYFLQ‑‑‑‑‑‑‑‑‑‑‑NE‑PERWQAALKEISQ‑MPGYT‑‑LTD‑RSESELIDEIV

>D7KPJ8

GFDTRANFCERLYVALEKQNVRVFRDEGM‑E‑KGDKIDP‑SLFEAIEDSAASVIVLSKNYANSAWCLNELALICELRSSLK‑RP‑MIPIFYGVNPSDVRKQSGHF‑KDFEENAKTF‑‑‑DE‑ET‑IQRWKRAMNLVGN‑IPGFV‑CTEN‑DKVDDMIELVV

>D7M0D4

GFDTRNNFTGHLQKALRLRGIDSFIDDRL‑H‑RGDNL‑‑TALFDRIEKSKIAIIIFSTNYANSAWCLRELVKILECRNR‑NQQL‑VVPIFYKVEKSDV‑‑‑‑‑‑‑‑‑‑‑KIQELTFG‑‑VS‑PEEISSWKAALVSASN‑ILGYV‑VKES‑TSEANLVDEIA

>D7KV07

GEQLRQNFVSHLVEALRRNAINVFIDNQ‑‑ELRGEDI‑‑SILLKRIEDSRIAIVVFSSRYTESRWCLREAVKIKECVE‑QD‑MLKVLPIFYKVTTTGVKQLKGEFGDHFRDREWEY‑R‑FDKPR‑IERWKEALAFLSGK‑LGL‑TFDEK‑SSESDFIESIV

>D7KXT5

SHDVDEQFMEAILKELHERGITPLTYN‑‑‑‑‑‑‑‑‑‑‑‑‑‑LSEMLNRSSVGIMVFSNSYVCSKQSLDHLVAIMEHWKAKD‑IV‑IIPIYFKVTLQHICGLKGMSEAAFLHLQS‑‑‑‑‑SE‑DR‑VQKWKMALAEIES‑IDGHE‑‑WTK‑GTEVMLAEEVV

>D7MKX2

GEDLRLGFVSHLVEALENDNIKVFIDNYA‑D‑KGEPL‑‑ETLLTKIQESRIALAIFSGKYTESTWCLRELAMIKDCVEK‑GNLV‑AIPIFYKLDPSTVRGVRGQFGDAFRDLEER‑‑‑‑‑D‑VLKKKEWKKALKWVPD‑LIGIT‑VHNK‑SPESEILNEIV

>D7KR53

GLDTRRNFISFLYQELVRRKIRTFKDDKELK‑NGQRISP‑ELKRAIEESRFAVVVVSQNYAASRWCLKELVKIMDF‑ENKDSIT‑VIPIFYGVEPGHVRWQTGVVAEHFKKHE‑‑‑SREKH‑EK‑VLQWKQALAAFAQ‑LSGDC‑‑SG‑‑DDDSKLV‑‑‑A

>Q9CAE0

GPDVRKTLLSNLREHFQGKGITMFDDEKI‑K‑RGGDLSP‑SLKRAIKTSKISIVILSQKYASSSWCLDELLEIMKRKKAMK‑QI‑VMTVFYGVEPSDVRKQTGDFGIAFNKTCV‑‑NK‑TD‑KE‑RKEWSKALTDVSN‑IAGED‑‑FKKWDNEANMIKKIA

>F4I819

GDELRNSFVGFLVKAMRLEKINVFTDEV‑‑ELRGTNL‑‑NYLFRRIEESRVAVAIFSERYTESCWCLDELVKMKEQME‑QG‑KLVVVPVFYRLNATACKRFMGAFGDNLRNLEWEY‑R‑SEPER‑IQKWKEALSSVFSN‑IGL‑TSDIR‑SN‑SKFVDSIV

>Q8LPH7

GEDVRVTFRSHFLKELDRKLITAFRDNEI‑E‑RSHSLWP‑DLEQAIKESRIAVVVFSKNYASSSWCLNELLEIVNCND‑‑‑‑KI‑VIPVFYHVDPSQVRHQIGDFGKIFENTCK‑‑RQ‑TE‑EV‑KNQWKKALTLVAN‑MLGFD‑‑SAKWNDEAKMIEEIA

>Q9C7X0

GPDVRIKFLSHLRQQFVYNGITMFDDNGI‑E‑RSQIIAP‑ALKKAIGESRVAIVLLSKNYASSSWCLDELLEILKCKEYIG‑QI‑VMTVFYEVDPSHVRKQTGDFGIAFKETCA‑‑HK‑TE‑EE‑RSKWSQALTYVGN‑IAGED‑‑FIHWKDEAKMIEKIA

>O80617

GEQLRRSFVSHLIDAFERNEINFFVDKY‑‑EQRGKDL‑‑KNLFLRIQESKIALAIFSTRYTESSWCLDELVKIKKLAD‑KK‑KLHVIPIFYKVKVEDVRKQTGEFGDNFWTLAKVS‑‑‑‑SGDQ‑IKKWKEALECIPNK‑MGL‑SLGDK‑SSEADFIKEVV

>F4KG41

GGDVRVTFRSHFLKELDRKLITAFRDNEI‑E‑RSHSLWP‑DLEQAIKDSRIAVVIFSKNYASSSWCLNELLEIVNCND‑‑‑‑KI‑VIPVFYGVDPSQVRHQIGDFGKIFEKTCK‑‑RQ‑TE‑QV‑KNQWKKALTDVAN‑MLGFD‑‑SATWDDEAKMIEEIA

>F4KD45

GAELRHKFISHLLKALERERINVFIDTRE‑T‑MGTGL‑‑ENLFQRIQESKIAIVVISSRYTESQWCLNELVKIKECVEA‑GTLV‑VFPVFYKVDVKIVRFLTGSFGEKLETLVLR‑‑‑‑HS‑ERY‑EPWKQALEFVTS‑KTGKR‑VEEN‑SDEGAEVEQIV

>Q9FT77

GEDVRHSLVSHLRKELDRKFINTFNDNRI‑E‑RSRKITP‑ELLLAIENSRISLVVFSKNYASSTWCLDELVKIQECYEKLD‑QM‑VIPIFYKVDPSHVRKQTGEFGMVFGETCK‑‑GR‑TE‑NE‑KRKWMRALAEVAH‑LAGED‑‑LRNWRSEAEMLENIA

>F4IF04

GKDTRKNFVSFLYKALVSKGIRTFKDDEELE‑RGRPIPP‑ELRQAIKGSRIAVVVVSVTYPASSWCLEELREILKL‑EKLGLLT‑VIPIFYEINPSDVRRQSGVVSKQFKKHE‑‑‑KRQSR‑ER‑VKSWREALTKLAS‑LSGEC‑‑SKNWEDDSKLVDGIT

>F4JU08

GEDQSDGFIRHVERALNDEGFNVFIDDERR‑‑RGEHI‑‑‑‑‑‑RAIDNSNVALVIFSDRYTASELCLHEAVRIYDRRR‑EG‑KV‑LIPVFYRVS‑DDVN‑‑‑GRFGESFLETIQGFDH‑PA‑EH‑‑‑‑WMRNVNFICTD‑TGFT‑‑SADYSSDTSLVEEIV

>F4KHH8

GEDVRNTFLSHFLKELDRKLIISFKDNEI‑E‑RSQSLDP‑ELKHGIRNSRIAVVVFSKTYASSSWCLNELLEIVKCKKEFG‑QL‑VIPIFYNLDPSHVRKQTGDFGKIFEKTCR‑‑NK‑TV‑DE‑KIRWKEALTDVAN‑ILGYH‑‑IVTWDNEASMIEEIA

>F4IFF6

GADVRKSFLSHILKEFKRKGIDTFIDNNI‑E‑RSKSIGP‑ELIEAIKGSKIAVVLLSKDYASSSWCLNELVEIMKCRKMLD‑QT‑VMTIFYEVDPTDVKKQTGDFGKVFKKTCM‑‑GK‑TN‑AV‑SRKWIEALSEVAT‑IAGEH‑‑SINWDTEAAMIEKIS

>F4JNB2

GVDVRKTFLSNLLEAFDRRSINTFMDHGI‑E‑RSRTIAP‑ELISAIREARISIVIFSKNYASSTWCLDELVEIHNRLNDWG‑QL‑VISVFYDVDPSEVRKQTGEFGDVFKKTCE‑‑DK‑EE‑DQ‑KQRWMQALVDITN‑IAGED‑‑LRNGPSEAAMVVKIA

>O23536

GVDVRKTFLSHLIEALDRRSINTFMDHGI‑V‑RSCIIAD‑ELITAIREARISIVIFSENYASSTWCLNELVEIHKCHKDLD‑QM‑VIPVFYGVDPSHVRKQIGGFGDVFKKTCE‑‑DK‑PE‑DQ‑KQRWVKALTDISN‑LAGED‑‑LRNGPSEAAMVVKIA

>F4JNB7

GVDVRKTFLSHLIEALDGKSINTFIDHGI‑E‑RSRTIAP‑ELISAIREARISIVIFSKNYASSTWCLNELVEIHKCFNDLG‑QM‑VIPVFYDVDPSEVRKQTGEFGKVFEKTCEVSDKQPG‑DQ‑KQRWVQALTDIAN‑IAGED‑‑LLNGPNEAHMVEKIS

>F4KFY5

GEDTRHSIVSHLYEALTSRGIATFKDDKRLE‑LGDHISE‑ELQRAIEGSDFVVVVLSENYPTSRWCLMELQSIMEL‑QMEGRLG‑VFPVFYRVEPSAVRYQLGSF‑‑DLEGYQR‑‑DPQ‑A‑DM‑VPKWRQALKLIAD‑LSGVA‑‑SGQCIDEATMVRKIV

>F4I552

GPDVRIKFLSHLRQQFIYNGITMFDDNGI‑E‑RSQIIAP‑ALKKAIGESRIAILLLSKNYASSSWSLDELLEILKCKEDIG‑QI‑VMTVFYEVDPSDVRNQTGDFGIAFKETCA‑‑HK‑TE‑EE‑RQKWTQALTYVGN‑IAGED‑‑FKHWPNEAKMIEKIA

>O82500

GEDVRNNFLSHLLKEFESKGIVTFRDDHI‑K‑RSHTIGH‑ELRAAIRESKISVVLFSENYASSSWCLDELIEIMKCKEEQG‑LK‑VMPVFYKVDPSDIRKQTGKFGMSFLETCC‑‑GK‑TE‑ER‑QHNWRRALTDAAN‑ILGDH‑‑PQNWDNEAYKITTIS

>Q9FHG0

GEDVRKTFLSHFLRELERKSIITFKDNEM‑E‑RSQSIAP‑ELVEAIKDSRIAVIVFSKNYASSSWCLNELLEIMRCNKYLG‑QQ‑VIPVFYYLDPSHLRKQSGEFGEAFKKTCQ‑‑NQ‑TE‑EV‑KNQWKQALTDVSN‑ILGYH‑‑SKNCNSEATMIEEIS

>F4JT81

GADTRHDFTSHLVKYLRGKGIDVFSDAKL‑R‑GGEYI‑‑SLLFDRIEQSKMSIVVFSEDYANSWWCLEEVGKIMQRRKE‑FNHG‑VLPIFYKVSKSDVSNQTGSFEAVFQSPTKIFG‑‑DE‑Q‑KIEELKVALKTASN‑IRGFV‑YPEN‑SSEPDFLDEIV

>Q9SSN9

REDTGRTFVSHLYRSLDQKEIRTYKFNQQAG‑DGKRISS‑EVKQAINESRIAVVVISENYVSSVLCLDVLAKIIERL‑‑‑‑‑LK‑IETVFYEVDPGDLTRPTGKFADDFRRHEARENRRT‑‑‑‑‑VNRWRDALDQLVS‑ITNSN‑CSRNWEDDSKMILGLM

>F4J361

GADVRKTILSHILESFRRKGIDPFIDNNI‑E‑RSKSIGH‑ELKEAIKGSKIAIVLLSKNYASSSWCLDELAEIMKCRELLG‑QI‑VMTIFYEVDPTDIKKQTGEFGKAFTKTCK‑‑GK‑TK‑EY‑VERWRKALEDVAT‑IAGEH‑‑SRNWRNEADMIEKIA

>F4JWL8

GKDVRRTFLSHLLKEFRRKGIRTFIDNDI‑K‑RSQMISS‑ELVRAIRESRIAVVVLSRTYASSSWCLNELVEIKK‑‑‑‑VS‑QM‑IMPVFYEVDPSDVRKRTGEFGKAFEEACE‑‑RQ‑PD‑EE‑VQKWREALVYIAN‑IAGES‑‑SQNWDNEADLIDKIA

>F4J359

GADVRRTFLSHIMESFRRKGIDTFIDNNI‑E‑RSKSIGP‑ELKEAIKGSKIAIVLLSRKYASSSWCLDELAEIMKCRQMVG‑QI‑VMTIFYEVEPTDIKKQTGEFGKAFTKTCR‑‑GK‑PK‑EQ‑VERWRKALEDVAT‑IAGYH‑‑SHSWRNEADMIEKIA

>Q0WQ93

GVDTRQTIVSHLYVALRNNGVLTFKDDRKLE‑IGDTIAD‑GLVKAIQTSWFAVVILSENYATSTWCLEELRLIMQL‑HSEEQIK‑VLPIFYGVKPSDVRYQEGSFATAFQRYEA‑‑DPE‑E‑EK‑VSKWRRALTQVAN‑LSGKH‑‑SRNCVDEADMIAEVV

>Q9FKB8

GLDTRRTFVSHLRRSLDRKGIKTFEDNESL‑‑RG‑ELSS‑AVYQTIGESKVAVVLISVNYASSPLCLDSLLKILKFHQ‑SGSLV‑LIPIFYEVDPMDVRKQIGKY‑EAFSLHER‑‑‑‑‑‑EPEK‑VQTWRQALSQLVS‑IGGQY‑‑SE‑WDGDAELIHQIT

>Q6NPD9

GEKLRDGFLGFLVDALLKENVNVFIDDH‑‑ELRGRDL‑‑DHLFSRIEESRVALTIFSKNFTNSRWCLDELAKIKECVD‑QE‑SLTVIPIFFKMKTDDVKKLKGNFGDNFRDLKLTH‑R‑GEPET‑YRRWKDAILYVSKK‑TGL‑SSSRY‑SR‑NDLVNTIV

>F4KDB8

GEDVRKSFLSHLLKKLHRKSINTFIDNNI‑E‑RSHAIAP‑DLLSAINNSMISIVVFSKKYASSTWCLNELVEIHKCYKELT‑QI‑VIPIFYEVDPSDVRKQTREFGEFFKVTCV‑‑GK‑TE‑DV‑KQQWIEALEEVAS‑IAGHD‑‑SKNWPNEANMIEHIA

>Q9FXA6

GPDVRKTFLSHLRKQFNYNGITMFDDQRI‑E‑RSQIIAP‑ALTEAIRESRIAIVLLSKNYASSSWCLDELLEILDCKEQLG‑QI‑VMTVFYGVHPSDVRKQTGDFGIAFNETCA‑‑RK‑TE‑EQ‑RQKWSQALTYVGN‑IAGEH‑‑FQNWDNEAKMIEKIA

>Q9M0P9

GADTRNNIVSYLHKALVDVGIRTFKDDKELE‑EGDIISE‑KLVNAIQTSWFAVVVLSEKYVTSSWCLEELRHIMEL‑SIQDDII‑VVPIFYKVEPSDVRYQKNSFEVKLQHYR‑‑‑DPE‑‑‑‑K‑ILKWKGALTQVGN‑MSGKH‑‑FQTCSDEATNIAEIV

>A8MR18

GKDLRKGFMSFLKPALKKEKINVFIDEQ‑‑EERGKYL‑‑ISLFDTIGESKIALVIFSEGYCESHWCMDELVKIKEYMD‑QN‑RLIIIPIFYRLDLDVVKDLTGKFGDNFWDLVDKY‑Q‑PEPKK‑LHKWTEALFSVCEL‑FSL‑ILPKH‑SDDRDFVKSIV

>O48573

GADLRNGFISHLAGALTSAGITYYIDTEE‑V‑PSEDL‑‑TVLFKRIEESEIALSIFSSNYAESKWCLDELVKIMEQVKK‑GKLR‑IMPVFFNVKPEEVREQNGEFGLKLYGEGKS‑‑‑‑‑K‑RPNIPNWENALRSVPS‑KIGLN‑LANF‑RNEKELLDKII

>F4IBL4

GEDVRRGFLSHIHKEFQRKGITPFIDNEI‑K‑RGESIGL‑EIIHAIRESKIAIVLLSRNYASSSWCLDELVEIMKCKEEFS‑QI‑VIPIFYRVDPSDVKKLTGNFGNVFKNNCV‑‑GK‑TN‑EV‑IRKWRQALAKMGT‑TTGYD‑‑SRNWDNEATMIENIA

>Q9FHF0

GEDLRLGFVSHLVEALENDNIKVFIDNYA‑D‑KGEPL‑‑ETLLTKIHDSKIALAIFSGKYTESTWCLRELAMIKDCVEK‑GKLV‑AIPIFYKVDPSTVRGVRGQFGDAFRDLEER‑‑‑‑‑D‑VIKKKEWKQALKWIPG‑LIGIT‑VHDK‑SPESEILNEIV

>Q9SKM4

GEQLRRSFVSHLIDAFERNEINFFVDKY‑‑EQRGKDL‑‑KNLFLRIQESKIALAIFSTRYTESSWCMDELVKIKKLAD‑KR‑KLHVIPIFYKVKVEDVRKQTGEFGDNFWTLAKVS‑‑‑‑SGDQ‑IKKWKEALECIPNK‑MGL‑SLGDK‑SDEADFIKEVV

>F4KD49

KNDSSVSFISYLMAGFGCRGIKQF‑‑‑‑‑‑‑‑‑‑‑‑‑‑‑‑‑‑‑‑‑‑‑‑LGIYLVILSRDYASSVLCLENLELCCDDKKSYE‑‑‑‑VVPVFYGVSRSDVRQQSGPFSDAFTKLER‑‑‑‑‑SA‑DH‑VTKWRRMFAKIAE‑LKGHE‑‑YDE‑SEESEFVEEIV

>Q9FFS6

GSDVRRKFLSHLRFHFAIKGIVAFKDQEI‑E‑RGQRIGP‑ELVQAIRESRVSLVVLSKNYPSSSWCLDELVEILKCKEDQE‑QI‑VMPIFYEIDPSDVRKQSGDFGKAFGKTCV‑‑GK‑TK‑EV‑KQRWTNALTEAAN‑IGGEH‑‑SLNWTDEAEMIEKIV

>F4I820

GDELRNSFVGFLVKAMRLEKINVFTDEV‑‑ELRGTNL‑‑NYLFRRIEESRVAVAIFSERYTESCWCLDELVKMKEQME‑QG‑KLVVVPVFYRLNATACKRFMGAFGDNLRNLEWEY‑R‑SEPER‑IQKWKEALSSVFSN‑IGL‑TSDIR‑SN‑SKFVDSIV

>F4JWM0

GEDVRKGFLSHIQKEFKSKGIVPFIDDEM‑K‑RGESIGP‑GLFQAIRESKIAIVLLSKNYASSSWCLNELVEIMNCREEIG‑QT‑VMTVFYQVDPSDVRKQTGDFGKAFKKTCV‑‑GK‑TQ‑EV‑KQRWSRALMDVAN‑ILGQD‑‑SRKWDKEADMIVKVA

>F4JNL2

GKDERNGLLTLLKQKLIDGNVNVFT‑DD‑‑KLTGQPL‑‑QNLFGHIRKSRIAIVIFSKNYAESGWCLDELVEIKKCFE‑TE‑ALAVIPIFHRVKVSSVKKQSGKFGEKFLALQNYLDKKK‑‑SR‑IKRWKKALKIVTEI‑AGL‑THDKN‑SPELAFVEKVV

>O81430

VEDIRQTFLSHFLKDLDRKLIIAFKDNEI‑E‑RSQSLNP‑DLKRPIRDSRIAIVIFSKNYASSSWCLNELLEIVRCKEDSNRLV‑VIPVFYGLDPSHVRKQIGNFGKIFKKTCQ‑‑NR‑TE‑DE‑INLRRRALIDVAN‑TLGYH‑‑STIC‑‑KANMTKEIT

>F4IEZ7

GVDTRQTIVSHLYVALRNNGVLTFKDDRKLE‑IGDTIAD‑GLVKAIQTSWFAVVILSENYATSTWCLEELRLIMQL‑HSEEQIK‑VLPIFYGVKPSDVRYQEGSFATAFQRYEA‑‑DPE‑E‑EK‑VSKWRRALTQVAN‑LSGKH‑‑SRNCVDEADMIAEVV

>F4JYI5

GPDVRKGFLSHLHSLFASKGITTFNDQNI‑E‑RGQTIGP‑ELIQGIKEARVSIVVLSKNYASSSWCLDELVEILKCKEALG‑QI‑VMT‑‑‑‑‑‑‑‑‑‑‑‑‑SGVFGKAFEKTCQ‑‑GK‑NE‑EV‑KIRWRNALAHVAT‑IAGEH‑‑SLNWDNEAKMIQKIA

>Q9SCZ3

SEDTVRSFVSHLCAAFRRRGISSFIRE‑N‑G‑SDES‑‑‑‑‑‑‑‑‑LETSRASVVVFSEKYSSSKSCMEELVKVSERRRKNC‑LA‑VVPVFYPVTKSFMKKQIWNLG‑‑‑‑‑‑‑‑‑‑‑‑‑‑‑‑DV‑RSDWPSALLETVD‑LPGHE‑‑LYD‑QSDSDFVEEIV

>Q9FLA7

GNESRDNFIKYLVWGLRDERVNVFVDRA‑‑EANRRDI‑‑RNISTKIEESNIAVVIFSKRYTESEMCLNELQKMYEHVE‑QS‑NLKVIPVFYDVSISGVKNLEDEFGNHFEELREKY‑A‑NDPLK‑ILKWEDSLSSIVE‑‑‑GL‑TSEDH‑GTGLGLVRAIV

>Q9SSN3

GHDTRHNFISFLYKELVRRSIRTFKDDKELE‑NGQRFSP‑ELKSPIEVSRFAVVVVSENYAASSWCLDELVTIMDF‑EKKGSIT‑VMPIFYGVEPNHVRWQTGVLAEQFKKHA‑‑‑SREDP‑EK‑VLKWRQALTNFAQ‑LSGDC‑‑SG‑‑DDDSKLVDKIA

>F4I270

GDDVRRNFLSHIQKEFRRKGITPFIDNEI‑R‑RGESIGP‑ELIKAIRESKIAIVLLSRNYASSKWCLEELVEIMKCKKEFG‑LT‑VFAIFYEVDPSHVKKLTGEFGAVFQKTCK‑‑GR‑TK‑EN‑IMRWRQAFEEVAT‑IAGYD‑‑SRNWENEAAMIEEIA

>Q9FKR7

GPDVRSGFLSHLHNHFESKGITPFKDQEI‑E‑RGHTIGP‑ELIQAIRESRVSIVVLSEKYASSCWCLDELVEILKCKEASG‑QV‑VMTIFYKVDPSDVRKQRGDFGSTFKKTCE‑‑GK‑TW‑IV‑KQRWIKALEYIAT‑VAGEH‑‑SLSWANEAELIQKIA

>Q9SSN1

GLDTRRSFISFLYKELIRRNIRTFKDDKELK‑NGRRITP‑ELIRAIEGSRFAVVVVSVNYAASRWCLEELVKIMDF‑ENMGSLK‑VMPIFYGVDPCHVRRQIGEVAEQFKKHE‑‑‑GREDH‑EK‑VLSWRQALTNLAS‑ISGDC‑‑SWKWEDDSKMVEEIT

>F4I902

G‑‑‑‑‑KIALDVDYDLSRNGIKAFKSESWKS‑SFKPIDL‑RTLEALTESKVAVVMTSDEEVSSVGFLEELIVIIEFQ‑EKRSLT‑VIPVFLTKHPLDV‑EKVSQF‑‑‑‑‑‑‑‑‑‑‑‑‑‑‑P‑ER‑AKIWRTAIAKLDN‑IAAQY‑FSRN‑MHGTHRIKQIA

>Q9C5Q9

GKDLRKGFMSFLKPALKKEKINVFIDEQ‑‑EERGKYL‑‑ISLFDTIGESKIALVIFSEGYCESHWCMDELVKIKEYMD‑QN‑RLIIIPIFYRLDLDVVKDLTGKFGDNFWDLVDKY‑Q‑PEPKK‑LHKWTEALFSVCEL‑FSL‑ILPKH‑SDDRDFVKSIV

>F4K382

GLDTRRTFVSHLRRSLDRKGIKTFEDNESL‑‑RG‑ELSS‑AVYQTIGESKVAVVLISVNYASSPLCLDSLLKILKFHQ‑SGSLV‑LIPIFYEVDPMDVRKQIGKY‑EAFSLHER‑‑‑‑‑‑EPEK‑VQTWRQALSQLVS‑IGGQY‑‑SE‑WDGDAELIHQIT

>Q9CAK1

GPDVRKTLLSHIRLQFNRNGITMFDDQKI‑V‑RSATIGP‑SLVEAIKESRISIVILSKKYASSSWCLDELVEILECKKAMG‑QI‑VMTIFYGVDPSDVRKQIGKFGIAFNETCA‑‑RK‑TE‑EE‑RQKWSKALNQVSN‑IAGED‑‑FLRWDNEAIMIEKIA

>F4IF00

GEDTRKNIVSHLHKQLVDKGVVTFKDDKKLE‑LGDSISE‑EISRAIQNSTYALVILSENYASSSWCLDELRMVMDL‑HLKNKIK‑VVPIFYGVDPSHVRHQTGSF‑‑TFDKYQ‑‑‑DSK‑P‑NK‑VTTWREALTQIAS‑LAGKD‑‑FETCEDEASMIEEIV

>Q9FHE5

EEDVSKGLINFLEPVLQNKNINVFIDEE‑‑EVRGKGL‑‑KNLFKRIQDSKISLAIFSESKCD‑‑‑‑FNDLLKNNESAD‑E‑‑‑‑‑AIPIFYKVDAT‑‑‑‑‑‑‑‑‑GD‑‑‑‑‑‑‑‑‑‑‑‑‑‑‑‑‑‑‑‑‑‑‑‑‑‑‑‑‑‑‑‑‑‑‑‑‑‑LADL‑QNKKDLINSAV

>Q9SUS7

GSDVR‑NFFSFLKDALIKNGINVVTDEDA‑‑‑RGKPI‑‑ENLLKLIKDSRIAVVIFSENYPESTWCLDELVEIEKQMDK‑‑‑MLDSCPIFFEVETCHVKRST‑‑FNYNLLQLARQISKKAEAEK‑‑REWRKALISVASR‑LGLT‑‑YKK‑SNQATFVNEIV

>Q9FVT6

GKDIRHGFVSHLKDALKRKNINFFIDTH‑‑EQKGRDL‑‑NHLFKRIEEATIALVILSPRYGESKWCLEELTTIMDQEE‑KG‑QMIVIPIFYKVRTEDVEKQTGEFGHMFWSCDEEA‑‑‑‑SLEE‑MEKWQVALKAVCNK‑IGL‑TLDLK‑‑‑EAKFIKKVL

>F4I3S8

GPDVRKTLLSHMRKQFDFNGITMFDDQGI‑E‑RSEEIAP‑SLKKAIKESRISIVILSKKYASSSWCLDELVDILKRKKAMK‑QI‑VMTVFYGVEPFEVRNQTGEFGIAFNETCA‑‑RK‑TD‑EE‑RQKWSKALNEVAN‑IAGED‑‑FLRCDNEAKRIEKIA

>O49471

GDELRNNFVSHLDKALRGKQINVFIDEAV‑E‑KGENL‑‑DNLFKEIEKSRIALAIISQKYTESKWCLNELVKMKEL‑‑E‑GKLV‑TIPIFYNVEPATVRYQKEAFGAALTKTQEN‑‑‑‑SD‑G‑QMKKWKEALTYVSL‑LVGFP‑FNSK‑SKEKELIDKIV

>Q9SSN6

GPDTRRKFISFLYKELVGRDIRTFKDDKELE‑NGQMISP‑ELILAIEDSRFAVVVVSVNYAASSWCLDELVKIMDI‑QNKGSIT‑VMPIFYGVNPCHLRRQIGDVAEQFKKHE‑‑‑AREDL‑EK‑VLKWRQALAALAD‑ISGDC‑‑SG‑‑EDDSKLVDVIA

>Q9C7W9

GPDVRIKFLSHLRQQFIYNGITMFDDNGI‑E‑RSQIIAP‑ALKKAIGESRIAILLLSKNYASSSWSLDELLEILKCKEDIG‑QI‑VMTVFYEVDPSDVRNQTGDFGIAFKETCA‑‑HK‑TE‑EE‑RQKWTQALTYVGN‑IAGED‑‑FKHWPNEAKMIEKIA

>F4I901

GEDTRKTIVSHLYAALDSRGIVTFKDDQRLE‑IGDHISD‑ELHRALGSSSFAVVVLSENYATSRWCLLELQLIMEL‑MKEGRLE‑VFPIFYGVDPSVVRHQLGSF‑‑SLVKYQG‑‑‑LE‑V‑DK‑VLRWREALNLIAN‑LSGVV‑‑SSHCVDEAIMVGEIA

>F4I9F1

GEDVRVTFRSHFLKELDRKLITAFRDNEI‑E‑RSHSLWP‑DLEQAIKESRIAVVVFSKNYASSSWCLNELLEIVNCND‑‑‑‑KI‑VIPVFYHVDPSQVRHQIGDFGKIFENTCK‑‑RQ‑TE‑EV‑KNQWKKALTLVAN‑MLGFD‑‑SAKWNDEAKMIEEIA

>F4HT77

GEEIRHGFISHLADALERYGIMFIIDKD‑‑EQRGNDL‑‑TSLLLRIKESKVALVIFSSRFAESRFCMDEIVKMKECVD‑ER‑KLLVIPIFYKVRARDVSGRTGDFGKKFWALAQKS‑‑‑‑RGCQ‑IKEWMEALECISNK‑MGL‑SLGDG‑RSEADFIKEIV

>Q9FNJ2

GEDVRVTFLSHFLKELDRKLISVFKDNDI‑Q‑RSQSLDP‑ELKLAIRDSRIAIVVFSKNYAASSWCLDELLEIVKCKEEFG‑QI‑VIPVFYGLDPCHVRKQSGEFGIVFENTCQ‑‑TK‑TD‑DE‑IQKWRRALTDVAN‑ILGFH‑‑SSNWDNEATMVEDIA

>Q9CAK0

GPNVRKTLLSHMRKQFNFNGITMFDDQGI‑E‑RSEEIVP‑SLKKAIKESRISIVILSKKYALSRWCLDELVEILKCKEVMG‑HI‑VMTIFYGVEPSDVRKQTGEFGFHFNETCA‑‑HR‑TD‑ED‑KQNWSKALKDVGN‑IAGED‑‑FLRWDNEAKMIEKIA

>Q9SYC9

GDELRNSFVGFLVKAMRLEKINVFTDEV‑‑ELRGTNL‑‑NYLFRRIEESRVAVAIFSERYTESCWCLDELVKMKEQME‑QG‑KLVVVPVFYRLNATACKRFMGAFGDNLRNLEWEY‑R‑SEPER‑IQKWKEALSSVFSN‑IGL‑TSDIR‑RYINKNMDHTS

>F4IUF0

SKDTRDNFVSHLCGCLRRKRIKTFLYDEL‑P‑ADERYEE‑SL‑KAIEVSKISVIVFSENFGDSRWCLDEVVAILKCKEKFG‑QI‑VIPVLYHVDPLDIENQTGSFGDAFAK‑‑R‑‑RD‑KA‑EQ‑LQEWKDSFTEAIN‑LPGWS‑‑TAYLSDEEMLVNGIA

>O65507

GKQLRNGFVSHLEKALRRDGINVFIDRNE‑T‑KGRDL‑‑SNLFSRIQESRIALAIFSSMYTESYWCLDELVKIKDCVDL‑GTLV‑VIPIFYMVDTDDVKNLKGAFGYTFWKLAKT‑‑‑‑CN‑GEKLDKWKQALKDVPK‑KLGFT‑LSEM‑SDEGESINQIV

>Q9FHE9

GKDLRNGFLSFLEPAMREANINVFIDKD‑‑EVVGTDL‑‑VNLFVRIQESRVAVVIFSKDYTSSEWCLDELAEIKDCIN‑QG‑GLNAIPIFYKLAPSSVLELKGGFGDTFRVLKEKY‑K‑NDPER‑TQKWQEALESIPKL‑KGL‑RLAEK‑SDEREFMNEMI

>F4HR52

‑RDARHKFTERLYEVLKEQ‑VRVW‑NDDV‑E‑RGNELGA‑SLVEAMEDSVALVVVLSPNYAKSHWCLEELAMLCDLKSSLG‑RL‑VLPIFYEVEPCMLRKQNGPY‑MDFEEHSKRF‑‑‑SE‑EK‑IQRWRRALNIIGN‑IPGFV‑YSK‑‑SKDDDMIELVV

>Q9ZVX6

GPDVRKSFLSHFRKQFICNGITMFDDQKI‑V‑RSQTIAP‑SLTQGIRESKISIVILSKNYASSTWCLNELLEILKCREDIG‑QI‑VMTVFYGVDPSDVRKQTGEFGTVFNKTCA‑‑RR‑TE‑KE‑RRNWSQALNVVGN‑IAGEH‑‑FLNWDNEAEMIEKIA

>Q9SSP1

SEDPSKTFVSVLDRWLEQKDITNFKDD‑‑‑‑‑‑‑‑‑‑‑S‑FLAE‑‑‑ESKLAVVVVSESYPISVLCLNQLEKIVNS‑HSEGRLS‑ILPIFYGVDPYNVRKQTGYLAEPFQELG‑‑‑‑‑‑PD‑DK‑IQEWRVSLTKLTN‑IPALD‑‑SRYWSNEADMIELIA

>Q9FGT2

GPDVRKTFLSHLRKQFSYNGISMFNDQSI‑E‑RSQTIVP‑ALTGAIKESRISIVVLSKNYASSRWCLDELLEILKCREDIG‑QI‑VMTVFYGVDPSDVRKQTGEFGIAFNKTCE‑‑GK‑TN‑EE‑TQKWSKALNDVGN‑IAGEH‑‑FFNWDNEAKMIEKIA

>Q0WPW2

GEDVRKSFLSHLLKKLHRKSINTFIDNNI‑E‑RSHAIAP‑DLLSAINNSMISIVVFSKKYASSTWCLNELVEIHKCYKELT‑QI‑VIPIFYEVDPSDVRKQTREFGEFFKVTCV‑‑GK‑TE‑DV‑KQQWIEALEEVAS‑IAGHD‑‑SKNWPNEANMIEHIA

>Q9SW60

GEDIRVTFLTHFLKELDRKMIIAFKDNEI‑E‑RGNSIGT‑ELIQAIKDSRIAVVVFSKKYSSSSWCLNELVEIVNCK‑‑‑‑‑EI‑VIPVFYDLDPSDVRKQEGEFGESFKETCK‑‑NR‑TD‑YE‑IQRWGQALTNVAN‑IAGYH‑‑TRKPNNEAKLIEEIT

>Q9FMB7

GGDIRKTFLSHLRKQFNSNGITMFDDQGI‑E‑RSQTIAP‑ALIQAIRESRISIVVLSKNYASSSWCLNELVEILKCKD‑‑‑‑‑V‑VMPIFYEVDPSDVRKQTGDFGKAFKNSCK‑‑SK‑TK‑EE‑RQRWIQALIFVGN‑IAGEH‑‑SLKWENEADMIEKIA

>Q9FKB9

GEDTRRTIVSHLYAALGAKGIITFKDDQDLE‑VGDHISS‑HLRRAIEGSKFAVVVLSERYTTSRWCLMELQLIMEL‑YNLGKLK‑VLPLFYEVDPSDVRHQRGSF‑‑GLERYQ‑‑‑GPE‑A‑DI‑VQRWRVALCMVAN‑LSGMV‑‑SRYCADEAMMLEEIV

>F4JT79

NLDVDESFIEAISKELHKQGFIPLTYN‑‑‑‑‑‑‑‑‑‑‑‑‑‑LLEMLYGSRVGIMILSSSYVSSRQSLDHLVAVMEHWKTTD‑LV‑IIPIYFKVRLSDICGLKGRFEAAFLQLHM‑‑‑‑‑SE‑DR‑VQKWKAAMSEIVS‑IGGHE‑‑WTK‑GSQFILAEEVV

>F4I818

GDELRNSFVGFLVKAMRLEKINVFTDEV‑‑ELRGTNL‑‑NYLFRRIEESRVAVAIFSERYTESCWCLDELVKMKEQME‑QG‑KLVVVPVFYRLNATACKRFMGAFGDNLRNLEWEY‑R‑SEPER‑IQKWKEALSSVFSN‑IGL‑TSDIR‑SN‑SKFVDSIV

>Q9FHE8

GADVRKHFISFLVPALREANINVFIDEN‑‑EFLGSEM‑‑ANLLTRIEESELALVIFSVDFTRSHRCLNELAKIKERKD‑QG‑RLIVIPIFYKVKPSAVKFLEGKFGDNFRALERNN‑R‑HMLPI‑TQKWKEALESIPGS‑IGM‑PLAEQ‑SEDNDFINSMV

>F4ISS7

GKELRKGFISFLVPALKKKNINVFIDEH‑‑EVRGKDL‑‑ISLFRRIGESKIALVIFSEGYTESKWCLDELVQIKKCVD‑QK‑KIIAIPIFYKLDPAVVKGLKGKFGDKFRDLIERY‑H‑HEPER‑YQKWTEALTSVSRT‑FAL‑CLPEH‑SDEKDFIRSII

>Q9LSX5

GPDVRKGFLSHLHSVFASKGITTFNDQKI‑D‑RGQTIGP‑ELIQGIREARVSIVVLSKKYASSSWCLDELVEILKCKEALG‑QI‑VMTVFYEVDPSDVKKQSGVFGEAFEKTCQ‑‑GK‑NE‑EV‑KIRWRNALAHVAT‑IAGEH‑‑SLNWDNEAKMIQKIV

>Q9FH20

GEDVRGNFLSHLMKEFESKGIVTFKDDLI‑E‑RSQTIGL‑ELKEAVRQSKIFVVIFSKNYASSSWCLDELVEILKCKEERR‑L‑‑‑IPIFYKVNPSDVRNQTGKFGRGFRETCE‑‑GK‑ND‑ET‑QNKWKAALTEAAN‑IAGED‑‑SQSWKNEADFLTKIA

>F4JWL7

GEDVRKGFLSHIQKEFERKGIFPFVDTKM‑K‑RGSSIGP‑VLSDAIIVSKIAIVLLSKNYASSTWCLNELVNIMKCREEFG‑QT‑VMTVFYEVDPSDVRKQTGDFGIAFETTCV‑‑GK‑TE‑EV‑KQSWRQALIDVSN‑IVGEV‑‑YRIWSKESDLIDKIA

>Q9FL35

GGDVRVTFRSHFLKEFDRKLITAFRDNEI‑E‑RSHSLWP‑DLEQAIKESRIAVVVFSKNYASSSWCLNELLEIVNCND‑‑‑‑KI‑IIPVFYGVDPSQVRYQIGEFGKIFEKTCK‑‑RQ‑TE‑EV‑KNQWKKALTHVAN‑MLGFD‑‑SSKWDDEAKMIEEIA

>Q9XGM3

GADLRRRFVSHLVTALKLNNINVFIDDYE‑D‑RGQPL‑‑DVLLKRIEESKIVLAIFSGNYTESVWCVRELEKIKDCTDE‑GTLV‑AIPIFYKLEPSTVRDLKGKFGDRFRSMAKG‑‑‑‑‑D‑E‑RKKKWKEAFNLIPN‑IMGII‑IDKK‑SVESEKVNEIV

>F4IF05

GKDTRKNFVSFLYKALVSKGIRTFKDDEELE‑RGRPIPP‑ELRQAIKGSRIAVVVVSVTYPASSWCLEELREILKL‑EKLGLLT‑VIPIFYEINPSDVRRQSGVVSKQFKKHE‑‑‑KRQSR‑ER‑VKSWREALTKLAS‑LSGEC‑‑SKNWEDDSKLVDGIT

>Q9SZ66

GFDTRNNFTGHLQKALRLRGIDSFIDDRL‑R‑RGDNL‑‑TALFDRIEKSKIAIIVFSTNYANSAWCLRELVKILECRNS‑NQQL‑VVPIFYKVDKSDVEKQRNSFAVPFKLPELTFG‑‑VT‑PEEISSWKAALASASN‑ILGYV‑VKES‑TSEAKLVDEIA

>Q93ZC0

GGDVRVTFRSHFLKEFDRKLITAFRDNEI‑E‑RSHSLWP‑DLEQAIKDSRIAVVVFSKNYASSSWCLNELLEIVNCND‑‑‑‑KI‑IIPVFYGVDPSQVRYQIGDFGRIFEKTCK‑‑RQ‑TE‑EV‑KNQWKKALTLVAN‑MLGFD‑‑SAKWDDEAKMIEEIA

>F4KIF3

GVELRKNFVSHLEKGLKRKGINAFIDTDE‑E‑MGQEL‑‑SVLLERIEGSRIALAIFSPRYTESKWCLKELAKMKERTEQ‑KELV‑VIPIFYKVQPVTVKELKGDFGDKFRELVKS‑‑‑‑TD‑KKTKKEWKEALQYVPF‑LTGIV‑LDEK‑SDEDEVINIII

>Q9M1P1

GADVRRTFLSHIMESFRRKGIDTFIDNNI‑E‑RSKSIGP‑ELKEAIKGSKIAIVLLSRKYASSSWCLDELAEIMKCRQMVG‑QI‑VMTIFYEVEPTDIKKQTGEFGKAFTKTCR‑‑GK‑PK‑EQ‑VERWRKALEDVAT‑IAGYH‑‑SHSWRNEADMIEKIA

>F4J3L8

GEDVRKDFLSHIQKEFQRQGITPFVDNNI‑K‑RGESIGP‑ELIRAIRGSKIAIILLSKNYASSSWCLDELVEIIKCKEEMG‑QT‑VIVIFYKVDPSLVKKLTGDFGKVFRNTCK‑‑GK‑ER‑EN‑IERWREAFKKVAT‑IAGYD‑‑SRKWDNESGMIEKIV

>F4JNB8

GVDVRKTFLSHLIEALDGKSINTFIDHGI‑E‑RSRTIAP‑ELISAIREARISIVIFSKNYASSTWCLNELVEIHKCFNDLG‑QM‑VIPVFYDVDPSEVRKQTGEFGKVFEKTCEVSDKQPG‑DQ‑KQRWVQALTDIAN‑IAGED‑‑LLNGPNEAHMVEKIS

>Q9FI14

GEDVRKGLLSHIQKEFQRNGITPFIDNEM‑K‑RGGSIGP‑ELLQAIRGSKIAIILLSRNYGSSKWCLDELVEIMKCREELG‑QT‑VMTVFYDVDPSDVRKQKGDFGKVFKKTCV‑‑GR‑PE‑EM‑VQRWKQALTSAAN‑ILGED‑‑SRNWENEADMIIKIS

>F4J339

GADVRRTFLSHIMESFRRKGIDTFIDNNI‑E‑RSKSIGP‑ELKEAIKGSKIAIVLLSRKYASSSWCLDELAEIMKCRQMVG‑QI‑VMTIFYEVDPTDIKKQTGEFGKAFTKTCR‑‑GK‑PK‑EQ‑VERWRKALEDVAT‑IAGYH‑‑SHSWRNEADMIEKIS

>Q9M0Q0

VAET‑‑‑LVSDLRSSFSENGI‑‑MKDDDLEK‑GVSSLGS‑ERSEGIRESKVAVVVISQSYAISAQCLNELQTIVNF‑HDERRIS‑ILPIFYGVDYDDVRNQIKELAASFRKLG‑‑‑‑‑‑PS‑EK‑VQAWMIALIKLIN‑ISRSD‑‑SRIHDDETTIDMVIT

>Q9SSN5

GHDTRQNFISFLYKELVRRSIRTFKDDKELE‑NGQRISS‑ELKRTIEVSRFAVVVVSETYAASSWCLDELVTIMDF‑EKKGSIT‑VMPIFYGVEPNHVRWQTGVLAEQFKKHG‑‑‑SREDH‑EK‑VLKWRQALTNFAQ‑LSGDC‑‑SG‑‑DDDSKLVDKIA

>Q0WSX8

GDELREIFVNHLELQLRNAGINVFIDTK‑‑EQKGRRL‑‑QYLFTRIKKSKIALAIFSKRYCESKWCLDELVTMNEQMK‑EK‑KLVVIPIFYNVRSDDVKNLDGEFSLPFKQLKQNH‑A‑GEPER‑VEGWERALRSVTK‑‑FSR‑SNSKY‑KHDTDFVLDIV

>F4KHI3

GKDVRVTFRSHFLKELDRKLISAFRDNEI‑E‑RSHSLWP‑DLEQAIKDSRIAVVVFSKNYASSSWCLNELLEIVNCND‑‑‑‑KI‑IIPVFYGVDPSQVRYQIGEFGSIFEKTCK‑‑RQ‑TE‑EV‑KNQWKKALTDVAN‑MLGFD‑‑SAKWDDEAKMIEEIA

>Q4PT31

GKDTRRTFISFLYKELIEMGIRTFKDDVELQ‑SGRRIAS‑DLLAAIENSKIAVVIISKNYSASPWCLQELVMIMDV‑EKKGSII‑VMPIFYNVEPAHVRRQIEQVAKQFRKHE‑‑‑GRENY‑ET‑VVSWRQALTNLAS‑ISGHC‑‑SRDCEDDSKLLDEIT

>F4I3S7

GPDVRKTLLSHMRKQFDFNGITMFDDQGI‑E‑RSEEIAP‑SLKKAIKESRISIVILSKKYASSSWCLDELVDILKRKKAMK‑QI‑VMTVFYGVEPFEVRNQTGEFGIAFNETCA‑‑RK‑TD‑EE‑RQKWSKALNEVAN‑IAGED‑‑FLRCDNEAKRIEKIA

>B3H776

GADVRKNFLSHLYDSLRRCGISTFMDVEL‑Q‑RGEYISP‑ELLNAIETSKILIVVLTKDYASSAWCLDELVHIMKSHKNPS‑HM‑VFPIFLYVDPSDIRWQQGSYAKSFSKHKNSHNK‑‑‑‑‑‑‑LKDWREALTKVAN‑ISGWD‑‑IKN‑RNEAECIADIT

>Q9FFS5

GPDVRKGFLSHLHYHFASKGITTFKDQEI‑E‑KGNTIGP‑ELVNAIRESRVSIVLLSKKYASSSWCLDELVEILKCKEDQG‑QI‑VMTIFYDVDPSSVRKQKGDFGSTFMKTCE‑‑GK‑SE‑EV‑KQRWTKALTHVAN‑IKGEH‑‑SLNWANEADMIQKIA

>Q9FJG4

GEDVRRNFLSHLHKELQHNGIDAFKDGGI‑K‑RSRSIWP‑ELKQAIWESKIFIVVLSKNYAGSCWCLDELVEIMECREVVG‑KT‑LVPIFYDVDPSSVRKQTGDFGKAFDKICD‑‑VR‑TE‑EE‑RQRWRQALTNVGN‑IAGEC‑‑SSKWDNDAKMIEKIV

>F4JU10

GNDLRKGFVSHVVKALKDARVNVFVDNDH‑ERRGHDD‑‑HLFVRRIHNSKLALVIFSDQYAESQQCLNELTTIHERVA‑EG‑KLMVIPIFYKVNIEEVNNLEGRFGKCFDEMVRTQ‑GRQNHQL‑THHIVGCLRSIARK‑PSF‑‑‑‑‑‑‑SGDSDLVEAII

>F4JNA9

GVDVRKTFLSHLIEALDRRSINTFMDHGI‑V‑RSCIIAD‑ALITAIREARISIVIFSENYASSTWCLNELVEIHKCYKKGE‑QM‑VIPVFYGVDPSHVRKQIGGFGDVFKKTCE‑‑DK‑PE‑DQ‑KQRWVKALTDISN‑LAGED‑‑LRNGPTEAFMVKKIA

>Q9C515

GPDVRNTFLSHLRKQFNTNGITMFDDQRM‑E‑RSQTLAP‑TLTQAIRESKIYIVLLSKNYASSSWCLDELLEILNCKEKRG‑QR‑VMTIFYGVNPSDVRKQTGEFGIAFNETCA‑‑RK‑TE‑EE‑RRKWSHALTCVGN‑ITGVH‑‑VQDRDDEANMIEKIA

>F4JU09

GKELRHGFVSHVVKALRIAGVNVFIDSN‑‑EMKGRDL‑‑QNLFKRIENSKMALVIFSDRFSESDWCLNELVKIDDCVK‑EG‑KLTVIPVFYRVNTDDVKNFKGKFGSCFIETVQRQ‑SPKEEPM‑AERWVNSVKSISSK‑TGF‑‑‑‑‑‑‑SEDSYLVDAIV

>Q9SS05

GEDVRRGFLSHIHKEFQRKGITPFIDNEI‑K‑RGESIGL‑EIIHAIRESKIAIVLLSRNYASSSWCLDELVEIMKCKEEFS‑QI‑VIPIFYRVDPSDVKKLTGNFGNVFKNNCV‑‑GK‑TN‑EV‑IRKWRQALAKMGT‑TTGYD‑‑SRNWDNEATMIENIA

>F4JYI4

GPDVRKGFLSHLHSLFASKGITTFNDQNI‑E‑RGQTIGP‑ELIQGIKEARVSIVVLSKNYASSSWCLDELVEILKCKEALG‑QI‑VMT‑‑‑‑‑‑‑‑‑‑‑‑‑SGVFGKAFEKTCQ‑‑GK‑NE‑EV‑KIRWRNALAHVAT‑IAGEH‑‑SLNWDNEAKMIQKIA

>F4IMF2

GSELRYTFVYYLRTALVKNGINVFTDNME‑P‑KGRNQ‑‑KILFKRIEESKIALAIFSSRYTESSWCLEELVKMKECMDA‑EKLV‑IIPIFYIVTPYTIKKQMGDFGDKFRVLVDY‑‑‑‑VD‑DVTEKKWTDALKSVPL‑ILGIT‑YDGQ‑SEEQLLINQIV

>Q9SSN4

GLDTRRNFISFLYKELVRRKIRTFKDDKELE‑NGRRISP‑ELKRAIEESKFAVVVVSVNYAASPWCLDELVKIMDF‑ENKGSIT‑VMPIFYGVDPCHLRRQIGDVAEQFKKHE‑‑‑AREDH‑EK‑VASWRRALTSLAS‑ISGEC‑‑SLKWEDEANLVDEIA

>Q9FKE4

SGIETRSFVSHLSAAFRRRSVSVCLGGDC‑T‑‑DVT‑‑‑‑‑‑PKTNEGCKVFVVVFSEDYALSKQCLDTLVEF‑‑LEKDDG‑LV‑IVPVYYGVTESMVKQQTERFGVAFTQHQN‑‑‑‑‑NY‑DQ‑VAKWRDCLIQTAS‑LPGHE‑‑LNL‑QEDSEFVEKIV

>Q9LUJ8

‑‑‑‑‑‑CQTSHGYNILIRNGIRTFLSYRCLK‑RFGPIGQ‑RTLKALEESRVAVVMTSTTKPCSVGFLEELLVILEFQ‑EKGSLM‑VIPIFLTDLSFNV‑EEICRH‑‑‑‑‑‑‑‑‑‑‑‑‑‑‑P‑EK‑APSWRTALTKLTN‑LAAEY‑LSQN‑MDQSDLLNQIA

>Q9FHF4

GTDVRRNFLSHLLKGL‑HKSVNSFRDQNM‑E‑RSQSLDP‑MLKQAIRDSRIALVVFSKNYASSSWCLNELLEIVKCKEEFG‑QM‑VIPIFYCLDPSHVRHQDGDFGKNFEETCG‑‑RN‑TE‑EE‑KIQWEKALTDVAN‑LAGFD‑‑SVTWDDEAKMIEEIA

>F4KBQ6

GDELREIFVNHLELQLRNAGINVFIDTK‑‑EQKGRRL‑‑QYLFTRIKKSKIALAIFSKRYCESKWCLDELVTMNEQMK‑EK‑KLVVIPIFYNVRSDDVKNLDGEFSLPFKQLKQNH‑A‑GEPER‑VE‑‑‑‑‑‑‑‑‑‑‑‑‑FSR‑SNSKY‑KHDTDFVLDIV

>Q9FKM9

GADVRKTFLSHMLKEFKRKGIVPFIDNDI‑D‑RSKSIGP‑ELDEAIRGSKIAIVMLSKNYASSSWCLNELVEITKCRKDLN‑QT‑VMTIFYGVDPTDVKKQTGEFGKVFERTCE‑‑SK‑TE‑EQ‑VKTWREVLDGAAT‑IAGEH‑‑WHIWDNEASMIEKIS

>Q9FHF6

GGDVRVTFRSHFLKEFDRKLITAFRDNEI‑E‑RSHSLWP‑DLEQAIKDSRIAVVVFSKNYASSSWCLNELLEIVNCND‑‑‑‑KI‑IIPVFYGVDPSQVRYQIGDFGRIFEKTCK‑‑RQ‑TE‑EV‑KNQWKKALTLVAN‑MLGFD‑‑SAKWDDEAKMIEEIA

>Q9CAD8

GPDVRKTVLSHLRKQFICNGITMFDDQRI‑E‑RGQTISP‑ELTRGIRESRISIVVLSKNYASSSWCLDELLEILKCKEDIG‑QI‑VMTVFYGVDPSDVRKQTGEFGIRFSETWA‑‑RK‑TE‑EE‑KQKWSQALNDVGN‑IAGEH‑‑FLNWDKESKMVETIA

>O23530

GEDVRDSFLSHLLKELRGKAI‑TFIDDEI‑E‑RSRSIGP‑ELLSAIKESRIAIVIFSKNYASSTWCLNELVEIHKCYTNLN‑QM‑VIPIFFHVDASEVKKQTGEFGKVFEETCK‑‑AK‑SE‑DE‑KQSWKQALAAVAV‑MAGYD‑‑LRKWPSEAAMIEELA

>F4KIC7

GEDVRKTFVSHLFCEFDRMGIKAFRDDLDLQ‑RGKSISP‑ELIDAIKGSRFAIVVVSRNYAASSWCLDELLKIMECNKD‑‑‑‑T‑IVPIFYEVDPSDVRRQRGSFGEDVESHS‑‑‑‑‑‑DK‑EK‑VGKWKEALKKLAA‑ISGED‑‑SRNW‑DDSKLIKKIV

>Q9SUK4

GEDVRKNFLSHLLKQLNRRSINTFMDHVI‑E‑RSCIIAD‑ALISAIREARISIVIFSKNYAASTWCLNELVEIDNCSKYFG‑QK‑VIPVFYDVDPSHVRKQIGEFGKVFKKTCE‑‑DK‑PA‑DQ‑KQRWVKALTDISN‑IAGED‑‑LRNGPNDAHMVEKIA

>Q8H1N6

GADVRKSFLSHIMKEFKSKGIDIFIDKDI‑K‑RGKSIGP‑ELTEAIRGSRVAIVFLSRKYASSSWCLNELALIMKCRKELG‑LT‑VMTLFYDLDPTDVRKQTGDFGMAFKETCK‑‑GK‑TK‑DE‑IGRWRHALEEVAK‑IAGYH‑‑SSIWDNEADMIGIVT

>F4I594

GSDVRTSFLSHFRKQFNNNGITMFDDQRI‑L‑RGETISP‑ALTQAIRESRISIVLLSKNYASSGWCLDELLEILKCKDDMG‑QI‑VMTVFYGVDPSDVRKQTGEFGIAFNETCA‑‑CR‑TE‑EE‑RQKWSQALNYVGN‑IAGEH‑‑LLNWDNEAKMIEKIA

>Q9SSN2

GVDTRRNFISFLYKEFVRRKIRTFKDDKELE‑NGRRISP‑ELKRAIEESKFAVVVVSVNYAASPWCLDELVKIMDF‑ENKGSIT‑VMPIFYGVDPCHLRRQIGDVAEQFKKHE‑‑‑AREDH‑EK‑VASWRRALTSLAS‑ISGDC‑‑SSKCEDEAKLVDEIA

>F4JT80

GADTRHDFTSHLVKYLRGKGIDVFSDAKL‑R‑GGEYI‑‑SLLFDRIEQSKMSIVVFSEDYANSWWCLEEVGKIMQRRKE‑FNHG‑VLPIFYKVSKSDVSNQTGSFEAVFQSPTKIFG‑‑DE‑Q‑KIEELKVALKTASN‑IRGFV‑YPEN‑SSEPDFLDEIV

>Q9M285

GADVRRTFLSHIKESFRRKGIDTFIDNNI‑E‑RSKSIGP‑ELKEAIKGSKIAIVLLSRKYASSSWCLDELAEIMKCREMVG‑QI‑VMTIFYEVEPTDIKKQTGEFGKAFTKTCR‑‑GK‑TK‑EH‑IERWRKALEDVAT‑IAGYH‑‑SHKWCDEAEMIEKIS

>Q9SCZ2

GEELRNSFVSHLRSALVRHGVNIFIDTNE‑E‑KGKPL‑‑HVFFQRIEESRIALAIFSVRYTESKWCLNELVKMKECMDK‑GKLL‑IIPIFYKVKAYEVRYQKGRFGCVFKNL‑RN‑‑‑‑VD‑VHKKNQWSEALSSVAD‑RIGFS‑FDGK‑SDEHNFINGIV

>Q9FKE2

GDELRKTFISHLHKRLQRDGINAFIDSDE‑A‑VGEEL‑‑KNLFKRIENSEIALAVLSSRYTESHWCLQELVKMMECSMKNKKLL‑VIPIFYKLKIDTVKELDGDFGRNLWDLWRK‑GC‑DR‑DSRIVKWNEALKYFLS‑RNALV‑FSET‑GKEEEFVSTIA

>Q9LFN1

GEDVRRDFLSHIQMEFQRMGITPFVDNEI‑K‑RGESIGP‑ELLRAIRGSKIAIILLSRNYASSKWCLDELVEIMKCREEYG‑QT‑VMAIFYKVDPSDVKNLTGDFGKVFRKTCA‑‑GK‑PK‑KD‑IGRWRQAWEKVAT‑VAGYH‑‑SINWDNEAAMIKKIA

>F4HR53

GADTRDNFGDHLYKALKDK‑VRVFRDEGM‑E‑RGDEISS‑SLKAGMEDSAASVIVISRNYSGSRWCLDELAMLCKMKSSLD‑RR‑ILPIFYHVDPSHVRKQSDHI‑KDFEEHQVRF‑‑‑SEKEK‑VQEWREALTLVGN‑LAGYV‑CDK‑‑SKDDDMIELVV

>V4LSJ3

GPDVRKGFLSHLHDLFARKEITIFKDQEI‑E‑RGQTIGS‑ELVKAIREARLSIVLLSKNYASSSWCLDELVEILKCREVQG‑QI‑VMPIFYDVDPSHVRKQRGDFGIAFEKTCE‑‑GE‑TE‑EV‑KQRWVEALTCVAT‑IAGEH‑‑SRNWTDEAEMVEKLS

>V4MFB1

GEDVREEFLSHIKKEFGRKGITPFNDNGI‑K‑RGESIGP‑ELIRAIRESKIAIVLLSKNYASSKWCLDELVEIMKCREELG‑QT‑VMAVFYKVDPSDVKKLLGDFGKVFRKTCA‑‑GK‑RK‑ED‑IGRWRQALEKVAT‑IAGYH‑‑SNNWDDEAAMIEKIA

>V4L7N8

SDETIRSLVPHLSAAFGRKGISVLTDK‑‑‑‑‑‑‑‑‑‑‑‑‑‑‑‑DEFSMSIASVLIFSKNYASSKESLDDFLKTIQRRHDKG‑HV‑VTTVFCGVSRSNVK‑‑‑ANFAKALFEHWT‑‑‑‑‑‑S‑YQ‑ASQWCNALEEIAS‑LPGHE‑‑DIN‑KSDCEFVEKIA

>V4MHR6

GADVRKKLVSHLNDALNEEGIKTFHDDRDLQ‑RGDVIWE‑ALEEAINQSRFAIVVISEGYADSHWCLRELSLMVDL‑AEKKRLQ‑LIPIFYEIDPSNLKSRTGCFSKAFEKHE‑‑‑PRFGK‑ET‑VKPWRSALATVGN‑ISGWD‑‑SKCRNEDSKLVQNVV

>V4KFI3

GADVRRSFLSHIMKEFRSKGISPFVDNEI‑K‑RGEFIGP‑ELKTAIQGSRIALVLLSKNYASSSWCLDELVEIMKCKEDLG‑QT‑VKPIFYEVDPTDIKKQTRDFGKVFNKTCE‑‑GK‑TN‑EV‑IGKWSQALEIVAT‑IAGYH‑‑SNKWRDEATMIEDIA

>V4KQN6

RPDIRKTLLSHLREQFNLKGITMFDDNNI‑N‑RGEDLDP‑SLKEAIRESKI‑‑‑‑‑‑‑‑‑‑‑‑‑‑SLQEICFIKLSKNAMK‑QI‑VMTTFYRVETSYVRKQTGEFRIAFNETCA‑‑RK‑TD‑KE‑KQKWSKALTGVSN‑IAGED‑‑FKNWDNEAYMIKKIA

>V4KGM7

GEDTRGNITKRLYEALEKEKIRVFFDDGM‑K‑KGEEVDP‑SLVAGMEDSAASVIILSPKYADSHWCLDELAMLCDLKKSLN‑RL‑MIPIFYMVDPSNVRKQNAHF‑KDFENHAKRF‑‑‑SE‑EK‑IQRWKRAMTLVGN‑LSGFV‑CKED‑SADDEMIELVV

>V4KFF3

GPDVRRGFLSHLHNHFASKGITTFKDQRI‑E‑RGHTIGP‑ELVQAIRESRVSIVLLSKNYASSSWCLDELVEILKCKEASG‑QI‑VITIFYDVDPSDVRKQRGDFGSTFMKTCE‑‑GK‑AE‑GE‑KKRWIKALAYVAT‑IAGEH‑‑SLNWDDEAAMVEKIA

>V4KSP7

GEELRENFVSHLTDAFERHEIKFFIDKD‑‑EQRGKDL‑‑KHLFVRMEESSIALAIFSTRYPESTWCMEELVKMKKLEE‑QG‑KLQVIPIFYKVEAQDVRGQEGKFGENFWMLARAS‑‑‑‑SGEQ‑IKEWKEALEYISKK‑MGL‑SLGDK‑SSEADFVKKIV

>V4M3H5

GPDVRRGFLSHLHDLFARKEITIFKDQEI‑E‑RGHTIGS‑ELVKAIREARLSIVLLSKNYASSSWCLDELVEILKCREAQG‑QI‑VMPIFYDVDPSSVRKQSGDFGIAFEKTCE‑‑GE‑TE‑EV‑KQRWVEALTCVAT‑IAGEH‑‑SRNWTDEAAMVEKLS

>V4M3H1

GPDVRKGFLSHLHDLFARKEITIFKDQEI‑E‑RGQTIGS‑ELVKAIREARLSIVLLSKNYASSSWCLDELVEILKCREVQG‑QI‑VMPIFYDVDPSHVRKQRGDFGIAFEKTCE‑‑GE‑TE‑EV‑KQRWVEALTCVAT‑IAGEH‑‑SRNWTDEAEMVEKLS

>V4L8Q0

GPDVRRGFLSHLHDLFARKEITIFKDQEI‑E‑RGHTIGS‑ELVKAIREARLSIVLLSKNYASSSWCLDELVEILKCREAQG‑QI‑VMPIFYDVDPSSVRKQSGDFGIAFEKTCE‑‑GE‑TE‑EV‑KQRWVEALTCVAT‑IAGEH‑‑SRNWTDEAAMVEKLS

>V4N330

GAELRKNFVSHLKDALERNGINAYIDSNE‑H‑AGEDL‑‑DILFRRIEESTVALTILSRRYTESHWCLGELVHIMKCVDR‑RTLW‑VIPIFYKLEPGTVKKLDGEFGVQLWNLWRK‑G‑‑‑R‑DDRILKWDAALQGVAK‑KIALE‑SEIS‑RDEVAFLDKII

>V4L7H1

RDELRDNFIRYLVWALIDERINVFIDRG‑‑EANKREI‑‑RNFSTMIEDSDIAVVIFSKRYTESEICLNELQKMHEHAE‑QN‑RLRVIPVFYDVSTSDVKNLEGEFGTHFKEMKEKY‑R‑NDPLK‑FLNWEGSLSSIAC‑‑‑GL‑TSEEH‑GTGLGLVREIV

>V4KZZ6

GEELRNSFVSHLADDFKRHGIDFFVDNS‑‑ELRGKDL‑‑KTLFERIEESRIALAIFSTRYAESRWCLDELVKMKKRAD‑KK‑KLHVIPIFYKVRARDVKEQEGEFGEHFRKLARAS‑‑‑‑SGNQ‑IKKWKEALERISEK‑MGL‑PLKDN‑STEAGFVKEIV

>V4NKA3

GPDVRKGLLSHLHDHFLRKEIKMYKDDKM‑E‑RCNTIKH‑ELVKAIRESRVLMVLLSKNYASSIWCLDELVEILKCRKDKE‑QI‑VMPIFYDVDPFHVRTQSGDFGSAFEKTCE‑‑NQ‑TE‑EV‑KQRWVEALKCVTT‑IAGEH‑‑SCNWPDEAAMVQKLC

>V4LDJ8

GVELRYNFVSHLDKSLKRNAINAFIDTDE‑E‑MGQNL‑‑DVLLTRIEGSRIALAIFSPRYTESDWCLKELAKMKERMEQ‑RKLV‑VIPIFYKVEPATVKELKGEFGDKFRELARF‑‑‑‑ID‑KKTKKKWKEALKSVPL‑LTGFV‑LNDK‑SDEDEIIVKVV

>V4L3N2

GEDTRDNFVGLLYDELKKQ‑IRVFRDEGM‑D‑RGNEIAP‑SLVAAMEDSAASVVVLSPRYADSRWCLDELAMLCDLRSSLK‑RP‑ILPIFYKVDPSHVRKQSHHF‑EDFEEHAKRF‑‑‑TK‑EE‑‑‑‑‑‑‑‑‑‑‑‑‑‑‑‑‑‑‑‑‑‑‑‑‑‑SGEDEMIKLVV

>V4MQM0

GADTRNNFTDHLRQCLRRKSIDAFFDEKL‑R‑RGQDI‑‑SVLFERIEQSKMSIVVFSENYANSSWCLDELWKILQCREK‑SGHE‑VIPIFYKVKKSDVENQKGSFGAPFQSPMEIFE‑‑DE‑QEKIGTWRESLRTASN‑ILGFV‑YHED‑ISETKFLDDIA

>V4L7J0

GEDVRKNFISFLDPALRRANINVFIDEN‑‑EFLGAEL‑‑ANLLTRIEESEIALVIFSENYADSDWCLDELAKMKERKD‑QG‑RLIVIPIFYNLDPSVVKELRGNFGDKFRDLKRRH‑L‑HQLER‑TQKWEEALVSIPDI‑KGM‑PRAEQ‑SDDNDFINSMV

>V4MS84

GEDVREKFVSHLYEALNEEGIITFHDDRNLE‑KGDFIWE‑ALEEGINQSRFAIVVISEGYAESQWCLRELSFMVEL‑AEKKRLE‑LIPVFYEIDPSILKSRFGCFKKAFEKHE‑‑‑VRFDQ‑ET‑VSRWRSALATVGN‑ISGWD‑‑SK‑KNEDSKLVQKIV

>V4L7T2

GADLRHGFVSHLIPALKMNGVGVFVDQME‑A‑RGQEI‑‑GSLFKRIEESEIALVIFSERYTESAWCLDELVKIKETKEE‑GKLI‑AIPIFYKVEPSQVRKLMGQFGDNFWRLCRS‑‑‑‑SR‑AGHIMKWKEALESTAS‑TLGFV‑LSEG‑SSESRIVTDIV

>V4M9Q0

GADTRKNFVSFLYKELEAKGIRTFKDDKELV‑RGRLISP‑ELLQAIKESRIAVVVVSANYPGSNWCLEELRAILKL‑EAKGLLT‑VMPIFYEVEPSHVGKQIGEVAKQFKKHE‑‑‑KRQSR‑EK‑VKSWRAALARLAN‑LSGEC‑‑SKNCEDDAKLVDDFT

>V4KHQ3

GHDVRRTFLSHFLEGLKSKDIKTFKDNGI‑K‑RSESINS‑ELIRAIRESRIAVVILSKNYASSSWCLNELQMIMECRVSLG‑QT‑VMTIFYELEPSDVRKQTGDFGKAFKETCV‑‑GK‑TE‑KE‑KQRWREALTQVAV‑IAGEH‑‑SFSWPSEVDMISKII

>V4LAG4

GPDVRKTFLSHLRKQFSYNGITMFDDNGI‑E‑RGQIIAP‑SLTQAIRESRIAVVLLSKNYASSSWCLDELLEILNCKEARG‑QK‑VMTVFYGVDPSEVRKQTGDFGKAFHKTCA‑‑RK‑TK‑EE‑RRKWSEALTYVGN‑IAGEH‑‑FQNWKSEAEMIEKIA

>V4LWX4

GEDVRRSFLSHIQKEFERKGITPFNDNGI‑K‑RGKSIGP‑ELIRAIRGSKIAIILISKNYASSSWCLDELVEIMKCEEELG‑QT‑VLAIFYKVDPSDVKKLTGYFGEVFKKTCA‑‑DK‑SK‑ED‑IRRWRQALEKVAK‑IAGYH‑‑SINWDDEANMIETIA

>V4JUG9

GPDVRKTLLSHMREQFKRNGITMFDDQKI‑V‑RSATIAP‑SLTDGIRESRISIVILSKRYASSSWCLDELVEILRCKEVMG‑QI‑VMTIFYGADPSQVRKQTGEFGMVFDETCS‑‑SK‑TG‑EE‑RQKWKEALKDVGN‑IAGED‑‑FLTWDNEANMIEKIA

>V4JRJ4

GLDTRRNFISFLYRELIRRHIRTFKDDKELE‑TGRRISS‑ELIRAIEESKFAVVVISANYAASTWCLEELVKIMDF‑ANKGSLT‑VMPIFYGVDPCHVRWQIGEVAEQFKKHE‑‑‑ARQDH‑EK‑LLSWRQALTNLAS‑ISGLC‑‑SLKWEDDSKLVDKIT

>V4MBH8

GPDVRKRFLSHLHHYFACKGIKMYKDNNM‑E‑RCHTIKR‑ELRQAIKESRVLMVLLSKNYASSSWCLDELVEILKCRKDKE‑HI‑VMPIFYDVDPSHVRTQSGDFGIAFEKPAK‑‑TQ‑TE‑EL‑KQRWVEALKRVTT‑IAGEH‑‑SRNWTDEAEMVQKLC

>V4M428

GEDVRRGLLSYLLKEFREKVIDVFIDNDI‑E‑RSKPIGP‑ELKEAIKGSMIAIVLISRNYASSTWCLDELVEIMKCREEFA‑QT‑VMVVFHEVEPSDVKKQNGYFGSVFAKTCE‑‑GK‑KP‑EA‑VERWKQALQEVAK‑IAGYH‑‑SLKFDSDSDMIETIA

>V4NK95

GADVRKRFLSHLHNYFEIKGITTFKDQEI‑E‑RGNSIGP‑ELVKAIRESRVSIVLFSTNYASSSWCLDELVEILKCKEASG‑LI‑VMTIFYDVDPSSVRKQKGDFGNAFMKTCQ‑‑GK‑TE‑EV‑KQRWSKALTDVAN‑IEGEH‑‑SLNWPNEAEMIQKIA

>V4KV35

GSDVRQTFLSHVLEELRRRGITPFIDNEI‑K‑RGESIGP‑ELVRAIRESRVAIVVLSRSYASSSWCLDELVEIIKCREDRQ‑QK‑VTPVFYQVDPSDVRNQTGDFGKAFEETCK‑‑KK‑TE‑EV‑TQAWRQALKEVAN‑IAGYH‑‑SSNWSNEADLINNIA

>V4LRH4

GADLRIGLIGYLKEALIENNIKYYIDSEE‑P‑RGAPI‑‑EILFERIRESQIALVFFSIRYAESEWCLDELVEIMKNMEK‑GKLT‑VIPVFFKVEPGDVKGQKKEFGVALYGEGRR‑‑‑‑‑K‑RPRMPQWEDALEIIPT‑RMGLE‑FWEQ‑SEEVVFRNKLI

>V4KP15

SMKDTRSFVSHLSAAFHRRNISTFLGEED‑I‑ISVT‑‑‑‑‑‑‑SAIEGAKVFVVVFSENYAFSPLSLETLAKFLDLRRENG‑PV‑VIPVFYGVTPSIVEQQTEKFGKAFSEHRS‑‑‑‑‑SD‑DK‑VERWRNGLVEAAK‑LQGHD‑‑SNE‑QNDSDLVEEIV

>V4L9E0

N‑‑‑‑‑TTTSHAIE‑‑‑‑‑‑‑‑‑‑‑‑‑‑‑‑T‑RVRPIDK‑QTLEALEESKVAVVMTSETKLCSVGFLEELIVILEFQ‑ERGSLT‑VIPIFLTAFSFDVEEEIYQY‑‑‑‑‑‑‑‑‑‑‑‑‑‑‑P‑EN‑APSWRIALTKLAN‑IAADSSLSPS‑MGQSDFLEQIA

>V4MII2

GEDVRRSFLSHIQKEFERKGITPFNDNGI‑K‑RGKSIGP‑ELIRAIRGSKIAIILLSKNYASSRWCLDELVEIMKCREELG‑QT‑VMAIFYKVDPSDVKKQTGYFGEVFKKTCA‑‑GK‑SK‑ED‑IRRWRQALEKVAK‑IAGYH‑‑SNRWDNEAAMIEEIV

>V4N333

N‑DSSVPFISHLIPAIRRK‑VTSSFY‑‑‑‑‑‑‑‑‑‑‑‑‑‑‑‑‑‑‑‑‑GFTLLLVIFSRDYAYSVSCLEKLVKALESSSDEN‑SYVVVPVFYGVSRLAVKQQLATFSDAFTEHRR‑‑‑‑‑SA‑DQ‑VTKWRRALKEAAE‑FLGHE‑‑YND‑SEESEFLEKIV

>V4MFV5

GKELRKGFVSFLVPALRKENINVFIDEI‑‑EIRSIDL‑‑QHIFKRIEESSVAVVIFSELYTESKWCLNELVKINERMI‑EG‑KLKVIPIFFNVTVSDVKIHEGDFGKNFRETKRKC‑Q‑GDSDI‑IRNWEEALNSIPQK‑FGL‑ASSTY‑‑‑EYDLVHAIV

>V4LX86

GPDIRKTFLTHLRKQFNSNGITMFDDQGI‑E‑RSKFIAS‑ELIRAIIESRISIVILSKNYGSSSWCLNELVEILECKN‑‑‑‑‑I‑VMPIFFQV‑‑‑‑‑‑‑‑‑‑‑‑‑‑VHPISCA‑‑GK‑TK‑EE‑EQRWSQALTDVAN‑IAGLH‑‑LLNWENEADMIEKIA

>V4LR44

GPDVRKSFLSHLRKQFNYNGITMFDDQGI‑E‑RSQIIAP‑ALTQAIRESRISIVLLSKNYASSGWCLDELLEILNCKEDLG‑QI‑VMTVFYGVNPSDVRKQSGDFGFAFNETCS‑‑RK‑TE‑EE‑SRNWSKALTYVGN‑IAGEH‑‑SQNWDNEAEMIEKIA

>V4LS85

WEDTVRSFISHLAAAFHRKGISSFVG‑‑‑‑G‑SDES‑‑‑‑‑‑‑VAMEKSRACVVVFSGKYSSSKPCLEELVNVSERRRNNG‑LA‑VVPVFFPVTKLFVKKQIWNLG‑‑‑‑‑‑‑‑‑‑‑‑‑‑‑‑HV‑RSEWQSALLETAE‑LPGHQ‑‑LYD‑QSDSEFVQEIV

>V4LBN3

GEDVRRDFLSHIQMEFQRNGITPFIDNEI‑K‑RGESIGP‑ELIRAIRGSKIAIILLSRNYASSKWCLDELVEIMKCREELG‑QT‑VMAIFYKVDPSDVKKLTGDFGRVFRKTCS‑‑GK‑KK‑ED‑TERWRQALAKVAT‑IAGYH‑‑SNNWDNEVAMIKKIA

>V4KNX1

TTELPDDFMRHLVWGLSELGINIFIDRD‑‑EWWGRDL‑‑GHVFTCIEESTIALAIFSSCYPETEWCLDELVKMKERVN‑EN‑RLFVIPVFYNVSKNDVRNLEGEFGDRFMEMRQKY‑V‑EDPFR‑AQRWETSVTSISR‑‑‑SL‑TWEAH‑SSNIPLASNIV

>V4ME37

GPDVRVTFLSHLQMQFERNGITTFNDEGI‑E‑RSQLIGS‑ELTQAIRESRISIVVLSENYASSNWCLKELVEILKFQESAG‑QI‑VMTVFYCVDPCDVKKQMGELGKAFKKTCQ‑‑GK‑TE‑TE‑MESWIQALTHVAN‑IAGEH‑‑SLKFNKSANMIEKIA

>V4KBN9

GEDVRKSFLGHLRKQFNYNGITMFDDKGI‑E‑RSDTISP‑SLIQAIRQSRISIVILSKNYASSSWCLNELVEILECKKAMG‑LI‑VMTIFYGVDPSHVRKQTGHFGSAFNETCL‑‑RK‑TD‑EE‑RRKWSRALTDVSN‑ILGED‑‑FLNWDSEANMIEKIA

>V4KP22

GDELRKSFISHLYSRLRSEGINAFIDTDE‑G‑AGQEL‑‑ENLFKRIEESKIALAVLSSRYTESHWCLQELVKIKECSMKNNNLF‑VIPIFYKLETSTVRELTGKFGLNLWDLWRV‑GH‑NR‑DNRIVKWNEALENVLG‑KKALI‑LTET‑GKEDDFLSTIV

>V4KUY9

GVELRYNFVSHLDKSLKRNAINAFIDTDE‑E‑MGQNL‑‑DVLLTRIEGSRIALAIFSPRYTESDWCLKELAKMKERMEQ‑RKLV‑VIPIFYKVEPATVKELKGEFGDKFRELARF‑‑‑‑ID‑KKTKKKWKEALKSVPL‑LTGFV‑LNDK‑SDEDEIIVKVV

>V4LPJ0

GEDVRKTFVSHLFCELDRMGINAFRDDLDLE‑KGKSVSP‑ELVDAIRGSRFAIVVVSRNYAASSWCLDELLKIMEC‑KDTFDQT‑VVPIFYEVDPSVVRRQTESFGKDVESHS‑‑‑‑‑‑DK‑EK‑VRKWKEALTKLAA‑ISGED‑‑SRNWRDESKLIKKIV

>V4LB82

GKDLRLGFVSHLVRAFKRNKINVFMDEFE‑D‑RGKPL‑‑DSLLKRIEGSRIALAIFSESYTESNWCLKEVEKMNDCMEQ‑GNLV‑VIPIFYKVEPSTVRYLKGDFGDKLWILVKG‑‑‑‑‑D‑E‑KKKKWEEVLKSIPN‑LFGIT‑VDEK‑SDEGQAVNEIV

>V4MNA0

GDQLRYNFVSHLIDAFERHGISIFVDKY‑‑EMRGKDL‑‑KNLFVRLKESRIALAIFSTRYAESSWCMDELVKMKKFAD‑KE‑KLQVIPIFYKVRARDVRRQTGEFGDNFWKLARAS‑‑‑‑SGDE‑IKNWKE‑‑‑‑‑‑DK‑MGL‑SLKDM‑SSEADFVKEIA

>V4KPU4

GEDVRKNFLSHLLKEFENKGIVTFRDDQI‑E‑RSHSIGP‑ELVEAIRESKISLVLFSENYASSSWCLDELVEILKCKEEQR‑LK‑VMPIFYKVDPSDVRKQTGKFGMCFWETCY‑‑GK‑TE‑EK‑QRSWRQALTDAAS‑IVGDH‑‑SQDWDNEANMITKIA

>V4MFZ2

SHDVDDHFMKTILKELREREVTPLTYN‑‑‑‑‑‑‑‑‑‑‑‑‑‑ISELLDRSRVGILVLSNNYACSSESLDHLVAIMEHWKAN‑‑‑‑‑‑‑PVYFRVTLSNI‑ELEDPFEAVQLQCLN‑‑‑‑‑PA‑DR‑VQNWKEAMAEISS‑LGGNA‑‑VPQFGTQVMLAEEVV

>V4LWM1

GSDVRRGFLSHLHNDFALMGIKTFNDQKM‑E‑RGHSLER‑SLDLAIRESRVLMVLLSKNYASSLWCLNELVEILECRKNMV‑QC‑VLPVFYHVDPSDVRSQSGDFGNGFKKSCQ‑‑GK‑NE‑EE‑KQIWSKALTKVAN‑IAGLH‑‑CVNSYDDSEMIKKIA

>V4M356

RPDVRKTLLSHMREQFKRTGITMFDDQEI‑E‑RSAIIAP‑SLIEAIRESRISIVILSKKYASSSWCLDELVEILECKKA‑G‑QT‑VMTIFYGVHPSDIRKQTGEFGNTFNETCA‑‑HK‑TD‑QE‑RQKWSKALNDVGN‑IAGED‑‑FLKWDNEAIMIKKIA

>V4LVY3

GPDVRKTLLSHMREQFKRTGITLFDDQEI‑E‑RSAIIAP‑SLIEAIRESRISIVILSKKYASSSWCLDELVEILECKKA‑G‑QI‑VMTIFYGVDPSDIRKQAGEFGIAFNETYA‑‑HK‑TD‑KK‑RQKWSKALNDVGN‑IAGED‑‑FLKWDNEAIMIKKIA

>V4JVL9

GDELRKGFVGFLVEALKREKVNVFVDDH‑‑ELRGREL‑‑DHLFIRIENPKVAQTIFSERYTQSIWCLDELIKIKERMN‑QG‑NLQVIPIFYKVSSEDVKRLKGQL‑‑‑‑‑‑‑‑‑‑‑‑‑‑‑‑‑‑‑‑‑‑‑‑‑‑‑‑‑‑‑‑‑P‑GSE‑ASEIY‑‑‑‑‑‑FVKVIV

>V4L7L4

GKEVRGNFASHLKNALIREGINVFTDNNE‑R‑MGKAL‑‑DIFFTRIEESKIAIAIISSLYTESKWCLNELVKIHECVKK‑ETLE‑VFPVFYKVNVDTVKRRKEKFGENFDRLVKK‑‑‑‑EH‑TER‑KKWSRALRFVAG‑IKGEV‑VDEK‑SDEVQVINKIT

>V4KGQ2

GPDVRKTLLSHLREQFNLKGITMFNDNKI‑K‑RGEELDP‑SLKEAIRESKIWIVILSKKYASSSWCLDELVEILERKNAMK‑QI‑VMTVFYGVEPSDVRKHTGEFGIAFNETCA‑‑RK‑TD‑EE‑RQKWSKALTDVSN‑IAGED‑‑FKNWDNEANMIKKIA

>V4NGQ8

GKELRNNFVSHLRNALQRHGVNIFIDTNE‑Q‑KGKPL‑‑NVLFQRIEESRIALAIFSVKYTESKWCLNELVKMKECMDK‑GKLL‑IIPIFYKVKAYHVRFQRGRFGYVFKNL‑RN‑‑‑‑VD‑DDKKNQWSEALSSVAD‑RIGFC‑FEGM‑SDENDFINCIV

>V4L370

GEDVRVTFLSHFLKELDRKLIIPFKDNEM‑E‑RSRSLDP‑ELKQAIKDSRIAVVVISKNYASSSWCLNELLEIVNCKEEYG‑QM‑VIPVFYALDPSHVRKQTGDFGKIFEETCK‑‑NS‑TK‑EV‑TNRWRSALTDVAN‑ILGYH‑‑SVSWGNEAKMIEEIA

>V4NC85

‑‑‑VRLTMMIHVVRKLKSKGITPFIDNEI‑K‑RGQSIGP‑ELILAIRESRVAIVLLSSNYASSSWCLDELVEIIKCKEENQ‑QT‑VMTIFYDVDPSDVRKQTGDVGKAFEKTCV‑‑GK‑TK‑EV‑KQRWSQALKDVAS‑IA‑VE‑‑SSFFDNEADLINKIA

>V4M5V3

SKDTRDNFVSHLCGCLRRKRIKTYLYDEL‑P‑SEERYEE‑SL‑KAIQVSRVSVIVFSENFGDSKGLV‑‑‑‑‑‑‑‑‑‑‑‑‑‑‑RF‑VIPVLYHVDPLDIENQTGSFGDAFAK‑‑R‑‑QD‑KA‑EQ‑LQEWRDCFTEAIN‑LPGWS‑‑TNYLRDDEMLVNEIA

>V4LJ22

GEDIRKTFVSHFLTELDRKFISAFKDNKI‑K‑KGQTLDP‑VLKEAIKDSRIAIVIFTENYASSAWCLNEMLKIVKCKKKLG‑QL‑VIPVFYRLDPSHVRKQSGDFGKIFEETCRCNDK‑TE‑DE‑IKLWRTALTDVAN‑QVGFD‑‑SRNWENDAKLVEDMV

>V4NK81

GPDVRRRFLSHLHKHFESKGIMAFKDQGI‑E‑RGHTIGP‑ELVQAIRESRVSIVVLSKNYASSSWCLDELVEILKCREDQG‑QA‑VMTVFYDVDPSHVRKQSDDFGIAFKKTCQ‑‑GK‑TE‑EE‑KQRWSRALTDVAN‑IAGEH‑‑CLTWDDEAEMIQKIA

>V4LS88

RPDVRKTLLSHMREQFKRTGITMFDDQEI‑E‑RSAIIAP‑SLIEAIRESRISIVILSKKYASSSWCLDELVEILECKKA‑G‑QT‑VMTIFYG‑‑‑‑‑‑‑‑‑TGEFGNTFNETCA‑‑HK‑TD‑QE‑RQKWSKALNDVGN‑IAGED‑‑FLKWDNEAIMIKKIA

>V4L4M8

GEDVRRTFLSHLVGALDCRLVTVFKDSQI‑E‑RGHSISP‑ALVQAIRESKISIVVLSKNYASSSWCLNELLEILKCREELD‑QI‑VMTIFYDLDPSHVRNQTGDFGKAFEITCE‑‑DK‑TE‑DE‑AKQWRLALTQVAN‑IHGH‑‑‑‑‑‑LLVNVHMIEDFV

>V4KZY8

GEELRSSFLSHLIDAFERHGIDFFVDEY‑‑ELRGKEL‑‑KNLFVRIQQSRIALAIFSTRYAESSWCMDELVNMKKLAD‑KE‑KLQVIPIFYKVEAQDVREQTGVFGEHFWTLARAS‑‑‑‑SGDQ‑IKKWKEALECISDK‑MGL‑PLKDN‑SSEADFVKEIV

>V4MEX6

GEDLRLTFVPHLKHHLKESNVNVFT‑DA‑‑DAAGEPL‑‑KNLFNHIRNSRIVIVIFSISYLESKWCLDELVEIRRCLK‑SK‑KLFVIPIFFKVRASHVKEQSGDFGSKFLALQ‑‑‑‑‑KK‑‑‑R‑IMRWKRALRFVAKQ‑IGL‑AYESS‑ITELDFIKNIV

>V4LBM0

GEDVRVTFLSHFLKELDRKLIISFKDNEI‑E‑RSQSLDP‑ELKQAIRTSRIAVVVFSEKYPSSSWCLDELLEIVRCKKESG‑QL‑VIPVFYGLDPSHVRKQTGKFGEAFTKTCQ‑‑TK‑TE‑DE‑TKLWRESLIEVAN‑VLGYH‑‑SQNWHNEAKMIEAIA

>V4LSJ8

GPDVRRGFLSHLHDLFARKEITIFKDQEI‑E‑RGHTIGS‑ELVKAIREARLSIVLLSKNYASSSWCLDELVEILKCREAQG‑QI‑VMPIFYDVDPSSVRKQSGDFGIAFEKTCE‑‑GE‑TE‑EV‑KQRWVEALTCVAT‑IAGEH‑‑SRNWTDEAAMVEKLS

>M4D289

GADVRTNFLSHVLKELRSKGIDSFIDNDI‑E‑RSKLIGP‑ELVEAIRGSRIAIVLLSRNYASSTWCLNELVEIIKCREEFG‑QT‑VVPLFYELDPTDVKKQTGDFGKVLGKTCR‑‑GK‑EK‑ED‑IQRWKRALTEVAQ‑IAGFH‑‑SANGENEAELIEYIA

>M4EG65

GPDVRKTLLSHMREQFNVNGITMYNDQKM‑V‑RSEEIAP‑SLTNGIRESRIAIVILSKKYASSSWCLDELVEILECKKTMG‑QI‑VMTIFYGVEPSDVRKQTGEFGIAFEDTCE‑‑HK‑TK‑EA‑KQKWIKALTDVSN‑IAGED‑‑FLRWANEADMIKQIA

>M4DA90

NQVSEEYLISYIFNELCARGFAPLRYD‑‑‑‑‑‑‑‑‑‑‑‑‑‑MKQKLLHSRVGIIIFSMNFAHSRECLDGFVAVMDHLKANE‑LV‑LIPVFFKVSVSDVRGQSGSFGKAFTRLGD‑‑‑‑‑SA‑SQ‑VLKWRAAMIKLAS‑IIGYA‑‑YKK‑GDEVILAKNIV

>M4F6T0

GDELRYNFVSHLTSALLRDGVNIFIDTNE‑E‑KGKSL‑‑NVLFERIEESRIALALFSVRYTESKWCLNELLKMKECMDK‑GQLL‑IIPIFYKVQAYEVRFQRGRFGYLFNKL‑RH‑‑‑‑VD‑VDKKKQWSEALNSVAD‑RIGFC‑FDGK‑SDENKFIHSIV

>M4EG76

GPDVRKTFLSHLRKQFNNNGISMFDDQGI‑E‑RGQTIAP‑ELIRAIRESRISIVVLSKNYASSSWCLDELVEIFKCKEDKN‑QI‑VMTVFYGVDPSDVRKQTGDFGKAFKKTCA‑‑R‑‑‑‑‑‑‑‑‑‑‑‑‑‑‑‑‑‑‑‑‑‑IRGEA‑‑‑‑‑‑DKESEMIEKIA

>M4EFM4

GEDVRKHFISFLDPALRRANINVFIDEN‑‑ELLGADL‑‑ANLFTRIEESEIALVIFSKNYADSDWCLDELAKMKERKD‑QG‑RLRVIPIFYNLSPSVVKELRQDFGDKFRDMQRRH‑K‑HQPER‑TKKWEEALVSVPDI‑KGM‑PLSEQ‑SDDNEFINSLV

>M4DZK9

GEELRENFVSHLYKALRQSGINAFIDSDM‑V‑LGDKL‑‑ITLFKTIKESKIALAILSSKYTASQWCLEELVKIMECSTN‑KNLV‑VIPIFYKVSTSIVDKLEGEFGVNLLNVWRRQ‑‑‑AR‑NSRIVKWNAALQDMLS‑RAALI‑YDGS‑MEENAFVARIV

>M4ECP6

GQELRYRFVSHLVAAFERDEINFFIDKN‑‑ELRGTDL‑‑KNIFVRIQESRIALVIFSNRYAESSWCMNELAKIKELAD‑KE‑KLHVVPIFYKLKVGDVRGQTGKFGTKFWNLARVS‑‑‑‑TGDQ‑IKTWKEALECISDK‑MGLFAFGDD‑AQSAGLLSAVA

>M4CPF6

GEDVRRDFFSHIQREFERKGITPFIDNEI‑K‑RGESIGP‑ELIRAIRGSKIAIILLSRNYASSKWCLDELVEIMKCREEFG‑QT‑VMAIFHKVDPSDVKKLTGDFGKFFKKTCA‑‑GK‑AK‑DC‑IERWRQALAKVAT‑IAGYH‑‑SSNWDNEADMIKKIA

>M4EG77

GPDVRKTFLSHLRKEFNNNGISMFDDQGI‑E‑RGQTIAP‑ELRRAIRESRISIVVLSEYYASSSWCLDELVEILKCKKDKN‑QI‑VMTIFYGVDPSDVRKQTGDFGKVFKKTCA‑‑RK‑TE‑EE‑RRKWSQALNRAGK‑IAGEH‑‑FLNWHNESEMIEKIA

>M4DWG7

GEDVRIGFLSHIQKEFKRKGITPFIDNEI‑R‑RGESIGP‑ELIRAIRGSKIAIILLSRNYASSKWCLDELVEIMKCKEELG‑QT‑VIPVFYKVDPSDVKKLRGYFGKVFEKTCE‑‑GK‑SK‑ED‑TEKWRHALEKVAT‑IAGYD‑‑SRTWDNEAAMIEEIA

>M4DHP9

GPDTRRNFISFLYKELVQRNIRTFKDDKELE‑SGQRISP‑ELDRAIEESKFAVVVVSANYAASTWCLEELVKIMDV‑ENKGSLT‑VIPVFHGVDPCHVRRQIGQVAVQLEKHE‑‑‑MREDR‑EK‑VLSWRQALTNLAS‑ISGVC‑‑TLKWEDDSMMVDEIA

>M4CV10

‑EEIRSGFLSHLRHALARQSITCFIDQEEER‑‑‑KQLSA‑VLH‑AIPESKVAVVVLSKNYASSSWSLNELAEIMETN‑‑‑L‑‑M‑MVPVYYEVDISDVRYQKGSFGEDLRRHGQSESQ‑T‑‑‑‑‑MKRWKACLTKLTD‑TKPEF‑‑SPQ‑KDEAKLVRDIT

>D1GEG9

GKDVRQTFLSHLIVALDRKLVTVFKDSQI‑E‑RGHSISP‑ALVQAIRDSRVSIVVLSKNYASSSWCLDELLEILKCREELG‑QI‑VMTIFYDLDPSDVRYQIGEFGKAFEKTCE‑‑KK‑TA‑DV‑TKQWGLALTEVAN‑IHGHH‑‑SRKWDSEAHMVDDFV

>M4CIG3

GADVRKKILSHVLKEFKRRGIDTFIDNNI‑E‑RSKSIGP‑KLIEAIRGSRVAIVLLSKNYASSTWCLNELVEITKCRREFG‑QT‑VMPVFYEVDPSDVKKQSGEFGKVFQDICN‑‑GK‑KE‑ED‑TRTWREALVEVAT‑IAGEH‑‑SSNWCSEAEMIEKIA

>M4DZ07

GPDVRKTLLSHVRKQLSCNGISMFDDQWI‑E‑RSQTIAP‑ALTQAIRESRISIVVLSKKYASSSWCLDELVEILKCKEKMG‑QI‑VMTVFYGVDPSHVRNQTRDFGIAFDETCQ‑‑GK‑TE‑EK‑MRIWRQALTNVGN‑IAGEH‑‑FLNWDNESMMIEKIA

>M4FGI2

GPDVRRGCLSYLLKEFKEKAIDVFIDNDI‑E‑RSKLIGP‑ELTEAIRGSLIAIVLISRNYASSTWCLNELVEIMRCWDEDK‑QT‑VEVIFYEVDPSDVKKQKGDFGAVFDKTCA‑‑EK‑ST‑EE‑VERWRKALHIVAQ‑LAGYH‑‑TSNFDDDAVMTAKVV

>M4E9N7

‑‑DFSAIYAISLQ‑‑‑‑‑‑‑‑‑‑‑KDHEI‑E‑RGHTIGP‑ELVQAIRESRVSVILLSKNYASSSWCLDELVEILNCKKASG‑QI‑VMTIFYQVDPSDVRKQTGDFGIAFKKTCE‑‑KK‑TE‑ED‑KKRWMEALAYVAN‑IAGEH‑‑SLNWTDEAAMVEKFA

>M4EFU7

GPDVRKTFLSHLRKQFACNGISMFNDQAI‑E‑RSHTIAP‑ALTQAIRESRISIVVLTKNYASSSWCLDELLEILKCKEEMG‑QI‑VMTIFYGVDPSHVRKQTGDFGKVLKKTCS‑‑GK‑TE‑EE‑KQRWSQALTDVGN‑IAGEH‑‑FLNWDKESEMIEKIA

>M4E4E3

GQDVRRSFLSHFLEGLKTNGVNTFVDDGI‑M‑MSGSINS‑ELVRAIRESRIAVVILSKNYASSSWCLHELQLIMDCRASLG‑QT‑VMTIFYDVEPSDVRKQTGDFGKAFEETCN‑‑GS‑TE‑EE‑KKTWRQALTQVAL‑IAGEH‑‑VTSWASEAQMISKIV

>M4CQG3

GEDVRKGFLSHVVKEFKSKGIEAFIDNEM‑E‑RGKSVGP‑TLEKAIRQSRVAIVLLSRNYASSSWCLDELVEIMKCREEDK‑QR‑VITVFYEVDPSDVRKQIGDFGKAFDDTCV‑‑GR‑TE‑EV‑THVWRQALKEVAD‑IAGYA‑‑SSNCGSEADLINELA

>M4CAD3

GEDVRKGFLSHVLKEFKSKGINVFIDNEI‑K‑RGESVGP‑ELVKAIRHSRVGVVLLSRNYASSSWCLDELVEIMKCREEVG‑QT‑VMTIFYNVDPSEVRKQTGDFGKAFDETCV‑‑GR‑TE‑EV‑KRAWRQALNDVAS‑IAGYD‑‑ASNCDNEADLINKVA

>M4EFN2

GDELRKNFISHLVEALQRSEINFFTDKQ‑‑EKKGEDL‑‑SNLFNRIEEAKIALAVFSKRYTESRWCLDELVKIKERAD‑LG‑KLKVVPIFYNVTTDNVKYLTEEFGSNLGRHQSPH‑‑‑‑EQNK‑IGKWKEALACISCK‑LGF‑PFIDN‑SSESEFIDSIV

>M4F5Q7

GADVRKSFLSHLVKEFGSKGINLFIDNEI‑T‑RGEFIGP‑ELKKTIQGSRIAIVLLSKRYASSSWCLDELVEIMKCKEELG‑QT‑VVPVFYEVDPSDVKKQAGEFGKVFKKTCK‑‑GK‑TN‑EV‑IRKWSKALAKVAT‑LAGYH‑‑SKNWDNEAKMIEDVA

>M4CV11

GEDVRKSFISHLIRELNQKGIRSFKDEEEVK‑RGKQISA‑‑LHRAISESYVALVVLSKNYSSSVGCLDELDKIMDRAQKLT‑‑V‑VVPVFYEVDLSDVKYQRGGFREDFERHGQVEPK‑T‑‑‑‑‑LKR‑‑‑‑‑‑‑‑‑‑‑‑‑‑‑‑‑‑‑‑‑‑KDEAKLVRDIT

>M4DMS2

GADVRKAFLSHVLKEFRRKGIDPFIDNDM‑E‑RSKSIGS‑ELIEAIRGSRIALVLLSKNYASSTWCLNELVEIIKCREELD‑HT‑VMVLFYEVDPADVKTQTGDFGKVFRKTCK‑‑GK‑TK‑EE‑IGRWKHALAEVAK‑ITGYH‑‑SRNWDKEADMIEKIA

>M4EQC6

G‑‑‑‑‑KKALDMDYDLSRNGIKSFKSESWKK‑SFKPIDR‑QTLEALTESKVAVVMTSDEEASSVGFLEELLVILEFQ‑EKRSLT‑VIPIFLTKRPLDM‑EEVSHF‑‑‑‑‑‑‑‑‑‑‑‑‑‑‑P‑ER‑DRTWRTVIAKLEN‑ISAQY‑LSRN‑IHGTHRIKQVA

>D1GEF8

GEDVRRTFLSHLLKEFRRKGIRTFIDNDI‑K‑RSQLIGP‑ELVQAIRESRFAVVVLSKRYASSRWCLNELVEIKE‑‑‑‑SS‑KN‑VMPVFYEVNPSDVRNLSGEFGTAFEEACQ‑‑GK‑P‑‑ED‑VQRWRQALVYVAN‑IAGES‑‑SQNWDNEADMIEKIA

>D1GED5

GEDVRKDFLSHIQMEFQRKGITPFIDNEI‑K‑RRDDIGP‑ELIRAIRGSKIAIILLSRNYASSKWCLDELVEIMKCREELG‑QT‑VMAIFYRVDPSDVKKLAGDFGRVFKKTCA‑‑GR‑TK‑EN‑IERWRQALAKVAT‑IAGYH‑‑SSNWDNEAAMIKKIA

>M4EI84

GPDIRRSFLSHLHKHFESKGITMFKDHEI‑E‑RGHTIGP‑ELVQAIRESRVLMVVLSKKYASSSWCLDELVEILKRKEDQG‑KI‑VMTIFYKVDPSCVRKQNGDFGSIFEKTCE‑‑GK‑TK‑EL‑KLRWTKALTDVAN‑IEGDY‑‑SLNWHDEAEMIGKIA

>M4E4G8

GVDVRKGFLSHVLKELKSKGILPFIDNEI‑K‑RGESVGP‑VLVGAIRQSRVAVVLLSRNYAYSSWCLDELVEIMKCRKEDQ‑QK‑VMTIFYEVDPSHVRKQNGDFGKAFDETCV‑‑GK‑TE‑EV‑KQAWKQALKEVAG‑IAGYD‑‑FSNCDNEADLINKVA

>M4D251

CANTLQSFASHLSMGFHRKGIYASAN‑‑‑‑‑‑‑‑‑‑‑‑‑‑‑‑‑DVMEGASASVVVFSKNYLSSPSCLDKLVRVLQCRRKSG‑QL‑VVPVFYDVSPSNVEVQEQESV‑‑‑‑‑‑‑‑‑‑‑‑‑‑‑‑DR‑I‑‑‑‑SALQELRE‑FTGYQ‑‑FRE‑CSECELVEEIV

>M4FCQ3

GADVRKSFLSHFLKELGSKGINLFTEKEI‑P‑RGEFIGP‑ELKKAIQGSRIAIVLLSKRYASSSWCLDELVEIMKCKEELG‑QT‑VMPIFYEVDPSDVKKQAGEFWKVFKETCK‑‑GK‑TN‑EV‑IGKWSKALAKVAT‑LAGYH‑‑SNNWDNDVKMIEDVA

>M4DZU2

GLDVRKTFLSHFLKELDLRLITAFKDSKI‑E‑RSQAIEP‑ELLQAIRSSRIAVVMFSKNYASSKWCLDELLEIVKCKQELE‑QI‑VIPVFYGLDPSDIRKQLGEFGEAFDKTCK‑‑NR‑TE‑SK‑IQLWRQALTDVAN‑LEGHH‑‑SRNWDNEAKMIEAIV

>D1GEE4

GEDVRKNFLSHFLKELDRKLIKAFKDNEI‑E‑RSHSIAP‑ALVTAIRTSRIAVVVFSPKYASSSWCLDELVEIVRCMEELG‑QL‑VLPIFYGLDPSHVRKQTGKFGEGFAKTCK‑‑MK‑TK‑AV‑KIRWQQALTVVAN‑LLGYH‑‑SQNFNNEAKMIEVIV

>M4D878

GEDVRKSFLSHLLKELHRKSINTFIDHGI‑E‑RSRPIGP‑ELLSAIRESRISIVVFSKNYASSSWCLNELVEIYKSFKELN‑QM‑VIPVFYGLDPSHVRKQTGEFGEAFMVSCQ‑‑GK‑TD‑DE‑KQWWIQALAEVAN‑MAGED‑‑SRNWSDESNMIERIA

>M4EFN3

HHDAGELFIDEIIIELQKRAITSLRYD‑‑‑‑‑‑‑‑‑‑‑‑‑‑LVPGLHTYGVFLLFISKNYT‑SGESLDKLVTLTEYQKANG‑LL‑LIPIFYKVTPSEF‑‑PKFFTEERLLQLDD‑‑‑‑‑SI‑RR‑VQKWKEAMNELAL‑SDDCK‑‑WIF‑GNDSILPEEIV

>M4D290

GADVRKTFLSHVLKEFRSKGIDLFIDKDI‑E‑RSKSIGP‑ALIEAIRGSRIAIVLLSENYASSTWCLNELVEIIKCRQEFG‑QT‑VMPIFYQVNPTDIKKQKGYFGKVFRKTCK‑‑GK‑RK‑EE‑IQRWKHALTQVAQ‑IEGLM‑IPLSRETEAEMIDDIA

>M4CV15

GADTRRKFVSFLYNDLEAKEIRTFKDDKELE‑SGRPIPP‑ELIQAIKGSKIAVVVVSATYPASYWCLEELVKILKY‑ERKGLLK‑VLPVFYEVDPSHLRWQIGEVAKQFKKHE‑‑‑KRQSK‑ER‑VKSWRDALAYLAN‑LSGEC‑‑SKKWDDDSKLVDGIT

>M4DZK5

SCDGTRTFVSHLSAALKRVNITVMEEDDK‑N‑KVETR‑‑QYLPLGIERSKICVVVLSEDFASSKHSLTTLAEIIEWRSKTG‑AT‑VVPVFYGVDRSLVEQQIGKYGEAFSKHEA‑‑‑‑‑SK‑DR‑VTEWRNALTEAAS‑IEGLH‑‑SNA‑SSDLKLMEDIV

>M4CPK7

GEDVRRKFFSHIQMELERKGITPFIDNEI‑R‑RGESIGP‑ELIRAIRGSKIAIILISRNYASSKWCLDELVEIMKCREELG‑QT‑VMPVFYEVDPSNVKKLTGDFGKVFRKTCA‑‑GK‑TK‑EC‑IKRWRQAFAKVAT‑IAGYH‑‑SSNWDNEADMIKKIT

>M4DZU0

GEDVRKSFLSHFLKELDRKLISAFKDKKI‑E‑TSESLDP‑VLKQAIKKSRIAIVIFSQNYVSSSWCLNELLEIVKCQQELS‑QI‑VIPVFYDVDPCHVRHQTKEFGEVFKKTCL‑‑RR‑TD‑DE‑IKLWKKALTDVAN‑LVGYH‑‑SQNWENEATMIEVIA

>M4ETB5

GPDVRKTFLSHVRKQFNNSGIMMFDDQGI‑E‑RSQTLAP‑SLTQAIINSRISIVVLSKNYASSSWCLDELVKILECKRVNG‑QT‑VMTIFYGVDPSDVRKQAGNFGRAFNDTCV‑‑GK‑TD‑EE‑RQRWTQALTDVSN‑ILGEH‑‑FLNWDNESNLIEKVT

>D1GEI4

NQDSEEYFISYISKELCLRGFTPLIYD‑‑‑‑‑‑‑‑‑‑‑‑‑‑LTEMLHRSRVGIIIFSNNYASSRQCLDKFVAILDYSKANN‑FV‑LLPVFFKVKVSDIRGQSGSFRRAFSRLEH‑‑‑‑‑SS‑SQ‑V‑‑‑‑‑‑‑‑‑LTA‑INKYQ‑‑YMK‑GEDVILAKSIV

>M4E4C8

GEDVRKTFVSHLFCELDRMGINAFRDDLDLE‑RGKHISS‑ELVDTIRGSRFAVVVVSRNYASSSWCLDELLEIMER‑KNTVDQT‑IIPVFYEVDPSDVRRQTGSFGEGVESHS‑‑‑‑‑‑DK‑KK‑VMKWREALTQLAA‑ISGED‑‑SRNWRDESKLIKKIV

>M4EG59

GPDVRKTFLSHLRKQFTFNGITMFDDQGI‑E‑RGQVIAP‑AITQAIRQSRISIIVLSKNYASSSWCLDELLEILKCKEDMG‑QI‑VMTVFYGVDPSHVRKQTGDFGKAFKETCA‑‑RK‑TK‑EK‑EERWSQALEYVGN‑IEGEH‑‑FLNWVNEADMIEKIA

>M4DVZ9

GEDVRNSFLSHLME‑LERNLITTFIDHGI‑D‑RSRPIGS‑ELLLAIKESRIAIVIFSKNYASSTWCLNELVEIHKCFKDLN‑QM‑VIPIFYHVDPSDVRKQTGEFGDRFKEICM‑‑DK‑TE‑DE‑IERLVRALTDVAN‑LAGQD‑‑SKNWEGEAKMIEHIA

>M4DM13

GEDVRKTFLSHFMKELNRKLITAFKDNEI‑E‑RSRSLDP‑ELRQAIKDSRIAVVIFSTNYASSSWCLNELLEIVRCKEECA‑QM‑VIPVFYGLDPSHVRKQTGDFGKIFDKTCQ‑‑NK‑TE‑DE‑IILWREALTDVAN‑ILGYH‑‑SVTWDNEARMIDEIA

>M4EW13

GEDVRKDFLSHIQKGFERKGIRQFNDNEM‑E‑RGESISF‑QLVRAIRGSKIAVVLFSKNYASSKWCLDELVEIMKCRREFG‑QI‑VIAVFYKVDPSDIRKQTGDFGKVFRKTCA‑‑GK‑TN‑EE‑IRRWRVALAEVAA‑IAGYH‑‑SSNWDNEADMVENIA

>D1GEJ1

GADTRNNFTGHLQDKLLGKGIDSFIDDRL‑R‑RGDDI‑‑TALFDRIEQSKIAIVVFSENYANSVWCLRELVKILQCRDR‑NQQL‑VIPILYKIDKSKLK‑‑‑‑‑‑‑‑‑‑NVRKTRFG‑‑VT‑EDEIVSWEAAISTAVD‑ISGYV‑VDRS‑TSEAKLVNDIA

>M4CD72

GGDVRKGFLSHLLKELESKGISPFIDNNI‑E‑RGQSIAP‑ELVQAIKESRVALVLLSPNYASSRWCLDELVEIMKCREREQ‑QT‑VITIFYGVDPSDVRNQTGDFGKVFNKTCD‑‑GR‑TE‑GV‑KEAWKKALVDVGN‑IAGYD‑‑SSRWDNEAKMIEEIA

>M4DZK6

GADVRKNFLSFLTDGLKRACVNYYVDTKE‑T‑KGEVL‑‑DILLQRIQESRLVLIILSENYMQSNWCIKELRTTTKDIKE‑SRRK‑VIPIFYNVQVADVK‑‑‑‑‑‑‑‑‑‑‑‑‑‑‑‑‑‑‑‑‑‑‑‑‑‑‑DKWKEALMILTR‑HMGMR‑SDEY‑GTDCEFIEHIV

>M4EFM2

KEELGDNFVRHLAWALRELGINVLMDSY‑‑NRRGDEQ‑‑QQVFTNIEKSNIVLAIFSKRYSESDRCLNELVKMEELTK‑EG‑KLVVIPVFYNVKTNEVRRLQGEFGIHFADSVKRF‑S‑MEPMM‑VQSWEEALNFIIK‑‑‑GL‑SLERH‑RNEFALVAAIV

>M4DNF0

GTDVRKKFISHLNDALTEEGIITFHDDRDLE‑RGNPILK‑GLEEAMNQSRFAIVVVSEDYATSQWCLRELAFMVEL‑AEKKRFD‑LIPIFYEIDPSALKSRTGCFNKAFEDHE‑‑‑KRFDA‑ET‑VRKWRRAVDIVAN‑ISGWD‑‑SKTRSDDSKLIQEVV

>M4EFL8

ATELPDDFIKHLVSGLTDLGINIFMDRD‑‑DWWGRDL‑‑DRIFTCIEESTIALVIFSPSYPETEWCLDELVKMKERAN‑NN‑KLLVIPIFFNVSKNDVRNFEGEFGDRFMELRKRY‑T‑KDPFR‑VQRWETSVMTISR‑‑‑SL‑TWETQ‑SSSISIAMDII

>M4EQC4

GIDTRRTFVSHLLKQF‑‑‑‑‑KTFRQ‑‑‑‑E‑‑‑EEMQPTQVLEAIENSKIAIVVISKNYTASVSCLDELAKIVECEEK‑‑‑QLVMIPVLHEVDPSDVLEQAGDI‑‑‑‑‑‑‑‑‑‑‑NN‑‑N‑ET‑VERWRKALASIKTQL‑‑‑‑‑YSH‑WDGDSSMLEKMA

>M4F5S6

GLDVRQTFLSHLLKEFERKGINTFKDSQI‑K‑RGKYISP‑ELKQAIRESRICLIILSKNYASSSWTLGELVEILESRKASG‑KT‑VMTVFYDIDPSHVRKQSGEFGMAFRKTCE‑‑RK‑TE‑HQ‑KQRWKQALTNVAS‑ILGED‑‑SHKWDNEADMISKIA

>M4F4L9

GGDEAKMLERHLQSCFEKYGIRTFFDRTLLE‑IGAVVGPVDLIQSLRDSKPIIVVTEKDYNCSNWCLDELVEILKCKEASE‑QN‑VMTIFYDIDPSSVRKQKGDFGSAFKKTCV‑‑GK‑TE‑EV‑KQRWARALTHVAN‑IKGEH‑‑SLNWASEAEMIQKIA

>M4DHQ1

GLDTRRNFISFLYKELVRRNIRTFKDDKELE‑SGRKISP‑ELERAIQESKFAVVVISANYAASTWCLQELVKIMDF‑VNKGSLT‑VIPVFHGVEPCHVRWQMEKFAVQFEEH‑‑‑‑KSEDR‑EK‑VLSWGHALTNLAN‑ISGHC‑‑SSEWEDDSMMVDEIA

>M4EE36

GPDVRKGILSHLHIVFERKKITMFKDQEM‑E‑RCQQIGS‑KLIQAIREAKASLVLISKNYASSRCCLDELLEILKCKESSG‑QI‑VMPIFYDVDPSDVRKQKGDFGITFKTTCQ‑‑GA‑TE‑EK‑KQRWIEALTCVAT‑ITGED‑‑SRTWANDAAMLEKIS

>M4EB03

GKDTRRIFISFLYKELIRMSIRTFKDDVELK‑SGRRISS‑DLLLAIEGSKIAVVVVSKKYPASPWCLHELVKIMDV‑EKQGSLT‑VMPIFYNVEPSHVRRQIEKVAEQFTKHE‑‑‑GRENH‑ET‑VVSWRQALTNLAS‑ISGHC‑‑SRDCDDDSKLVDEII

>M4EQC7

GEDTRKTIVSHLYAALDSRGIVTFKDDQRLE‑KGDHISD‑QLHIALKGSSFAVVVLSENYATSRWCLMELQLIMEY‑MKEGTLE‑VFPVFYGVDPSTVRHQLGSF‑‑SLERYKG‑‑RPE‑V‑HK‑VHKWREALHLIAN‑LSGLD‑‑SRHCVDEAVMVGEIA

>M4E4E5

GQDVRRTFLSHFLEALKSKGIKTFIDNGI‑I‑RSESINS‑ELIRAIRESRIAVVILSKNYASSSWCLNELQLIMECTVSLG‑QT‑VMTVFYDVEPSDVRKQTGDFGKAFKETCY‑‑RK‑TE‑EE‑KKKWSEALSQVAV‑IAGEH‑‑SVSWAGEAEMISKIV

>M4DSB6

GEDVRNNFLSHIQKEFKRKGITYFNDNGI‑K‑RGESIAP‑ELIRGIRGSKIVIVLLSRNYGSSKWCLEELVEIMKCREELK‑QT‑VMAIFCKVDPSDVKKLTGDFGKVFRKTCE‑‑GQ‑TK‑ED‑IWRWKQVWRR‑‑‑‑‑‑‑‑‑‑‑‑‑‑‑DDEASMVEEIA

>M4EG66

GPDVRKTLLSHIREQFTRSGITMFDDQEI‑V‑RSATIAP‑SLTEAIRESRISIVILSKNYASSSWCLNEMVEILECKKAMG‑HI‑VMTIFYGVDPSDVRKQTGEFGIAFNETCA‑‑SK‑TK‑EE‑KQRWRQALNEVGN‑IAGED‑‑FLRWSNEAKVIKKIA

>M4EFN6

CVDEVRSFVSHLSDALRRNVISSVFV‑‑‑‑D‑SGLL‑‑‑‑‑‑‑GKVERAKVSVVVLP‑‑‑ANRQVCLEKLEKVLNCQRNKE‑QV‑MIPVLYGDSKLHG‑‑‑‑‑‑‑‑‑‑‑‑‑‑‑‑‑‑‑‑‑‑‑‑‑‑‑‑‑EWLSAM‑NLRG‑LPVFQ‑‑SRN‑CSDSKLVEKIV

>M4CJ08

GEDVRKTFLSHFLRELERNSIVAFKDNEM‑E‑RSQSIAP‑ELVQAIRDSRIAVVVFSKNYASSSWCLNELLEILQCNEEFG‑QL‑VIPIFYGLDPSHLRKQTGDFGEAFKKTCL‑‑NQ‑TH‑EV‑EDQWKQALTNVAN‑ILGYH‑‑SKNCDSEAAMIEEIS

>M4E3J8

GPDVRNGFLSHLYQSLVTSGIYTFKDEEL‑E‑KGESISP‑ELRKAIENSKIHLVVLSESYASSSWCLDELVHMMRRLKNPG‑HL‑VFPVFYKIEPSHVRRQSGPFGESFHKHRSRHSK‑‑‑‑‑‑‑LKQWRKALTSIAN‑LKGYH‑‑SSNGDNDAELVDQLT

>M4CAD2

GPDVRKGFLSHVRKELKSKGLIVFFDDEI‑K‑RGESIDQ‑ELVEAIRQSRTAIVLLSPNYTSSSWCLNELVEIIKCREEDR‑QT‑VLTIFYEVDPSDVRKQTGVFGKLFKKTCV‑‑GK‑TE‑KV‑KKAWKQALEDVAG‑IAGYH‑‑SSNCANEADLIKKVA

>M4EI85

GPDVRRKFLSHLHYHFASKGITVFKDQEI‑V‑RGQTIGP‑ELKQAIRESRISMVVLSKNYASSSWCLDELVEILECEEACG‑QK‑VMTIFYDVDPSDVRQQSGDFGRAFDRTCK‑‑RQ‑TE‑EV‑KQIWSKALTDVAE‑IAGVH‑‑SLSWDDEAKMMQKIV

>M4CI30

GADTRKNFVSFLYKQLETKGIRAFKDDNALV‑CGRSIAP‑VIVQGIKGSTIAVVVISPTYPASFWCLEELVMILKL‑EREKLLT‑VVPIFYEVEPNDLKRQTGKLVKQFKKHE‑‑‑KRHST‑ER‑VHSWRDALNRLAT‑LSGDC‑‑SKISEDDATLVDRVT

>M4EKW4

GFDTRNNFTGHLQKALRLRGIDSFIDDKL‑R‑RGDDL‑‑TALFDRIEHSKIAIIVFSKNYSNSAWCLRELVKILECRDR‑NQQL‑VIPILYKVDKSELK‑‑‑‑‑‑‑‑‑‑NVPKKSFE‑‑VK‑EEETSTWEAALTTAFN‑ISGYV‑VNES‑TSEAKLVDEIA

>M4D1Z6

GVDVLENFLSHIVKEFKSNAIDLFIDNNI‑E‑RSKSIGP‑ALIEAIRGSRVAIVLLSKNYASSTWCLNELVEIMECREEVG‑QT‑VITIFYQVNPTDIKKQKSYFGKVFRKTCK‑‑GK‑RN‑EE‑IQRWKHALTDVAQ‑IEGYH‑‑SINWKNESEMIEYIA

>M4DII4

GEETRHNITKRIYDALVKEKFRVFRDDGL‑EGGGDETSP‑NIVEAMKDSAASVVV‑‑‑‑‑‑‑‑‑‑‑‑‑‑‑‑‑‑‑‑‑‑‑‑‑‑‑‑‑‑‑‑‑‑‑‑‑‑‑‑‑‑‑‑‑‑‑‑‑‑‑‑SYALHVRNV‑‑‑SE‑‑‑‑‑‑‑‑‑‑‑‑‑‑‑‑‑‑‑‑‑‑‑‑TPE‑‑SVDEDMIELVV

>M4EFE8

GEDVRKDFLSHIHMEFQRKGITPFVDNEI‑K‑RGESIGP‑ELVRAIRGSKIAIILISRNYASSKWCLDELVEIMKCREELG‑QT‑VMAIFYRVDPSDVKKLAGDFGRVFRKTCA‑‑GK‑TK‑DN‑IGRWRQALAKVAT‑VAGYH‑‑SSNWDNEAAMIKKIA

>M4D879

PEDVYKFFFCRRLSALF‑KDVNRYL‑‑‑‑‑‑‑‑‑‑‑‑‑‑‑‑‑‑‑PIRKA‑‑TNIVFSKSYASSSWCLNELVEIHKCYMEVD‑QT‑VIPIFYGVDPSDVRKQTGEFGKAFGETSK‑‑GT‑TE‑DE‑KQRWMRALAEVAN‑MAGED‑‑LQNWCNEANLIDKIA

>M4EZ36

GEDVRKTFLSHLLLSLDRKLITCFKDNEI‑E‑RSQSIGL‑KLVHAIRDSRIAIVILSKTYASSTWCLNELLEIVKCKEDKG‑QI‑VIPVFYGLDPSHVRKQTGEFGETFQMICK‑‑NR‑SD‑EL‑PDLWKGALTHVAN‑IHGYH‑‑SDNWNNEAHLIEDIT

>M4D288

GEDVRTNFLSHVLKELKSKAIDLFIDNDI‑E‑RSKSIGP‑ELIEAIRGSRIAIIFLSKNYASSTWCLNELVEIMTCREEFG‑QT‑VISLFYEVDPTHVKKQTGDFGKVFKKTCV‑‑GK‑TK‑DE‑IQRWKHALTEVAQ‑IAGFH‑‑SSNWETEAKMIEVIA

>M4F6T1

CEDKVRSFISHLSAAFHRRGISSYIG‑‑‑‑G‑SDKS‑‑‑‑‑‑‑GDMEKSKACVVVFSEKYSSSKPCLEELVKVSERRYEGG‑HA‑VVPVFYRATKSSVKKLIWKSS‑‑‑‑‑‑‑‑‑‑‑‑‑‑‑‑DL‑TSERRSALLEVVD‑LPGHE‑‑SYV‑QSESDLVEEIV

>M4FEG2

GADVRKSFLSHILKEFRSKGIDTFIDDDI‑E‑RNKSIGP‑QLIDAIKGSKIGIILLSKNYASSSWCLNELVEIMKCRTELG‑QT‑VMTIFYEVDPADVKKQRKDFGKSFRKTCK‑‑GK‑TS‑DE‑IETWKKALEGVAT‑IAGYH‑‑SNNWDNEAAMIEKIA

>M4F6T9

GEDVRTSLLTHILKEFKSKAIYPFIDDKM‑K‑RGKIIGP‑ELKKAIQGSRIAVVLLSKNYASSSWCLDELAEIMKCQEELD‑QM‑VIPILYEVNPSDVKKQRGDFGKVFKKTCE‑‑GK‑TN‑EV‑IEKWSQALSKVAT‑ITGYH‑‑SINWNDDAKMIEDIT

>M4D252

GKQLRYGFVSHLEKALRRDGINVFVDKNE‑T‑KGKDL‑‑SSLFSRIEESRIALAIFSSMYTESKWCLNELEKIKECVDL‑GKLV‑VIPIFYKVDTDDVKNLNGVFGDKFWELAKT‑‑‑‑CN‑GEKFEKWRQALQNIPQ‑KLGFT‑LGET‑SDEGDYINQIV

>M4E8F1

VKDTRDNFVSHLCGCLRRKRIKTYLFDEL‑P‑YEERYEE‑SL‑KAIEVSRVSVIVFSENFGDSKFCLDEVVAILKCKKRFG‑QI‑VIPVLYHVDRVDIENQTGSFGEAFAK‑‑R‑‑QD‑KA‑DQ‑IKEWKDGFTEAIN‑LPGWS‑‑TSHLRDEEMLVNGIA

>M4D5A9

GKQLRNGFVSHLEKALRKDGINVFIDKNE‑T‑KGKDL‑‑SILFSRIEESRIALAIFSTLYTESNWCLNELEKIKECVDL‑GKLV‑VIPIFYKVETDDVKNLKGVFGDKFWELAKT‑‑‑‑CR‑GEKLDKWKEALEDVPK‑KLGFT‑LSEM‑SDEGEYISKIV

>M4E4E4

GEDVRRNFLSHFHKELQLNGIDAFKDGGI‑K‑RSRSIWP‑ELKQAIWESRVSIVVLSKNYGGSSWCLDELVEIMECKEVSG‑QT‑VMPIFYGVDPTDVRKQSGDFGKSFDTICH‑‑VR‑TE‑EE‑RQRWKQALTSVAS‑IAGDC‑‑SSKWDNDAVMIERIV

>D1GEH3

GKDTRADFAERLYTEIKRE‑VKIFRDEGM‑E‑RGEEINA‑SLIAGMEDSAASLVLFSPHYADSRWCLDELATLCDLSSSLD‑RP‑MIPIFYKVDPSHVRKQSGDF‑KHFEAHAERF‑‑‑SK‑ER‑IQPWREAMKLVGH‑LPGFI‑YRE‑‑ENEDALIRLVV

>M4EG67

GPDVRSSYLSHLRKQFERNGIITFNDQEI‑E‑RSQTIKP‑ELTRAIQESRISIVVLSQNYASSSWCLNELVEILDCKAT‑G‑QI‑VMTVFYKVNPSDVRKQIGGFGKAFKETCQ‑‑GK‑TE‑TE‑IQSWSKALTYVAN‑IEGEH‑‑SLNWVNEADMIEKIA

>M4DBU9

GEDVRKTFVSHLFCELDRMGINAFRDDLDLE‑RGKSISP‑ELVDVIKGSRFAIVVVSRNYAASSWCLDELLKIMEC‑KDALEQT‑IIPIFYEVDPSDVRRQHGSFGEDVESHR‑‑‑‑‑‑DK‑KK‑VKKWKEALTILAA‑ICGED‑‑SRNWRDESKLIKKIV

>M4E4E6

SKDVGRTFLSHFLEGLKSKGIKTFQNNGI‑M‑RSEYITT‑ELARAIEESRISVVILSKNYPSSSWCLNELQRIMKCKVSLG‑QI‑VMAIFYDVDPSDVREQTGDFGKVFEETCY‑‑GK‑TD‑EQ‑KKKWRKALSHVAV‑IAGEH‑‑SISWASEAEMISKIV

>D1GED4

GPDTRKNFVSFLYKELVAKEIRTFKDDKELE‑RGRLISP‑ELLQAIEGSEIAVVVVSKTYSASNWCLEELVKILKL‑EKKGLIK‑VLPIFYEVDPSHVRWQREEVAKQFKKHE‑‑‑KRQSR‑EK‑VKSWRDALNYLAE‑LSGEC‑‑SKNWEDDSKLVDGIT

>D1GEI5

GADTRHSFTCYLLDFLRRKGIDAFIDEEL‑R‑RGNDL‑‑SGLLERIEQSKISIVVFSENYANSAWCLEELAKIMDCKRT‑FDQV‑VLPVFYKVPASDVRYQTGKFGAPFERSEEVFG‑‑SE‑H‑RVPAWKEALRAASD‑IAGYV‑LPER‑SPECDFVDKIA

>M4FFC5

GEDVRKGFLSHVRKGLESKGIIAFVDEEI‑K‑RGESVCT‑VLVGAIRQSRVAVVLLSPNYASPSWCLDELVEIMKCREEYQ‑QT‑VMTIFYEVDPSDVRKQTGDFGKAFDATCV‑‑GK‑IE‑EV‑KQAWRQALTDVAG‑IAGYH‑‑TSNCDNEAEMINKVA

>M4DSB7

GEDVRKNFLSHIKKEFKRKTITFFNDNGI‑E‑RGESIAP‑ELIRGIRGSKIAIVLLSKNYASSKWCLEELVEIMNCREELG‑QT‑VMAIFYEVDPSDVKKLNGDFGKVFRKTCE‑‑GK‑SK‑ED‑IRRWKQALEKVAT‑IAGYH‑‑SCNWVDEAAMIEDIS

>M4EBB9

GDELRNNFISHLVDALRRNTINVFIDKE‑‑EKKGEDI‑‑NNLFKRIEESKIAVAVFSRRYTESRWCLDELVKMKERAD‑LG‑KLKIFPIFYNVTTYDVKLREGDFGIHFRRLKREY‑R‑SEQHR‑VGKWKEALACVSGK‑TGL‑TFND‑‑KSESDFINNIV

>M4CI31

GADTRKNFVSFLYKELETKGIQTFKDDKALV‑RGRPIAP‑ELVQAIQGTRIAVVVVSPTYSASYWCLEELVKILKL‑EKKGLLV‑VVPIFYEVDPCQVRRQKGEVAEQFKKHK‑‑‑RRYSR‑ER‑VRSWRNALTRVTI‑LSGDC‑‑SKNCKDDATLVDGIT

>M4E9L1

GPDVRRTFLSHLHREFVSKGIVAFKDKEI‑E‑RGHTIGP‑DLVQAIRESRVSIVVLSKNYASSGWCLDELVEILKCKEDQG‑QV‑VMTIFYDVNPSDVRKRCGDFGRAFETTCQ‑‑GK‑TE‑EV‑KQRWNKALTDVAD‑IAGEH‑‑YLNWEDEGEMVENIA

>D1GEG8

GQDVRKTFLSHFLEGLKREGINTFIDNGI‑T‑RSESINS‑ELVRAIKEARIAIVILSNNYDSSSWCLNELQLIMECRLALG‑QT‑VMTIFYEVDPSDVRKQTGDFGKAFEETCD‑‑GK‑TE‑EE‑KHRWRQALTQVAV‑IAGEH‑‑SVSWASEAEMMLKIV

>M4EG62

GPDVRKTLLSHMREQFKRSGITMFNDQEI‑V‑RSQEISP‑SLTNGIRESKISIVILSKKYASSSWCLDELVEILKCKETMG‑QI‑VMTIFYGVEPSDVRFQTGDFGIAFNETCA‑‑DK‑PD‑EE‑RQKWSKALKDVGN‑IAGED‑‑FQKWDNEANMIKKIT

>M4E4G7

GEDVRKGFLSHVRKGLERKGIIAFVDDKI‑E‑RGESVGP‑VLVGAIRQSRVAVVLLSRNYASSSWCLDELVEIMKCRKEDQ‑QK‑VMTIFYEVDPSHVRKQTGDFGKAFEKTCM‑‑GK‑TE‑EV‑KQEWRQALEDVAG‑IAGYH‑‑SSNSDNEAEMIDKVA

>M4E797

GEDTRHTITERVYDALRKEKVRVFRDEGM‑Q‑RGDEINP‑SLVAAMEDSAASVVVLSPRYADSHWCLDELATLCDLRASLR‑RP‑MIPIFYEVDPSHVRKQNDHF‑KDFEVHAKRF‑‑‑KE‑EK‑IQRWRKAMTLVGN‑LSGFV‑CKE‑‑SVDDEMIGLLV

>M4DZK4

GEELRCGFVSHLVEALQRHGINVFIDKL‑‑ESVGQDL‑‑SNLFARIEESTIALVIFSRRYTESRWCLDELVKIKERAA‑QG‑LLKVIPIFFKVEPVTVKQLRGAFGDKFRDREWEY‑R‑CDKPR‑TGRWKEALASVSCK‑TGL‑TFDRK‑TNESTFVRIIV

>M4D9I4

GPDVRRGFLSHLHNVFAKKEITVFNDQKI‑E‑RGHTIGS‑ELVLAIREAEASIVLLSQNYASSSWCLDELVEILKCKEASG‑QI‑VMPIFYDVDPSDVRKQKGGFGIAFEKTCE‑‑GE‑TE‑EQ‑KQRWVDALTYVAT‑IAGEH‑‑SRNWTDEAVMVEKIS

>M4F229

GTDSRRSFVSHLYEALTKEGIKAFHDDRELT‑RGGFIWK‑ELVKAIEESRFAVVVLTEGYATSRWCLEELSLIVDL‑ASKKRLE‑LIPVFLDIDPSELKRRNGCFEKALAKHE‑‑‑LRYDL‑ET‑VGRWRKALAEVGN‑ISGWD‑‑SKTRSEEAVLVQEVV

>M4DLL0

GEQLRHSFVSHLTDAFERHGINFIVDKY‑‑EQRGKDL‑‑KNIFARIEESSIALAIFSTRYPESSWCMDELVKMKKLAD‑KG‑KIQVIPIFYKVSARDVRRQTGKFGDKFWNLARAS‑I‑TSGDQ‑IKKWKEALECISGK‑MGL‑SLKNK‑RYEMDMLQEEA

>M4DII7

SEE‑‑NDFSERLYNALRKE‑VRVFR‑EGM‑E‑QGDEDNK‑RLFKAMEDSAASVVVFTQHYADSRSCLDELATLCDLGTSLD‑RP‑ILPVFFKVDPSHVRKQNDHF‑KDFDEHKKIF‑‑‑SK‑EE‑VQRWRKAMELVGN‑LAGYV‑YKL‑‑KDEDDTIKLVV

>M4ET25

GADVRRGFLSHLLKEFKREAIDTFVDNNI‑E‑RGKSIGP‑RFIKSIRGSKIAIVLLSKNYASSTWCLNELAEIMSCRKDLG‑LI‑VMVIFYEVDPSDVKKLTGHFGRVFRKTCA‑‑GK‑IK‑DD‑IVRWRQALAKVAT‑IAGYH‑‑STNWDNEAAMIEQIA

>M4DZK7

GAQLRHNFIDHLVNAMKGRGINVFIDTD‑‑EQKGKDI‑‑KILLKRIEESRVTLAIFSTKYTESSWCLDELATIKKRVD‑LG‑‑‑‑‑‑‑MLEKVLNNLWESLATTLG‑‑‑VVHEWEY‑R‑CEKSK‑IDEWKKALECVSGK‑IGF‑TLDEK‑SSESNFIGLII

>M4CD36

GEDVRKNFLSHFHKELKLKGNDTFKDDGI‑K‑RSTSIWP‑ELKQAIWESRISIVVLSMNYAGSSWCLNELVEIMECREVSG‑QT‑LMPIFYEVDPSDVRKQKGEFGKAFEKICA‑‑GR‑TV‑EE‑TQRWRQALTNVGS‑IAGEC‑‑SSNWDNDAEMIEKIV

>M4CI33

SEDTAKTFVTDLFSSLSEKGITYYKDEKLEE‑GVSSSGS‑DLSKCIRDSKLAVVVVSESYPTSVLCLNELQTILNL‑QDEGQLS‑VLLIFYGVDTSNIRKQTGEYAEPFRKLG‑‑‑‑‑‑SA‑EK‑VQSWRRTLTKLTG‑ISGLD‑‑SRFWSREAEMVDLIT

>M4DZX4

GEDVRITFLSHFLKELDKRLIIAFKDNET‑Q‑RSLSLGP‑ELKQAIRDSRIAVVIFSNKYASSSWCLNELLEIVKCREECG‑QM‑VIPVFYRLDPSHVRKQTGDFGNIFEETCK‑‑NK‑TE‑EV‑IIQWRRALTDVAN‑TLGYH‑‑SVNWDSEAKMIEEIV

>M4F5R1

GKDVRKSFLSHFLKEFGSKGINLFIDNEI‑T‑RGEYIGP‑ELKKAIQGSRIAIVLLSKRYASSSWCLDELVEIMKCKEELG‑QT‑VMPVFYEVDPTDVKKQAGDFGKVFKKTCK‑‑GK‑TN‑EV‑TRKWSEALAKVAT‑LAGYH‑‑SNNWDNEAKMIEDVA

>M4EFN7

GKDLRRGFVGFLVDALKREKINVFMDEFE‑E‑RGKPL‑‑DSLLTRIEGSRVAVAIFSENYTESNWCLKEAEKMNECREK‑GNLV‑VIPIFYKVEPSTVRGLKGDFGYKLWILAKG‑‑‑‑‑D‑E‑KRKKFDEALESIPN‑LFGIT‑VDDT‑SDECQKINDIV

>M4CAD1

GEDVRKGFLSHVLKEFKSKGINVFIDNEI‑K‑RGESVGP‑ELVKAIRHSRVGVVLLSRNYASSSWCLDELVEIMKCREEVG‑QT‑VMTIFYNVDPSEVRKQTGDFGKAFDETCV‑‑GK‑TE‑EV‑KKAWRQALNDVAG‑IAGYH‑‑SSNCGNEADLINKVA

>M4CAE6

GVDVRVTFLSHLLKEFDKKLITAFKDNEI‑E‑RSRSLDP‑ELKQAIKDSRIAVVIFSQNYASSSWCLNELLEIVK‑‑‑‑CG‑QM‑VIPVFYRLDPSHVRKQTGDFGKIFEETCK‑‑NQ‑TE‑EVIIIQWRRALTDVAN‑TLGYH‑‑SVNWGNEAAMIEEIA

>M4D2G3

GADVRTNFLSHVLKELRSKGINSFIDDDM‑E‑RSKLVGL‑ELIEAIRGSRIAIVLLSRNYASSTWCLNELVEIIKCRQEFS‑QT‑VIPLFYEVDPTDVKKQTGDFGKVFRKTCK‑‑GK‑AK‑ED‑IQRWKCALTEVAQ‑ITGYH‑‑STNWKTEAKMIEDIA

>M4DJB6

GGDTRKNIISHLHKELVRRGIRTFKDDETLE‑TGDRFPE‑RLREAINTSRFAIVVISKNYASSRWCLEELRMIMKL‑QREKNIA‑VIPVFYEVDISDVRNHRSGF‑‑GLVQHH‑‑‑‑‑‑KD‑PK‑IPFWKDALRGIAN‑TQATE‑‑SRKCKDDATLVEGVV

>D1GEB3

GPDTRKIFVGHLYGSLSIRGIFTFKDDRRLE‑PGDSITD‑ELCQAIRTSRFAVVVISKNYATSSWCLDELQLIMEL‑VENKEIE‑VFPIFYEVKPSDVRHQQESF‑‑SL‑‑‑R‑‑‑‑‑‑MT‑EK‑VPGWKKALKDIAN‑RKGME‑‑SSKFSDDATMIEEIV

>M4CV08

GPDTRKNFVSHLYAALCRKGLYTYKDEEM‑E‑KGGLI‑PDELIKAIKTSRFFIVVISENFDNSYWCLEELRAIMEVEEDIN‑‑V‑LIPIFYRVKPGRINREN‑‑LAAAFSDMKH‑‑‑‑‑PE‑ETAMNEWENTLSQLAN‑RASYI‑FSTRYVDEATRIEEVV

>M4CQG9

RPDVGKTFISHLQKQFTSNRITMFDDEGV‑E‑RGHTIDP‑ALTQAIRESTISIVVLTKNYASSSWCLDGLLEILKCRQAGK‑LI‑VMPVFYGVRPNDVQRQTGDFGKGFEKTCR‑‑GK‑TI‑LD‑KGRWSQALNKVCS‑LQGFM‑‑FSPRDDESELFEKIG

>M4DRY0

GEDLRKNFLSHFLKELQRKGITTFIDHEI‑K‑RSKAIGP‑ELVAAIRGSRMAVILLSKNYASSTWCLNELLEIMSCKEEIG‑QT‑VMPVFYEVDPSDVRKQAGDFGNIFEETCL‑‑GK‑SE‑EV‑RQRWSRALTDLAN‑LAGVD‑‑SRLWNNEADMIEKLA

>D7LZJ0

GSDVR‑NFFSFLKDALVKNGINVVTDEDA‑‑‑RGKPI‑‑ENLLKLIKDSRIAIVIFSENYPESTWCLDELVEIEKQMDK‑‑‑MLDSCPIFFEVETCHVKRST‑‑FNNNLLQLARQISKKAEAEK‑‑REWRKALISVSSR‑LGLT‑‑YKK‑‑NQATFVNEIV

>M4FAM9

GDQLRYGFVSHLIDAFERYGIMFFIDKH‑‑EQRGKDL‑‑TNLFVRMKESKIALVIFSSRYAESSWCMDELVNIKKRAE‑KG‑KLEVIPIFYKVRAKDVRAQAGKFGDKFWALAKVS‑‑‑‑SGDQ‑IKKWKDALECISNK‑MGL‑SLRDK‑SSEADFIKGIV

>V4MJX9

GEVVRKSFLSHLLKELDRKSINAFIDDGI‑E‑RSRPIGP‑ELLSAIRESRISIVVFSKSYASST‑C‑‑‑‑‑‑‑‑‑‑‑‑‑‑‑‑‑‑‑‑‑‑‑‑‑‑VDPSEVRKHTGEFGKVFKETCD‑‑GK‑TE‑DQ‑KQRWMQALVDVAN‑MAGED‑‑LRNWCNEASMIEKIA

>M4DHQ0

GADTRKNFVSFLYRDLVAKEIRTFKDDKELE‑RG‑‑‑‑‑‑‑‑‑‑‑‑‑‑‑‑‑‑‑‑‑‑‑‑‑‑‑‑‑‑‑‑‑‑QLVKILKL‑EKQGLIK‑VLPIFYDVDPCDVRRQTGAVKKHFEKHK‑‑‑KRLSR‑EK‑VKSWRDALNYLAE‑LSGEC‑‑SQNWEDDSKLVERIT

>D7KPJ7

R‑DTSHNFTDPLYEALKKE‑LRVWND‑‑L‑E‑RGDELRP‑SLVEAIEDSAASVIILSTNYANSSWCLDELALLCDLRSSLK‑RP‑MIPIFYGVNPSDVRKQSGHF‑EDFNDGEDTA‑‑‑ME‑ES‑YEKYPWICLHVGTYIHLLI‑CKSN‑EKVDELIGLVV

>M4DA47

GEELRSGFVSHLVEALQRHGINVFIDKL‑‑ETIGQDL‑‑SNLFARIEESTIALVIFSRRYTESRWCLDELSQVVEIAS‑KNFLLQVIPIFFKVEPVTVKHLRGAFGDKFRDREWEY‑R‑CDKPR‑TNRWKEALASVSSK‑IGL‑TFDRK‑SNESMFVRIIV

>O65506 (0‑318)

SVDVPKSFLSRIRKELRRKGFEPLIDNET‑E‑RCVSIGP‑ELRNAISVSRIVIVVLSRNYALSPWCLDELVEIMKCKEELG‑QR‑VVTIFYNLDPIDVLKQTGDFGDNFRKTCE‑‑RK‑NK‑ED‑IDRWIKALEQVAT‑IDGYR‑‑SRDWDDEKAMVKKIA

>O65506 (319‑1607)

CEDTLQSFASHLSMDFRRKGISAFVN‑‑‑‑‑‑‑‑‑‑‑‑‑‑‑‑‑DVIERVSASVLVFSKSCVSSTSCLDMLVRVFQCRRKTG‑QL‑VVPVYYGISSSDVVVQEHKSV‑‑‑‑‑‑‑‑‑‑‑‑‑‑‑‑DR‑IREWSSALQELRE‑LPGHH‑‑NRE‑CSESELVEEIV

>M4FEN8 (0‑354)

EEEISRGFFGYFKKEFERKGIKLFID‑‑‑‑‑‑RRESFGP‑GLIEAIRRSRIAIVILSKHYASSTLRLDELVEIMKCRE‑‑‑‑‑‑‑‑‑‑‑‑‑ELGAADVKKQSGYFGSVFEKACV‑‑GR‑SV‑ED‑VEKWKRALNELSF‑IFGYL‑‑SGNWKSEDDMMEEVA

>M4FEN8 (355‑1330)

GVDVRKSFLRHMLMVLRNKGITLFTDIEI‑E‑TGTSIAH‑ELKEAIHRSRISIILISNKYVSRSWEMEEIIE‑‑‑‑‑‑KVA‑NN‑LSDLLYEDVPS‑‑‑‑‑‑GDFDGIWGPSGI‑‑GK‑TT‑TA‑RALYRKLSSNFTH‑TAFME‑‑SIKAKNEAEMIENVA

>F4JNL1 (0‑278)

GKAQRKTLVSFIKSKLEESEINVFMDEY‑‑EIRGRPI‑‑TTLFERIRESSIALVIFSDKYPESRWCLDELVEIKKQME‑TG‑SIP‑FPIFYKVKAESVKNQTGHFRNVLLKTEEDV‑RKKTEDM‑IWGWRQALVSVGGR‑MGF‑SY‑NH‑KCDNDFVNDIV

>F4JNL1 (279‑635)

EKHLGKFLVSSLKEELESNQILVYV‑ED‑‑ETK‑‑‑‑‑‑‑‑‑‑SRIKESGVAVVFFSKKYPNSEKCLDELVEIKKLMD‑AG‑KIP‑LPVFYSLKDEPVKNLKGYFLNRLLKIENEV‑RKNTEAK‑IWGWRDALSSIASR‑PGL‑SY‑EL‑STDDVFVSDIV

>F4JT82

WEDTIRSFVSHLSAEFQRKGVSVFASEDS‑A‑SDRF‑‑‑‑‑‑‑AAIAKARVSVVIFSENFASSKGCLNEFLKVSKCRRSKG‑LV‑VVPVFYGLTNSIVKKHCLELKKMY‑‑‑‑P‑‑‑‑‑‑D‑DK‑VDEWRNALWDIAD‑LGGHV‑‑SSH‑RSDSELVEKIV

>D7MRD9 (0‑469)

GADVRKTFLSHFLKELDLKSIKPFKDSEI‑E‑RSHSIAP‑ELIQAIRGSRIAVVVFSENYATSKWCLDELVEILKCKEELG‑QI‑VIPIFYDLDPFHVRKQLGKFGEAFKNTCL‑‑NK‑TK‑NE‑IQLWRQALNDVAN‑LLGYH‑‑SHTCNNEPKMIEDIV

>D7MRD9 (470‑1750)

GADVRKTFLSHFLKELDLKSIKPFKDSEI‑E‑RSHSIAP‑ELIQAIRGSRIAVVVFSENYATSKWCLDELVEILKCKEELG‑QI‑VIPIFYALDPFHVRKQLGKFGEAFKKTCL‑‑NK‑TE‑DE‑RQLWRQALTDVAN‑LLGYH‑‑SHTCNSEAKMIEDIV

>F4J910

GEDIQESLMVHVDKEFQRKGITPFNDNEI‑K‑RGESISP‑ELVLAIRGSRIALILLSRNYASSSWCLDELAEIIKCREEFG‑QT‑VMVVFYKVDPSDIKKLTGDFGSVFRKTCA‑‑GK‑TN‑ED‑TRRWIQALAKVAT‑LAGYV‑‑SNNWDNEAVMIEKIA

>Q56XU3 (0‑206)

GEELRKSFLGFLLKAMRDAKINVFTDEI‑‑EVRGRDI‑‑QNLLSRIEESRVAIAILSKRYTESSWCLDELVKMKERID‑QD‑ELVVIPIFYRLDATNCKRLEGPFGDNFRNLERDY‑R‑SEPER‑IKKWKEALISIPQK‑IGL‑TSAGH‑RD‑SELVDSIV

>Q56XU3 (207‑419)

NDELGDNFIKHLVWALRDSGINVFKDSF‑‑KLIGGQK‑‑QEVFMSIENSNIALAIFSKRYSESYRCLNELVKMEELAK‑EG‑KLVVIPVFYSVKTNEVRRLEGEFGIHFRNTKERF‑A‑MEPMM‑VESWEKSL‑‑‑‑KR‑IGL‑SLEAH‑MNEFALVGAIV

>M4EA32

GEDVRVRFRSHFLKELNRKLITPFKDDEI‑V‑KGRSIGH‑ELINAIRGSRISVVAFSDNYASSSWCLDELVEIIKCREELG‑QI‑LIPIFYDVDPSHVKKQTERFGVIFEKTCQ‑‑GR‑KE‑EE‑KLRWRRALTHAAT‑IAGED‑‑SRNWSDEAKMIEKIV

>D7MKB0 (0‑266)

GRDLRYGFVSHLEKILKDHKIEVFVDSGE‑D‑RGEHL‑‑ENLLTRIEESRIALAIFSENYTESEWCLRELAKIKDCVDQ‑KRLV‑AIPIYYKVDRYSVKYLTEKFGDAFRELAKD‑‑‑‑‑D‑L‑RKKEWKQALQSIVN‑RIGFM‑VDEK‑SNENEILNEIV

>D7MKB0 (267‑495)

GDQLRNNFVGYLRHALRISKINVFIDNE‑‑EQRGEDL‑‑NTLFKRIEESQIAIVVFSSRYTESKWCLDELVKIKERVD‑QG‑LL‑‑‑‑‑‑‑‑‑‑‑‑‑‑‑‑‑KGEFGDHFRDIEYMY‑K‑SDEPM‑IKQWKEAIVFVSHK‑FAL‑TLDEK‑SSEIDFVETIV

>Q9FKE3 (0‑265)

GS‑‑RMGFIYHLIMALEKKNINVFVGFNG‑C‑ICEPV‑‑ERLSNRIE‑SIIVLVIFTSRYTESKWCLMKLVDINKCAEK‑DHLV‑AIPIFYKLDPSTVRGLSGQFGDAFRDLRES‑GL‑‑‑‑‑‑‑‑EKWKEALKSISD‑RPGIR‑VDKS‑SPKAKRIEIV‑

>Q9FKE3 (266‑546)

GDQLRNNFVGYLVDALRRSEINVFIDNQ‑‑EQRGEDL‑‑NTLFKRIEDSGIAIVVFSSRYTESKWCLEELVKIKERVH‑QG‑LLKVLPIFYKVTPTNVKRPKGEFGDHFRDKEYMY‑E‑SDEPM‑IKRWKEAIVFISHR‑FAL‑TLDEKWSSEIDFVETIV

>D7MIU3

GPDVRRGFLSHLHNHFTSKGITTFKDQEI‑E‑RGQTIGP‑ELVQAIRESRISVVVLSKSYGSSSWCLDELVEILRCKEDQG‑QI‑VMTIFYEIDTSDVRKQSGDFGRDFKRTCE‑‑GK‑TE‑EV‑KQRWIQALAHVAT‑IAGEH‑‑LLNWDNEAAMVQKFA

>V4LWJ7 (0‑268)

GKGQRDKLVSFLKKQLERSDINFFMDEN‑‑EVRGMPL‑‑TTLFERIRESSVALVFFSDKYPESCWCLDELVEIKKQME‑KG‑SLP‑FPIFYKVKAETIKRQTGCFGNSLLRTEDLV‑RKKTEAV‑IWERRQALVSVGGR‑MGF‑SY‑KH‑SSDEAFVSDLV

>V4LWJ7 (269‑600)

EKQLRENLVSFLKTELESNRISVCV‑ED‑‑EMK‑‑‑‑‑‑‑‑‑‑KRIKESKVAIIIFSAKYPESQHCLDELVEIKKLMD‑TG‑EIP‑FPIFYKLKAESVKVIKGWFRNRLLKIEEKV‑RKNTEAR‑IWGWREALASLVSR‑PGL‑SY‑QH‑SSDSLFVTDVV

>M4D5A8

CEETLQTFASHLSVDFRRKRIAAFVN‑‑‑‑‑‑‑‑‑‑‑‑‑‑‑‑‑DVAEGASASVVVFSKSYSSSASCLDKLVTVLRCRRNTG‑QMVVVPVFYGISPSDVAVRVHGSA‑‑‑‑‑‑‑‑‑‑‑‑‑‑‑‑DR‑IREWSNALRELRE‑LPSHQ‑‑CSE‑SDEGQVVEEIV

>D7KPJ9

GEDTREIFAGPLYKALKEK‑VRVFLDDGM‑E‑RGDEIGS‑SLQAGMEDSAASVIVLSRNYANSRWCLNELAMLCKLKSSLD‑RR‑MLPIFYKVDPSHVRKQSDHI‑ADFKRHEERF‑‑‑DK‑EK‑VQEWRDAMKLVGN‑LAGYV‑CVE‑‑SNEDEMIELVV

>F4HR54

GFDTRTNFCERLYIALEKQNVRVFRDEGM‑E‑KGDKIDP‑SLFEAIEDSAASVIILSTNYANSSWCLDELALLCDLRSSLK‑RP‑MIPIFYGVNPEDVRKQSGEF‑KDFEEKAKSF‑‑‑DE‑ET‑IQRWKRAMNLVGN‑IPGYV‑CTAN‑EKVDDMIDLVV

>F4JT78 (0‑382)

KVDVRRSFLAHLLKELDRRLINTFTDHGM‑E‑RNLPIDA‑ELLSAIAESRISIVIFSKNYASSTWCLDELVEIHTCYKELA‑QI‑VVPVFFNVHPSQVKKQTGEFGKVFGKTCK‑‑GK‑PE‑NR‑KLRWMQALAAVAN‑IAGYD‑‑LQNWPDEAVMIEMVA

>F4JT78 (383‑1309)

NLDVDESFIEAISKELHKQGFIPLTYN‑‑‑‑‑‑‑‑‑‑‑‑‑‑LLEMLYGSRVGIMILSSSYVSSRQSLDHLVAVMEHWKTTD‑LV‑IIPIYFKVRLSDICGLKGRFEAAFLQLHM‑‑‑‑‑SE‑DR‑VQKWKAAMSEIVS‑IGGHE‑‑WTK‑GSQFILAEEVV

>M4DB38

EKQLKMNLVSSLKTEFESNEISVYI‑ED‑‑ETK‑‑‑‑‑‑‑‑‑‑ERIKESKVAIVVFSDKYPESPQCLDELVEIKKLMD‑AG‑EIP‑FPIFYKLKAQSVKQLKGCFRNRLLKIEQEV‑HKTTEAR‑IWDWRQAISSISSR‑PGL‑SN‑EN‑SSDPVFFTDVV

>D7M939 (0‑224)

GKDERYGFLTHLKQKLIDGNVNVFT‑DD‑‑NVTGQPL‑‑QNLFGHIRKSRIVIVIFSKNYAESDWCLDELVEIKKCFE‑TE‑ALAVIPIFHKVKVSSVKKQSGKFGEKFLALQNSLNKKK‑‑SR‑IKRWKKALKIVTEI‑AGL‑THDKN‑SPELAFVEKVV

>D7M939 (225‑506)

GKAQRKTLVSFIKSKLEESEINVFMDEY‑‑EIRGRPI‑‑TTLFERIRESSIALIIFSDKYPESRWCLDELVEIKKQMD‑TG‑SIP‑FPIFYKVKAESVKYQTGHFRNVLLKTEDDV‑RKKTEDM‑IWGWRQALVSVGGR‑MGF‑SY‑NH‑KCDNDFVNDIV

>D7M939 (507‑879)

EKHLGKFLVNSLKEELESNQILVYA‑ED‑‑ETK‑‑‑‑‑‑‑‑‑‑SRIKESGVAVVVFSNKYPKSEKCLDELVEIKKLMD‑AG‑KIP‑LPVFYSLKVEPVKKLKGCFLNRLLKIENEV‑RKNTEAK‑IWGWRDALSSIASR‑PGL‑SY‑EL‑STDDVFVSDIV

>9758205

GEDVRKSFLSHLLKKLHRKSINTFIDNNI‑E‑RSHAIAP‑DLLSAINNSMISIVVFSKKYASSTWCLNELVEIHKCYKELT‑QI‑VIPIFYEVDPSDVRKQTREFGEFFKVTCV‑‑GK‑TE‑DV‑KQQWIEALEEVAS‑IAGHD‑‑SKNWPNEANMIEHIA

>12324938

GPDVRKTFLSHLRKQFICNGTTMFDDQAI‑E‑RGQTISP‑ELTRGIRESRISIVVLSKNYASSSWCLDELLEILKCKEDIG‑QI‑VMTVFYGVDPSDVRKQTGDILKVFKKTCS‑‑GK‑TE‑EK‑RRRWSQALNDVGN‑IAGEH‑‑FLNWDNESKMMEKIA

>685339467

GTDVRKGFLSHLYKALTDNGIHTFRDDAELQ‑RGNFISP‑ALLGAIEQSRFAVVVLSENYATSRWCLQELVHITKC‑VEKKQME‑LIPVFFGVDPSHVKRQSG‑FAKAFAEHD‑‑‑KRPNK‑DA‑VESWRKAMATVGF‑ISGWD‑‑SRNWNEESKLIEELV

>685356609

GEDVRKNFLSHLLKEFENKGILTFRDDQI‑E‑RSHSIGP‑ELVEAIRESKISVVLFSENYASSSWCLDELVEILKCKEEQG‑LK‑VMPIFYKVDPSEVRKQTGKFGMGFLKTCH‑‑GK‑TE‑EQ‑QQSWRQALTDAAS‑IVGDH‑‑PQDWDNEADMITTIA

>923621281

GSDTKRNIATLLYDNLNARNLRPFLD‑‑‑‑S‑‑‑KNMKPDKLFRAILTSKVAVTVFSPNYCDSYFCLHELALIMESKK‑‑‑‑R‑‑IIPIFFDINPSQLD‑‑‑‑‑‑‑VMIEKVCS‑‑‑‑‑‑D‑NE‑IQRW‑‑‑‑‑‑‑‑‑‑L‑‑‑‑‑‑‑‑Q‑‑‑EAKTV‑‑‑‑

>685355951

GQGLRQTFLSHLCRQLNENGITVFTNQDL‑V‑RGEPVLP‑SLVQRIRESRISIVVLSQKYASSSWCLNELVEILRCRETMG‑HI‑VMTIFYRVDPSHVRNQTGDFGNIFVQTCA‑‑GK‑TE‑EE‑RRMWSQALTDVGN‑IAGED‑‑SRNWDNESKMIEKIV

**Aligned Fabale sequences from Pfam used to build profile HMM after alignment pruning and exclusion.**

>K7MH68

GEDTRYGFTGNLYNVLRERGIHTFIDDEELQKGDEITTALEEAIEKSKIFIIVLSENYAYSSFCLNELTHILNFT‑EGKNPLVLPVFYKVNPSYVRHHRGSYGEALANHEKKLNSN‑‑‑‑‑NMEKLETWKMALRQVSNISGHHL‑QHGNKYEYKFIKEIV

>K7MIY3

GEDTRYGFTGNLYRALSDKGIRTFFDEEKLHSGEEITPALLKAIKDSRIAITVLSEDFASSSFCLDELTSIVHCA‑QYNGMMIIPVFYKVYPSDVRHQKGTYGEALAKHKIRF‑‑‑‑‑P‑‑‑‑EKFQNWEMALRQVADLSGFHF‑KYRDEYEYKFIERIV

>C6ZS22

GEDTRYTFTSHLYAALTRLQVKTYIDN‑ELERGDEISPSLLRAIDDAKVAVIVFSENYASSRWCLDELVKIMECK‑RKNGQIIVPVFYHVDPTHVRHQTGSYGHAFAMHEQRFVGNM‑‑‑‑‑‑NKVQTWRLVLGEVANISGWDC‑LT‑TRVESELVEKIA

>K7LYT2

GEDTRRNFTCHLYEALMQKKIKTYIDE‑QLEKGDQIALALTKAIEDSCISIVIFSDNYASSKWCLGELFKILECK‑KEKGQIVIPVFYNIDPSHVRKQIGSYKQAFAKLEGEP‑‑‑‑‑‑‑‑‑‑‑ECNKWKDALTEAANLVGLDS‑KN‑YRNDVELLKDIV

>K7MWR6

GEDTRHSFTGNLYKALSDRGIHTFIDDKKLPRGDQISSALEKAIEESRIFIIVLSENYASSSFCLNELGYILKFI‑KGKGLLVLPVFYKVDPSDVRNHAGSFGESLAHHEKKFNAD‑‑KETNLVKLETWKMALHQVANLSGYHF‑KHGEEYEYKFIQRIV

>I1KEG1

GEDTRYEFTGHLHQALCKKGIRAFFDEEDLQTGDEITTKLEEAIKGSRIAITVFSKGYASSSFCLNELATILGCY‑REKTLLVIPVFYKVDPSDVRHQRGSYEQGLDSLEKRLHPN‑‑‑‑‑‑‑‑‑MEKWRTALHEVAGFSGHHF‑TDGAGYEYQFIEKIV

>K7N1L0

GEDTRMNFTSHLHEALKQKKVETYIDY‑QLEKGDEISPALIKAIEDSHVSIVILSENYASSKWCLEELSKILECK‑KKQGQIVIPVFHNIDPSHVRKQNGSYEKAFAKHEGEA‑‑‑‑‑‑‑‑‑‑‑KCNKWKATLTEVANLAGWDS‑R‑‑NRTESELLKDIV

>K7KXK5

GEDTRNNFTAFLFDALFENGIHAFKDDTHLQKGESIAPELLLAIQESRLFLVVFSKNYASSTWCLRELAHICNCTIEPSSSRVLPIFYDVDPSEVRKQSGYYGIAFAEHERRFRED‑‑IE‑KMEEVQRWREALIQVANISGWDI‑Q‑‑NESQPAMIKEIV

>I1JM77

GEDTRRSFVCHLNCALSKAGVKTFLDEENLHKGMKL‑DELMTAIEGSQIAIVVFSKSYTESTWCLRELEKVIECN‑ETYGQSVLPVFYNIDPSVVRHEKHDFGKVLKSTAEK‑‑‑‑‑GEHL‑ENALSRWSRALSEASKFSGWDASK‑‑FRNDAELVEKIV

>K7L9U4

GDDTRSGFTGSLYKSLCDQGIHTFMDDEGLRRGEEIRHALFKAIQQSRIAIVVFSENYASSTYCLEELVMILECI‑MKKGRLVWPVFYGVTPSYVRHQKGSYGKALDKLGERF‑‑‑‑‑KN‑DKEKLQKWKLALQEAANLSGSHF‑KLKHGYEHEVIQKIV

>Q84ZV1

GEDTRYGFTSNLYRALSDKGIRTFFDEEKLHSGEEITPALLKAIKDSRIAITVLSEDFASSSFCLDELTSIVHCA‑QYNGMMIIPVFYKVYPSDVRHQKGTYGEALAKHKIRF‑‑‑‑‑P‑‑‑‑EKFQNWEMALRQVADLSGFHF‑KYRDEYEYKFIERIV

>K7MHM7

GKDTRQNFTGHLYNSLFKNGILTFIDDKGLRRGEEITPALLNAIKNSRIAIIVFSEDYASSTYCLDELVTILESF‑KEEGRSIYPIFYYVDPSQVRHQTGTYSDALAKHEERFQYD‑‑‑‑‑I‑DKVQQWRQALYQAANLSGWHF‑HGS‑QPEYKFILKIV

>I1KEB4

GEDTRNSFTAFLFGALKKQGIEAFKDDKDIRKGESIAPELIRAIEGSHVFVVVFSKDYASSTWCLRELAHIWDC‑IQTSHRPLLPIFYDVDPSQVRNQSGDYEKAFAQHQQRFQE‑‑‑‑‑‑‑‑KEIKTWREVLEQVASLSGWDI‑R‑‑NKQQHPVIEEIV

>K7MRQ3

GEDTRHTFTAHLLAAFYRLKIRTYVDY‑KLGRGDEISPTLIRAIEESKVSVIVLSKNYATSKWCLEELVKIMECR‑RTKGQIAIPVFYHVDPSDVRNQTGSYADAFANHEQRFKDNV‑‑‑‑‑‑QKVELWRASLREVTNLSGWDC‑LV‑NRTFEKFVEIKA

>K7MH74

GEDTRYCFTGNLYNVLRERGIHTFIDDDELQKGDQITSALQEAIEKSKIFIIVLSENYASSSFCLNELTHILNFT‑KGKNLLVLPVFYIVDPSDVRHHRGSFGEALANHEKKLNST‑‑‑‑‑NMENLETWKIALHQVSNISGYHF‑QHGDKYEYKFIKEIV

>K7KCW4

GEDIRHGFLGYLTEAFHQKQIHAFIDD‑KLEKGDEIWPSLVGAIQGSLISLTIFSENYSSSRWCLEELVKIIECR‑ETYGQTVIPVFYHVNPTDVRHQKGSYEKALSEHEK‑‑‑‑‑‑‑KY‑NLTTVQNWRHALKKAADLSGIKS‑FD‑YKTEVELLGEIV

>K7LUI8

GLDTRNSFTDHLFAALQRKGIFAFRDNQNINKGELLEPELLQAIEGSHVFIVVFSKDYASSTWCLKELRKIFDG‑VEETGRSVLPIFYDVTPSEVRKQSGKFGKAFAEHEERFK‑‑‑‑‑D‑ELEMVKKWREALKAIGNRSGWDV‑Q‑‑NKPEHEEIEKIV

>I1M0Q0

GKDTRFTFTGNLYRALQDRGFRVFMDDQKIDKGKKISQELPKAIKESRIYIIVLSENFASSWYCLVEVVMILDEFAK‑KGR‑IVPIFFYVDPSVLV‑‑‑RTYEQALADQRK‑‑‑‑‑‑‑‑‑‑SDDKIEEWRTALTKLSKFPGFCVSRDG‑NFEYQHIDEIV

>K7LW75

GPDTRNTFVDHLYAHLLRKGIFVFKDDKKLQKGESISAQLLQAIQDSRLSIIVFSKQYASSTWCLDEMAAIADC‑KQQSNQTVFPVFYDVDPSHVRHQNGAYEVAFVSHRSRFR‑‑‑‑‑E‑DPDKVDRWARAMTDLANSAGWDV‑M‑‑NKPEFREIENIV

>K7KDW7

GEDIRKNFVSHLHSALLHAEVKTFLDDENLLKGMKS‑EELIRAIEGSQIAVVVFSKTYTESSLCLRELEKIIESH‑ETRGQRVLPIFYEVDPSDVRQQKGDFGEALKAAAQK‑‑‑‑‑GEHL‑ESGLSRWSQAITKAANLPGWDESN‑‑HENDAELVEGII

>K7KQM9

ATDTRSNFTDFLFQALIRKGIVAFKD‑‑ESRAPDQ‑‑‑‑‑‑‑AIEDSRLFIVVLSKNYAFSTQCLHELSQIFHC‑‑ES‑‑RRVLPIFYDVDPSDVRKQTG‑YEKAFSKYEERFLVN‑‑KKG‑METVQTWRKALTQVANLSGWYI‑R‑‑NKPQYTEIEEFV

>K7KCV9

‑‑‑LETNYGHHLL‑‑‑‑‑‑‑‑‑‑‑‑‑‑‑‑‑‑‑‑‑‑‑‑‑‑‑‑‑‑‑‑GSLISLTILSENYASSSWSLNELVTILECR‑EKYNRIVIPVFYKVYPTDVRHQNGSYKSDFAEHEK‑‑‑‑‑‑‑KY‑NLATVQNWRHALSKAANLSGIKS‑FN‑YKTEVELLEKIV

>K7KV37

GSDTRHGFTGNLYKALADRGIYTFIDDEELQSGKEITPTLLKAIQESRIAINALSINYASSSFCLDELATILGCA‑ERKTLLVL‑‑‑‑‑‑‑PSHVRHREDSYGEALVKHEERF‑‑‑‑‑EH‑NTEKLQKWKMTLYQVALLSGYHF‑KYGDGYEYEFIGRIV

>K7LE88

GEDTRHGFTGHLYSALHSKGIHTFIDDEGLQRGEEITPALVKAIQESKIAIIVLSINYASSSFCLHELATILECL‑MGKGRLVLPVFYKVDPSHVRHQNGSYEEALAKHEERF‑‑‑‑‑KA‑EKEKLQKWKMALHQVANLSGYHF‑KDGEGYEYKFIEKIV

>K7KDW4

GEDTRKKFVCHIYKALSNAGINTFIDEENIQKGMTL‑DELMTAIEGSQIAIVVFSKTYTESTWCLRELQKIIECH‑ENYGQRVVPVFYHIDPSHIRHQEGDFGSALNAVAER‑‑‑‑‑GEDL‑KSALSNWKRVLKKATDFSGWNERD‑‑FRNDAELVKEIV

>K7MH79

GEDTRHGFTGNLYNVLRERGIDTFIDDEELQKGHEITKALEEAIEKSKIFIIVLSENYASSSFCLNELTHILNFT‑KGKSRSILPVFYKVDPSDVRYHRGSFGEALANHEKKLKSN‑‑‑‑‑YMEKLQIWKMALQQVSNFSGHHF‑QPGDKYEYDFIKEIV

>K7KXJ5

GEDTRNSFTAFLFDALSQNGIHAFKDDTHLQKGESIAPELLLAIQGSGLFVVVFSKNYASSTWCLRELAHICNCTIQASPSRVLPIFYDVDPSELRKQSGYYGIAFAEHERRFRGD‑‑KE‑KMEELQRWREALKQVANISGWNI‑Q‑‑NESQPAVIEKIV

>I1MND6

REDTRHGFTGNLYNVLRERGIHTFIDDDEPQKADQITKALEEAIKNSKIFIIVLSENYASSFFCLNELTHILNFT‑KGWDVLVLPVFYKVDPSDVRHHRGSFGEALANHEKNLNSN‑‑‑‑‑YMGKLKTWKMALRQVSNFSGHHF‑QPGNKYEYKFIKEIL

>K7KBB3

GEDTRNNFIGHLRKELSRKGMKIFFDDRDLPVGNVISPSLSKAIEESKILIIVFSKNYASSTWCLDELVKILEQS‑KEMKQLVFPVFYHVDPSDVRKQTESYGEHMTKHEENF‑‑‑‑‑GK‑ASQKLQAWRTALFEASNFPGHHI‑TT‑SGYEIDFIEKIV

>K7K6Z8

GEDTRQKFTGNLYNSLCEKGVHTFIDDEGLRRGEEITPALLNAIQNSRIAIVVFSKNYASSTFCLDKLVKILECL‑KEKGRSVFPIFYDVDPSHVRHQKGTYSEALAKHEERFPDD‑‑‑‑‑S‑DKVQKWRKALYEAANLSGWHF‑QHG‑ELEYKSIRKIV

>K7KDV6

GEDTRRSFVCHLNCALSKAGVKTFLDEENLHKGMKL‑DELMTAIEGSQIAIVVFSKSYTESTWCLRELEKVIECN‑ETYGQSVLPVFYNIDPSVVRHEKHDFGKVLKSTAEK‑‑‑‑‑GEHL‑ENALSRWSRALSEASKFSGWDASK‑‑FRNDAELVEKIV

>K7MIM5

GLDTRHGFTGNLYKALDDRGIYTFIDDQELPRGDQITPALSKAIQESRIAITVLSENYASSSFCLDELVTILHCK‑S‑EGLLVIPVFYKVDPSDVRHQKGSYGEAMAKHQKSF‑‑‑‑‑KA‑KKEKLQKWRMALQQVADLSGYHF‑KDGDAYEYKFIGSIV

>I1J5B8

GEDTRDNFISHIYAELQRNKIETYIDY‑RLARGEEISPALHKAIEESMIYVVVFSQNYASSTWCLDELTKILNCK‑KRYGRVVIPVFYKVDPSIVRNQRETYAEAFVKYKHRFADNI‑‑‑‑‑‑DKVHAWKAALTEAAEIAGWDS‑QK‑TSPEATLVAEIV

>K7MIZ0

GEDTRHGFTGNLYKALDDRGIYTFIDDQELPRGDQITSALSKAIQESRIAITVLSQNYASSSFCLDELVTILHCK‑R‑KGLLVIPVFYKIDPSDVRHQKGSYGEAMAKHQKSF‑‑‑‑‑KA‑K‑‑KLQKWRMALQQVADLSGYHF‑KDGDSYEYKFIGSIV

>K7MDD3

GEDTRLGFVGNLYKALTEKGFHTFFRE‑KLVRGEEIAASPSKAIQHSRVFVVVFSQNYASSTRCLEELLSILR‑FSQDNRRPVLPVFYYVDPSDVGLQTGIYGEALAMHEKRF‑‑‑‑‑NS‑ESDKVMKWRKALCEAAALSGWPF‑KHGDGYEYELIEKIV

>I1LWA0

GEDTRKNFTSHLYEALKQKKIETYIDY‑RLEKGDEISAALIKAIEDSHVSVVIFSENYASSKWCLGELGKIMECK‑KERGQIVIPVFYNIDPSHVRKQTGSYEQSFAKHTGEP‑‑‑‑‑‑‑‑‑‑‑RCSKWKAALTEAANLAAWDS‑QI‑YRTESEFLKDIV

>K7MRQ4

GEDTRHTFTAHLLAAFYRLKIRTYVDY‑KLGRGDEISPTLIRAIEESKVSVIVLSKNYATSKWCLEELVKIMECR‑RTKGQIAIPVFYHVDPSDVRNQTGSYADAFANHEQRFKDNV‑‑‑‑‑‑QKVELWRASLREVTNLSGWDC‑LV‑NRTESQLVEDA‑

>I1MND4

GEDTRYGFTGYLYNVLRERGIHTFIDDDEPQEGDEITTALEAAIEKSKIFIIVLSENYASSSFCLNSLTHILNFT‑KENNVLVLPVFYRVNPSDVRHHRGSFGEALANHEKKSNSN‑‑‑‑‑NMEKLETWKMALHQVSNISGHHF‑QHGNKYEYKFIKEIV

>K7MWS1

GKDTRHSFTGNLYKALSERGINTFIDDKKLPRGDEITSALEKAIEESRIFIIVLSENYAWSSFCLNELDYILKFI‑KGKGLLVLPVFYKVDPSDVRNHTGSFGESLAYHEKKFKST‑‑N‑‑NMEKLETWKMALNQVANLSGYHF‑KHGEEYEYQFIQRIV

>I1M0P9

GEDTRRSFTGNLYNCLEKRGIHTFIGDYDFESGEEIKASLSEAIEHSRVFVIVFSENYASSSWCLDGLVRILD‑FTEDNHRPVIPVFFDVEPSHVRHQKGIYGEALAMHERRL‑‑‑‑‑NP‑ESYKVMKWRNALRQAANLSGYAF‑KHGDGYEYKLIEKIV

>K7L9P5

GIDTRDTITKGLYSSLEARGVRVFLDDVGLERGEEIKQGLMEAIDDSAAFIVIISESYATSHWCLEELTKI‑‑C‑‑D‑TGRLVLPVFYRVDPSHVRDQKG‑FEAGFVEHERRFG‑‑‑‑‑‑‑‑KNEVSMWREAFNKLGGVSGWPFDS‑‑‑‑EEDTLIRLLV

>I1L1A5

GQDIRDGFLSHLIDTFERKKINFFVDY‑NLEKGDEIWPSLVGAIRGSLILLVIFSPDYASSCWCLEELVKILECR‑EEYGRIVIPVFYHIQPTHVRHQLGSYAEAFAVHGR‑‑‑‑‑‑‑KQ‑M‑MKVQHWRHALNKSADLAGIDS‑SK‑FPNDAAVLNEIV

>I1N650

GSDTRHGFVGNLYKALNDKGIHTFIDDEKLQGGEEITPTLMKAIEESQIAITVLSHNYASSSFCLDELVHIIDC‑‑KRKGLLVLPVFYNLDPSDVRHQKGSYGEALARHEERFKAKKLNQ‑NMERLEKWKMALHQVANLSGYHF‑KQGDGYEYEFIGKIV

>K7LWN8

GIDTRHSFTDNLYNSLKQRGIHAFIDDEGLRRGEEITPTLLKAIRESRIGIIVFSKSYASSTYCLDELVEILECL‑KE‑GRLVWPVFYDVDPSQVRYQTGTYAEALAKHKERFQDD‑‑‑‑‑K‑GKVQKWRKALHEAANLSGWHF‑QHGSESEYKFIKKIV

>K7K614

SEDTRKTFTSHLNAALERLDIKTYLDN‑NLDRGEEIPTTLVRAIEEAKLSVIVFSKNYADSKWCLDELLKILEFG‑RAKTLIIMPVFYDIDPSDVRNQRGTYAEAFDKHERYFQEKK‑‑‑‑‑‑‑KLQEWRKGLVEAANYSGWDC‑DV‑NRTESEIVEEIA

>K7MH77

GEDTRHGFTGNLYNVLRERGIDTFIDDEELQKGHEITKALEEAIEKSKIFIIVLSENYASSSFCLNELTHILNFT‑KGKSRSILPVFYKVDPSDVRYHRGSFGEALANHEKKLKSN‑‑‑‑‑YMEKLQIWKMALQQVSNFSGHHF‑QPGDKYEYDFIKEIV

>I1NDU4

GEDTRHTFTCKLYDALWLKGIDTFMDNKELKNGDKIGPTLHKAIEEARISVVVLSENYADSSWCLDELVKIHECM‑ESKNQLVWPIFYKVNPSDVRHQKGSYGVAMTKHETSP‑‑‑‑‑GI‑DLEKVHKWRSTLNEIANLKGKYL‑EE‑GRDESKFIDDLA

>K7KYE4

GEDTRRTFTGSLYHGLHQRGINVFIDDEKLRRGEEISPALIGAIEESRIAIIVFSQNYASSTWCLDELAKILECY‑KTRGQLVWPVFFHVDPSAVRHQRGSFATAMAKHEDRF‑‑‑‑‑KG‑DVQKLQKWKMALFEAANLSGWTL‑K‑‑NGYEFKLIQEII

>K7MMQ0

GEDTRRNTTSHLYEALLHKKIESYIDY‑QLEKGDEIT‑‑LIQAIKD‑‑‑SAYLFS‑‑‑FSQRTML‑‑‑‑‑‑‑‑‑‑‑PQSGQIVIPVFYNIYPSHVRKKTGSYDQAFAKHVGEP‑‑‑‑‑‑‑‑‑‑‑RCNKWKAALTEAANLAGWDS‑QT‑FWTDSELLKDII

>I1NDU3

GEDTRHTFTCKLYDALWLKGIDTFMDNKELKNGDKIGPTLHKAIEEARISVVVLSENYADSSWCLDELVKIHECM‑ESKNQLVWPIFYKVNPSDVRHQKGSYGVAMTKHETSP‑‑‑‑‑GI‑DLEKVHKWRSTLNEIANLKGKYL‑EE‑GRDESKFIDDLA

>K7L9P4

GIDTRDTITKGLYSSLEARGVRVFLDDVGLERGEEIKQGLMEAIDDSAAFIVIISESYATSHWCLEELTKI‑‑C‑‑D‑TGRLVLPVFYRVDPSHVRDQKG‑FEAGFVEHERRFG‑‑‑‑‑‑‑‑KNEVSMWREAFNKLGGVSGWPFDS‑‑‑‑EEDTLIRLLV

>K7MH72

GEDTRYGFTGNLYNVLRERGIHTFIDDDELQKGDEITTALEEAIEKSKIFIIVLSENYASSSFCLNELTHILNFT‑EGKNRLVLPVFYKVNPSIVRKHRGSYGEALANHEKKLNSN‑‑‑‑‑NMEKLETWKMALQQVSNISGHHF‑QHGGKYEYKFIKEIV

>K7LC01

GVDIRRGFLSHLIGTFKSKQINAFVDD‑KLERGEEIWPSLIEAIQGSSISLIIFSPDYASSRWCLEELVTILECK‑EKYGQIVIPIFYHIEPTEVRHQRGSYENAFAEHVK‑‑‑‑‑‑‑KY‑K‑SKVQIWRHAMNKSVDLSGIES‑SK‑FQDDDELLKEIV

>K7KXN2

GEDTRYEFTGHLHKAHCNKGIRAFIDEDDLERGDEITTTLEEAIKGSRIAITVFSKDYASSSFCLDELVTIFGCY‑PE‑‑‑‑‑‑‑‑‑‑‑‑‑‑‑‑‑‑‑‑‑‑‑‑‑‑‑‑‑‑‑‑‑‑‑‑‑‑‑‑‑‑‑‑‑‑‑‑‑‑‑‑‑‑‑‑‑‑‑‑‑‑‑‑‑‑‑‑‑‑‑‑YEYKFIGKIV

>K7MIT6

GEDTRHAFTGHLYKALHDKGIHTFIDDEKLQRGEQITRALMEAIQDSRVAITVLSQNYASSSFCLDELATILHCH‑QRKRLLVIPVFYKVDPSDVRHQKGSYAEALEKLETRF‑‑‑‑‑QH‑DPEKLQKWKMALKQVADLSGYHF‑KEGDGYEFKFIEKIV

>K7K3H1

GKDIRDGFLGYLTRAFHQKQIYAFIDD‑KLEKGDEIWPSLVGAIQGSSISLTIFSENYTSSRWCLEELVKILECR‑EKYRQTVIPVFYGVNPTDVRHQKGNYGEALAVLGK‑‑‑‑‑‑‑KY‑NLTTVQNWRNALKKAADLSGIKS‑FD‑YKTEVDLLGEIV

>K7L036

GDDTRKGFTHNLFASLERRGIKAYRDDHDLERGKVISVELIEAIEESMFALIILSSNYASSTWCLDELQKILECK‑K‑‑‑‑EVFPIFLGVDPSDVRHQRGSFAKAFRDHEEKF‑‑‑‑‑RE‑EKKKVETWRHALREVASYSGWDS‑KD‑‑KHEAALIETIV

>K7LF16

GEDTRGDFTSHLHAALCRNGIQTYIDY‑RIQKGYEVWPQLVKAIRESTLLLVIFSENYSSSSWCLNELVELMECK‑KQEDVH‑‑‑‑‑‑‑‑‑‑‑‑VRKQSGSYHTALAKHKKDWKVSK‑‑‑‑‑‑EKMQKWKDALFEAANLSGFHS‑QT‑YRTEPDLIEDII

>I1MQH8

GEDTRYGFTGNLYKALCDKGIHTFFDEDKLHSGDDITPALSKAIQESRIAITVLSQNYASSSFCLDELVTILHCK‑R‑EGLLVIPVFHNVDPSAVRHLKGSYGEAMAKHQKRF‑‑‑‑‑KA‑KKEKLQKWRMALHQVADLSGYHF‑KDGDAYEYKFIGNIV

>K7N1L2

GEDTRMNFTSHLHEALKQKKVETYIDY‑QLEKGDEISPALIKAIEDSHVSIVILSENYASSKWCLEELSKILECK‑KKQGQIVIPVFHNIDPSHVRKQNGSYEKAFAKHEGEA‑‑‑‑‑‑‑‑‑‑‑KCNKWKATLTEVANLAGWDS‑R‑‑NRTESELLKDIV

>I1MKU1

GDDTRKGFTGHLFASLERRGIKTFKDDHDLQRGKLISVELMKAIEGSMLALIILSPNYASSTWCLDELKKILECK‑K‑‑‑‑EVFPIFHGVDPSDVRHQRGSFAKAFSEHEEKF‑‑‑‑‑RE‑DKKKLERWRHALREVASYSGWDS‑KE‑‑QHEATLIETIV

>K7L9Q3

GEDTRKTFTSHLHAAFKRMEINTYIDY‑NLERGDEISGTLLRAIEDAKLSVIVFSKNFGTSKWCLDEVKKIMECK‑KTRRQMVVPVFYDIEPTHVRNQTGSFASAFARHEERFMDRP‑‑‑‑‑‑NKVQKWKDALREATNLSGWDC‑SV‑DRLESEIVEEIA

>K7K1U0

GEDTRLGFTGHLYHALCEVGVNTFMDDQGLRKGEEITPFLMKAIQESRIAIVIFSENYASSTFCLQELVMIMECL‑KHQGRLVWPVFYKVDPSDVRHQKGSYAEALAKHETRI‑‑‑‑‑S‑‑DKDKVEKWRLALQKAASLSGWHS‑NR‑‑RYEYDIIRDIV

>K7KDI2

GEDTRATFTSHLYAALQNAGIIVFKDDESLPRGDQISDSLLLAIEQSQISVVVFSTNYADSRWCLQELEKIMNCK‑RTIGQVVLPVFYDVDPSQVRYQTGHFGESFQNLSNRI‑‑‑‑DDEKE‑VLISRWRKVLREAASIAGVVVLN‑‑SRNESETIKNIV

>K7KW83

GEDTPNNFTGFLFNALRKKGIDAFRDDTDIKKGESIAPELLQAIEGSRIFVVVFSKSYASSTWCLCELAKICK‑YIDTSERHVLPVFYDVDPSEVGKQSGYYEKAFAEHEETFGED‑‑KE‑KIEEVPGWREALTRVTNLSGWDI‑G‑‑NKPQYAKVEEIV

>Q8H6S7

GEDTRFAFTGHLHKALCNKGIRAFMDENDIKRGDEIRATLEEAIKGSRIAITVFSKDYASSSFCLDELATILGCY‑REKTLLVIPVFYKVDPSDVRRLQGSYAEGLARLEERFHPN‑‑‑‑‑‑‑‑‑MENWKKALQKVAELAGHHF‑KDGAGYEFKFIRKIV

>K7K1J3

GEDTRDNFIRHIYEQLQRKKIETYIDY‑RLSRGQEISPALHRAIEESMIYVVVFSENYASSTWCLDELTKILDCK‑KRYGRVVIPVFYKVDPSIVRNQRETYAEAFVKHEHRFQDKF‑‑‑‑‑‑DKVHGWKAALTEAAGLSGWDS‑QV‑TRPEATLVAEIV

>K7MWR9

GKDTRHSFTGNLYKALSERGINTFIDDKKLPRGDEITSALEKAIEESRIFIIVLSENYAWSSFCLNELDYILKFI‑KGKGLLVLPVFYKVDPSDVRNHTGSFGESLAYHEKKFKST‑‑N‑‑NMEKLETWKMALNQVANLSGYHF‑KHGEEYEYQFIQRIV

>K7KXK8

GLDTRNNFAALLLQALHRNGIDAFNDNVHVMKGEFIESELYMAIDGSRNFIVVFTKNYASSTWCLHELARICMN‑IETSTRRILPIFYVVDPLKVQKQSGCYEKAFMDYEERFRGA‑‑KE‑R‑EQVWRWRKGLKQVSHLPCLHI‑Q‑‑NDLQQAEIEEIL

>K7K360

GKDTRASFTSHLYAALKNAGITVFKDDETLSRGKHISHSLRLGIEQSRISVVVFSRNYAESRWCLQELEKIMECH‑RTTGQVVLPVFYDVDPSQVRHQKSHFGKAFEKLLNTI‑‑‑‑IGDKNDDLIQSWKDALHKAAGISGVVVQN‑‑FKNESEAIKHIV

>I1J5H4

GTDTRDTFTMSLYHALHRRGLRVFRDDDGLERGDEIQKKLLEAIEDSAAAVVVLSPDYASSHWCLDELAKI‑‑C‑‑K‑CGRLILPVFYWVDPSHVRKQKG‑FEDSFGSHANKFP‑‑‑‑‑‑‑‑EESVQQWRDAMKKVGGIAGYVLEKD‑SEKSDKLIQHLV

>K7LV88

GEDTRNSFTGFLFQALSRKGIDAFKDGKDLKKGESIAPELIQAIQGSRLFIVVFSNNYAFST‑‑‑‑‑‑‑‑‑‑‑‑‑‑‑‑‑‑‑‑‑‑‑‑‑‑‑‑‑‑‑‑‑‑‑‑‑‑‑‑‑‑‑‑‑‑‑‑‑‑‑‑‑‑‑‑‑‑‑‑‑‑‑‑‑‑‑‑‑‑‑‑‑‑‑‑‑‑‑‑‑I‑R‑‑KKLQYAEIEDL‑

>K7KA46

GSDTRFGFTGNLYKALHDRGFQTFIDDEWLSMRKAIISNM‑DALHFTDLLL‑‑‑‑‑HY‑‑‑‑FCMIEF‑‑‑‑‑‑‑‑‑‑‑‑‑‑‑‑‑‑‑‑‑‑‑‑‑‑‑‑‑‑‑‑QLSEDL‑‑‑‑‑‑‑‑‑‑‑‑‑‑‑‑‑‑‑‑‑‑‑‑‑‑‑‑‑‑‑‑‑‑‑‑KF‑KRSNGYESKFIERIV

>K7N1K4

GEDTRMNFTSHLHEALKQKKVETYIDY‑QLEKGDEISPALIKAIEDSHVSIVILSENYASSKWCLEELSKILECK‑KKQGQIVIPVFHNIDPSHVRKQNGSYEKAFAKHEGEA‑‑‑‑‑‑‑‑‑‑‑KCNKWKATLTEVANLAGWDS‑R‑‑NRTESELLKDIV

>D6PT14

GADTRHGFTGNLYKALDDRGIYTFIDDEELQSGEEITPALLKAIQESRIAITVLSINYASSSFCLDELAYILECF‑KSKNLLVVPVFYNVDPSDVRHQKGSYGEALAKHQERF‑‑‑‑‑NH‑NMEKLEYWKKALHQVANLSGFHF‑KHGEGYEYEFIGRIV

>K7K1I5

GEDTRKIITSHLYHALFQAELATYIDY‑RLQKGDEISQALIEAIEESQVSVIIFSEKYATSKWCLDEITKIIECK‑EGQGQVVIPVFYKIDPSHIRKQQGSFKQAFVEHEQDLKITT‑‑‑‑‑‑DRVQKWREALTKAANLAGWDF‑QT‑YRTEAEFIKDIV

>K7L0Z9

GDDTRSDFASHLHAALRRNNVDTYIDY‑RIEKGAKIWLEIERAIKDSTLFLVIFSENYASSSWCLNELLQLMQCK‑KQENVHVIPVFYKIDPSQVRKQSENYHVAFAKHKKDGKVSE‑‑‑‑‑‑EKMQKWKDALSEAANLSGFHS‑NT‑YRTEPDLIEDII

>K7MH08

GEDTRLGFTGHLYKALHDKGIRTFIDDAELQRGEEITPALMKAIQDSRVAITVLSEDYASSSFCLDELATIL‑‑D‑QRKRLMVIPVFYKVDPSDVRNQRGSYEDALAKLEGKF‑‑‑‑‑QH‑DPEKLQKWKMALKQVANLSGYHF‑KEGDGYEFEFIEKIV

>K7KCW2

GEDIRHGFLGYLTEAFHQKQIHAFIDD‑KLEKGDEIWPSLVGAIQGSLISLTIFSENYSSSRWCLEELVKIIECR‑ETYGQTVIPVFYHVNPTDVRHQKGSYEKALSEHEK‑‑‑‑‑‑‑KY‑NLTTVQNWRHALKKAADLSGIKS‑FD‑YKTEVELLGEIV

>K7N1K8

GEDTRMNFTSHLHEALKQKKVETYIDY‑QLEKGDEISPALIKAIEDSHVSIVILSENYASSKWCLEELSKILECK‑KKQGQIVIPVFHNIDPSHVRKQNGSYEKAFAKHEGEA‑‑‑‑‑‑‑‑‑‑‑KCNKWKATLTEVANLAGWDS‑R‑‑NRTESELLKDIV

>K7L7M1

GEDTRGDFTSHLHAALGRSSIETYIDY‑RIQKGEEVWVELVKAIKGSTLFLVIFSENYANSSWCLNELVELMECR‑KQEEVHVIPVFYKIDPSQVRKQTGSYRAAVAN‑‑‑‑‑‑‑‑‑‑‑‑‑‑‑‑‑‑QKWKDALYEAANLSGFHS‑HT‑YRTETDLIEDII

>K7LWN5

G‑GTRYGFTNRLYNALRQKGIYTFRDTEELRIGADIRPALLKAIENSRMSMVVLCEDYASSTWCLDELAKIIQCY‑HNKPKQVLLIFYKVQPSDVWDQKNSYAKAMADHENRF‑‑‑‑‑AK‑QPEKVKNWRKALSQLRHLTREYC‑KD‑DGYEAELIKKIV

>K7MBP2

GKDIRDGFLSHLTDTFLRKKINVFVDETNLKKGDEIWPSLAVAIEVSSISLIIFSQDYASSRWCLEELVKILECR‑EKYGRIVIPIFYHVQPKNVRHQLGSYENIFAQRGR‑‑‑‑‑‑‑KY‑K‑TKVQIWKDALNISADLSGVES‑SR‑FQNDAELIQEIV

>K7KDW6

GEDIRKNFVSHLHSALLHAEVKTFLDDENLLKGMKS‑EELIRAIEGSQIAVVVFSKTYTESSLCLRELEKIIESH‑ETRGQRVLPIFYEVDPSDVRQQKGDFGEALKAAAQK‑‑‑‑‑GEHL‑ESGLSRWSQAITKAANLPGWDESN‑‑HENDAELVEGII

>K7K6E2

GTDVRKGLLSHLKTELRRRQIDAYVD‑‑RLDRGDEISSSLLRAIEESQISLVIFSKDYASSQWCLEELAKMIES‑‑E‑NKQIVLPVFFNVDPSHVRHQCGDYGDALAKHEEKL‑‑‑‑‑KE‑NMLKVKTWRSAMKKAADLSGFHY‑‑PTNEDESDLVHGIV

>I1J777

GKDTRASFTSHLYAALKNAGITVFKDDETLSRGKHISHSLRLGIEQSRISVVVFSRNYAESRWCLQELEKIMECH‑RTTGQVVLPVFYDVDPSQVRHQKSHFGKAFEKLLNTI‑‑‑‑IGDKNDDLIQSWKDALHKAAGIS‑‑‑‑‑‑‑‑‑‑NESEAIKHIV

>I1MNC5

GEDTRYSFTGNLYNVLRERGIHTFIDDDEFQKGDQITSALEEAIEKSKIFIIVLSENYASSSFCLNELTHILNFT‑KGKNLLVLPVFYIVDPSDVRHHRGSFGEALANHEKKLNSD‑‑‑‑‑NMENLETWKMALHQVSNISGHHF‑QHGNKYEYKFIKEIV

>K7KB13

GEDTRLDFTDHLYAALVRKGIIAFRDDKQLEKGDAIAEELPKAIEESLGAIVILSENYASSSWCLDELNKILESN‑RVLGREVFPVFYGVSPGEVQHQKTQFYEAFKKHERRS‑‑‑‑‑GK‑DTEKVQKWRDSLKELGQIPGWES‑KH‑YQHQTELIENIV

>K7KBB2

GEDTRHKFIGHLRKELCQKGIKVFSDDKDLRIGEGISPALSSAIEKSKILIVVFSENYAESTWCLDELVKILECT‑KDKKQLVFPIFYHVDPSDIRHQKKSYGEHMLEHQKRF‑‑‑‑‑GK‑DSQRVQAWRSALSEASNFPGHHI‑ST‑‑GYETEFIEKIA

>K7MIT8

GEDTRSAFTGHLYNTLQSKGIHTFIDDEKLQRGEQITPALMKAIEDSRVAITVLSEHYASSSFCLDELATILHCD‑QRKRLLVIPVFYKVDPSDVRHQKGSYGEALAKLERRF‑‑‑‑‑QH‑DPEKLQNWKMALQRVADLSGYHF‑KEGEGYEYKFIEKIV

>K7KD09

GDDTRASFTSHLYTALHNAGISVFKDDETLPRGNKISTSLGLAIEESRLYVVVFSKNYAGSLWCLQELEKIMECH‑KATGQVVVPVFYDVDPSEVRHQTGHFGQAFRNLAYIN‑‑‑‑EEEMNEVLVIHWKEALHEAAGISGIVVLN‑‑SRNESEAIKTIV

>K7LWN3

GEDTRRNFVCHLHSVLSNAGVNTFLDDENLVKGMEL‑IQLMRAIEGSQISLVVFSKNYTQSTWCLTELENIIKCH‑RLHGHVVVPIFYHVSPSDVRRQEGDFGKALNASAEK‑‑‑‑‑‑‑ED‑KYVLSRWGSALTTAANFCGWDVMK‑‑PGNEAKLVKEIV

>K7L9W3

GEDTRRSFTSHLYESLNEVKVQTYIDD‑RLEKGEEISPTLTKAIENSRVSIVIFSENYASSKWCLGELIKIMESK‑KEKGQIVIPVFYNIDPSHVRKQTGSYEQAFEKHEGEP‑‑‑‑‑‑‑‑‑‑‑RCNKWKTALTEAAGLAGFDS‑RN‑YRTDPELLKDIV

>I1MM77

GEDTRGKFVSHLHYALSKAGVNTFIDDENLLKGMTLKDELMRAIEGSQISLVVFSKSYTESTWCLDELEKILECR‑KLHDQIVMPIFYDIEPSVVRHQKGAFGKALKSAVEK‑‑‑‑‑GEHA‑EQVLWRWSSALNRAADLSGFHVVD‑‑RRNEAILVKEIV

>I1KEB3

GEDTRNSFTGFLFEALKKQGIEAFKDDKDIRKGESIAPELIRAIEGSHVFLVVFSKDYASSTWCLRELAHIWDC‑IQKSPRHLLPIFYDVDPSQVRKQSGDYEKAFAQHQQRFED‑‑‑‑‑‑‑‑KEIKTWREVLNDVGNLSGWDI‑K‑‑NKQQHAVIEEIV

>K7LWN7

GIDTRHSFTDNLYNSLKQRGIHAFIDDEGLRRGEEITPTLLKAIRESRIGIIVFSKSYASSTYCLDELVEILECL‑KE‑GRLVWPVFYDVDPSQVRYQTGTYAEALAKHKERFQDD‑‑‑‑‑K‑GKVQKWRKALHEAANLSGWHF‑QHGSESEYKFIKKIV

>K7N1C8

GEDTRHTFTCKLYDALWLKGIDTFMDNKELKNGDKIGPTLHKAIEEARISVVVLSENYADSSWCLDELVKIHECM‑ESKNQLVWPIFYKVNPSDVRHQKGSYGVAMTKHETSP‑‑‑‑‑GI‑DLEKVHKWRSTLNEIANLKGKYL‑EE‑GRDESKFIDDLA

>K7MIX4

GLDTRNGFTGNLYKALGDRGIYTFIDDQELPRGDKITPALSNAINESRIAITVLSENYAFSSFCLDELVTILHCK‑S‑EGLLVIPVFYKVDPSDVRHQKGSYGETMTKHQKRF‑‑‑‑‑ES‑KMEKLREWRMALQQVADLSGYHF‑KDGDSYEYKFIGNIV

>K7MIX2

GEDTRYGFTGNLYKALCDKGIHTFFDEDKLHSGEEITPALLKAIQDSRIAITVLSEDFASSSFCLDELATILFCA‑QYNGMMVIPVFYKVYPCDVRHQKGTYGEALAKHKKRF‑‑‑‑‑P‑‑‑‑DKLQKWERALRQVANLSGLHF‑KDRDEYEYKFIGRIV

>I1MQE7

GEDTRHAFTGHLYKALHDKGIHTFIDDEKLQRGEQITRALMEAIQDSRVAITVLSQNYASSSFCLDELATILHCH‑QRKRLLVIPVFYKVDPSDVRHQKGSYAEALEKLETRF‑‑‑‑‑QH‑DPEKLQKWKMALKQVADLSGYHF‑KEGDGYEFKFIEKIV

>K7KLL5

GSDTRQGFAANLYKALANRGIYTSIDDEELQSGEEITPTLLKAIEESRISMAVLSVNYASSSFCLDELATIFDCA‑ERKALLVF‑‑‑YKVEPSHVRHRKVSYGEALAKKEERF‑‑‑‑‑KH‑NMDKLPKWKMPFYQAANLSGYHF‑KDGYAHEYEFIGRMV

>K7KDG9

GEDTRASFTSHLYTALLNAGIIVFKDDESLLRGDQIAPSLRLAIEQSRISVVVFSRNYAESRWCLDELEKIMECH‑RTIGQVVVPVFYDVDPSEVRHQTGEFGRTFEKLSDRI‑‑‑‑KQEV‑‑‑‑VQSWKEALREAAGISGVVVLN‑‑SRNESEAIKSIV

>K7MIY4

REDTHRGFTFYLYKALNDRGIYTFFYDQELPRETEVTPGLYKAILASRVAIIVLSENYAFSSFCLDELVTILHCE‑R‑E‑‑‑VIPVFHNVDPSDVRHQKGSYGEAMAKHQKRF‑‑‑‑‑KA‑K‑‑KLQKWRMALKQVANLCGYHF‑KDGGSYEYMLIGRIV

>K7MIV1

GSDTRYGFTGNLYNALSDRGIHTFIDEEELQRGDEIRPALVEAIKQSRMAILVFSKNYASSSFCLDELVKIMECV‑KAKGRLIFPIFYDVDPCHVRHQSGSYGEALAMHEERFTSS‑‑KENNMERLQKWKMALNQAADVSGKHY‑KLGNEYEHEFIGKIV

>K7KXS1

GIGTRNSFTDHLFAALQGITKISMKENSNMSSSKQLKDPI‑‑‑‑‑‑‑‑‑‑‑‑‑‑‑‑DYASSTWCMKELTKIVDW‑VQETGPSVLPVFYDVTPSEVRKQSGQFGEAFTEHEER‑‑‑‑‑‑‑‑‑‑‑‑‑‑‑‑‑‑‑‑‑‑‑‑‑‑‑‑‑‑‑‑‑‑‑‑‑‑PQNEEIEKIV

>K7KCW5

GEDIRHGFLGYLTEAFHQKQIHAFIDD‑KLEKGDEIWPSLVGAIQGSLISLTIFSENYSSSRWCLEELVKIIECR‑ETYGQTVIPVFYHVNPTDVRHQKGSYEKALSEHEK‑‑‑‑‑‑‑KY‑NLTTVQNWRHALKKAADLSGIKS‑FD‑YKTEVELLGEIV

>K7LX77

GEDTRKNFTSHLYEALKQKKIETYIDY‑RLEKGDEISAALIKAIEDSHVSVVIFSENYASSKWCLGELGKIMECK‑KERGQIVIPVFYNIDPSHVRKQTGSYEQSFAKHTGEP‑‑‑‑‑‑‑‑‑‑‑RCSKWKAALTEAANLAAWDS‑QI‑YRTESEFLKDIV

>C6THX9

TEDTGKTFTSHLSGALERVDIKTYVDN‑NLERGEEIPTTLVRAIEEAKLSIIVFSKNYAASKWCLDELLKILECG‑RAKRQIIVPVFYDIDPSDVRSQRGTYAEAFAKHERNFNEKK‑‑‑‑‑‑‑KVLEWKNGLVEAANYAGWDC‑KV‑NRTEFEIVEEIV

>K7KCV0

GSDIRLGFLSHLSKAFHQKQIHAFVDD‑KLQRGDEISQSLLEAIEGSSISLIIFSEDYASSRWCLEELVKIVECR‑EEYGQIVIPVFYNVDPTNVRHQKGSFETALAEHEK‑‑‑‑‑‑‑KY‑DLPIVRMWRRALKNSANLAGINS‑TN‑FRNDAELLEDII

>K7MWR5

GEDTRHSFTGNLYKALSDRGIHTFIDDKKLPRGDQISSALEKAIEESRIFIIVLSENYASSSFCLNELGYILKFI‑KGKGLLVLPVFYKVDPSDVRNHAGSFGESLAHHEKKFNAD‑‑KETNLVKLETWKMALHQVANLSGYHF‑KHGEEYEYKFIQRIV

>K7K5P7

GTDIRSGVLSHLIAALSNAGVNTF‑EDEKFERGERIMPSLLRAIAGSKIHIILFSNNYASSKWCLDELVKIMECH‑RTYGNEVLPVFYNVDPSDVRNQRGDFGQGLEALAQR‑‑‑‑‑LQGE‑NDVLKSWKSALNEAANLAGWVSRN‑‑YRTDADLVEDIV

>K7N1C6

GEDTRHTFTCKLYDALWLKGIDTFMDNKELKNGDKIGPTLHKAIEEARISVVVLSENYADSSWCLDELVKIHECM‑ESKNQLVWPIFYKVNPSDVRHQKGSYGVAMTKHETSP‑‑‑‑‑GI‑DLEKVHKWRSTLNEIANLKGKYL‑EE‑GRDESKFIDDLA

>I1KE93

GEDTRNSFTAFLFEALKKQGIEAFKDDKDIRKGESIAPELIRAIEGSHVFLVVFSKDYASSTWCLRELAHIWNC‑IQTSPRLLLPIFYDVDPSQVRKQSGDYEKAFAQHQQRFQD‑‑‑‑‑‑‑‑KEIKTWREVLNHVASLSGWDI‑R‑‑NKQQHAVIEEIV

>I1N6X6

GEDTRHSFTGNLYKALSDRGIHTFIDDKKLPRGDQISSALEKAIEESRIFIIVLSENYASSSFCLNELGYILKFI‑KGKGLLVLPVFYKVDPSDVRNHAGSFGESLAHHEKKFNAD‑‑KETNLVKLETWKMALHQVANLSGYHF‑KHGEEYEYKFIQRIV

>I1MD01

GTDVRCGFLSHLKKELRQKQVDAFVDD‑RLEGGDEISHSLDKAIEGSLISLVIFSKDYASSKWCLEEVVKIIECM‑HSNKQIVIPVFYNVDPSDVRHQKGTYGDAFAKHEKN‑‑‑‑‑‑KR‑NLAKVPNWRCALNIAANLSGFHS‑SK‑FVDEVELIEEIA

>K7MH86

GEDTRYGFTGNLYNVLRERGIHTFIDDQELQKGDQITKALEEAIEKSKIFIIVLSENYASSSFCLNELTHILNFT‑KGKNVLVLPVFYKVDPSDVRKHRGSFGEALANHEKKLNSN‑‑‑‑‑NMEKLETWKMALHQVSNISGHHF‑QHGDKYEYKFIKEIV

>I1MND5

REDTRHGFTGNLYNVLRERGIHTFIDDDEPQKADQITKALEEAIKNSKIFIIVLSENYASSFFCLNELTHILNFT‑KGWDVLVLPVFYKVDPSDVRHHRGSFGEALANHEKNLNSN‑‑‑‑‑YMGKLKTWKMALRQVSNFSGHHF‑QPGNKYEYKFIKEIL

>K7MEG0

GADIRQGFLSHLIEAFSRKHIAAFVDH‑NILKGDELSETLLGAINGSLISLIIFSQNYASSRWCLLELVKIVECR‑KRDGQIVVPVFYKVDPSDVRHQKGTYGDAFAKHEG‑‑‑‑‑‑‑KF‑SLTTIQTWRSALNESANLSGFHS‑ST‑FGDEAELVKEIV

>K7KD11

GEDTRASFTSHLYTALHNEGVFVFKNDETLPRGNQISPSLRLAIEESRISVVVFSTNYAESRWCLKMLENIMECQ‑RTTGQVVVPVFYGVYPSKVRHQTGDFGKAFRNLENRL‑‑‑‑‑‑‑‑‑‑‑‑‑‑‑‑‑‑‑‑‑‑‑‑‑‑‑‑‑‑‑‑‑‑‑‑‑NESEAIQTIV

>K7KXC3

GEDTRNSFTGFLLQALKKEGIEAFKDDKDIRKGESIAPELIRAIEGSHVFLVVFSKDYASSTWCLRELAHIRNC‑IQTSPRHLLPIFYDVDPSQVRKQSGDYQKAFAQHQQRFQE‑‑‑‑‑‑‑‑KEINIWREVLELVANLSGWDI‑R‑‑YKQQHAVIEEIV

>K7K3H2

GEDIRHSFLGYLTEAFYQKQINAFVDD‑KLEKGDEIWPSLVGAIQGSSISLTIFSENYTSSRWCLDELVKILECR‑EKYGQIVIPVFYGVNPTDVRHQKGSYGEALAQLGK‑‑‑‑‑‑‑KY‑NLTTVQNWRNALKKVADLSGIKS‑FD‑YKTEVELLGEIV

>K7MRG1

GEDTRRNFTSHLYEALKQKKVETYIDE‑HLEKGDEISPALIKAIEDSHVSIVVFSKNYASSKWCLVELIKILDCK‑KDRGQIVIPVFYEIDPSDVRKQTGSYEQAFAKHEGEP‑‑‑‑‑‑‑‑‑‑‑SCNKWKTALTEAANLAGWDS‑RT‑YRTDPELLKDIV

>K7N0U7

GEDTRNNFTSHLYAAFQLNKIQAFIDN‑RLHKGDEISPSIFKAIKHCNLSVVVLSKHYASSTWCLRELAEILDHK‑KRGGHIVIPVFYKIDPSHVRKQTGTYGKAFEKYERDVKHNM‑‑‑‑‑‑AMLQKWKAALTEVANLVGWEF‑KN‑HRTENELIEGIV

>I1LC91

GEDVRTSFISHLRSALSRDNIKAYMDH‑NLQKGDELWPSLCQAIQDSELAIVVFSEHYAASKWCLNELVEILHCR‑KSQGLAVIPVFYEVDPSHIRKYDGTCGEAISKYETYFGDKD‑‑‑‑‑‑ESIQKWKAALAEAAHISGWDSSRE‑YKNDSQLIEKIV

>K7KDV8

GEDLRKNFISHLSYALSKAGINTVLDGQQ‑‑‑‑MEL‑EELMKP‑EKSQISIVVFSKSYTESTWCLDELAKIIEIH‑ETYGQRVVVVFYEIDPSHVRDQKGDFGKGLKAAARK‑‑‑‑‑EEHL‑ESGLSRWSQALTKAANFSGLDLKN‑‑CRDEAELVKQIV

>K7KDW5

GEDIRKNFVSHLHSALLHAEVKTFLDDENLLKGMKS‑EELIRAIEGSQIAVVVFSKTYTESSLCLRELEKIIESH‑ETRGQRVLPIFYEVDPSDVRQQKGDFGEALKAAAQK‑‑‑‑‑GEHL‑ESGLSRWSQAITKAANLPGWDESN‑‑HENDAELVEGII

>K7LQJ9

GEDTRFGFTGHLYNTLRHRGINTFMDDEALERGEQISEAIFKAIEESGKAIVVFSKNYASSTWCLEELVKILSCM‑KTKELKVYPLFYNVDPSEVRYQRASYGQQLAKHEIKM‑‑‑‑‑KY‑SKQKVQNWRLALHEAANLVGWHF‑KDGHGYEYEFITRIV

>K7KDV7

GEDTRRSFVCHLNCALSKAGVKTFLDEENLHKGMKL‑DELMTAIEGSQIAIVVFSKSYTESTWCLRELEKVIECN‑ETYGQSVLPVFYNIDPSVVRHEKHDFGKVLKSTAEK‑‑‑‑‑GEHL‑ENALSRWSRALSEASKFSGWDASK‑‑FRNDAELVEKIV

>K7K1I4

GEDTRKIITSHLYHALFQAELATYIDY‑RLQKGDEISQALIEAIEESQVSVIIFSEKYATSKWCLDEITKIIECK‑EGQGQVVIPVFYKIDPSHIRKQQGSFKQAFVEHEQDLKITT‑‑‑‑‑‑DRVQKWREALTKAANLAGWDF‑QT‑YRTEAEFIKDIV

>K7LEA6

GSDTRHGFTGHLHKALHDSGIHAFIDDHDLMRGEEITPALKEAIEKSNVAITMLSEDYASSSFCLYELDYILECR‑RRKDLLVLPVFYKVSPSHVEHQTGCYGEALAKLNEKF‑‑‑‑‑QP‑KMDDC‑‑‑‑‑‑‑‑‑‑‑‑‑‑‑‑CI‑KTG‑‑YEHKFIGEIV

>K7LUI7

GKDTRNNFTDHLFGALQRKGILTFRDDTKLKKGERILSSLMQAIEGSQIFVIVFSKNYASSTWCLRELEKILDC‑VIVPGKRVLPIFYDVDPSEVRKQTGDYGKAFTKHEERFKDD‑‑VE‑KMEEVKRWRRALTQVANFSGWDM‑M‑‑NKSQYDEIEKIV

>K7KQN0

ATDTRSNFTDFLFQALIRKGIVAFKD‑‑ESRAPDQ‑‑‑‑‑‑‑AIEDSRLFIVVLSKNYAFSTQCLHELSQIFHC‑‑ES‑‑RRVLPIFYDVDPSDVRKQTG‑YEKAFSKYEERFLVN‑‑KKG‑METVQTWRKALTQVANLSGWYI‑R‑‑NKPQYTEIEEFV

>K7MDD0

GWDIRFSFTGFLYKGLFDHGFRTFMDDREIDKGSQIPQTLREAIEDSRVFIVVLSANFASSSFCLDEVVLILQEFAK‑KGR‑ILPVFYYVDPSHLAD‑SDSYKRALEDQTE‑‑‑‑‑‑‑‑‑‑DSQRIQIWKTALSKLATFSGLRLIRNG‑SLEYQYIELIL

>K7KDW3

GEDTRKKFVCHIYKALSNAGINTFIDEENIQKGMTL‑DELMTAIEGSQIAIVVFSKTYTESTWCLRELQKIIECH‑ENYGQRVVPVFYHIDPSHIRHQEGDFGSALNAVAER‑‑‑‑‑GEDL‑KSALSNWKRVLKKATDFSGWNERD‑‑FRNDAELVKEIV

>K7KCZ9

GEDTRASFTSHLYTALHNAGVFVFKDDETLSRGNKISPSLQLAIEESRVSVVVFSRNYAESRWCLKELEKIMECH‑RTTGQVVVPVFYDVDPSEVRHQTGHFGKAFRNLENRL‑‑‑‑EEEENKDLMMSWKEALREAAGISGIVVLN‑‑SRNESEAIKTIV

>K7KXJ1

GEDTRNNFTAFLFDALSQNCINAFKDDADLKKGESIAPELLQAIEGSRLFVVVFSKNYASSTWCLRELAHICNCTIEASPGRVLPIFYDVDPSEVRKQSAYYGIAFEEHEGRFRED‑‑KE‑KMEEVLRWREALTQVANLSGWDI‑R‑‑NKSQPAMIKEIV

>K7M4Z6

TEGTHLDFANTLCTSLQRNGISTFRYDKQKERGYLILEKLHKVIEQCLVVIVLLSENYASSTWCLDELHKILESK‑RVLGTPVFPLFYDVVPSDVRHQKNKFAEAFEEHATRP‑‑‑‑‑EE‑DKVKVQKWRESLHEVAGFSGWES‑KN‑WKKE‑ELIEEII

>Q84ZV0

GEDTRYGFTGNLYRALCEKGIHTFFDEEKLHGGDEITPALSKAIQESRIAITVLSQNYAFSSFCLDELVTILHCK‑S‑EGLLVIPVFYNVDPSDLRHQKGSYGEAMIKHQKRF‑‑‑‑‑ES‑KMEKLQKWRMALKQVADLSGHHF‑KDGDAYEYKFIGSIV

>K7KCX5

GDDIRRDFLGHLTKEFRRKQIHAFVDD‑KLKTGDELWPSFVEAIQGSLISLTILSENYASSSWSLNELVTILECR‑EKYNRIVIPVFYKVYPTDVRHQNGSYKSDFAEHEK‑‑‑‑‑‑‑KY‑NLATVQNWRHALSKAANLSGIKS‑FN‑YKTEVELLEKIV

>K7KD04

GEDTRASFTSHLYTALRNAGIFVFKDDETLPRGNKISPSLQLAIEESRVSVVVFSRNYAESRWCLKELENIMECH‑RTTGQVVVPVFYDVDPSEVRHQTGHFGKAFRNLENRL‑‑‑‑EEEENKDLMMSWKEALCEAAGISGIVVLN‑‑SKNESEAIKTIV

>K7K612

SEDTRKTFTSHLNGALERVDIKTYVDN‑NLERGEEIPITLVRAIEEAKLSVIVFSKNYADSKWCLDELLKILECG‑RTKRHIIVPVFYDIDPSDVRNQRGSYAEAFVNHERNFDE‑K‑‑‑‑‑‑‑KVLEWRNGLVEAANYAGWDCNKV‑TRTEYEIVEDFT

>K7MHF0

GEDTRYGFTGNLYKALYDKGIHTFIDE‑ELQRGDKITSTLEKAI‑‑‑‑‑‑‑‑‑‑‑QDYASSPFCLNELAYILNFI‑KGNRQLVLPVFHNVDTSHVRHHTGSF‑‑‑‑‑‑‑EQKN‑‑‑‑‑‑‑‑NVEKLDTWKMALHQAASLSGYHF‑KHGDGYEYQFINRIV

>I1MNC7

GE‑‑‑‑‑FTPSLMTTSSRKGTKS‑‑‑‑‑‑‑‑‑‑‑‑ITTALEEAIEKSKIFIIVLSENYASSSFCLNELTHILNFT‑KEKNVLVLPVFHKVNPSDVRHHRGSFGEALANHEKKLNSN‑‑‑‑‑NTEKLQTWKMALHQVSNISGYHF‑QDGNKYEYKFIKEIV

>C6ZS34

GEDTRYTFTGHLHASLTRLQVNTYIDY‑NLQRGEEISSSLLRAIEEAKLSVVVFSKNYGNSKWCLDELLKILECK‑NMRGQIVLPIFYDIDPSHVRNQTGTYAEAFAKHEKHLQGQM‑‑‑‑‑‑DKVQKWRVALREAANLSGWDC‑SV‑NRMESELIEKIA

>K7KZI3

GADIRQDFLSHLVEGFYRRQIHAFVDY‑KILKGDQLSEALLDAIEGSLISLIIFSENYASSHWCLFELVKIVECR‑KKDGQILLPIFYKVDPSNVRYQKGTYGDAFAKHEV‑‑‑‑‑‑‑RH‑NLTTMQTWRSALNESANLSGFHS‑ST‑FRDEAELVKEIV

>K7LUI6

GLDTRNSFTDHLFAALQRKGIVAFRDNQNINKGELLEPELLQAIEGSHVFIVVFSKDYASSTWCLKELRKIFDR‑VEETGRSVLPIFYDVTPSEVRKQSGKFGKAFAEYEERFK‑‑‑‑‑D‑DLEMVNKWRKALKAIGNRSGWDV‑Q‑‑NKPEHEEIEKIV

>K7MH83

GEDTRYGFTGNLYKVLQERGIHTFIDDEELQEGDQITTALEEAIEKSKIFIIVLSENYASSSFCLNELTHILNFT‑KENNVLVLPVFYKVDPSDVRHHRGSFGEALANHEKNLNSN‑‑‑‑‑NMEKLQIWKKALHQVSNISGYHF‑QDGNKYEYKFIKEIV

>K7M6W8

GEDTRADFTSHLHAALRRNNIDTYIDY‑RIHKGDEIWVEIMKAIKESTLFLVIFSENYASSSWCLNELIQLMEYK‑KHEDVDVIPVFYKIDPSEVRKQSGSYHMAFAKHEKDRKVTE‑‑‑‑‑‑DKMQKWKNALYEAANLSGFLS‑DA‑YRTESNMIEDII

>I1MQH7

GQDTRQGFTGYLYKALCDRGIYTFIDDQELRRGDEIKPALSNAIQESRIAITVLSQNYASSSFCLDELVTILHCK‑S‑QGLLVIPVFYKVDPSHVRHQKGSYGEAMAKHQKRF‑‑‑‑‑KA‑NKEKLQKWRMALHQVADLSGYHF‑KDGDSYEYEFIGSIV

>K7K361

GEDTRSSFTSHLYAALQNAGIIVFKDDESLPRGHHISDSLLLAIEQSQISVVVFSRNYADSRWCLKELERIMECH‑RTIGHVVVPVFYDVDPSEVRHQTSHFGNAFQNLLNRMSI‑‑SGEM‑‑‑‑‑KSWREALREAASISGVVVLD‑‑SRNESEAIKNIV

>K7MWS0

GKDTRHSFTGNLYKALSERGINTFIDDKKLPRGDEITSALEKAIEESRIFIIVLSENYAWSSFCLNELDYILKFI‑KGKGLLVLPVFYKVDPSDVRNHTGSFGESLAYHEKKFKST‑‑N‑‑NMEKLETWKMALNQVANLSGYHF‑KHGEEYEYQFIQRIV

>I1KEV5

GKDTRNNFTDHLFGAFHRKKIRTFRDDTRLKKGERILSNLMQAIEGSQIFVIVFSKNYAFSSWCLKELAKILDC‑VRVSGKHVLPIFYDVDPSEVRNQTGDYEKAFAKHEDR‑‑‑‑‑‑‑E‑KMEEVKRWREALTQVANLAGWDM‑R‑‑NKSQYAEIEKIV

>K7MIT9

GEDTRSAFTGHLYNTLQSKGIHTFIDDEKLQRGEQITPALMKAIEDSRVAITVLSEHYASSSFCLDELATILHCD‑QRKRLLVIPVFYKVDPSDVRHQKGSYGEALAKLERRF‑‑‑‑‑QH‑DPEKLQNWKMALQRVADLSGYHF‑KEGEGYEYKFIEKIV

>K7KXC2

GEDTRNSFTGFLLQALKKEGIEAFKDDKDIRKGESIAPELIRAIEGSHVFLVVFSKDYASSTWCLRELAHIRNC‑IQTSPRHLLPIFYDVDPSQVRKQSGDYQKAFAQHQQRFQE‑‑‑‑‑‑‑‑KEINIWREVLELVANLSGWDI‑R‑‑YKQQHAVIEEIV

>K7MWR8

GEDTRRGFTGNLYKALSDRGIHTFMDDKKIPRGDQITSGLEKAIEESRIFIIVLSENYASSSFCLNELDYILKFI‑KGKGILILPVFYKVDPSDVRNHTGSFGKALTNHEKKFKST‑‑N‑‑DMEKLETWKMALNKVANLSGYHF‑KHGEEYEYEFIQRIV

>I1MM78

GEDTRKTFVSHLYAALSNAGINTFID‑HKLRKGTELGEELLAVIKGSRISIVVFSANYASSTWCLHELVEIIYHR‑RAYGQVVVPVFYDVDPSDVRHQTGAFGQRLKALMQK‑‑‑‑‑SKPI‑DFMFTSWKSALKEASDLVGWDARN‑‑WRSEGDLVKQIV

>I1M0S0

AEDTHQGFVGHLFKSLTDLGFVVSGDHRDLKEE‑‑‑‑‑‑‑‑‑EIECFRVFIIVFSHHYATSSSRLDKLTEIINKYGA‑EDR‑IFPFFFEVEPNHVRFQSGSFEIAFDSHAN‑‑‑‑‑‑‑‑‑‑ESECLQRWKITLKKVTDFSGWSFNRSE‑KYQYQVIEKIV

>I1KE91

GEDTRNSFTAFLFEALKKQGIEAFKDDKDIRKGESIAPELIRAIEGSHVFVVVFSKDYASSTWCLRELAHIWNC‑IQTSRRPLLPIFYDVDPSQVRKQSGDYQKAFSQHQQKFQE‑‑‑‑‑‑‑‑KEITTWRKVLEQVAGLCGWDI‑R‑‑NKQQHAVIEEIV

>K7LSQ4

RDDTHHTFTCKLYDSLCRKGIITFMDNEELKVGDQIGHKLLKAIEESRISIVVLSENYAASSWCLDELVKIHECM‑KAKNLLVWPIFYKVDPSDVRHQNGSYGEAMTEHETRF‑‑‑‑‑GK‑DSEKVHKWRLTLTDMTNLKGEHV‑QE‑GRDESKFIDDLV

>K7MHM2

GSDTRHGFTGHLYKALCDRGIHTFIDDEELQRGEEITPLLVKAIEGSRIAIPVFSKNYASSTFCLDELVHILACV‑KEKGTLVLPVFYEVDPSDVRHQRGSYKDALNSHKERFNDD‑‑QE‑‑‑‑KLQKWRNSLSQAANLAGYHF‑KHGNEYEYDFIGNIV

>K7MWR4

GEDTRHSFTGNLYKALSDRGIHTFIDDKKLPRGDQISSALEKAIEESRIFIIVLSENYASSSFCLNELGYILKFI‑KGKGLLVLPVFYKVDPSDVRNHAGSFGESLAHHEKKFNAD‑‑KETNLVKLETWKMALHQVANLSGYHF‑KHGEEYEYKFIQRIV

>I1LSM9

GEDTRNNITSFLLGSLESKGIDVFKDNEDLRKGESIAPELLQAIEVSRIFVVVFSKNYASSTWCLRELTHICNCT‑QTSPGSVLPIFYDVDPSDVRKLSGSYEEAFAKYKERFRED‑‑RE‑KMKEVQTWREALKEVGELGGWDI‑R‑‑DKSQNAEIEKIV

>I1JC09

GEDTRGNFTSHLYDALIQAKLETYIDY‑RLQKGEEISQALIEAIEESQVSVVIFSEKYGTSKWCLDEITKIMECK‑EGQGQVVIPVFYKIDPSHIRKQQGSFNKAFEEHKRDPNITN‑‑‑‑‑‑DRVQKWRSALTKAANLAGWDS‑IT‑YRTEAKFIKDIV

>K7LK72

GEDIRTTFIGHLRSALSGPNIKAYADH‑DLQKGQEIWPSLCQAIQDSHFAIVVFSENYAESKWCLKELVQILHCR‑KTQGLVVIPVFYQVDPSHIRKCTGTYGEAIAKH‑‑‑‑‑‑KD‑‑‑‑‑‑QSVQDWKAALTEAANISGWDT‑‑‑‑‑RNESQLIEKIV

>I1KE95

GEDTRNSFTAFLFEALKKQGIEAFKDDKDIRKGESIAPELIRAIEGSHVFLVVFSKDYASSTWCLRELAHIWNC‑FQPSTRHLLPIFYDVDPSQVRKLSGDYEKAFAQHQQRFQE‑‑‑‑‑‑‑‑KEITTWREVLERVASLSGWDI‑R‑‑NKEQPTVIDEIV

>K7MG09

GGDTRRNFVSHLYYALSNAGVNTFFDEENLLKGMQL‑EELSRAIEGSQIAIVVFSETYTESSWCLSELEKIVECH‑ETYGQTIVPIFYDVDPSVVRHPTGHFGDALEAAAQK‑‑‑‑‑AKDR‑EYGFSRWKIALAKAANFSGWDVKN‑‑HRNKAKLVKKIV

>K7MBH2

GKDVRGNFLSHLDEIFKRNKIYAFVDD‑KLKKGDEIWSSLVEAIEQSFILLIIFSQSYASSRWCLKELEAILECN‑KKYGRIVIPVFYHVEPADVRHQRGSYKNAFKKHEK‑‑‑‑‑‑‑RN‑K‑TKVQIWRHALKKSANIVGIET‑SK‑IRNEVELLQEIV

>I1MQI3

GGDTRYGFTGNLYRALCDKGIHTFFDEKKLHRGEEITPALLKAIQESRIAITVLSKNYASSSFCLDELVTILHCK‑S‑EGLLVIPVFYNVDPSDVRHQKGSYGVEMAKHQKRF‑‑‑‑‑KA‑KKEKLQKWRIALKQVADLCGYHF‑KDGDAYEYKFIQSIV

>K7MIY7

GTDTRYGFTGNLYKALCDKGFHTFFDEDKLHSGEEITPALLKAIQDSRVAIIVLSENYAFSSFCLDELVTIFHCK‑R‑EGLLVIPVFYKVDPSYVRHQKGSYGEAMTKHQERF‑‑‑‑‑KD‑KMEKLQEWRMALKQVADLSGSHF‑KDGGSYEYEFIGSIV

>K7KXG4

SEDTRNNFTGFLFQALSRKGIDAFKDDKDLKKGESIAPELLQAIEGSCLFVVVFSKNYASSTWCLRELAEICNC‑IETSQRRVLPIFYDVDPSEVRKQSGYFEKAFAEHEKRFKED‑‑KK‑KMQEVQGWREALKQVSDQSLW‑‑‑‑‑‑‑‑PQCAEIEEIV

>K7KDW2

GEDTRKKFVCHIYKALSNAGINTFIDEENIQKGMTL‑DELMTAIEGSQIAIVVFSKTYTESTWCLRELQKIIECH‑ENYGQRVVPVFYHIDPSHIRHQEGDFGSALNAVAER‑‑‑‑‑GEDL‑KSALSNWKRVLKKATDFSGWNERD‑‑FRNDAELVKEIV

>K7MDC7

ADDTVAGFTSTLAKSLEDQGFRVLVDHRDLKKA‑‑‑‑‑‑‑‑‑EIETVRVFIVVLSEHYAICPFRLDKLAEIVD‑‑‑‑‑LGARVLPVFYYVPTSDVRYQTGSYEVALGVHEY‑‑‑‑‑‑‑‑‑‑ERERLEKWKNTLEKVAGFGGWPLQRTG‑KYEYQYIEEIG

>I1M0Q1

GEDTRRSFTGNLYNCLEKRGIHTFIGDYDFESGEEIKASLSEAIEHSRVFVIVFSENYASSSWCLDGLVRILD‑FTEDNHRPVIPVFFDVEPSHVRHQKGIYGEALAMHERRL‑‑‑‑‑NP‑ESYKVMKWRNALRQAANLSGYAF‑KHGDGYEYKLIEKIV

>I1JM80

GEDTRKKFVCHIYKALSNAGINTFIDEENIQKGMTL‑DELMTAIEGSQIAIVVFSKTYTESTWCLRELQKIIECH‑ENYGQRVVPVFYHIDPSHIRHQEGDFGSALNAVAER‑‑‑‑‑GEDL‑KSALSNWKRVLKKATDFSGWNERD‑‑FRNDAELVKEIV

>K7MG06

GEDTRRNFVSHLYSALSNAGVNTFLDEMNYPKGEELNEGLLRTIEGCRICVVVFSTNYPASSWCLKELEKIIECH‑KTYGHIVLPIFYDVDPSDIRHQQGAFGKNLKA‑‑‑‑‑‑‑‑‑GLWG‑ESVLSRWSTVLTQAANFSGWDVSN‑‑NRNEAQFVKEIV

>I1KEE0

GEDTRNNFTAFLFDALFENGIHAFKDDTHLQKGESIAPELLLAIQGSRLFVVVFSKNYASSTWCLRELAHICNCTIEASPSRVLPIFYDVDPSEVRKQSGYYGIAFAEHEERFRED‑‑KV‑KMEEVQRWREALTQMANLSGWDI‑R‑‑NKSQPAMIKEIV

>I1MMX8

GPDVRKGLLSHLKKELCRRQIEACVD‑‑ILDRGDEISSSLLRAIEESQILLVIFSKDYASSQWCLEELAKMIEC‑‑E‑NKQILVPVFFNVDPSDVRQQHGEYGDALAKHEEKL‑‑‑‑‑KE‑NMFKVQSWRSALKKAANLSGFHY‑‑PGNDDESDLVDKIV

>I1KE99

GEDTRNSFTGFLFEALKKQGIEAFKDDKDIRKGESIAPELIRAIEGSHVFLVVFSKDYASSTWCLRELAHIWNC‑IRTSSRLLLPIFYDVDPSQVRKQSGDYEKAFSQHQQRFQE‑‑‑‑‑‑‑‑KEIKTWREVLNHVGNLSGWDI‑R‑‑NKQQHAVIEEIV

>I1MQI7

GLDTRHGFTGNLYKALDDRGIYTSIDDQELPRGDEITPALSKAIQESRIAITVLSQNYASSSFCLDELVTILHCK‑S‑EGLLVIPVFYKVDPSDVRHQKGSYGEAMAKHQKRF‑‑‑‑‑KA‑KKEKLQKWRMALKQVADLSGYHF‑EDGDAYEYKFIGSIV

>K7MIX8

GEDTRHGFTGNLYRALCDKGIHTFFDEVKLHSGDEITPALSNAIQESRIAITVLSQNYASSSFCLDELVTILHCK‑S‑EGLLVIPVFYKVDPSDVRHQKGSYREAMAKHQKGF‑‑‑‑‑KA‑KKEKLQKWRMALHQVADLSGYHF‑KDGDAYEYKFIGSIV

>G7KJ68

GSDTRYGFTGNLYKALDDKGIHTFIDNHELQRGDEITPSLLKAIEESRIFIAVFSINYASSSFCLDELVHIIHCY‑KTKGRLVLPVFFAVEPTIVRHQKGSYGEALAEHEKRFQND‑‑PK‑SMERLQGWKEALSQAANLSGYHD‑SP‑PGYEYKLIGKIV

>G7KJS0

GSDTRYGFTGNLYKDLCKKGIRTFIDDRELPGGDKITPSLFKAIEESRIFIPVLSINYASSSFCLDELVHIIHCC‑KKNGRLVLPIFYDVEPSNVRHQIGSYGKALAEHIEKFQNS‑‑TD‑NMERLQKWKSALTQTANFSGHHF‑SSRNGYEYEFIEKIV

>G7KCQ4

GEDTRFGITDHLYDALIHKSIKTYIDY‑QLNRGEDVWPALSKAIEDSYISIIVFSENFATSKWCLEELVKVLECR‑KDHGQIVIPVFYKADPSHIRNQKASYETAFAKHERELGTKD‑IS‑NKSKVLKWKAALTEAANISGWDS‑HT‑YEKESILILKIV

>G7JVS5

G‑GTRYSFTDHLYHSLLRHGINVFRDDQNINIGDEIGTSLLKAIEASRISIVVLCRDYASSTWCLDELVKIVDC‑‑YKNRKSVFVIFYKIE‑‑‑‑‑‑‑‑‑‑‑‑‑‑‑‑PSDVRF‑‑‑‑‑GK‑ESEKVKAWRLALNRVCALSGLHC‑KD‑NIYEYEFIEKIV

>G7ZUP2

GEDTRKTFTSHLNSALRRLDIKTYIDD‑NLERGDEISQALLKEIDEAKLSVIVFSKNYATSKWCLDEVVKILECR‑KYKEQIILPDFYEVDPFHVRHQLGSL‑‑‑‑‑‑‑‑‑‑‑‑‑‑‑‑‑‑‑‑‑‑‑‑‑‑‑‑‑‑‑‑‑SANHSGWDC‑SI‑NRTEAELVEEIA

>G8A1T6

GEDTRRNFVAHLNAALSNAEINTYIDD‑RIQKGTDLEPELFRAIEDSRISIVVFSENYVHSSWCLKELEQIMKCR‑VNCGQIVEPVFYHVEPSVLRHQAGDFGKALEETAKR‑‑‑‑‑GEKM‑NTVLSTWQIALTEVANISGWDTKN‑‑FKDDVELISQIV

>G7KKS8

GFDTRNGFTGHLWKALNDIGILAFIDDTEFSRGEETKPAIFKAIHVSRIAIIVFSDNYAGSKFLLEELAFIVDNF‑QSDNRFIVPVYYNIEASHVRHQSGPFEAAFVKHEERF‑‑‑‑‑HE‑NREKVLKWKTALSQVANLPGWHF‑D‑GVEYEHQFLQKIV

>G7IQ97

GIDTRNTFTGSLYNSLDQKGIHTFIDEKEIQKGEEITPSLLQAIQQSRIYIVVFSSNYASSTFCLNELVMILECS‑NR‑RRLLLPVFYDVDPSQVRHQRGAYGEALRKHEERFSDD‑‑‑‑‑K‑DKVQKWRDALCQAANISGWHF‑QHGSQPEYKFIGNIV

>G7KGI3

GEDIHHGFLGHLFKAFSQKQINVFVDD‑KLKRGNDISHSLFEAIEGSFISLIIFSENYASSRWCLEELVKIIECK‑EKYGQIVIPVFYGVDPTDVRHQKKSYENAFVELGK‑‑‑‑‑‑‑RY‑NSSEVQIWRNTLKISANLSGITS‑SS‑FRNDAELLEEII

>G7KIF6

GIDTRSGFTGHLYKALCDKGIRTFIDDKELQRGDEITPSLLKSIEHSRIAIIVFSENYATSSFCLDELVHIINYF‑KEKGRLVLPVFYGVEPSHVRHQNNKYGEALTEFEEMFQNN‑‑KE‑NMDRLQKWKIALNQVGNLSGFHF‑KK‑DAYEYEFIKKIV

>G7KIG6

GSDTRYGFTGNLYKALTNKGIHTFIDDNHLPRGSEITPSLIKAIEESRIFIPIFSTNYASSSFCLDELVHM‑‑‑‑‑‑‑‑‑‑‑‑‑‑‑‑‑‑‑SFTATRQRVASYGEALADHEKRFQND‑‑KD‑NMERLQRWKMAMRQVANLSGYHF‑S‑‑LGYEYEFIGKIV

>G7L6S8

GSDIRKNFLSHVLDALSRKGIIVFSDK‑KLITGDELS‑AIQRAIEKSLISLVIFSPNFASSHWCLDELVKIVECR‑ANYGRVLMPVFYQVYPSDVRHQNGTYRDAFAQHEQ‑‑‑‑‑‑‑KY‑SSYKVLSWRSALKQSANMSGFDS‑SL‑FSDDAKLVEEIV

>G7KJ25

DIDTLYGFTGNLYKALIDKGIKTFIDDNDLERGDESTPSLVKAIEESRILIPIFSANYASSSFCLDELVHIIHCY‑KTRGCSVLPVFYGADPTHVRHQTGSYGEHLTKHEDKFQNN‑‑KE‑NMERLKKWKMALTQAANFSGHHF‑S‑‑QGYEYELIENIV

>G7KJC7

GADTRHGFTGNLYDALCKSGVHTFKDDEELQRGGEITASLMKAIEESRIFIPVFSKNYASSSFCLDELVHIIRYS‑KSKGRLVLPVFYDIAPTHVRKQTGSIGEELAKHQEKF‑‑‑‑‑QK‑NMERLQEWKMALKEAAELSGHHF‑NAGTEYESNFIQGIV

>G7IM52

GPDTRNTFVDHLYAHLKRKGIFAFKDDQRLEKGESLSPQLLQAIQNSRVSIVVFSERYAESTWCLEEMATVAEC‑RKRLKQTVFPVFYDVDPSHVRKHIGVFKAN‑NSHTK‑‑T‑‑‑‑‑Y‑DRNKVVRWQEAMTELGNLVGFDV‑R‑‑YKPEFTEIEKIV

>G7JKN1

GKDIRDGFLGHLVKAFRQKKINVFVDN‑IIKRGDEIKHSLVEAIEGSLISLVIFSKNYSSSHWCLDELVKIIECK‑KDRGQIIIPVFYGVRSKIV‑‑‑‑‑‑‑‑‑‑LDELEK‑‑‑‑‑‑‑KD‑NFSKVEDWKLALKKSTDVAGIRL‑SE‑FRNDAELLEEIT

>G7JF24

GEDTRNNFTDHLFDTFHREGISAFRDDTNLPKGESIGPKLLCAIENSQVFVVVLSRNYAFSTSCLQELEKILEW‑VKVSKKHVLPVFYDVDPSMVRKQSGIYGEAFVKHEQRFQ‑‑‑‑‑Q‑DSQMVQRWREALIQVADLSGWDL‑H‑‑DRRQSPEIKKIV

>G7KIF1

GSDTRDGFTGHLYKALTDKGIHTFIDDCDLKRGDEITPSLIKAIEESRIFIPVFSINYASSKFCLDELVHIIHCY‑KTKGRLVLPVFYGVDPTQIRHQSGSYGEHLTKHEESFQNN‑‑KK‑NKERLHQWKLALTQAANLSGYHY‑S‑‑PGYEYKFIGKIV

>G7LI64

GEDTRSTFTAQLYQTLKKENIITYIDE‑NLNKGEEVGPALVQAIQESRMSLVVFSENYASSKWCLDELLKILECG‑KFHDQVVIPVFYRIDPSDVRHQTGSYKEPFANYQIDRKSNE‑‑‑‑‑‑DKVSQWKAALTEIANISGWDS‑RI‑YGDDSQLIEKIV

>G7KJ66

GTDTRFGFTGNLYKALSDKGIHTFIDDKELPTGDEITPSLRKSIEESRIAIIIFSKNYATSSFCLDELVHIIHCF‑REKVTKVIPVFYGTEPSHVRKLEDSYGEALAKHEVEFQND‑‑ME‑NMERLLKWKEALHQFHSWVPLFI‑SILNKYEYKFIEEIV

>G7JLU8

GNDVRDGFLGKLYEAFIRKQINIFVDY‑KLKKGDDISHSLGEAIEGSSISLVIFSENYASSHWCLEELVKIIECR‑EKYGQLVIPIFYEVDPTNVRYQKKSYENAFVKLEK‑‑‑‑‑‑‑RY‑NSSEVKIWRHTLKISANLVGFTS‑SS‑FRNDAELLEEIT

>G7LDU6

GEDTRECFTKKLYESLHKQGVRAFMDDEGLDRGDHIATTLLEAIDDSAASIVIISPNYADSHWCLDELNRI‑‑C‑‑D‑LERLIIPVFYKVDPSHVRKQLG‑FQDGFNYLEKRFANE‑‑‑‑‑‑KDKILKWRDSMLKIGGLAGFVFSSD‑DGEHDNLIRRLV

>G7K8D4

SEDTRNNFTSHLNGALKRLDIRTYIDN‑NLNSGDEISTTLVRAIEEAELSVIVFSKNYAASKFCLDELMKILECK‑RMKGKMVVPIFYDVDPTDVRNQRGSYAEAFAKHEKNSEEKI‑‑‑‑‑‑‑KVQEWRNGLMEAANYSGWDC‑NV‑NRTELELVEEIA

>G7JCP8

GQDTHNNFADHLFAALQRKGIVAFRDDSNLKKGESIAPELLHAIEASKVFIVLFSKNYASSTWCLRELEYILHC‑SQVSGTRVLPIFYDVDPSEVRHQNGSYGEALAKHEERFQ‑‑‑‑‑H‑ESEMVQRWRASLTQVANLSGWDM‑H‑‑HKPQYAEIEKIV

>G7JCM4

GEDTRFNFIDHLFAALQRKGIFAFRDDANLQKGESIPPELIRAIEGSQVFIAVLSKNYSSSTWCLRELVHILDC‑SQVSGRRVLPVFYDVDPSEVRHQKGIYGEAFSKHEQTFQ‑‑‑‑‑H‑DSHVVQSWREALTQVGNISGWDL‑R‑‑DKPQYAEIKKIV

>G7KJS7

GTDTRYGFTGNLYKALCDGGVRTFIDHKDLHEGDRITQSLVKAIEESRILIPVFSKNYASSLFCLDELVHIIHRY‑EEKGCFVFPIFCDVEPSHVRHQTGSYGEALAKHEERFQNN‑‑KENNMKRLHKWKMALNQAANLSGHHF‑NPRNGYEFEFIREIV

>G7LC17

GLDTRYGFTGNLYKALYDKGIHTFIDDEELQRGHEITPSLLEAIEESRIAIIVLSKNYASSSFCLHELVKILDCI‑KGKGRLVWPIFYDVDPSDVRKQTGSYGEALAMLGERF‑‑‑‑‑‑‑‑NDNNLQIWKNALQQVANLSGWHF‑KIGDGYEYEFIGKIV

>G7L6S0

GPDIREVFLPHLIKAFSQKKIVYFVDY‑KLTKGNEISQSLFEAIETSSISLVIFSQNYASSSWCLDELVKVVDCR‑EKDGNILLPVFYKVDPTIVRHQNGTYADAFVEHEQ‑‑‑‑‑‑‑KY‑NWTVVQRWRSALKKSANINGFHT‑SK‑RLNDAELVEEIV

>G7KF73

GQDTVE‑‑‑‑‑‑LKGFCEKGINTFIDDQELRKGEEITPALMM‑‑‑‑‑‑‑AIVIFSENYASSTFCLEALRKIME‑‑‑‑‑‑‑‑‑‑‑‑‑‑‑‑FDPSDVRHQKGSYAKAI‑‑‑‑‑‑‑‑‑‑‑‑‑‑‑‑‑‑‑VKQWRLALQEAANLVGWHF‑RHRYDYEYELIGKIV

>G7KPT3

GTDTRFGFTGNLYKALSDKGIHTFIDDKELKRGDEITPSLRKSIEDSRIAIIVFSKDYASSSFCLDELVHIIHYF‑KEKSRLVLPIFYGTEPSQVRKLNDSYGESFAKHEEGFQNN‑‑KE‑HMERLLTWKKALNEAANLSGHHF‑NQGNEYERDFIEKIV

>G7IW57

GIDTRNNFTGNLYNSLNQSGIQTFIDDEEIQKGEEITPTLLKAIKESRIFIAILSPNYASSTFCLTELVTILECS‑KK‑GRWFLPIFYDVEPTQIRNLTGTYAEAFAKHEVRFRDE‑‑‑‑‑K‑DKVQKWRDALRQAASLSGWHF‑QPGSQQEYKFIRMIV

>G7KIF2

GIDTRHGFTGNLYKALIDKGIHTFIDDNDLLRGDEITPSLVKAIEESRIFIPIFSANYASSSFCLDELVHIIHCY‑KTKGCSVLPVFYGVDPTHIRHQTGSYGEHLTKHEKKFQNN‑‑KE‑NMQRLEQWKMALTKAANLSGYHC‑S‑‑QGYEYKFIENIV

>G7L6R9

GSDIRKHFLSHVLEALSRKRIVVFSDK‑KLKTGDELS‑AIQRAIEKSFISLVIFSPNFASSYWCMEELVKIVECR‑EKYGRILMPVFYQVEPTVVRYQNGIYRDAFAQHEQ‑‑‑‑‑‑‑NY‑SSYKVLRWRSALKQSANISGFDS‑SQ‑FSDDAKLVEEIL

>G7KIH4

GTDTRFHFIGHLYKALCDCGIRTFIDDKELHGGEEITPSLVKAIEDSGIAIPVFSINYATSSFCLDELVHIVDCF‑KTKGHLILPIFYEVDPSHVRHQTGSYGAYIG‑‑‑‑‑‑‑‑‑‑‑‑‑‑NMERLRKWKIALNQAANLSGHHF‑NLGNSYEYELIGKMV

>G7KJQ3

GSDTRYSFIGNLHKDLCRKGIRTFIDDRELKGGDEITPSLFKHIEETRIFIPVLSTNYASSSFCLDELVHIIHCF‑KESSRLVLPIFYDVEPSHVRHQHGSYAKALDDHIEKFQNN‑‑KN‑NMERLQKWKSALTQTANFSGHHF‑NPRNGYEYEFIEKIV

>G7KPH8

GEDTRNNFTDFLFDALQTKGIIVFLDDTNLPKGESIGPELIRAIEGSQVFVAFFSRNYASSTWCLQELEKICEC‑IKGSGKHVLPVFYDVDPSEVRKQSEIYSEAFVKHEQRFQ‑‑‑‑‑Q‑DSKKVSIWREALEQVGDISGWDL‑H‑‑DKPLAREIKEVV

>G7LEE4

GKDTRNNFVSHLYAALTNVRINTFLDDEELGKGNELGPELLQAIQGSQMFIVVFSENYARSSWCLDELLQIMECR‑ANKGQVVMPVFYGISPSDIRQLARRFGEAFNNNT‑‑‑‑‑‑‑‑DEL‑DQLIY‑‑‑MALSDASYLAGWDMSN‑‑YSNESNTVKQIV

>G7JZM1

GDDSRNSLVSHLYAALSNARINTFLDDEKLHKGSELQPQLLRAIQGSQICLVVFSENYSRSSWCLLELEKIMENR‑GTHGQIVIPIFYHIDPAIVRRQLGNFGKALEITAKK‑‑‑‑‑REKQ‑KLLLQTWKSALSQATNLSGWDVTS‑‑SRNESELVQKIV

>G7JKL7

GKEIRSGFLSHLVKAFCQKQINAFVDD‑KLKRGDDISDSLGEAIEGSFISLIIFSENYACSHWCLKELVKIVECK‑EKYAQIVIPVFFRVDPTDIRHQKRSYENAFAEHEK‑‑‑‑‑‑‑KY‑SSYEVQMWKHALKISANLSGITS‑SS‑FQRKEKSIETL‑

>G7JKM7

GEDIRANFLSHLIEDFDRKKIKAFVDD‑KLKRGDEIPQSLVRAIEGSLISLIIFSHDYASSCWCLEELVTTLQCR‑EKYGQIVIPIFYQVDPTDVRYQNKSYDNAFVELQR‑‑‑‑‑‑‑GY‑SSTKVQIWRHALNKSANLSGIKS‑SD‑FRNDVQLLKEIV

>G7IQA8

GIDTRNNFTRDLYDILDQNGIHTFFDEQEIQKGEEITPSLLQAIQQSRIFIVVFSNNYASSTFCLNELVMILDCS‑NH‑RRLLLPVFYDVDPSQVRHQSGAYGEALKKHEERFSDD‑‑‑‑‑K‑DKVQKWRDSLCQAANVSGWHF‑QHGSQSEYQFIGNIV

>G7J146

GKDTRLNFTDHLFA‑‑‑‑‑‑‑‑‑‑‑‑‑‑‑LKKGESIAPELLRAIEDSQIFVVVFSKNYASSVWCLRELECILQS‑FQLSGKRVLPVFYDVDPSEVRYQKGCYAEALAKHEERFQ‑‑‑‑‑Q‑NFEIVQRWREALTQVANLSGWDV‑R‑‑YKPQHAEIEKIV

>G7IW71

GIDTRNNFTGNLYHSLHQRGIQTFMDDEEIQKGEEITPTLLQAIKQSRIFIAIFSPNYASSTFCLTELVTILECS‑MQ‑GRLFLPVFYDVDPSQIRNLTGTYAEAFAKHEVRFGDE‑‑‑‑‑KDSKVQKWRDALRQAANVSGWHF‑KPGFESEYKFIEKIV

>G7LGU0

GSDTRCDFSGFLNKYLIDRGFRTFFDDGELERGTQITVEIPKAIEESRIFIPVLSENYASSSFCLDELVKILEEFKK‑NGR‑VFPVFYYVNISDVKNQTGSYGQALAVHKN‑‑‑‑‑‑‑‑‑‑MPERFEKWINALASVADFRGCHMERAR‑GYEIRYIYEII

>G7L6L8

GEDTRKGFTDHLRAALERKGITTFRDDKDLERGKNISEKLINAIKDSMFAITIISPDYASSTWCLDELQMIMECS‑SNNNLHVLPVFYGVDPSDVRHQRGSFEEAFRKHLEKF‑‑‑‑‑GQ‑NSDRVERWRNAMNKVAGYSGWDS‑KG‑‑QHEALLVESIA

>G7LIX4

GDDTRAGFTSHLYADLCRSKIYTYIDY‑RIEKGDEVWVELVKAIKQSTIFLVVFSENYASSTWCLNELVEIMECC‑NKDKVVVIPVFYHVDPSHVRKQTGSYGTALIKHKKQGKNDD‑‑‑‑‑‑KMMQNWKNALFQAANLSGFHS‑TT‑YRTESEMIEAIT

>G7LEF6

GEDIGKSFVSHLVNALRKARITTYIDGGQLHTGTELGPGLLAAIETSSISIIVFSKNYTESSWCLDVLQNVMECH‑ISDGQLVVPVFHDVDPSVVRHQKGAFGQVLRDTAKR‑‑‑‑‑KGEI‑EDVVSSWKNALAEAVSIPGWNAIS‑‑FRNEDELVELIV

>G7KL77

GEDTRYGFTGNLKKALDDKGVRTFIDDEKLKKGDEITPSLLKAIEDSMMAIIVLSENYASSSFCLQELSHILDTM‑KKAGRYVLPVFYKVDPSHVRKLKRSYGEAMKKHDVAS‑‑‑‑‑SS‑SHNMNNKWKDSLHQVANLSGSHY‑K‑GDKYEYEFIENIV

>G7LF39

GADTRKTFISHLYTALTNAGINTFLDNENLQKGKELGPELIRAIQGSQIAIVVFSKNYVHSRWCLSELKQIMECK‑ANDGQVVMPVFYCITPSNIRQYATR‑‑‑‑FSETTLF‑‑‑‑‑FDEL‑VPFM‑‑‑‑NTLQDASYLSGWDLSN‑‑YSNESKVVKEIV

>G7IM43

GTDTRNTFIDHLYHHLIRKGISAIRDS‑‑‑‑‑‑‑‑‑‑‑‑‑‑‑‑‑‑‑‑‑‑‑‑‑‑‑‑‑‑‑‑‑‑‑‑‑‑‑‑‑‑‑‑‑‑‑‑‑RELNQTVIPIFYDIDPSYVRSNLSAFNGDYNSRTKKLN‑‑‑‑‑Y‑DPDKVSRWERVVIQL‑‑‑‑‑‑‑‑‑‑‑‑‑SPEFTEIEKIV

>G7KJR3

GSDTRYRFTGNLNRALCDKGIRTFMDDRELQGGEEITSSLFKAIEESRIFIPVLSINYASSSFCLDELVHIINCF‑KESGRLVLPIFYDVEPSHVRHHTGSYGKALDDHIKKFQNN‑‑KD‑SMERLQKWKSALTQTANFSGHHF‑NPGNGYEHEFIEKIV

>G7LGU9

GDDTRFSFTGFLSHSLNNRGFYTAINHRD‑‑‑‑‑‑‑‑‑‑‑‑‑SIQHCRIFIFIISRDYASR‑‑‑LDELVNIMDLFAK‑NGRRILPVYYHVNPSDVRHQSGSFGEALSSFYN‑‑‑‑‑‑‑‑‑‑LVSDFEKRNTVLRQVADFRGWHLDPAR‑GYEHQYIEEIL

>G7KIG7

GSDTRFGFTGHLYKALCDSGIHTFIDDTELHRGDEISPSLIKAIEESMIYIPVLSINYASSIFCLEELVKIIKSF‑‑HSGHHILPVFYDVHPSQVRSRTGSFGEAIDKHKEK‑‑‑‑‑‑‑‑‑GTSRVYEWNNALIQVANLSGYHW‑SDGNKYEHEIIGMIV

>Q2HUD1

GEDTRKGFTDHLCAALERKGITTFKDDKDLERGQVISEKLINAIKDSMFAITILSPDYASSTWCLDELQMIMECS‑SKNNLHVLPVFYGVDPSDVRHQRGCFEEAFRKHQEKF‑‑‑‑‑GQ‑HSDRVDRWRDAFTQVASYSGWDS‑KG‑‑QHEASLVENIA

>G7LGT8

GSDTRCGFSGFLNKYLIDRGFRTFFDDGELERGTQITVEIPKAIEESRIFIPVLSENYASSSFCLDELVKILEEFKK‑NGR‑VFPVFYYINISDVKNQTGSYGQALAVHKN‑‑‑‑‑‑‑‑‑‑MPERFEKWINALASVADFRGCHMERAR‑GYEFRYIYEII

>G7K8C7

GEDTRYTFTSHLHATLTRLKVGTYIDY‑NLQRGDEISSTLLMAIEEAKVSIVIFSKNYGNSKWCLDELVKILECK‑KMKGQILLPIFYDIDPSHVRNQTGSYAEAFVKHEKQFQGKL‑‑‑‑‑‑EKVQTWRHALREAANISGWEC‑SV‑NRMESELLEKIA

>G7JF25

GEDTRNNFTYHLFDAFNREGILAFRDDTNLPKGESIASELLRAIEDSYIFVAVLSRNYASSIWCLQELEKILEC‑VHVSKKHVLPVFYDVDPPVVRKQSGIYCEAFVKHEQIFQ‑‑‑‑‑Q‑DSQMVLRWREALTQVAGLSGCDL‑R‑‑DKRQSPGIKNIV

>G7JCM5

GEDTRFNFIDHLFAALQRKGIFAFRDDTNLQKGESIPPELIRAIEGSQVFIAVLSKNYASSTWCLRELVHILDC‑SQVSGRRVLPVFYDVDPSEVRHQKGIYGEAFSKHEQTFQ‑‑‑‑‑H‑ESHVVQSWREALTQVGNISGWDL‑R‑‑DKPQYAEIKKIV

>G7IW33

GEDTRYGFTGHLYNALHQRGINTFMDDEEIKRGEQISPTLFKAIQESRIAIIVFSKTYASSKWCLQELVKIVECF‑KAKELVIFPVFYNVDPSEVRNQKTSYGEQLAKYEEKM‑‑‑‑‑‑‑‑‑KEEVQSWRLALHETASLAGWHF‑RDGYEYEYEFIKRIG

>G7KKS2

GDDTRYSFTGYLYNTLCQKGINTFKDDIKLKKGEEISTDLLQAIDESRIAIIVCSENYASSPWCLDELVKIMECK‑EEKGQLVCIVFFYVDPSNVRHQRKSFARSMAKHEENP‑‑‑‑‑KI‑SEEKISKWRSALSKAANLSGWHF‑K‑‑HGYEYELIQEIT

>G7LI82

GEDTRYGITSHLHAALIHKSIKTYVDS‑LLERGEDIWPTLAKAIEESHVSIVVFSENFATSTWCLEELVKVLECR‑KVKGQVVIPVFYKTDPSDIRNQTGSYENAFAKHERDLGTND‑‑‑‑‑‑LKVLNWKVALAEAATISGWHT‑QT‑HKEESILIDKIV

>G7KIF0

GTDTQFGFTGNLYKALSDKGINTFIDDKELKKGDEITPSLLKSIEESRIAIIVFSKEYASSLFCLDELVHIIHCS‑NEKGSKVIPVFYGTEPSHVRKLNDSYGEALAKHEDQFQNS‑‑KE‑NMEWLLKWKKALNQAANLSGHHF‑NLGNEYERDFIEKIV

>G7KIF5

GTDTRYGFTGNLYKALIDKGIHTFIDDNDLQRGDEITPSLIKAIEESRIFIPVFSINYASSKFCLDELVHIIHCY‑KTKGRLVLPIFFGVDPTNVRHHTCSYGEALAEHEKRFQND‑‑KD‑NMERLERWKVALSQAANLSGYHD‑SP‑PRYEYKLIGEIV

>G7IQB0

GIDTRNNFTRDLYDILDQNGIHTFFDEQEIQKGEEITPSLLQAIQQSRIFIVVFSNNYASSTFCLNELVMILECS‑NH‑GRLFLPVFYDVDPSQVRHQSGAYGDALKKHEKRFSD‑‑‑‑‑‑‑‑DKVQKWRDALCQAANVSGWDF‑QHGSQSEYKFIGNIV

>G7JDB8

G‑RTRYSFTDHLYRSLLRHGINVFRDNPNLNIGDEIRLSLLQAIEASRISIVVLCKDYASSTWCLDELVKIVDCY‑YMKGKTVFVIFYKVEASDVRHQRKSYEIAMIQHEKRF‑‑‑‑‑GK‑ESEKVKKWRSALKRVCALSGLYY‑KD‑DIYESEFIEKIV

>G7IQ90

GIDTRNNFTRDLYDSLDQNGIHTFFDEKQIQKGEQITPALFQAIQQSRIFIVVFSNNYASSTFCLNELALILDCS‑NH‑GRLLLPVFYDVDPSQVRHQSGAYGEALKKQEERFCDD‑‑‑‑‑K‑DKVQKWRDALCQAANVSGWHF‑QHGSQSEYKFIGNIV

>G7KK90

GADTRFGFTGNLYKALTDKKIRTFIDDKELQRGDEITPSLVKAIQESRIAIPIFSTNYASSSFCLDELVHIVECV‑KRKGRLVLPIFYDVDPSHVRHQTGSYGKGMTDLEERFKNN‑‑KE‑‑‑‑KLQKWKMALNQVANLAGYHF‑KLGNEYEYEFIVKIV

>G7KL58

GEDTRHGFTGNLWKALDDKGVRTFMDDENLQKGDEITPSLIKAIEDSQIAIVVLSKNYASSSFCLQELSKILDTM‑KKVGRFVMPVFYKVDPSDVRKLKGTYGDAMDKLGEAS‑‑‑‑‑SS‑SHN‑‑‑KWKDSLHQVANLSGFPY‑E‑KDGYVHEFIEKIV

>G7IQ96

GIDTRNTFTGNLYNSLDQKGIHTFLDEEEIQKGEQITRALFQAIQQSRIFIVVFSNNYASSTFCLNELAVILECS‑NH‑GRLLLPVFYDVEPSQVRHQSGAYGDALKKHEERFSDD‑‑‑‑‑K‑DKVQKWRDALCQAANVSGWHF‑QHGSQSEYKFIGNIV

>G7KKA1

GEDTRYGFTGNLWKALHDKGVRTFMDDEELQKGEEITPSLIKAIENSNMAIVVLSKNYASSSFCLKELSKILE‑‑‑‑‑VGLFVLPVFYKVDPSDVRKLEKSYGEAMDK‑‑HKA‑‑‑‑‑SS‑NLD‑‑‑KWKMSLHQVANLSGFHY‑K‑KDGYEHEFIGKIV

>G7JLT0

GDDTQCHFTSHFFSSKCR‑‑‑‑‑‑‑‑NYRLQRRSFRSKRFVHRKEGSRISIIVFSKNYADSPWCMQELIQILECY‑RTTGQVVLPVFYDVYPSDVRRQSREFGQSFQHLSNN‑‑‑‑‑‑‑‑‑‑HGASLKWIDALHDVAGIAGFVVPN‑‑YRNECEVIKDIV

>G7KJQ5

GSDTRYGFTGNLYKDLCRKRIRTFIDDKDLQRGDEITPSLFKAIEESRIFIPILSINYASSSFCLDELVHIIHCF‑KENGQV‑‑‑‑‑‑‑‑‑‑‑‑‑‑‑‑‑‑‑‑‑‑‑‑‑‑‑‑‑‑‑‑NS‑‑TD‑SMERLQKWKMALTQTANFSGHHF‑SPGNGYEYEFIEKIV

>G7KP04

GEDTRNNFTDFLFDALQTKGIIVFSDDTNLPKGESIGPELLRAIEGSQVFVAVFSINYASSTWCLQELEKICEC‑VKGSGKHVLPVFYDVDPSDVRKQSGIYGEAFIKHEQRFQ‑‑‑‑‑Q‑EHQKVSKWRDALKQVGSISGWDL‑R‑‑DKPQAGEIKKIV

>G7JLX5

GEDTRASFTSHLSTSLQSSGIIVFKDDHSLQRGHRISKTLLQAIQESRISVVVFSKNYADSQWCLQELMQIMECF‑RTTRQVVLPVFYDVHPSEVRSQTGDFGKAFQNLLNRV‑‑‑‑DEF‑‑‑‑MVPKWRDALRNAAGIAGFVVLN‑‑SRNESEVIKDIV

>G7J6M2

GEDTRRNFTSHLYEALSKK‑VITFIDN‑ELEKGDEISSALIKAIEKSSASIVIFSKDYASSKWCLNELVKILECK‑KDNGQIVIPVFYEIDPSHVRNQKGSYMLAFEKHEQDLKQSK‑‑‑‑‑‑DKLQKWKDALTEAANLAGWYS‑QN‑YKNDSIFIKYII

>G7KYW5

GEDTRRTIVSHLYTALCNAGINTFLDDKKLAKGEELGPELYTAIKMSHIFIAVFSPNYAQSSWCLNELAHIMELR‑HSYSRVVIPLFYHVDPSDVRKLKGDFGKGLKVSADKI‑‑‑‑AERE‑EVLMSKWRRALAEVTNLVGWDANN‑‑FRNEGDLVQKLV

>G7JKM5

GEDIRHGFLGHLAKM‑‑‑‑‑‑‑‑‑‑‑‑‑‑‑‑‑‑‑‑TYHSIFEAIEGSFISLIIFSENYASSRWCLEELVKIIECR‑EKNGQIVIPVFYEVGPTDVRHQKKSYENALVGHEK‑‑‑‑‑‑‑NY‑ILSRVQKWRQTLEKSANLSGIKS‑LD‑FQNDVEILEEII

>G7KL60

GEDTRHGFTGYLKKALDDKGVRTFMDAKELKKGEEITPSLLKAIEDSMMAIIVLSENYASSSFCLQELSHILDTM‑KKAGRYVLPVFYKVDPSDVRKLKRSYGEAMDKHDAAS‑‑‑‑‑SS‑SHDVNNKWKASLQQVANLSGSHY‑K‑GDEYEYEFIEKII

>G7LI75

GEDTRTNFTAQLHRALTDRSIESYIDY‑SLVKGDEVGPALAEAIKDSHMSIVVFSKDYATSKWCLDELLQILHCR‑ELFGQVVIPVFYNIDPSHVRHQKESYEMAFARYERDLVNSI‑‑S‑YVDRVSEWRAALKMAANISGWDS‑RK‑YRDDSQVIDNIV

>G7KPF0

GEDTRNNFTDFLFDALETKGIMVFRDVINLQKGECIGPELFRAIEISQVYVAIFSKNYASSTWCLQELEKICEC‑IKGSGKHVLPVFYDVDPSEVRKQSGIYSEAFVKHEQRFQ‑‑‑‑‑Q‑DSMKVSRWREALEQVGSISGWDL‑R‑‑DEPLAREIKEIV

>G7JLT1

GEDTRTSFTS‑‑‑‑‑‑‑‑‑‑‑‑‑‑‑‑‑‑‑‑‑QGDYISTSLLQGIYGSRVSVIVFSKNYAGPQWCQIQLLLVFNKK‑RVAGSIIISVI‑‑‑‑‑‑KMRILCWMCGKTRQNMITPI‑‑‑‑‑‑‑‑‑VETRLRW‑‑‑‑‑‑‑‑‑‑‑G‑‑‑‑‑‑‑‑‑NESEDIKDIV

>G7K107

GSDTRNTFVDHLYAHLIRKGIFTFKDDAQLNKGHSISTQLLHAIRQSRVSIIIFSKDYASSTWCLDEMATIADC‑QLNLN‑‑‑HTVFYDVAPSDVRKQKGVYQNVFAVHSKISK‑‑‑‑‑H‑EPHKVDCWKRAMTCLAGSSGWDV‑R‑‑NKPEFEEIEKIV

>G7KKT5

ADDTASDFAGNLYKALNDRGIRTFMD‑‑‑DNKKLENREKHFKTIEESKTAIIVLSRNYASSLSCLEQLASILDCR‑KR‑‑RLVWPVFYNMGPSCCKTRTGKMGKAWSNHYK‑‑‑‑‑‑‑‑‑‑NLKKTKK‑‑‑ALHQVADLSGFHL‑NNGDGSELELIERIV

>G7KJ34

GTDTRYGFTGNLLKALIDKGIRTFHDDDDLQRRDKVTPII‑‑‑IEESRILIPIFSANYASSSSCLDTLVHIIHCY‑KTKGCLVLPVFFGVEPTDVRHHTGRYGKALAEHENRFQND‑‑TK‑NMERLQQWKVALSLAANLPSYHD‑DS‑HGYEYELIGKIV

>G7JF29

GADTRFNFTDHLFSALQIRGIVAFRDDTKLKKGESIAPELLRAIEASRTFIVVFSNNYASSTWCLRELQYILHC‑VQLSGKRVLPVFYDVDPSEVRKQSGSYKKAFAQHEERFK‑‑‑‑‑Q‑DTEVLQGWRTALTQVANLSGWDI‑R‑‑DKPQSAEIKKIV

>G7JMY5

GEDTRASFTSHLTFSLQNAGIIVFKDDQSLERGEHISTSLLQAIEISRIAVIVFSKNYADSSWCLRELVQIMSCY‑STIGQVVLPVFYDVDPSEVRRQTGDFGKSFQNLLNRI‑‑‑‑EERR‑KDMVRKWIDALHTAAGLAGFVVLN‑‑SRNESEVIRDIV

>G7IUH0

GTDIRHGFLSHLRKELRQKQVDAYVDD‑RLEGGDEISKALVKAIEGSLMSLIIFSKDYASSKWCLEELVKIVECM‑ARNKQVVIPVFYNVNPTDVRHQKGTYGDSLAKHEKN‑‑‑‑‑‑KG‑SLAKVRNWGSALTIAANLSGFHS‑SK‑YGDEVELIEEIV

>G7KJ43

GADTRHGFTGNLYKALTDKGIYTFIDDNDLQRGDEITPSLKNAIEKSRIFIPVFSENYASSSFCLDELVHITHCY‑DTKGCLVLPVFIGVDPTDVRHHTGRYGEALAVHKKKFQND‑‑KD‑NTERLQQWKEALSQAANLSGQH‑‑YK‑HGYEYEFIGKIV

>G7JEW9

GEDTRASFTAHLNASLLNAGINVFKDDDSIYKGARISKSLPEAIEQSRIAVVVFSKHYADSKWCLNELVKIMKCH‑RAIRQIVLPVFYDVDPLEVRHQKKKFGKAFQNILTMLSNQTESSL‑RMLRRNWTTALHEAAGLAGFVVLH‑‑FMNESEAIKDIV

>G7LF30

GEDTRSSFVSHLHAALSNAGINTFLDDKKLEKGEELGPELLRAIEVSRISIIVFSKSYITSSWCLKELEQIMKCR‑KNYGQVVMPIFYHVDPSALRHQKDGYGKALQATAKR‑‑‑‑‑GERR‑KYALSNWKIALTEAANISGWDINK‑‑SSNEGELMPLII

>G7J7L4

GKDTRGSRAS‑‑‑‑‑‑‑‑‑‑‑‑‑‑‑‑‑‑‑‑‑KGRWHSDIIKTNNQKSQISIIVFSKNYVESSWCMDELLEIMECH‑KTIGQVVLPVFYNVDPSEVRHQIGEFGIAFQNLLTKI‑‑‑‑EHKL‑QYFEQAWRLALREAAGLVGFVVLN‑‑SKNESEVIKDIV

>G7LCP7

GEDTRTNFTSFLHAALCKNHIETYIDY‑RIEKGEEVWEELERAIKASALFLVVFSENYASSTWCLNELVEIMKCK‑KNDNVVVIPVFYRIEPSHVRKQTGSYHTALAKQKKQGK‑DK‑‑‑‑‑‑‑‑IQRWKNALFEVANLSGFDS‑ST‑YRTESDLIGDII

>G7KJR2

GGDTRYGFTGNLNRALCDKGIRTFMDDRELQGGEEITSSLFKAIEESRIFIPVLSINYASSSFCLDELVHIIHCF‑KESGRLVLPIFYDVEPSHVRHHKGSYGKALDDHIERFQNN‑‑KH‑SMDRLQKWKIALTQTANFSGHQI‑NPRNGYEYEFIEKIV

>G7LDV9

GEDTRAGFTSHLYETFLQSKFHTYIDY‑RIQKGDHVWAELTKAIKQSTIFLVVFSKNYASSTWCLNELVEIMECS‑NKDNVAVIPVFYHIDPSRVRKQTGSYGTALAKHKKQGC‑DH‑‑‑‑‑‑KMMQNWKNALFQAANLSGFHS‑TT‑YRTESDLIEDIT

>G7KK77

GSDTRFGFTGNLYKALRDCGIHTFIDDRELQGGDEISPSLVKAIEESRIFIPVFSINYASSSFCLDELVHIIDCF‑NTKGCLVLPVFYGVDPSHIRHQTECFGEAIAKQEVKFQNQ‑‑KD‑DMDRLLKWKCALNKAANFSGHHF‑NFGNEYEYEIITKIV

>G7KIH7

GTDTRFGFTGNLYKALSDKGIRTFIDDKELQKGDEITPSLLKRIEESRIAIIVFSKEYASSSFCLDELVHIIHYF‑KEKGRLVLPVFYDVEPSHVRHQNYSYGEALAKHEERFQKS‑‑KK‑NMERLLKWKIALNKVADLSGYHF‑NLGNEYERDFIEKIV

>G7K8D5

GEDTRYTFTSHLHATLTRLDVGTYIDY‑HLQRGDEISSALLRAIEEASLSVVVFSKNYGNSKWCLDELVKILECK‑KMRGQIVLPIFYDIEPSDVRNQTGSYADAFVKHEERFHGNL‑‑‑‑‑‑ERVQKWREALREAANLSGWDC‑ST‑NRMESELLEKIA

>G7KP09

GEDTRNNFTDFLFDALETKGIFAFRDDTNLQKGESIEPELLRAIEGSRVFVAVFSRNYASSTWCLQELEKICKC‑VQRSRKHILPVFYDVDPSVVRKQSGIYCEAFVKHEQRFQ‑‑‑‑‑Q‑DFEMVSRWREALKHVGSISGWDL‑R‑‑DKPQAGVIKKIV

>G7I276

GGDTREGFIGHLYKALTDKGIHTFIDDRELQRGDEIKPSLDNAIEESRIFIPVFSINYASSSFCLDELVHIIHCY‑KKKGRLILPVFYGVDPTHIRHQSGSYGEHLTKHEESFQNS‑‑KK‑NMERLHQWKLALTQASNLSGYHS‑S‑‑RGYEYKFIGEIV

>G7LI81

GEDTRTNFTAQLHQALSDRSIESYIDY‑NLVKGDEVGPALTKAIDDSHMSLVVFSKDYATSKWCLDELVHILQCR‑KLNGHVVIPVFYNIDPSHVRHQKESYQMAFARFERELAHSK‑‑S‑HVDKVSEWKAALNLAANISGWDS‑RK‑YRDDTQVIGNIV

>G7KIE7

GSDTRYGFTGNLYKALTDKGIHTFIDDRELQRGDEIKPSLDNAIEESRIFIPVFSPNYAYSSFCLDELVHIIHCY‑KTKGRLVLPVFYGVDPTHIRHQTGSYGEALAKHAKRFHNN‑‑NT‑NMERLQKWKIALAQAANLSGDHR‑H‑‑PGYEYDFIEKIV

>G7IW61

GKDTRNNFTGNLYNSLNQRGIQTFMDDEEIQKGEEITPTLLQAIEESRIFIAIFSPNYASSTFCLTELVTILECS‑MQ‑GRLFSPVFYDVDPSQIRYLTGTYAEAFKKHEERFGDD‑‑‑‑‑K‑HKMQKWRDALHQAANMSGWHF‑KPGYELEYKFIEKIV

>G7K8D0

GEDTRNNFTSHLNGALKRLDIRTYIDN‑DLNRGDEIPTTLIRAIEEAKVSVIVFSKNYAVSKWCLEELMKILEIK‑KMKGQIVVPIFYDVDPSDVRNQRGSYAEAFNNHERNFKKKI‑‑‑‑‑‑‑KVQEWRNGLMEAANYAGWDC‑NV‑NRTELALVEEIA

>G7KDY8

GEDTRNGFTGNLYKALCGKGINTFIDDKNLGKGEEITPALMMAIQESRIAIVIFSENYASSTFCLKELTKIMECI‑KHKGRLVLPIFYQVDPADVRHQKGSYANALASHERKK‑‑‑‑‑TI‑DKIMVKQWRLALQEAASILGWHF‑EH‑‑GYEYELIGKIV

>G7JKN6

GEDIRHGFLGHLAKAFSRKQINAFVDD‑KLKRGDDISNSLVEAIEGSFISLIIFSENYASSSWCLEELLKIIDCK‑EKYGQIVIPVFYGVDPTNVRHLKKSYGNAFAELEK‑‑‑‑‑‑‑RH‑SSLKVQIWRYALNKSANLSGIKS‑LD‑YRNDAELLEEII

>G7ZVE9

GEDTRAGFTSHLHAALSRTYLHTYIDY‑RIEKGDEVWPELEKAIKQSTLFLVVFSENYASSTWCLNELVELMECR‑NKDNIGVIPVFYHVDPSHVRKQTGSYGSALAKHKQENQ‑DD‑‑‑‑‑‑KMMQNWKNALFQAANLSGFHS‑ST‑YRTESNMIEDIT

>G7KJN1

GSDTRYGFTGNLYKALTDKGINTFIDKNGLQRGDEITPSLLKAIEESRIFIPVFSINYASSSFCLDELVHIIHCY‑KTKGRLVLPVFFGVEPTVVRHRKGSYGEALAEHEKRFQND‑‑PK‑NMERLQGWKKALSQAANLSGYHD‑SP‑PGYEYKLIGKIV

>G7LI62

GEDTRYGITNLIYDALIHKSIKTFIDY‑ELNRGEDVWPKLSKAIEESHISVVVFSENFATSKWCLEELVKVLECR‑KDHGQVVIPVFYKTNPSHIRNQTHSYEKAFAKHERDLGTKS‑AS‑NKLIVLKWRSALTEAATISGWDT‑HT‑HKDESNLIHKIV

>G7JUR1

GEDTRSNFTSHLHAALCRTKVKTYIDY‑NLKKGDYISETLVKAIQDSYVSIVVFSENYASSTWCLDELTHMMKCL‑KNNQIVVVPVFYNVDPSHVRKQSGSYMVAFEKHVCNLNHFN‑‑‑‑‑‑‑KVNDWREALAQATSLAGWDS‑RK‑YMLESELVEDIV

>G7J6M1

GEDTGRKFTSHLYEALSKK‑IITFIDN‑ELEKGDEISSALIKAIEDSSASIVIFSKDYASSKWCLNELVKILECK‑KDQGQIVIPIFYEIDPSHVRNQNGSYGQAFAKHARDLKQNK‑‑‑‑‑‑EMLKKWKDALTEAANLAGWHS‑QN‑YRIESNFIKDIV

>G7KPI2

GEDTRNNFTDYLFDALETKGIYAFRDDTNLKKGEVIGPELLRAIEGSQVFVAVFSRNYASSTWCLQELEKICEC‑VQGPEKHVLPVFYDIDPSEVRKQSGIYCESFVKHEQRFQ‑‑‑‑‑Q‑DPHKVSRWREALNQVGSISGWDL‑R‑‑DKPQAGEIKKIV

>G7IW59

GIDTRNNFTGNLYNSLNQRGIRTFFDDEEIQKGEEITPTLLQAIKESRIFIVVFSTNYASSTFCLTELVTILGCS‑KQ‑GRIFLPIFYDVDPSQIRNLTGTYAEAFAKHEMRFGDE‑‑‑‑‑E‑DKVQKWRDALRQAANMSGWHF‑KPGSESEYKFIGKIV

>G7LGU3

GEDTRLGFTGFLYKTLSEKGFHTFIDH‑HADAGRGTTKTLVDAIEESRIGIVVFSENYASSTWCLDELAYIIDSFSKNFRRSVFPVFYNVDPSHVRHQSGIYGQALDSHQKNN‑‑‑‑‑NF‑NSEKLNKWKNALKQAANLSGFHF‑KHGDGYEYELIDKIV

>G7KIG1

GTDTRFSFTGNLYKALSDNGIRTFIDDKDLQSGDEITPSLLKNIEDSRISILVFSENYATSSFCLDELVHIIHCS‑KEKGSMVIPVFYGIEPSHVRHQNSSYGEALAKHEEVFQNN‑‑KE‑SMERLRKWKKALNHAANLSGHHF‑NFGNEYEHHFIGKIV

>G7LHH8

GQDTRETFTSHLHYALCKENIITYIDD‑NLVKGDEIGEALAEAIQDSRISLVVFSKNYATSKWCLNELLKILECK‑K‑HGQVVIPVFYNTGTSEVRNQTGSYEKPFSHYEIEAINNE‑‑‑‑SANTVSEWRAALAEAANIPGWDS‑QS‑YKDDSQVIQSIV

>G7KHT0

GSDTRNKFTGNLYKALVDKGIRTFIDDNDLERGDEITPSLVKAIEESRIFIPIFSANYASSSFCLDELVHIIHCY‑KTKSCLVFPVFYDVEPTHIRNQSGIYGEHLTKHEERFQNN‑‑EK‑NMERLRQWKIALIQAANLSGYHY‑SP‑HGYEYKFIEKIV

>G7JLX1

GEDTRASFISHLTSSLQNAGILIFKDDQSLQRGDHISPSLVHAIESSKISVIVFSKNYADSKWCLQELWQIMVRH‑RTTGQVVLPVFYDVDPSEVRHQTGEFGKSFLNLLNRI‑‑‑‑EKW‑‑‑‑MALEWRNELRVAAGLAGFVVLN‑‑SRNESEVIKDIV

>G7LF48

GEDVRRTFVSHLYAVLSNAGINTFLDNEKLEKGEDIGHELLQAISVSRISIIVFSKNYTESSWCLNELEKIMECR‑RLHGHVVLPVFYDVDPSVVRHQKGDFGKALEVAAKS‑‑‑‑‑EEVM‑VKELGKWRKVLTEASNLSGWDGSA‑‑FRSDRELVKKIV

>G7INY1

GTDTRYGFTGNLYRALSDGGFCTFIDDRELHGGDEITQSLVKAIEESMIFIPVFSINYASSIFCLDELVHIIHCFDQEKGRKILPIFYDVEPSHVRHQTGSYGKAIARHEKRFQNN‑‑KEKNMKRLHKWKMALNQAANLSGHHF‑NPRNEYQYKFIGDIV

>G7KIF8

GADTRYEFTGNLYKALTDKGIHTFFDDRELQRGDKIEQSLNNAIEESRIFIPVFSANYASSSFCLDELVHIIRVY‑KEKGRLVLPVFYGVDPGDIRHQRGSYAIHLTKHEKRFGNN‑‑KE‑NMEKLLQWKKALKQAADLSGFHF‑SG‑NGYEYKRIGEII

>G7KP15

GDDTRNNFTGYLLDALKTNGIYAFRDDTNLQKGESIGPELLRAIEGSQVFVAVFSRNYASSTWCLQELEKICEC‑VHVSRKHILPVFYDVDPSEVRKQSGIYGEAFTIHEQTFQ‑‑‑‑‑Q‑DSQMVSRWREALKQVGSIAGWDL‑C‑‑DKPQSAEIRMIV

>G7INJ8

GDDTRRKFTSHLNEALKKSGLKTFIDN‑ELKKGDEISSALIKAIEESCASIVILSENYASSKWCLNELVKILECK‑KDNGQIVIPIFYEIDPSHVRYQIGSYGQAFAKYEKNLRHKK‑‑‑‑‑‑DNLQKWKDALTEVSKLSGWDS‑KN‑SRIESDFIKDIV

>G7KHV7

GADTRS‑‑‑‑‑‑‑RASPAISIKLFV‑‑‑‑‑‑‑TKESEPSLMREVMKSHL‑‑‑‑‑‑‑HYASSSFCLDELVH‑‑‑‑‑‑‑ENSRLVLPVFYDVEPSEVRHYNNRYGEALTEFEERFQNN‑‑KE‑NMERLQKWKIALNQAYNLSGYHF‑KE‑DEYEYEFIKKIV

>G7JF22

GDDTRNKFTDHLFGALRRKNIAAFRDNRHLNSGASIEPALFRAIEVSQIFIVVLSKSYASSTWCLRELVYILHC‑SQPSEKRVRTVFYDVNPSEVRKQSGSYAKAFAKHEENFG‑‑‑‑‑Q‑DHVKVRQWREALTQAGNISGCDL‑G‑‑NKPENEEIETIV

>G7K9Q6

GDDTRFHFTGNLYKALCDKGIRVFIDDKELQRGDKITPSLIKAIEDSRIAIPVFSKNYAFSSFCLDELVNIIDGF‑SAKGRLVLPVFYDVDPSHVRHQIGSYGEAIAMHEARLKRD‑‑KEMNMDRLQKWKTALNQAANLSGYHF‑NHGNEYEHEFIGRIM

>G7KSB9

GPDTRNTFVDHLYAHLTRKGISTFKDDKSLQKGESISLQLLQAIKDSRVSIIVFSKDYASSTWCLDEMAAIDES‑SRRLKLVVFPVFYDIDPSHVRKRSGAYEDAFVLHNELFK‑‑‑‑‑H‑DPDRVAQWRRAMTSLAGSAGWDV‑R‑‑NKPEFDEIEKIV

>G7JKN3

GEDIRHGFLGHLIKAFPRKQINAFVDE‑KLKRGDDISHALVEAIEGSFISLVIFSENYASSHWCLEELVKIIECK‑EKYGRIVLPVFYGVDPTNVRHQKKSYKSAFSELEK‑‑‑‑‑‑‑RY‑HLSKVQNWRHALNKSANLSGIKS‑LD‑FRNDAELLEEII

>G7JSB1

NKDAGS‑FALQLYTALSEARVSVFWDNARLGSGDRISTSAVNIIGNCRVAVIIFSMKYFNSMWCLQEFEKITECQ‑RTDG‑TVLPVFFDAYPGRLHK‑‑N‑FGDPFDYYDRLMVTET‑KE‑Q‑DIFMSWVAAVSKAFKYSASYSRKV‑SGNEGEYIKDVV

>G7K8C8

GEDTRNNFTSHLNGALKRLDIRTYIDN‑DLNSGDEIPTTLVRAIEEAKLSVIVFSKNYAVSKWCLEELMKILEIK‑KMKGQIVVPVFYDVDPSDVRNQRGSYAEAFAKHENNFEGKI‑‑‑‑‑‑‑KVQEWRNGLLEAANYAGWDC‑NV‑NRTELALVEEIA

>G7LF46

GKDTRKTFVSHLYAALTDAGINTFLDDENLKKGEELGPELVRAIQGSQIAIVVFSKNYVNSSWCLNELEQIMKCK‑ADNGQVVMPVFNGITPSNIRQHS‑‑‑‑‑‑‑‑‑PVIL‑‑‑‑‑VDEL‑DQIIFGKKRALRDVSYLTGWDMSN‑‑YSNQSKVVKEIV

>G7KJ27

GTDTRYGFTGNLLKALIDKGIRTFHDDDDLQRRDKVTPII‑‑‑IEESRILIPIFSANYASSSSCLDTLVHIIHCY‑KTKGCLVLPVFFGVEPTDVRHHTGRYGKALAEHENRFQND‑‑TK‑NMERLQQWKVALSLAANLPSYHD‑DS‑HGYEYELIGKIV

>G7IW72

GIDTRNNFTGNLYHSLHQRGIQTFMDDEEIQKGEEITPTLLQAIKQSRIFIAIFSPNYASSTFCLTELVTILECS‑MQ‑GRLFLPVFYDVDPSQIRNLTGTYAEAFAKHEVRFGDE‑‑‑‑‑KDSKVQKWRDALRQAANVSGWHF‑KPGFESEYKIIEKIV

>G7IM67

GADTRSTFVDHLHAHLTTKGIFAFKDDKRLEKGESLSPQLLQAIQSSRISIVVFSKNYAESTLCLEEMATIAEY‑HTELKQTVFPIFYDADPSHVRKQSGVYQNAFVLLQNKFK‑‑‑‑‑H‑DPNKVMRWVGAMESLAKLVGWDV‑R‑‑NKPEFREIKNIV

>G7IIT2

GHDTRVGFTSHLESALCRNYFLTYTDY‑RIKSGKKIWDELVKAMNESTLFLVVFSENYADSSWCLDELVEIMKCR‑RKRQVVVLPVFYRIEPSYVRKQTGSYGAALARHSQGHRDSH‑‑‑‑‑‑‑‑IQLWKDALREAGNLSGFHV‑TE‑KRSESAWIEDIN

>G7LGU7

GLDTRRGFSGFLNKYLTDRGFRTFFDDGEIEIGTQITVRIHKGIEDSRIFIPILSENYASSSFCLDELVKILAEFQK‑NGR‑VFPVFYYVSISDVKNQTGSYGQALTVLKN‑‑‑‑‑‑‑‑‑‑EHERFEKWISALTSIADFRGCHMERAK‑GYEFQYIYEII

>G7JY83

GEDTRNTIVSHLYAALQNSGVYTFLDDQKLTKGEVLGPALRKAIEESKIFIVVLSPDYAGSSWCLRELVHIMDCH‑ESYGRIVLPVFYGVEPSEVRKQSGDFGKALKLTATK‑‑‑‑‑‑‑RE‑DQLLSMWKTALTKVGNLAGWDYNI‑‑FRNEGELVELIV

>G7KJR6

GIDTRYGFTGNLYSDLCKKGIHTFFDDRELQGGDEITSSLFKVIEESRIFIPVLSINYASSSFCLDELVHIIHCF‑KENRRLVLPIFYDVEPSHVRHHKGSYGKALDDHIERFQNN‑‑KH‑SMDRLQKWKMALTQTANFSGHQI‑NPRNGYECEFIEKIV

>G7K3B3

GEDVRHNFIGYLRDALQHRGINAFFDDKNLRIGEDISPALSKAIEESKIAVIVFSENYASSRWCLGELVKIIECT‑KNKKQISFPIFFHVDPSDVRHQKNSYEKAMVDHEVKF‑‑‑‑‑GK‑DSENVKAWITALSEAADLKGHHI‑NT‑‑GSEIDHIKEIV

>G7JF20

GEDTRNNFTDFLFDALEEKGVFAFRDDTNLQKGESIAPELFHAIEGSQVFVVVLSKNYAFSTWCLKELEYILCC‑VQASKKYVLPVFYDVDPSLVRKQTGIYSEAFVQHGHRFK‑‑‑‑‑Q‑DSQMVLRWRAALTQVADLSGWDL‑R‑‑DKRQSLEIKKIV

>G7KL74

GEDTRHGFTGYLKKALDDKGVRTFMDDKELRKGEEITPSLLKAIEQSMMAIVVLSENYASSSFCLQELSKILDTM‑KMVGRSVFPVFYKVDPSDVRKLKRSFGEGMDK‑‑HKA‑‑‑‑‑NS‑NLD‑‑‑KWKVSLHQVTDLSGFHY‑K‑GDTPEHMFIGDIV

>G7L6T3

GEDTRTSLVSHMDAALTNAGINTYID‑QQLHKGTELGPELLRAIEGSHISILVFSKRYTESSWCLNELKKVMECH‑RTHGQVVVPIFYDVDPSVVRQQKGAFG‑‑‑‑‑‑‑‑‑‑‑‑‑‑‑EIL‑KYMLSRWTSALTQAANLSGWDVTN‑‑CRSEAELVQQIV

>G7LDU5

GEDTRECFTKKLYESLHKQGVRAFMDDEGLDRGDHIATTLLEAIDDSAASIVIISPNYADSHWCLDELNRI‑‑C‑‑D‑LERLIIPVFYKVDPSHVRKQLG‑FQDGFNYLEKRFANE‑‑‑‑‑‑KDKILKWRDSMLKIGGLAGFVFSSD‑DGDHENLIRRLV

>G7KDY7

GEDTRKGFTGNLYKALCGKGIDTFIDDQELRKGEEITPALMMAIQESRIAIVIFSENYASSTFCLKELTKIMECI‑KHKGRMVLPVFYHVDPCIVRHQKGSYAKALADHESNK‑‑‑‑‑KI‑DKAKVKQWRLVLQEAASISGWHF‑EH‑‑GYEYEFIEKII

>G7LDL6

GDDTRAGFTSHLHAALCRSNFHTYIDY‑RIEKGDEVWGELQKAINESTLFLVVFSENYAFSTWCLNELVQIMECS‑NNDNVVVIPVFYHVDPSHVRKQTGSYGTALAKHI‑‑‑‑‑DH‑‑‑‑‑‑KMLQNWKNALFEASNLSGFHS‑TT‑YRTESDLIEDII

>G7IM44

GPDTRNSFVDHLYAHLTRKGIFAFKDDKSLEKGEFISPQLLQAIRNSRIFIVVFSKTYAESTWCLEEMAAIADC‑CEYFKQTVFPIFYDVDPSDVRKQSGVYQNDFVLHKKKFT‑‑‑‑‑R‑DPDKVVRWTKAMGRLAELVGWDV‑R‑‑NKPEFREIENIV

>G7I3L5

GTDTRYTFTGHLYKALHNKGIMTFIDDDHLQKGDQITPSLLKAIENSRIAIVVLSKNYASSSFCLQELCKILEN‑‑‑‑‑GGLVWPVFYEVEPSNVRKLSGSFGEAMAVHEVRY‑‑‑‑‑SD‑DVDRLEKWKKGLYQVANLAGFHY‑K‑NDGYEHEFIGKIV

>G7KL67

GEDTRYGFTSYLKKALDDKGVRTFMDDEELQKGEEITPSLLKAIEDSQIAIVVLSKNYASSSFCLQELSHILHSI‑KK‑GRSVLPVFYKVDPSVIRKLEKSYGEAMDK‑‑HKA‑‑‑‑‑NS‑NLD‑‑‑KWKVCLHQVADLSGFHY‑K‑KDMPEHKFIGEIV

>G7IIM2

GEDTRNNFTDFLFDALERKDIFAFRDDTNLQKGESIGSELLRAIEGSQVFVAVFSRYYASSTWCLEELEKICEC‑VQVPGKHVLPVFYDVDPSEVRKQSGIYHEAFVKHEQRFQ‑‑‑‑‑Q‑DLQKVSRWREALKQVGSIAGWDL‑R‑‑DKPQCAEIKKIV

>G7KPI1

GEDTRNNFTDFLFDALETKGIFAFRDDTNLQQGESLEPELLRAIKGFQVFVVVFSRNYASSTWCLKELEKICEC‑VKGSKKHVIPVFYDVDPSEVRKQSGIYCEAFVKHEKRFQ‑‑‑‑‑Q‑GFEMVSRWREALKQVGSISGWDL‑C‑‑DKPQAGEIKKIV

>G7LCP9

GEDTRTNFTSFLHAALCKNHIETYIDY‑RIEKGEEVWEELEKAIKASALFLVVFSENYASSTWCLNELVEIMKCK‑KNDNVVVIPVFYRIEASHVRKQTGSYHTALLKQKKQGK‑DK‑‑‑‑‑‑‑‑IQRWKIALFEVANLSGFDS‑ST‑YRTEADLIGDII

>G7JKN5

GKDIRGDFLSHLIEALRRKKIKAFVDD‑ELKRGDEILQSLVRGIEGSLISLIIFSQDYASSRWCLEELVTILQCR‑EKYGQIVVPIFYGIDPADVRYQMKSYENAFVEHQR‑‑‑‑‑‑‑VY‑SSTKVQIWRHALNKSANLSGIKS‑SD‑FRNDVQLLKEIV

>G7LIZ9

GEDTRNNFTDHLFGALHKNRIVVFRDDINLKKGGNISSELLQAIKESHILIVIFSKNYASSTWCLQELVNIADC‑IHVQGQTVLPIFYDVSPSEVRKQTGDYEKPFLEHGERFK‑‑‑‑‑G‑NLEAVQRWRGALTQVANLSGWDI‑K‑‑DKSQYAEIEKII

>G7JY79

GEDTRNTIVSHLHAALQNSGVNTFLDDQKLKKGEELEPALRMAIEQSKISIVVLSPNYAGSSWCLDELVHIMDCR‑ESYGRTVVPVFYRVNPTQVRHQTGDFGKALELTATK‑‑‑‑‑‑‑KE‑DQQLSKWKRALTEVSNISGWRYNI‑‑SRNEGELVKGIV

>G7KKS7

GEDTRHGFTGNLWKALSDRGIHTFMDDEELQKGEEITPSLIKAIEDSNMAIIVLSKNYASSTFCLKELSTILYSI‑KK‑GRCVWPVFYDVEPSDVRKLKRSYGEAMVEHEARD‑‑‑‑‑HS‑NMDLLQKWKNALNQVANLSGFHF‑K‑NDEYEHVFIGKIV

>G7LI80

GEDTRTNFTAQLHRALTDSSIESYIDY‑SLVKGDEVGPALAKAIQDSHMSLVVFSENYATSKWCLDELLHILQCR‑KHHGQVVIPVFYNIDPSHVRHQKESYEMAFARYDRDLAHSK‑‑S‑QLDKVSEWKAALKLAANISGWDS‑RK‑YRDDSQVIDKIV

>G7JP38

GTDTRYGFTGNLYEALRVKGIHTFIDDRELQRGDQITPSLLKAIQESKIVIIVFSNHYASSSFCLDELVHIIHCS‑KENGCLVLPIFYGVEPSHVRYQTGSYGEALAEHEEARKKE‑‑KY‑NMEKLQKWEMALKQAANLSGYHF‑NARTGYEYEFIQMIV

>G7IQA0

GIDTRNNFTRDLYDILYQNGIHTFFDEEQIQKGEEITPALFQAIQQSRIFIVVFSNNYASSTFCLNELVVILDCS‑NH‑GRLLLPVFYDVDPSQVRHQSGAYGEALGKHEKRFCDD‑‑‑‑‑K‑DKVQKWRDALCQAANVSGWHF‑QHGSQSEYKFIGNIV

>G7KKT7

GEDTRIGFVNNLRKALNDKGIKTFID‑‑KEHQKEEITPAILNVIRECKIVIIVLSNNYAAS‑‑‑‑‑‑‑‑SFL‑‑‑‑‑‑‑‑‑‑‑‑‑‑FYNIEPCTVRKETGSYG‑ALENHER‑‑‑‑‑‑‑‑‑‑D‑KKTRKWRGALTEVSNLSGWQF‑KHG‑GNEHKIISNIV

>G7KIG4

GSDTRYGFTGNLYKALTDKGIHTFMDDRELQRGDEIKRSLDNAIEESRIFIPVFSANYASSSFCLDELVQIINC‑‑KEKGRVVLPVFYGMDPTNVRHHRGIYGEALAKHEKRFQND‑‑MD‑NMERLQRWKVALNQAANLSGYHF‑S‑‑PGYEYEFIGKIV

>G7JVS3

G‑GTRYSFTDHLYRSLLRQGINVFRDDQNLKIGHEIGPSLLQAIEASRISIVVLCKEYASSTWCLDELVKIVDC‑‑YNNGKSKNSYEDAIR‑‑‑‑‑‑‑‑‑‑‑‑‑‑‑‑KHEKRF‑‑‑‑‑GR‑ESEKVKAWKLALNRVCALSGLHC‑KD‑DVYESEFIEKIV

>G7KMZ4

GSDTRYGFTGNLYKALTDKGIHTFIDDSELQRGDEITPSLDNAIEESRIFIPVFSANYASSSFCLDELVHIIHLY‑KQNGRLVLPVFFGVDPSHVRHHRGSYGEALAKHEERFQHN‑‑TD‑HMERLQKWKIALTQAANLSGDHR‑S‑‑PGYEYDFIGDIV

>G7IN46

GDDTRRKFTSHLNEALKKSGVKTFIDS‑ELKKGDEISSALIKAIEESCASIVIFSEDYASSKWCLNELVKILECK‑KDNGQIVIPIFYEIDPSHVRNQIGSYGQAFAKHEKNLKQ‑‑‑‑‑‑‑‑‑‑‑QKWKDALTEVSNLSGWDS‑KS‑SRIESDFIKDIV

>G7J1L8

GEDTRKSLVSHLYAALSNAGINTFLDDEKLKKGWEVEPELLRAIQGSQICLVIFSEHYTESSWCLVELVKIMEHR‑KTNNNVVIPIFYHVDPSVVRRQVGDFGKALEAITKR‑‑‑‑‑KE‑R‑QELLRTWKRALTQAANISGWDSSI‑‑FRSESELVNKIV

>G7KJ53

GSDTRYGFTGNLYKALTDKGINTFIDDNGLQRGNEITPSLLKAIEESRIFIPVFSINYASSSFCLDELDHIIHCY‑KTKGRPVLPVFFGVDPSHVRHHKGSYGEALAEHEKRFQND‑‑PK‑NMERLQGWKDALSQAANLSGYHD‑SP‑PGYEYKLIGKIV

>G7JF23

GEDTRFNFTDHLFAALQRKGIFAFRDDTKLQKGESIAPELIRAIEGSQVFIAVLSKNYASSTWCLRELEYILHY‑SQVFGRRVLPVFYDVDPSEVRHQKGIYGEAFSKHEQTFQ‑‑‑‑‑H‑DSHVVQRWREALTQVGNISGWDL‑R‑‑DKPQYEEIKKIV

>G7L6L9

GEDTRKGFTDHLCAALERKGITTFRDDKDLERGQVISEKLINAIKDSMFAITVLSPDYASSTWCLDELQMIMEC‑‑SNKGLEVLPVFYGVDPSDVRHQRGCFEESFRKHLEKF‑‑‑‑‑GQ‑HSDRVDRWRDAFTQVASYSGWDS‑KG‑‑QHEALLVESIA

>G7L9E6

GKDTRNDFVSHLNAALQNRGIKTFLDDEKLGKGEKLGPQLEKAIEGSLISIVVLSPDYAESSWCLNELVHILKCQ‑KTYGQVVMPVFYHVDPSVVRKQTGDFGKALELTATK‑‑‑‑‑‑‑KE‑DKLLSNWKTALKQVATIAGWDCYN‑‑IRNKGELAKQIV

>G7KJ57

GTDTRHGFTGNLYKALTDKGIHTFIDDNDLPRGDEITPSLLKAIDESRIFIPVFSINYASSSFCLDELVHIIHCY‑KTKGRLVLPVFFGVEPTKVRHQKGSYGEALAEHEKRFQND‑‑KN‑NMERLQGWKVALSQAANFSGYHD‑SP‑PGYEYEFTGEIV

>G7KHU9

GSDTRNTFTGNLYKALVDKGIRTFFDDNDLQRGDEITPSLVKAIEESRIFIPIFSANYASSSFCLDELVHIIHCY‑KTKSCLVLPVFYDVEPTHIRHQSGSYGEYLTKHEERFQNN‑‑EK‑NMERLRQWKIALTQAANLSGYHY‑SP‑HGYEYKFIEKIV

>G7LI71

GEDTRKNFTGKLHEALKKENIETYIDL‑YVKVGDEVGPMLIQAIHESQISVIVFSKNFVTSKWCLEELLHILECR‑KHHGQVVLPFYYETDPSNVGLGKGSYEKAFARYERELMNNQDLT‑NPGKVSKWKAALVEVAAISARDS‑RH‑YSDDSQFIQCIV

>G7LF47

GEDTRKTFVSHLYAALTNAAIRTFRDDKELRKGNKLEPEIKRAIEGSRISIVVLSPYYAGSSWCLNELVHILHCS‑HTYGQVVMPVFYHVDPSHVRKLEGNFGTIFELHAIH‑‑‑‑‑‑‑RE‑HELLSKWKTVLTEVSNLSGWDLNN‑‑ISNEGELVKQIV

>G7ZVL3

GEDTHKTFKSHLNSALRRLDIKTYIED‑NLVRGDEISQPLLKAIDE‑‑‑‑‑‑‑‑‑‑‑‑‑‑‑‑‑‑‑‑‑‑‑‑ILECR‑KNKGQMILPVFYEVDPFHVRHQLGSYAEAFIKHEQRFGSTM‑‑‑‑‑‑NVLQKWRDALGEAANHSG‑‑‑‑‑‑‑‑‑TEAELVEEIA

>C6TIX3

EAETRHSFTGTLYHALQSARFKTYMENGKLRRGDKIATAILTAMEASRISIVVFSPYFASSTCCLDQLVHIHRC‑‑NTKNQLILPIFYDVD‑SDVRDQLNTFGQAMLQHQHRFGKS‑‑‑‑‑‑SDKVLQWSSVLSHVANLTAFCF‑SSGDQYEYQFVEEIV

>G7JKN4

GEDIRHGFFGHLVIAFPRKQINAFVDE‑KLKRGDDMSHSLVEAIEGSPISL‑‑‑‑‑‑‑‑‑‑‑‑‑‑‑‑‑‑‑‑‑‑CK‑EKYGQIVIPVFYGVDPTNVRHQKKSYENAFAELEK‑‑‑‑‑‑‑RC‑NSSKVQIWRHALNTSANLSGIKS‑SD‑FRNDAELLEEII

>G7KJR8

GSDTRYGFTENLYRALCHKGIHTFIDDRELQGGDEITPSLFKAIEESRIFIPVLSINY‑‑‑‑‑‑‑‑‑‑‑‑‑‑‑‑‑‑‑ENRRLVLPIFYDVEPSHVRHHKGSYGKALDDHIKKFQNN‑‑KD‑NMERLQKWKMALTQTSNFSGHHF‑NPGNGYEYKYIKKIV

>G7JSC4 (0‑355)

HQDAAS‑FATGIYTALRKSRFHVFWDDEKLGSGDRIPTSILNVIEDCKVAVIVFSRNYVNSRSCLQEFEKITEC‑‑LTSG‑IVLPVLYDLNSGTVE‑‑‑‑‑‑‑ETFHDFDRWI‑KET‑SE‑EKDKFMSWVAAVTKATTYSGVDF‑‑A‑DSYGREYVVDVV

>G7JSC4 (356‑1864)

GEDSRAKFMSHIFSSLQNAGIHTFRDDDQIQRGDQISVSLLRAIGQSRISIIILSTNYANSRWCMLELVKIMEIG‑RTRGLVVLPVFYEVDPSEVRHQEGQFGKSFEDLISTI‑‑‑‑‑‑‑‑‑ESTKSNWKRDLIDIGGIAGFVLKD‑‑SRNESADIKNIV

>G7JSA9 (0‑542)

DEDSRS‑FVLSIYTALSKPGVVVFWEDQWFGSEDRPSNSALNVIEDCEIAVIIFSKNYTKSRWCLQELEKITQCQ‑RTDG‑IFLSVFYDVYDLWVRR‑‑D‑FG‑‑‑EDFDRSIEKET‑SE‑DEDKFMTWVAAVTEASKYDELSLRHS‑HEHESELIKIV‑

>G7JSA9 (543‑1714)

GKDCCTKFISHLYTSLQNAGIYTFRDDDEIQRGDRISMSLLKAIGRSRISIVVLSTTYANSRWCMLELVKIMEIG‑RTMDLIVVPVFYEVDPSEVRHQKGKFGKAFEELISTI‑‑‑‑‑‑‑‑‑ESTKSDWRRDLSDIGGIAGIVLID‑‑SRNESEDIKNIV

>G7J7A7 (0‑730)

GEDTRLGFTDHLYAALVRKSIITFRDDEELARGEVISQKLLLAIEESLSAVLIISKNYANSAWCLDELVKILESK‑RLLGQQVFPVFYGVDPSDVRNQRGSFAEAFKKHEEKF‑‑‑‑‑SE‑SKEKVQKWRDALREVANLSGWDS‑KD‑‑QHETKLIEEVI

>G7J7A7 (731‑1664)

GEDTRLGFTDHLYASLVRKSIITFRDDEELARGEVISQKLLHAIEESLSAIVIISKNYADSAWCLDELVKILESK‑RLLGQQVFPIFYGVDPSDVRNQRGSFAEAFKKHEEKF‑‑‑‑‑SE‑SKEKVQRWRDALREVANFSGWDS‑KD‑‑QHETKLIEEVI

>G7JTC9 (0‑340)

AQDRDH‑FIWHLNTVLSKAGTALFGVEERLQY‑‑‑‑ESELDAIVGYCKLAIVVFSSNYNKSISCVQELEKITEC‑‑R‑SD‑VVFPVFFDVYPERSEG‑‑G‑FDNAFHDFDR‑‑‑‑‑I‑KD‑K‑DKFLSWVVGVTKATEYFGPDLYRE‑HEYVQDYIRDIG

>G7JTC9 (341‑1673)

GEDSRAKFMSHLFSSLQNEGIHAFKDDNEIQRGDQISISLLRAIGQSRISIIVLSTNYANSRWCMLELEKIMEIG‑RTKGLIVVPVFYEVAPSEVRDQKGRFGKAFKKLISKI‑‑‑‑‑‑‑‑‑ESKKSNWRRDLFDIGGIAGFVLLG‑‑SRNESADIKNIV

>G7JSB5 (0‑365)

AHDKGY‑FLSSLEEALLEAGINVFGDIKRFQH‑‑‑‑VESVLNVIQDCKVAVVLFSKNYTNSSSCIQELEKITQC‑‑R‑SD‑VVLPVFYQVGP‑‑‑HG‑‑D‑FGDTFHDFDRSM‑EEI‑KE‑E‑DKLMTWVAAITKANKYLGSDLYRE‑HVSITDYIKDIV

>G7JSB5 (366‑1684)

GEDNRAKFMSHLYSSLQNAGIYVFRDDDEIQRGDHISISLLRAIEQSRTCIVVLSTNYANSRWCMLELEKIMEIG‑RNRGLVVVPVFYEVAPSEVRHQEGQFGKSFDDLISKN‑‑‑‑‑‑‑‑‑ESTKSNWKRELFDIGGIAGFVLID‑‑SRNESADIKNIV

>G7JSB4 (0‑1121)

GEDSRAKFISHLHSSLENAGIHVFKDDFKIQRGDQISISLFRAIGQSRICIVVLSKNYANSRWCMLELENIMEIG‑RNRGLVVVPVFYEVDPSEVRHQKGHFGKGFDDLISKT‑‑‑‑‑‑‑‑‑ESTKSNWRRELFDICGISGFVLIN‑‑SRNESADVNSIV

>G7JSB4 (1122‑2026)

SEWSRNSMLSVVYYSTHTKTITSFKDDDNLEPGNKTTISLLKVIADSKVVVVILSKNYTHSRWCLQELEKITQCRTK‑DG‑VVLPVFYDVHPSRILQED‑MYGEAFHDFDRSMKEKT‑SE‑DEDKFMSWVAEISEASKYAAF‑‑LR‑GNQNRGEHITHVV

>G7JSB9 (0‑379)

DQDKHS‑FVFSIYNALRKAGVDVFWENERDGYGDRKPTSVLNVIRDCKVFVIVFSRDYFNSRSCLHEFKKITEC‑‑RKDD‑MVLPVFYDVDLGSWER‑‑G‑FGETLHDCDKLM‑KKT‑KE‑E‑DKFMTWVASISKATIYTGQDL‑‑E‑DRNSSIYIDDVV

>G7JSB9 (380‑1831)

GEDSRAKFISHLYSSLQNAGIYVFKDDDEIQRGDQISISLLRAIGHSRIFIVVLSTNYANSRWCMLELEKIMEIG‑RTGGLVVVPVFYEVDPSEVRRREGQFGKAFEKLIPTI‑‑‑‑‑‑‑‑‑ESTKSNWKRALFDIGSIAGFVLID‑‑SRNESADIKNIV

>G7JSD3 (0‑465)

GDDGSAKFVSHLHSSLQNAGISVFR‑GDEIQQGDDISISLLRAIRHSRISIVVLSINYANSRWCMFELEKIMEIG‑RTGGLVVVPVLYEVDPSEVRHQEGQFGKALEDLILEI‑‑‑‑‑‑‑‑‑ESTKSNWRRDLIDIGGKDGFIVTD‑‑SRNESADIKNIV

>G7JSD3 (466‑1898)

GEDNRPRFISHLHSSLHSAGIYAFKDDDGIQRGDQISVSLGKAIEQSRISIVVLSTNYANSRWCMLELEKIMEVG‑RMNGRVVVPVFYDVDPSEVRHQKGRFGKAFEELLSTI‑‑‑‑‑‑‑‑‑ESTYSNWRRQLFDIGGIAGFVLVG‑‑SRNESAAVKNIV

>G7JDC6 (0‑347)

DKYIGKSFALDLSSALTQAGYAVYINNHDLTSGEQ‑‑‑‑‑‑‑AIKACRTSIIIFSSKFDGSTWFLEEMEKILECR‑RTIKQVFVPVFYDVDPSDVLKQKGVFGEAFVDCIAR‑‑‑‑‑‑‑‑‑‑EDSSIRYRDALFEAANISGFRMMD‑‑TRSQYNEINDIV

>G7JDC6 (348‑1626)

GEDCRAKFISHLYISLQNSGLYVFKDDDGIQRGDQISVALIQAVGQSKISIVVLSKNFANSKWCMTELERIVEIS‑RTKGMVLVPVFYEVDPSEVRHQTGEFGKAFECLLSTK‑‑‑‑‑‑‑‑‑EYTKRNWKAALHEVGSIAGVVILK‑‑SSDESEDIKKIV

>K7KXJ4

GLDTRNNFAALLLQALHRNGIDAFNDNVHVMKGEFIEYELYKAIDGSRNFIVVFSKNYASSTWCLRELARICKN‑IETSRRRILPIFYVVDPLKVQKQSGCYEKAFLDHEERFRGA‑‑KE‑R‑EQVWRWRKALKQVSHLPCLHI‑Q‑‑NELQQAEIEEIL

>G7L5T5 (0‑352)

DKDTSESLASYLYTALTVAGIVVYKDEDKLLNHDQITSSVLHAIAGSRLSIIVFSKLYAVSTCCRQELEKIMECR‑RTTCQIVVPVFYDADPSGVFHQEDLLGEASKYL‑‑‑‑‑‑‑‑‑‑‑‑‑KQRILKKDKLIHEVCNISGFAV‑H‑‑SRNESEDIMKIV

>G7L5T5 (353‑853)

GDDTHAKFISHLYTALENAGIYVFRGDDEIQRGDQVSVSLLQAIGQSRISIIVLSRNYANSRWCMLELENIMGNS‑RTQGMVVVPVFYKIDPTEVRNQSGRFGEDFESLLLRM‑‑‑‑‑‑‑‑‑THKFSNWRRALAEVRGTTGVVIIN‑‑SRNESEDITKIV

>G7L5T5 (854‑2084)

GNDTRAKFISHLYTALENAGIYVFRDDDEIQRGDQISASLLQAIEQSKISIVVLSRSYADSRWCMLELENIMGNS‑RTQGMVVVPVFYEIDPSEVRNQSGKFGEDFESLLLRT‑‑‑‑‑‑‑‑‑TLKLSNWKTALAEVGGTAGVVIIN‑‑SRNESEDIRKIV

>K7KXM9 (0‑301)

GLDTLYGFTGNLYNALYDRGIYTFIDDQERSRGDEIAPALSKAIQESRIAITVLSENYAFSSFRLNELVTILDCK‑S‑EGLLVIPVFYNVDPSDVRHQKGSYGEAMTYHQKRF‑‑‑‑‑KA‑NKEKLQKWRMALHQVADLSGYHF‑KDGDSYEYKFIGSIV

>K7KXM9 (302‑599)

GEDTRYSFTGNLCRALHDSGIHTFVDDEELQRGDEITSELEKEIEDSRFFIIVLSQNYASSSFCLNVLAYILECV‑KRKRLLVLPIFYKVDPSNIGYHRGSFGEALANHEMKFKAKMLEH‑NMEKLEKWKMALHETANFSGYHF‑KQGDGYEYEFITRIV

>K7KXM9 (600‑1238)

GSDTLHGFTGYLYEALHDSGIHTFID‑EDLKRGEEITPAIVKAIEESRIAIIVLSINYASSSSCLDELATILDCL‑KRKRLLVLPVFYNVDHSQVRLQEGSYGEALVKHEESL‑‑‑‑‑KH‑SMEKLEKWKMALHQVANLSDIKI‑KHGARYEYDFIGEIV

>K7KXJ8

GEDTGNNFTAFLLQALRRKGINAFKDDENLKKGEFIEPELREAIEGSRIFIVVFSKNYASSNWCLGELAHICYC‑‑ETSRRPVLPIFYDVDPLEVRKQSG‑YEKAFVEHEERFVEDS‑KK‑‑MKEVHRWREALKQVANFKGWDI‑R‑‑NKALYAEIEQIV

>K7KXP0

GEDTRHGFTGHLYKALCDKGIRAFMEEVDLKRGEEITRTLEEAIKGSRIAITVLSENYASSSFCLNVLAYILECV‑KRKRLLVLPIFYKVDPSNIRYHRGSFGEALANHEMKFKAKMLEH‑NMEKLEKWKMALHETANFSGYHF‑KQGDGYEYEFITRIV

>K7KXP2 (0‑381)

GEDTLNNFTVFLFDALSQNGIDAFKDDTHLQKGESIAPEVLQAIEESLLFLVVFSKNYASSTRCLRELAHICNCTDEASPSRVLPVFYDVDPSEVRKQSGYYGIAFAEHERRFRED‑‑IE‑KMEEVLRWREALTQVANISGWDI‑R‑‑NKSHPAMIKEIV

>K7KXP2 (382‑743)

GEDTRYSFTGNLCRALRDSGIHTFVDDDELQRGDEITSELEKEIEDSRFFIIVLSQNYASSSFCLNVLAYILECV‑KRKRLLVLPIFYKVDPSSIRFHGGSFGEALANHEMKFKAKMLEH‑NMEKLEKWKMALHETANFSGYHF‑KQGDGYEYEFITRIV

>K7KXP2 (744‑1382)

GSDTLHGFTGYLYKALHDRGIHTFID‑EDLKRGEEITPEIVKAIEESRIAIIVLSINYASSSFCLDELATILDCL‑ERKRLLVLPVFYNVDHYQV‑‑LGGSYVEALVKHGKSL‑‑‑‑‑KH‑SMEKLEKWEMALYEVADLSDFKI‑KHGARYEYDFIGEIV

>947078674

G‑GTSNPFVDPLCRALRDKGISIFRSED‑‑‑‑‑GETRPA‑IEEIEKSKMVIVVFCQNYAFSTESLDELVKIRE‑Y‑VNRRKQVWTIFYIVEPSDVRKQRNSYKDAMNGHEMTY‑‑‑‑‑GK‑DSEKVKAWREALTRVCDLSGIHC‑KD‑HMFEAEL‑QKIV

>947059181

GSDTRYGFTGHLYKALCDRKVRTFIDDEELQRGEQITPSLLKAIEESRIFIPVFSKNYASSTFCLDELVHIFACV‑KEKSRLVLPVFYEVDPSHVRHQRGSYKEALNSHKKRFNDD‑‑QE‑‑‑‑KLQKWRNALSQAANLSGYHF‑KQG‑EYEYDFIAKIV

>947107202

GEDTRNNFTAFLFDSLSQNGIHAFKDDTHFPKGESIAPELLQAIEESRLFLVVFSKNYASSTWCLRELAHICNCTIEASPSRVLPIFYDVDPSEVRKQSGYFGIDFAEHEERFRED‑‑KE‑EMEEVQRWREALTQVAHLSGWDI‑R‑‑NKSQPAMIKEIV

**Aligned Malpighiale sequences from Pfam used to build profile HMM after alignment pruning and exclusion.**

>B9I808

GKDTRNNFTSHLYSNLVQRGIDVYMDDRGLERGKTIEPALWKAIEDSRFSIVVFSRDYASSPWCLDELVKIVQCMK‑EMGHTVLPVFYDVDPSEVADQKGNYKKAFIEHKEKLSEN‑‑LDRVKCWSDCLSTVANLSGWDVRNS‑‑‑DESQSIKKIA

>B9INW3

GEDTRKTFTDHLYTALVQAGIRAFRDDDDLPRGEEISDHLLRAIQESKISIVVFSKGYASSRWCLNELVEILECKKRKTGQIVLPIFYDIDPSDVRKQTGSFAKAFDKHEKRFEEK‑‑‑‑LVKEWRKALEDAANLSGRSLNNMANGHEAKFIKKII

>U5FYE6

GEDTRRKFTDHLYTALVQAGIHTFRDDDEIQRGHNIELEIQKAIQQSKISIIVFSIDYARSRWCLDELVMIMERKR‑TTNSIVLPVFYDVDPSQVRNQTGSFAAAFVEHEKRFKEE‑‑MERVNGWRIALKEVADLGGMVLGD‑‑‑GYEAQFVQSIV

>B9HZC1

GEDTRKNFTDHLYTALVQAGIHTFRDDNEIRRGENIDFELQKAIQQSKISIIVFSKDYASSRWCLDELVMIMERKR‑NADCIVLPIFYDVDPSQVGRQTGSFSAAFVEHEKSFNKE‑‑MERVNGWRIALKEVADLAGMVLGD‑‑‑GCEAPFVQSIV

>U5FES9

GEDTRVGFTSHLHAALDRKQILTFIDYQ‑LVRGDEISASLLRTIEEAKLSVIVISENYASSKWCLEELAKIIERRR‑NNRQIVIPVFYKVDPSHVRNQTGSFGDAFAR‑‑‑LIRNKLTLEEVQSFREALTDAASLSGWNLGNS‑‑DLESEFIEKIV

>U5GDG4

GTDTRNSFTSHLYDALQRNQIDAYIDNK‑LDGGEKIEPALLERIEESFISLVIFSENYADSTFCLRELSKILECME‑TKQQMVLPVFYQLDPSHVQNLTGSYGDALCKHERDRS‑‑‑‑SEEVESWRRALKEIANLKGWDSNVI‑‑KDETKLIQEIV

>U5G9F5

GEDTRKTFTDHLHTALVQAGIHTFQDDDELPRGEEISDHLLKAIRESKISTVVFSKGYASSRWCLNELVEILKCRKRKTGQIALPIFYDIDPSDVRKQTGSFAEAFVKHEERSKEK‑‑‑‑‑VKEWRETLEEAGNLSGWNLKDMANGHEAKFIQEII

>U5FXY6

GEDTRKNFTDHLYKALIHAGFHTFRDDDEIRRGKNIRLELQKAIKQSKIAIIVFSKNYAWSKWCLDELVKIMERKR‑NAECIVFPVFYHVDPSEVRNQTGSFAAAFVEHEKHYKEK‑‑MERVNGWRIALKEVANLAGMDLGD‑‑‑GYEAQFVQSIV

>U7DUN2

GKDTRNNFTSHLCKDLRRQKIKTFIDDR‑LERGEEITPALLKTIEESRVSIVIFSENYASSPWCLDELVKILECKE‑TYGQIVLPVFYHVDPSDVDEQTGSFGNAFSELEKNFKGK‑‑MGKVPRWRADLTYAASISGWDSQVT‑‑SPEAKLISEVV

>U5GDK8

GTDTRNSVTSHLYDALQRNQIDAYIDDK‑LDRGEKIEPALLERIEESCISLVIFSENYADSTFCLRELSKILECME‑TKQQMVLPVFYRLDPSHVQNLTGSYGDALCKHERDCS‑‑‑‑SEEVQSWRHALKEIANLKGWDSNFN‑‑KDETELIQEIV

>B9IQX2

EQDTLAGFTSHLYAALDRKQILTFIDYQ‑LVRGDEISASLLRTIEEAKLSVIVFSENYASSKWCLEELVRIFECRK‑NNGQIVIPVFYKVDPTHLRHQTGSFGDAFAR‑‑‑LIRNKLTLEEVQSFRDALTDAANLSGWSLGNS‑‑GPESEFIEKIV

>U5GRB6

GEDTRKNFTDHLFTALQKAGIRTFRDDDELRIGEEISFQLPKAIQESKISIVVFSKGYASSTWCLDELEKILDC‑RQPTGQIVLPVFYDIDPSDIRKQTGSFAEAFDRHEERFKEE‑‑MEKVQKWRKALVEAANISGLDLRSFANGHESKLIQKIV

>U5GDJ5

GADTRKGFTSHLYHVLQRNQIDAYIDNK‑LDGGEKIEPALLERIEESFISLVIFSENYADSTFCLRELSKILECME‑TKQQMVLPVFYRLDPSHVQNLTGGHGDALCKHERECS‑‑‑‑SEEVESWRHALKEIASVKGWDSNVI‑‑KDETKLIQEIV

>U5FIF6

GKDTRNNFTSHLYDATRRKKIKTFIDDG‑LERGEEITPALLKTIEESRISVVIFSKNYASSPWCVDELVKILECKE‑TYGQIVLPVFYHVDPSDVDEQTGSFGNAFAELEIFFKGK‑‑MDKVPRWRDDLRKAASISGWDSQVT‑‑SPESTLVREVV

>U5FE91

GEDTRKTFTDHLYAALDQAGIHTFRDDDELPRGEEISEHLLKAIRESKISIVVFSKGYASSRWCLNELVEILKCKRKKTGQIVLPIFYDIDPSDVRKQTGSFAEAFDKHEERFEEK‑‑‑‑LVKEWRKALEDAGNLSGWSLNDMANGHEAKFIKEII

>U5GJU3

GEDTRKNFTDHLFTALQKAKVRTFRDDDELRIGEEISLQLPKAIQESKISIVVFSKGYASSTWCLDELEKILDC‑KHTTGQIVIPVFYDIDPSDIRKQTGSFAEAFDKHEERFKEE‑‑MEKVHKWRKALVEAADLSGLDPHSIANGHESKLIQKIV

>U5FEH3

GEDTRKTFTDHLYTALVQAGIHTFRDDDELPRGEEISDHLLRAVQESKISIVVFSKGYASSRWCLNELVAILKCKRKKRGQIALPIFYDIDPSDVRKQNGSFAEAFVKHEERFEEK‑‑‑‑LVKEWRKALEEAGNLSGWNLNDMANGHEAKFIKEII

>B9HZC8

GKDTRKNFTDHLYTALVQAGIHTFRDGNEIWRGENIDVELQKAIQQSKISIIVFSKDYASSRWCLDELVMIMERKR‑NADCIVLPVFYDVDPSQVGRQTGSFSAAFVEHEKSFNEE‑‑IERVNGWTIALKEVADLAGMVLGD‑‑‑GYEAPFVQSIV

>U5G374

GEDTRKSFTDHLYTALCHRGVITFRDDQELERGNEISRELLQAIQDSRFSVIVFSRNYTSSTWCLNELVKIVECMK‑QGRQTVIPVFYDVDPSEVRNQTGRLQQAFADHEEVFKDN‑‑IEKVQTWRIAMKLVANLSGWDLQDR‑‑‑HESEFIQGIV

>U5GME6

GEDTRKKFTDHLYTALIHAGIHTFRDNDELPRGEDISSIISRPIQESRIAIVVFSKGYASSTWCLGELSEILACKS‑AIGQLAVPIFYDIDPSDVRKQTASFAEAFKRHEERFKEN‑‑IEMVNKWRKVLVEAANLSGWHLQEMENGHEAKFIEKMV

>U5FE61

GEDTRKNFTDHLYFALKDAGINTFRDDNELRSGEDISTELLQAIQKSRISVILFSRNYANSRWCLEGLVKIMECWR‑SWRQLVFPIFYDVDPSDVRKQTGSFAEAFSGHEER‑‑‑‑‑‑‑‑‑‑‑‑‑‑‑‑‑‑‑‑‑‑‑‑‑‑‑‑‑‑‑‑‑‑HEAKFIKKIV

>B9HZ91

GEDTRKNFTDHLYTALVQAGIHTFRDDDEIGRGENIESELQKAIQQSKIAIIVFSKDYASSRWCLDELVMIMERRR‑TADCRVLPVFYDVDPSQVRKQTGSFAAAFVEHEKRFKEE‑‑MERVNGWRIALKEVADLAGMVLGD‑‑‑GYEALLVQCIV

>U7E1S2

‑‑‑‑‑‑‑‑TSHLYDAMRRKKIKTFIDDG‑LERGEEITPALLKSIEESRISVVIFSKNYASSPWCVDELVKILECKE‑TYGQIVLPVFYHVDPSDVDEQTGSFGNAFAELEKNFKWK‑‑MDKVPRWRADLTYAASISGWDSQVT‑‑SPEAKLIREVV

>U5FFQ3

GEDTRKTFTDHLYAALDQAGIRAFRDDDELPRGEEISDHLLRAIQESKISIVVFSKGYASSRWCLNELVEILECKKRKTGQIVLPIFYDIDPSDVRKQNGSFAEAFVKHEERFEEK‑‑‑‑LVKEWRKALEEAGNLSGWNLNDMANGHEAKFIKEII

>U5FG45

GKDTRNNFTSHLYDALCRKKIKTFIDDR‑LERGGEITPALLKTIEESRISVVIFSKNYASSPWCVDELVKILECKE‑TYGQIVLPVFYHVNPSDVDEQTGSFGNAFAELEKNFKGK‑‑MDKVPRWRADLTNAASISGWDSQVT‑‑SPESKLVTDVV

>B9I2I8

GEDTRDSFTKHLYDSLNKQEIRVFLDASGMIQGDEIAPTLMEAIQDSASSIIILSPRYANSHWCLEELARICELRR‑‑‑‑‑LILPVFYQVDPSNVRRQKGPFEQDFESHSKRF‑‑‑‑GDDKVVKWRAAMNKVGGISGFVFDTS‑‑‑GEDHLIRRLV

>Q2XPG2

GEDTRKTFTDHLYTALVQAGIHTFRDDDELPRGEEISDHLLRAIQESKISIVVFSKGYASSRWCLNELVEILKCKNRKTGQIVLPIFYDIDPSDVRKQTGSFAEPFDKHEERFEEK‑‑‑‑LVKEWRKALEEAGKLSGWNLNDMANGHEAKFIKEII

>U5G975

HQEIGKNFADHLYKDLNYAGIRTFRDDGGIYTGQ‑‑KSDVKRAIQESRISVVVFSKDYASSTKCLDQLGLIMDARR‑TTGLVVLPVFYNADPSEVWEQKGLFEEAFAKHEKSFHKE‑‑MARVESWRAALKEAADLKGKERKQD‑‑RYESKFIESIV

>U5FF36

GEDTRFDFTSHLHAALKRKQILTFIDDQ‑QVRGDEIPESLLRTIEEAKLSVPVFSENYASSKWCLEELVKIFERRK‑NNGQIVIPVFYKVNPSHVR‑‑‑‑SFRDAFAG‑‑‑LIKNKLTEYKEKSFRDALTDTANLSGWTLGKS‑‑EPESEFIDKIV

>U7E104

GEDTRKNFTDHLYTALQNAGIHTFRDDNELPKGEEISSHLLKAIKESKISIVVFSKGYASSTWCLDELSEILDCRQ‑TAGQIVLPVFYDIDPSDIRKQTGSFAEAFDRHEERFKEE‑‑MEKVQKWRKALVEAGRLS‑‑‑‑‑‑‑‑‑GHESKLIQMIV

>B9HZD5

GEDTRKNFTDHLYKALVDAGFHTFRDDDEIRRGKNIELELQKAIQQSKIAIIVFSKNYAWSRWCLDELVMIMERKR‑NADCIVFPVFYHVDPSEVRNQTGSFAAAFVEHEKHYKEE‑‑MERVNGWRIALKEVANLAGMDLGD‑‑‑GYEAPFVQSIV

>B9GWM3

GEDTRKNFTDHLYTALHHARIHAFRDDDELRRGEEISLQLLKAIQESKISIVVFSKGYASSTWCLAELEKILDC‑RHTTGQIVLPVFYDIDPSDIRKQTGSFAEAFDRHEERFKEE‑‑MEKVQKWRKALMEAANLSGLDLRSFANGHESKLIQKIV

>U5FFC1

GEETRKTFTGHLYAALDEAGIHTFLDDVELPRGEEISEHLLKAIRESKISIVVFSKGYASSRWCLNELVEILKCKRKKTGQIVLPIFYDIDPSDVRKQTGSFAEAFDKHEERFEEE‑‑‑‑LVKEWRKALEDAGNLSGWSLNDMAYGHEAKFIRGII

>U5FHF5

GEDTRKTFVDHLYTALVQAGIHTFRDDDELPRGEEISEHLLEAIRESKISIVVFSKGYASSRWCLNELVEILKCKRKKTGQIVLPIFYDIDPSDVRKQTGSFAEAFDKHEERFEEK‑‑‑‑LVKEWRKALEDAGNLSGWSLNDMANGHEAKFIKGII

>B9INY7

GADTRKTFLGHLYNALVQAGIHTFKDDEELPPGEEISQQLKKAIQESKISIVVFSRDYASSRWCLNELVEILECRN‑TKGRTVFPIFCGVDPSHVRKQEGSFKKAFKAYEN‑‑KEE‑‑KEKINKWKNALKDAANLSGKDIYSTANGDESVLIKKIV

>U7DXH7

GEDTRRNFTDHLYKALSREGIPTFRDDDGIRRGENIELEIKKAIQETKLSIIVFSKEYASSRWCLDELEMIMERRR‑TVGHIVFPVFYDVDPSEVGTQTGRYGEEFAKHEIHFKD‑‑‑‑‑RVEGWRKALKEVAYMEGMVLED‑‑‑GYESKFIESIV

>U5FFT0

GQETRNTFTAHLYHALCNKGINAFIDD‑KLERGEHITSQLNQIIEDSRISLVIFSENYARSIYCLDELVKILECKE‑SKGQVVLPVFYNVDPSDVEEQKGSFGESLDFHETYLGIN‑‑AEQLKQWREALTKAAQLSGWH‑LDR‑‑GNEAVFIRKIV

>B9HZ97

GEDTRKNFTDHLYTALVQAGIHTFRDDDEIGRGENIESELQKAIQQSKIAIIVFSKDYASSRWCLDEIVMIMERRR‑TADCRVLPVFYDVDPSQVRKQTGSFAAAFVEHEKHFKEE‑‑MERVNGWRIALKEVADLAGMVLGD‑‑‑GYEALLVQCIV

>U5FIR8

GEDTRFDFTSHLYAALNRKQILTFIDYQ‑LVRGDEISASLLRTIEEAKLSVIVFSENYASSKWCLEELAKIFERRK‑NNGQIVIPVFYQVDPSHVRNQTGSFGDAFAR‑‑‑LIKKKLTMDKEQSFRDALKDTSTLSGWTVGNS‑‑ELESEFIEKIV

>U7E153

GEDTRKNFTDHLYTALQKAGILTFQDDDELPKGEEISSHLLKAIKESNISIVVFSKGYASSTWCLDELSEILDCRQ‑TARQIFLPVFYDIDPSDIRKQTGSFAEAFDRHEERFKEE‑‑MEKVQKLKNALVEAASLS‑‑‑‑‑‑‑‑‑GHESKLIQMIV

>U5FEN2

GKDTRDNFVSHLRDALCRKQIKTFIDDK‑LERGEEITGALLRTIEESRISVIIFSRNYASSPWCVDELVKILECKK‑AYGQIVLPVFYHVDPSDVDQQTGSFGNAFAELERNFKQK‑‑MDKVPRWRADLTSAANISGWDSQVT‑‑RPESSLVEQIV

>U7E235

GQDTRKNLTDHLYTALHHARIHAFRDDEKLRRGEEISLQLSKAIQESKISIVVFSKGYASSTWCLGELQKILECRRQPTGQIVLPVFYDIDPSDIRKQTGSFAEAFDRHEARFKEE‑‑MEKVQKWRKALVEAANLS‑‑‑‑‑‑‑‑‑GHESKLIQKIV

>U7E0N2

GEDARKNFTDHLYFALKDAGINTFRDDNGLRRGEDISTELLQAIQKSRISVIVFSRNYANSRWCLEELVKIMECRR‑SFRQLVFPIFYDVDPSDVRKQTGSFAEAFAGHEERFQTD‑‑KGKVATWRMALTEAANLSGWDLRNVADGHEAKFIKKIV

>U5FIU6

GEDTRGGFTSHLYAALDRKQIRAFIDYQ‑LRRGDEISASLLRTIEEAKLSVIVFSENYASSKWCLEELAKIIERRR‑NNGQIVIPVFYKVDPSHVRNQTRSFGDALAR‑‑‑LIKKKLTMDKEQSFRDALTAAANLSGWSLGNS‑‑ELEFEFIKNIV

>U5FJU0

GEDTRVGFTSHLHAALERKNILTFIDND‑LRRGEEISPSLVKAIEDSMLSVIIFSQNYASSKWCLDELLKILESRK‑VRGQIAIPVFYEVDPSDIRKQSGSFGDVFAQ‑‑‑LVKRKLKMEEEQCFRAALNEAANISGHDSRKI‑‑ESESKFIEVIV

>U5G3U7

GEDTRKTFTDHLYTALVQAGIQTFRDDDELPRGKEISQHLLEAIQESKISIVVFSKGYA‑SRWCLDELVEIIKCKYRKTGHIALPIFYDIDPSDVRKQTGSFAEAFVKHEERSKEK‑‑‑‑‑VKEWREALEEAGNLSGWNLKDMANGHEAKFIQEII

>U7DZF6

GKDTRNNFTSHLYSNLKQRGIDVYMDDRELERGKTIEPALWKAIEESRFSVIIFSRDYASSPWCLDELVKIVQCMK‑EKGQTVLPVFYDVDPSEVAEQKGKYKKAFVKHEKDFKEN‑‑LDRVRSWKDCLSTVANLSGWDIRNR‑‑‑NESEHIEKIV

>U5FJZ2

GKDTRNNFTSHLCSNLAQRGIDVYVDDRELERGKTIEPALWKAIEESRFSVIIFSRDYASSPWCLDELVKIVQCMK‑ETGHTVLPVFYDVDPSEVAEQKGQYEKAFGEHEQNFKEN‑‑LEKVRNWKDCLSTVANLSGWDVRDR‑‑‑NESESIKIIA

>U5FHF8

GEDTRFDFTSHLYAALNRKQILTFIDYQ‑LVRGDEISASLLRTIEEAKLSVIVFSENYASSKWCLEELAKIFERRK‑NNGQIVIPVFYQVDPSHVRNQTGSFGDAFAR‑‑‑LIKKKLTMDKEQSFRDALKDTATLSGWTLGNS‑‑QLESEFIEKIV

>U5FEA9

GEDNRKNFTDHLYTALVQAGIYTFRDHNEIPRGEEISKHLLKAIQESKISIVVFSKGYASSRWCLNELVEILECKNRKTGQIVLPVFYDIDPSDVRKQTGSFVKAFDKHEDCFKEK‑‑‑‑‑VKEWRKALEETGNLSGWNLSDMENGHESKFIQDII

>U5GNQ5

GADTRKNFTGHLYMALQGAGIRTFRDEDEIEGGEHIGFKITKAIQESKMSLVVFSRDYASSKWCLEELLMIMKRRE‑TIGHIVLPVFYEVDPDDVSMQTGFFAEAFASHEKNFMDN‑‑‑RDMEEWREALRKVADLKGPVLRDR‑‑‑YEAQFIQDIV

>B9HGH2

GKDTRKTFTDHLYTALVQAGIHTFRDDDELPRGEEISQHLLEAIQESKICIVVFSKGYASSRWCLDELVEILKCKYRKTGQIALPIFYDIDPSDVRKQTGSFAEAFVKHEERSEEK‑‑‑‑‑VKEWREALEEAGNLSGWNLKDMTNGHEAKFIQHII

>U5FG49

GEDTRKTFTDHLYTALVQAGIHTFRDDDELPRGEEISDHLLRAVQESKISIVVFSKGYASSRWCLNELVEILKCKNRKTGQIVLPIFYDIDPSDVRKQNGSFAEAFVKHEERSEEK‑‑‑‑LVKEWRKALEEAGNLSGRNLNDMANGHEAKFIKEII

>B9HF56

GEDTRKNFTDHLHTALVQAGFHTFRDDDEIQRGENIEIEIQKAIKESRMSIIVFSKDYASSRWCLDELVMIMELKK‑LGGHVVLPIFYDLDPSHVSNQTGSFAEAFVRHEERFKKE‑‑‑DRVEGWRMALKEVADLGGMVLQD‑‑‑GYESKFIQNVV

>U5FRN8

GKDTRNNFTSHLYSNLVQRGIDVYMDDRGLERGKTIEPALWKAIEDSRFSIVVFSRDYASSPWCLDELVKIVQCMK‑EMGHTVLPVFYDVDPSEVADQKGNYKKAFIEHKEKLSEN‑‑LDRVKCWSDCLSTVANLSGWDVRNS‑‑‑DESQSIKKIA

>B9N1N5

GEDTRVCFVSHLYAALKRKQISTFIDYK‑LNRGEEISPSLLKAIEDSKLSVVVFSDNYASSKWCLEELAKILECKK‑VKGQMVIPVFYRVDPSHVRNQTGSFADAFARHDQLLKEK‑‑MEKVLNWRAAMREAANLSGWDSHNI‑‑KSESEFVDDIV

>U5FF28

GEDTRFDFTSHLYAALNRKQILTFIDYQ‑LVRGDEISASLLRTIEEAKLSVIVFSENYASSKWCLEELAKIFERRK‑NNGQIVIPVFYQVDPSHVRNQTGSFGDAFAR‑‑‑LIKKKLTMDKEQSFRDALKDTATLSGWTLGNS‑‑HPESQFIEKIV

>B9IQ82

GQDTRNNFTSHLYDALCRKKIKTFIDNG‑LERGEEITPALLKTIEESRISVVIFSKNYASSPWCVDELVKILECKE‑TYGQIVLPVFYHVDPSEVDEQTGSFENAFAELEKNFKGK‑‑MDKLPRWRAGLTYAASISGWDSQVT‑‑SPESKLVREVV

>Q2XPG3

GEDTRKTFTDHLYTALVQAGIHTFRDDDELPRGEEISDHLLRAIQESKMSIVVFSKGYASSRWCLKELVEILKCKNGKTGQIALPIFYDIDPSDVRKQTGSFAEAFVKHEERFEEK‑‑Y‑LVKEWRKALEEAGNLSGWNLNDMANGHEAKFIKEII

>U5GAC7

GTDTRNSFTSHLYDALQRNQIDAYIDNK‑LDGGEKIEPALLERIEESFISLVIFSENYADSTFCLRELSKILECME‑TKQQMVLPVFYRLDPSHVQNLTGSYGDALCKHERDCS‑‑‑‑SEEVESWRRASKEIANLKGWDSNVI‑‑KDETKLIQEIV

>U5FE89

GKDTRKTFTDHLYTALVQAGIHTFRDDDELPRGEEIHDHLLRAIQESKISIVVFSKGYASSRWCLNELVEILKCKNRKTGQIVQPIFYNIDPSDVRKQNGSFAKAFVKHEERFEEK‑‑‑‑LVKEWRKALEEAGNLSGWNLNDMANGHEAKFIKEII

>U5GA93

GTDTRNSFTSHLYDALQRNQIDAYIDNK‑LDGGEKIEPALLERIEESFISLVIFSQNYADSTFCLRELSKILECME‑TKQQMVLPVFYRLDPSHVQNLTGSYGDALCKHERDCS‑‑‑‑SEEVESWRHALKEIANLKGWDSDVI‑‑KDETKLIQEIV

>U5FZ55

GEDTRNNFTDHLYKALVQAGIHTFRDDDEIRIGENIELELQKAIQQSKISIIVFSKNYAWSRWCLDELVKIMERKR‑NAACIVYPVFYHVDPSEVRNQTGSFAVAFVEQDKRFKEE‑‑MDRVNGWRIALKEVADLAGMDLGD‑‑‑GYEAQFVQSIV

>B9H393

GADTRNSFTSHLYKALCQNQIHAYIDYK‑LHGGEKIEPALLERIEESYISVVIFSENYADSTFCLRELSKILECME‑TKGQKVLPVFHQLDPSHVQDLTGSYGDAICKHESDCS‑‑‑‑SQEVESWRHASKEIANLKGWDSKVI‑‑RDETKLIEEIV

>U5FEH7

GEDTRKTFTDHLYAALVQAKIHTFRDDDELPRGEEISDHVLRAIQESKISIVVFSKGYASSRWCLDELVEILKCKRKKTGQIVLPIFYDIDPLDVRKQTGRFAEAFVKHEERFEEK‑‑‑‑LVKEWRKALKEAGNLSGWNLNDMANGPEANFVKEII

>B9HZ86

GEDTRKNFTDHLYTTLVQAGIHTFRDDNEIRRGENIDFELQKAIQQSKISIIVFSKNYAWSRWCLDELVMIMERRR‑TTGSIVFPVFYDVLPSEVRNQTGSFAAAFVEQEKRFKEE‑‑MERVNGWRIALKEVADLAGMVLGD‑‑‑GYEAQFVQSIV

>U5GA98

GTDTRYSFTSHLYDALQRKQIDAYIDDK‑LDGGEKIEPAILERIEESFISAVIFSENYADSTFCLRELSKILECME‑TKQQMVLPVFYRLDPCQVQNLTGSYGDALCKHEKDCG‑‑‑‑SKEVESWRHASKEIANLKGWNSNVI‑‑KDEIKLIEEIV

>U5FF18

GEDTRKNFTDHLYFAFKDAGINTFRDDNELRRGEDISTELLQAIQKSRISVIVFSENYANSRWCLEELVKIMECRR‑SCRQLVFPIFYDVDPSDVRKQTGSFAKAFAGHEERFQTD‑‑KGKVATWRMALTEAANLSGWDLRNVADGHEAKFIKKIV

>U5FVE5

GEDTRKNFTDHLYKALVDAGIHTFRDDDEIQRGENIDFELQKAIQQSKISIIVFFKDYASSRWCLDELVMIMERKR‑NDDCIVLPVFYDVDPSQVGRQTGSFAAAFVEHEKSFNEE‑‑KERVSGWRIALKEVADLAGMVLGD‑‑‑GYEAQFVQSIV

>B9IQ77

GEDTRNNFTSHLYDALCRKKIKTFIDDG‑LERGEEITPALLKKIEESRISVVIFSKNYASSPWCVDELVKILECKE‑TCGQIVLPVFYHVDPSDVDEQTGSFGNAFSELENIFKGK‑‑MDKVPRWRADMTYAASISGWDSQVT‑‑SPESKLVREVV

>B9IQ03

GKDTRKTFTNHLYTALVQAGIHTYRDDDELPRGEEISDHLLRAIQKSKISIPVFSKGYASSRWCLNELLEILKCKNRKTGQIVLPIFYDIDPSDVRKQNDSFAEAFVKHEKRFEEK‑‑‑‑LVKEWRKALEEAGNLSGWNLNAMANGYEAKFIKKII

>B9HZD8

GEDTRKNFTDHLYKALVDAGFHTFRDDDEIRRGKNIQLELQKAIQQSKIAIIVFSKNYAWSRWCLDELVKIMERNR‑NADCIVFPVFYHVDPSEVRNQNGSFAAAFVEHEKHYKEE‑‑MERVNGWRIALKEVANLAGMDLGD‑‑‑GYEAQFVQSIV

>U7E249

GKDTRNNFTSHLYYNLAQRGIDVYMDDRGLERGKTIELALWKAIEESRFSVIIFSRDYASSPWCLDELVKIVQCMK‑EKGHTVLPVFYNVDPSET‑‑‑‑‑‑YEKAFVEHEQNFKEN‑‑LEKVRNWKDCLSTVANLSGWDVRNR‑‑‑NESESIKIIA

>B9INW6

GEDTRKTFTDHLYSALVQAGIHAFRDDDDLPRGEEISDHLLRAIQESKISIVVFSKGYASSRWCLNELVEILECKNRKTGQIVLPIFYHIDPSDVRKQNGSFAEAFANNEERFEEK‑‑‑‑LVKEWRKALEEAGNLSGWNLNHMANGHEAKFIKEII

>U5GJU8

GEDTRKNFTDHLYTALLQAGIHTFRDDEKLRRGEEISFQLSKAIQESKISIVVFSEGYASSTWCLGELQKILDC‑RHTTGQIVLPVFYDIDPSDIRKQTGSLAEAFGKHEESFKEE‑‑MEKVQKWRKALLEAANLSGLDRRSIANGHESKLIQKIV

>U5FFB8

GEDTRKTFTDHLYTALVQAGIHTFRDDDELPRGEEISDHLLRAIQESKISIVVFSKGYASSRWCLNELVEILKCKNRKTGQIVLPIFYDIDPSYVRKQDGSFAEAFVKHEERFEEK‑‑‑‑LVKEWRKALEEAGNLSGWNLNDMANGHEAKFIEGII

>U7E062

GPDTRKNFTDHLYKALIQAGIHTFRDVDEIRRGENIDFELQKAIQQSKISIIVFSKDYASSRWCLDELVMIMERKR‑NDDCIVLPVFYDVDPSQVGRQTGSFAAAFMEHEKHFNEE‑‑KERVSGWRIALKEVADLAGMVLGD‑‑‑GYEAQFVQSIV

>U5FFQ2

GEDTRKTFTDHLYTALVQAGIHTFRDDDELSRGEEISKHLLRAIQESKISIVVFSKGYASSRWCLNELVEILKCKNRKTDQIVLPIFYDIDPSDVRKQNGSFAEAFVKHEERFEEK‑‑‑‑LVKEWRKALEESGNLSGWNHNDMANGHEAKFIKEVV

>B9IE71

GEDTRHGFTKNLYDSLSKQDIRVFLDDSGMTQGDEIAPTLMEAIEDSALSIIILSPRYANSHWCLEELARICELRR‑‑‑‑‑LILPVFYQVDPSHVRRQKGPLEQDFMNHMERF‑‑‑‑GEEKVGKWREAMYKVGGISGFVFDTR‑‑‑SEDQLIRRLG

>U5GA00

GTDTRYSFTSHLYDALQRKQIDAYIDDK‑LDGGEKIEPAILEGIEESFISVVIFSENYADSTFCLRELSKILECME‑TKQQMVLPVFYRLDPCQVQNLTGSYGAALCKHEKDFG‑‑‑‑SKEVESWRHALKEIANLKGWNSKVI‑‑MDEIKLIEEIV

>U5FGD1

GEDTRVGFTSHLYAALKREQILTFIDNQ‑LVRGDEISASLLRTIEEAKLSVIVFSANYASSKWCLEELAKIFERRK‑NNGQIVIPVFYQVDPSHVRNQAGSFGDALAR‑‑‑LIKEKLTMDTEQSFGDALTDAANLSGWRLGNS‑‑ELEAEFIEKIV

>U5FN17

GKDTRNNFTSHLCSNLAQRGIDVYVDDRELERGKTIEPALWKAIEESRFSVIIFSRDYASSPWCLDELVKIVQCMK‑ETGHTVLPVFYDVDPSEVAEQKGQYEKAFGEHEQNFKEN‑‑LEKVRNWKDCLSTVANLSGWDVRDR‑‑‑NESESIKIIA

>U5GKK7

GEDTRKNFTDHLYSTLSKAGIVTFRDDNSIQRGENIELEIEKAIQESQMSVVVLSKDYASSTWCLDELVMIMDRKR‑TAGHIVLPVFYDVDPSQVGEQTGNYAEAFAKHQDHFQDD‑‑MERVEKWKATLKEVAYLGGMVLQDR‑‑‑HESQFIGDIV

>U5GDH5

GTDTRNSVTSHLYDALKRNHIDAYIDNK‑LDGGEKIEPALLERIEESCISLVIFSEKYADSTFCLRELSKILECKE‑TKGQMVLPVFYRLDPSHVQNLTGSYGDALCRHERDCC‑‑‑‑SQEVESWRHASKEIANLKGWDSNVI‑‑KDETKLIQEIV

>U5GDL2

GADTRKGFTSHLYDALKRSQIDAYIDNK‑LDGGEKIEPALLKRIEESFISLVIFSENYADSTFCLRELSKILECME‑TKQQMVLPVFYRLDPSHVQNLTGSYGDALCKHERDCS‑‑‑‑SEEVESWGHALKEIASVKGWDSNVI‑‑KDETKLIQEIV

>U5FHD1

GEDTRLGFTSHLHAALDRKQILTFIDDQ‑LERGDEISASLLRTIEEAKLSVIVFSANYASSKWCLEELVKIFECRK‑NNGQIVFPVFYKVDPTHVRNLTGSFGGAFAR‑‑‑LIRNKLTLEEVQSFRDALTDAASLSGWNLGNS‑‑ELEAEFIEKIV

>B9IP51

GADTRKTFLGHLYNALVQAGIHTFKDDEELPPGEEISHHLKKAIQESKISIVVFSRDYASSRWCLNELVEILECRN‑TKGRTVFPIFCGVDPSHVRKQEGSFKKAFKAYEN‑‑KEE‑‑KEKIDKWKNALKDAANLSGKDIYSTANGDESVLIKKIV

>U7E0N6

GEDTRKNFTDHLYFALKDAGINTFRDDNELRSGEDISTELLQAIQKSRISVILFSRNYANSRWCLEGLVKIMECWR‑SCRQLVFPIFYDVDPSDVRKQTGSFAEAFSGHEERFQTD‑‑KGKVATWRMALTEAANLSGWDLRNVADGHEAKFIKKIV

>U5FG53

GEDTRQTFTDHLYTALVQAGIHTFRDDDELPRGEEISDHLLRAIHESKISLVVFSKGYASSRWCLNELVEILQCKNRKTNQIVLPIFYDIDPSDVRKQNGSFAEAFVKHEERFEEK‑‑‑‑LVKEWRKALEEAGNLSGWNLNDMANGHEAKFTKEII

>U5GA82

GFDTRNSFTSHLYDALQRNQIDAYIDNK‑LDGGEKIEPALLKRIEESFISLVIFSENYADSTFCLRELSKILECME‑TKQQMVLPVFYRLDPSHVQNLTGSYGDALCKHEKDCS‑‑‑‑SEEVKSWRRALKEIANLKGWDSDVI‑‑KDETKLIQEIV

>B9S9D9

GLDTRNGFVSHLFKALSEKQIITFKDEN‑LDRGEQISDTLSQTIKESYVSVVIFSKNYACSAWCLDELVTILQCNK‑EMGQVVLPVFYEIDPTEVQELTGSYGNALMNHRKEFENCL‑‑‑‑VESWSHALMEIAAMAGFVSWNT‑‑KPESKLIDEIA

>B9RM27

GTDTRNNFTSHLYSALIRCGIITFIDTR‑LERGEGIESAILKAIEESIISVVILSKNYASSPWCLDELVKIFECRD‑KQGQKIIPVFYHVDPTELDNQTGSFGEALAKHEQDFNEI‑IMDKVPNWRIVLSRAANIAREVMTPS‑‑RVESELIEVVL

>B9SVQ3

GKDTRFNFTSHLYHALCSKGINCFIDG‑RIERGVEISHAIIRAIRGSRISIAVFSQDYASSSYCLDELLAMLSCNA‑SRDHFFFPIFYKVDPEDVEKQTGNFGKAFGEVEAEFSGN‑‑LEKVSRWKAALAKAAKFAGWPLLDN‑‑GDEAKFIQSIV

>B9S744

GQDTRENFTDHLYTGLVERGIKTYRDDKKLARGNVITATLLRAIERSRMSIIVFSKNYAASRWCLDELVKIVKCNK‑LTGHMILPVFLNVKPDHVSKQTGAYRKAFRDHEKEFKKK‑‑‑‑RVDKWRNALKKVTDVSGWDKDNY‑‑RSESKLIKIIG

>B9SNY0

GTDTRNSFVSHLYAALCRERISTFLDIG‑LKRQEEITATMHKSIEASRTSIVIFSKNYGASPWCLDELVKILECRK‑TMGQIVLPVFYEVDPREVRKQSGAFGEAFSRHVIDF‑‑‑‑‑TDKVSRWRTALAEAANYSGWVLGDT‑‑RPESLVINDIV

>B9T880

GDWSKKSVRDFMYLCLQRLTNDTWRDA‑‑LHAGKRGSSHLLSAVKKLTRYLCIFHRNYASSSWSLDELVKIVECKE‑TIGQKVLPVFYQVDPTDVQELTGSFADAFVKHRKEFKHN‑‑LDKVEKWSQALMEIANLKGWDSQVI‑‑KPESKLIEEIV

>B9REV4

GDDTGKNFSDHLYAALEHSGIHTFRGDYGVERGEIVDAEFQKAMQQSKLCLVVFSKDYASSIWCLEELVKIMEVRK‑NGGLIVMPVFYDADPNQVWEQSGSYAKAFAIHEE‑‑MEE‑‑MEKVQRWRAVLREITDLSGMDLQQR‑‑‑HEAEFIQDIV

>B9RM35

GEDTGKNFTSHLYAALCQKGVITFKDDQELERGTLSDQEIFKAIQDSSISIVIFSRNSASSTRCLDELVEIFECMK‑TKGQNVLPVFYSVDPAEVRKQTGRFGESFAKYEKLFKNN‑‑IGKVQQWRAAATGMANLSGWDTQNR‑‑‑HESELIEEIV

>B9S9E3

GLDTRNGFLSHLFKALREKQIIAFKDEN‑LDRGEQISDTLSRTIEESYVLVVILSKNYVDSPWCLDELVKILQCNK‑EKGQVVLPVFYEIDPTEVQELTGSYADALMNHRKEFEDCL‑‑‑‑VESWSHALKEIAGMAGFVSRNM‑‑KPESKLIEEIV

>B9RYD1

GEDTRYNFTSHLHAALNGKKIPTFIDDD‑LERGNEISPSLLKAIEESKISVVIISQDYPSSKWCLEELVKILECMK‑NRGQMVIPVFYRVDPSHVRNQTGSFEDVFARHEESLSVS‑‑KEKVQSWRAALKEVANLSGWHSTST‑‑RPEAEAVKEII

>B9SBW5

GEDTRNDFTSHLYAALQRKQVRTFIDNE‑LVRGVEIAPTLLKVIEEVAISVVIFSENYGNSPWCLDELVKIIECKK‑TMKQMVLPVFYRVDPAHVAELKGSFGVAFAMHEVRFSR‑‑‑‑DKLKRWRSALSEAANLSGWDSLVI‑‑RPESKLIGDIV

>B9RYC9

GEDTRYNFTSHLHAALNGKRIPTFIDDD‑LERGKEISPSLLKAIEESKISVVIISQDYPSSKWCLEELVKILECMK‑NRGQMVIPVFYRVDPSHVRNQTGSFEDVFAQHKESLLVS‑‑KEKVQSWRAALKEVANLSGWHSTST‑‑SHQGKS‑‑‑‑‑

>B9S039

GGDTRKNFTDHLYTALIQAGIHTFRDDDEIKRGENIESEIKNAIRESKISVLVLSKDYASSRWCLDELAMIMERRR‑TDGHIVVPVFYDADPTEVGKQIGSYGEAFERHEKVFKEE‑‑MEMVEGWRAALREVADMGGMVLEN‑‑‑RHQSQFIQNIV

>B9SBV5

GADTRHNLISHLYAALSRKHVTTFIDDHGLDRGEEISPTLLKAIEESKISVIIFSENYASSKWCLDELVKIMECMK‑TMSRNVLPVFYHVDPSDVRKQTGSFGQAFGVVKEKFKGS‑‑MDRVQRWSTALTEAANLSGWDSNNY‑‑RLESELIEGVI

>B9RVW4

GEDTRKKFTSHLYKELCQKGIITFKDDRELPKGEPFPTELPKAIQDSRILVVVFSENYATSTWCLDELVKILECKK‑‑AGQTVLPIFYDVIPDEVREQDGKFGEPFIEYEILYKDN‑‑IEKVQQWRVASTEIANLSGWHLHDR‑‑‑EEADFIQDIV

>B9RBV2

GEDTRDNFTSHLFAALSRKSVITFMDNNDLHVGEEITPAISKAIEESKIAIVIFSERYAFSRWCLNEIVRIIECKE‑TCGQLVLPVFYHVGPSDVS‑‑‑‑VFAEAFPSYD‑‑‑‑‑‑‑QFEKVQKWKNALSKAANLSAFDSRVT‑‑RPESKLVDEIV

>B9SHJ1

GKDIRDGFLSHLHEALRQSQINAFIDES‑IERGKEISSSLLKIIEESHVSVVIFTENYADSPW‑‑‑‑‑‑‑‑‑‑‑‑‑‑‑‑‑‑‑‑‑‑‑‑‑‑‑‑‑‑‑‑‑‑‑‑‑‑‑‑‑‑‑‑‑‑‑‑‑‑‑‑‑‑‑‑‑‑‑‑‑‑‑‑‑‑‑‑‑‑‑‑‑‑‑‑‑‑‑‑‑‑‑PDSKLIGEVV

>B9SFT6

GEDTRKNFTDHLYNALLQAGIHAFRDDKHLSRGNHISSELLKAIQESKVSIVVFSKGYASSRWCLDELVKIMQCKN‑TAGQIVVPIFYDVSPSDVRKQTGSFAEALQRH‑EQFSER‑‑E‑KVNDWRNALLEAANLSGWDLQNVANGHESKNIRKVV

>B9RYC7

GEDTRINFTSHLHDALLKNNILTFIDNE‑LVRGEALSPSLLKAIEESKISVVILSENYPYSKWCLEELVKILECMK‑INGQMVIPVFYKVDPSHVRNQTGSFADAFARHEESLLVT‑‑EDKVKSWRAALKDVANISGWDSRVT‑‑SPESELIKKII

>B9S6Y9

GEDTRDNFTSHLYSALNKKKIFTFMDKE‑IKRGEEISPSIAKAIKGSKLSVIIFSEKYAFSKWCLDELTKILECKK‑MNGQIVIPVFYRVDPVHVRNQRGSFACAFAKHEETLKER‑‑MEKVESWRSALNEAGSISGWNSLVA‑‑RPESKLIEEIV

>B9RVC7

GGDTRKNFTDHLYTALVQEGIHTFRDDDEIKRGEDIELEIQRAITESKLSVIVLSKDYASSRWCLDELVLIMERRK‑LVGHVVVPVFYDVEPYQVRNQTGSYGEAFAKHEKDFKED‑‑MSRVEEWRAALKEAAELGGMVLQD‑‑‑GYESQFIQTIV

>B9RBV1

GADTRQNFTSHLHFALCRKSIRTFIDDE‑LSRGEQITPALLEVVEESRIAVIIFSKNYGSSTFCLDEVAKIIECNE‑THRQTVVPVFYHVDPLDVENQTGSFETAFAKHE‑‑‑‑‑‑INFDRVQRWKAALSKAASMAGWDSKVI‑‑RMESQLVENIV

>B9S6Z6

‑‑DEDNNFVSHLYRKLSLEGIHTVENGGKLE‑‑‑‑‑‑‑‑‑‑‑AIQESRLIVVVLSEKYACSAQCLDELVKITDCWE‑KTRKMVVPIFHNVDPDDLGNQRGKVAEAFAKHEENF‑‑‑‑‑KEKVKMWKDALTKVASICGWDSLQW‑‑‑EETIFIEQIV

>B9S2G3

GEDTRHTFTENLYRELIKHGVRTFRDDEELQRGDEIAPSLLDAIEDSAAAIAVISKRYADSRWCLEELARIIECRR‑‑‑‑LLLLPVFHQVDPSDVRKQTGPFERDFKRLEERF‑‑‑‑GVEKVGRWRNAMNKAGGISGWDSKLW‑‑‑EDEKLIESLV

>B9SBW2

GEDTRDNFTSHLYAALHQKQIKAFVDDK‑LSRGEEISAALVKVIEESMVSVIIFSENYAFSPWCLDELVKILECKK‑TVGQIVLPVFYHVDPSDVAEQKGGFGAAFIEHEKCFKER‑‑IDKLQKWRAALTEAANISGWSSSVI‑‑RSESKLIQEIA

>B9S6Z7

GKDTRDSFTNYLYKDLYQKGIETFIDNK‑LNRGEEITPELLKAIQESMVAVVVFSHNYADSPWCLDELVHIMECKR‑AHGQIVLPVFYRVDPSEVEEQIGEFGKGFDRAKKQANGD‑‑MRLVKKWKAALKDAANLSGWDSSVV‑‑RPDSKLITEIV

>B9SXA8

GEDTRKNFTSHLYAALRQKGINAFKDDRQLERGKTISQELVKAIRASKILMIIFSRNYAFSRWCLEEAVEIAECAK‑GNGQMVVPVFYNVNPNEVRKQTGDFGKAFGEHQLRFRNN‑‑LLTVQRWRLALTQLGSLSGWDLQER‑‑‑TESELIEEII

>B9SAH3

GVDTRSNFVSHLYKALTTKGILTFIDDA‑LLRGKEISPFLLQAIEDSSMGITLFTQKYASSPWCLDELVKMTQCRS‑THRQIIIPVFYGVDRSHVKELSGEFGNEFKRLETVP‑‑‑‑DTDRVEKWKAALPEAASLSGWVSGTI‑‑GSDVKLIDEIV

>B9S9D5

GEDTRNNFISHLHAALSRKSIRTFIDDE‑LRRGDEITRSLLKKIEESKIAVVIFSRNYASSTYCLDELEKIIEFHE‑CYGQTVIPIFFNVNPSDLLEDTGIFAEALSRHEKDIQEK‑‑LDKVQRWKVALKKAGNLSGHDLQII‑‑RRESELVDKIV

>B9S9E4

GLDTRNAFLSHLFKALTEKQIITFKDEN‑LDRGERISNTLLQTIRESYVSVVIFSKNYACSTWCLEELVTILQCNE‑EMGQVVLPVFYEIDPTEVQELTGSYGNALMNHRKEFEDCS‑‑‑‑VESWSHALKKVGAMAGFVSWDT‑‑KPESKLIEEIV

>B9RM36

GEDTRKNFTSHLYAALCQKGVITFRDDEELERGKTISQALLQAIHGSKIAVIVFSRDYASSSWCLDELAEIHKCRK‑EKGQIVMPVFCNVNPYEVRKQAAGFGKAFAKHELRFKND‑‑VQKVQRWRAAISELANLAGWDSLDR‑‑‑HESELIQEIV

>B9SHM4

GKDTRDNFTSHLYDALCRKKIKTFIDNG‑LERGEEITPALLRTIEESLISVIVFSENYASSPWCLDEMVKILECRE‑THGQAVLPVFYHVDPSDVEEQNGSFALTLVELEKNFK‑‑‑‑‑DKVSKWRTDLMKAASISGWDSRAI‑‑GSEAKLVKHIV

>B9SX40

GADVRDGFLSHLHQSLDRNQVNAFVDEK‑LKRGKEITSSLLEIIEKSYVSIVIFSKNYADSPWCLDELVKIFECYK‑KMKQIVV‑‑‑‑‑‑‑‑‑‑‑‑‑‑‑‑‑‑‑‑‑‑‑‑‑‑‑‑‑‑‑‑‑‑‑‑‑‑‑‑‑‑‑‑‑‑‑‑‑‑‑‑‑‑‑‑‑‑‑‑‑RPDSRLIREIV

>B9SNY2

‑‑STRFKFTSYVCDAKLHKEIEYFNEDT‑VRKGVVRFREIKKIIKDSWISIVIISEAYATKD‑CLDQLLIILEEKR‑EKGYMVLPVFYLVSPDDVETQIGNFAKLFADL‑‑‑‑RQK‑GAQMAEKWGKALAELVGIGGRVLEPN‑‑KSEAFFVTLII

>B9RIH0

IEDTCRSFVRNLYKHLEHKGLLCFKHDGKPESGKPIPLDLLKAIEGSKIAVVVISQNYASSSWCLDELVKIIECKE‑IKGQSVFPIFHDVDPLQVKDQTGSFAQVLAEYE‑‑‑KDDM‑VEKAQRWRVALTKVALIDGWNSRDW‑‑PDDHKLTEEVS

>B9HZC5

GEDTRKNFTDHLYTALVQAGIHTLRDDDEIGRGENIN‑‑‑‑‑‑‑‑‑‑‑‑‑‑‑‑‑‑‑‑‑‑‑‑‑‑‑‑‑‑‑‑‑‑‑‑‑‑‑‑NTDCIILPVFYDVDPFEVRNQTGSFAAAFVDHDKRFKKE‑‑MEQVNGWRIAL‑‑‑‑‑‑‑‑‑‑‑‑‑‑‑‑‑YEAQLVQS‑‑

>U7DX85

GKDTRNNFTSHLYYNLAQRGIDVYMDDREFERGKTIEPALWKPFEESRFSVIIFSRDYASSPWCLDELVKIVQCMK‑EMGQTVLPVFYDVDPSEVTDRKRKYEEAFGEHEQNFKEN‑‑LEKVRNWKDCLSTVANLSGWDVRNR‑‑‑NESESIKIIV

>U7E0X1

KKKNRSHWNKKKVVRALERGIDVYMDDRELERGKAIEPALWKAIEESRISVVIFSRDYASSPWCLDELVKIVQCMK‑EMGHTVLPVFYDVDPSDVAERKRKYEKAFVEHEQNFKEN‑‑MEKVRNWKDCLSTVANLSGWDVRHR‑‑‑NESESIRIIA

>U7E2T9

GKETRNNFSSHLYSNLKQRGIDVYMDDRELERGKAIEPALWKAIEESRISVVIFSRDYASSPWCLDELVKIVQCMK‑EMGHTVLPVFYDVDPSDVAERKRKYEKAFVEHEQNFKEN‑‑MEKVRNWKDCLSTVANLSGWDVRHR‑‑‑NESESIRIIA

>105922849

GKDTRNNFTSHLYSNLAQRGIDVYMDDSELERGKTIETALWKAVEESRFSVIIFSRDYASSPWCLDELVKIVQCMK‑EMGQTVLPVFYDVDPSEVAKRKGQYEKAFVEHEQNFKEN‑‑LEKVRNWKDCLSTVANLSGWDIRNR‑‑‑NESESIKIIV

**Aligned Pentapetalae sequences from Pfam used to build profile HMM after alignment pruning and exclusion.**

>B9I808

GKDTRNNFTSHLYSNLVQRGIDV‑YMDDRGLE‑RGKTIEPALWKAIEDSRFSIVVFSRDYASSPWCLDELVKIVQCM‑KEMGHT‑VLPVFYDVDPSEVADQKGNYKKAFIEH‑KEKLSEN‑‑‑LD‑‑RVKCWSDCLSTVAN‑LSGW‑‑‑DV‑R‑‑‑‑NSDESQSIKKIA

>B9INW3

GEDTRKTFTDHLYTALVQAGIRA‑FRDDDDLP‑RGEEISDHLLRAIQESKISIVVFSKGYASSRWCLNELVEILECK‑RKTGQI‑VLPIFYDIDPSDVRKQTGSFAKAFDKH‑EKRF‑‑‑‑‑‑EE‑‑KVKEWRKALEDAAN‑LSGR‑‑‑SL‑NN‑‑ANGHEAKFIKKII

>U5FYE6

GEDTRRKFTDHLYTALVQAGIHT‑FRDDDEIQ‑RGHNIELEIQKAIQQSKISIIVFSIDYARSRWCLDELVMIMERK‑RTTNSI‑VLPVFYDVDPSQVRNQTGSFAAAFVEH‑EKRFKEE‑‑‑ME‑‑RVNGWRIALKEVAD‑LGGM‑‑‑VL‑G‑‑‑‑DGYEAQFVQSIV

>B9HZC1

GEDTRKNFTDHLYTALVQAGIHT‑FRDDNEIR‑RGENIDFELQKAIQQSKISIIVFSKDYASSRWCLDELVMIMERK‑RNADCI‑VLPIFYDVDPSQVGRQTGSFSAAFVEH‑EKSFNKE‑‑‑ME‑‑RVNGWRIALKEVAD‑LAGM‑‑‑VL‑G‑‑‑‑DGCEAPFVQSIV

>U5FES9

GEDTRVGFTSHLHAALDRKQILT‑FID‑YQLV‑RGDEISASLLRTIEEAKLSVIVISENYASSKWCLEELAKIIERR‑RNNRQI‑VIPVFYKVDPSHVRNQTGSFGDAFARL‑IRNKALT‑‑‑LE‑‑EVQSFREALTDAAS‑LSGW‑‑‑NL‑G‑‑‑NSDLESEFIEKIV

>U5GDG4

GTDTRNSFTSHLYDALQRNQIDA‑YIDN‑KLD‑GGEKIEPALLERIEESFISLVIFSENYADSTFCLRELSKILECM‑ETKQQM‑VLPVFYQLDPSHVQNLTGSYGDALCKH‑ERDRSSE‑‑‑‑‑‑‑EVESWRRALKEIAN‑LKGW‑‑‑DS‑N‑‑‑‑‑‑DETKLIQEIV

>U5G9F5

GEDTRKTFTDHLHTALVQAGIHT‑FQDDDELP‑RGEEISDHLLKAIRESKISTVVFSKGYASSRWCLNELVEILKCR‑RKTGQI‑ALPIFYDIDPSDVRKQTGSFAEAFVKH‑EERS‑‑‑‑‑‑KE‑‑KVKEWRETLEEAGN‑LSGW‑‑‑NL‑KD‑‑ANGHEAKFIQEII

>U5FXY6

GEDTRKNFTDHLYKALIHAGFHT‑FRDDDEIR‑RGKNIRLELQKAIKQSKIAIIVFSKNYAWSKWCLDELVKIMERK‑RNAECI‑VFPVFYHVDPSEVRNQTGSFAAAFVEH‑EKHYKEK‑‑‑ME‑‑RVNGWRIALKEVAN‑LAGM‑‑‑DL‑G‑‑‑‑DGYEAQFVQSIV

>U7DUN2

GKDTRNNFTSHLCKDLRRQKIKT‑FIDDR‑LE‑RGEEITPALLKTIEESRVSIVIFSENYASSPWCLDELVKILECK‑ETYGQI‑VLPVFYHVDPSDVDEQTGSFGNAFSEL‑EKNFKGK‑‑‑MG‑‑KVPRWRADLTYAAS‑ISGW‑‑‑DS‑Q‑‑‑VTSPEAKLISEVV

>U5GDK8

GTDTRNSVTSHLYDALQRNQIDA‑YIDD‑KLD‑RGEKIEPALLERIEESCISLVIFSENYADSTFCLRELSKILECM‑ETKQQM‑VLPVFYRLDPSHVQNLTGSYGDALCKH‑ERDCSSE‑‑‑‑‑‑‑EVQSWRHALKEIAN‑LKGW‑‑‑DS‑N‑‑‑‑‑‑DETELIQEIV

>B9IQX2

EQDTLAGFTSHLYAALDRKQILT‑FID‑YQLV‑RGDEISASLLRTIEEAKLSVIVFSENYASSKWCLEELVRIFECR‑KNNGQI‑VIPVFYKVDPTHLRHQTGSFGDAFARL‑IRNKALT‑‑‑LE‑‑EVQSFRDALTDAAN‑LSGW‑‑‑SL‑G‑‑‑NSGPESEFIEKIV

>U5GRB6

GEDTRKNFTDHLFTALQKAGIRT‑FRDDDELR‑IGEEIS‑QLPKAIQESKISIVVFSKGYASSTWCLDELEKILDCR‑QPTGQI‑VLPVFYDIDPSDIRKQTGSFAEAFDRH‑EERFKE‑‑‑EME‑‑KVQKWRKALVEAAN‑ISGL‑‑‑DSFA‑‑‑‑NGHESKLIQKIV

>U5GDJ5

GADTRKGFTSHLYHVLQRNQIDA‑YIDN‑KLD‑GGEKIEPALLERIEESFISLVIFSENYADSTFCLRELSKILECM‑ETKQQM‑VLPVFYRLDPSHVQNLTGGHGDALCKH‑ERECSSE‑‑‑‑‑‑‑EVESWRHALKEIAS‑VKGW‑‑‑DS‑N‑‑‑‑‑‑DETKLIQEIV

>U5FIF6

GKDTRNNFTSHLYDATRRKKIKT‑FIDDG‑LE‑RGEEITPALLKTIEESRISVVIFSKNYASSPWCVDELVKILECK‑ETYGQI‑VLPVFYHVDPSDVDEQTGSFGNAFAEL‑EIFFKGK‑‑‑MD‑‑KVPRWRDDLRKAAS‑ISGW‑‑‑DS‑Q‑‑‑VTSPESTLVREVV

>U5FE91

GEDTRKTFTDHLYAALDQAGIHT‑FRDDDELP‑RGEEISEHLLKAIRESKISIVVFSKGYASSRWCLNELVEILKCK‑KKTGQI‑VLPIFYDIDPSDVRKQTGSFAEAFDKH‑EERF‑‑‑‑‑‑EE‑‑KVKEWRKALEDAGN‑LSGW‑‑‑SL‑ND‑‑ANGHEAKFIKEII

>U5GJU3

GEDTRKNFTDHLFTALQKAKVRT‑FRDDDELR‑IGEEIS‑QLPKAIQESKISIVVFSKGYASSTWCLDELEKILDCK‑HTTGQI‑VIPVFYDIDPSDIRKQTGSFAEAFDKH‑EERFKE‑‑‑EME‑‑KVHKWRKALVEAAD‑LSGL‑‑‑DSIA‑‑‑‑NGHESKLIQKIV

>U5FEH3

GEDTRKTFTDHLYTALVQAGIHT‑FRDDDELP‑RGEEISDHLLRAVQESKISIVVFSKGYASSRWCLNELVAILKCK‑KKRGQI‑ALPIFYDIDPSDVRKQNGSFAEAFVKH‑EERF‑‑‑‑‑‑EE‑‑KVKEWRKALEEAGN‑LSGW‑‑‑NL‑ND‑‑ANGHEAKFIKEII

>B9HZC8

GKDTRKNFTDHLYTALVQAGIHT‑FRDGNEIW‑RGENIDVELQKAIQQSKISIIVFSKDYASSRWCLDELVMIMERK‑RNADCI‑VLPVFYDVDPSQVGRQTGSFSAAFVEH‑EKSFNEE‑‑‑IE‑‑RVNGWTIALKEVAD‑LAGM‑‑‑VL‑G‑‑‑‑DGYEAPFVQSIV

>U5G374

GEDTRKSFTDHLYTALCHRGVIT‑FRDDQELE‑RGNEISRELLQAIQDSRFSVIVFSRNYTSSTWCLNELVKIVECM‑KQGRQT‑VIPVFYDVDPSEVRNQTGRLQQAFADH‑EEVFKDN‑‑‑IE‑‑KVQTWRIAMKLVAN‑LSGW‑‑‑DL‑Q‑‑‑‑DRHESEFIQGIV

>U5GME6

GEDTRKKFTDHLYTALIHAGIHT‑FRDNDELP‑RGEDISSIISRPIQESRIAIVVFSKGYASSTWCLGELSEILACK‑SAIGQL‑AVPIFYDIDPSDVRKQTASFAEAFKRH‑EERF‑‑K‑‑‑EN‑‑IVNKWRKVLVEAAN‑LSGW‑‑‑HL‑QE‑‑ENGHEAKFIEKMV

>U5FE61

GEDTRKNFTDHLYFALKDAGINT‑FRDDNELR‑SGEDISTELLQAIQKSRISVILFSRNYANSRWCLEGLVKIMECW‑RSWRQL‑VFPIFYDVDPSDVRKQTGSFAEAFSGH‑EER‑‑‑‑‑‑‑‑‑‑‑‑‑‑‑‑‑‑‑‑‑‑‑‑‑‑‑‑‑‑‑‑‑‑‑‑‑‑‑‑‑‑‑HEAKFIKKIV

>B9HZ91

GEDTRKNFTDHLYTALVQAGIHT‑FRDDDEIG‑RGENIESELQKAIQQSKIAIIVFSKDYASSRWCLDELVMIMERR‑RTADCR‑VLPVFYDVDPSQVRKQTGSFAAAFVEH‑EKRFKEE‑‑‑ME‑‑RVNGWRIALKEVAD‑LAGM‑‑‑VL‑G‑‑‑‑DGYEALLVQCIV

>U7E1S2

‑‑‑‑‑‑‑‑TSHLYDAMRRKKIKT‑FIDDG‑LE‑RGEEITPALLKSIEESRISVVIFSKNYASSPWCVDELVKILECK‑ETYGQI‑VLPVFYHVDPSDVDEQTGSFGNAFAEL‑EKNFKWK‑‑‑MD‑‑KVPRWRADLTYAAS‑ISGW‑‑‑DS‑Q‑‑‑VTSPEAKLIREVV

>U5FFQ3

GEDTRKTFTDHLYAALDQAGIRA‑FRDDDELP‑RGEEISDHLLRAIQESKISIVVFSKGYASSRWCLNELVEILECK‑RKTGQI‑VLPIFYDIDPSDVRKQNGSFAEAFVKH‑EERF‑‑‑‑‑‑EE‑‑KVKEWRKALEEAGN‑LSGW‑‑‑NL‑ND‑‑ANGHEAKFIKEII

>U5FG45

GKDTRNNFTSHLYDALCRKKIKT‑FIDDR‑LE‑RGGEITPALLKTIEESRISVVIFSKNYASSPWCVDELVKILECK‑ETYGQI‑VLPVFYHVNPSDVDEQTGSFGNAFAEL‑EKNFKGK‑‑‑MD‑‑KVPRWRADLTNAAS‑ISGW‑‑‑DS‑Q‑‑‑VTSPESKLVTDVV

>B9I2I8

GEDTRDSFTKHLYDSLNKQEIRV‑FLDASGMI‑QGDEIAPTLMEAIQDSASSIIILSPRYANSHWCLEELARICELR‑‑‑‑‑RL‑ILPVFYQVDPSNVRRQKGPF‑QDFESH‑SKRFG‑‑‑‑‑DD‑‑KVVKWRAAMNKVGG‑ISGF‑‑‑‑VFDT‑‑‑‑SGEDHLIRRLV

>Q2XPG2

GEDTRKTFTDHLYTALVQAGIHT‑FRDDDELP‑RGEEISDHLLRAIQESKISIVVFSKGYASSRWCLNELVEILKCK‑RKTGQI‑VLPIFYDIDPSDVRKQTGSFAEPFDKH‑EERF‑‑‑‑‑‑EE‑‑KVKEWRKALEEAGK‑LSGW‑‑‑NL‑ND‑‑ANGHEAKFIKEII

>U5G975

HQEIGKNFADHLYKDLNYAGIRT‑FRDDGGIY‑TGQ‑‑KSDVKRAIQESRISVVVFSKDYASSTKCLDQLGLIMDAR‑RTTGLV‑VLPVFYNADPSEVWEQKGLFEEAFAKH‑EKSFHKE‑‑‑MA‑‑RVESWRAALKEAAD‑LKGK‑‑‑ER‑K‑‑‑QDRYESKFIESIV

>U5FF36

GEDTRFDFTSHLHAALKRKQILT‑FID‑DQQV‑RGDEIPESLLRTIEEAKLSVPVFSENYASSKWCLEELVKIFERR‑KNNGQI‑VIPVFYKVNPSHVR‑‑‑‑SFRDAFAGL‑IKNKTLT‑‑‑EY‑‑KEKSFRDALTDTAN‑LSGW‑‑‑TL‑G‑‑‑KSEPESEFIDKIV

>B9HZD5

GEDTRKNFTDHLYKALVDAGFHT‑FRDDDEIR‑RGKNIELELQKAIQQSKIAIIVFSKNYAWSRWCLDELVMIMERK‑RNADCI‑VFPVFYHVDPSEVRNQTGSFAAAFVEH‑EKHYKEE‑‑‑ME‑‑RVNGWRIALKEVAN‑LAGM‑‑‑DL‑G‑‑‑‑DGYEAPFVQSIV

>B9GWM3

GEDTRKNFTDHLYTALHHARIHA‑FRDDDELR‑RGEEIS‑QLLKAIQESKISIVVFSKGYASSTWCLAELEKILDCR‑HTTGQI‑VLPVFYDIDPSDIRKQTGSFAEAFDRH‑EERFKE‑‑‑EME‑‑KVQKWRKALMEAAN‑LSGL‑‑‑DSFA‑‑‑‑NGHESKLIQKIV

>U5FFC1

GEETRKTFTGHLYAALDEAGIHT‑FLDDVELP‑RGEEISEHLLKAIRESKISIVVFSKGYASSRWCLNELVEILKCK‑KKTGQI‑VLPIFYDIDPSDVRKQTGSFAEAFDKH‑EERF‑‑‑‑‑‑EE‑‑EVKEWRKALEDAGN‑LSGW‑‑‑SL‑ND‑‑AYGHEAKFIRGII

>U5FHF5

GEDTRKTFVDHLYTALVQAGIHT‑FRDDDELP‑RGEEISEHLLEAIRESKISIVVFSKGYASSRWCLNELVEILKCK‑KKTGQI‑VLPIFYDIDPSDVRKQTGSFAEAFDKH‑EERF‑‑‑‑‑‑EE‑‑KVKEWRKALEDAGN‑LSGW‑‑‑SL‑ND‑‑ANGHEAKFIKGII

>B9INY7

GADTRKTFLGHLYNALVQAGIHT‑FKDDEELP‑PGEEISQQLKKAIQESKISIVVFSRDYASSRWCLNELVEILECR‑NTKGR‑‑VFPIFCGVDPSHVR‑QEGSFKKAFKAY‑‑‑‑‑‑‑EKEEKE‑‑KINKWKNALKDAAN‑LSGK‑‑‑DIYS‑‑‑ANGDESVLIKKIV

>U7DXH7

GEDTRRNFTDHLYKALSREGIPT‑FRDDDGIR‑RGENIELEIKKAIQETKLSIIVFSKEYASSRWCLDELEMIMERR‑RTVGHI‑VFPVFYDVDPSEVGTQTGRYGEEFAKH‑EIHFKD‑‑‑‑‑‑‑‑RVEGWRKALKEVAY‑MEGM‑‑‑VL‑‑‑‑‑EDGYESKFIESIV

>U5FFT0

GQETRNTFTAHLYHALCNKGINA‑FIDDK‑LE‑RGEHITSQLNQIIEDSRISLVIFSENYARSIYCLDELVKILECK‑ESKGQV‑VLPVFYNVDPSDVEEQKGSFGESLDFH‑ETYLGIN‑‑‑AE‑‑QLKQWREALTKAAQ‑LSGW‑‑‑H‑‑‑‑‑‑LDRNEAVFIRKIV

>B9HZ97

GEDTRKNFTDHLYTALVQAGIHT‑FRDDDEIG‑RGENIESELQKAIQQSKIAIIVFSKDYASSRWCLDEIVMIMERR‑RTADCR‑VLPVFYDVDPSQVRKQTGSFAAAFVEH‑EKHFKEE‑‑‑ME‑‑RVNGWRIALKEVAD‑LAGM‑‑‑VL‑G‑‑‑‑DGYEALLVQCIV

>U5FIR8

GEDTRFDFTSHLYAALNRKQILT‑FID‑YQLV‑RGDEISASLLRTIEEAKLSVIVFSENYASSKWCLEELAKIFERR‑KNNGQI‑VIPVFYQVDPSHVRNQTGSFGDAFARL‑IKKKALT‑‑‑MD‑‑KEQSFRDALKDTST‑LSGW‑‑‑TV‑G‑‑‑NSELESEFIEKIV

>U5FEN2

GKDTRDNFVSHLRDALCRKQIKT‑FIDDK‑LE‑RGEEITGALLRTIEESRISVIIFSRNYASSPWCVDELVKILECK‑KAYGQI‑VLPVFYHVDPSDVDQQTGSFGNAFAEL‑ERNFKQK‑‑‑MD‑‑KVPRWRADLTSAAN‑ISGW‑‑‑DS‑Q‑‑‑VTRPESSLVEQIV

>U7E235

GQDTRKNLTDHLYTALHHARIHA‑FRDDEKLR‑RGEEIS‑QLSKAIQESKISIVVFSKGYASSTWCLGELQKILECR‑QPTGQI‑VLPVFYDIDPSDIRKQTGSFAEAFDRH‑EARFKE‑‑‑EME‑‑KVQKWRKALVEAAN‑LS‑‑‑‑‑‑‑‑‑‑‑‑‑‑GHESKLIQKIV

>U7E0N2

GEDARKNFTDHLYFALKDAGINT‑FRDDNGLR‑RGEDISTELLQAIQKSRISVIVFSRNYANSRWCLEELVKIMECR‑RSFRQL‑VFPIFYDVDPSDVRKQTGSFAEAFAGH‑EERFVLQ‑‑‑TD‑‑KVATWRMALTEAAN‑LSGW‑‑‑DL‑RN‑‑ADGHEAKFIKKIV

>U5FIU6

GEDTRGGFTSHLYAALDRKQIRA‑FID‑YQLR‑RGDEISASLLRTIEEAKLSVIVFSENYASSKWCLEELAKIIERR‑RNNGQI‑VIPVFYKVDPSHVRNQTRSFGDALARL‑IKKKALT‑‑‑MD‑‑KEQSFRDALTAAAN‑LSGW‑‑‑SL‑G‑‑‑NSELEFEFIKNIV

>U5FJU0

GEDTRVGFTSHLHAALERKNILT‑FID‑‑DLR‑RGEEISPSLVKAIEDSMLSVIIFSQNYASSKWCLDELLKILESR‑KVRGQI‑AIPVFYEVDPSDIRKQSGSFGDVFAQL‑‑KR‑‑‑K‑‑KME‑‑EEQCFRAALNEAAN‑ISGH‑‑‑DS‑R‑‑‑‑‑KSESKFIEVIV

>U5G3U7

GEDTRKTFTDHLYTALVQAGIQT‑FRDDDELP‑RGKEISQHLLEAIQESKISIVVFSKGYA‑SRWCLDELVEIIKCK‑RKTGHI‑ALPIFYDIDPSDVRKQTGSFAEAFVKH‑EERS‑‑‑‑‑‑KE‑‑KVKEWREALEEAGN‑LSGW‑‑‑NL‑KD‑‑ANGHEAKFIQEII

>U7DZF6

GKDTRNNFTSHLYSNLKQRGIDV‑YMDDRELE‑RGKTIEPALWKAIEESRFSVIIFSRDYASSPWCLDELVKIVQCM‑KEKGQT‑VLPVFYDVDPSEVAEQKGKYKKAFVKH‑EKDFKEN‑‑‑LD‑‑RVRSWKDCLSTVAN‑LSGW‑‑‑DI‑R‑‑‑‑NRNESEHIEKIV

>U5FJZ2

GKDTRNNFTSHLCSNLAQRGIDV‑YVDDRELE‑RGKTIEPALWKAIEESRFSVIIFSRDYASSPWCLDELVKIVQCM‑KETGHT‑VLPVFYDVDPSEVAEQKGQYEKAFGEH‑EQNFKEN‑‑‑LE‑‑KVRNWKDCLSTVAN‑LSGW‑‑‑DV‑R‑‑‑‑DRNESESIKIIA

>U5FHF8

GEDTRFDFTSHLYAALNRKQILT‑FID‑YQLV‑RGDEISASLLRTIEEAKLSVIVFSENYASSKWCLEELAKIFERR‑KNNGQI‑VIPVFYQVDPSHVRNQTGSFGDAFARL‑IKKKALT‑‑‑MD‑‑KEQSFRDALKDTAT‑LSGW‑‑‑TL‑G‑‑‑NSQLESEFIEKIV

>U5FEA9

GEDNRKNFTDHLYTALVQAGIYT‑FRDHNEIP‑RGEEISKHLLKAIQESKISIVVFSKGYASSRWCLNELVEILECK‑RKTGQI‑VLPVFYDIDPSDVRKQTGSFVKAFDKH‑EDCF‑‑‑‑‑‑KE‑‑KVKEWRKALEETGN‑LSGW‑‑‑NL‑SD‑‑ENGHESKFIQDII

>U5GNQ5

GADTRKNFTGHLYMALQGAGIRT‑FRDEDEIE‑GGEHIGFKITKAIQESKMSLVVFSRDYASSKWCLEELLMIMKRR‑ETIGHI‑VLPVFYEVDPDDVSMQTGFFAEAFASH‑EKNFM‑DNRDME‑‑‑‑‑‑WREALRKVAD‑LKGP‑‑‑‑VLR‑‑‑‑DR‑EAQFIQDIV

>B9HGH2

GKDTRKTFTDHLYTALVQAGIHT‑FRDDDELP‑RGEEISQHLLEAIQESKICIVVFSKGYASSRWCLDELVEILKCK‑RKTGQI‑ALPIFYDIDPSDVRKQTGSFAEAFVKH‑EERS‑‑‑‑‑‑EE‑‑KVKEWREALEEAGN‑LSGW‑‑‑NL‑KD‑‑TNGHEAKFIQHII

>U5FG49

GEDTRKTFTDHLYTALVQAGIHT‑FRDDDELP‑RGEEISDHLLRAVQESKISIVVFSKGYASSRWCLNELVEILKCK‑RKTGQI‑VLPIFYDIDPSDVRKQNGSFAEAFVKH‑EERS‑‑‑‑‑‑EE‑‑KVKEWRKALEEAGN‑LSGR‑‑‑NL‑ND‑‑ANGHEAKFIKEII

>B9HF56

GEDTRKNFTDHLHTALVQAGFHT‑FRDDDEIQ‑RGENIEIEIQKAIKESRMSIIVFSKDYASSRWCLDELVMIMELK‑KLGGHV‑VLPIFYDLDPSHVSNQTGSFAEAFVRH‑EERFKKE‑‑‑‑D‑‑RVEGWRMALKEVAD‑LGGM‑‑‑VL‑Q‑‑‑‑DGYESKFIQNVV

>U5FRN8

GKDTRNNFTSHLYSNLVQRGIDV‑YMDDRGLE‑RGKTIEPALWKAIEDSRFSIVVFSRDYASSPWCLDELVKIVQCM‑KEMGHT‑VLPVFYDVDPSEVADQKGNYKKAFIEH‑KEKLSEN‑‑‑LD‑‑RVKCWSDCLSTVAN‑LSGW‑‑‑DV‑R‑‑‑‑NSDESQSIKKIA

>B9N1N5

GEDTRVCFVSHLYAALKRKQIST‑FID‑YKLN‑RGEEISPSLLKAIEDSKLSVVVFSDNYASSKWCLEELAKILECK‑KVKGQM‑VIPVFYRVDPSHVRNQTGSFADAFARH‑DQLLKEK‑‑‑ME‑‑KVLNWRAAMREAAN‑LSGW‑‑‑DS‑H‑‑‑NIKSESEFVDDIV

>U5FF28

GEDTRFDFTSHLYAALNRKQILT‑FID‑YQLV‑RGDEISASLLRTIEEAKLSVIVFSENYASSKWCLEELAKIFERR‑KNNGQI‑VIPVFYQVDPSHVRNQTGSFGDAFARL‑IKKKALT‑‑‑MD‑‑KEQSFRDALKDTAT‑LSGW‑‑‑TL‑G‑‑‑NSHPESQFIEKIV

>B9IQ82

GQDTRNNFTSHLYDALCRKKIKT‑FIDNG‑LE‑RGEEITPALLKTIEESRISVVIFSKNYASSPWCVDELVKILECK‑ETYGQI‑VLPVFYHVDPSEVDEQTGSFENAFAEL‑EKNFKGK‑‑‑MD‑‑KLPRWRAGLTYAAS‑ISGW‑‑‑DS‑Q‑‑‑VTSPESKLVREVV

>Q2XPG3

GEDTRKTFTDHLYTALVQAGIHT‑FRDDDELP‑RGEEISDHLLRAIQESKMSIVVFSKGYASSRWCLKELVEILKCK‑GKTGQI‑ALPIFYDIDPSDVRKQTGSFAEAFVKH‑EERF‑‑‑‑‑‑EE‑‑KVKEWRKALEEAGN‑LSGW‑‑‑NL‑ND‑‑ANGHEAKFIKEII

>U5GAC7

GTDTRNSFTSHLYDALQRNQIDA‑YIDN‑KLD‑GGEKIEPALLERIEESFISLVIFSENYADSTFCLRELSKILECM‑ETKQQM‑VLPVFYRLDPSHVQNLTGSYGDALCKH‑ERDCSSE‑‑‑‑‑‑‑EVESWRRASKEIAN‑LKGW‑‑‑DS‑N‑‑‑‑‑‑DETKLIQEIV

>U5FE89

GKDTRKTFTDHLYTALVQAGIHT‑FRDDDELP‑RGEEIHDHLLRAIQESKISIVVFSKGYASSRWCLNELVEILKCK‑RKTGQI‑VQPIFYNIDPSDVRKQNGSFAKAFVKH‑EERF‑‑‑‑‑‑EE‑‑KVKEWRKALEEAGN‑LSGW‑‑‑NL‑ND‑‑ANGHEAKFIKEII

>U5GA93

GTDTRNSFTSHLYDALQRNQIDA‑YIDN‑KLD‑GGEKIEPALLERIEESFISLVIFSQNYADSTFCLRELSKILECM‑ETKQQM‑VLPVFYRLDPSHVQNLTGSYGDALCKH‑ERDCSSE‑‑‑‑‑‑‑EVESWRHALKEIAN‑LKGW‑‑‑DS‑D‑‑‑‑‑‑DETKLIQEIV

>U5FZ55

GEDTRNNFTDHLYKALVQAGIHT‑FRDDDEIR‑IGENIELELQKAIQQSKISIIVFSKNYAWSRWCLDELVKIMERK‑RNAACI‑VYPVFYHVDPSEVRNQTGSFAVAFVEQ‑DKRFKEE‑‑‑MD‑‑RVNGWRIALKEVAD‑LAGM‑‑‑DL‑G‑‑‑‑DGYEAQFVQSIV

>B9H393

GADTRNSFTSHLYKALCQNQIHA‑YIDY‑KLH‑GGEKIEPALLERIEESYISVVIFSENYADSTFCLRELSKILECM‑ETKGQK‑VLPVFHQLDPSHVQDLTGSYGDAICKH‑ESDCSSQ‑‑‑‑‑‑‑EVESWRHASKEIAN‑LKGW‑‑‑DS‑K‑‑‑‑‑‑DETKLIEEIV

>U5FEH7

GEDTRKTFTDHLYAALVQAKIHT‑FRDDDELP‑RGEEISDHVLRAIQESKISIVVFSKGYASSRWCLDELVEILKCK‑KKTGQI‑VLPIFYDIDPLDVRKQTGRFAEAFVKH‑EERF‑‑‑‑‑‑EE‑‑KVKEWRKALKEAGN‑LSGW‑‑‑NL‑ND‑‑ANGPEANFVKEII

>B9HZ86

GEDTRKNFTDHLYTTLVQAGIHT‑FRDDNEIR‑RGENIDFELQKAIQQSKISIIVFSKNYAWSRWCLDELVMIMERR‑RTTGSI‑VFPVFYDVLPSEVRNQTGSFAAAFVEQ‑EKRFKEE‑‑‑ME‑‑RVNGWRIALKEVAD‑LAGM‑‑‑VL‑G‑‑‑‑DGYEAQFVQSIV

>U5GA98

GTDTRYSFTSHLYDALQRKQIDA‑YIDD‑KLD‑GGEKIEPAILERIEESFISAVIFSENYADSTFCLRELSKILECM‑ETKQQM‑VLPVFYRLDPCQVQNLTGSYGDALCKH‑EKDCGSK‑‑‑‑‑‑‑EVESWRHASKEIAN‑LKGW‑‑‑NS‑N‑‑‑‑‑‑DEIKLIEEIV

>U5FF18

GEDTRKNFTDHLYFAFKDAGINT‑FRDDNELR‑RGEDISTELLQAIQKSRISVIVFSENYANSRWCLEELVKIMECR‑RSCRQL‑VFPIFYDVDPSDVRKQTGSFAKAFAGH‑EERFVLQ‑‑‑TD‑‑KVATWRMALTEAAN‑LSGW‑‑‑DL‑RN‑‑ADGHEAKFIKKIV

>U5FVE5

GEDTRKNFTDHLYKALVDAGIHT‑FRDDDEIQ‑RGENIDFELQKAIQQSKISIIVFFKDYASSRWCLDELVMIMERK‑RNDDCI‑VLPVFYDVDPSQVGRQTGSFAAAFVEH‑EKSFNEE‑‑‑KE‑‑RVSGWRIALKEVAD‑LAGM‑‑‑VL‑G‑‑‑‑DGYEAQFVQSIV

>B9IQ77

GEDTRNNFTSHLYDALCRKKIKT‑FIDDG‑LE‑RGEEITPALLKKIEESRISVVIFSKNYASSPWCVDELVKILECK‑ETCGQI‑VLPVFYHVDPSDVDEQTGSFGNAFSEL‑ENIFKGK‑‑‑MD‑‑KVPRWRADMTYAAS‑ISGW‑‑‑DS‑Q‑‑‑VTSPESKLVREVV

>B9IQ03

GKDTRKTFTNHLYTALVQAGIHT‑YRDDDELP‑RGEEISDHLLRAIQKSKISIPVFSKGYASSRWCLNELLEILKCK‑RKTGQI‑VLPIFYDIDPSDVRKQNDSFAEAFVKH‑EKRF‑‑‑‑‑‑EE‑‑KVKEWRKALEEAGN‑LSGW‑‑‑NL‑NA‑‑ANGYEAKFIKKII

>B9HZD8

GEDTRKNFTDHLYKALVDAGFHT‑FRDDDEIR‑RGKNIQLELQKAIQQSKIAIIVFSKNYAWSRWCLDELVKIMERN‑RNADCI‑VFPVFYHVDPSEVRNQNGSFAAAFVEH‑EKHYKEE‑‑‑ME‑‑RVNGWRIALKEVAN‑LAGM‑‑‑DL‑G‑‑‑‑DGYEAQFVQSIV

>U7E249

GKDTRNNFTSHLYYNLAQRGIDV‑YMDDRGLE‑RGKTIELALWKAIEESRFSVIIFSRDYASSPWCLDELVKIVQCM‑KEKGHT‑VLPVFYNVDPSET‑‑‑‑‑‑YEKAFVEH‑EQNFKEN‑‑‑LE‑‑KVRNWKDCLSTVAN‑LSGW‑‑‑DV‑R‑‑‑‑NRNESESIKIIA

>B9INW6

GEDTRKTFTDHLYSALVQAGIHA‑FRDDDDLP‑RGEEISDHLLRAIQESKISIVVFSKGYASSRWCLNELVEILECK‑RKTGQI‑VLPIFYHIDPSDVRKQNGSFAEAFANN‑EERF‑‑‑‑‑‑EE‑‑KVKEWRKALEEAGN‑LSGW‑‑‑NL‑NH‑‑ANGHEAKFIKEII

>U5GJU8

GEDTRKNFTDHLYTALLQAGIHT‑FRDDEKLR‑RGEEIS‑QLSKAIQESKISIVVFSEGYASSTWCLGELQKILDCR‑HTTGQI‑VLPVFYDIDPSDIRKQTGSLAEAFGKH‑EESFKE‑‑‑EME‑‑KVQKWRKALLEAAN‑LSGL‑‑‑DSIA‑‑‑‑NGHESKLIQKIV

>U5FFB8

GEDTRKTFTDHLYTALVQAGIHT‑FRDDDELP‑RGEEISDHLLRAIQESKISIVVFSKGYASSRWCLNELVEILKCK‑RKTGQI‑VLPIFYDIDPSYVRKQDGSFAEAFVKH‑EERF‑‑‑‑‑‑EE‑‑KVKEWRKALEEAGN‑LSGW‑‑‑NL‑ND‑‑ANGHEAKFIEGII

>U7E062

GPDTRKNFTDHLYKALIQAGIHT‑FRDVDEIR‑RGENIDFELQKAIQQSKISIIVFSKDYASSRWCLDELVMIMERK‑RNDDCI‑VLPVFYDVDPSQVGRQTGSFAAAFMEH‑EKHFNEE‑‑‑KE‑‑RVSGWRIALKEVAD‑LAGM‑‑‑VL‑G‑‑‑‑DGYEAQFVQSIV

>U5FFQ2

GEDTRKTFTDHLYTALVQAGIHT‑FRDDDELS‑RGEEISKHLLRAIQESKISIVVFSKGYASSRWCLNELVEILKCK‑RKTDQI‑VLPIFYDIDPSDVRKQNGSFAEAFVKH‑EERF‑‑‑‑‑‑EE‑‑KVKEWRKALEESGN‑LSGW‑‑‑NH‑ND‑‑ANGHEAKFIKEVV

>B9IE71

GEDTRHGFTKNLYDSLSKQDIRV‑FLDDSGMT‑QGDEIAPTLMEAIEDSALSIIILSPRYANSHWCLEELARICELR‑‑‑‑‑RL‑ILPVFYQVDPSHVRRQKGPL‑QDFMNH‑MERFG‑‑‑‑‑EE‑‑KVGKWREAMYKVGG‑ISGF‑‑‑‑VFDT‑‑‑‑RSEDQLIRRLG

>U5GA00

GTDTRYSFTSHLYDALQRKQIDA‑YIDD‑KLD‑GGEKIEPAILEGIEESFISVVIFSENYADSTFCLRELSKILECM‑ETKQQM‑VLPVFYRLDPCQVQNLTGSYGAALCKH‑EKDFGSK‑‑‑‑‑‑‑EVESWRHALKEIAN‑LKGW‑‑‑NS‑K‑‑‑‑‑‑DEIKLIEEIV

>U5FGD1

GEDTRVGFTSHLYAALKREQILT‑FID‑NQLV‑RGDEISASLLRTIEEAKLSVIVFSANYASSKWCLEELAKIFERR‑KNNGQI‑VIPVFYQVDPSHVRNQAGSFGDALARL‑IKEKALT‑‑‑MD‑‑TEQSFGDALTDAAN‑LSGW‑‑‑RL‑G‑‑‑NSELEAEFIEKIV

>U5FN17

GKDTRNNFTSHLCSNLAQRGIDV‑YVDDRELE‑RGKTIEPALWKAIEESRFSVIIFSRDYASSPWCLDELVKIVQCM‑KETGHT‑VLPVFYDVDPSEVAEQKGQYEKAFGEH‑EQNFKEN‑‑‑LE‑‑KVRNWKDCLSTVAN‑LSGW‑‑‑DV‑R‑‑‑‑DRNESESIKIIA

>U5GKK7

GEDTRKNFTDHLYSTLSKAGIVT‑FRDDNSIQ‑RGENIE‑EIEKAIQESQMSVVVLSKDYASSTWCLDELVMIMDRK‑RTAGHI‑VLPVFYDVDPSQVGEQTGNYAEAFAKH‑QDHFQD‑‑‑DME‑‑RVEKWKATLKEVAY‑LGGM‑‑‑‑VLQ‑‑‑‑DRHESQFIGDIV

>U5GDH5

GTDTRNSVTSHLYDALKRNHIDA‑YIDN‑KLD‑GGEKIEPALLERIEESCISLVIFSEKYADSTFCLRELSKILECK‑ETKGQM‑VLPVFYRLDPSHVQNLTGSYGDALCRH‑ERDCCSQ‑‑‑‑‑‑‑EVESWRHASKEIAN‑LKGW‑‑‑DS‑N‑‑‑‑‑‑DETKLIQEIV

>U5GDL2

GADTRKGFTSHLYDALKRSQIDA‑YIDN‑KLD‑GGEKIEPALLKRIEESFISLVIFSENYADSTFCLRELSKILECM‑ETKQQM‑VLPVFYRLDPSHVQNLTGSYGDALCKH‑ERDCSSE‑‑‑‑‑‑‑EVESWGHALKEIAS‑VKGW‑‑‑DS‑N‑‑‑‑‑‑DETKLIQEIV

>U5FHD1

GEDTRLGFTSHLHAALDRKQILT‑FID‑DQLE‑RGDEISASLLRTIEEAKLSVIVFSANYASSKWCLEELVKIFECR‑KNNGQI‑VFPVFYKVDPTHVRNLTGSFGGAFARL‑IRNKALT‑‑‑LE‑‑EVQSFRDALTDAAS‑LSGW‑‑‑NL‑G‑‑‑NSELEAEFIEKIV

>B9IP51

GADTRKTFLGHLYNALVQAGIHT‑FKDDEELP‑PGEEISHHLKKAIQESKISIVVFSRDYASSRWCLNELVEILECR‑NTKGR‑‑VFPIFCGVDPSHVR‑QEGSFKKAFKAY‑‑‑‑‑‑‑EKEEKE‑‑KIDKWKNALKDAAN‑LSGK‑‑‑DIYS‑‑‑ANGDESVLIKKIV

>U7E0N6

GEDTRKNFTDHLYFALKDAGINT‑FRDDNELR‑SGEDISTELLQAIQKSRISVILFSRNYANSRWCLEGLVKIMECW‑RSCRQL‑VFPIFYDVDPSDVRKQTGSFAEAFSGH‑EERFVLQ‑‑‑TD‑‑KVATWRMALTEAAN‑LSGW‑‑‑DL‑RN‑‑ADGHEAKFIKKIV

>U5FG53

GEDTRQTFTDHLYTALVQAGIHT‑FRDDDELP‑RGEEISDHLLRAIHESKISLVVFSKGYASSRWCLNELVEILQCK‑RKTNQI‑VLPIFYDIDPSDVRKQNGSFAEAFVKH‑EERF‑‑‑‑‑‑EE‑‑KVKEWRKALEEAGN‑LSGW‑‑‑NL‑ND‑‑ANGHEAKFTKEII

>U5GA82

GFDTRNSFTSHLYDALQRNQIDA‑YIDN‑KLD‑GGEKIEPALLKRIEESFISLVIFSENYADSTFCLRELSKILECM‑ETKQQM‑VLPVFYRLDPSHVQNLTGSYGDALCKH‑EKDCSSE‑‑‑‑‑‑‑EVKSWRRALKEIAN‑LKGW‑‑‑DS‑D‑‑‑‑‑‑DETKLIQEIV

>B9S9D9

GLDTRNGFVSHLFKALSEKQIIT‑FK‑DENLD‑RGEQISDTLSQTIKESYVSVVIFSKNYACSAWCLDELVTILQCN‑KEMGQV‑VLPVFYEIDPTEVQELTGSYGNALMNH‑RKEFENC‑‑‑L‑‑‑‑VESWSHALMEIAA‑MAGF‑‑‑VSTK‑‑‑‑‑‑PESKLIDEIA

>B9SVQ3

GKDTRFNFTSHLYHALCSKGINC‑FIDGR‑IE‑RGVEISHAIIRAIRGSRISIAVFSQDYASSSYCLDELLAMLSCN‑ASRDHF‑FFPIFYKVDPEDVEKQTGNFGKAFGEV‑EAEFSGN‑‑‑LE‑‑KVSRWKAALAKAAK‑FAGW‑‑‑PL‑‑‑‑‑LDNDEAKFIQSIV

>B9S744

GQDTRENFTDHLYTGLVERGIKT‑YRDDKKLA‑RGNVI‑TTLLRAIERSRMSIIVFSKNYAASRWCLDELVKIVKCN‑KL‑THM‑ILPVFLNVKPDHVSKQTGAYRKAFRDH‑E‑KFKK‑‑‑‑‑K‑‑RVDKWRNALKKVTD‑VSGW‑‑‑D‑KD‑‑‑‑NRSESKLIKIIG

>B9SNY0

GTDTRNSFVSHLYAALCRERIST‑FL‑DIGLK‑RQEEITATMHKSIEASRTSIVIFSKNYGASPWCLDELVKILECR‑KTMGQI‑VLPVFYEVDPREVRKQSGAFGEAFSRH‑VIDFTD‑‑‑‑‑‑‑‑KVSRWRTALAEAAN‑YSGW‑‑‑VLTR‑‑‑‑‑‑PESLVINDIV

>B9T880

GEGVCSGYFGHYNQSWSKKSVRD‑FMDRDALH‑AGKRGSSHLLSPSMQQLAAVKKLTRNYASSSWSLDELVKIVECK‑ETIGQK‑VLPVFYQVDPTDVQELTGSFADAFVKH‑RKEFKHN‑‑‑LD‑‑KVEKWSQALMEIAN‑LKGW‑‑‑DSIK‑‑‑‑‑‑PESKLIEEIV

>B9REV4

GDDTGKNFSDHLYAALEHSGIHT‑FRGDYGVE‑RGEIVDAEFQKAMQQSKLCLVVFSKDYASSIWCLEELVKIMEVR‑KNGGLI‑VMPVFYDADPNQVWEQSGSYAKAFAIH‑E‑‑‑‑‑‑‑‑EME‑‑KVQRWRAVLREITD‑LSGM‑‑‑‑DLQ‑‑‑‑QR‑EAEFIQDIV

>B9RM35

GEDTGKNFTSHLYAALCQKGVIT‑FKDDQELESRGTLSDQEIFKAIQDSSISIVIFSRNSASSTRCLDELVEIFECM‑KTKGQN‑VLPVFYSVDPAEVRKQTGRFGESFAKY‑EKLFKNN‑‑‑IG‑‑KVQQWRAAATGMAN‑LSGW‑‑‑DT‑‑‑‑‑QNRHESELIEEIV

>B9S9E3

GLDTRNGFLSHLFKALREKQIIA‑FK‑DENLD‑RGEQISDTLSRTIEESYVLVVILSKNYVDSPWCLDELVKILQCN‑KEKGQV‑VLPVFYEIDPTEVQELTGSYADALMNH‑RKEFEDC‑‑‑L‑‑‑‑VESWSHALKEIAG‑MAGF‑‑‑VSMK‑‑‑‑‑‑PESKLIEEIV

>B9RYD1

GEDTRYNFTSHLHAALNGKKIPT‑FIDDD‑LE‑RGNEISPSLLKAIEESKISVVIISQDYPSSKWCLEELVKILECM‑KNRGQM‑VIPVFYRVDPSHVRNQTGSFEDVFARH‑EESLSVS‑‑‑KE‑‑KVQSWRAALKEVAN‑LSGW‑‑‑HS‑‑‑‑‑STRPEAEAVKEII

>B9SBW5

GEDTRNDFTSHLYAALQRKQVRT‑FIDNE‑LV‑RGVEIAPTLLKVIEEVAISVVIFSENYGNSPWCLDELVKIIECK‑KTMKQM‑VLPVFYRVDPAHVAELKGSFGVAFAMH‑EVRFSR‑‑‑‑‑D‑‑KLKRWRSALSEAAN‑LSGW‑‑‑DS‑L‑‑‑VIRPESKLIGDIV

>B9RYC9

GEDTRYNFTSHLHAALNGKRIPT‑FIDDD‑LE‑RGKEISPSLLKAIEESKISVVIISQDYPSSKWCLEELVKILECM‑KNRGQM‑VIPVFYRVDPSHVRNQTGSFEDVFAQH‑KESLLVS‑‑‑KE‑‑KVQSWRAALKEVAN‑LSGW‑‑‑HS‑‑‑‑‑STSHQGKS‑‑‑‑‑

>B9S039

GGDTRKNFTDHLYTALIQAGIHT‑FRDDDEIK‑RGENIESEIKNAIRESKISVLVLSKDYASSRWCLDELAMIMERR‑RTDGHI‑VVPVFYDADPTEVGKQIGSYGEAFERH‑EKVFKEE‑‑‑ME‑‑MVEGWRAALREVAD‑MGGM‑‑‑VL‑E‑‑‑‑NRHQSQFIQNIV

>B9SBV5

GADTRHNLISHLYAALSRKHVTT‑FIDDHGLD‑RGEEISPTLLKAIEESKISVIIFSENYASSKWCLDELVKIMECM‑KTMSRN‑VLPVFYHVDPSDVRKQTGSFGQAFGVV‑KEKFKGS‑‑‑MD‑‑RVQRWSTALTEAAN‑LSGW‑‑‑DS‑‑‑‑‑‑NNLESELIEGVI

>B9RVW4

GEDTRKKFTSHLYKELCQKGIIT‑FKDDRELP‑KGEPF‑PELPKAIQDSRILVVVFSENYATSTWCLDELVKILECK‑KA‑GQT‑VLPIFYDVIPDEVREQDGKFGEPFIEY‑E‑IYKDN‑‑‑‑E‑‑KVQQWRVASTEIAN‑LSGW‑‑‑H‑LH‑‑‑‑DREEADFIQDIV

>B9RBV2

GEDTRDNFTSHLFAALSRKSVIT‑FMDNNDLH‑VGEEITPAISKAIEESKIAIVIFSERYAFSRWCLNEIVRIIECK‑ETCGQL‑VLPVFYHVGPSDV‑‑‑‑SVFAEAFPSY‑‑‑‑‑‑‑‑‑‑‑FE‑‑KVQKWKNALSKAAN‑LSAF‑‑‑DS‑R‑‑‑VTRPESKLVDEIV

>B9SHJ1

GKDIRDGFLSHLHEALRQSQINA‑FI‑DESIE‑RGKEISSSLLKIIEESHVSVVIFTENYADSPW‑‑‑‑‑‑‑‑‑‑‑‑‑‑‑‑‑‑‑‑‑‑‑‑‑‑‑‑‑‑‑‑‑‑‑‑‑‑‑‑‑‑‑‑‑‑‑‑‑‑‑‑‑‑‑‑‑‑‑‑‑‑‑‑‑‑‑‑‑‑‑‑‑‑‑‑‑‑‑‑‑‑‑‑‑‑‑‑‑‑‑‑‑‑PDSKLIGEVV

>B9SFT6

GEDTRKNFTDHLYNALLQAGIHA‑FRDDKHLS‑RGNHISSELLKAIQESKVSIVVFSKGYASSRWCLDELVKIMQCK‑‑TAGQI‑VVPIFYDVSPSDVRKQTGSFAEALQRH‑‑EQF‑‑‑‑‑‑SE‑‑RVNDWRNALLEAAN‑LSGW‑‑‑DL‑QN‑‑ANGHESKNIRKVV

>B9RYC7

GEDTRINFTSHLHDALLKNNILT‑FIDNE‑LV‑RGEALSPSLLKAIEESKISVVILSENYPYSKWCLEELVKILECM‑KINGQM‑VIPVFYKVDPSHVRNQTGSFADAFARH‑EESLLVT‑‑‑ED‑‑KVKSWRAALKDVAN‑ISGW‑‑‑DS‑‑‑‑‑VTSPESELIKKII

>B9S6Y9

GEDTRDNFTSHLYSALNKKKIFT‑FMDKE‑IK‑RGEEISPSIAKAIKGSKLSVIIFSEKYAFSKWCLDELTKILECK‑KMNGQI‑VIPVFYRVDPVHVRNQRGSFACAFAKH‑EETLKER‑‑‑ME‑‑KVESWRSALNEAGS‑ISGW‑‑‑NS‑‑‑‑‑VARPESKLIEEIV

>B9RVC7

GGDTRKNFTDHLYTALVQEGIHT‑FRDDDEIK‑RGEDIELEIQRAITESKLSVIVLSKDYASSRWCLDELVLIMERR‑KLVGHV‑VVPVFYDVEPYQVRNQTGSYGEAFAKH‑EKDFKED‑‑‑MS‑‑RVEEWRAALKEAAE‑LGGM‑‑‑VL‑Q‑‑‑‑DGYESQFIQTIV

>B9RBV1

GADTRQNFTSHLHFALCRKSIRT‑FIDD‑ELS‑RGEQITPALLEVVEESRIAVIIFSKNYGSSTFCLDEVAKIIEC‑NETHRQ‑‑VVPVFYHVDPLDVENQTGSFETAFAKH‑E‑‑‑‑‑‑‑‑‑FD‑‑RVQRWKAALSKAAS‑MAGW‑‑‑DS‑K‑‑‑‑VRMESQLVENIV

>B9S6Z6

DEDN‑‑NFVSHLYRKLSLEGIHT‑‑‑‑‑‑‑‑E‑NGKEF‑P‑‑‑‑AIQESRLIVVVLSEKYACSAQCLDELVKITDCE‑KTR‑KM‑VVPIFHNVDPDDLGNQRGKVAEAFAKH‑EEK‑‑‑‑‑‑‑‑E‑‑KVKMWKDALTKVAS‑ICGW‑‑‑DSLQ‑W‑‑‑‑EETIFIEQIV

>B9S2G3

GEDTRHTFTENLYRELIKHGVRT‑FRDDEELQ‑RGDEIAPSLLDAIEDSAAAIAVISKRYADSRWCLEELARIIECR‑‑‑‑‑RL‑LLPVFHQVDPSDVRKQTGPF‑RDFKRL‑EERFG‑‑‑‑‑VE‑‑KVGRWRNAMNKAGG‑ISGW‑‑‑‑‑‑DS‑K‑‑WEDEKLIESLV

>B9SBW2

GEDTRDNFTSHLYAALHQKQIKA‑FVDDK‑LS‑RGEEISAALVKVIEESMVSVIIFSENYAFSPWCLDELVKILECK‑KTVGQI‑VLPVFYHVDPSDVAEQKGGFGAAFIEH‑EKCFKER‑‑‑ID‑‑KLQKWRAALTEAAN‑ISGW‑‑‑SS‑S‑‑‑VIRSESKLIQEIA

>B9S6Z7

GKDTRDSFTNYLYKDLYQKGIET‑FIDNK‑LN‑RGEEITPELLKAIQESMVAVVVFSHNYADSPWCLDELVHIMECK‑RAHGQI‑VLPVFYRVDPSEVEEQIGEFGKGFDRA‑KKQANGD‑‑‑MR‑‑LVKKWKAALKDAAN‑LSGW‑‑‑DS‑S‑‑‑VVRPDSKLITEIV

>B9SXA8

GEDTRKNFTSHLYAALRQKGINA‑FKDDRQLE‑RGKTISQELVKAIRASKILMIIFSRNYAFSRWCLEEAVEIAECA‑KGNGQM‑VVPVFYNVNPNEVRKQTGDFGKAFGEH‑QLRFRNN‑‑‑LL‑‑TVQRWRLALTQLGS‑LSGW‑‑‑DL‑‑‑‑‑QERTESELIEEII

>B9S9D5

GEDTRNNFISHLHAALSRKSIRT‑FIDD‑ELR‑RGDEITRSLLKKIEESKIAVVIFSRNYASSTYCLDELEKIIEF‑HECYGQ‑‑VIPIFFNVNPSDLEPDTGIFAEALSRH‑EKKVQGDQEKLD‑‑KVQRWKVALKKAGN‑LSGH‑‑‑DL‑Q‑‑‑‑IRRESELVDKIV

>B9S9E4

GLDTRNAFLSHLFKALTEKQIIT‑FK‑DENLD‑RGERISNTLLQTIRESYVSVVIFSKNYACSTWCLEELVTILQCN‑EEMGQV‑VLPVFYEIDPTEVQELTGSYGNALMNH‑RKEFEDC‑‑‑S‑‑‑‑VESWSHALKKVGA‑MAGF‑‑‑VSTK‑‑‑‑‑‑PESKLIEEIV

>B9RM36

GEDTRKNFTSHLYAALCQKGVIT‑FRDDEELE‑RGKTISQALLQAIHGSKIAVIVFSRDYASSSWCLDELAEIHKCR‑KEKGQI‑VMPVFCNVNPYEVRKQAAGFGKAFAKH‑ELRFKND‑‑‑VQ‑‑KVQRWRAAISELAN‑LAGW‑‑‑DS‑‑‑‑‑LDRHESELIQEIV

>B9SHM4

GKDTRDNFTSHLYDALCRKKIKT‑FIDNG‑LE‑RGEEITPALLRTIEESLISVIVFSENYASSPWCLDEMVKILECR‑ETHGQA‑VLPVFYHVDPSDVEEQNGSFALTLVEL‑EKNFK‑‑‑‑‑‑D‑‑KVSKWRTDLMKAAS‑ISGW‑‑‑DS‑R‑‑‑AIGSEAKLVKHIV

>B9SX40

GADVRDGFLSHLHQSLDRNQVNA‑FV‑DEKLK‑RGKEITSSLLEIIEKSYVSIVIFSKNYADSPWCLDELVKIFECY‑KKMKQI‑VV‑‑‑‑‑‑‑‑‑‑‑‑‑‑‑‑‑‑‑‑‑‑‑‑‑‑‑‑‑‑‑‑‑‑‑‑‑‑‑‑‑‑‑‑‑‑‑‑‑‑‑‑‑‑‑‑‑‑‑‑‑‑‑‑‑R‑‑‑‑‑‑PDSRLIREIV

>B9SNY2

T‑STRFKFTSYVCDALERKKIEY‑FND‑‑TV‑‑RGREI‑‑‑‑‑KKIKDSWISIVIISE‑YA‑TKDCLDQLLIILEEK‑REKGEM‑VLPVFYLVSPDDVETQIGNFAKLFADL‑RQK‑‑‑‑‑‑‑‑HGAQMEKWGKALAEVG‑‑IGGR‑‑‑‑VLE‑‑‑‑NKSEAFFVTLII

>B9RIH0

IEDTCRSFVRNLYKHLEHKGLLC‑FKD‑‑KPE‑SGKPIPLDLLKAIEGSKIAVVVISQNYASSSWCLDELVKIIEC‑‑KEKGQ‑‑VFPIFHDVDPLQVKDQTGSFAQVLAEY‑EKD‑‑‑D‑‑MVE‑‑KAQRWRVALTKVAL‑IDGW‑‑‑NS‑R‑‑‑‑D‑DDHKLTEEVS

>K7MH68

GEDTRYGFTGNLYNVLRERGIHT‑FIDDEELQ‑KGDEITTALEEAIEKSKIFIIVLSENYAYSSFCLNELTHILNFT‑EGKNPL‑VLPVFYKVNPSYVRHHRGSYGEALANH‑EKKLNSN‑‑NME‑‑KLETWKMALRQVSN‑ISGH‑‑‑HL‑QH‑G‑NKYEYKFIKEIV

>K7MIY3

GEDTRYGFTGNLYRALSDKGIRT‑FFDEEKLH‑SGEEITPALLKAIKDSRIAITVLSEDFASSSFCLDELTSIVHCA‑QYNGMM‑IIPVFYKVYPSDVRHQKGTYGEALAKH‑KIRF‑‑‑P‑‑‑E‑‑KFQNWEMALRQVAD‑LSGF‑‑‑HF‑KY‑R‑DEYEYKFIERIV

>C6ZS22

GEDTRYTFTSHLYAALTRLQVKT‑YID‑NELE‑RGDEISPSLLRAIDDAKVAVIVFSENYASSRWCLDELVKIMECK‑RKNGQI‑IVPVFYHVDPTHVRHQTGSYGHAFAMH‑EQRFVGN‑‑‑MN‑‑KVQTWRLVLGEVAN‑ISGW‑‑‑DC‑L‑‑‑TTRVESELVEKIA

>K7LYT2

GEDTRRNFTCHLYEALMQKKIKT‑YIDE‑QLE‑KGDQIALALTKAIEDSCISIVIFSDNYASSKWCLGELFKILECK‑KEKGQI‑VIPVFYNIDPSHVRKQIGSYKQAFAKL‑EGEP‑‑‑‑‑‑‑‑‑‑ECNKWKDALTEAAN‑LVGL‑‑‑DS‑KN‑‑‑‑‑NDVELLKDIV

>K7MWR6

GEDTRHSFTGNLYKALSDRGIHT‑FIDDKKLP‑RGDQISSALEKAIEESRIFIIVLSENYASSSFCLNELGYILKFI‑KGKGLL‑VLPVFYKVDPSDVRNHAGSFGESLAHH‑EKKFNADKENLV‑‑KLETWKMALHQVAN‑LSGY‑‑‑HF‑KH‑G‑EEYEYKFIQRIV

>I1KEG1

GEDTRYEFTGHLHQALCKKGIRA‑FFDEEDLQ‑TGDEITTKLEEAIKGSRIAITVFSKGYASSSFCLNELATILGCY‑REKTLL‑VIPVFYKVDPSDVRHQRGSYEQGLDSL‑EKRLH‑‑‑‑‑‑P‑‑NMEKWRTALHEVAG‑FSGH‑‑‑HF‑TD‑G‑AGYEYQFIEKIV

>K7N1L0

GEDTRMNFTSHLHEALKQKKVET‑YIDY‑QLE‑KGDEISPALIKAIEDSHVSIVILSENYASSKWCLEELSKILECK‑KKQGQI‑VIPVFHNIDPSHVRKQNGSYEKAFAKH‑EGEA‑‑‑‑‑‑‑‑‑‑KCNKWKATLTEVAN‑LAGW‑‑‑DS‑R‑‑‑‑‑‑TESELLKDIV

>K7KXK5

GEDTRNNFTAFLFDALFENGIHA‑FKDDTHLQ‑KGESIAPELLLAIQESRLFLVVFSKNYASSTWCLRELAHICNCTIEPSSS‑‑VLPIFYDVDPSEVRKQSGYYGIAFAEH‑ERRFREDIEKME‑‑EVQRWREALIQVAN‑ISGW‑‑‑DI‑Q‑‑‑‑NESQPAMIKEIV

>I1JM77

GEDTRRSFVCHLNCALSKAGVKT‑FLDEENLH‑KGMKL‑DELMTAIEGSQIAIVVFSKSYTESTWCLRELEKVIECN‑ETYGQS‑VLPVFYNIDPSVVRHEKHDFGKVLKST‑EKNYSGE‑‑HLE‑‑NLSRWSRALSEASK‑FSGW‑‑‑DA‑S‑‑‑‑KRNDAELVEKIV

>K7L9U4

GDDTRSGFTGSLYKSLCDQGIHT‑FMDDEGLR‑RGEEIRHALFKAIQQSRIAIVVFSENYASSTYCLEELVMILECI‑MKKGRL‑VWPVFYGVTPSYVRHQKGSYGKALDKL‑GERF‑‑‑KNDKE‑‑KLQKWKLALQEAAN‑LSGS‑‑‑HF‑KL‑K‑HGYEHEVIQKIV

>Q84ZV1

GEDTRYGFTSNLYRALSDKGIRT‑FFDEEKLH‑SGEEITPALLKAIKDSRIAITVLSEDFASSSFCLDELTSIVHCA‑QYNGMM‑IIPVFYKVYPSDVRHQKGTYGEALAKH‑KIRF‑‑‑P‑‑‑E‑‑KFQNWEMALRQVAD‑LSGF‑‑‑HF‑KY‑R‑DEYEYKFIERIV

>K7MHM7

GKDTRQNFTGHLYNSLFKNGILT‑FIDDKGLR‑RGEEITPALLNAIKNSRIAIIVFSEDYASSTYCLDELVTILESF‑KEEGRS‑IYPIFYYVDPSQVRHQTGTYSDALAKH‑EERFQYD‑‑I‑D‑‑KVQQWRQALYQAAN‑LSGW‑‑‑HF‑HG‑S‑‑QPEYKFILKIV

>I1KEB4

GEDTRNSFTAFLFGALKKQGIEA‑FKDDKDIR‑KGESIAPELIRAIEGSHVFVVVFSKDYASSTWCLRELAHIWDC‑IQTSHR‑‑LLPIFYDVDPSQVRNQSGDYEKAFAQH‑QQRFQE‑‑‑‑‑K‑‑EIKTWREVLEQVAS‑LSGW‑‑‑DI‑R‑‑‑‑NKQQHPVIEEIV

>K7MH74

GEDTRYCFTGNLYNVLRERGIHT‑FIDDDELQ‑KGDQITSALQEAIEKSKIFIIVLSENYASSSFCLNELTHILNFT‑KGKNLL‑VLPVFYIVDPSDVRHHRGSFGEALANH‑EKKLNST‑‑NME‑‑NLETWKIALHQVSN‑ISGY‑‑‑HF‑QH‑G‑DKYEYKFIKEIV

>K7KCW4

GEDIRHGFLGYLTEAFHQKQIHA‑FIDD‑KLE‑KGDEIWPSLVGAIQGSLISLTIFSENYSSSRWCLEELVKIIECR‑ETYGQT‑VIPVFYHVNPTDVRHQKGSYEKALSEH‑EKKYNLT‑‑‑‑‑‑‑TVQNWRHALKKAAD‑LSGI‑‑‑KS‑‑‑‑‑‑‑‑TEVELLGEIV

>K7LUI8

GLDTRNSFTDHLFAALQRKGIFA‑FRDNQNIN‑KGELLEPELLQAIEGSHVFIVVFSKDYASSTWCLKELRKIFDG‑VEETGR‑‑VLPIFYDVTPSEVRKQSGKFGKAFAEH‑EERFK‑‑‑DELE‑‑MVKKWREALKAIGN‑RSGW‑‑‑DV‑Q‑‑‑‑NKPEHEEIEKIV

>I1M0Q0

GKDTRFTFTGNLYRALQDRG‑RV‑FMDDQKID‑KGKKIS‑ELPKAIKESRIYIIVLSENFASSWYCLVEVVMILDEA‑KGKGR‑‑IVPIFFYVDPSVLV‑‑‑RTYEQALADQ‑RKW‑‑‑‑‑‑‑SD‑‑KIEEWRTALTKLSK‑FPGF‑‑‑CVSR‑‑‑‑DNFEYQHIDEIV

>K7LW75

GPDTRNTFVDHLYAHLLRKGIFV‑FKDDKKLQ‑KGESISAQLLQAIQDSRLSIIVFSKQYASSTWCLDEMAAIADCK‑QQN‑QT‑VFPVFYDVDPSHVRHQNGAYEVAFVSH‑RSRFRE‑‑‑DPD‑‑KVDRWARAMTDLAN‑SAGW‑‑‑DV‑M‑‑‑‑NKPEFREIENIV

>K7KDW7

GEDIRKNFVSHLHSALLHAEVKT‑FLDDENLL‑KGMKS‑EELIRAIEGSQIAVVVFSKTYTESSLCLRELEKIIESH‑ETRGQR‑VLPIFYEVDPSDVRQQKGDFGEALKAA‑QKGFSGE‑‑HLE‑‑SLSRWSQAITKAAN‑LPGW‑‑‑DE‑S‑‑‑‑NENDAELVEGII

>K7KQM9

ATDTRSNFTDFLFQALIRKGIVA‑FKD‑‑‑‑ESR‑‑‑‑AP‑‑‑QAIEDSRLFIVVLSKNYAFSTQCLHELSQIFHC‑VEF‑SR‑‑VLPIFYDVDPSDVRKQTG‑Y‑KAFSKY‑EERFLVNKKGMET‑‑VQTWRKALTQVAN‑LSGW‑‑‑‑YIR‑‑‑‑NKTEIEFVYTI‑

>K7KCV9

‑‑‑LETNYGHHLL‑‑‑‑‑‑‑‑‑‑‑‑‑‑‑‑‑‑‑‑‑‑‑‑‑‑‑‑‑‑‑‑‑‑GSLISLTILSENYASSSWSLNELVTILECR‑EKYNRI‑VIPVFYKVYPTDVRHQNGSYKSDFAEH‑EKKYNLA‑‑‑‑‑‑‑TVQNWRHALSKAAN‑LSGI‑‑‑KS‑‑‑‑‑‑‑‑TEVELLEKIV

>K7KV37

GSDTRHGFTGNLYKALADRGIYT‑FIDDEELQ‑SGKEITPTLLKAIQESRIAINALSINYASSSFCLDELATILGCA‑ERKTLL‑VL‑‑‑‑‑‑‑PSHVRHREDSYGEALVKH‑EERF‑‑‑EHNTE‑‑KLQKWKMTLYQVAL‑LSGY‑‑‑HF‑KY‑G‑DGYEYEFIGRIV

>K7LE88

GEDTRHGFTGHLYSALHSKGIHT‑FIDDEGLQ‑RGEEITPALVKAIQESKIAIIVLSINYASSSFCLHELATILECL‑MGKGRL‑VLPVFYKVDPSHVRHQNGSYEEALAKH‑EERF‑‑‑KAEKE‑‑KLQKWKMALHQVAN‑LSGY‑‑‑HF‑KD‑G‑EGYEYKFIEKIV

>K7KDW4

GEDTRKKFVCHIYKALSNAGINT‑FIDEENIQ‑KGMTL‑DELMTAIEGSQIAIVVFSKTYTESTWCLRELQKIIECH‑ENYGQR‑VVPVFYHIDPSHIRHQEGDFGSALNAV‑ERRHSGE‑‑DLK‑‑SLSNWKRVLKKATD‑FSGW‑‑‑NE‑R‑‑‑‑DRNDAELVKEIV

>K7MH79

GEDTRHGFTGNLYNVLRERGIDT‑FIDDEELQ‑KGHEITKALEEAIEKSKIFIIVLSENYASSSFCLNELTHILNFT‑KGKSRS‑ILPVFYKVDPSDVRYHRGSFGEALANH‑EKKLKSN‑‑YME‑‑KLQIWKMALQQVSN‑FSGH‑‑‑HF‑QP‑G‑DKYEYDFIKEIV

>K7KXJ5

GEDTRNSFTAFLFDALSQNGIHA‑FKDDTHLQ‑KGESIAPELLLAIQGSGLFVVVFSKNYASSTWCLRELAHICNCTIQASPS‑‑VLPIFYDVDPSELRKQSGYYGIAFAEH‑ERRFRGDKEKME‑‑ELQRWREALKQVAN‑ISGW‑‑‑NI‑Q‑‑‑‑NESQPAVIEKIV

>I1MND6

REDTRHGFTGNLYNVLRERGIHT‑FIDDDEPQ‑KADQITKALEEAIKNSKIFIIVLSENYASSFFCLNELTHILNFT‑KGWDVL‑VLPVFYKVDPSDVRHHRGSFGEALANH‑EKNLNSN‑‑YMG‑‑KLKTWKMALRQVSN‑FSGH‑‑‑HF‑QP‑G‑NKYEYKFIKEIL

>K7KBB3

GEDTRNNFIGHLRKELSRKGMKI‑FFDDRDL‑‑PGNVISPSLSKAIEESKILIIVFSKNYASSTWCLDELVKILEQKISEMKQL‑VFPVFYHVDPSDVRKQTESYGEHMTKH‑EENFGKS‑‑‑‑Q‑‑KLQAWRTALFEASN‑FPGH‑‑‑H‑IT‑‑‑‑TRYEIDFIEKIV

>K7K6Z8

GEDTRQKFTGNLYNSLCEKGVHT‑FIDDEGLR‑RGEEITPALLNAIQNSRIAIVVFSKNYASSTFCLDKLVKILECL‑KEEGRS‑VFPIFYDVDPSHVRHQKGTYSEALAKH‑EERFPDD‑‑S‑D‑‑KVQKWRKALYEAAN‑LSGW‑‑‑HF‑QH‑G‑‑ELEYKSIRKIV

>K7KDV6

GEDTRRSFVCHLNCALSKAGVKT‑FLDEENLH‑KGMKL‑DELMTAIEGSQIAIVVFSKSYTESTWCLRELEKVIECN‑ETYGQS‑VLPVFYNIDPSVVRHEKHDFGKVLKST‑EKNYSGE‑‑HLE‑‑NLSRWSRALSEASK‑FSGW‑‑‑DA‑S‑‑‑‑KRNDAELVEKIV

>K7MIM5

GLDTRHGFTGNLYKALDDRGIYT‑FIDDQELP‑RGDQITPALSKAIQESRIAITVLSENYASSSFCLDELVTILHCK‑S‑EGLL‑VIPVFYKVDPSDVRHQKGSYGEAMAKH‑QKSF‑‑‑KAKKE‑‑KLQKWRMALQQVAD‑LSGY‑‑‑HF‑KD‑G‑DAYEYKFIGSIV

>I1J5B8

GEDTRDNFISHIYAELQRNKIET‑YIDY‑RLA‑RGEEISPALHKAIEESMIYVVVFSQNYASSTWCLDELTKILNCK‑KRYGRV‑VIPVFYKVDPSIVRNQRETYAEAFVKY‑KHRFADN‑‑‑ID‑‑KVHAWKAALTEAAE‑IAGW‑‑‑DS‑Q‑‑‑‑‑‑PEATLVAEIV

>K7MIZ0

GEDTRHGFTGNLYKALDDRGIYT‑FIDDQELP‑RGDQITSALSKAIQESRIAITVLSQNYASSSFCLDELVTILHCK‑R‑KGLL‑VIPVFYKIDPSDVRHQKGSYGEAMAKH‑QKSF‑‑‑KAK‑‑‑‑KLQKWRMALQQVAD‑LSGY‑‑‑HF‑KD‑G‑DSYEYKFIGSIV

>K7MDD3

GEDTRLGFVGNLYKALTEKGFHT‑FFRE‑KLV‑RGEEIAASPSKAIQHSRVFVVVFSQNYASSTRCLEELLSILR‑F‑QDNRRP‑VLPVFYYVDPSDVGLQTGIYGEALAMH‑EKRF‑‑‑NSESD‑‑KVMKWRKALCEAAA‑LSGW‑‑‑PF‑KH‑G‑DGYEYELIEKIV

>I1LWA0

GEDTRKNFTSHLYEALKQKKIET‑YIDY‑RLE‑KGDEISAALIKAIEDSHVSVVIFSENYASSKWCLGELGKIMECK‑KERGQI‑VIPVFYNIDPSHVRKQTGSYEQSFAKH‑TGEP‑‑‑‑‑‑‑‑‑‑RCSKWKAALTEAAN‑LAAW‑‑‑DS‑QI‑‑‑‑‑TESEFLKDIV

>I1MND4

GEDTRYGFTGYLYNVLRERGIHT‑FIDDDEPQ‑EGDEITTALEAAIEKSKIFIIVLSENYASSSFCLNSLTHILNFT‑KENNVL‑VLPVFYRVNPSDVRHHRGSFGEALANH‑EKKSNSN‑‑NME‑‑KLETWKMALHQVSN‑ISGH‑‑‑HF‑QH‑G‑NKYEYKFIKEIV

>K7MWS1

GKDTRHSFTGNLYKALSERGINT‑FIDDKKLP‑RGDEITSALEKAIEESRIFIIVLSENYAWSSFCLNELDYILKFI‑KGKGLL‑VLPVFYKVDPSDVRNHTGSFGESLAYH‑EKKFKSTN‑NME‑‑KLETWKMALNQVAN‑LSGY‑‑HHF‑KH‑G‑EEYEYQFIQRIV

>I1M0P9

GEDTRRSFTGNLYNCLEKRGIHT‑FIGDYDFE‑SGEEIKASLSEAIEHSRVFVIVFSENYASSSWCLDGLVRILD‑F‑EDNHRP‑VIPVFFDVEPSHVRHQKGIYGEALAMH‑ERRL‑‑‑NPESY‑‑KVMKWRNALRQAAN‑LSGY‑‑‑AF‑KH‑G‑DGYEYKLIEKIV

>K7L9P5

GIDTRDTITKGLYSSLEARGVRV‑FLDDVGLE‑RGEEIKQGLMEAIDDSAAFIVIISESYATSHWCLEELTKICDTG‑‑‑‑‑RL‑VLPVFYRVDPSHVRDQKGPF‑AGFVEH‑ERRFG‑‑‑‑‑KN‑‑EVSMWREAFNKLGG‑VSGW‑‑‑‑PFND‑S‑‑‑EEDTLIRLLV

>I1L1A5

GQDIRDGFLSHLIDTFERKKINF‑FVDY‑NLE‑KGDEIWPSLVGAIRGSLILLVIFSPDYASSCWCLEELVKILECR‑EEYGRI‑VIPVFYHIQPTHVRHQLGSYAEAFAVH‑GRKQM‑M‑‑‑‑‑‑‑KVQHWRHALNKSAD‑LAGI‑‑‑DS‑‑‑‑‑‑‑‑NDAAVLNEIV

>I1N650

GSDTRHGFVGNLYKALNDKGIHT‑FIDDEKLQ‑GGEEITPTLMKAIEESQIAITVLSHNYASSSFCLDELVHIIDC‑‑KRKGLL‑VLPVFYNLDPSDVRHQKGSYGEALARH‑EERFKAKNQNME‑‑RLEKWKMALHQVAN‑LSGY‑‑‑HF‑KQ‑G‑DGYEYEFIGKIV

>K7LWN8

GIDTRHSFTDNLYNSLKQRGIHA‑FIDDEGLR‑RGEEITPTLLKAIRESRIGIIVFSKSYASSTYCLDELVEILECL‑KVEGRL‑VWPVFYDVDPSQVRYQTGTYAEALAKH‑KERFQDD‑‑K‑G‑‑KVQKWRKALHEAAN‑LSGW‑‑‑HF‑QH‑G‑SESEYKFIKKIV

>K7K614

SEDTRKTFTSHLNAALERLDIKT‑YLDNNNLD‑RGEEIPTTLVRAIEEAKLSVIVFSKNYADSKWCLDELLKILEFG‑RAKTLI‑IMPVFYDIDPSDVRNQRGTYAEAFDKH‑ERYFQEK‑‑‑K‑‑‑KLQEWRKGLVEAAN‑YSGW‑‑‑DC‑D‑‑‑VNRTESEIVEEIA

>K7MH77

GEDTRHGFTGNLYNVLRERGIDT‑FIDDEELQ‑KGHEITKALEEAIEKSKIFIIVLSENYASSSFCLNELTHILNFT‑KGKSRS‑ILPVFYKVDPSDVRYHRGSFGEALANH‑EKKLKSN‑‑YME‑‑KLQIWKMALQQVSN‑FSGH‑‑‑HF‑QP‑G‑DKYEYDFIKEIV

>I1NDU4

GEDTRHTFTCKLYDALWLKGIDT‑FMDNKELK‑NGDKIGPTLHKAIEEARISVVVLSENYADSSWCLDELVKIHECM‑ESKNQL‑VWPIFYKVNPSDVRHQKGSYGVAMTKH‑ETSP‑‑‑‑‑DLE‑‑KVHKWRSTLNEIAN‑LKGK‑‑‑YL‑EE‑G‑‑‑‑ESKFIDDLA

>K7KYE4

GEDTRRTFTGSLYHGLHQRGINV‑FIDDEKLR‑RGEEISPALIGAIEESRIAIIVFSQNYASSTWCLDELAKILECY‑KTRGQL‑VWPVFFHVDPSAVRHQRGSFATAMAKH‑EDRF‑‑‑KGDVQ‑‑KLQKWKMALFEAAN‑LSGW‑‑‑TL‑K‑‑‑‑NGYEFKLIQEII

>K7MMQ0

GEDTRRNTTSHLYEALLHKKIES‑YIDY‑QLE‑KGDEIT‑‑LIQAIKD‑‑‑SAYLFS‑‑‑FSQRTML‑‑‑‑‑‑‑‑‑‑‑PQSGQI‑VIPVFYNIYPSHVRKKTGSYDQAFAKH‑VGEP‑‑‑‑‑‑‑‑‑‑RCNKWKAALTEAAN‑LAGW‑‑‑DS‑QT‑‑‑‑‑TDSELLKDII

>I1NDU3

GEDTRHTFTCKLYDALWLKGIDT‑FMDNKELK‑NGDKIGPTLHKAIEEARISVVVLSENYADSSWCLDELVKIHECM‑ESKNQL‑VWPIFYKVNPSDVRHQKGSYGVAMTKH‑ETSP‑‑‑‑‑DLE‑‑KVHKWRSTLNEIAN‑LKGK‑‑‑YL‑EE‑G‑‑‑‑ESKFIDDLA

>K7L9P4

GIDTRDTITKGLYSSLEARGVRV‑FLDDVGLE‑RGEEIKQGLMEAIDDSAAFIVIISESYATSHWCLEELTKICDTG‑‑‑‑‑RL‑VLPVFYRVDPSHVRDQKGPF‑AGFVEH‑ERRFG‑‑‑‑‑KN‑‑EVSMWREAFNKLGG‑VSGW‑‑‑‑PFND‑S‑‑‑EEDTLIRLLV

>K7MH72

GEDTRYGFTGNLYNVLRERGIHT‑FIDDDELQ‑KGDEITTALEEAIEKSKIFIIVLSENYASSSFCLNELTHILNFT‑EGKNRL‑VLPVFYKVNPSIVRKHRGSYGEALANH‑EKKLNSN‑‑NME‑‑KLETWKMALQQVSN‑ISGH‑‑‑HF‑QH‑G‑GKYEYKFIKEIV

>K7LC01

GVDIRRGFLSHLIGTFKSKQINA‑FVDD‑KLE‑RGEEIWPSLIEAIQGSSISLIIFSPDYASSRWCLEELVTILECK‑EKYGQI‑VIPIFYHIEPTEVRHQRGSYENAFAEH‑VKKYK‑S‑‑‑‑‑‑‑KVQIWRHAMNKSVD‑LSGI‑‑‑ES‑‑‑‑‑‑‑‑DDDELLKEIV

>K7KXN2

GEDTRYEFTGHLHKAHCNKGIRA‑FIDEDDLE‑RGDEITTTLEEAIKGSRIAITVFSKDYASSSFCLDELVTIFGCY‑PE‑‑‑‑‑‑‑‑‑‑‑‑‑‑‑‑‑‑‑‑‑‑‑‑‑‑‑‑‑‑‑‑‑‑‑‑‑‑‑‑‑‑‑‑‑‑‑‑‑‑‑‑‑‑‑‑‑‑‑‑‑‑‑‑‑‑‑‑‑‑‑‑‑‑‑‑‑‑‑YEYKFIGKIV

>K7MIT6

GEDTRHAFTGHLYKALHDKGIHT‑FIDDEKLQ‑RGEQITRALMEAIQDSRVAITVLSQNYASSSFCLDELATILHCH‑QRKRLL‑VIPVFYKVDPSDVRHQKGSYAEALEKL‑ETRF‑‑‑QHDPE‑‑KLQKWKMALKQVAD‑LSGY‑‑‑HF‑KE‑G‑DGYEFKFIEKIV

>K7K3H1

GKDIRDGFLGYLTRAFHQKQIYA‑FIDD‑KLE‑KGDEIWPSLVGAIQGSSISLTIFSENYTSSRWCLEELVKILECR‑EKYRQT‑VIPVFYGVNPTDVRHQKGNYGEALAVL‑GKKYNLT‑‑‑‑‑‑‑TVQNWRNALKKAAD‑LSGI‑‑‑KS‑‑‑‑‑‑‑‑TEVDLLGEIV

>K7L036

GDDTRKGFTHNLFASLERRGIKA‑YRDDHDLE‑RGKVISVELIEAIEESMFALIILSSNYASSTWCLDELQKILECK‑K‑‑‑E‑‑VFPIFLGVDPSDVRHQRGSFAKAFRDH‑EEKFREE‑‑‑KK‑‑KVETWRHALREVAS‑YSGW‑‑‑DS‑‑‑‑‑KD‑HEAALIETIV

>K7LF16

GEDTRGDFTSHLHAALCRNGIQT‑YID‑YRIQ‑KGYEVWPQLVKAIRESTLLLVIFSENYSSSSWCLNELVELMECK‑KQE‑DVH‑‑‑‑‑‑‑‑‑‑‑‑VRKQSGSYHTALAKH‑KKDWKVS‑‑‑‑‑‑‑KMQKWKDALFEAAN‑LSGF‑‑‑HS‑‑‑‑‑‑‑‑TEPDLIEDII

>I1MQH8

GEDTRYGFTGNLYKALCDKGIHT‑FFDEDKLH‑SGDDITPALSKAIQESRIAITVLSQNYASSSFCLDELVTILHCK‑R‑EGLL‑VIPVFHNVDPSAVRHLKGSYGEAMAKH‑QKRF‑‑‑KAKKE‑‑KLQKWRMALHQVAD‑LSGY‑‑‑HF‑KD‑G‑DAYEYKFIGNIV

>K7N1L2

GEDTRMNFTSHLHEALKQKKVET‑YIDY‑QLE‑KGDEISPALIKAIEDSHVSIVILSENYASSKWCLEELSKILECK‑KKQGQI‑VIPVFHNIDPSHVRKQNGSYEKAFAKH‑EGEA‑‑‑‑‑‑‑‑‑‑KCNKWKATLTEVAN‑LAGW‑‑‑DS‑R‑‑‑‑‑‑TESELLKDIV

>I1MKU1

GDDTRKGFTGHLFASLERRGIKT‑FKDDHDLQ‑RGKLISVELMKAIEGSMLALIILSPNYASSTWCLDELKKILECK‑K‑‑‑E‑‑VFPIFHGVDPSDVRHQRGSFAKAFSEH‑EEKFRED‑‑‑KK‑‑KLERWRHALREVAS‑YSGW‑‑‑DS‑‑‑‑‑KE‑HEATLIETIV

>K7L9Q3

GEDTRKTFTSHLHAAFKRMEINT‑YID‑YNLE‑RGDEISGTLLRAIEDAKLSVIVFSKNFGTSKWCLDEVKKIMECK‑KTRRQM‑VVPVFYDIEPTHVRNQTGSFASAFARH‑EERFMDR‑‑‑PN‑‑KVQKWKDALREATN‑LSGW‑‑‑DC‑S‑‑‑VDRLESEIVEEIA

>K7K1U0

GEDTRLGFTGHLYHALCEVGVNT‑FMDDQGLR‑KGEEITPFLMKAIQESRIAIVIFSENYASSTFCLQELVMIMECL‑KHQGRL‑VWPVFYKVDPSDVRHQKGSYAEALAKH‑ETRI‑‑‑S‑DKD‑‑KVEKWRLALQKAAS‑LSGW‑‑‑HS‑NR‑‑‑‑RYEYDIIRDIV

>K7KDI2

GEDTRATFTSHLYAALQNAGIIV‑FKDDESLP‑RGDQISDSLLLAIEQSQISVVVFSTNYADSRWCLQELEKIMNCK‑RTIGQV‑VLPVFYDVDPSQVRYQTGHFGESFQNL‑DDE‑‑‑‑‑‑‑‑‑‑‑‑ISRWRKVLREAAS‑IAGV‑‑‑VV‑‑‑‑‑LNSNESETIKNIV

>K7KW83

GEDTPNNFTGFLFNALRKKGIDA‑FRDDTDIK‑KGESIAPELLQAIEGSRIFVVVFSKSYASSTWCLCELAKICK‑YIDTSER‑‑VLPVFYDVDPSEVGKQSGYYEKAFAEH‑EETFGEDKEKIE‑‑EVPGWREALTRVTN‑LSGW‑‑‑DI‑G‑‑‑‑NKPQYAKVEEIV

>Q8H6S7

GEDTRFAFTGHLHKALCNKGIRA‑FMDENDIK‑RGDEIRATLEEAIKGSRIAITVFSKDYASSSFCLDELATILGCY‑REKTLL‑VIPVFYKVDPSDVRRLQGSYAEGLARL‑EERFH‑‑‑‑‑‑P‑‑NMENWKKALQKVAE‑LAGH‑‑‑HF‑KD‑G‑AGYEFKFIRKIV

>K7K1J3

GEDTRDNFIRHIYEQLQRKKIET‑YIDY‑RLS‑RGQEISPALHRAIEESMIYVVVFSENYASSTWCLDELTKILDCK‑KRYGRV‑VIPVFYKVDPSIVRNQRETYAEAFVKH‑EHRFQDK‑‑‑FD‑‑KVHGWKAALTEAAG‑LSGW‑‑‑DS‑Q‑‑‑‑‑‑PEATLVAEIV

>K7MWR9

GKDTRHSFTGNLYKALSERGINT‑FIDDKKLP‑RGDEITSALEKAIEESRIFIIVLSENYAWSSFCLNELDYILKFI‑KGKGLL‑VLPVFYKVDPSDVRNHTGSFGESLAYH‑EKKFKSTN‑NME‑‑KLETWKMALNQVAN‑LSGY‑‑HHF‑KH‑G‑EEYEYQFIQRIV

>K7KXK8

GLDTRNNFAALLLQALHRNGIDA‑FNDNVHVM‑KGEFIESELYMAIDGSRNFIVVFTKNYASSTWCLHELARICMN‑IETSTR‑‑ILPIFYVVDPLKVQKQSGCYEKAFMDY‑EERFRGAKER‑E‑‑QVWRWRKGLKQVSH‑LPCL‑‑‑HI‑Q‑‑‑‑NDLQQAEIEEIL

>K7K360

GKDTRASFTSHLYAALKNAGITV‑FKDDETLS‑RGKHISHSLRLGIEQSRISVVVFSRNYAESRWCLQELEKIMECH‑RTTGQV‑VLPVFYDVDPSQVRHQKSHFGKAFEKL‑IGDWREA‑‑TISGSDIQSWKDALHKAAG‑ISGV‑‑‑VV‑‑‑‑‑QNFNESEAIKHIV

>I1J5H4

GTDTRDTFTMSLYHALHRRGLRV‑FRDDDGLE‑RGDEIQKKLLEAIEDSAAAVVVLSPDYASSHWCLDELAKICKCG‑‑‑‑‑RL‑ILPVFYWVDPSHVRKQKGPF‑DSFGSH‑ANKFP‑‑‑‑‑EE‑‑SVQQWRDAMKKVGG‑IAGY‑‑‑‑VLDE‑KCDEKSDKLIQHLV

>K7LV88

GEDTRNSFTGFLFQALSRKGIDA‑FKDGKDLK‑KGESIAPELIQAIQGSRLFIVVFSNNYAFST‑‑‑‑‑‑‑‑‑‑‑‑‑‑‑‑‑‑‑‑‑‑‑‑‑‑‑‑‑‑‑‑‑‑‑‑‑‑‑‑‑‑‑‑‑‑‑‑‑‑‑‑‑‑‑‑‑‑‑‑‑‑‑‑‑‑‑‑‑‑‑‑‑‑‑‑‑‑‑‑‑‑‑‑‑‑I‑R‑‑‑‑KKLQYAEIEDL‑

>K7KA46

GSDTRFGFTGNLYKALHDRGFQT‑FIDDEWLS‑MRKAIISNM‑DALHFTDLLL‑‑‑‑‑HY‑‑‑‑FCMIEF‑‑‑‑‑‑‑‑‑‑‑‑‑‑‑‑‑‑‑‑‑‑‑‑‑‑‑‑‑‑‑‑‑QLSEDL‑‑‑‑‑‑‑‑‑‑‑‑‑‑‑‑‑‑‑‑‑‑‑‑‑‑‑‑‑‑‑‑‑‑‑‑‑‑‑‑KF‑KR‑S‑NGYESKFIERIV

>K7N1K4

GEDTRMNFTSHLHEALKQKKVET‑YIDY‑QLE‑KGDEISPALIKAIEDSHVSIVILSENYASSKWCLEELSKILECK‑KKQGQI‑VIPVFHNIDPSHVRKQNGSYEKAFAKH‑EGEA‑‑‑‑‑‑‑‑‑‑KCNKWKATLTEVAN‑LAGW‑‑‑DS‑R‑‑‑‑‑‑TESELLKDIV

>D6PT14

GADTRHGFTGNLYKALDDRGIYT‑FIDDEELQ‑SGEEITPALLKAIQESRIAITVLSINYASSSFCLDELAYILECF‑KSKNLL‑VVPVFYNVDPSDVRHQKGSYGEALAKH‑QERF‑‑‑NHNME‑‑KLEYWKKALHQVAN‑LSGF‑‑‑HF‑KH‑G‑EGYEYEFIGRIV

>K7K1I5

GEDTRKIITSHLYHALFQAELAT‑YIDY‑RLQ‑KGDEISQALIEAIEESQVSVIIFSEKYATSKWCLDEITKIIECK‑EGQGQV‑VIPVFYKIDPSHIRKQQGSFKQAFVEH‑EQDLKIT‑‑‑TD‑‑RVQKWREALTKAAN‑LAGW‑‑‑DF‑QT‑‑‑‑‑TEAEFIKDIV

>K7L0Z9

GDDTRSDFASHLHAALRRNNVDT‑YID‑YRIE‑KGAKIWLEIERAIKDSTLFLVIFSENYASSSWCLNELLQLMQCK‑KQE‑NVHVIPVFYKIDPSQVRKQSENYHVAFAKH‑KKDGKVS‑‑‑‑‑‑‑EMQKWKDALSEAAN‑LSGF‑‑‑HS‑‑‑‑‑‑‑‑TEPDLIEDII

>K7MH08

GEDTRLGFTGHLYKALHDKGIRT‑FIDDAELQ‑RGEEITPALMKAIQDSRVAITVLSEDYASSSFCLDELATIL‑‑D‑QRKRLM‑VIPVFYKVDPSDVRNQRGSYEDALAKL‑EGKF‑‑‑QHDPE‑‑KLQKWKMALKQVAN‑LSGY‑‑‑HF‑KE‑G‑DGYEFEFIEKIV

>K7KCW2

GEDIRHGFLGYLTEAFHQKQIHA‑FIDD‑KLE‑KGDEIWPSLVGAIQGSLISLTIFSENYSSSRWCLEELVKIIECR‑ETYGQT‑VIPVFYHVNPTDVRHQKGSYEKALSEH‑EKKYNLT‑‑‑‑‑‑‑TVQNWRHALKKAAD‑LSGI‑‑‑KS‑‑‑‑‑‑‑‑TEVELLGEIV

>K7N1K8

GEDTRMNFTSHLHEALKQKKVET‑YIDY‑QLE‑KGDEISPALIKAIEDSHVSIVILSENYASSKWCLEELSKILECK‑KKQGQI‑VIPVFHNIDPSHVRKQNGSYEKAFAKH‑EGEA‑‑‑‑‑‑‑‑‑‑KCNKWKATLTEVAN‑LAGW‑‑‑DS‑R‑‑‑‑‑‑TESELLKDIV

>K7L7M1

GEDTRGDFTSHLHAALGRSSIET‑YID‑YRIQ‑KGEEVWVELVKAIKGSTLFLVIFSENYANSSWCLNELVELMECR‑KQE‑EVHVIPVFYKIDPSQVRKQTGSYRAAVAN‑‑‑‑‑‑‑‑‑‑‑‑‑‑‑‑‑‑QKWKDALYEAAN‑LSGF‑‑‑HS‑‑‑‑‑‑‑‑TETDLIEDII

>K7LWN5

G‑GTRYGFTNRLYNALRQKGIYT‑FRDTEEL‑‑RGADIRPALLKAIENSRMSMVVLCEDYASSTWCLDELAKIIQCH‑ANKPKQ‑VLLIFYKVQPSDVWDQKNSYAKAMADH‑ENRFAKP‑‑‑‑E‑‑KVKNWRKALSQLRH‑LTRE‑‑‑Y‑CK‑‑‑‑D‑YEAELIKKIV

>K7MBP2

GKDIRDGFLSHLTDTFLRKKINV‑FVDETNLK‑KGDEIWPSLAVAIEVSSISLIIFSQDYASSRWCLEELVKILECR‑EKYGRI‑VIPIFYHVQPKNVRHQLGSYENIFAQR‑GRKYK‑T‑‑‑‑‑‑‑KVQIWKDALNISAD‑LSGV‑‑‑ES‑‑‑‑‑‑‑‑NDAELIQEIV

>K7KDW6

GEDIRKNFVSHLHSALLHAEVKT‑FLDDENLL‑KGMKS‑EELIRAIEGSQIAVVVFSKTYTESSLCLRELEKIIESH‑ETRGQR‑VLPIFYEVDPSDVRQQKGDFGEALKAA‑QKGFSGE‑‑HLE‑‑SLSRWSQAITKAAN‑LPGW‑‑‑DE‑S‑‑‑‑NENDAELVEGII

>K7K6E2

GTDVRKGLLSHLKTELRRRQI‑‑‑‑‑DDERLD‑RGDEISSSLLRAIEESQISLVIFSKDYASSQWCLEELAKMIES‑MEIK‑QI‑VLPVFFNVDPSHVRHQCGDYGDALAKH‑EEKLKE‑‑‑NM‑‑‑KVKTWRSAMKKAAD‑LSGF‑‑‑HYTN‑F‑‑E‑DESDLVHGIV

>I1J777

GKDTRASFTSHLYAALKNAGITV‑FKDDETLS‑RGKHISHSLRLGIEQSRISVVVFSRNYAESRWCLQELEKIMECH‑RTTGQV‑VLPVFYDVDPSQVRHQKSHFGKAFEKL‑IGDWREA‑‑TISGSDIQSWKDALHKAAG‑IS‑‑‑‑‑‑‑‑‑‑‑‑‑‑‑NESEAIKHIV

>I1MNC5

GEDTRYSFTGNLYNVLRERGIHT‑FIDDDEFQ‑KGDQITSALEEAIEKSKIFIIVLSENYASSSFCLNELTHILNFT‑KGKNLL‑VLPVFYIVDPSDVRHHRGSFGEALANH‑EKKLNSD‑‑NME‑‑NLETWKMALHQVSN‑ISGH‑‑‑HF‑QH‑G‑NKYEYKFIKEIV

>K7KB13

GEDTRLDFTDHLYAALVRKGIIA‑FRDDKQLE‑KGDAIAEELPKAIEESLGAIVILSENYASSSWCLDELNKILESN‑RVLGE‑‑VFPVFYGVSPGEVQHQKTQFYEAFKKH‑ERRSGKD‑‑‑TE‑‑KVQKWRDSLKELGQ‑IPGW‑‑‑ES‑‑‑‑‑KHYHQTELIENIV

>K7KBB2

GEDTRHKFIGHLRKELCQKGIKV‑FSDDKDL‑‑RGEGISPALSSAIEKSKILIVVFSENYAESTWCLDELVKILECKIRDKKQL‑VFPIFYHVDPSDIRHQKKSYGEHMLEH‑QKRFGKS‑‑‑‑Q‑‑RVQAWRSALSEASN‑FPGH‑‑‑H‑IS‑‑‑‑T‑YETEFIEKIA

>K7MIT8

GEDTRSAFTGHLYNTLQSKGIHT‑FIDDEKLQ‑RGEQITPALMKAIEDSRVAITVLSEHYASSSFCLDELATILHCD‑QRKRLL‑VIPVFYKVDPSDVRHQKGSYGEALAKL‑ERRF‑‑‑QHDPE‑‑KLQNWKMALQRVAD‑LSGY‑‑‑HF‑KE‑G‑EGYEYKFIEKIV

>K7KD09

GDDTRASFTSHLYTALHNAGISV‑FKDDETLP‑RGNKISTSLGLAIEESRLYVVVFSKNYAGSLWCLQELEKIMECH‑KATGQV‑VVPVFYDVDPSEVRHQTGHFGQAFRNL‑EEEWRVS‑‑GISGGEVIHWKEALHEAAG‑ISGI‑‑‑VV‑‑‑‑‑LNSNESEAIKTIV

>K7LWN3

GEDTRRNFVCHLHSVLSNAGVNT‑FLDDENLV‑KGMEL‑IQLMRAIEGSQISLVVFSKNYTQSTWCLTELENIIKCH‑RLHGHV‑VVPIFYHVSPSDVRRQEGDFGKALNAS‑EKIYS‑‑‑‑EDK‑‑YLSRWGSALTTAAN‑FCGW‑‑‑DV‑M‑‑‑‑KGNEAKLVKEIV

>K7L9W3

GEDTRRSFTSHLYESLNEVKVQT‑YIDD‑RLE‑KGEEISPTLTKAIENSRVSIVIFSENYASSKWCLGELIKIMESK‑KEKGQI‑VIPVFYNIDPSHVRKQTGSYEQAFEKH‑EGEP‑‑‑‑‑‑‑‑‑‑RCNKWKTALTEAAG‑LAGF‑‑‑DS‑RN‑‑‑‑‑TDPELLKDIV

>I1MM77

GEDTRGKFVSHLHYALSKAGVNT‑FIDDENLL‑KGMTLKDELMRAIEGSQISLVVFSKSYTESTWCLDELEKILECR‑KLHDQI‑VMPIFYDIEPSVVRHQKGAFGKALKSA‑EKTYSGE‑‑HAE‑‑QLWRWSSALNRAAD‑LSGF‑‑‑HV‑V‑‑‑‑DRNEAILVKEIV

>I1KEB3

GEDTRNSFTGFLFEALKKQGIEA‑FKDDKDIR‑KGESIAPELIRAIEGSHVFLVVFSKDYASSTWCLRELAHIWDC‑IQKSPR‑‑LLPIFYDVDPSQVRKQSGDYEKAFAQH‑QQRFED‑‑‑‑‑K‑‑EIKTWREVLNDVGN‑LSGW‑‑‑DI‑K‑‑‑‑NKQQHAVIEEIV

>K7LWN7

GIDTRHSFTDNLYNSLKQRGIHA‑FIDDEGLR‑RGEEITPTLLKAIRESRIGIIVFSKSYASSTYCLDELVEILECL‑KVEGRL‑VWPVFYDVDPSQVRYQTGTYAEALAKH‑KERFQDD‑‑K‑G‑‑KVQKWRKALHEAAN‑LSGW‑‑‑HF‑QH‑G‑SESEYKFIKKIV

>K7N1C8

GEDTRHTFTCKLYDALWLKGIDT‑FMDNKELK‑NGDKIGPTLHKAIEEARISVVVLSENYADSSWCLDELVKIHECM‑ESKNQL‑VWPIFYKVNPSDVRHQKGSYGVAMTKH‑ETSP‑‑‑‑‑DLE‑‑KVHKWRSTLNEIAN‑LKGK‑‑‑YL‑EE‑G‑‑‑‑ESKFIDDLA

>K7MIX4

GLDTRNGFTGNLYKALGDRGIYT‑FIDDQELP‑RGDKITPALSNAINESRIAITVLSENYAFSSFCLDELVTILHCK‑S‑EGLL‑VIPVFYKVDPSDVRHQKGSYGETMTKH‑QKRF‑‑‑ESKME‑‑KLREWRMALQQVAD‑LSGY‑‑‑HF‑KD‑G‑DSYEYKFIGNIV

>K7MIX2

GEDTRYGFTGNLYKALCDKGIHT‑FFDEDKLH‑SGEEITPALLKAIQDSRIAITVLSEDFASSSFCLDELATILFCA‑QYNGMM‑VIPVFYKVYPCDVRHQKGTYGEALAKH‑KKRF‑‑‑P‑‑‑D‑‑KLQKWERALRQVAN‑LSGL‑‑‑HF‑KD‑R‑DEYEYKFIGRIV

>I1MQE7

GEDTRHAFTGHLYKALHDKGIHT‑FIDDEKLQ‑RGEQITRALMEAIQDSRVAITVLSQNYASSSFCLDELATILHCH‑QRKRLL‑VIPVFYKVDPSDVRHQKGSYAEALEKL‑ETRF‑‑‑QHDPE‑‑KLQKWKMALKQVAD‑LSGY‑‑‑HF‑KE‑G‑DGYEFKFIEKIV

>K7KLL5

GSDTRQGFAANLYKALANRGIYT‑SIDDEELQ‑SGEEITPTLLKAIEESRISMAVLSVNYASSSFCLDELATIFDCA‑ERKALL‑VF‑‑‑YKVEPSHVRHRKVSYGEALAKK‑EERF‑‑‑KHNMD‑‑KLPKWKMPFYQAAN‑LSGY‑‑‑HF‑KD‑G‑YAHEYEFIGRMV

>K7KDG9

GEDTRASFTSHLYTALLNAGIIV‑FKDDESLL‑RGDQIAPSLRLAIEQSRISVVVFSRNYAESRWCLDELEKIMECH‑RTIGQV‑VVPVFYDVDPSEVRHQTGEFGRTFEKL‑KQEWKE‑‑‑‑‑‑‑‑‑VQSWKEALREAAG‑ISGV‑‑‑VV‑‑‑‑‑LNSNESEAIKSIV

>K7MIY4

REDTHRGFTFYLYKALNDRGIYT‑FFYDQELP‑RETEVTPGLYKAILASRVAIIVLSENYAFSSFCLDELVTILHCE‑R‑E‑‑‑‑VIPVFHNVDPSDVRHQKGSYGEAMAKH‑QKRF‑‑‑KAK‑‑‑‑KLQKWRMALKQVAN‑LCGY‑‑‑HF‑KD‑G‑GSYEYMLIGRIV

>K7MIV1

GSDTRYGFTGNLYNALSDRGIHT‑FIDEEELQ‑RGDEIRPALVEAIKQSRMAILVFSKNYASSSFCLDELVKIMECV‑KAKGRL‑IFPIFYDVDPCHVRHQSGSYGEALAMH‑EERFTSSKENME‑‑RLQKWKMALNQAAD‑VSGK‑‑‑HY‑KL‑G‑NEYEHEFIGKIV

>K7KXS1

GIGTRNSFTDHLFAALQGITKIS‑MKENSNMS‑SSKQLKDPI‑‑‑‑‑‑‑‑‑‑‑‑‑‑‑‑DYASSTWCMKELTKIVDW‑VQETGP‑‑VLPVFYDVTPSEVRKQSGQFGEAFTEH‑EER‑‑‑‑‑‑‑‑‑‑‑‑‑‑‑‑‑‑‑‑‑‑‑‑‑‑‑‑‑‑‑‑‑‑‑‑‑‑‑‑‑‑‑PQNEEIEKIV

>K7KCW5

GEDIRHGFLGYLTEAFHQKQIHA‑FIDD‑KLE‑KGDEIWPSLVGAIQGSLISLTIFSENYSSSRWCLEELVKIIECR‑ETYGQT‑VIPVFYHVNPTDVRHQKGSYEKALSEH‑EKKYNLT‑‑‑‑‑‑‑TVQNWRHALKKAAD‑LSGI‑‑‑KS‑‑‑‑‑‑‑‑TEVELLGEIV

>K7LX77

GEDTRKNFTSHLYEALKQKKIET‑YIDY‑RLE‑KGDEISAALIKAIEDSHVSVVIFSENYASSKWCLGELGKIMECK‑KERGQI‑VIPVFYNIDPSHVRKQTGSYEQSFAKH‑TGEP‑‑‑‑‑‑‑‑‑‑RCSKWKAALTEAAN‑LAAW‑‑‑DS‑QI‑‑‑‑‑TESEFLKDIV

>C6THX9

TEDTGKTFTSHLSGALERVDIKT‑YVDNNNLE‑RGEEIPTTLVRAIEEAKLSIIVFSKNYAASKWCLDELLKILECG‑RAKRQI‑IVPVFYDIDPSDVRSQRGTYAEAFAKH‑ERNFNEK‑‑‑K‑‑‑KVLEWKNGLVEAAN‑YAGW‑‑‑DC‑K‑‑‑VNRTEFEIVEEIV

>K7KCV0

GSDIRLGFLSHLSKAFHQKQIHA‑FVDD‑KLQ‑RGDEISQSLLEAIEGSSISLIIFSEDYASSRWCLEELVKIVECR‑EEYGQI‑VIPVFYNVDPTNVRHQKGSFETALAEH‑EKKYDLP‑‑‑‑‑‑‑IVRMWRRALKNSAN‑LAGI‑‑‑NS‑‑‑‑‑‑‑‑NDAELLEDII

>K7MWR5

GEDTRHSFTGNLYKALSDRGIHT‑FIDDKKLP‑RGDQISSALEKAIEESRIFIIVLSENYASSSFCLNELGYILKFI‑KGKGLL‑VLPVFYKVDPSDVRNHAGSFGESLAHH‑EKKFNADKENLV‑‑KLETWKMALHQVAN‑LSGY‑‑‑HF‑KH‑G‑EEYEYKFIQRIV

>K7K5P7

GTDIRSGVLSHLIAALSNAGVNT‑F‑EDEKFE‑RGERIMPSLLRAIAGSKIHIILFSNNYASSKWCLDELVKIMECH‑RTYGNE‑VLPVFYNVDPSDVRNQRGDFGQGLEAL‑QR‑YLLQ‑‑GEN‑‑DLKSWKSALNEAAN‑LAGW‑‑‑VS‑R‑‑‑‑NRTDADLVEDIV

>K7N1C6

GEDTRHTFTCKLYDALWLKGIDT‑FMDNKELK‑NGDKIGPTLHKAIEEARISVVVLSENYADSSWCLDELVKIHECM‑ESKNQL‑VWPIFYKVNPSDVRHQKGSYGVAMTKH‑ETSP‑‑‑‑‑DLE‑‑KVHKWRSTLNEIAN‑LKGK‑‑‑YL‑EE‑G‑‑‑‑ESKFIDDLA

>I1KE93

GEDTRNSFTAFLFEALKKQGIEA‑FKDDKDIR‑KGESIAPELIRAIEGSHVFLVVFSKDYASSTWCLRELAHIWNC‑IQTSPR‑‑LLPIFYDVDPSQVRKQSGDYEKAFAQH‑QQRFQD‑‑‑‑‑K‑‑EIKTWREVLNHVAS‑LSGW‑‑‑DI‑R‑‑‑‑NKQQHAVIEEIV

>I1N6X6

GEDTRHSFTGNLYKALSDRGIHT‑FIDDKKLP‑RGDQISSALEKAIEESRIFIIVLSENYASSSFCLNELGYILKFI‑KGKGLL‑VLPVFYKVDPSDVRNHAGSFGESLAHH‑EKKFNADKENLV‑‑KLETWKMALHQVAN‑LSGY‑‑‑HF‑KH‑G‑EEYEYKFIQRIV

>I1MD01

GTDVRCGFLSHLKKELRQKQVDA‑FVDD‑RLE‑GGDEISHSLDKAIEGSLISLVIFSKDYASSKWCLEEVVKIIECM‑HSNKQI‑VIPVFYNVDPSDVRHQKGTYGDAFAKH‑EKKRNLA‑‑‑‑‑‑‑KVPNWRCALNIAAN‑LSGF‑‑‑HS‑‑‑‑‑‑‑‑DEVELIEEIA

>K7MH86

GEDTRYGFTGNLYNVLRERGIHT‑FIDDQELQ‑KGDQITKALEEAIEKSKIFIIVLSENYASSSFCLNELTHILNFT‑KGKNVL‑VLPVFYKVDPSDVRKHRGSFGEALANH‑EKKLNSN‑‑NME‑‑KLETWKMALHQVSN‑ISGH‑‑‑HF‑QH‑G‑DKYEYKFIKEIV

>I1MND5

REDTRHGFTGNLYNVLRERGIHT‑FIDDDEPQ‑KADQITKALEEAIKNSKIFIIVLSENYASSFFCLNELTHILNFT‑KGWDVL‑VLPVFYKVDPSDVRHHRGSFGEALANH‑EKNLNSN‑‑YMG‑‑KLKTWKMALRQVSN‑FSGH‑‑‑HF‑QP‑G‑NKYEYKFIKEIL

>K7MEG0

GADIRQGFLSHLIEAFSRKHIAA‑FVDH‑NIL‑KGDELSETLLGAINGSLISLIIFSQNYASSRWCLLELVKIVECR‑KRDGQI‑VVPVFYKVDPSDVRHQKGTYGDAFAKH‑EGKFSLT‑‑‑‑‑‑‑TIQTWRSALNESAN‑LSGF‑‑‑HS‑‑‑‑‑‑‑‑DEAELVKEIV

>K7KD11

GEDTRASFTSHLYTALHNEGVFV‑FKNDETLP‑RGNQISPSLRLAIEESRISVVVFSTNYAESRWCLKMLENIMECQ‑RTTGQV‑VVPVFYGVYPSKVRHQTGDFGKAFRNL‑‑‑‑WREA‑‑‑‑‑‑‑‑‑‑‑‑‑‑‑‑‑‑‑‑‑‑‑‑‑‑‑‑‑‑‑‑‑‑‑‑‑‑‑NESEAIQTIV

>K7KXC3

GEDTRNSFTGFLLQALKKEGIEA‑FKDDKDIR‑KGESIAPELIRAIEGSHVFLVVFSKDYASSTWCLRELAHIRNC‑IQTSPR‑‑LLPIFYDVDPSQVRKQSGDYQKAFAQH‑QQRFQE‑‑‑‑‑K‑‑EINIWREVLELVAN‑LSGW‑‑‑DI‑R‑‑‑‑YKQQHAVIEEIV

>K7K3H2

GEDIRHSFLGYLTEAFYQKQINA‑FVDD‑KLE‑KGDEIWPSLVGAIQGSSISLTIFSENYTSSRWCLDELVKILECR‑EKYGQI‑VIPVFYGVNPTDVRHQKGSYGEALAQL‑GKKYNLT‑‑‑‑‑‑‑TVQNWRNALKKVAD‑LSGI‑‑‑KS‑‑‑‑‑‑‑‑TEVELLGEIV

>K7MRG1

GEDTRRNFTSHLYEALKQKKVET‑YIDE‑HLE‑KGDEISPALIKAIEDSHVSIVVFSKNYASSKWCLVELIKILDCK‑KDRGQI‑VIPVFYEIDPSDVRKQTGSYEQAFAKH‑EGEP‑‑‑‑‑‑‑‑‑‑SCNKWKTALTEAAN‑LAGW‑‑‑DS‑RT‑‑‑‑‑TDPELLKDIV

>K7N0U7

GEDTRNNFTSHLYAAFQLNKIQA‑FIDNR‑LH‑KGDEISPSIFKAIKHCNLSVVVLSKHYASSTWCLRELAEILD‑H‑KKRGHI‑VIPVFYKIDPSHVRKQTGTYGKAFEKY‑ERDV‑‑‑KHNMA‑‑MLQKWKAALTEVAN‑LVGW‑‑‑EF‑K‑‑N‑HRTENELIEGIV

>I1LC91

GEDVRTSFISHLRSALSRDNIKA‑YMDDHNLQ‑KGDELWPSLCQAIQDSELAIVVFSEHYAASKWCLNELVEILHCR‑KSQGLA‑VIPVFYEVDPSHIRKYDGTCGEAISKY‑ETK‑‑‑D‑‑NES‑‑‑IQKWKAALAEAAH‑ISGW‑‑‑DS‑H‑‑‑REKNDSQLIEKIV

>K7KDV8

GEDLRKNFISHLSYALSKAGINT‑VLDGQQ‑‑‑‑‑MEL‑EELMKP‑EKSQISIVVFSKSYTESTWCLDELAKIIEIH‑ETYGQR‑VVVVFYEIDPSHVRDQKGDFGKGLKAA‑RKRFSEE‑‑HLE‑‑SLSRWSQALTKAAN‑FSGL‑‑‑DL‑K‑‑‑‑NRDEAELVKQIV

>K7KDW5

GEDIRKNFVSHLHSALLHAEVKT‑FLDDENLL‑KGMKS‑EELIRAIEGSQIAVVVFSKTYTESSLCLRELEKIIESH‑ETRGQR‑VLPIFYEVDPSDVRQQKGDFGEALKAA‑QKGFSGE‑‑HLE‑‑SLSRWSQAITKAAN‑LPGW‑‑‑DE‑S‑‑‑‑NENDAELVEGII

>K7LQJ9

GEDTRFGFTGHLYNTLRHRGINT‑FMDDEALE‑RGEQISEAIFKAIEESGKAIVVFSKNYASSTWCLEELVKILSCK‑KE‑‑‑LKVYPLFYNVDPSEVRYQRASYGQQLAKH‑E‑KMKY‑‑‑SKQ‑‑KVQNWRLALHEAAN‑LVGW‑‑‑HF‑K‑‑‑‑DGYEYEFITRIV

>K7KDV7

GEDTRRSFVCHLNCALSKAGVKT‑FLDEENLH‑KGMKL‑DELMTAIEGSQIAIVVFSKSYTESTWCLRELEKVIECN‑ETYGQS‑VLPVFYNIDPSVVRHEKHDFGKVLKST‑EKNYSGE‑‑HLE‑‑NLSRWSRALSEASK‑FSGW‑‑‑DA‑S‑‑‑‑KRNDAELVEKIV

>K7K1I4

GEDTRKIITSHLYHALFQAELAT‑YIDY‑RLQ‑KGDEISQALIEAIEESQVSVIIFSEKYATSKWCLDEITKIIECK‑EGQGQV‑VIPVFYKIDPSHIRKQQGSFKQAFVEH‑EQDLKIT‑‑‑TD‑‑RVQKWREALTKAAN‑LAGW‑‑‑DF‑QT‑‑‑‑‑TEAEFIKDIV

>K7LEA6

GSDTRHGFTGHLHKALHDSGIHA‑FIDDHDLM‑RGEEITPALKEAIEKSNVAITMLSEDYASSSFCLYELDYILECR‑RKRKLL‑VLPVFYKVSPSHVEHQTGCYGEALAK‑‑‑‑‑‑‑‑‑‑‑‑‑‑‑‑LNEKFQPKMDDCCI‑KTGY‑‑‑EH‑K‑‑‑‑‑‑‑‑‑‑FIGEIV

>K7LUI7

GKDTRNNFTDHLFGALQRKGILT‑FRDDTKLK‑KGERILSSLMQAIEGSQIFVIVFSKNYASSTWCLRELEKILDC‑VIVPGK‑‑VLPIFYDVDPSEVRKQTGDYGKAFTKH‑EERFKDDVEKME‑‑EVKRWRRALTQVAN‑FSGW‑‑‑DM‑M‑‑‑‑NKSQYDEIEKIV

>K7KQN0

ATDTRSNFTDFLFQALIRKGIVA‑FKD‑‑‑‑ESR‑‑‑‑AP‑‑‑QAIEDSRLFIVVLSKNYAFSTQCLHELSQIFHC‑VEF‑SR‑‑VLPIFYDVDPSDVRKQTG‑Y‑KAFSKY‑EERFLVNKKGMET‑‑VQTWRKALTQVAN‑LSGW‑‑‑‑YIR‑‑‑‑NKTEIEFVYTI‑

>K7MDD0

GWDIRFSFTGFLYKGLFDHG‑RT‑FMDDREID‑KGSQIP‑TLREAIEDSRVFIVVLSANFASSSFCLDEVVLILQEA‑KGKGR‑‑ILPVFYYVDPSHLAD‑SDSYKRALEDQ‑TEW‑‑‑‑‑‑‑DQ‑‑RIQIWKTALSKLAT‑FSGL‑‑‑RLIR‑‑‑‑NSLEYQYIELIL

>K7KDW3

GEDTRKKFVCHIYKALSNAGINT‑FIDEENIQ‑KGMTL‑DELMTAIEGSQIAIVVFSKTYTESTWCLRELQKIIECH‑ENYGQR‑VVPVFYHIDPSHIRHQEGDFGSALNAV‑ERRHSGE‑‑DLK‑‑SLSNWKRVLKKATD‑FSGW‑‑‑NE‑R‑‑‑‑DRNDAELVKEIV

>K7KCZ9

GEDTRASFTSHLYTALHNAGVFV‑FKDDETLS‑RGNKISPSLQLAIEESRVSVVVFSRNYAESRWCLKELEKIMECH‑RTTGQV‑VVPVFYDVDPSEVRHQTGHFGKAFRNL‑EEEWREAGASISGGKMMSWKEALREAAG‑ISGI‑‑‑VV‑‑‑‑‑LNSNESEAIKTIV

>K7KXJ1

GEDTRNNFTAFLFDALSQNCINA‑FKDDADLK‑KGESIAPELLQAIEGSRLFVVVFSKNYASSTWCLRELAHICNCTIEASPG‑‑VLPIFYDVDPSEVRKQSAYYGIAFEEH‑EGRFREDKEKME‑‑EVLRWREALTQVAN‑LSGW‑‑‑DI‑R‑‑‑‑NKSQPAMIKEIV

>K7M4Z6

TEGTHLDFANTLCTSLQRNGIST‑FRYDKQKE‑RGYLILEKLHKVIEQCLVVIVLLSENYASSTWCLDELHKILESK‑RVLGP‑‑VFPLFYDVVPSDVRHQKNKFAEAFEEH‑ATRPEED‑‑‑KV‑‑KVQKWRESLHEVAG‑FSGW‑‑‑ES‑‑‑‑‑KNWKE‑ELIEEII

>Q84ZV0

GEDTRYGFTGNLYRALCEKGIHT‑FFDEEKLH‑GGDEITPALSKAIQESRIAITVLSQNYAFSSFCLDELVTILHCK‑S‑EGLL‑VIPVFYNVDPSDLRHQKGSYGEAMIKH‑QKRF‑‑‑ESKME‑‑KLQKWRMALKQVAD‑LSGH‑‑‑HF‑KD‑G‑DAYEYKFIGSIV

>K7KCX5

GDDIRRDFLGHLTKEFRRKQIHA‑FVDD‑KLK‑TGDELWPSFVEAIQGSLISLTILSENYASSSWSLNELVTILECR‑EKYNRI‑VIPVFYKVYPTDVRHQNGSYKSDFAEH‑EKKYNLA‑‑‑‑‑‑‑TVQNWRHALSKAAN‑LSGI‑‑‑KS‑‑‑‑‑‑‑‑TEVELLEKIV

>K7KD04

GEDTRASFTSHLYTALRNAGIFV‑FKDDETLP‑RGNKISPSLQLAIEESRVSVVVFSRNYAESRWCLKELENIMECH‑RTTGQV‑VVPVFYDVDPSEVRHQTGHFGKAFRNL‑EEEWREAGASILGGKMMSWKEALCEAAG‑ISGI‑‑‑VV‑‑‑‑‑LNSNESEAIKTIV

>K7K612

SEDTRKTFTSHLNGALERVDIKT‑YVDNNNLE‑RGEEIPITLVRAIEEAKLSVIVFSKNYADSKWCLDELLKILECG‑RTKRHI‑IVPVFYDIDPSDVRNQRGSYAEAFVNH‑ERNFDE‑‑‑‑K‑‑‑KVLEWRNGLVEAAN‑YAGW‑‑‑DCNK‑‑‑VTRTEYEIVEDFT

>K7MHF0

GEDTRYGFTGNLYKALYDKGIHT‑FIDE‑ELQ‑RGDKITSTLEKAI‑‑‑‑‑‑‑‑‑‑‑QDYASSPFCLNELAYILNFI‑KGNRQL‑VLPVFHNVDTSHVRHHTGSF‑‑‑‑‑‑‑‑EQKN‑‑‑‑‑NVE‑‑KLDTWKMALHQAAS‑LSGY‑‑‑HF‑KH‑G‑DGYEYQFINRIV

>I1MNC7

GE‑‑‑‑‑FTPSLMTTSSRKGTKS‑‑‑‑‑‑‑‑‑‑‑‑‑‑ITTALEEAIEKSKIFIIVLSENYASSSFCLNELTHILNFT‑KEKNVL‑VLPVFHKVNPSDVRHHRGSFGEALANH‑EKKLNSN‑‑NTE‑‑KLQTWKMALHQVSN‑ISGY‑‑‑HF‑QD‑G‑NKYEYKFIKEIV

>C6ZS34

GEDTRYTFTGHLHASLTRLQVNT‑YID‑YNLQ‑RGEEISSSLLRAIEEAKLSVVVFSKNYGNSKWCLDELLKILECK‑NMRGQI‑VLPIFYDIDPSHVRNQTGTYAEAFAKH‑EKHLQGQ‑‑‑MD‑‑KVQKWRVALREAAN‑LSGW‑‑‑DC‑S‑‑‑VNRMESELIEKIA

>K7KZI3

GADIRQDFLSHLVEGFYRRQIHA‑FVDY‑KIL‑KGDQLSEALLDAIEGSLISLIIFSENYASSHWCLFELVKIVECR‑KKDGQI‑LLPIFYKVDPSNVRYQKGTYGDAFAKH‑EVRHNLT‑‑‑‑‑‑‑TMQTWRSALNESAN‑LSGF‑‑‑HS‑‑‑‑‑‑‑‑DEAELVKEIV

>K7LUI6

GLDTRNSFTDHLFAALQRKGIVA‑FRDNQNIN‑KGELLEPELLQAIEGSHVFIVVFSKDYASSTWCLKELRKIFDR‑VEETGR‑‑VLPIFYDVTPSEVRKQSGKFGKAFAEY‑EERFK‑‑‑DDLE‑‑MVNKWRKALKAIGN‑RSGW‑‑‑DV‑Q‑‑‑‑NKPEHEEIEKIV

>K7MH83

GEDTRYGFTGNLYKVLQERGIHT‑FIDDEELQ‑EGDQITTALEEAIEKSKIFIIVLSENYASSSFCLNELTHILNFT‑KENNVL‑VLPVFYKVDPSDVRHHRGSFGEALANH‑EKNLNSN‑‑NME‑‑KLQIWKKALHQVSN‑ISGY‑‑‑HF‑QD‑G‑NKYEYKFIKEIV

>K7M6W8

GEDTRADFTSHLHAALRRNNIDT‑YID‑YRIH‑KGDEIWVEIMKAIKESTLFLVIFSENYASSSWCLNELIQLMEYK‑KH‑‑DVDVIPVFYKIDPSEVRKQSGSYHMAFAKH‑EKDRKVT‑‑‑‑‑‑‑EMQKWKNALYEAAN‑LSGF‑‑‑LS‑‑‑‑‑‑‑‑TESNMIEDII

>I1MQH7

GQDTRQGFTGYLYKALCDRGIYT‑FIDDQELR‑RGDEIKPALSNAIQESRIAITVLSQNYASSSFCLDELVTILHCK‑S‑QGLL‑VIPVFYKVDPSHVRHQKGSYGEAMAKH‑QKRF‑‑‑KANKE‑‑KLQKWRMALHQVAD‑LSGY‑‑‑HF‑KD‑G‑DSYEYEFIGSIV

>K7K361

GEDTRSSFTSHLYAALQNAGIIV‑FKDDESLP‑RGHHISDSLLLAIEQSQISVVVFSRNYADSRWCLKELERIMECH‑RTIGHV‑VVPVFYDVDPSEVRHQTSHFGNAFQNL‑SGEETN‑‑‑‑‑‑‑‑‑‑KSWREALREAAS‑ISGV‑‑‑VV‑‑‑‑‑LDSNESEAIKNIV

>K7MWS0

GKDTRHSFTGNLYKALSERGINT‑FIDDKKLP‑RGDEITSALEKAIEESRIFIIVLSENYAWSSFCLNELDYILKFI‑KGKGLL‑VLPVFYKVDPSDVRNHTGSFGESLAYH‑EKKFKSTN‑NME‑‑KLETWKMALNQVAN‑LSGY‑‑HHF‑KH‑G‑EEYEYQFIQRIV

>I1KEV5

GKDTRNNFTDHLFGAFHRKKIRT‑FRDDTRLK‑KGERILSNLMQAIEGSQIFVIVFSKNYAFSSWCLKELAKILDC‑VRVSGK‑‑VLPIFYDVDPSEVRNQTGDYEKAFAKH‑EDR‑‑‑‑‑EKME‑‑EVKRWREALTQVAN‑LAGW‑‑‑DM‑R‑‑‑‑NKSQYAEIEKIV

>K7MIT9

GEDTRSAFTGHLYNTLQSKGIHT‑FIDDEKLQ‑RGEQITPALMKAIEDSRVAITVLSEHYASSSFCLDELATILHCD‑QRKRLL‑VIPVFYKVDPSDVRHQKGSYGEALAKL‑ERRF‑‑‑QHDPE‑‑KLQNWKMALQRVAD‑LSGY‑‑‑HF‑KE‑G‑EGYEYKFIEKIV

>K7KXC2

GEDTRNSFTGFLLQALKKEGIEA‑FKDDKDIR‑KGESIAPELIRAIEGSHVFLVVFSKDYASSTWCLRELAHIRNC‑IQTSPR‑‑LLPIFYDVDPSQVRKQSGDYQKAFAQH‑QQRFQE‑‑‑‑‑K‑‑EINIWREVLELVAN‑LSGW‑‑‑DI‑R‑‑‑‑YKQQHAVIEEIV

>K7MWR8

GEDTRRGFTGNLYKALSDRGIHT‑FMDDKKIP‑RGDQITSGLEKAIEESRIFIIVLSENYASSSFCLNELDYILKFI‑KGKGIL‑ILPVFYKVDPSDVRNHTGSFGKALTNH‑EKKFKSTN‑DME‑‑KLETWKMALNKVAN‑LSGY‑‑HHF‑KH‑G‑EEYEYEFIQRIV

>I1MM78

GEDTRKTFVSHLYAALSNAGINT‑FID‑HKLR‑KGTELGEELLAVIKGSRISIVVFSANYASSTWCLHELVEIIYHR‑RAYGQV‑VVPVFYDVDPSDVRHQTGAFGQRLKAL‑QK‑‑‑SK‑‑PID‑‑FFTSWKSALKEASD‑LVGW‑‑‑DA‑R‑‑‑‑NRSEGDLVKQIV

>I1M0S0

AEDTHQGFVGHLFKSLTDLGVVV‑SGDHRDLK‑EE‑‑‑‑‑‑‑‑‑EIECFRVFIIVFSHHYATSSSRLDKLTEIINKG‑AAEDR‑‑IFPFFFEVEPNHVRFQSGSFEIAFDSH‑ANR‑‑‑‑‑‑‑EE‑‑CLQRWKITLKKVTD‑FSGW‑‑‑SFNR‑‑‑‑SKYQYQVIEKIV

>I1KE91

GEDTRNSFTAFLFEALKKQGIEA‑FKDDKDIR‑KGESIAPELIRAIEGSHVFVVVFSKDYASSTWCLRELAHIWNC‑IQTSRR‑‑LLPIFYDVDPSQVRKQSGDYQKAFSQH‑QQKFQE‑‑‑‑‑K‑‑EITTWRKVLEQVAG‑LCGW‑‑‑DI‑R‑‑‑‑NKQQHAVIEEIV

>K7LSQ4

RDDTHHTFTCKLYDSLCRKGIIT‑FMDNEELK‑VGDQIGHKLLKAIEESRISIVVLSENYAASSWCLDELVKIHECM‑KAKNLL‑VWPIFYKVDPSDVRHQNGSYGEAMTEH‑ETRF‑‑‑‑‑DSE‑‑KVHKWRLTLTDMTN‑LKGE‑‑‑HV‑QE‑G‑‑‑‑ESKFIDDLV

>K7MHM2

GSDTRHGFTGHLYKALCDRGIHT‑FIDDEELQ‑RGEEITPLLVKAIEGSRIAIPVFSKNYASSTFCLDELVHILACV‑KEKGTL‑VLPVFYEVDPSDVRHQRGSYKDALNSH‑KERFNDDQE‑‑‑‑‑KLQKWRNSLSQAAN‑LAGY‑‑‑HF‑KH‑GENEYEYDFIGNIV

>K7MWR4

GEDTRHSFTGNLYKALSDRGIHT‑FIDDKKLP‑RGDQISSALEKAIEESRIFIIVLSENYASSSFCLNELGYILKFI‑KGKGLL‑VLPVFYKVDPSDVRNHAGSFGESLAHH‑EKKFNADKENLV‑‑KLETWKMALHQVAN‑LSGY‑‑‑HF‑KH‑G‑EEYEYKFIQRIV

>I1LSM9

GEDTRNNITSFLLGSLESKGIDV‑FKDNEDLR‑KGESIAPELLQAIEVSRIFVVVFSKNYASSTWCLRELTHICNCT‑QTSPG‑‑VLPIFYDVDPSDVRKLSGSYEEAFAKY‑KERFREDREKMK‑‑EVQTWREALKEVGE‑LGGW‑‑‑DI‑R‑‑‑‑DKSQNAEIEKIV

>I1JC09

GEDTRGNFTSHLYDALIQAKLET‑YIDY‑RLQ‑KGEEISQALIEAIEESQVSVVIFSEKYGTSKWCLDEITKIMECK‑EGQGQV‑VIPVFYKIDPSHIRKQQGSFNKAFEEH‑KRDPNIT‑‑‑ND‑‑RVQKWRSALTKAAN‑LAGW‑‑‑DS‑IT‑‑‑‑‑TEAKFIKDIV

>K7LK72

GEDIRTTFIGHLRSALSGPNIKA‑YADDHDLQ‑KGQEIWPSLCQAIQDSHFAIVVFSENYAESKWCLKELVQILHCR‑KTQGLV‑VIPVFYQVDPSHIRKCTGTYGEAIAKH‑‑‑K‑‑‑D‑‑NQS‑‑‑VQDWKAALTEAAN‑ISGW‑‑‑DT‑‑‑‑‑‑‑RNESQLIEKIV

>I1KE95

GEDTRNSFTAFLFEALKKQGIEA‑FKDDKDIR‑KGESIAPELIRAIEGSHVFLVVFSKDYASSTWCLRELAHIWNC‑FQPSTR‑‑LLPIFYDVDPSQVRKLSGDYEKAFAQH‑QQRFQE‑‑‑‑‑K‑‑EITTWREVLERVAS‑LSGW‑‑‑DI‑R‑‑‑‑NKEQPTVIDEIV

>K7MG09

GGDTRRNFVSHLYYALSNAGVNT‑FFDEENLL‑KGMQL‑EELSRAIEGSQIAIVVFSETYTESSWCLSELEKIVECH‑ETYGQT‑IVPIFYDVDPSVVRHPTGHFGDALEAA‑QKKYSAK‑‑DRE‑‑YFSRWKIALAKAAN‑FSGW‑‑‑DV‑K‑‑‑‑NRNKAKLVKKIV

>K7MBH2

GKDVRGNFLSHLDEIFKRNKIYA‑FVDD‑KLK‑KGDEIWSSLVEAIEQSFILLIIFSQSYASSRWCLKELEAILECN‑KKYGRI‑VIPVFYHVEPADVRHQRGSYKNAFKKH‑EKRNK‑T‑‑‑‑‑‑‑KVQIWRHALKKSAN‑IVGI‑‑‑ET‑‑‑‑‑‑‑‑NEVELLQEIV

>I1MQI3

GGDTRYGFTGNLYRALCDKGIHT‑FFDEKKLH‑RGEEITPALLKAIQESRIAITVLSKNYASSSFCLDELVTILHCK‑S‑EGLL‑VIPVFYNVDPSDVRHQKGSYGVEMAKH‑QKRF‑‑‑KAKKE‑‑KLQKWRIALKQVAD‑LCGY‑‑‑HF‑KD‑G‑DAYEYKFIQSIV

>K7MIY7

GTDTRYGFTGNLYKALCDKGFHT‑FFDEDKLH‑SGEEITPALLKAIQDSRVAIIVLSENYAFSSFCLDELVTIFHCK‑R‑EGLL‑VIPVFYKVDPSYVRHQKGSYGEAMTKH‑QERF‑‑‑KDKME‑‑KLQEWRMALKQVAD‑LSGS‑‑‑HF‑KD‑G‑GSYEYEFIGSIV

>K7KXG4

SEDTRNNFTGFLFQALSRKGIDA‑FKDDKDLK‑KGESIAPELLQAIEGSCLFVVVFSKNYASSTWCLRELAEICNC‑IETSQR‑‑VLPIFYDVDPSEVRKQSGYFEKAFAEH‑EKRFKEDKKKMQ‑‑EVQGWREALKQVSD‑QSLW‑‑‑‑‑‑‑‑‑‑‑‑‑PQCAEIEEIV

>K7KDW2

GEDTRKKFVCHIYKALSNAGINT‑FIDEENIQ‑KGMTL‑DELMTAIEGSQIAIVVFSKTYTESTWCLRELQKIIECH‑ENYGQR‑VVPVFYHIDPSHIRHQEGDFGSALNAV‑ERRHSGE‑‑DLK‑‑SLSNWKRVLKKATD‑FSGW‑‑‑NE‑R‑‑‑‑DRNDAELVKEIV

>K7MDC7

ADDTVAGFTSTLAKSLEDQG‑RV‑LVDHRDLK‑KA‑‑‑‑‑‑‑‑‑EIETVRVFIVVLSEHYAICPFRLDKLAEIVD‑‑‑‑GLGAQRVLPVFYYVPTSDVRYQTGSYEVALGVH‑EYY‑‑‑‑‑‑‑EE‑‑RLEKWKNTLEKVAG‑FGGW‑‑‑PLQR‑‑‑‑TKYEYQYIEEIG

>I1M0Q1

GEDTRRSFTGNLYNCLEKRGIHT‑FIGDYDFE‑SGEEIKASLSEAIEHSRVFVIVFSENYASSSWCLDGLVRILD‑F‑EDNHRP‑VIPVFFDVEPSHVRHQKGIYGEALAMH‑ERRL‑‑‑NPESY‑‑KVMKWRNALRQAAN‑LSGY‑‑‑AF‑KH‑G‑DGYEYKLIEKIV

>I1JM80

GEDTRKKFVCHIYKALSNAGINT‑FIDEENIQ‑KGMTL‑DELMTAIEGSQIAIVVFSKTYTESTWCLRELQKIIECH‑ENYGQR‑VVPVFYHIDPSHIRHQEGDFGSALNAV‑ERRHSGE‑‑DLK‑‑SLSNWKRVLKKATD‑FSGW‑‑‑NE‑R‑‑‑‑DRNDAELVKEIV

>K7MG06

GEDTRRNFVSHLYSALSNAGVNT‑FLDEMNYP‑KGEELNEGLLRTIEGCRICVVVFSTNYPASSWCLKELEKIIECH‑KTYGHI‑VLPIFYDVDPSDIRHQQGAFGKNLKA‑‑‑‑‑FQGL‑‑WGE‑‑SLSRWSTVLTQAAN‑FSGW‑‑‑DV‑S‑‑‑‑NRNEAQFVKEIV

>I1KEE0

GEDTRNNFTAFLFDALFENGIHA‑FKDDTHLQ‑KGESIAPELLLAIQGSRLFVVVFSKNYASSTWCLRELAHICNCTIEASPS‑‑VLPIFYDVDPSEVRKQSGYYGIAFAEH‑EERFREDKVKME‑‑EVQRWREALTQMAN‑LSGW‑‑‑DI‑R‑‑‑‑NKSQPAMIKEIV

>I1MMX8

GPDVRKGLLSHLKKELCRRQI‑‑‑‑‑EDEILD‑RGDEISSSLLRAIEESQILLVIFSKDYASSQWCLEELAKMIEC‑LERK‑QI‑LVPVFFNVDPSDVRQQHGEYGDALAKH‑EEKLKE‑‑‑NM‑‑‑KVQSWRSALKKAAN‑LSGF‑‑‑HYGN‑F‑‑D‑DESDLVDKIV

>I1KE99

GEDTRNSFTGFLFEALKKQGIEA‑FKDDKDIR‑KGESIAPELIRAIEGSHVFLVVFSKDYASSTWCLRELAHIWNC‑IRTSSR‑‑LLPIFYDVDPSQVRKQSGDYEKAFSQH‑QQRFQE‑‑‑‑‑K‑‑EIKTWREVLNHVGN‑LSGW‑‑‑DI‑R‑‑‑‑NKQQHAVIEEIV

>I1MQI7

GLDTRHGFTGNLYKALDDRGIYT‑SIDDQELP‑RGDEITPALSKAIQESRIAITVLSQNYASSSFCLDELVTILHCK‑S‑EGLL‑VIPVFYKVDPSDVRHQKGSYGEAMAKH‑QKRF‑‑‑KAKKE‑‑KLQKWRMALKQVAD‑LSGY‑‑‑HF‑ED‑G‑DAYEYKFIGSIV

>K7MIX8

GEDTRHGFTGNLYRALCDKGIHT‑FFDEVKLH‑SGDEITPALSNAIQESRIAITVLSQNYASSSFCLDELVTILHCK‑S‑EGLL‑VIPVFYKVDPSDVRHQKGSYREAMAKH‑QKGF‑‑‑KAKKE‑‑KLQKWRMALHQVAD‑LSGY‑‑‑HF‑KD‑G‑DAYEYKFIGSIV

>G7KJ68

GSDTRYGFTGNLYKALDDKGIHT‑FIDNHELQ‑RGDEITPSLLKAIEESRIFIAVFSINYASSSFCLDELVHIIHCY‑KTKGRL‑VLPVFFAVEPTIVRHQKGSYGEALAEH‑EKRFQNDPKSME‑‑RLQGWKEALSQAAN‑LSGY‑‑‑HD‑SP‑‑‑PGYEYKLIGKIV

>G7KJS0

GSDTRYGFTGNLYKDLCKKGIRT‑FIDDRELP‑GGDKITPSLFKAIEESRIFIPVLSINYASSSFCLDELVHIIHCC‑KKNGRL‑VLPIFYDVEPSNVRHQIGSYGKALAEH‑IEKFQNSTDNME‑‑RLQKWKSALTQTAN‑FSGH‑‑‑HF‑SS‑R‑NGYEYEFIEKIV

>G7KCQ4

GEDTRFGITDHLYDALIHKSIKT‑YI‑DYQLN‑RGEDVWPALSKAIEDSYISIIVFSENFATSKWCLEELVKVLECR‑KDHGQI‑VIPVFYKADPSHIRNQKASYETAFAKH‑ERK‑‑‑D‑‑SISN‑KVLKWKAALTEAAN‑ISGW‑‑‑DS‑H‑‑‑‑‑EKESILILKIV

>G7JVS5

G‑GTRYSFTDHLYHSLLRHGINV‑FRDDQNI‑‑NGDEIGTSLLKAIEASRISIVVLCRDYASSTWCLDELVKIVDCY‑DKNRKS‑VFVIFYKIE‑‑‑‑‑‑‑‑‑‑‑‑‑‑‑‑PS‑DVRFGKS‑‑‑‑E‑‑KVKAWRLALNRVCA‑LSGL‑‑‑H‑CK‑‑‑‑D‑YEYEFIEKIV

>G7ZUP2

GEDTRKTFTSHLNSALRRLDIKT‑YID‑DNLE‑RGDEISQALLKEIDEAKLSVIVFSKNYATSKWCLDEVVKILECR‑KYKEQI‑ILPDFYEVDPFHVRHQLGSL‑‑‑‑‑‑‑‑‑‑‑‑‑‑‑‑‑‑‑‑‑‑‑‑‑‑‑‑‑‑‑‑‑SAN‑HSGW‑‑‑DC‑S‑‑‑INRTEAELVEEIA

>G8A1T6

GEDTRRNFVAHLNAALSNAEINT‑YIDD‑RIQ‑KGTDLEPELFRAIEDSRISIVVFSENYVHSSWCLKELEQIMKCR‑VNCGQI‑VEPVFYHVEPSVLRHQAGDFGKALEET‑KRSSEGE‑‑KMN‑‑TLSTWQIALTEVAN‑ISGW‑‑‑DT‑K‑‑‑‑NKDDVELISQIV

>G7KKS8

GFDTRNGFTGHLWKALNDIGILA‑FIDDTEFS‑RGEETKPAIFKAIHVSRIAIIVFSDNYAGSKFLLEELAFIVDNF‑QSDNRF‑IVPVYYNIEASHVRHQSGPFEAAFVKH‑EERF‑‑‑HENRE‑‑KVLKWKTALSQVAN‑LPGW‑‑‑HF‑D‑‑G‑VEYEHQFLQKIV

>G7IQ97

GIDTRNTFTGSLYNSLDQKGIHT‑FIDEKEIQ‑KGEEITPSLLQAIQQSRIYIVVFSSNYASSTFCLNELVMILECS‑NTRRRL‑LLPVFYDVDPSQVRHQRGAYGEALRKH‑EERFSDD‑‑K‑D‑‑KVQKWRDALCQAAN‑ISGW‑‑‑HF‑QH‑G‑SQPEYKFIGNIV

>G7KGI3

GEDIHHGFLGHLFKAFSQKQINV‑FVDD‑KLK‑RGNDISHSLFEAIEGSFISLIIFSENYASSRWCLEELVKIIECK‑EKYGQI‑VIPVFYGVDPTDVRHQKKSYENAFVEL‑GKRYNSS‑‑‑‑‑‑‑EVQIWRNTLKISAN‑LSGI‑‑‑TS‑‑‑‑‑‑‑‑NDAELLEEII

>G7KIF6

GIDTRSGFTGHLYKALCDKGIRT‑FIDDKELQ‑RGDEITPSLLKSIEHSRIAIIVFSENYATSSFCLDELVHIINYF‑KEKGRL‑VLPVFYGVEPSHVRHQNNKYGEALTEF‑EEMFQNNKENMD‑‑RLQKWKIALNQVGN‑LSGF‑‑‑HF‑KK‑‑‑DAYEYEFIKKIV

>G7KIG6

GSDTRYGFTGNLYKALTNKGIHT‑FIDDNHLP‑RGSEITPSLIKAIEESRIFIPIFSTNYASSSFCLDELVHM‑‑‑‑‑‑‑‑‑‑‑‑‑‑‑‑‑‑‑‑SFTATRQRVASYGEALADH‑EKRFQNDKDNME‑‑RLQRWKMAMRQVAN‑LSGY‑‑‑HF‑S‑‑‑‑LGYEYEFIGKIV

>G7L6S8

GSDIRKNFLSHVLDALSRKGIIV‑FSDK‑KLI‑TGDELS‑AIQRAIEKSLISLVIFSPNFASSHWCLDELVKIVECR‑ANYGRV‑LMPVFYQVYPSDVRHQNGTYRDAFAQH‑EQKYSSY‑‑‑‑‑‑‑KVLSWRSALKQSAN‑MSGF‑‑‑DS‑‑‑‑‑‑‑‑DDAKLVEEIV

>G7KJ25

DIDTLYGFTGNLYKALIDKGIKT‑FIDDNDLE‑RGDESTPSLVKAIEESRILIPIFSANYASSSFCLDELVHIIHCY‑KTRGCS‑VLPVFYGADPTHVRHQTGSYGEHLTKH‑EDKFQNNKENME‑‑RLKKWKMALTQAAN‑FSGH‑‑‑HF‑S‑‑‑‑QGYEYELIENIV

>G7KJC7

GADTRHGFTGNLYDALCKSGVHT‑FKDDEELQ‑RGGEITASLMKAIEESRIFIPVFSKNYASSSFCLDELVHIIRYS‑KSKGRL‑VLPVFYDIAPTHVRKQTGSIGEELAKH‑QEKF‑‑‑QKNME‑‑RLQEWKMALKEAAE‑LSGH‑‑‑HF‑NA‑G‑TEYESNFIQGIV

>G7IM52

GPDTRNTFVDHLYAHLKRKGIFA‑FKDDQRLE‑KGESLSPQLLQAIQNSRVSIVVFSERYAESTWCLEEMATVAECR‑KRK‑QT‑VFPVFYDVDPSHVRKHIGVFKAN‑NSH‑TK‑‑TY‑‑‑DRN‑‑KVVRWQEAMTELGN‑LVGF‑‑‑DV‑R‑‑‑‑YKPEFTEIEKIV

>G7JKN1

GKDIRDGFLGHLVKAFRQKKINV‑FVDN‑IIK‑RGDEIKHSLVEAIEGSLISLVIFSKNYSSSHWCLDELVKIIECK‑KDRGQI‑IIPVFYGVRSKIV‑‑‑‑‑‑‑‑‑‑LDEL‑EKKDNFS‑‑‑‑‑‑‑KVEDWKLALKKSTD‑VAGI‑‑‑RL‑‑‑‑‑‑‑‑NDAELLEEIT

>G7JF24

GEDTRNNFTDHLFDTFHREGISA‑FRDDTNLP‑KGESIGPKLLCAIENSQVFVVVLSRNYAFSTSCLQELEKILEW‑VKVSKK‑‑VLPVFYDVDPSMVRKQSGIYGEAFVKH‑EQRFQ‑‑‑QDSQ‑‑MVQRWREALIQVAD‑LSGW‑‑‑DL‑H‑‑‑‑DRRQSPEIKKIV

>G7KIF1

GSDTRDGFTGHLYKALTDKGIHT‑FIDDCDLK‑RGDEITPSLIKAIEESRIFIPVFSINYASSKFCLDELVHIIHCY‑KTKGRL‑VLPVFYGVDPTQIRHQSGSYGEHLTKH‑EESFQNNKKNKE‑‑RLHQWKLALTQAAN‑LSGY‑‑‑HY‑S‑‑‑‑PGYEYKFIGKIV

>G7LI64

GEDTRSTFTAQLYQTLKKENIIT‑YI‑DENLN‑KGEEVGPALVQAIQESRMSLVVFSENYASSKWCLDELLKILECG‑KFHDQV‑VIPVFYRIDPSDVRHQTGSYKEPFANY‑QIK‑‑‑S‑‑NED‑‑KVSQWKAALTEIAN‑ISGW‑‑‑DS‑R‑‑‑‑‑GDDSQLIEKIV

>G7KJ66

GTDTRFGFTGNLYKALSDKGIHT‑FIDDKELP‑TGDEITPSLRKSIEESRIAIIIFSKNYATSSFCLDELVHIIHCF‑REKVTK‑VIPVFYGTEPSHVRKLEDSYGEALAKH‑EVEFQNDMENME‑‑RLLKWKEALHQFHS‑WVPL‑‑‑FI‑SI‑L‑NKYEYKFIEEIV

>G7JLU8

GNDVRDGFLGKLYEAFIRKQINI‑FVDY‑KLK‑KGDDISHSLGEAIEGSSISLVIFSENYASSHWCLEELVKIIECR‑EKYGQL‑VIPIFYEVDPTNVRYQKKSYENAFVKL‑EKRYNSS‑‑‑‑‑‑‑EVKIWRHTLKISAN‑LVGF‑‑‑TS‑‑‑‑‑‑‑‑NDAELLEEIT

>G7LDU6

GEDTRECFTKKLYESLHKQGVRA‑FMDDEGLD‑RGDHIATTLLEAIDDSAASIVIISPNYADSHWCLDELNRICDLE‑‑‑‑‑RL‑IIPVFYKVDPSHVRKQLGPF‑DGFNYL‑EKRFAN‑‑‑EKD‑‑KILKWRDSMLKIGG‑LAGF‑‑‑‑VFNS‑SDDGEHDNLIRRLV

>G7K8D4

SEDTRNNFTSHLNGALKRLDIRT‑YID‑NNLN‑SGDEISTTLVRAIEEAELSVIVFSKNYAASKFCLDELMKILECK‑RMKGKM‑VVPIFYDVDPTDVRNQRGSYAEAFAKH‑EKNSEEK‑‑‑I‑‑‑KVQEWRNGLMEAAN‑YSGW‑‑‑DC‑N‑‑‑VNRTELELVEEIA

>G7JCP8

GQDTHNNFADHLFAALQRKGIVA‑FRDDSNLK‑KGESIAPELLHAIEASKVFIVLFSKNYASSTWCLRELEYILHC‑SQVSGT‑‑VLPIFYDVDPSEVRHQNGSYGEALAKH‑EERFQ‑‑‑HESE‑‑MVQRWRASLTQVAN‑LSGW‑‑‑DM‑H‑‑‑‑HKPQYAEIEKIV

>G7JCM4

GEDTRFNFIDHLFAALQRKGIFA‑FRDDANLQ‑KGESIPPELIRAIEGSQVFIAVLSKNYSSSTWCLRELVHILDC‑SQVSGR‑‑VLPVFYDVDPSEVRHQKGIYGEAFSKH‑EQTFQ‑‑‑HDSH‑‑VVQSWREALTQVGN‑ISGW‑‑‑DL‑R‑‑‑‑DKPQYAEIKKIV

>G7KJS7

GTDTRYGFTGNLYKALCDGGVRT‑FIDHKDLH‑EGDRITQSLVKAIEESRILIPVFSKNYASSLFCLDELVHIIHRY‑EEKGCF‑VFPIFCDVEPSHVRHQTGSYGEALAKH‑EERFQNNKENMK‑‑RLHKWKMALNQAAN‑LSGH‑‑‑HF‑NP‑R‑NGYEFEFIREIV

>G7LC17

GLDTRYGFTGNLYKALYDKGIHT‑FIDDEELQ‑RGHEITPSLLEAIEESRIAIIVLSKNYASSSFCLHELVKILDCI‑KGKGRL‑VWPIFYDVDPSDVRKQTGSYGEALAML‑GERF‑‑‑‑‑NDN‑‑NLQIWKNALQQVAN‑LSGW‑‑‑HF‑KI‑G‑DGYEYEFIGKIV

>G7L6S0

GPDIREVFLPHLIKAFSQKKIVY‑FVDY‑KLT‑KGNEISQSLFEAIETSSISLVIFSQNYASSSWCLDELVKVVDCR‑EKDGNI‑LLPVFYKVDPTIVRHQNGTYADAFVEH‑EQKYNWT‑‑‑‑‑‑‑VVQRWRSALKKSAN‑INGF‑‑‑HT‑‑‑‑‑‑‑‑NDAELVEEIV

>G7KF73

GQDTVE‑‑‑‑‑‑LKGFCEKGINT‑FIDDQELR‑KGEEITPALMM‑‑‑‑‑‑‑AIVIFSENYASSTFCLEALRKIME‑‑‑‑‑‑‑‑‑‑‑‑‑‑‑‑‑FDPSDVRHQKGSYAKAI‑‑‑‑‑‑‑‑‑‑‑‑‑‑‑‑‑‑‑VKQWRLALQEAAN‑LVGW‑‑‑HF‑RH‑R‑YDYEYELIGKIV

>G7KPT3

GTDTRFGFTGNLYKALSDKGIHT‑FIDDKELK‑RGDEITPSLRKSIEDSRIAIIVFSKDYASSSFCLDELVHIIHYF‑KEKSRL‑VLPIFYGTEPSQVRKLNDSYGESFAKH‑EEGFQNNKEHME‑‑RLLTWKKALNEAAN‑LSGH‑‑‑HF‑NQ‑G‑NEYERDFIEKIV

>G7IW57

GIDTRNNFTGNLYNSLNQSGIQT‑FIDDEEIQ‑KGEEITPTLLKAIKESRIFIAILSPNYASSTFCLTELVTILECS‑KSKGRW‑FLPIFYDVEPTQIRNLTGTYAEAFAKH‑EVRFRDE‑‑K‑D‑‑KVQKWRDALRQAAS‑LSGW‑‑‑HF‑QP‑G‑SQQEYKFIRMIV

>G7KIF2

GIDTRHGFTGNLYKALIDKGIHT‑FIDDNDLL‑RGDEITPSLVKAIEESRIFIPIFSANYASSSFCLDELVHIIHCY‑KTKGCS‑VLPVFYGVDPTHIRHQTGSYGEHLTKH‑EKKFQNNKENMQ‑‑RLEQWKMALTKAAN‑LSGY‑‑‑HC‑S‑‑‑‑QGYEYKFIENIV

>G7L6R9

GSDIRKHFLSHVLEALSRKRIVV‑FSDK‑KLK‑TGDELS‑AIQRAIEKSFISLVIFSPNFASSYWCMEELVKIVECR‑EKYGRI‑LMPVFYQVEPTVVRYQNGIYRDAFAQH‑EQNYSSY‑‑‑‑‑‑‑KVLRWRSALKQSAN‑ISGF‑‑‑DS‑‑‑‑‑‑‑‑DDAKLVEEIL

>G7KIH4

GTDTRFHFIGHLYKALCDCGIRT‑FIDDKELH‑GGEEITPSLVKAIEDSGIAIPVFSINYATSSFCLDELVHIVDCF‑KTKGHL‑ILPIFYEVDPSHVRHQTGSYGAYIG‑‑‑‑‑‑‑‑‑‑‑‑NME‑‑RLRKWKIALNQAAN‑LSGH‑‑‑HF‑NL‑GNNSYEYELIGKMV

>G7KJQ3

GSDTRYSFIGNLHKDLCRKGIRT‑FIDDRELK‑GGDEITPSLFKHIEETRIFIPVLSTNYASSSFCLDELVHIIHCF‑KESSRL‑VLPIFYDVEPSHVRHQHGSYAKALDDH‑IEKFQNNKNNME‑‑RLQKWKSALTQTAN‑FSGH‑‑‑HF‑NP‑R‑NGYEYEFIEKIV

>G7KPH8

GEDTRNNFTDFLFDALQTKGIIV‑FLDDTNLP‑KGESIGPELIRAIEGSQVFVAFFSRNYASSTWCLQELEKICEC‑IKGSGK‑‑VLPVFYDVDPSEVRKQSEIYSEAFVKH‑EQRFQ‑‑‑QDSK‑‑KVSIWREALEQVGD‑ISGW‑‑‑DL‑H‑‑‑‑DKPLAREIKEVV

>G7LEE4

GKDTRNNFVSHLYAALTNVRINT‑FLDDEELG‑KGNELGPELLQAIQGSQMFIVVFSENYARSSWCLDELLQIMECR‑ANKGQV‑VMPVFYGISPSDIRQLARRFGEAFNNN‑‑‑‑‑‑‑D‑‑ELD‑‑QIY‑‑‑MALSDASY‑LAGW‑‑‑DM‑S‑‑‑‑NSNESNTVKQIV

>G7JZM1

GDDSRNSLVSHLYAALSNARINT‑FLDDEKLH‑KGSELQPQLLRAIQGSQICLVVFSENYSRSSWCLLELEKIMENR‑GTHGQI‑VIPIFYHIDPAIVRRQLGNFGKALEIT‑KKQSKRE‑‑KQK‑‑LLQTWKSALSQATN‑LSGW‑‑‑DV‑T‑‑‑‑SRNESELVQKIV

>G7JKL7

GKEIRSGFLSHLVKAFCQKQINA‑FVDD‑KLK‑RGDDISDSLGEAIEGSFISLIIFSENYACSHWCLKELVKIVECK‑EKYAQI‑VIPVFFRVDPTDIRHQKRSYENAFAEH‑EKKYSSY‑‑‑‑‑‑‑EVQMWKHALKISAN‑LSGI‑‑‑TS‑‑‑‑‑‑‑‑RKEKSIETL‑

>G7JKM7

GEDIRANFLSHLIEDFDRKKIKA‑FVDD‑KLK‑RGDEIPQSLVRAIEGSLISLIIFSHDYASSCWCLEELVTTLQCR‑EKYGQI‑VIPIFYQVDPTDVRYQNKSYDNAFVEL‑QRGYSST‑‑‑‑‑‑‑KVQIWRHALNKSAN‑LSGI‑‑‑KS‑‑‑‑‑‑‑‑NDVQLLKEIV

>G7IQA8

GIDTRNNFTRDLYDILDQNGIHT‑FFDEQEIQ‑KGEEITPSLLQAIQQSRIFIVVFSNNYASSTFCLNELVMILDCS‑NTHRRL‑LLPVFYDVDPSQVRHQSGAYGEALKKH‑EERFSDD‑‑K‑D‑‑KVQKWRDSLCQAAN‑VSGW‑‑‑HF‑QH‑G‑SQSEYQFIGNIV

>G7J146

GKDTRLNFTDHLFA‑‑‑‑‑‑‑‑‑‑‑‑‑‑‑‑LK‑KGESIAPELLRAIEDSQIFVVVFSKNYASSVWCLRELECILQS‑FQLSGK‑‑VLPVFYDVDPSEVRYQKGCYAEALAKH‑EERFQ‑‑‑QNFE‑‑IVQRWREALTQVAN‑LSGW‑‑‑DV‑R‑‑‑‑YKPQHAEIEKIV

>G7IW71

GIDTRNNFTGNLYHSLHQRGIQT‑FMDDEEIQ‑KGEEITPTLLQAIKQSRIFIAIFSPNYASSTFCLTELVTILECS‑MLQGRL‑FLPVFYDVDPSQIRNLTGTYAEAFAKH‑EVRFGDE‑‑KDS‑‑KVQKWRDALRQAAN‑VSGW‑‑‑HF‑KP‑G‑FESEYKFIEKIV

>G7LGU0

GSDTRCDFSGFLNKYLIDRG‑RT‑FFDDGELE‑RGTQIT‑EIPKAIEESRIFIPVLSENYASSSFCLDELVKILEEK‑KGNGR‑‑VFPVFYYVNISDVKNQTGSYGQALAVH‑KNR‑‑‑‑‑‑‑ME‑‑RFEKWINALASVAD‑FRGC‑‑‑HMER‑‑‑‑AGYEIRYIYEII

>G7L6L8

GEDTRKGFTDHLRAALERKGITT‑FRDDKDLE‑RGKNISEKLINAIKDSMFAITIISPDYASSTWCLDELQMIMECS‑SNNNH‑‑VLPVFYGVDPSDVRHQRGSFEEAFRKH‑LEKFGQN‑‑‑SD‑‑RVERWRNAMNKVAG‑YSGW‑‑‑DS‑‑‑‑‑KG‑HEALLVESIA

>G7LIX4

GDDTRAGFTSHLYADLCRSKIYT‑YID‑YRIE‑KGDEVWVELVKAIKQSTIFLVVFSENYASSTWCLNELVEIMECC‑NKEDKVVVIPVFYHVDPSHVRKQTGSYGTALIKH‑KKQGKND‑‑‑‑‑‑‑DMQNWKNALFQAAN‑LSGF‑‑‑HS‑‑‑‑‑‑‑‑TESEMIEAIT

>G7LEF6

GEDIGKSFVSHLVNALRKARITT‑YIDGGQLH‑TGTELGPGLLAAIETSSISIIVFSKNYTESSWCLDVLQNVMECH‑ISDGQL‑VVPVFHDVDPSVVRHQKGAFGQVLRDT‑KRTSRKG‑‑EIE‑‑DVSSWKNALAEAVS‑IPGW‑‑‑NA‑I‑‑‑‑SRNEDELVELIV

>G7KL77

GEDTRYGFTGNLKKALDDKGVRT‑FIDDEKLK‑KGDEITPSLLKAIEDSMMAIIVLSENYASSSFCLQELSHILDTM‑KDKGRY‑VLPVFYKVDPSHVRKLKRSYGEAMKKH‑DVAS‑‑‑SSSHN‑‑MNNKWKDSLHQVAN‑LSGS‑‑‑HY‑K‑‑G‑DKYEYEFIENIV

>G7LF39

GADTRKTFISHLYTALTNAGINT‑FLDNENLQ‑KGKELGPELIRAIQGSQIAIVVFSKNYVHSRWCLSELKQIMECK‑ANDGQV‑VMPVFYCITPSNIRQYATR‑‑‑‑FSET‑LF‑‑‑FD‑‑ELV‑‑PM‑‑‑‑NTLQDASY‑LSGW‑‑‑DL‑S‑‑‑‑NSNESKVVKEIV

>G7IM43

GTDTRNTFIDHLYHHLIRKGISA‑IRDS‑‑‑‑‑‑‑‑‑‑‑‑‑‑‑‑‑‑‑‑‑‑‑‑‑‑‑‑‑‑‑‑‑‑‑‑‑‑‑‑‑‑‑‑‑‑‑‑‑‑REN‑QT‑VIPIFYDIDPSYVRSNLSAFNGDYNSR‑TKKLNY‑‑‑DPD‑‑KVSRWERVVIQL‑‑‑‑‑‑‑‑‑‑‑‑‑‑‑‑‑‑‑SPEFTEIEKIV

>G7KJR3

GSDTRYRFTGNLNRALCDKGIRT‑FMDDRELQ‑GGEEITSSLFKAIEESRIFIPVLSINYASSSFCLDELVHIINCF‑KESGRL‑VLPIFYDVEPSHVRHHTGSYGKALDDH‑IKKFQNNKDSME‑‑RLQKWKSALTQTAN‑FSGH‑‑‑HF‑NP‑G‑NGYEHEFIEKIV

>G7LGU9

GDDTRFSFTGFLSHSLNNRG‑YT‑AINHRD‑‑‑‑‑‑‑‑‑‑‑‑‑‑SIQHCRIFIFIISRDYASR‑‑‑LDELVNIMDLA‑KGNGRQRILPVYYHVNPSDVRHQSGSFGEALSSF‑YNN‑‑‑‑‑‑‑LS‑‑DFEKRNTVLRQVAD‑FRGW‑‑‑HLDP‑‑‑‑AGYEHQYIEEIL

>G7KIG7

GSDTRFGFTGHLYKALCDSGIHT‑FIDDTELH‑RGDEISPSLIKAIEESMIYIPVLSINYASSIFCLEELVKIIKSF‑HSGHHH‑ILPVFYDVHPSQVRSRTGSFGEAIDKH‑KEK‑‑‑‑‑‑GTS‑‑RVYEWNNALIQVAN‑LSGY‑‑‑HW‑SD‑G‑NKYEHEIIGMIV

>Q2HUD1

GEDTRKGFTDHLCAALERKGITT‑FKDDKDLE‑RGQVISEKLINAIKDSMFAITILSPDYASSTWCLDELQMIMECS‑SKNNH‑‑VLPVFYGVDPSDVRHQRGCFEEAFRKH‑QEKFGQH‑‑‑SD‑‑RVDRWRDAFTQVAS‑YSGW‑‑‑DS‑‑‑‑‑KG‑HEASLVENIA

>G7LGT8

GSDTRCGFSGFLNKYLIDRG‑RT‑FFDDGELE‑RGTQIT‑EIPKAIEESRIFIPVLSENYASSSFCLDELVKILEEK‑KGNGR‑‑VFPVFYYINISDVKNQTGSYGQALAVH‑KNR‑‑‑‑‑‑‑ME‑‑RFEKWINALASVAD‑FRGC‑‑‑HMER‑‑‑‑AGYEFRYIYEII

>G7K8C7

GEDTRYTFTSHLHATLTRLKVGT‑YID‑YNLQ‑RGDEISSTLLMAIEEAKVSIVIFSKNYGNSKWCLDELVKILECK‑KMKGQI‑LLPIFYDIDPSHVRNQTGSYAEAFVKH‑EKQFQGK‑‑‑LE‑‑KVQTWRHALREAAN‑ISGW‑‑‑EC‑S‑‑‑VNRMESELLEKIA

>G7JF25

GEDTRNNFTYHLFDAFNREGILA‑FRDDTNLP‑KGESIASELLRAIEDSYIFVAVLSRNYASSIWCLQELEKILEC‑VHVSKK‑‑VLPVFYDVDPPVVRKQSGIYCEAFVKH‑EQIFQ‑‑‑QDSQ‑‑MVLRWREALTQVAG‑LSGC‑‑‑DL‑R‑‑‑‑DKRQSPGIKNIV

>G7JCM5

GEDTRFNFIDHLFAALQRKGIFA‑FRDDTNLQ‑KGESIPPELIRAIEGSQVFIAVLSKNYASSTWCLRELVHILDC‑SQVSGR‑‑VLPVFYDVDPSEVRHQKGIYGEAFSKH‑EQTFQ‑‑‑HESH‑‑VVQSWREALTQVGN‑ISGW‑‑‑DL‑R‑‑‑‑DKPQYAEIKKIV

>G7IW33

GEDTRYGFTGHLYNALHQRGINT‑FMDDEEIK‑RGEQISPTLFKAIQESRIAIIVFSKTYASSKWCLQELVKIVECK‑KE‑‑‑LVIFPVFYNVDPSEVRNQKTSYGEQLAKY‑E‑KM‑‑‑‑‑‑KE‑‑EVQSWRLALHETAS‑LAGW‑‑‑HF‑R‑‑‑‑DGYEYEFIKRIG

>G7KKS2

GDDTRYSFTGYLYNTLCQKGINT‑FKDDIKLK‑KGEEISTDLLQAIDESRIAIIVCSENYASSPWCLDELVKIMECK‑EEKGQL‑VCIVFFYVDPSNVRHQRKSFARSMAKH‑EENP‑‑‑KISEE‑‑KISKWRSALSKAAN‑LSGW‑‑‑HF‑K‑‑‑‑HGYEYELIQEIT

>G7LI82

GEDTRYGITSHLHAALIHKSIKT‑YV‑DSLLE‑RGEDIWPTLAKAIEESHVSIVVFSENFATSTWCLEELVKVLECR‑KVKGQV‑VIPVFYKTDPSDIRNQTGSYENAFAKH‑ERN‑‑‑D‑‑‑‑‑‑‑KVLNWKVALAEAAT‑ISGW‑‑‑HT‑Q‑‑‑‑‑KEESILIDKIV

>G7KIF0

GTDTQFGFTGNLYKALSDKGINT‑FIDDKELK‑KGDEITPSLLKSIEESRIAIIVFSKEYASSLFCLDELVHIIHCS‑NEKGSK‑VIPVFYGTEPSHVRKLNDSYGEALAKH‑EDQFQNSKENME‑‑WLLKWKKALNQAAN‑LSGH‑‑‑HF‑NL‑G‑NEYERDFIEKIV

>G7KIF5

GTDTRYGFTGNLYKALIDKGIHT‑FIDDNDLQ‑RGDEITPSLIKAIEESRIFIPVFSINYASSKFCLDELVHIIHCY‑KTKGRL‑VLPIFFGVDPTNVRHHTCSYGEALAEH‑EKRFQNDKDNME‑‑RLERWKVALSQAAN‑LSGY‑‑‑HD‑SP‑‑‑PRYEYKLIGEIV

>G7IQB0

GIDTRNNFTRDLYDILDQNGIHT‑FFDEQEIQ‑KGEEITPSLLQAIQQSRIFIVVFSNNYASSTFCLNELVMILECS‑NTHGRL‑FLPVFYDVDPSQVRHQSGAYGDALKKH‑EKRFSD‑‑‑‑‑D‑‑KVQKWRDALCQAAN‑VSGW‑‑‑DF‑QH‑G‑SQSEYKFIGNIV

>G7JDB8

G‑RTRYSFTDHLYRSLLRHGINV‑FRDNPNL‑‑NGDEIRLSLLQAIEASRISIVVLCKDYASSTWCLDELVKIVDCY‑EMKGKT‑VFVIFYKVEASDVRHQRKSYEIAMIQH‑EKRFGKS‑‑‑‑E‑‑KVKKWRSALKRVCA‑LSGL‑‑‑Y‑YK‑‑‑‑D‑YESEFIEKIV

>G7IQ90

GIDTRNNFTRDLYDSLDQNGIHT‑FFDEKQIQ‑KGEQITPALFQAIQQSRIFIVVFSNNYASSTFCLNELALILDCS‑NTHGRL‑LLPVFYDVDPSQVRHQSGAYGEALKKQ‑EERFCDD‑‑K‑D‑‑KVQKWRDALCQAAN‑VSGW‑‑‑HF‑QH‑G‑SQSEYKFIGNIV

>G7KK90

GADTRFGFTGNLYKALTDKKIRT‑FIDDKELQ‑RGDEITPSLVKAIQESRIAIPIFSTNYASSSFCLDELVHIVECV‑KRKGRL‑VLPIFYDVDPSHVRHQTGSYGKGMTDL‑EERFKNNKE‑‑‑‑‑KLQKWKMALNQVAN‑LAGY‑‑‑HF‑KL‑G‑NEYEYEFIVKIV

>G7KL58

GEDTRHGFTGNLWKALDDKGVRT‑FMDDENLQ‑KGDEITPSLIKAIEDSQIAIVVLSKNYASSSFCLQELSKILDTM‑KDKGRF‑VMPVFYKVDPSDVRKLKGTYGDAMDKL‑GEAS‑‑‑SSSHN‑‑‑‑‑KWKDSLHQVAN‑LSGF‑‑‑PY‑E‑‑KRDGYVHEFIEKIV

>G7IQ96

GIDTRNTFTGNLYNSLDQKGIHT‑FLDEEEIQ‑KGEQITRALFQAIQQSRIFIVVFSNNYASSTFCLNELAVILECS‑NTHGRL‑LLPVFYDVEPSQVRHQSGAYGDALKKH‑EERFSDD‑‑K‑D‑‑KVQKWRDALCQAAN‑VSGW‑‑‑HF‑QH‑G‑SQSEYKFIGNIV

>G7KKA1

GEDTRYGFTGNLWKALHDKGVRT‑FMDDEELQ‑KGEEITPSLIKAIENSNMAIVVLSKNYASSSFCLKELSKILE‑‑‑‑‑‑GLF‑VLPVFYKVDPSDVRKLEKSYGEAMDK‑‑‑HKA‑‑‑SSNLD‑‑‑‑‑KWKMSLHQVAN‑LSGF‑‑‑HY‑K‑‑KRDGYEHEFIGKIV

>G7JLT0

GDDTQCHFTSHFFSSKCR‑‑‑‑‑‑‑‑‑NYRLQ‑RRSFRSKRFVHRKEGSRISIIVFSKNYADSPWCMQELIQILECY‑RTTGQV‑VLPVFYDVYPSDVRRQSREFGQSFQHL‑‑‑‑‑‑‑‑‑‑NVEG‑HSLKWIDALHDVAG‑IAGF‑‑‑VV‑‑‑‑‑PNYNECEVIKDIV

>G7KJQ5

GSDTRYGFTGNLYKDLCRKRIRT‑FIDDKDLQ‑RGDEITPSLFKAIEESRIFIPILSINYASSSFCLDELVHIIHCF‑KENGQV‑‑‑‑‑‑‑‑‑‑‑‑‑‑‑‑‑‑‑‑‑‑‑‑‑‑‑‑‑‑‑‑‑‑NSTDSME‑‑RLQKWKMALTQTAN‑FSGH‑‑‑HF‑SP‑G‑NGYEYEFIEKIV

>G7KP04

GEDTRNNFTDFLFDALQTKGIIV‑FSDDTNLP‑KGESIGPELLRAIEGSQVFVAVFSINYASSTWCLQELEKICEC‑VKGSGK‑‑VLPVFYDVDPSDVRKQSGIYGEAFIKH‑EQRFQ‑‑‑QEHQ‑‑KVSKWRDALKQVGS‑ISGW‑‑‑DL‑R‑‑‑‑DKPQAGEIKKIV

>G7JLX5

GEDTRASFTSHLSTSLQSSGIIV‑FKDDHSLQ‑RGHRISKTLLQAIQESRISVVVFSKNYADSQWCLQELMQIMECF‑RTTRQV‑VLPVFYDVHPSEVRSQTGDFGKAFQNL‑DEF‑‑‑‑‑‑‑‑‑‑‑‑VPKWRDALRNAAG‑IAGF‑‑‑VV‑‑‑‑‑LNSNESEVIKDIV

>G7J6M2

GEDTRRNFTSHLYEALSKK‑VIT‑FIDDNELE‑KGDEISSALIKAIEKSSASIVIFSKDYASSKWCLNELVKILECK‑KDNGQI‑VIPVFYEIDPSHVRNQKGSYMLAFEKH‑EQDLKQS‑‑‑KD‑‑KLQKWKDALTEAAN‑LAGW‑‑‑YS‑QN‑‑‑‑‑NDSIFIKYII

>G7KYW5

GEDTRRTIVSHLYTALCNAGINT‑FLDDKKLA‑KGEELGPELYTAIKMSHIFIAVFSPNYAQSSWCLNELAHIMELR‑HSYSRV‑VIPLFYHVDPSDVRKLKGDFGKGLKVS‑DKQSGAE‑‑REE‑‑VMSKWRRALAEVTN‑LVGW‑‑‑DA‑N‑‑‑‑NRNEGDLVQKLV

>G7JKM5

GEDIRHGFLGHLAKM‑‑‑‑‑‑‑‑‑‑‑‑‑‑‑‑‑‑‑‑‑‑TYHSIFEAIEGSFISLIIFSENYASSRWCLEELVKIIECR‑EKNGQI‑VIPVFYEVGPTDVRHQKKSYENALVGH‑EKNYILS‑‑‑‑‑‑‑RVQKWRQTLEKSAN‑LSGI‑‑‑KS‑‑‑‑‑‑‑‑NDVEILEEII

>G7KL60

GEDTRHGFTGYLKKALDDKGVRT‑FMDAKELK‑KGEEITPSLLKAIEDSMMAIIVLSENYASSSFCLQELSHILDTM‑KDKGRY‑VLPVFYKVDPSDVRKLKRSYGEAMDKH‑DAAS‑‑‑SSSHD‑‑VNNKWKASLQQVAN‑LSGS‑‑‑HY‑K‑‑G‑DEYEYEFIEKII

>G7LI75

GEDTRTNFTAQLHRALTDRSIES‑YI‑DYSLV‑KGDEVGPALAEAIKDSHMSIVVFSKDYATSKWCLDELLQILHCR‑ELFGQV‑VIPVFYNIDPSHVRHQKESYEMAFARY‑ERI‑‑‑S‑‑YVD‑‑RVSEWRAALKMAAN‑ISGW‑‑‑DS‑R‑‑‑‑‑RDDSQVIDNIV

>G7KPF0

GEDTRNNFTDFLFDALETKGIMV‑FRDVINLQ‑KGECIGPELFRAIEISQVYVAIFSKNYASSTWCLQELEKICEC‑IKGSGK‑‑VLPVFYDVDPSEVRKQSGIYSEAFVKH‑EQRFQ‑‑‑QDSM‑‑KVSRWREALEQVGS‑ISGW‑‑‑DL‑R‑‑‑‑DEPLAREIKEIV

>G7JLT1

GEDTRTSFTS‑‑‑‑‑‑‑‑‑‑‑‑‑‑‑‑‑‑‑‑‑‑‑QGDYISTSLLQGIYGSRVSVIVFSKNYAGPQWCQIQLLLVFNKK‑RVAGSI‑IISVI‑‑‑‑‑‑KMRILCWMCGKTRQNM‑‑‑‑‑‑‑‑‑‑‑VE‑‑VRLRW‑‑‑‑‑‑‑‑‑‑‑‑G‑‑‑‑‑‑‑‑‑‑‑‑‑‑NESEDIKDIV

>G7K107

GSDTRNTFVDHLYAHLIRKGIFT‑FKDDAQLN‑KGHSISTQLLHAIRQSRVSIIIFSKDYASSTWCLDEMATIADCQ‑LNN‑‑‑‑‑HTVFYDVAPSDVRKQKGVYQNVFAVH‑SKISKH‑‑‑EPH‑‑KVDCWKRAMTCLAG‑SSGW‑‑‑DV‑R‑‑‑‑NKPEFEEIEKIV

>G7KKT5

ADDTASDFAGNLYKALNDRGIRT‑FMDDN‑‑K‑K‑E‑NREKHFKTIEESKTAIIVLSRNYASSLSCLEQLASILDCR‑KR‑‑RL‑VWPVFYNMGPSCCKTRTGKMGKAWSNH‑Y‑K‑‑‑N‑‑RLK‑‑KTKK‑‑‑ALHQVAD‑LSGF‑‑‑H‑LNN‑G‑DGSELELIERIV

>G7KJ34

GTDTRYGFTGNLLKALIDKGIRT‑FHDDDDLQ‑RRDKVTPII‑‑‑IEESRILIPIFSANYASSSSCLDTLVHIIHCY‑KTKGCL‑VLPVFFGVEPTDVRHHTGRYGKALAEH‑ENRFQNDTKNME‑‑RLQQWKVALSLAAN‑LPSY‑‑‑HD‑DS‑‑‑HGYEYELIGKIV

>G7JF29

GADTRFNFTDHLFSALQIRGIVA‑FRDDTKLK‑KGESIAPELLRAIEASRTFIVVFSNNYASSTWCLRELQYILHC‑VQLSGK‑‑VLPVFYDVDPSEVRKQSGSYKKAFAQH‑EERFK‑‑‑QDTE‑‑VLQGWRTALTQVAN‑LSGW‑‑‑DI‑R‑‑‑‑DKPQSAEIKKIV

>G7JMY5

GEDTRASFTSHLTFSLQNAGIIV‑FKDDQSLE‑RGEHISTSLLQAIEISRIAVIVFSKNYADSSWCLRELVQIMSCY‑STIGQV‑VLPVFYDVDPSEVRRQTGDFGKSFQNL‑EER‑‑‑‑‑‑PFSN‑KVRKWIDALHTAAG‑LAGF‑‑‑VV‑‑‑‑‑LNSNESEVIRDIV

>G7IUH0

GTDIRHGFLSHLRKELRQKQVDA‑YVDD‑RLE‑GGDEISKALVKAIEGSLMSLIIFSKDYASSKWCLEELVKIVECM‑ARNKQV‑VIPVFYNVNPTDVRHQKGTYGDSLAKH‑EKKGSLA‑‑‑‑‑‑‑KVRNWGSALTIAAN‑LSGF‑‑‑HS‑‑‑‑‑‑‑‑DEVELIEEIV

>G7KJ43

GADTRHGFTGNLYKALTDKGIYT‑FIDDNDLQ‑RGDEITPSLKNAIEKSRIFIPVFSENYASSSFCLDELVHITHCY‑DTKGCL‑VLPVFIGVDPTDVRHHTGRYGEALAVH‑KKKFQNDKDNTE‑‑RLQQWKEALSQAAN‑LSGQ‑‑‑H‑‑YK‑‑‑HGYEYEFIGKIV

>G7JEW9

GEDTRASFTAHLNASLLNAGINV‑FKDDDSIY‑KGARISKSLPEAIEQSRIAVVVFSKHYADSKWCLNELVKIMKCH‑RAIRQI‑VLPVFYDVDPLEVRHQKKKFGKAFQNIQESS‑‑‑‑‑‑‑‑‑‑‑RRRNWTTALHEAAG‑LAGF‑‑‑VV‑‑‑‑‑LHFNESEAIKDIV

>G7LF30

GEDTRSSFVSHLHAALSNAGINT‑FLDDKKLE‑KGEELGPELLRAIEVSRISIIVFSKSYITSSWCLKELEQIMKCR‑KNYGQV‑VMPIFYHVDPSALRHQKDGYGKALQAT‑KRPSGGE‑‑RRK‑‑YLSNWKIALTEAAN‑ISGW‑‑‑DI‑N‑‑‑‑KSNEGELMPLII

>G7J7L4

GKDTRGSRAS‑‑‑‑‑‑‑‑‑‑‑‑‑‑‑‑‑‑‑‑‑‑‑KGRWHSDIIKTNNQKSQISIIVFSKNYVESSWCMDELLEIMECH‑KTIGQV‑VLPVFYNVDPSEVRHQIGEFGIAFQNL‑EHK‑‑‑‑‑‑RLEN‑QEQAWRLALREAAG‑LVGF‑‑‑VV‑‑‑‑‑LNSNESEVIKDIV

>G7LCP7

GEDTRTNFTSFLHAALCKNHIET‑YID‑YRIE‑KGEEVWEELERAIKASALFLVVFSENYASSTWCLNELVEIMKCK‑KNE‑NVVVIPVFYRIEPSHVRKQTGSYHTALAKQ‑KKQGK‑D‑‑‑‑‑‑‑KIQRWKNALFEVAN‑LSGF‑‑‑DS‑‑‑‑‑‑‑‑TESDLIGDII

>G7KJR2

GGDTRYGFTGNLNRALCDKGIRT‑FMDDRELQ‑GGEEITSSLFKAIEESRIFIPVLSINYASSSFCLDELVHIIHCF‑KESGRL‑VLPIFYDVEPSHVRHHKGSYGKALDDH‑IERFQNNKHSMD‑‑RLQKWKIALTQTAN‑FSGH‑‑‑QI‑NP‑R‑NGYEYEFIEKIV

>G7LDV9

GEDTRAGFTSHLYETFLQSKFHT‑YID‑YRIQ‑KGDHVWAELTKAIKQSTIFLVVFSKNYASSTWCLNELVEIMECS‑NK‑‑NVAVIPVFYHIDPSRVRKQTGSYGTALAKH‑KKQGC‑D‑‑‑‑‑‑‑HMQNWKNALFQAAN‑LSGF‑‑‑HS‑‑‑‑‑‑‑‑TESDLIEDIT

>G7KK77

GSDTRFGFTGNLYKALRDCGIHT‑FIDDRELQ‑GGDEISPSLVKAIEESRIFIPVFSINYASSSFCLDELVHIIDCF‑NTKGCL‑VLPVFYGVDPSHIRHQTECFGEAIAKQ‑EVKFQNQKDDMD‑‑RLLKWKCALNKAAN‑FSGH‑‑‑HF‑NF‑G‑NEYEYEIITKIV

>G7KIH7

GTDTRFGFTGNLYKALSDKGIRT‑FIDDKELQ‑KGDEITPSLLKRIEESRIAIIVFSKEYASSSFCLDELVHIIHYF‑KEKGRL‑VLPVFYDVEPSHVRHQNYSYGEALAKH‑EERFQKSKKNME‑‑RLLKWKIALNKVAD‑LSGY‑‑‑HF‑NL‑G‑NEYERDFIEKIV

>G7K8D5

GEDTRYTFTSHLHATLTRLDVGT‑YID‑YHLQ‑RGDEISSALLRAIEEASLSVVVFSKNYGNSKWCLDELVKILECK‑KMRGQI‑VLPIFYDIEPSDVRNQTGSYADAFVKH‑EERFHGN‑‑‑LE‑‑RVQKWREALREAAN‑LSGW‑‑‑DC‑S‑‑‑TNRMESELLEKIA

>G7KP09

GEDTRNNFTDFLFDALETKGIFA‑FRDDTNLQ‑KGESIEPELLRAIEGSRVFVAVFSRNYASSTWCLQELEKICKC‑VQRSRK‑‑ILPVFYDVDPSVVRKQSGIYCEAFVKH‑EQRFQ‑‑‑QDFE‑‑MVSRWREALKHVGS‑ISGW‑‑‑DL‑R‑‑‑‑DKPQAGVIKKIV

>G7I276

GGDTREGFIGHLYKALTDKGIHT‑FIDDRELQ‑RGDEIKPSLDNAIEESRIFIPVFSINYASSSFCLDELVHIIHCY‑KKKGRL‑ILPVFYGVDPTHIRHQSGSYGEHLTKH‑EESFQNSKKNME‑‑RLHQWKLALTQASN‑LSGY‑‑‑HS‑S‑‑‑‑RGYEYKFIGEIV

>G7LI81

GEDTRTNFTAQLHQALSDRSIES‑YI‑DYNLV‑KGDEVGPALTKAIDDSHMSLVVFSKDYATSKWCLDELVHILQCR‑KLNGHV‑VIPVFYNIDPSHVRHQKESYQMAFARF‑ERK‑‑‑S‑‑HVD‑‑KVSEWKAALNLAAN‑ISGW‑‑‑DS‑R‑‑‑‑‑RDDTQVIGNIV

>G7KIE7

GSDTRYGFTGNLYKALTDKGIHT‑FIDDRELQ‑RGDEIKPSLDNAIEESRIFIPVFSPNYAYSSFCLDELVHIIHCY‑KTKGRL‑VLPVFYGVDPTHIRHQTGSYGEALAKH‑AKRFHNNNTNME‑‑RLQKWKIALAQAAN‑LSGD‑‑‑HR‑H‑‑‑‑PGYEYDFIEKIV

>G7IW61

GKDTRNNFTGNLYNSLNQRGIQT‑FMDDEEIQ‑KGEEITPTLLQAIEESRIFIAIFSPNYASSTFCLTELVTILECS‑MSQGRL‑FSPVFYDVDPSQIRYLTGTYAEAFKKH‑EERFGDD‑‑K‑H‑‑KMQKWRDALHQAAN‑MSGW‑‑‑HF‑KP‑G‑YELEYKFIEKIV

>G7K8D0

GEDTRNNFTSHLNGALKRLDIRT‑YID‑NDLN‑RGDEIPTTLIRAIEEAKVSVIVFSKNYAVSKWCLEELMKILEIK‑KMKGQI‑VVPIFYDVDPSDVRNQRGSYAEAFNNH‑ERNFKKK‑‑‑I‑‑‑KVQEWRNGLMEAAN‑YAGW‑‑‑DC‑N‑‑‑VNRTELALVEEIA

>G7KDY8

GEDTRNGFTGNLYKALCGKGINT‑FIDDKNLG‑KGEEITPALMMAIQESRIAIVIFSENYASSTFCLKELTKIMECI‑KHKGRL‑VLPIFYQVDPADVRHQKGSYANALASH‑ERKK‑‑‑TIDKI‑‑MVKQWRLALQEAAS‑ILGW‑‑‑HF‑EH‑‑‑‑GYEYELIGKIV

>G7K8G0

‑TQTRFNFHRHLHTTLSQKSI‑‑‑‑‑SSK‑‑T‑RSR‑ITP‑‑‑‑PI‑‑NSVNVTIHSENHPIAKMGLNNIATVSDVNFES‑‑R‑‑‑‑‑‑FFQFDPSNAEK‑‑GSF‑REFEET‑DAK‑‑‑‑‑‑‑‑DSN‑IDT‑SASLKNV‑‑‑I‑‑‑‑‑‑H‑‑‑‑‑‑‑N‑Y‑‑‑TVENVV

>G7JKN6

GEDIRHGFLGHLAKAFSRKQINA‑FVDD‑KLK‑RGDDISNSLVEAIEGSFISLIIFSENYASSSWCLEELLKIIDCK‑EKYGQI‑VIPVFYGVDPTNVRHLKKSYGNAFAEL‑EKRHSSL‑‑‑‑‑‑‑KVQIWRYALNKSAN‑LSGI‑‑‑KS‑‑‑‑‑‑‑‑NDAELLEEII

>G7ZVE9

GEDTRAGFTSHLHAALSRTYLHT‑YID‑YRIE‑KGDEVWPELEKAIKQSTLFLVVFSENYASSTWCLNELVELMECR‑NKEDNIGVIPVFYHVDPSHVRKQTGSYGSALAKH‑KQENQ‑D‑‑‑‑‑‑‑DMQNWKNALFQAAN‑LSGF‑‑‑HS‑‑‑‑‑‑‑‑TESNMIEDIT

>G7KJN1

GSDTRYGFTGNLYKALTDKGINT‑FIDKNGLQ‑RGDEITPSLLKAIEESRIFIPVFSINYASSSFCLDELVHIIHCY‑KTKGRL‑VLPVFFGVEPTVVRHRKGSYGEALAEH‑EKRFQNDPKNME‑‑RLQGWKKALSQAAN‑LSGY‑‑‑HD‑SP‑‑‑PGYEYKLIGKIV

>G7LI62

GEDTRYGITNLIYDALIHKSIKT‑FI‑DYELN‑RGEDVWPKLSKAIEESHISVVVFSENFATSKWCLEELVKVLECR‑KDHGQV‑VIPVFYKTNPSHIRNQTHSYEKAFAKH‑ERK‑‑‑S‑‑NASN‑IVLKWRSALTEAAT‑ISGW‑‑‑DT‑H‑‑‑‑‑KDESNLIHKIV

>G7JUR1

GEDTRSNFTSHLHAALCRTKVKT‑YID‑‑NLK‑KGDYISETLVKAIQDSYVSIVVFSENYASSTWCLDELTHMMKC‑LKNN‑QIVVVPVFYNVDPSHVRKQSGSYMVAFEKHC‑‑NLNHN‑‑‑‑‑‑‑KVNDWREALAQATS‑LAGW‑‑‑DS‑R‑‑‑‑‑KLESELVEDIV

>G7J6M1

GEDTGRKFTSHLYEALSKK‑IIT‑FIDDNELE‑KGDEISSALIKAIEDSSASIVIFSKDYASSKWCLNELVKILECK‑KDQGQI‑VIPIFYEIDPSHVRNQNGSYGQAFAKH‑ARDLKQN‑‑‑KE‑‑MLKKWKDALTEAAN‑LAGW‑‑‑HS‑QN‑‑‑‑‑IESNFIKDIV

>G7KPI2

GEDTRNNFTDYLFDALETKGIYA‑FRDDTNLK‑KGEVIGPELLRAIEGSQVFVAVFSRNYASSTWCLQELEKICEC‑VQGPEK‑‑VLPVFYDIDPSEVRKQSGIYCESFVKH‑EQRFQ‑‑‑QDPH‑‑KVSRWREALNQVGS‑ISGW‑‑‑DL‑R‑‑‑‑DKPQAGEIKKIV

>G7IW59

GIDTRNNFTGNLYNSLNQRGIRT‑FFDDEEIQ‑KGEEITPTLLQAIKESRIFIVVFSTNYASSTFCLTELVTILGCS‑KSQGRI‑FLPIFYDVDPSQIRNLTGTYAEAFAKH‑EMRFGDE‑‑E‑D‑‑KVQKWRDALRQAAN‑MSGW‑‑‑HF‑KP‑G‑SESEYKFIGKIV

>G7LGU3

GEDTRLGFTGFLYKTLSEKGFHT‑FIDH‑HAD‑AGRGTTKTLVDAIEESRIGIVVFSENYASSTWCLDELAYIIDSFKKNFRRS‑VFPVFYNVDPSHVRHQSGIYGQALDSH‑QKNN‑‑‑NFNSE‑‑KLNKWKNALKQAAN‑LSGF‑‑‑HF‑KH‑G‑DGYEYELIDKIV

>G7KIG1

GTDTRFSFTGNLYKALSDNGIRT‑FIDDKDLQ‑SGDEITPSLLKNIEDSRISILVFSENYATSSFCLDELVHIIHCS‑KEKGSM‑VIPVFYGIEPSHVRHQNSSYGEALAKH‑EEVFQNNKESME‑‑RLRKWKKALNHAAN‑LSGH‑‑‑HF‑NF‑G‑NEYEHHFIGKIV

>G7LHH8

GQDTRETFTSHLHYALCKENIIT‑YID‑‑NLV‑KGDEIGEALAEAIQDSRISLVVFSKNYATSKWCLNELLKILECK‑KLHGQV‑VIPVFYNTGTSEVRNQTGSY‑KPFSHY‑EEN‑‑‑N‑‑‑‑E‑‑TVSEWRAALAEAAN‑IPGW‑‑‑DSSRTY‑‑K‑DDSQVIQSIV

>G7KHT0

GSDTRNKFTGNLYKALVDKGIRT‑FIDDNDLE‑RGDEITPSLVKAIEESRIFIPIFSANYASSSFCLDELVHIIHCY‑KTKSCL‑VFPVFYDVEPTHIRNQSGIYGEHLTKH‑EERFQNNEKNME‑‑RLRQWKIALIQAAN‑LSGY‑‑‑HY‑SP‑‑‑HGYEYKFIEKIV

>G7JLX1

GEDTRASFISHLTSSLQNAGILI‑FKDDQSLQ‑RGDHISPSLVHAIESSKISVIVFSKNYADSKWCLQELWQIMVRH‑RTTGQV‑VLPVFYDVDPSEVRHQTGEFGKSFLNL‑EKW‑‑‑‑‑‑‑‑‑‑‑‑ALEWRNELRVAAG‑LAGF‑‑‑VV‑‑‑‑‑LNSNESEVIKDIV

>G7LF48

GEDVRRTFVSHLYAVLSNAGINT‑FLDNEKLE‑KGEDIGHELLQAISVSRISIIVFSKNYTESSWCLNELEKIMECR‑RLHGHV‑VLPVFYDVDPSVVRHQKGDFGKALEVA‑KSYIIEE‑‑VMV‑‑KLGKWRKVLTEASN‑LSGW‑‑‑DG‑S‑‑‑‑ARSDRELVKKIV

>G7INY1

GTDTRYGFTGNLYRALSDGGFCT‑FIDDRELH‑GGDEITQSLVKAIEESMIFIPVFSINYASSIFCLDELVHIIHCF‑QEKGRK‑ILPIFYDVEPSHVRHQTGSYGKAIARH‑EKRFQNNKENMK‑‑RLHKWKMALNQAAN‑LSGH‑‑‑HF‑NP‑R‑NEYQYKFIGDIV

>G7KIF8

GADTRYEFTGNLYKALTDKGIHT‑FFDDRELQ‑RGDKIEQSLNNAIEESRIFIPVFSANYASSSFCLDELVHIIRVY‑KEKGRL‑VLPVFYGVDPGDIRHQRGSYAIHLTKH‑EKRFGNNKENME‑‑KLLQWKKALKQAAD‑LSGF‑‑‑HF‑SG‑‑‑NGYEYKRIGEII

>G7KP15

GDDTRNNFTGYLLDALKTNGIYA‑FRDDTNLQ‑KGESIGPELLRAIEGSQVFVAVFSRNYASSTWCLQELEKICEC‑VHVSRK‑‑ILPVFYDVDPSEVRKQSGIYGEAFTIH‑EQTFQ‑‑‑QDSQ‑‑MVSRWREALKQVGS‑IAGW‑‑‑DL‑C‑‑‑‑DKPQSAEIRMIV

>G7INJ8

GDDTRRKFTSHLNEALKKSGLKT‑FIDDNELK‑KGDEISSALIKAIEESCASIVILSENYASSKWCLNELVKILECK‑KDNGQI‑VIPIFYEIDPSHVRYQIGSYGQAFAKY‑EKNLRHK‑‑‑KD‑‑NLQKWKDALTEVSK‑LSGW‑‑‑DS‑KN‑‑‑‑‑IESDFIKDIV

>G7KHV7

GADTRS‑‑‑‑‑‑‑RASPAISIKL‑FV‑‑‑‑‑‑‑‑TKESEPSLMREVMKSHL‑‑‑‑‑‑‑HYASSSFCLDELVH‑‑‑‑‑‑‑ENSRL‑VLPVFYDVEPSEVRHYNNRYGEALTEF‑EERFQNNKENME‑‑RLQKWKIALNQAYN‑LSGY‑‑‑HF‑KE‑‑‑DEYEYEFIKKIV

>G7JF22

GDDTRNKFTDHLFGALRRKNIAA‑FRDNRHLN‑SGASIEPALFRAIEVSQIFIVVLSKSYASSTWCLRELVYILHC‑SQPSEK‑‑VRTVFYDVNPSEVRKQSGSYAKAFAKH‑EENFG‑‑‑QDHV‑‑KVRQWREALTQAGN‑ISGC‑‑‑DL‑G‑‑‑‑NKPENEEIETIV

>G7K9Q6

GDDTRFHFTGNLYKALCDKGIRV‑FIDDKELQ‑RGDKITPSLIKAIEDSRIAIPVFSKNYAFSSFCLDELVNIIDGF‑SAKGRL‑VLPVFYDVDPSHVRHQIGSYGEAIAMH‑EARLKRDKENMD‑‑RLQKWKTALNQAAN‑LSGY‑‑‑HF‑NH‑G‑NEYEHEFIGRIM

>G7KSB9

GPDTRNTFVDHLYAHLTRKGIST‑FKDDKSLQ‑KGESISLQLLQAIKDSRVSIIVFSKDYASSTWCLDEMAAIDESS‑RRK‑LV‑VFPVFYDIDPSHVRKRSGAYEDAFVLH‑NELFKH‑‑‑DPD‑‑RVAQWRRAMTSLAG‑SAGW‑‑‑DV‑R‑‑‑‑NKPEFDEIEKIV

>G7JKN3

GEDIRHGFLGHLIKAFPRKQINA‑FVDE‑KLK‑RGDDISHALVEAIEGSFISLVIFSENYASSHWCLEELVKIIECK‑EKYGRI‑VLPVFYGVDPTNVRHQKKSYKSAFSEL‑EKRYHLS‑‑‑‑‑‑‑KVQNWRHALNKSAN‑LSGI‑‑‑KS‑‑‑‑‑‑‑‑NDAELLEEII

>G7JSB1

NKDAG‑SFALQLYTALSSEA‑SV‑FWDNARLG‑SGDQIS‑SAVNIIGNCRVAVIIFSMKYFNSMWCLQEFEKITECQ‑RVTDGT‑VLPVFFDAYPCNGRLHKNMFGDPFDYY‑VDRVTET‑‑KED‑‑IFMSWVAAVSDKAS‑ASRY‑‑‑SLRK‑‑‑‑SSNEGEYIKDVV

>G7K8C8

GEDTRNNFTSHLNGALKRLDIRT‑YID‑NDLN‑SGDEIPTTLVRAIEEAKLSVIVFSKNYAVSKWCLEELMKILEIK‑KMKGQI‑VVPVFYDVDPSDVRNQRGSYAEAFAKH‑ENNFEGK‑‑‑I‑‑‑KVQEWRNGLLEAAN‑YAGW‑‑‑DC‑N‑‑‑VNRTELALVEEIA

>G7LF46

GKDTRKTFVSHLYAALTDAGINT‑FLDDENLK‑KGEELGPELVRAIQGSQIAIVVFSKNYVNSSWCLNELEQIMKCK‑ADNGQV‑VMPVFNGITPSNIRQHS‑‑‑‑‑‑‑‑‑P‑IL‑‑‑VD‑‑ELD‑‑QIFGKKRALRDVSY‑LTGW‑‑‑DM‑S‑‑‑‑NSNQSKVVKEIV

>G7KJ27

GTDTRYGFTGNLLKALIDKGIRT‑FHDDDDLQ‑RRDKVTPII‑‑‑IEESRILIPIFSANYASSSSCLDTLVHIIHCY‑KTKGCL‑VLPVFFGVEPTDVRHHTGRYGKALAEH‑ENRFQNDTKNME‑‑RLQQWKVALSLAAN‑LPSY‑‑‑HD‑DS‑‑‑HGYEYELIGKIV

>G7IW72

GIDTRNNFTGNLYHSLHQRGIQT‑FMDDEEIQ‑KGEEITPTLLQAIKQSRIFIAIFSPNYASSTFCLTELVTILECS‑MLQGRL‑FLPVFYDVDPSQIRNLTGTYAEAFAKH‑EVRFGDE‑‑KDS‑‑KVQKWRDALRQAAN‑VSGW‑‑‑HF‑KP‑G‑FESEYKIIEKIV

>G7IM67

GADTRSTFVDHLHAHLTTKGIFA‑FKDDKRLE‑KGESLSPQLLQAIQSSRISIVVFSKNYAESTLCLEEMATIAEYH‑TEK‑QT‑VFPIFYDADPSHVRKQSGVYQNAFVLL‑QNKFKH‑‑‑DPN‑‑KVMRWVGAMESLAK‑LVGW‑‑‑DV‑R‑‑‑‑NKPEFREIKNIV

>G7IIT2

GHDTRVGFTSHLESALCRNYFLT‑YTD‑YRIK‑SGKKIWDELVKAMNESTLFLVVFSENYADSSWCLDELVEIMKCR‑RK‑‑QVVVLPVFYRIEPSYVRKQTGSYGAALARH‑SQGHRDS‑‑‑‑‑‑‑HIQLWKDALREAGN‑LSGF‑‑‑HV‑‑‑‑‑‑‑‑SESAWIEDIN

>G7LGU7

GLDTRRGFSGFLNKYLTDRG‑RT‑FFDDGEIE‑IGTQIT‑RIHKGIEDSRIFIPILSENYASSSFCLDELVKILAEQ‑KGNGR‑‑VFPVFYYVSISDVKNQTGSYGQALTVL‑KNG‑‑‑‑‑‑‑EE‑‑RFEKWISALTSIAD‑FRGC‑‑‑HMER‑‑‑‑AGYEFQYIYEII

>G7JY83

GEDTRNTIVSHLYAALQNSGVYT‑FLDDQKLT‑KGEVLGPALRKAIEESKIFIVVLSPDYAGSSWCLRELVHIMDCH‑ESYGRI‑VLPVFYGVEPSEVRKQSGDFGKALKLT‑TK‑‑‑‑‑‑‑RED‑‑QLSMWKTALTKVGN‑LAGW‑‑‑DY‑N‑‑‑‑IRNEGELVELIV

>G7KJR6

GIDTRYGFTGNLYSDLCKKGIHT‑FFDDRELQ‑GGDEITSSLFKVIEESRIFIPVLSINYASSSFCLDELVHIIHCF‑KENRRL‑VLPIFYDVEPSHVRHHKGSYGKALDDH‑IERFQNNKHSMD‑‑RLQKWKMALTQTAN‑FSGH‑‑‑QI‑NP‑R‑NGYECEFIEKIV

>G7K3B3

GEDVRHNFIGYLRDALQHRGINA‑FFDDKNL‑‑RGEDISPALSKAIEESKIAVIVFSENYASSRWCLGELVKIIECK‑RNKKQI‑SFPIFFHVDPSDVRHQKNSYEKAMVDH‑EVKFGKS‑‑‑‑E‑‑NVKAWITALSEAAD‑LKGH‑‑‑H‑IN‑‑‑‑T‑SEIDHIKEIV

>G7JF20

GEDTRNNFTDFLFDALEEKGVFA‑FRDDTNLQ‑KGESIAPELFHAIEGSQVFVVVLSKNYAFSTWCLKELEYILCC‑VQASKK‑‑VLPVFYDVDPSLVRKQTGIYSEAFVQH‑GHRFK‑‑‑QDSQ‑‑MVLRWRAALTQVAD‑LSGW‑‑‑DL‑R‑‑‑‑DKRQSLEIKKIV

>G7KL74

GEDTRHGFTGYLKKALDDKGVRT‑FMDDKELR‑KGEEITPSLLKAIEQSMMAIVVLSENYASSSFCLQELSKILDTM‑KDMGRS‑VFPVFYKVDPSDVRKLKRSFGEGMDK‑‑‑HKA‑‑‑NSNLD‑‑‑‑‑KWKVSLHQVTD‑LSGF‑‑‑HY‑K‑‑G‑DTPEHMFIGDIV

>G7L6T3

GEDTRTSLVSHMDAALTNAGINT‑YID‑QQLH‑KGTELGPELLRAIEGSHISILVFSKRYTESSWCLNELKKVMECH‑RTHGQV‑VVPIFYDVDPSVVRQQKGAFG‑‑‑‑‑‑‑‑‑‑‑‑‑E‑‑ILK‑‑YLSRWTSALTQAAN‑LSGW‑‑‑DV‑T‑‑‑‑NRSEAELVQQIV

>G7LDU5

GEDTRECFTKKLYESLHKQGVRA‑FMDDEGLD‑RGDHIATTLLEAIDDSAASIVIISPNYADSHWCLDELNRICDLE‑‑‑‑‑RL‑IIPVFYKVDPSHVRKQLGPF‑DGFNYL‑EKRFAN‑‑‑EKD‑‑KILKWRDSMLKIGG‑LAGF‑‑‑‑VFNS‑S‑DGDHENLIRRLV

>G7KDY7

GEDTRKGFTGNLYKALCGKGIDT‑FIDDQELR‑KGEEITPALMMAIQESRIAIVIFSENYASSTFCLKELTKIMECI‑KHKGRM‑VLPVFYHVDPCIVRHQKGSYAKALADH‑ESNK‑‑‑KIDKA‑‑KVKQWRLVLQEAAS‑ISGW‑‑‑HF‑EH‑‑‑‑GYEYEFIEKII

>G7LDL6

GDDTRAGFTSHLHAALCRSNFHT‑YID‑YRIE‑KGDEVWGELQKAINESTLFLVVFSENYAFSTWCLNELVQIMECS‑NNENNVVVIPVFYHVDPSHVRKQTGSYGTALAKH‑I‑‑‑‑‑D‑‑‑‑‑‑‑HLQNWKNALFEASN‑LSGF‑‑‑HS‑‑‑‑‑‑‑‑TESDLIEDII

>G7IM44

GPDTRNSFVDHLYAHLTRKGIFA‑FKDDKSLE‑KGEFISPQLLQAIRNSRIFIVVFSKTYAESTWCLEEMAAIADCC‑EYK‑QT‑VFPIFYDVDPSDVRKQSGVYQNDFVLH‑KKKFTR‑‑‑DPD‑‑KVVRWTKAMGRLAE‑LVGW‑‑‑DV‑R‑‑‑‑NKPEFREIENIV

>G7I3L5

GTDTRYTFTGHLYKALHNKGIMT‑FIDDDHLQ‑KGDQITPSLLKAIENSRIAIVVLSKNYASSSFCLQELCKILEN‑‑‑‑‑GGL‑VWPVFYEVEPSNVRKLSGSFGEAMAVH‑EVRY‑‑‑SDDVD‑‑RLEKWKKGLYQVAN‑LAGF‑‑‑HY‑K‑‑NGDGYEHEFIGKIV

>G7KL67

GEDTRYGFTSYLKKALDDKGVRT‑FMDDEELQ‑KGEEITPSLLKAIEDSQIAIVVLSKNYASSSFCLQELSHILHSI‑KDKGRS‑VLPVFYKVDPSVIRKLEKSYGEAMDK‑‑‑HKA‑‑‑NSNLD‑‑‑‑‑KWKVCLHQVAD‑LSGF‑‑‑HY‑K‑‑KRDMPEHKFIGEIV

>G7IIM2

GEDTRNNFTDFLFDALERKDIFA‑FRDDTNLQ‑KGESIGSELLRAIEGSQVFVAVFSRYYASSTWCLEELEKICEC‑VQVPGK‑‑VLPVFYDVDPSEVRKQSGIYHEAFVKH‑EQRFQ‑‑‑QDLQ‑‑KVSRWREALKQVGS‑IAGW‑‑‑DL‑R‑‑‑‑DKPQCAEIKKIV

>G7KPI1

GEDTRNNFTDFLFDALETKGIFA‑FRDDTNLQ‑QGESLEPELLRAIKGFQVFVVVFSRNYASSTWCLKELEKICEC‑VKGSKK‑‑VIPVFYDVDPSEVRKQSGIYCEAFVKH‑EKRFQ‑‑‑QGFE‑‑MVSRWREALKQVGS‑ISGW‑‑‑DL‑C‑‑‑‑DKPQAGEIKKIV

>G7LCP9

GEDTRTNFTSFLHAALCKNHIET‑YID‑YRIE‑KGEEVWEELEKAIKASALFLVVFSENYASSTWCLNELVEIMKCK‑KNE‑NVVVIPVFYRIEASHVRKQTGSYHTALLKQ‑KKQGK‑D‑‑‑‑‑‑‑KIQRWKIALFEVAN‑LSGF‑‑‑DS‑‑‑‑‑‑‑‑TEADLIGDII

>G7JKN5

GKDIRGDFLSHLIEALRRKKIKA‑FVDD‑ELK‑RGDEILQSLVRGIEGSLISLIIFSQDYASSRWCLEELVTILQCR‑EKYGQI‑VVPIFYGIDPADVRYQMKSYENAFVEH‑QRVYSST‑‑‑‑‑‑‑KVQIWRHALNKSAN‑LSGI‑‑‑KS‑‑‑‑‑‑‑‑NDVQLLKEIV

>G7LIZ9

GEDTRNNFTDHLFGALHKNRIVV‑FRDDINLK‑KGGNISSELLQAIKESHILIVIFSKNYASSTWCLQELVNIADC‑IHVQGQ‑‑VLPIFYDVSPSEVRKQTGDYEKPFLEH‑GERFK‑‑‑GNLE‑‑AVQRWRGALTQVAN‑LSGW‑‑‑DI‑K‑‑‑‑DKSQYAEIEKII

>G7JY79

GEDTRNTIVSHLHAALQNSGVNT‑FLDDQKLK‑KGEELEPALRMAIEQSKISIVVLSPNYAGSSWCLDELVHIMDCR‑ESYGRT‑VVPVFYRVNPTQVRHQTGDFGKALELT‑TK‑‑‑‑‑‑‑KED‑‑QLSKWKRALTEVSN‑ISGW‑‑‑RY‑N‑‑‑‑IRNEGELVKGIV

>G7KKS7

GEDTRHGFTGNLWKALSDRGIHT‑FMDDEELQ‑KGEEITPSLIKAIEDSNMAIIVLSKNYASSTFCLKELSTILYSI‑KDKGRC‑VWPVFYDVEPSDVRKLKRSYGEAMVEH‑EARD‑‑‑HSNMD‑‑LLQKWKNALNQVAN‑LSGF‑‑‑HF‑K‑‑NGDEYEHVFIGKIV

>G7LI80

GEDTRTNFTAQLHRALTDSSIES‑YI‑DYSLV‑KGDEVGPALAKAIQDSHMSLVVFSENYATSKWCLDELLHILQCR‑KHHGQV‑VIPVFYNIDPSHVRHQKESYEMAFARY‑DRK‑‑‑S‑‑QLD‑‑KVSEWKAALKLAAN‑ISGW‑‑‑DS‑R‑‑‑‑‑RDDSQVIDKIV

>G7JP38

GTDTRYGFTGNLYEALRVKGIHT‑FIDDRELQ‑RGDQITPSLLKAIQESKIVIIVFSNHYASSSFCLDELVHIIHCS‑KENGCL‑VLPIFYGVEPSHVRYQTGSYGEALAEH‑EEARKKEKYNME‑‑KLQKWEMALKQAAN‑LSGY‑‑‑HF‑NA‑R‑TGYEYEFIQMIV

>G7IQA0

GIDTRNNFTRDLYDILYQNGIHT‑FFDEEQIQ‑KGEEITPALFQAIQQSRIFIVVFSNNYASSTFCLNELVVILDCS‑NTHGRL‑LLPVFYDVDPSQVRHQSGAYGEALGKH‑EKRFCDD‑‑K‑D‑‑KVQKWRDALCQAAN‑VSGW‑‑‑HF‑QH‑G‑SQSEYKFIGNIV

>G7KKT7

GEDTRIGFVNNLRKALNDKGIKT‑FIDEH‑‑Q‑K‑E‑ITPAILNVIRECKIVIIVLSNNYAAS‑‑‑‑‑‑‑‑SFL‑‑‑‑‑‑‑‑‑‑‑‑‑‑‑FYNIEPCTVRKETGSYG‑ALENH‑E‑R‑‑‑D‑‑N‑K‑‑KTRKWRGALTEVSN‑LSGW‑‑‑Q‑FKH‑G‑‑GNEHKIISNIV

>G7KIG4

GSDTRYGFTGNLYKALTDKGIHT‑FMDDRELQ‑RGDEIKRSLDNAIEESRIFIPVFSANYASSSFCLDELVQIINC‑‑KEKGRV‑VLPVFYGMDPTNVRHHRGIYGEALAKH‑EKRFQNDMDNME‑‑RLQRWKVALNQAAN‑LSGY‑‑‑HF‑S‑‑‑‑PGYEYEFIGKIV

>G7JVS3

G‑GTRYSFTDHLYRSLLRQGINV‑FRDDQNL‑‑KGHEIGPSLLQAIEASRISIVVLCKEYASSTWCLDELVKIVDCY‑ENNGKS‑KNSYEDAIR‑‑‑‑‑‑‑‑‑‑‑‑‑‑‑‑KH‑EKRFGRS‑‑‑‑E‑‑KVKAWKLALNRVCA‑LSGL‑‑‑H‑CK‑‑‑‑D‑YESEFIEKIV

>G7KMZ4

GSDTRYGFTGNLYKALTDKGIHT‑FIDDSELQ‑RGDEITPSLDNAIEESRIFIPVFSANYASSSFCLDELVHIIHLY‑KQNGRL‑VLPVFFGVDPSHVRHHRGSYGEALAKH‑EERFQHNTDHME‑‑RLQKWKIALTQAAN‑LSGD‑‑‑HR‑S‑‑‑‑PGYEYDFIGDIV

>G7IN46

GDDTRRKFTSHLNEALKKSGVKT‑FIDDSELK‑KGDEISSALIKAIEESCASIVIFSEDYASSKWCLNELVKILECK‑KDNGQI‑VIPIFYEIDPSHVRNQIGSYGQAFAKH‑EKNLKQ‑‑‑‑‑‑‑‑‑‑QKWKDALTEVSN‑LSGW‑‑‑DS‑KS‑‑‑‑‑IESDFIKDIV

>G7J1L8

GEDTRKSLVSHLYAALSNAGINT‑FLDDEKLK‑KGWEVEPELLRAIQGSQICLVIFSEHYTESSWCLVELVKIMEHR‑KTNNNV‑VIPIFYHVDPSVVRRQVGDFGKALEAI‑KRHPPKE‑‑‑RQ‑‑ELRTWKRALTQAAN‑ISGW‑‑‑DS‑S‑‑‑‑IRSESELVNKIV

>G7KJ53

GSDTRYGFTGNLYKALTDKGINT‑FIDDNGLQ‑RGNEITPSLLKAIEESRIFIPVFSINYASSSFCLDELDHIIHCY‑KTKGRP‑VLPVFFGVDPSHVRHHKGSYGEALAEH‑EKRFQNDPKNME‑‑RLQGWKDALSQAAN‑LSGY‑‑‑HD‑SP‑‑‑PGYEYKLIGKIV

>G7JF23

GEDTRFNFTDHLFAALQRKGIFA‑FRDDTKLQ‑KGESIAPELIRAIEGSQVFIAVLSKNYASSTWCLRELEYILHY‑SQVFGR‑‑VLPVFYDVDPSEVRHQKGIYGEAFSKH‑EQTFQ‑‑‑HDSH‑‑VVQRWREALTQVGN‑ISGW‑‑‑DL‑R‑‑‑‑DKPQYEEIKKIV

>G7L6L9

GEDTRKGFTDHLCAALERKGITT‑FRDDKDLE‑RGQVISEKLINAIKDSMFAITVLSPDYASSTWCLDELQMIMEC‑‑SNKGE‑‑VLPVFYGVDPSDVRHQRGCFEESFRKH‑LEKFGQH‑‑‑SD‑‑RVDRWRDAFTQVAS‑YSGW‑‑‑DS‑‑‑‑‑KG‑HEALLVESIA

>G7L9E6

GKDTRNDFVSHLNAALQNRGIKT‑FLDDEKLG‑KGEKLGPQLEKAIEGSLISIVVLSPDYAESSWCLNELVHILKCQ‑KTYGQV‑VMPVFYHVDPSVVRKQTGDFGKALELT‑TK‑‑‑‑‑‑‑KED‑‑KLSNWKTALKQVAT‑IAGW‑‑‑DC‑Y‑‑‑‑NRNKGELAKQIV

>G7KJ57

GTDTRHGFTGNLYKALTDKGIHT‑FIDDNDLP‑RGDEITPSLLKAIDESRIFIPVFSINYASSSFCLDELVHIIHCY‑KTKGRL‑VLPVFFGVEPTKVRHQKGSYGEALAEH‑EKRFQNDKNNME‑‑RLQGWKVALSQAAN‑FSGY‑‑‑HD‑SP‑‑‑PGYEYEFTGEIV

>G7KHU9

GSDTRNTFTGNLYKALVDKGIRT‑FFDDNDLQ‑RGDEITPSLVKAIEESRIFIPIFSANYASSSFCLDELVHIIHCY‑KTKSCL‑VLPVFYDVEPTHIRHQSGSYGEYLTKH‑EERFQNNEKNME‑‑RLRQWKIALTQAAN‑LSGY‑‑‑HY‑SP‑‑‑HGYEYKFIEKIV

>G7LI71

GEDTRKNFTGKLHEALKKENIET‑YID‑‑YVK‑VGDEVGPMLIQAIHESQISVIVFSKNFVTSKWCLEELLHILECR‑KHHGQV‑VLPFYYETDPSNIVGGKGSY‑KAFARY‑EEN‑‑‑N‑‑‑‑QC‑KVSKWKAALVEVAA‑ISAR‑‑‑D‑SRHY‑‑S‑DDSQFIQCIV

>G7LF47

GEDTRKTFVSHLYAALTNAAIRT‑FRDDKELR‑KGNKLEPEIKRAIEGSRISIVVLSPYYAGSSWCLNELVHILHCS‑HTYGQV‑VMPVFYHVDPSHVRKLEGNFGTIFELH‑IH‑‑‑‑‑‑‑REH‑‑ELSKWKTVLTEVSN‑LSGW‑‑‑DL‑N‑‑‑‑NSNEGELVKQIV

>D7SMZ2

GEDTRYKFTDHLYAALVNKGIRT‑FRDDK‑LK‑RGEEIAPLLLKVIEESRLSIVVFSENYASSRWCLDELVKIMECR‑QKIRQI‑LVPIFYHVDPSDLRTQKGSFEKSFASH‑ERHGRDS‑‑‑KE‑‑KIQRWRAALTEASN‑LSGW‑‑‑HL‑F‑‑‑‑EGYESEHIKK‑‑

>D7UDZ7

GEDTRNNFTAHLYHALCQKGIYT‑FIDDDKLE‑RGEVISSALVEAIENSMFSIIVLSENYASSRWCLEELVKILECK‑ENKGQ‑‑VLPIFYHVDPADVRKQRGKFGEALAKH‑KKE‑‑‑‑‑‑NME‑‑RVKIWKDALTKVAY‑LSGW‑‑‑DS‑Q‑‑‑‑NKNELLLIKEVA

>F6HJI7

GEDTRNNFTDHLYTDLVRKGIRI‑FRDDK‑LK‑RGEKIAPELLNAIEKSRSSIVVFSKSYADSRWCLDELAKIMECR‑REYGQK‑VLPIFYHVDPSDVRKQTGSFGEAFTMY‑EET‑‑‑‑‑‑‑KN‑‑KVRSWREALTEASN‑LSGW‑‑‑HV‑‑‑‑‑NEGYESEHIKKIT

>F6I7C7

GEDTRNNFTAHLYDALHCKGINA‑FIDADKLR‑IGEIISPALLSAIEGSRFSIVVLSENYASSRWCLEELVKILECK‑KTKGQV‑VLPIFYQVDPSDVRKQKGSYGKAFAKH‑EENMKEN‑‑‑ME‑‑KVHIWREALSEVGN‑ISGR‑‑‑DS‑R‑‑‑‑NKDESVLIKEIV

>F6GW26

GEDTRKSFTDHLYSALIRNNIHT‑FRDDEEL‑‑RGEEIAPELLKAIEESRSAIIVFSKTYAHSKWCLEELVKIMKCK‑EER‑QMVVIPIFYHVDPSELRNQT‑EYGEAFTHH‑E‑K‑‑‑N‑‑DEERKKIRKWKIALRQASN‑LAGY‑‑‑D‑AK‑‑‑‑DRYETELIDKII

>F6HMY4

GEDTGKTFTDHLYTALDENGFYA‑FRDDEKHE‑KREEIAPEFLTAIEESKISILVFSKNYASSRWCLDELETIIKSM‑KKPGRM‑VMPVFYHVDPSEVRDQIGS‑CEVFLSH‑E‑R‑‑‑D‑‑‑EET‑KVNRWRAALREASN‑LVGW‑‑‑RL‑H‑‑‑‑NQYESQLIKEII

>F6H7N9

GEDTRFNFTDHLYKKLRRTGIRT‑FRDDEGLE‑RGGEIQPSLLKAIEDSSISVVVFSKNYAHSKWCLDELDKIMQSM‑‑EKGQL‑VLPVFYHVDPSDVRKQRGSFGEAFAGY‑EVA‑‑‑‑‑‑‑EE‑‑RVQRWKAALTKAGS‑LAGW‑‑‑HV‑E‑‑‑‑HGYESQIIKGIV

>F6HN39

GDDTRNNFTAHLLQELRTKGINT‑FFDEDKLE‑KGRVISPALITAIENSMFSIIVLSENYASSRWCLEEMVKILECN‑RSKEER‑VLPIFYNVDPSDVRNHMGKFGEALAKH‑EENLEEN‑‑‑GE‑‑RVKIWRDALTEVAN‑LSGW‑‑‑DS‑R‑‑‑‑NKNEPLLIKEIV

>F6GYU1

GEDTRNNFTVHLFKILGRMGINT‑FRNDE‑PL‑RREEIQSGILKTIEESRISIVVFSRNYAHSQWCLDELAKIMECR‑KQNEQI‑VLPVFYHVDPSDVRKQTGSFGNAFSNY‑ERGVDE‑‑‑‑‑K‑‑KVQRWRDAFTEAAD‑TDGF‑‑‑RVPE‑‑‑‑DGDEPTIIKKII

>F6H8Z5

GEDTRKSFTDHLHEALHRCGINT‑FIDDQ‑LR‑RGEQISSALLQAIEESRFSIIIFSEHYASSSWCLDELTKILECV‑KVGGHT‑VFPVFYNVDPSHVRKQTGSYGVAFTKH‑EKVYRDN‑‑‑ME‑‑KVLKWREALTVASG‑LSGW‑‑‑DS‑R‑‑‑‑DRHESKVIKEIV

>D7SS75

GEDTRRNFTGHLYAALIRKGIVT‑FRDDEGLS‑RGEEIAPSLLTAIEKSRCALVILSEHYADSRWCLEELAKIMEWR‑EM‑GLI‑VYPVFYHVDPSHVRHQRGHYGEALADH‑E‑R‑‑‑N‑‑SGH‑‑Q‑QRWRAALTEVAN‑LSGW‑‑‑HA‑E‑‑‑‑NGSESEVVNDIT

>F6I433

‑‑DV‑‑AITAT‑‑RAA‑‑HGT‑‑‑‑‑‑‑‑‑‑‑‑‑G‑‑L‑PETLEYLRD‑‑‑AMVILKKNYAVSNWLLSE‑‑‑‑LEK‑‑‑‑‑‑E‑‑VMEILQKGD‑REIQ‑QKGTF‑KNYRSS‑‑‑‑‑‑‑‑‑‑‑‑‑‑‑KIEK‑‑‑‑‑‑‑IASTI‑GV‑‑‑E‑LR‑‑‑‑‑‑HDFSIVIRLR

>F6HN37

GEDTRKNFTDHLYTTLVAYGIHT‑FRDDEELE‑KGGDIASDLLRAIEESKIFIIIFSTNYANSRWCLNELVKIFECT‑QK‑‑QT‑ILPIFYHVNPSDVRKQSGSYGDAFVDH‑EKD‑‑‑DEKKME‑‑‑IQKWRTALNQVAS‑LCGL‑‑‑HV‑‑‑‑‑‑DEYETLVVKEIT

>F6HWC4

‑‑‑‑‑MNFVDHLYEGLVGNRFNT‑FRDDEQLE‑RGGEISSQLLDAIEESRICIVVFSKNYADSRWCLNELLAIIESISDD‑GRI‑VLPIFYHVDPSHVRHQTGSYCTRYTYP‑E‑R‑‑‑D‑‑DKE‑‑KIEKWGNALTAAAN‑MSGY‑‑‑HV‑D‑‑‑PKTHEGNIIEEIA

>F6H967

GEDTRKSFTDHLHKALRRCGIHA‑FIDDR‑LR‑RGEQISSALLRAIEESRFSIIIFSEHYASSSWCLDELTKILQCV‑KEGRHT‑AFPVFYNVDPSHVRKQEGSYGVAFTKH‑EQVYRDN‑‑‑ME‑‑KVVEWRKALTVASN‑LSGW‑‑‑DS‑R‑‑‑‑DKHESEVIKEIV

>F6HMY1

GEDTRNNFTDHLFVNLHRMGINT‑FRDDQ‑LE‑RGEEIKSELLKTIEESRISIVVFSKDYAQSKWCLDELAKIMECR‑EEMEQI‑VLPVFYHVDPSDVRKQTGSFGEAFSIH‑ERNVDE‑‑‑‑‑K‑‑KVQRWKDSLTKASN‑LSGF‑‑‑HV‑N‑‑‑‑DGYESKHIKEIV

>F6H8V9

GEDTRYNFTDHLYSALGRRGIHT‑FRDDK‑LR‑RGEAIAPELLKAIEESRSSVIVFSENYAHSRWCLDELVKIMECQ‑KDPAHV‑VFPIFYHVDPSHVRKQEGSFGEAFAGY‑EENWKD‑‑‑‑‑‑‑‑KIPRWRRALTEAAN‑LSGW‑‑‑HI‑L‑‑‑‑DGYESNQIKEIT

>D7TLC5

GEDTRKNFTDHLYNTLVAYGIHT‑FRDDEELL‑KGEDIKSGLSRAIEGSKIFIIIFSENYAASKWCLNELAMIIEYT‑TLEDNK‑VIPVFYHVKPSDVGHQSESFEVAFFNH‑EKDDQEK‑‑‑KE‑‑LIEKWRITLKKAAK‑LSGY‑‑‑HVDN‑‑‑‑‑‑HEAEVIQKIR

>F6I3U8

GEDTRNNFTAHLYQELRTKGINT‑FIDDDKLE‑RGRLISPALVTAIENSMFSIIVLSENYASSKWCLEELAKILECM‑KTRGQR‑VLPIFYNVDPSDVKKQRGKFGAALAEH‑EKNLTEN‑‑‑ME‑‑RVQIWKDALTQVAN‑LSGW‑‑‑ES‑R‑‑‑‑NKNELLLIKEIV

>F6I421

GEDTRKSFTDHLHTALCQKGINT‑FMDDQ‑LR‑RGEQISPALLNAIEESRFSIIIFSDNYASSSWCLDELVKILDCI‑KVMGHR‑ALPVFYNLNPSHVKKQTGSFAEAFAKH‑EQEYREK‑‑‑ME‑‑KVVKWREALTEVAT‑ISGW‑‑‑DS‑R‑‑‑‑DRHESKLIEEIV

>F6I423

GEDTRKNFTTHLHAALCQKGINT‑FKDNL‑LL‑RGEKISAGLLQAIEESRFSIIIFSENYASSSWCLDELTKILECV‑EEGGHT‑ALPVFYNVDPSHVRKQKGCFADAFAEH‑EQVYREK‑‑‑ME‑‑KVVKWRKALTEVAT‑ISGW‑‑‑DS‑R‑‑‑‑DRDESEVIEEIV

>F6GYX0

GEDTRYGFTDHLYEALISCGIRT‑FRDDEELA‑RGGIIASELLEAIEESKIFVIIFSENYAASRWCLDELVKISECG‑ATEGRR‑ILPIFYHVDPSHVRKQRGSYEKAFVDH‑EKEDEEK‑‑‑RE‑‑KIQKWRSALAKVGN‑LAGY‑‑‑DLQK‑‑‑‑‑‑YEARLIKEII

>F6GW04

GEDTRQTFTGHLYAKLVARGIHT‑FRDDEELE‑KGGDIASDLSRAIEESKIFIIIFSKRYADSKWCLNELAKIMDCK‑KEKGSV‑VVPVFYHVEPTDVRNQGGSFEDAFLEH‑AKDDQEK‑‑‑KK‑‑TIETWKNALKTAAN‑LSGY‑‑‑HLQN‑‑‑‑‑‑SEAEFIQGIF

>F6HPI4

GEDTRGTFTDCLYTRLQHKGVRA‑FRDNEGLN‑RGDKIDRCLLDAIEDSAAFIAIISPNYANSRWCLEELAKVCECN‑‑‑‑‑RL‑ILPVFYNVDPSHVRGQRGPF‑QHFKDL‑EARFG‑‑‑‑‑EE‑‑DVSKWRKAMKYVGG‑LAGF‑‑‑‑FLSI‑G‑RGDEADVIQTLL

>F6I419

GEDTRKSFTDHLHTALCQKGINT‑FMDDQ‑LR‑RGEQVSPALLNAIEESRFSIIIFSDNYASSSWCLDELVKILDCI‑KVMGHR‑ALPVFYNVNPSHVKKQTGSFAEAFAKH‑EQENREK‑‑‑ME‑‑KVVKWREALTEVAT‑ISGW‑‑‑DS‑R‑‑‑‑DRHESKLIEEIV

>F6I445

GEDTRYGFTDHLYEAFISHGIRT‑FRDDEELE‑RGGMIASDILNAIEESKIFVIIFSENYATSRWCLDELVRIFECT‑ATEKRL‑ILPVFYHVDPSEVGEQSGSYEKAFVDH‑EKEDEEK‑‑‑KE‑‑EIQKWRIALRKAAN‑LAGY‑‑‑DLQK‑‑‑‑‑‑YETRLIKEII

>F6HN42

GEDTRNNFTAHLLKELRTKGIDT‑FIDEERLE‑TGQVISPALVAAIESSKLSIIVLSENYASSRWCLEELVKILECK‑RTRGQ‑‑VLPIFYDVDPSDVRNHRGKFGEALAKH‑DVR‑‑‑‑‑‑NMD‑‑RVPIWRVALTEVAN‑LSGR‑‑‑DS‑R‑‑‑‑NKNEATFIEEIA

>F6H8W1

GADTRSNFTDHLYSALGRRGIRT‑FRDDK‑LR‑EGEAIGPELLTAIEESRSSVIVFSENYAHSTWCLDELVKIMERH‑KDRGHA‑VFPIFYHVDPSHVRRKTESFGKAFAGY‑EGNWKD‑‑‑‑‑‑‑‑KIPRWKTALTEAAN‑LSGW‑‑‑HQ‑R‑‑‑‑DGSESNKIKEIT

>D7LQF8

GEQLRYSFVSHLVDAFERNEVNF‑FVDKY‑‑EQRGKDLK‑NLFLRIQESRIALAIFSTRYTESSWCMDELVKMKKL‑ADK‑RKLHVIPIFYKVKVDDVRKQTGEFGDNFWTL‑AKVS‑‑‑‑‑‑‑SGDQIKKWKEALECISNKM‑GLSLGD‑‑K‑‑‑‑S‑SEADFVKEVV

>D7MW48

GADVRKHFISFLDPALRKANINV‑FIDED‑‑EYLGSDLV‑NLLKRIEESEIALVIFSEEFTSSYWCLEELAKIKDC‑KDQ‑‑‑‑‑‑‑‑‑‑‑‑‑‑‑‑‑‑‑‑‑‑GKFGDHFRDQ‑IRNLRH‑‑‑‑‑QPERTQKWEDALLSIPESI‑GMPLAA‑‑Q‑‑‑‑S‑DDKDFITSMV

>D7MKY7

GEDLRLGFVSHLVEALENDNIKV‑FIDNY‑‑ADKGEPLE‑TLLTKIQESRIALAIFSGKYTESTWCLRELAMIKDC‑VEK‑GNLVAIPIFYKLDPSTVRGVRGKFGDAFRDL‑EER‑‑‑‑‑‑‑‑DVLKKKEWKKALKWIPDLI‑GITVHD‑‑K‑‑‑‑S‑PESEILNEIV

>D7KPK0

GADTRDNFGGRLYEALMKK‑VRV‑FRDNEGMK‑RGDEIGSSLQASMEDSAASVIVLSPNYANSHWCLDELAMLCDLK‑SSLDRR‑MLPVFYMVDPSHVRKQSGDF‑KDFQKL‑AKTFS‑‑‑‑‑EA‑‑EIKRWKDAMKLVGN‑LAGY‑‑‑‑VCHK‑‑‑‑SKEDDIIELVV

>D7MKA7

SSKDRHSFVSHLHAAFGRRGISV‑FLESE‑KG‑F‑AE‑IQ‑‑‑LAIERSKIYVVVFSKNYASSPLCLETLMTFMDLQ‑RKDGPV‑VIPVFYGVTRSIVEQQTERFKEDFSKH‑R‑G‑‑‑F‑‑EKD‑‑RVERWRKGLTEAAK‑LHGH‑‑‑ES‑E‑‑‑‑Q‑NDSELVEDIV

>D7MBP1

GKQLRKGFVSHLEKALKRDGINA‑FIDED‑‑ETRGNDLS‑ILFSRIDESRIALAIFSSMYTESNWCLDELVKIKEC‑VDL‑GKLVVIPIFYKVETDDVKNLKGVFGDKFWEL‑VKT‑‑C‑‑‑‑‑NGEKLDKWKEALKVVTNKM‑GFTLGE‑‑M‑‑‑‑S‑NEGEYVEKIV

>D7MLF8

GDELRDNFIRYLVWGLRDERVNV‑FIDRA‑‑EANRRDIR‑NISTKIEESNIAVIIFSKRYTESEMCLNEHQKMHEH‑VKQ‑SNLKVIPVFYDVSISDVKNLEGEFGNHFEEM‑KMK‑‑‑‑‑‑‑‑DPLKILNWENSLSSIVE‑‑‑GLTSEE‑‑H‑‑‑‑G‑TGLGLVMAIV

>D7MKX8

GAGVRKHFISFLDPALRKANINV‑FIDED‑‑EYLGSDLV‑NLLKRIEESEIALVIFSEDFTSSYWCLEELAKIKEC‑KDQ‑GRLKVIPIFYKVKPSVVKYLKGKFGDHFRDQ‑IRNLRH‑‑‑‑‑QPERTQKWEDALLSIPESI‑GMPLAA‑‑Q‑‑‑‑‑‑‑DKDFITSMV

>D7MKX0

GEELRRGFISFLEPTLKNENINV‑FIDEL‑‑ELRGRDLQ‑NLFVRIKESKIALVIFSKDYANSEWCLDELAMIKEC‑MDQ‑GNLDVIPIFYKVEPSVVKYLLGYFGENFMNL‑KNRYEN‑‑‑‑‑DPERTRKWEEALASVSQKF‑GLPFPE‑‑K‑‑‑‑S‑DDREFINSIV

>D7KQ34

GPDVRKSFLSHLRKQFNYNGI‑TMFDD‑QGIE‑RSETIAPSLIQAIRESRILIVILSTNYASSSWCLNELVEIMECK‑KVMGQI‑VMTIFYGVDPTHVRKQIGDFGKAFSETCSRN‑‑‑T‑‑‑DVE‑M‑RKWSKALTDVSN‑ILGE‑‑‑HLLN‑W‑‑D‑NEANMIEKVA

>D7MUT0

A‑DQ‑‑KFTPYLQ‑‑‑‑‑PGVMT‑‑‑‑‑‑QLSWR‑N‑‑‑‑TLV‑GF‑‑‑‑‑AMLVFSE‑YS‑‑‑‑‑‑‑DVTGLIRCR‑KNMGQIEAVPVFFDVKPTD‑‑E‑‑G‑Y‑DIFADLVEKRVDDTKRDAETSKMSSWSKKVEEIST‑‑SGL‑‑‑Q‑‑‑‑‑‑‑‑‑‑EMDFLEDV‑

>D7MGR3

GADTRNSFTSYLVQFLQRKGIDT‑FFDGK‑‑LRRGKDIS‑VVFDRIEQSKMSIVVFSENYANSTWCLEELWKIIQC‑REK‑FGHGVLPVFYKVRKSDVENQKGTFGVPFLSP‑KES‑‑D‑‑‑‑‑GQ‑KVGAWKEALKIASNIL‑GYVLPE‑‑E‑‑‑‑R‑PESEFVEKIA

>D7MQS4

GEDVRRDFLSHIQMEFQRMGI‑TPFID‑NEIE‑RGQSIGPELIRAIRESKIAIILLSRNYASSSWCLDELAEIMKCR‑EELGQT‑VLAVFYKVDPSDVKKLTGDFGKVFKKTCAGK‑‑‑T‑‑‑KEH‑V‑GRWRQALANVAT‑IAGY‑‑‑HSTN‑W‑‑D‑NEATMIRNIA

>D7MM05

GEDVRKGFLSHIQKEFQRMGI‑TPFID‑NEMK‑RGGSIGPELLQAIRGSKIAIILLSRNYGSSKWCLDELVEIMKCR‑EELGQT‑VMTVFYDVDPSDVRKQKGDFGKVFRKTCVGR‑‑‑P‑‑‑EEV‑K‑QKWKQALTSAAN‑ILGE‑‑‑DSRN‑W‑‑E‑NEADMIIKIA

>D7MK04

GADVRKTFLAHILKEFKGKGI‑VPFID‑NDIE‑RSKSIGPELIEAIKGSKIAIVLLSRNYASSSWCLNELVEIMNCR‑EELGQT‑VMTIFYDVDPTDVKKQTGDFGKVFKKTCKGK‑‑‑T‑‑‑KED‑I‑KRWQNVLEAVAT‑IAGE‑‑‑HSRN‑W‑‑D‑NEAAMTKKIA

>D7KR50

GKDTRKNFISFLHKELESKGIRT‑FKDDTELE‑RGRPISPELLQAIKGSRIAVVVVSVNYPDSFWCLEELKEILKLE‑NQ‑GLLTVIPIFYEIDPSAVRRQIGVVAKQFKKH‑EKR‑‑‑E‑‑SKE‑‑RVKSWKEALNKLAN‑LSGE‑‑‑CS‑KDW‑‑E‑DDSKLVDGIT

>D7LMJ8

GQDVRVDFLSHIQKEFRRKGI‑IPFID‑NEIR‑RGESIGPELIKAIRESKIAVVLFSRNYGSSKWCLDELVEIMKCR‑EEFGQT‑VIPIFYKVDPSNVKKLTGDFGSVFRNTCAGK‑‑‑T‑‑‑KEV‑I‑GRWRQALAKLAT‑IAGY‑‑‑DSHN‑W‑‑Y‑NEAAMIEKIV

>D7LI65

GKDMRRHFVSYLTHALKMNGVSF‑FLDEM‑‑EVKGVDLG‑YLFKRIEESKLALVIISSRYTESAWCLNELVKIKEL‑RDE‑GKLVAIPIFYKVEPSQVKKLKGVFGDNFRSL‑CRM‑‑N‑‑‑‑‑QDHHITKWMEALMSMASTM‑GFYLDE‑‑Y‑‑‑‑S‑SESEFIKHIV

>D7MW45

EEDVSKGLINFLEPILKNENINV‑FIDEE‑‑TVRGKDLK‑NLFKRIQDTRISLAIFSESKCD‑‑‑‑FNELRKIKEP‑VDE‑‑‑‑‑AIPIFYKVDAI‑‑‑‑‑‑‑‑‑GD‑‑‑‑‑‑‑‑‑‑‑‑‑‑‑‑‑‑‑‑‑‑‑‑‑‑‑‑‑‑‑‑‑‑‑‑‑‑‑‑LAD‑‑L‑‑‑‑Q‑NKKDLINSAV

>D7KC67

GKDVRKTFLSHQLKEFGRKAI‑NFFVD‑NEIK‑RGEFIGPELKRAIKGSKIAVVLLSKNYASSSWCLDELVEIMK‑‑‑KESGQT‑VITIFYEVDPTDVKKQKGDFGKVFKKTCKGK‑‑‑G‑‑‑KEK‑V‑QTWKKALEGVAT‑IAGY‑‑‑HSSN‑W‑‑V‑DESTMIENIA

>D7KFH5

GKDTRRTFISFLYKELIGMSIRT‑FKDDVELK‑SGRRIASDLLMAIENSKIAVVIVSKNYPASPWCLQELEMIMDVE‑KK‑GSLIVMPIFYNVEPAHVRRQIEKVAQQFRKH‑ENR‑‑‑E‑‑NYE‑‑TVVSWRQALTNLAS‑ISGH‑‑‑CS‑RDC‑‑E‑DDSKLLDEIT

>D7MK93

GKDLRNGFVSHLVEALIRNKINV‑FMDKF‑‑EDRGKSLE‑SLLTRIEESRIALAIFSENYTESDWCVKEADKMNDC‑MKE‑GTLVVIPIFYKVKPSTVRDLEGRFGNKFWSL‑VKG‑‑‑‑‑‑‑‑DE‑RKKKWEEVWKSIPNLF‑GITVDE‑‑K‑‑‑‑S‑DENRTVNEIV

>D7MK94

GEELRRPFVSHLHEALRNVGINA‑FIDSD‑‑EDPGEDLE‑NLFKRIEESEIALAILSSKYTESQWCLDELVKIMEC‑SSKEKKLWVIPIFYKLDTSIVKGLDGDFGVNLWKL‑WTKGGV‑‑‑‑‑RDDRIVKWNAALQDARNKT‑ALILKE‑‑S‑‑‑‑S‑EEMAFLAKIV

>D7LXN7

CEETRRSFVSHLSSALHREGISV‑CVA‑‑‑‑‑‑‑‑DD‑FD‑‑‑DQNQGARVTVVVFSENYAFPHPMLDNFAKILQLR‑SNSGHE‑VIPVFYGVDPSAVNPNH‑‑‑‑‑‑‑‑‑‑‑‑‑‑‑‑‑‑‑‑‑‑‑‑‑‑‑‑DWLPL‑‑HMEG‑HSMN‑‑‑SS‑V‑‑‑‑R‑SDSQLVEDIV

>D7MCA2

GEDVRQSLISHLRKELDGKLV‑NTFND‑TRIE‑RSRKINPELLLAIEGSRISLVVFSKNYASSTWCLDELVKIQECH‑EQLDQM‑VIPIFYNVDPSHVRKQTGEFGKVFVETCKGR‑‑‑T‑‑‑ENE‑K‑RKWMRALTEVAN‑LAGE‑‑‑DLRN‑G‑‑R‑SEAEMLENIA

>D7MRE0

GEDVRITFLSHFLKELDRKLI‑IAFKD‑NEIE‑RSQSLDPELKQAIRSSRIAVVVFSEKYPSSSWCLDELLEIVRCK‑EELGQL‑VIPVFYGLDPSHVRKQTGQFGEAFAKTCQRK‑‑‑T‑‑‑EDE‑T‑KLWRQSLTDVAN‑VLGY‑‑‑HSQN‑W‑‑P‑SEAKMIEAIA

>D7MIU1

GEDVRRGFLSHLHYHFASKGI‑MTFND‑QKIE‑RGHTIGPELVRAIRESRVSIVVLSKRYASSSWCLDELLEILKCK‑EDDGQI‑VLTIFYQVDPSDVRKQRGDFGSAFEITCQGK‑‑‑P‑‑‑EEV‑K‑LRWSNALAHVAT‑IAGE‑‑‑HSLH‑W‑‑P‑NETEMIQKIA

>D7KYM9

GAELRYSFVSHLSDAFERHGINF‑FLDNH‑‑ELRGKDLA‑NLFVRIEESRIALAIFSTRYAESSWCMDELVKMKKC‑VDK‑GKLKVIPIFYKIRARDVRGQTGKFGDKFWEL‑AKVS‑‑‑‑‑‑‑RGDKIKKWKDALECITGKM‑GLSLGK‑‑K‑‑‑‑C‑SEAGFIMEII

>D7MKB3

SEERRWSFVPHLSAAFGRKGISV‑STM‑‑‑‑‑‑‑‑‑‑‑‑N‑‑‑DEF‑‑‑VASLLVFSEKYVSSKESLDEVVKTIQQR‑HDKGHV‑VATVFYGVSRSDVQELKGNFGKVLLEN‑G‑A‑‑‑‑‑‑‑SD‑‑QVTQWHNALAEIAS‑LPGY‑‑‑EA‑N‑‑‑‑T‑SDYEFVEKIT

>D7MRE5

GEDVRNTFLSHFLKELDSKLI‑ISFKD‑NEIE‑RSQSLDPELKHGIRNSRIAVVVFSKNYASSSWCLNELLEIVKCK‑KEFGQL‑VIPIFYHLDPSHVRKQTGDFGKIFEKTCRNK‑‑‑T‑‑‑VDE‑K‑IRWKEALTDVAN‑ILGY‑‑‑HIVT‑W‑‑D‑NEASMIKEIA

>D7KVG7

GPDVRKTFLSHLRKQFNYNGI‑TMFDD‑QGIE‑RSQTIAPALTRAINESRIAIVVLSKNYASSSWCLDELVQILKCK‑EDRGQI‑VMTVFYGVDPHDVRKQTGDFGRAFNETCARK‑‑‑T‑‑‑EEE‑R‑RKWSQALNYVGN‑IAGE‑‑‑HFRN‑W‑‑D‑NEAKMIEKIA

>D7LI64

GADLRYGFIDHLKKAFMANNIRY‑YIDEI‑‑EPRGENLG‑ILFQRIRESRIALVFFSNRYPESEWCLDELVEIMKN‑MEN‑DTLRVIPIFFKVKPEDVRGQKKEFGVALYGE‑GRR‑‑‑‑‑‑‑‑RRPRMPQWEDALEAIPSNM‑GLVFQE‑‑Q‑‑‑‑S‑SEADFLAKLI

>D7MKY4

GAELRNGFVSHLVTALQSKDINV‑FIDKL‑‑EDRGKPIE‑ILLDRIQKSRIALVIFSGKYTESVWCMREVAKIKDC‑MDE‑GTLEVIPIFYKVEPSTVKYLMGDFGDTFRSL‑AMN‑EY‑‑‑‑‑DE‑GKEKWEDALKAVSGIM‑GTVVDE‑‑K‑‑‑‑S‑EESEIVKKTV

>D7MGR4

NHDVETSFIEAISKELHKREVTP‑LMN‑‑‑LS‑R‑EL‑DE‑‑‑KMLYRSSVGIMILSHSYACSRQALDHLVEIMEHG‑KARNLV‑IIPIYFKATLSDICGLEGRFEPIYLQY‑M‑D‑‑‑S‑‑‑LS‑‑RVQKWKAAMAEIAS‑IDGH‑‑‑EW‑K‑‑‑‑‑‑KQVLLAEEVV

>D7MKB6

GADVRYNFISHLEKALKDAGINV‑FVDED‑‑EKRGKDLT‑VLFHRIEGSNMAIVVFSERYMESEWCLNELAKIKER‑VDE‑GKLVAIPIFFKVGADELKELLDVACETHGNV‑PGT‑‑‑‑‑‑‑‑‑‑‑‑‑QKWKVALECTTLKM‑GLTLGK‑‑K‑‑‑‑S‑DEANFVKMVV

>D7KR56

GLDTRRNFISFLYNELVRRNIRT‑FKDDNELK‑NGRRITPELVRAIEGSKFAVVVVSVNYAASRWCLEELVKIMDFE‑NK‑GSLKVMPIFYGVDPCHVRRQIGVVAEQFKKH‑EAR‑‑‑E‑‑DHE‑‑KVLSWRQALTNLAS‑ISGD‑‑‑CS‑WKW‑‑E‑DDSKMVDEIT

>D7LXP5

GQDVRRSFLSHFLEGLKGKGI‑KTFVD‑HGIM‑RSDSINSELVRAIRESRIAVVILSKNYASSSWCLNELQLILECR‑VTLGQT‑VMTIFYDVDPSDVRKQTGDFGKVFEETCDGK‑‑‑T‑‑‑EEE‑K‑QRWRKALTEVAV‑IAGE‑‑‑HSVS‑W‑‑A‑SEAAMISKIV

>D7MIU4

GPDVRRTFLSHLQHHFASKGI‑TVFKD‑QEIK‑RGQTIGLELKQAIRESRVSIVVLSKKYASSSWCLDELVEILKCR‑EACGKI‑VMTIFYEIDPFHVRKQIGDFGRAFRETCFSK‑‑‑T‑‑‑KKV‑R‑LKWSKALTDVAN‑IAGE‑‑‑HSLR‑W‑‑E‑DEAKMIEKIA

>D7MKB2

GDQLHYNFVSYLVDALRRSEINV‑FIDNE‑‑EQRGEDLN‑TLFKRIEESGIAIVVLLTHITN‑‑‑‑FGNIVTLYHK‑V‑‑‑‑‑‑‑‑LPIFYKVTPTNVKRLKGEFGDHFRDK‑EYMYKS‑‑‑‑‑DEPMIKQWKEAIVSVSHKF‑ALALDE‑‑K‑‑‑‑S‑SEIDFVETIV

>D7MRZ0

GGDVRVTFRSHFLKELDRKLI‑TAFRD‑NEIE‑RSHSLWPDLEQAIKESRIAVVLFSKNYASSSWCLNELLEIVNCN‑D‑‑‑KI‑VIPVFYGVDPSQVRHQIGDFGSIFEKTCRRH‑‑‑S‑‑‑EEV‑K‑NQWKKALTDVAN‑MLGF‑‑‑DSAT‑W‑‑D‑DEAKMIEEIA

>D7LX32

GEDVRKTFVSHLFCEFDRMGINA‑FRDDLDLQ‑RGKSISPELIDAIKGSRFAIVVVSRNYAASSWCLDELLKIMEC‑‑KDTSQT‑ILPIFYEVDPSDVRRQRGSFGEDVESH‑DKE‑‑‑‑‑‑‑‑‑‑‑KVRKWKEALKKLAA‑ISGE‑‑‑DS‑R‑‑‑‑NRDESKLIKKIV

>D7KR43

GKDTRRTVVSFLYKDLIRQGILT‑YKDDQGIG‑AGSEIKERLIEAIKTSQVAVVFISENYATSQWCLEELRLIMELH‑SV‑NRIHVVPIFYRVDPSDVRHQKGRFAAAFQKH‑E‑‑‑‑‑‑‑‑‑PN‑‑RASQWRRALNQISH‑ISGI‑‑‑HS‑TEW‑‑D‑DDSAMIDEVV

>D7LMK1

GADVRRTLLSHIMESFRRKGI‑DTFID‑NNIE‑RSKPIGPELKEAIKGSKIAIVLLSKNYASSSWCLDELAEIMKCR‑EVLGQI‑VMTIFYEVDPTDIKKQTGDFGKAFRKTCKGK‑‑‑T‑‑‑KEH‑I‑ERWRKALKDVAI‑IAGE‑‑‑HSRN‑W‑‑S‑NEAEMIEKIS

>D7MUS0

GADLRLRFVSHLVTALKLNNINV‑FIDDY‑‑EDRGQPLD‑VLLKRIEESKIVLAIFSGNYTESIWCVRELEKIKDC‑TDE‑GTLVAIPIFYKLEPSTVRDLKGKFGDRFRSM‑AKG‑‑‑‑‑‑‑‑DE‑RKKKWKEAFNLIPNIM‑GITIDK‑‑K‑‑‑‑S‑VESEKVNEIV

>D7KRU8

GKDLRKGFLSFLSPALKKEKINV‑FIDEQ‑‑EERGKYLI‑SLFNTIGESKIALVIFSEGYCESHWCMDELVTIKEY‑MDK‑NRLKIIPIFYRLELDVVKDLTGKFGDNFWNM‑VDNYQP‑‑‑‑‑EPEKLHKWTEALFSVCQLF‑ALILPK‑‑H‑‑‑‑S‑DDRDFVKVIV

>D7MKY8

CVSERYSFVSHLSEALRRKGISS‑VIDDD‑‑‑‑‑‑SE‑SQ‑‑‑AKIEISRVSVMVLSRICEPTRVC‑QNFVNVIECQ‑RNKNQV‑VVPVLYGESPLLG‑‑‑‑‑‑‑‑‑‑‑‑‑‑‑‑‑‑‑‑‑‑‑‑‑‑‑‑‑‑‑‑EWLSVL‑DLRD‑LPVH‑‑‑QS‑K‑‑‑‑D‑SDSQFVKEIV

>D7MKX9

GKDVRNGFLSFLEPAMREANINV‑FIDKH‑‑EVVGTDLV‑NLFVRIQESRVVVVIFSKDYTSSEWCLDELAQIKDC‑IDQ‑GGLNVIPIFYKLAPSSVEELKGGFGDSFRVL‑KCKYKD‑‑‑‑‑EPERTQKWEEALKSIPKIK‑GLTLSE‑‑K‑‑‑‑S‑DEREFMNETI

>D7MKA4

N‑DSVSSFISYLIAAFNRQGIIS‑AFDHD‑‑‑‑‑‑RM‑GP‑‑‑EEFSKLRVVVVVFSKNYALHVSFLEKQI‑‑LEYS‑RNNNDFVVVPVFYGVSISSVNQHMERFGEEFDAI‑Q‑R‑‑‑S‑‑‑‑‑‑‑‑‑IKWRP‑‑‑‑‑‑‑‑‑‑GH‑‑‑EYCK‑‑‑RR‑SESEFLEEIA

>D7MUS9

GADIRFGFVSHLVEAFKKHKINF‑VYDDY‑‑EDRGQPIE‑ILLTRIEQSRIALAIFSGKYTESFWCLEELTKIRNC‑EKE‑GKLVAIPIFYKVEPSTVRYLMGEFGDSFRSL‑PKD‑‑‑‑‑‑‑‑DE‑KKKEWEEALNVIPGIM‑GIIVNE‑‑R‑‑‑‑S‑SESEIIKKIV

>D7MKY3

CVKQRYSFVSHLSEALRRKGIID‑VF‑DD‑‑‑‑‑‑SE‑SQ‑‑‑SKVERARVSVVVLS‑‑‑GNSTVCLDKLVNVLGCQ‑RNIDQV‑VVPVLYGEIPLQV‑‑‑‑‑‑‑‑‑‑‑‑‑‑‑‑‑‑‑‑‑‑‑‑‑‑‑‑‑‑‑‑EWDKAL‑NSRG‑LSVH‑‑‑QS‑N‑‑‑‑K‑TDSELVEEIT

>D7KR54

GLDTRRNFISFLYKELVRRNIRT‑FKDDKELE‑NGRRISPELKRAIEESKFAVVVVSVNYAASPWCLDELVKIMDFE‑NK‑GSITVMPIFYGVDPCHLRRQSGDVAEQFKKH‑EAR‑‑‑E‑‑DLD‑‑KVASWRRALTSLAS‑ISGD‑‑‑CS‑LKC‑‑E‑DESKLVDEIA

>D7MRE1

GEDVRVSFRSHFLKELDRKLI‑TAFKD‑NEIK‑KSHSLWPELVQAIKESRIAVVVFSKNYASSSWCLNELLEIVNCN‑D‑‑‑KI‑VIPVFYGVDPSHVRNQTGDFGRIFEETCEKN‑‑‑T‑‑‑EQV‑K‑NRWKKALSDVAN‑MFGF‑‑‑HSAT‑W‑‑D‑DEAKMIEEIA

>D7KR46

GKEMRKTFVSHLLSSFTTKNITS‑FID‑‑PFE‑‑‑D‑‑‑PDLK‑AMEQSLVAIPVISKNYVSNLWMDD‑LRKIIEC‑‑EK‑GTLTAIPIFFQVSPLDILHAT‑‑‑‑EKYADT‑QDE‑‑‑T‑‑‑‑E‑‑‑VRKW‑‑‑‑‑‑LAQ‑VSSF‑‑‑HS‑NDW‑‑D‑DDSELVDKIV

>D7MVQ3

GEYVRRDFLSHIQMEFQRMGI‑TPFID‑NEIE‑RGQSIGPELIRAIRESKIAIILLSRNYASSSWCLDELAEIMKCR‑DELGQT‑VLAVFYKVDPSDVKKLTGDFGKVFKKTCAGK‑‑‑T‑‑‑KEH‑V‑GRWRQALANVAT‑IAGY‑‑‑HSTN‑W‑‑D‑NEAAMIKKIA

>D7MIS2

GPDVRRGFLSHLHNHFASKGI‑TTFND‑EKID‑RGQTIGPELVQAIRESRVSVVLLSKKYASSSWCLDELLEILKCN‑EAQGQI‑VMTIFYDVDPSDVKKQRGEFGKAFEKTCEGK‑‑‑T‑‑‑EEV‑K‑QRWIEALAHVAT‑IAGE‑‑‑HSLN‑WARA‑NEAEMIQKIA

>D7LXP7

GEDVRKNFLSHLQKELQLRGI‑NAFKD‑HGIK‑RSRSIWPELKQAIWESRISIVVLSSNYAGSSWCLDELLEIMECR‑EAVGQT‑LLTVFYEVDPSDVRKQTGAFGKVFEKTCLGR‑‑‑T‑‑‑VEE‑T‑QRWKQALTDVAN‑VSGY‑‑‑CSEK‑W‑‑D‑NEASMIEKIV

>D7MJ12

GPDVRNGFLSHLHNHFESKGI‑TTFND‑QEIE‑RGHTIGPELVQAIRESRVSIVVLSEKYASSGWCLDELVEILKCK‑EASGQA‑VLTIFYKVDPSDVRKQRGDFGNTFKKTCEGK‑‑‑T‑‑‑EEV‑K‑QRWIKALTDVAT‑IAGE‑‑‑HSLN‑W‑‑A‑NEAEMIQKIA

>D7MRE2

GEDVRITFLSHFLKELDRKLI‑IAFKD‑NEIE‑RSQSLDPELKQAIRTSRIAVVVFSEKYPSSSWCLDELLEIVRCK‑EELGQL‑VIPVFYGLDPSHARKQTGKFGEAFVKTCQRK‑‑‑T‑‑‑EDE‑T‑KLWRQSLTDVAN‑VLGY‑‑‑HSQN‑W‑‑P‑NEAQMIEAIA

>D7LLZ1

GEDVRKDFLSHIQKGFERKGI‑RQFND‑YEIE‑RGESISFQLIRAIRGSKIAVILFSRNYASSKWCLDELMEIMKCR‑RELGQI‑VIAIFYKVDPSDVRNQSGDFGKVFRKTCAGK‑‑‑T‑‑‑KEE‑I‑RRWRTALAEVAT‑IAGY‑‑‑HSSN‑W‑‑D‑NEAAMIENIA

>D7MRD3

GDDLRHNFLAHFRKELDRKLI‑RTFND‑MEIE‑KGESLDPVLTQAIRGSKIAVVLFSKNYASSGWCLNELLEIVKCK‑KEIGQL‑VIPIFHGVDPSHVRHQIGDFGSIFEKTCRRH‑‑‑S‑‑‑EEV‑K‑NQWKKALTEVAN‑MVGT‑‑‑HLQN‑W‑‑D‑NEAKQIEYIV

>D7MJ13

GPDVRSGFLSHLHNHFESKGI‑TTFND‑QEIE‑RGHTIGPELVQAIRESRVSIVVLSEKYASSGWCLDELVEILKCK‑EASGQA‑VMTIFYKVDPSDVRKQRGDFGYTFKKTCEGK‑‑‑T‑‑‑EEV‑K‑QRWIKALNDAAT‑IAGE‑‑‑NSLN‑W‑‑A‑NEAEMIQKIA

>D7KSY0

GEDVRKTFLSHIRKQFICNGI‑TMFDD‑QGIK‑RGKTITPELIQGIRESRISIIVLSKNYASSSWCLDELLEILKCR‑EDIGQI‑VMTVFYGVDTSDVRKQTGEFGIAFNKTCAGK‑‑‑T‑‑‑EEE‑S‑RRWSQALTDAAN‑IAGV‑‑‑DFKN‑C‑‑K‑NEAEMIEEIA

>D7KXD9

GPDVRVTFLSHLQKQFQHNGI‑ITFND‑EGIE‑RSQTISSELTRAIRESRISIVVLSENYASSSWCLNELLEISKCQ‑ESAGQI‑VMTVFYKVDPSDVRKQMGEFGKAFKKTCQGK‑‑‑T‑‑‑EAK‑I‑HRWTQSLTHVAN‑IAGE‑‑‑HSLN‑W‑‑D‑NEANMIEKIA

>D7MVQ6

GEDVRRDFLSHIHMEFQRMGI‑TPFID‑NEIE‑RGQSIGPELIRAIRESKIAIILLSRNYASSSWCLDELAEIMKCR‑EELGQT‑VLAVFYKVDPSDVKKLTGDFGKVFKKTCAGK‑‑‑T‑‑‑KEH‑V‑GRWRQALANVAT‑IAGY‑‑‑HSTN‑W‑‑D‑NEATMIRNIA

>D7LM60

GEDVRVTFLSHFLKELDRKLI‑IAFKD‑NEIK‑KSESLDPVLKQAIKDSRIAVVVFSINYASSTWCLNELVEIVKCK‑EEFSQM‑VIPVFYRLDPSHVRKQTGDFGKIFEKTCHNK‑‑‑T‑‑‑EEV‑K‑IQWKEALTSVAN‑ILGY‑‑‑HSTT‑W‑‑F‑NEAKMIEEIA

>D7MVI5

‑‑‑‑‑‑GFIDHLYINLKRSGIHT‑FKDDEALK‑RGENLSPTLLKAIKSSKVHLVVLTENYSSSMWCLDELMHIMECR‑RNNGHV‑VVPIFYDVEPRDVRRQRGSFGAYFSKH‑EARHPE‑‑‑‑‑‑‑‑KVQKWKDALTEVAN‑RLGH‑‑‑‑VRA‑‑‑‑NYSEVELIYEIT

>D7MEA2

GVDVRRDFLSHIQKEFQRKGI‑TPFID‑NEIK‑RGESIGPELIHAIRGSKIAIILLSRNYASSKWCLDELVEIMKCR‑EELGQS‑VMVIFYKVDPYDVKTLAGEFGKVFSKTCAGN‑‑‑T‑‑‑KED‑I‑KRWRQALGKVAT‑IAGY‑‑‑HSSN‑W‑‑D‑NEAAMIERLA

>D7MCA5

GEDVRKTFLSHLLKALDGKSI‑NTFMD‑HGIE‑RSRTIAPELISAIREARISIVIFSKNYASSTWCLNELVEIHKCC‑KDLDQM‑VIPVFYYIDPSEVRKQIGEFGDVFKKTCEDK‑‑‑P‑‑‑EDQ‑K‑QRWVQALTDISN‑IAGE‑‑‑DLRN‑G‑‑P‑DEAHMVEKIV

>D7KFH3

GEDTRKTIVSHLYAALDSRGIVT‑FKDDQRLE‑IGDHISDELRRALGSSSFAVVVLSENYATSRWCLLELQLIMELM‑KE‑GRLEVFPIFYGVDPSVVRHQLGSF‑‑ALEKY‑Q‑‑‑‑‑‑‑‑‑AD‑‑KVLRWREALNLIAN‑LSGV‑‑‑VS‑SHC‑‑V‑DEAIMVGEIA

>D7MW47

GKDVRNGFLSFLEPAMREANINV‑FIDKH‑‑EVVGTDLV‑NLFVRIQESRVVVVIFSKDYTSSEWCLDELAQIKDC‑IDQ‑GGLNVIPIFYKLAPSSVEELKGGFGDSFRVL‑KCKYKD‑‑‑‑‑EPERTQKWEEALKYIPKIK‑GLTLSE‑‑K‑‑‑‑S‑DEREFIYETI

>D7MKZ4

GDELRKSFLGFLVKAMRDANINV‑FTDEI‑‑EVKGKDLQ‑NLFSRIEESRVAVAILSKRYTESSWCLDELVKMKER‑NDQ‑DKLVVIPIFYRLDANNCKRLEGPFGDNFRKL‑EREYRS‑‑‑‑‑EPERIKKWKEALIYIPQKI‑GLTSAG‑‑H‑‑‑‑R‑R‑TVVSSRIH

>D7KXG7

GEDVRRKILSYIQQEFQRKGI‑IPFID‑NEIK‑RGESIGPELIKAIRESKIAIVLLSRNYASSKWCLDELVEIMECK‑KKFGLT‑VFVVFYEVDPSHVKKLTGEFGAVFQKTCKGR‑‑‑T‑‑‑KEE‑I‑WRWRQAFEEVAT‑IAGY‑‑‑DSRD‑W‑‑E‑NEAAMV‑‑‑‑

>D7L878

GPDVRKSFLSHFRKQFISNGI‑TMFDD‑QKIV‑RSQTIAPSLTQGIRESRISIVILSKNYASSTWCLDELLEILKCR‑EDIGQI‑VMTVFYGVDPSDVRKQTGEFGTVFNKTCARR‑‑‑T‑‑‑KEE‑R‑RNWSQALNDVGN‑IAGE‑‑‑HFLN‑W‑‑D‑NEAEMIEKIA

>D7MKA8

GTELRNNFISHLEKALLNKKVNV‑FIDIR‑‑ERIGKDKD‑IFFQRIRESRITIAVISSKYTESKWCLNELAEIQKC‑VLA‑ETMEVFPVFYKVDVGTVEKQTGEFGENFKKL‑LEQ‑‑H‑‑‑‑‑HSER‑EKWERALKFVTSKL‑GVRVDE‑‑K‑‑‑‑S‑FECDIVDHVV

>D7KC69

G‑NLRYGFVSHLTDALKRHNINF‑FIDTH‑‑EQKGRDLK‑HLFKRIEEATVALVILSTRYAESKWCLDELTKIMDQ‑AEK‑MEMIVIPIFYKVKPKDVELQEGVFGDRFWSH‑ADQS‑‑‑‑‑‑‑SREEMEKWQVALKAVCNKV‑GITLYR‑‑K‑‑‑‑‑‑‑EAKFIKKVV

>D7KB25

GPDVRKTFLSHLRNQFNQNGI‑TMFDD‑NGIP‑RSENIPSALIQGIRESRISIIVLSKMYASSRWCLDELLEILKCK‑EDVGKI‑VMTVFYGVDPSDVRNQTGDFGIAFNKTCARK‑‑‑T‑‑‑KEH‑G‑RKWSEALDYVGN‑IAGE‑‑‑H‑‑N‑W‑‑G‑NEAEMIAKIA

>D7KC63

GKDVRKAFLSHILKEFGRKAI‑NFFVD‑NEIK‑RGEFIGPELKRAIKGSKIALVLLSKNYASSSWCLDELAEIMK‑‑‑QESGQT‑VITIFYEVDPTDVKKQKGDFGKVFKKTCKGK‑‑‑D‑‑‑KEK‑I‑KTWRKALEDVAT‑IAGY‑‑‑HSSN‑W‑‑V‑DEAAMIENIA

>D7KT09

GEDVRRDFLSHIHKEFQRKGI‑TPFID‑SEIK‑RGESIGLEIVQAIRGSKIAIVLLSRNYASSSWCLDELVEIMKCK‑EELSQI‑VIPIFYKVDPSDVKKLTGSFGSVFEDRCAGK‑‑‑T‑‑‑NEL‑I‑RRWRQALAKVAT‑ITGY‑‑‑DSRC‑W‑‑D‑NEAAMIEKIA

>D7MK98

GRDLRYGFVSHLEKILKDHKIEV‑FVDSG‑‑EDRGEHLE‑NLLTRIEESRIALAIFSENYTESEWCLRELAKIKDC‑VDQ‑KRLVAIPIFYKVEPSTVKYLMGEFGDAFRKL‑AKN‑‑‑‑‑‑‑‑DK‑RKKEWKAALRAIPEFM‑GIPVHE‑‑K‑‑‑‑S‑PESEILKTIV

>D7MW46

GADVRSHFISHLDPALREANINV‑FIDDD‑‑EFLGTDLV‑NLLKRIEESEIALVIFSEDFTSSYRCLDELAKIKEC‑KDQ‑GRLIVIPIFYKVKPSVVKYLKGNFGDNFREL‑ERNNLH‑‑‑‑‑MQQRTQKWKKALVSIPESK‑GMPRAE‑‑Q‑‑‑‑S‑EDKDFITSMV

>D7KTD8

GPDVRKTFLSHLRKQFICNGI‑TMFDD‑QGIE‑RGQTISPELTRGIRESRISIVVLSKNYASSSWCLDELLEILKCK‑EDIGQI‑VMTIFYGVYPSHVRKQTGEFGIRLSETCDGK‑‑‑T‑‑‑EEE‑R‑RRWSQALNDVGN‑IAGE‑‑‑HFLN‑W‑‑D‑KESKMVEKIA

>D7MVQ1

GEDVRRDFLSHIQMEFQRMGI‑TPFID‑NEIK‑RGQSIGPELIRAIRESKIAIILLSRNYASSSWCLDELAEIMKCR‑EELGQT‑VLAVFYKVDPSDVKKLTGDFGKVFKKTCAGK‑‑‑T‑‑‑KEH‑V‑GRWRQALANVAT‑IAGY‑‑‑HSTN‑W‑‑D‑NEAAMIKKIA

>D7LTW2

GEELRNSFVSHLRSALVRHGVNI‑FIDTN‑‑EQKGKPLH‑VFFERIEESRIALAIFSLRYTESKWCLNELVKMKEC‑MDK‑GKLLIIPIFYKVKAYEVRYQKGRFGYVFKNL‑‑RN‑‑A‑‑‑‑‑DVHQKNQWSEALSSVADRI‑GFPFDG‑‑K‑‑‑‑S‑DENNFINGIV

>D7L8B6

GEDVRRTFLSHLLLALDRKLI‑TCFKD‑SEIQ‑RSQSIGLELVHAIRGSRIAIVVFSKIYASSSWCLNELLEIVKCK‑EEKGQM‑VIPIFYALDPSHVRKQTGDFGKAFEMICESK‑‑‑T‑‑‑DEL‑Q‑IQWRRALTDVAN‑IHGY‑‑‑HSEN‑W‑‑Y‑NEAHLIEEIA

>D7MVQ4

GEDVRRDFLSHIQMEFQRMGI‑TPFID‑NEIE‑RGQSIGPELIRAIRESKIAIILLSRNYASSSWCLDELAEIMKCR‑EELGQT‑VLAVFYKVDPSDVNKLTGDFGKVFKKTCAGK‑‑‑T‑‑‑KEH‑V‑GRWRQALANVAT‑IAGY‑‑‑HSTN‑W‑‑D‑NEATMIRNIA

>D7M8F3

GEDVRVTFLTHFFKELDRKMI‑IAFKD‑NEIE‑RGHSIGPKLIKAIKDSRIAVVVFSKNYSSSSWCLNELLEIVKCQ‑‑‑‑‑EI‑VIPIFYDLDPSDVRKQEGEFGESFKKTCKNR‑‑‑T‑‑‑KDE‑I‑QRWREALTNVAN‑IAGY‑‑‑HTGK‑P‑‑N‑DEAKLIEEIA

>D7KR45

GEDTRKGIVSHLHRAFLARGIKI‑FKDDQTLE‑IGDSISEEIKEAIHNSKFAILVISMNYASSTWCLDELQMIMELH‑KE‑KQLTAVPIFYNVDPSDVRHQRGTF‑‑ALERY‑EKR‑‑‑A‑‑MAA‑‑KIQKWREALREVAG‑TSGK‑‑‑DL‑STC‑‑K‑DEATMVADIV

>D7KSY5

GPDVRKTLLSHMRKQFNRNGI‑TMFDD‑EKIE‑RSATIAPSLIGGIRDSRISIVILSKKYASSSWCLDELVEILECK‑KVMGQI‑VMTIFYGADPSDVRKQLGEFGIAFDETCAHK‑‑‑T‑‑‑DEE‑R‑KKWSEALNEVGN‑IAGE‑‑‑DFNR‑W‑‑D‑NEANMIKKIA

>D7MLF9

GEKLRDGFLGFLVDALLKENVNV‑FIDDH‑‑ELRGRDLD‑HLFSRIEESRVALTIFSKNFTESRWCLDELAKIREC‑VDQ‑GSLTVIPIFFKMKTDDVKKLKGKFGDNFRDL‑KSTHRG‑‑‑‑‑EPENFRRWKEALIFVSEKA‑GLSSSR‑‑Y‑‑‑‑S‑R‑NDLVSTIV

>D7MRH7

GEDVRKTFLSHFLKELDRRLI‑LAFKD‑NEIE‑RSRSLDPELNHAFKGSKIAVVVFSRNYASSSWCLNELLEIVRCK‑EEFGQM‑VVPIFYHLDPSHVRNQTGDFGKMFEQTCQHK‑‑‑T‑‑‑EDQ‑K‑IRWRRALTDVAN‑ILGY‑‑‑HSVA‑W‑‑D‑NEASMVEEFA

>D7LXN6

GVELRYNFVSHLKKGLKRNGINA‑FIDTD‑‑EDMGQELN‑ILLKRIEGSKIALAIFSPRYTESDWCLKELAKMKEC‑REQ‑GKLVVIPIFYKVEPSTVKRQKGEFGDNFRDL‑VEF‑‑I‑‑‑‑‑DEETKNNWTEALKSIPLLT‑GFVLNE‑‑N‑‑‑‑S‑DEDDLIFKVV

>D7MJC1

GEDTRHSIVSHLYEALTSRGIAT‑FKDDKRLE‑LGDHISEELQRAIEGSDFAVVVLSENYPTSRWCLMELQSIMKLQ‑VE‑GRLGVFPVFYSVEPSAVRYHLGSF‑‑DLEGY‑Q‑‑‑‑‑‑‑‑‑AD‑‑VVPKWRQALKLIAD‑LSGV‑‑‑AS‑GQC‑‑I‑DEATMVRKIV

>D7MK10

GADVRKNFLAHILKEFKGKGI‑VPFID‑NDIE‑RSKSIGPELIEAIKGSKIAIVLLSRNYASSSWCLNELVEIMNCR‑EELGQT‑VMTIFYDVDPTDVKKQTGDFGKVFKKTCKGK‑‑‑T‑‑‑KED‑I‑KRWQNVLEAVAT‑IAGE‑‑‑HSCN‑W‑‑D‑NEAAMTEKIA

>D7LTW1

REDTRYSFVSHLSAAFHRRGVSS‑FTEDS‑‑‑‑‑‑NF‑SK‑‑‑‑‑LEKSRASVVVFSEKYPSSKSCMEELLKVSEHR‑RKNCLA‑VVPVFYPVTKSFVKKQICNLA‑‑‑‑‑‑‑‑‑‑‑‑‑‑‑‑‑‑D‑‑VRSDWRTALLETVD‑LPGH‑‑‑EL‑D‑‑‑‑T‑SDSDFVVEIV

>D7LNZ5

GEDVRRNFLSHIQKEFQRKGI‑TTFVD‑NEIK‑RGESIGPKLIHAIRGSKIALVLLSKNYASSSWCLDELVEIMKCK‑EELGQT‑VLPIFYKIDPSDVKKLTGKFGSAFKNICACK‑‑‑T‑‑‑NEI‑I‑RKWRQALAKVAT‑TTGY‑‑‑SSRN‑W‑‑D‑NEADMIEKIS

>D7LMK5

GADVRRTLLSHIMESFRRKGI‑DTFID‑NNIE‑RSKPIGPELKEAIKGSKIAIVLLSKNYASSSWCLDELAEIMKCR‑EVLGQI‑VMTIFYEVDPTDIKKQTGDFGKAFRKTCKGK‑‑‑T‑‑‑KEH‑I‑ERWRKALKDVAI‑IAGE‑‑‑HSRN‑W‑‑S‑NEAEMIEKIS

>D7KZ23

GEDVRKNFVCHFIKELDRKLI‑TAFKD‑NQIE‑RSRSLDPELKQAIRDSRIAVVVFSKNYASSSWCLDELLEIVRCK‑KEYGQL‑VIPIFYRLDPSHVRKQTGEFGKIFEKTCQHK‑‑‑T‑‑‑KQV‑Q‑NRWSRALSHVSN‑ILGY‑‑‑HSVT‑W‑‑E‑NEAKMIEEIT

>D7MKY5

GKELRKGFISFLVPALKDNNINV‑FIDDQ‑‑EERGKYLT‑SLFDRMGESKIALVIFSEDYTESKWCLDELVQIKEC‑MDQ‑NKLRVIPIFYKLDPAVVKRLQGKFGDQFRDL‑EYRYKH‑‑‑‑‑KPERPQKWKEAVISVCQTF‑ALFLPE‑‑H‑‑‑‑S‑DDKDFIMLIV

>D7MRE4

GEDVRLTFLSHLLKELDRKMI‑IAFKD‑NEIP‑RGQSLDPELKQAIRDSRIAVVVFSKNYASSTWCLNELLEIVQYK‑EEFGQM‑VIPVFYDLDPSHVRKQTGDFGKIFQETCKNK‑‑‑T‑‑‑EDV‑I‑NRWKKALTDVAN‑ILGY‑‑‑HSVT‑Q‑‑V‑NEAKMIEEIA

>D7LXU4

GEDVRRTFLSHLLKKFQLKGI‑RTFMD‑NDIE‑RGQMIGPELIQAIRESRFAVVVLSKTYASSKWCLDELVEIKE‑‑‑‑‑ASKK‑VIPIFYNVEPSDVKNIGGEFGNEFEKACKEK‑‑‑P‑‑‑‑EK‑L‑DRWREALVYVAD‑IAGE‑‑‑CSQN‑W‑‑V‑SEADMIENIA

>D7MLG0

GAELRNSFISHLEGALALAGIKY‑YIDTK‑‑EVPSEDLS‑VLFERIEQSEIALSIFSSKYAESNWCLDELVKIMEQ‑VKK‑EKLRIIPVFFNVKPEEVREQKGEFGLKLYGE‑GKR‑‑‑‑‑‑‑‑KRPNIPNWENALQSVPSKI‑GLNLSN‑‑Y‑‑‑‑R‑NERELVEKIV

>D7KR44

AEDTTNIFVSDLHRSLSEKGITTYQKDEKQEE‑KDSSVVSDLKKCIIESKLAVVVVSKSYPTSVLCLNQLQTIINFH‑DE‑GQLSVLPIFYGVDLSNIRNQTGEYTEAFRNL‑AEE‑‑‑F‑‑SPE‑‑KVQAWRSALAKLTS‑VSSL‑‑‑DS‑RFW‑‑S‑KEETMVDLVT

>D7MK07

GADVRKTFLAHILKEFKGKGI‑VPFID‑NDIE‑RSKSIGPELVEAIRGSKIAIVLLSRNYASSSWCLNELVEIMKCR‑EELGQT‑VMTIFYDVDPTDVKKQTGDFGKVFKKTCKGK‑‑‑T‑‑‑KED‑I‑KRWQNVLEAVAT‑IAGE‑‑‑HSCN‑W‑‑D‑NEAAMTEKIA

>D7MKB5

SEETRCSFVPHLSAAFGRKGISV‑LTK‑‑‑‑‑‑‑‑‑‑‑‑H‑‑‑DQSYKSIASVLIFSENYVSSKESLDEFIKTIQRR‑HEKGHI‑VTAIFYGVSRSNVQELMGNFSKAFLEH‑R‑D‑‑‑‑‑‑‑SD‑‑QVNQWRNALAEITS‑LPGY‑‑‑ET‑N‑‑‑‑N‑SDYKSVEKIA

>D7MRH5

GEDIRKSFLSHFYKELDRKPI‑LVFKD‑NEIK‑RGISLGPKLKRAIRDSRIAVVIFSRKYASSSWCLNELLEIVRCK‑KEFSQV‑VIPIFFHLDPTHVRKQTGVFGMNFEKTCHNK‑‑‑T‑‑‑EKM‑K‑IRLRRALTEVAN‑ITGY‑‑‑HSVT‑C‑‑K‑NEAKMIEAII

>D7MKA3

GVELRKTFISHLHTRLRRDGINA‑FIDSD‑‑EAPGRELK‑NLFKRIEDSKIALAVLSSRYTESHWCLQELVKMMEC‑SPKENKLLVIPIFYKLKISTVAELDGDFGRNLWDL‑WRLGRD‑‑‑‑‑RDNRIVKWNEALQDVLSRN‑ALVLPE‑‑T‑‑‑‑G‑KEDDFLSTIV

>D7LIG9

GPDVRRGFLSHLHNLFASKGI‑TTFND‑EKID‑RGQPIGPELVQAIRESRVSIVLLSKKYASSSWCLDELLEILKCK‑EDDGQI‑LMTIFYDVNPSHVKKQRGEFGKAFEKTCQGK‑‑‑T‑‑‑EEL‑K‑QRWSKALAHVAT‑IAGE‑‑‑HSLN‑W‑‑P‑YEAEMIQKIA

>D7MKY0

GADVRSHFISYLDPALREANINV‑FIDDD‑‑ELLGSDLV‑HLLKRIEESEIALVIFSEDFTSSYWCLEELAKIKEC‑KDQ‑GRLKVIPIFYKVKPSVVKYLKGKFGDHFRDQ‑IRNLRH‑‑‑‑‑QPERTQKWEDALLSIPESI‑GMPLAA‑‑Q‑‑‑‑S‑EDKDFITSMV

>D7KSY1

GPDVRKTFLSHLRKEFICNGI‑TMFDD‑QGIE‑RGQTISPELTQGIRESRISIVLLSKNYASSSWCLDELLEILKCK‑EDMGQI‑VMTVFYGVNPSDVRKQTGEFGMAFNETCARK‑‑‑T‑‑‑EEE‑R‑RKWSQALNDVGN‑IAGE‑‑‑HFLN‑W‑‑D‑NESKMIEKIA

>D7M0D5

QIND‑‑‑FISHLRAALCRRGISV‑F‑‑‑‑‑‑‑‑N‑ED‑EV‑‑‑DAVPKCRVFIILLTSTYVPS‑‑‑‑‑NLLNILEHQ‑QTEYQA‑VYPIFYRLSPYDLISNSKNYERYFLQ‑‑‑‑‑‑‑‑‑‑‑‑‑N‑‑EPERWQAALKEISQ‑MPGY‑‑‑TL‑D‑‑‑‑‑‑SESELIDEIV

>D7KPJ8

GFDTRANFCERLYVALNEKQVRV‑FRDNEGME‑KGDKIDPSLFEAIEDSAASVIVLSKNYANSAWCLNELALICELR‑SSLKRP‑MIPIFYGVNPSDVRKQSGHF‑KDFEEN‑AKTFD‑‑‑‑‑EE‑‑TIQRWKRAMNLVGN‑IPGF‑‑‑‑VCTE‑‑INDKVDDMIELVV

>D7M0D4

GFDTRNNFTGHLQKALRLRGIDS‑FIDDR‑‑LHRGDNLT‑ALFDRIEKSKIAIIIFSTNYANSAWCLRELVKILEC‑RNR‑NQQLVVPIFYKVEKSDV‑‑‑‑‑‑‑‑‑‑‑KIQ‑ELT‑‑V‑‑‑‑‑SPEEISSWKAALVSASNIL‑GYVVKE‑IS‑‑‑‑T‑SEANLVDEIA

>D7KV07

GEQLRQNFVSHLVEALRRNAINV‑FIDNQ‑‑ELRGEDIS‑ILLKRIEDSRIAIVVFSSRYTESRWCLREAVKIKEC‑VEQ‑DMLKVLPIFYKVTTTGVKQLKGEFGDHFRDR‑EWEYRF‑‑‑‑‑DKPRIERWKEALAFLSGKL‑GLTFDE‑‑K‑‑‑‑S‑SESDFIESIV

>D7KXT5

SHDVETQFMEAILKELHERGITP‑LTN‑‑‑LG‑R‑EL‑NV‑‑‑EMLNRSSVGIMVFSNSYVCSKQSLDHLVAIMEHW‑KAKDIV‑IIPIYFKVTLQHICGLKGMSEAAFLHL‑Q‑S‑‑‑S‑‑‑ED‑‑RVQKWKMALAEIES‑IDGH‑‑‑EW‑K‑‑‑‑‑‑TEVMLAEEVV

>D7MKX2

GEDLRLGFVSHLVEALENDNIKV‑FIDNY‑‑ADKGEPLE‑TLLTKIQESRIALAIFSGKYTESTWCLRELAMIKDC‑VEK‑GNLVAIPIFYKLDPSTVRGVRGQFGDAFRDL‑EER‑‑‑‑‑‑‑‑DVLKKKEWKKALKWVPDLI‑GITVHN‑‑K‑‑‑‑S‑PESEILNEIV

>D7KR53

GLDTRRNFISFLYQELVRRKIRT‑FKDDKELK‑NGQRISPELKRAIEESRFAVVVVSQNYAASRWCLKELVKIMDFE‑NK‑DSITVIPIFYGVEPGHVRWQTGVVAEHFKKH‑ESR‑‑‑E‑‑KHE‑‑KVLQWKQALAAFAQ‑LSGD‑‑‑CS‑G‑‑‑‑D‑DDSKLV‑‑‑A

>Q9CAE0

GPDVRKTLLSNLREHFQGKGI‑TMFDD‑EKIK‑RGGDLSPSLKRAIKTSKISIVILSQKYASSSWCLDELLEIMKRK‑KAMKQI‑VMTVFYGVEPSDVRKQTGDFGIAFNKTCVNK‑‑‑T‑‑‑DKE‑R‑KEWSKALTDVSN‑IAGE‑‑‑DFKK‑W‑‑D‑NEANMIKKIA

>F4I819

GDELRNSFVGFLVKAMRLEKINV‑FTDEV‑‑ELRGTNLN‑YLFRRIEESRVAVAIFSERYTESCWCLDELVKMKEQ‑MEQ‑GKLVVVPVFYRLNATACKRFMGAFGDNLRNL‑EWEYRS‑‑‑‑‑EPERIQKWKEALSSVFSNI‑GLTSDI‑‑R‑‑‑‑S‑N‑SKFVDSIV

>Q8LPH7

GEDVRVTFRSHFLKELDRKLI‑TAFRD‑NEIE‑RSHSLWPDLEQAIKESRIAVVVFSKNYASSSWCLNELLEIVNCN‑D‑‑‑KI‑VIPVFYHVDPSQVRHQIGDFGKIFENTCKRQ‑‑‑T‑‑DEEV‑K‑NQWKKALTLVAN‑MLGF‑‑‑DSAK‑W‑‑N‑DEAKMIEEIA

>Q9C7X0

GPDVRIKFLSHLRQQFVYNGI‑TMFDD‑NGIE‑RSQIIAPALKKAIGESRVAIVLLSKNYASSSWCLDELLEILKCK‑EYIGQI‑VMTVFYEVDPSHVRKQTGDFGIAFKETCAHK‑‑‑T‑‑‑EEE‑R‑SKWSQALTYVGN‑IAGE‑‑‑DFIH‑W‑‑K‑DEAKMIEKIA

>O80617

GEQLRRSFVSHLIDAFERNEINF‑FVDKY‑‑EQRGKDLK‑NLFLRIQESKIALAIFSTRYTESSWCLDELVKIKKL‑ADK‑KKLHVIPIFYKVKVEDVRKQTGEFGDNFWTL‑AKVS‑‑‑‑‑‑‑SGDQIKKWKEALECIPNKM‑GLSLGD‑‑K‑‑‑‑S‑SEADFIKEVV

>F4KG41

GGDVRVTFRSHFLKELDRKLI‑TAFRD‑NEIE‑RSHSLWPDLEQAIKDSRIAVVIFSKNYASSSWCLNELLEIVNCN‑D‑‑‑KI‑VIPVFYGVDPSQVRHQIGDFGKIFEKTCKRQ‑‑‑T‑‑‑EQV‑K‑NQWKKALTDVAN‑MLGF‑‑‑DSAT‑W‑‑D‑DEAKMIEEIA

>F4KD45

GAELRHKFISHLLKALERERINV‑FIDTR‑‑ETMGTGLE‑NLFQRIQESKIAIVVISSRYTESQWCLNELVKIKEC‑VEA‑GTLVVFPVFYKVDVKIVRFLTGSFGEKLETL‑VLR‑‑H‑‑‑‑‑SERY‑EPWKQALEFVTSKT‑GKRVEE‑‑N‑‑‑‑S‑DEGAEVEQIV

>Q9FT77

GEDVRHSLVSHLRKELDRKFI‑NTFND‑NRIE‑RSRKITPELLLAIENSRISLVVFSKNYASSTWCLDELVKIQECY‑EKLDQM‑VIPIFYKVDPSHVRKQTGEFGMVFGETCKGR‑‑‑T‑‑‑ENE‑K‑RKWMRALAEVAH‑LAGE‑‑‑DLRN‑W‑‑R‑SEAEMLENIA

>F4IF04

GKDTRKNFVSFLYKALVSKGIRT‑FKDDEELE‑RGRPIPPELRQAIKGSRIAVVVVSVTYPASSWCLEELREILKLE‑KL‑GLLTVIPIFYEINPSDVRRQSGVVSKQFKKH‑EKR‑‑‑Q‑‑SRE‑‑RVKSWREALTKLAS‑LSGE‑‑‑CS‑KNWGRE‑DDSKLVDGIT

>F4KHH8

GEDVRNTFLSHFLKELDRKLI‑ISFKD‑NEIE‑RSQSLDPELKHGIRNSRIAVVVFSKTYASSSWCLNELLEIVKCK‑KEFGQL‑VIPIFYNLDPSHVRKQTGDFGKIFEKTCRNK‑‑‑T‑‑‑VDE‑K‑IRWKEALTDVAN‑ILGY‑‑‑HIVT‑W‑‑D‑NEASMIEEIA

>F4IFF6

GADVRKSFLSHILKEFKRKGI‑DTFID‑NNIE‑RSKSIGPELIEAIKGSKIAVVLLSKDYASSSWCLNELVEIMKCR‑KMLDQT‑VMTIFYEVDPTDVKKQTGDFGKVFKKTCMGK‑‑‑T‑‑‑NAV‑S‑RKWIEALSEVAT‑IAGE‑‑‑HSIN‑W‑‑D‑TEAAMIEKIS

>F4JNB2

GVDVRKTFLSNLLEAFDRRSI‑NTFMD‑HGIE‑RSRTIAPELISAIREARISIVIFSKNYASSTWCLDELVEIHNRL‑NDWGQL‑VISVFYDVDPSEVRKQTGEFGDVFKKTCEDK‑‑‑E‑‑‑EDQ‑K‑QRWMQALVDITN‑IAGE‑‑‑DLRN‑G‑‑P‑SEAAMVVKIA

>O23536

GVDVRKTFLSHLIEALDRRSI‑NTFMD‑HGIV‑RSCIIADELITAIREARISIVIFSENYASSTWCLNELVEIHKCHDKDLDQM‑VIPVFYGVDPSHVRKQIGGFGDVFKKTCEDK‑‑‑P‑‑‑EDQ‑K‑QRWVKALTDISN‑LAGE‑‑‑DLRN‑G‑‑P‑SEAAMVVKIA

>F4JNB7

GVDVRKTFLSHLIEALDGKSI‑NTFID‑HGIE‑RSRTIAPELISAIREARISIVIFSKNYASSTWCLNELVEIHKCF‑NDLGQM‑VIPVFYDVDPSEVRKQTGEFGKVFEKTCEDK‑‑‑P‑‑‑GDQ‑K‑QRWVQALTDIAN‑IAGE‑‑‑DLLN‑G‑‑P‑NEAHMVEKIS

>F4KFY5

GEDTRHSIVSHLYEALTSRGIAT‑FKDDKRLE‑LGDHISEELQRAIEGSDFVVVVLSENYPTSRWCLMELQSIMELQ‑ME‑GRLGVFPVFYRVEPSAVRYQLGSF‑‑DLEGY‑Q‑‑‑‑‑‑‑‑‑AD‑‑MVPKWRQALKLIAD‑LSGV‑‑‑AS‑GQC‑‑I‑DEATMVRKIV

>F4I552

GPDVRIKFLSHLRQQFIYNGI‑TMFDD‑NGIE‑RSQIIAPALKKAIGESRIAILLLSKNYASSSWSLDELLEILKCK‑EDIGQI‑VMTVFYEVDPSDVRNQTGDFGIAFKETCAHK‑‑‑T‑‑‑EEE‑R‑QKWTQALTYVGN‑IAGE‑‑‑DFKH‑W‑‑P‑NEAKMIEKIA

>O82500

GEDVRNNFLSHLLKEFESKGI‑VTFRD‑DHIK‑RSHTIGHELRAAIRESKISVVLFSENYASSSWCLDELIEIMKCK‑EEQGLK‑VMPVFYKVDPSDIRKQTGKFGMSFLETCCGK‑‑‑T‑‑‑EER‑Q‑HNWRRALTDAAN‑ILGD‑‑‑HPQN‑W‑‑D‑NEAYKITTIS

>Q9FHG0

GEDVRKTFLSHFLRELERKSI‑ITFKD‑NEME‑RSQSIAPELVEAIKDSRIAVIVFSKNYASSSWCLNELLEIMRCN‑KYLGQQ‑VIPVFYYLDPSHLRKQSGEFGEAFKKTCQNQ‑‑‑T‑‑‑EEV‑K‑NQWKQALTDVSN‑ILGY‑‑‑HSKN‑C‑‑N‑SEATMIEEIS

>F4JT81

GADTRHDFTSHLVKYLRGKGIDV‑FSDAK‑‑LRGGEYIS‑LLFDRIEQSKMSIVVFSEDYANSWWCLEEVGKIMQR‑RKE‑FNHGVLPIFYKVSKSDVSNQTGSFEAVFQSP‑TKI‑‑D‑‑‑‑‑EQ‑KIEELKVALKTASNIR‑GFVYPE‑‑N‑‑‑‑S‑SEPDFLDEIV

>Q9SSN9

REDTRT‑FVSHLYRSLDQKEIRT‑YK‑E‑‑‑Q‑QGDKISSEVKQAINESRIAVVVISENYVSSVLCLDVLAKII‑‑‑‑‑ER‑LIKIETVFYEVDPGDLTRPTGKFADDFRRH‑EAR‑‑‑‑‑‑‑‑ENRRVNRWRDALDQVSNNFRNW‑‑EDS‑K‑‑‑IDKPDVDIIASVM

>F4J361

GADVRKTILSHILESFRRKGI‑DPFID‑NNIE‑RSKSIGHELKEAIKGSKIAIVLLSKNYASSSWCLDELAEIMKCR‑ELLGQI‑VMTIFYEVDPTDIKKQTGEFGKAFTKTCKGK‑‑‑T‑‑‑KEY‑V‑ERWRKALEDVAT‑IAGE‑‑‑HSRN‑W‑‑R‑NEADMIEKIA

>F4JWL8

GKDVRRTFLSHLLKEFRRKGI‑RTFID‑NDIK‑RSQMISSELVRAIRESRIAVVVLSRTYASSSWCLNELVEIKK‑‑‑‑‑VSQM‑IMPVFYEVDPSDVRKRTGEFGKAFEEACERQ‑‑‑P‑‑‑DEE‑V‑QKWREALVYIAN‑IAGE‑‑‑SSQN‑W‑‑D‑NEADLIDKIA

>F4J359

GADVRRTFLSHIMESFRRKGI‑DTFID‑NNIE‑RSKSIGPELKEAIKGSKIAIVLLSRKYASSSWCLDELAEIMKCR‑QMVGQI‑VMTIFYEVEPTDIKKQTGEFGKAFTKTCRGK‑‑‑P‑‑‑KEQ‑V‑ERWRKALEDVAT‑IAGY‑‑‑HSHS‑W‑‑R‑NEADMIEKIA

>Q0WQ93

GVDTRQTIVSHLYVALRNNGVLT‑FKDDRKLE‑IGDTIADGLVKAIQTSWFAVVILSENYATSTWCLEELRLIMQLH‑SE‑EQIKVLPIFYGVKPSDVRYQEGSFATAFQRY‑E‑‑‑‑‑‑‑‑‑EE‑‑KVSKWRRALTQVAN‑LSGK‑‑‑HS‑RNC‑‑V‑DEADMIAEVV

>Q9FKB8

GLDTRRTFVSHLRRSLDRKGIKT‑FEDENESL‑RGELDSSAVYQTIGESKVAVVLISVNYASSPLCLDSLLKILKFH‑QS‑GSLVLIPIFYEVDPMDVRKQIGKLYEAF‑SL‑HER‑‑‑E‑‑NPE‑‑KVQTWRQALSQLVS‑IPGG‑‑‑QS‑EIW‑‑D‑GDAELIHQIT

>Q6NPD9

GEKLRDGFLGFLVDALLKENVNV‑FIDDH‑‑ELRGRDLD‑HLFSRIEESRVALTIFSKNFTNSRWCLDELAKIKEC‑VDQ‑ESLTVIPIFFKMKTDDVKKLKGNFGDNFRDL‑KLTHRG‑‑‑‑‑EPETYRRWKDAILYVSKKT‑GLSSSR‑‑Y‑‑‑‑S‑R‑NDLVNTIV

>F4KDB8

GEDVRKSFLSHLLKKLHRKSI‑NTFID‑NNIE‑RSHAIAPDLLSAINNSMISIVVFSKKYASSTWCLNELVEIHKCY‑KELTQI‑VIPIFYEVDPSDVRKQTREFGEFFKVTCVGK‑‑‑T‑‑‑EDV‑K‑QQWIEALEEVAS‑IAGH‑‑‑DSKN‑W‑‑P‑NEANMIEHIA

>Q9FXA6

GPDVRKTFLSHLRKQFNYNGI‑TMFDD‑QRIE‑RSQIIAPALTEAIRESRIAIVLLSKNYASSSWCLDELLEILDCK‑EQLGQI‑VMTVFYGVHPSDVRKQTGDFGIAFNETCARK‑‑‑T‑‑‑EEQ‑R‑QKWSQALTYVGN‑IAGE‑‑‑HFQN‑W‑‑D‑NEAKMIEKIA

>Q9M0P9

GADTRNNIVSYLHKALVDVGIRT‑FKDDKELE‑EGDIISEKLVNAIQTSWFAVVVLSEKYVTSSWCLEELRHIMELS‑IQ‑DDIIVVPIFYKVEPSDVRYQKNSFEVKLQHY‑R‑‑‑‑‑‑‑‑‑‑‑‑‑KILKWKGALTQVGN‑MSGK‑‑‑HF‑QTC‑‑S‑DEATNIAEIV

>A8MR18

GKDLRKGFMSFLKPALKKEKINV‑FIDEQ‑‑EERGKYLI‑SLFDTIGESKIALVIFSEGYCESHWCMDELVKIKEY‑MDQ‑NRLIIIPIFYRLDLDVVKDLTGKFGDNFWDL‑VDKYQP‑‑‑‑‑EPKKLHKWTEALFSVCELF‑SLILPK‑‑H‑‑‑‑S‑DDRDFVKSIV

>O48573

GADLRNGFISHLAGALTSAGITY‑YIDTE‑‑EVPSEDLT‑VLFKRIEESEIALSIFSSNYAESKWCLDELVKIMEQ‑VKK‑GKLRIMPVFFNVKPEEVREQNGEFGLKLYGE‑GKS‑‑‑‑‑‑‑‑KRPNIPNWENALRSVPSKI‑GLNLAN‑‑F‑‑‑‑R‑NEKELLDKII

>F4IBL4

GEDVRRGFLSHIHKEFQRKGI‑TPFID‑NEIK‑RGESIGLEIIHAIRESKIAIVLLSRNYASSSWCLDELVEIMKCK‑EEFSQI‑VIPIFYRVDPSDVKKLTGNFGNVFKNNCVGK‑‑‑T‑‑‑NEV‑I‑RKWRQALAKMGT‑TTGY‑‑‑DSRN‑W‑‑D‑NEATMIENIA

>Q9FHF0

GEDLRLGFVSHLVEALENDNIKV‑FIDNY‑‑ADKGEPLE‑TLLTKIHDSKIALAIFSGKYTESTWCLRELAMIKDC‑VEK‑GKLVAIPIFYKVDPSTVRGVRGQFGDAFRDL‑EER‑‑‑‑‑‑‑‑DVIKKKEWKQALKWIPGLI‑GITVHD‑‑K‑‑‑‑S‑PESEILNEIV

>Q9SKM4

GEQLRRSFVSHLIDAFERNEINF‑FVDKY‑‑EQRGKDLK‑NLFLRIQESKIALAIFSTRYTESSWCMDELVKIKKL‑ADK‑RKLHVIPIFYKVKVEDVRKQTGEFGDNFWTL‑AKVS‑‑‑‑‑‑‑SGDQIKKWKEALECIPNKM‑GLSLGD‑‑K‑‑‑‑S‑DEADFIKEVV

>F4KD49

N‑DSVASFISYLMAGFGCRGIKQ‑‑‑‑‑‑‑‑‑‑‑‑‑‑‑‑‑‑‑‑‑‑‑‑FLGIYLVILSRDYASSVLCLEN‑‑‑‑LELC‑DDKKSYEVVPVFYGVSRSDVRQQSGPFSDAFTKL‑E‑R‑‑‑S‑‑‑AD‑‑HVTKWRRMFAKIAE‑LKGH‑‑‑EY‑E‑‑‑EL‑EESEFVEEIV

>Q9FFS6

GSDVRRKFLSHLRFHFAIKGI‑VAFKD‑QEIE‑RGQRIGPELVQAIRESRVSLVVLSKNYPSSSWCLDELVEILKCK‑EDQEQI‑VMPIFYEIDPSDVRKQSGDFGKAFGKTCVGK‑‑‑T‑‑‑KEV‑K‑QRWTNALTEAAN‑IGGE‑‑‑HSLN‑W‑‑T‑DEAEMIEKIV

>F4I820

GDELRNSFVGFLVKAMRLEKINV‑FTDEV‑‑ELRGTNLN‑YLFRRIEESRVAVAIFSERYTESCWCLDELVKMKEQ‑MEQ‑GKLVVVPVFYRLNATACKRFMGAFGDNLRNL‑EWEYRS‑‑‑‑‑EPERIQKWKEALSSVFSNI‑GLTSDI‑‑R‑‑‑‑S‑N‑SKFVDSIV

>F4JWM0

GEDVRKGFLSHIQKEFKSKGI‑VPFID‑DEMK‑RGESIGPGLFQAIRESKIAIVLLSKNYASSSWCLNELVEIMNCR‑EEIGQT‑VMTVFYQVDPSDVRKQTGDFGKAFKKTCVGK‑‑‑T‑‑‑QEV‑K‑QRWSRALMDVAN‑ILGQ‑‑‑DSRK‑W‑‑D‑KEADMIVKVA

>F4JNL2

GKDERNGLLTLLKQKLIDGNVNV‑FTDD‑‑‑KLTGQPLQ‑NLFGHIRKSRIAIVIFSKNYAESGWCLDELVEIKKC‑FET‑ELKAVIPIFHRVKVSSVKKQSGKFGEKFLAL‑QKKIKR‑‑‑‑‑INSRIKRWKKALKIVTEIA‑GL‑‑‑D‑‑K‑‑‑‑N‑SELAFVEKVV

>O81430

VEDIRQTFLSHFLKDLDRKLI‑IAFKD‑NEIE‑RSQSLNPDLKRPIRDSRIAIVIFSKNYASSSWCLNELLEIVRCK‑EDSNLV‑VIPVFYGLDPSHVRKQIGNFGKIFKKTCQNR‑‑‑T‑‑‑EDE‑I‑NLRRRALIDVAN‑TLGY‑‑‑HSTI‑C‑‑‑‑‑KANMTKEIT

>F4IEZ7

GVDTRQTIVSHLYVALRNNGVLT‑FKDDRKLE‑IGDTIADGLVKAIQTSWFAVVILSENYATSTWCLEELRLIMQLH‑SE‑EQIKVLPIFYGVKPSDVRYQEGSFATAFQRY‑E‑‑‑‑‑‑‑‑‑EE‑‑KVSKWRRALTQVAN‑LSGK‑‑‑HS‑RNC‑‑V‑DEADMIAEVV

>F4JYI5

GPDVRKGFLSHLHSLFASKGI‑TTFND‑QNIE‑RGQTIGPELIQGIKEARVSIVVLSKNYASSSWCLDELVEILKCK‑EALGQI‑VMT‑‑‑‑‑‑‑‑‑‑‑‑‑SGVFGKAFEKTCQGK‑‑‑N‑‑‑EEV‑K‑IRWRNALAHVAT‑IAGE‑‑‑HSLN‑W‑‑D‑NEAKMIQKIA

>Q9SCZ3

SEDTRYSFVSHLCAAFRRRGISS‑FIEDS‑‑‑‑‑‑NF‑SK‑‑‑‑‑LETSRASVVVFSEKYSSSKSCMEELVKVSERR‑RKNCLA‑VVPVFYPVTKSFMKKQIWNLG‑‑‑‑‑‑‑‑‑‑‑‑‑‑‑‑‑‑D‑‑VRSDWPSALLETVD‑LPGH‑‑‑EL‑D‑‑‑‑T‑SDSDFVEEIV

>Q9FLA7

GNESRDNFIKYLVWGLRDERVNV‑FVDRA‑‑EANRRDIR‑NISTKIEESNIAVVIFSKRYTESEMCLNELQKMYEH‑VEQ‑SNLKVIPVFYDVSISGVKNLEDEFGNHFEEL‑REK‑‑‑‑‑‑‑‑DPLKILKWEDSLSSIVE‑‑‑GLTSED‑‑H‑‑‑‑G‑TGLGLVRAIV

>Q9SSN3

GHDTRHNFISFLYKELVRRSIRT‑FKDDKELE‑NGQRFSPELKSPIEVSRFAVVVVSENYAASSWCLDELVTIMDFE‑KK‑GSITVMPIFYGVEPNHVRWQTGVLAEQFKKH‑ASR‑‑‑E‑‑DPE‑‑KVLKWRQALTNFAQ‑LSGD‑‑‑CS‑G‑‑‑‑D‑DDSKLVDKIA

>F4I270

GDDVRRNFLSHIQKEFRRKGI‑TPFID‑NEIR‑RGESIGPELIKAIRESKIAIVLLSRNYASSKWCLEELVEIMKCK‑KEFGLT‑VFAIFYEVDPSHVKKLTGEFGAVFQKTCKGR‑‑‑T‑‑‑KEN‑I‑MRWRQAFEEVAT‑IAGY‑‑‑DSRN‑W‑‑E‑NEAAMIEEIA

>Q9FKR7

GPDVRSGFLSHLHNHFESKGI‑TPFKD‑QEIE‑RGHTIGPELIQAIRESRVSIVVLSEKYASSCWCLDELVEILKCK‑EASGQV‑VMTIFYKVDPSDVRKQRGDFGSTFKKTCEGK‑‑‑T‑‑‑WIV‑K‑QRWIKALEYIAT‑VAGE‑‑‑HSLS‑W‑‑A‑NEAELIQKIA

>Q9SSN1

GLDTRRSFISFLYKELIRRNIRT‑FKDDKELK‑NGRRITPELIRAIEGSRFAVVVVSVNYAASRWCLEELVKIMDFE‑NM‑GSLKVMPIFYGVDPCHVRRQIGEVAEQFKKH‑EGR‑‑‑E‑‑DHE‑‑KVLSWRQALTNLAS‑ISGD‑‑‑CS‑WKW‑‑E‑DDSKMVEEIT

>Q9C5Q9

GKDLRKGFMSFLKPALKKEKINV‑FIDEQ‑‑EERGKYLI‑SLFDTIGESKIALVIFSEGYCESHWCMDELVKIKEY‑MDQ‑NRLIIIPIFYRLDLDVVKDLTGKFGDNFWDL‑VDKYQP‑‑‑‑‑EPKKLHKWTEALFSVCELF‑SLILPK‑‑H‑‑‑‑S‑DDRDFVKSIV

>F4K382

GLDTRRTFVSHLRRSLDRKGIKT‑FEDENESL‑RGELDSSAVYQTIGESKVAVVLISVNYASSPLCLDSLLKILKFH‑QS‑GSLVLIPIFYEVDPMDVRKQIGKLYEAF‑SL‑HER‑‑‑E‑‑NPE‑‑KVQTWRQALSQLVS‑IPGG‑‑‑QS‑EIW‑‑D‑GDAELIHQIT

>Q9CAK1

GPDVRKTLLSHIRLQFNRNGI‑TMFDD‑QKIV‑RSATIGPSLVEAIKESRISIVILSKKYASSSWCLDELVEILECK‑KAMGQI‑VMTIFYGVDPSDVRKQIGKFGIAFNETCARK‑‑‑T‑‑‑EEE‑R‑QKWSKALNQVSN‑IAGE‑‑‑DFLR‑W‑‑D‑NEAIMIEKIA

>F4IF00

GEDTRKNIVSHLHKQLVDKGVVT‑FKDDKKLE‑LGDSISEEISRAIQNSTYALVILSENYASSSWCLDELRMVMDLH‑LK‑NKIKVVPIFYGVDPSHVRHQTGSF‑‑TFDKY‑Q‑‑‑‑‑‑‑‑‑PN‑‑KVTTWREALTQIAS‑LAGK‑‑‑DF‑ETC‑‑E‑DEASMIEEIV

>Q9FHE5

EEDVSKGLINFLEPVLQNKNINV‑FIDEE‑‑EVRGKGLK‑NLFKRIQDSKISLAIFSESKCD‑‑‑‑FNDLLKNNES‑ADE‑‑‑‑‑AIPIFYKVDAT‑‑‑‑‑‑‑‑‑GD‑‑‑‑‑‑‑‑‑‑‑‑‑‑‑‑‑‑‑‑‑‑‑‑‑‑‑‑‑‑‑‑‑‑‑‑‑‑‑‑LAD‑‑L‑‑‑‑Q‑NKKDLINSAV

>Q9FVT6

GKDIRHGFVSHLKDALKRKNINF‑FIDTH‑‑EQKGRDLN‑HLFKRIEEATIALVILSPRYGESKWCLEELTTIMDQ‑EEK‑GQMIVIPIFYKVRTEDVEKQTGEFGHMFWSC‑DEEA‑‑‑‑‑‑‑SLEEMEKWQVALKAVCNKI‑GLTLDL‑‑K‑‑‑‑‑‑‑EAKFIKKVL

>F4I3S8

GPDVRKTLLSHMRKQFDFNGI‑TMFDD‑QGIE‑RSEEIAPSLKKAIKESRISIVILSKKYASSSWCLDELVDILKRK‑KAMKQI‑VMTVFYGVEPFEVRNQTGEFGIAFNETCARK‑‑‑T‑‑‑DEE‑R‑QKWSKALNEVAN‑IAGE‑‑‑DFLR‑C‑‑D‑NEAKRIEKIA

>O49471

GDELRNNFVSHLDKALRGKQINV‑FIDEA‑‑VEKGENLD‑NLFKEIEKSRIALAIISQKYTESKWCLNELVKMKEL‑‑‑E‑GKLVTIPIFYNVEPATVRYQKEAFGAALTKT‑QEN‑DS‑‑‑‑‑DG‑QMKKWKEALTYVSLLV‑GFPFNS‑‑K‑‑‑‑S‑KEKELIDKIV

>Q9SSN6

GPDTRRKFISFLYKELVGRDIRT‑FKDDKELE‑NGQMISPELILAIEDSRFAVVVVSVNYAASSWCLDELVKIMDIQ‑NK‑GSITVMPIFYGVNPCHLRRQIGDVAEQFKKH‑EAR‑‑‑E‑‑DLE‑‑KVLKWRQALAALAD‑ISGD‑‑‑CS‑G‑‑‑‑E‑DDSKLVDVIA

>Q9C7W9

GPDVRIKFLSHLRQQFIYNGI‑TMFDD‑NGIE‑RSQIIAPALKKAIGESRIAILLLSKNYASSSWSLDELLEILKCK‑EDIGQI‑VMTVFYEVDPSDVRNQTGDFGIAFKETCAHK‑‑‑T‑‑‑EEE‑R‑QKWTQALTYVGN‑IAGE‑‑‑DFKH‑W‑‑P‑NEAKMIEKIA

>F4I901

GEDTRKTIVSHLYAALDSRGIVT‑FKDDQRLE‑IGDHISDELHRALGSSSFAVVVLSENYATSRWCLLELQLIMELM‑KE‑GRLEVFPIFYGVDPSVVRHQLGSF‑‑SLVKY‑Q‑‑‑‑‑‑‑‑‑VD‑‑KVLRWREALNLIAN‑LSGV‑‑‑VS‑SHC‑‑V‑DEAIMVGEIA

>F4I9F1

GEDVRVTFRSHFLKELDRKLI‑TAFRD‑NEIE‑RSHSLWPDLEQAIKESRIAVVVFSKNYASSSWCLNELLEIVNCN‑D‑‑‑KI‑VIPVFYHVDPSQVRHQIGDFGKIFENTCKRQ‑‑‑T‑‑DEEV‑K‑NQWKKALTLVAN‑MLGF‑‑‑DSAK‑W‑‑N‑DEAKMIEEIA

>F4HT77

GEEIRHGFISHLADALERYGIMF‑IIDKD‑‑EQRGNDLT‑SLLLRIKESKVALVIFSSRFAESRFCMDEIVKMKEC‑VDE‑RKLLVIPIFYKVRARDVSGRTGDFGKKFWAL‑AQKS‑‑‑‑‑‑‑RGCQIKEWMEALECISNKM‑GLSLGD‑‑G‑‑‑‑R‑SEADFIKEIV

>Q9FNJ2

GEDVRVTFLSHFLKELDRKLI‑SVFKD‑NDIQ‑RSQSLDPELKLAIRDSRIAIVVFSKNYAASSWCLDELLEIVKCK‑EEFGQI‑VIPVFYGLDPCHVRKQSGEFGIVFENTCQTK‑‑‑T‑‑‑DDE‑I‑QKWRRALTDVAN‑ILGF‑‑‑HSSN‑W‑‑D‑NEATMVEDIA

>Q9CAK0

GPNVRKTLLSHMRKQFNFNGI‑TMFDD‑QGIE‑RSEEIVPSLKKAIKESRISIVILSKKYALSRWCLDELVEILKCK‑EVMGHI‑VMTIFYGVEPSDVRKQTGEFGFHFNETCAHR‑‑‑T‑‑‑DED‑K‑QNWSKALKDVGN‑IAGE‑‑‑DFLR‑W‑‑D‑NEAKMIEKIA

>Q9SYC9

GDELRNSFVGFLVKAMRLEKINV‑FTDEV‑‑ELRGTNLN‑YLFRRIEESRVAVAIFSERYTESCWCLDELVKMKEQ‑MEQ‑GKLVVVPVFYRLNATACKRFMGAFGDNLRNL‑EWEYRS‑‑‑‑‑EPERIQKWKEALSSVFSNI‑GLTSDI‑‑R‑‑‑‑R‑YINKNMDHTS

>F4IUF0

SKDTRDNFVSHLCGCLRRKRIKT‑FLD‑‑ELE‑R‑EE‑‑‑SLK‑AIE‑SKISVIVFSENFGDSRWCLDEVVAILKCK‑EK‑GQI‑VIPVLYHVDPLDIENQTGSFGDAFAKR‑RDK‑‑‑A‑‑‑‑E‑‑QLQEWKDSFTE‑AN‑LPGW‑‑‑‑STA‑Y‑SD‑‑EEMLVNGIA

>O65507

GKQLRNGFVSHLEKALRRDGINV‑FIDRN‑‑ETKGRDLS‑NLFSRIQESRIALAIFSSMYTESYWCLDELVKIKDC‑VDL‑GTLVVIPIFYMVDTDDVKNLKGAFGYTFWKL‑AKT‑‑C‑‑‑‑‑NGEKLDKWKQALKDVPKKL‑GFTLSE‑‑M‑‑‑‑S‑DEGESINQIV

>Q9FHE9

GKDLRNGFLSFLEPAMREANINV‑FIDKD‑‑EVVGTDLV‑NLFVRIQESRVAVVIFSKDYTSSEWCLDELAEIKDC‑INQ‑GGLNAIPIFYKLAPSSVLELKGGFGDTFRVL‑KEKYKN‑‑‑‑‑DPERTQKWQEALESIPKLK‑GLRLAE‑‑K‑‑‑‑S‑DEREFMNEMI

>F4HR52

‑RDARHKFTERLYEVLVKEQVRV‑W‑NNDDVE‑RGNELGASLVEAMEDSVALVVVLSPNYAKSHWCLEELAMLCDLK‑SSLGRL‑VLPIFYEVEPCMLRKQNGPY‑MDFEEH‑SKRFS‑‑‑‑‑EE‑‑KIQRWRRALNIIGN‑IPGF‑‑‑‑VYSK‑‑‑‑SKDDDMIELVV

>Q9ZVX6

GPDVRKSFLSHFRKQFICNGI‑TMFDD‑QKIV‑RSQTIAPSLTQGIRESKISIVILSKNYASSTWCLNELLEILKCR‑EDIGQI‑VMTVFYGVDPSDVRKQTGEFGTVFNKTCARR‑‑‑T‑‑‑EKE‑R‑RNWSQALNVVGN‑IAGE‑‑‑HFLN‑W‑‑D‑NEAEMIEKIA

>Q9SSP1

SEDPSKTFVSVLDRWLEQKDITTNFKDD‑‑‑‑‑‑‑‑‑‑‑SFLAE‑‑‑ESKLAVVVVSESYPISVLCLNQLEKIVNSH‑SE‑GRLSILPIFYGVDPYNVRKQTGYLAEPFQEL‑GEG‑‑‑Y‑‑PDD‑‑KIQEWRVSLTKLTN‑IPAL‑‑‑DS‑RYW‑‑S‑NEADMIELIA

>Q9FGT2

GPDVRKTFLSHLRKQFSYNGI‑SMFND‑QSIE‑RSQTIVPALTGAIKESRISIVVLSKNYASSRWCLDELLEILKCR‑EDIGQI‑VMTVFYGVDPSDVRKQTGEFGIAFNKTCEGK‑‑‑T‑‑‑NEE‑T‑QKWSKALNDVGN‑IAGE‑‑‑HFFN‑W‑‑D‑NEAKMIEKIA

>Q0WPW2

GEDVRKSFLSHLLKKLHRKSI‑NTFID‑NNIE‑RSHAIAPDLLSAINNSMISIVVFSKKYASSTWCLNELVEIHKCY‑KELTQI‑VIPIFYEVDPSDVRKQTREFGEFFKVTCVGK‑‑‑T‑‑‑EDV‑K‑QQWIEALEEVAS‑IAGH‑‑‑DSKN‑W‑‑P‑NEANMIEHIA

>Q9SW60

GEDIRVTFLTHFLKELDRKMI‑IAFKD‑NEIE‑RGNSIGTELIQAIKDSRIAVVVFSKKYSSSSWCLNELVEIVNCK‑‑‑‑‑EI‑VIPVFYDLDPSDVRKQEGEFGESFKETCKNR‑‑‑T‑‑‑DYE‑I‑QRWGQALTNVAN‑IAGY‑‑‑HTRK‑P‑‑N‑NEAKLIEEIT

>Q9FMB7

GGDIRKTFLSHLRKQFNSNGI‑TMFDD‑QGIE‑RSQTIAPALIQAIRESRISIVVLSKNYASSSWCLNELVEILKCK‑D‑‑‑‑V‑VMPIFYEVDPSDVRKQTGDFGKAFKNSCKSK‑‑‑T‑‑‑KEE‑R‑QRWIQALIFVGN‑IAGE‑‑‑HSLK‑W‑‑E‑NEADMIEKIA

>Q9FKB9

GEDTRRTIVSHLYAALGAKGIIT‑FKDDQDLE‑VGDHISSHLRRAIEGSKFAVVVLSERYTTSRWCLMELQLIMELY‑NL‑GKLKVLPLFYEVDPSDVRHQRGSF‑‑GLERY‑Q‑‑‑‑‑‑‑‑‑AD‑‑IVQRWRVALCMVAN‑LSGM‑‑‑VS‑RYC‑‑A‑DEAMMLEEIV

>F4JT79

NLDVETSFIEAISKELHKQGFIP‑LTN‑‑‑LG‑R‑EL‑DE‑‑‑EMLYGSRVGIMILSSSYVSSRQSLDHLVAVMEHW‑KTTDLV‑IIPIYFKVRLSDICGLKGRFEAAFLQL‑H‑M‑‑‑S‑‑‑ED‑‑RVQKWKAAMSEIVS‑IGGH‑‑‑EW‑K‑‑‑‑‑‑SQFILAEEVV

>F4I818

GDELRNSFVGFLVKAMRLEKINV‑FTDEV‑‑ELRGTNLN‑YLFRRIEESRVAVAIFSERYTESCWCLDELVKMKEQ‑MEQ‑GKLVVVPVFYRLNATACKRFMGAFGDNLRNL‑EWEYRS‑‑‑‑‑EPERIQKWKEALSSVFSNI‑GLTSDI‑‑R‑‑‑‑S‑N‑SKFVDSIV

>Q9FHE8

GADVRKHFISFLVPALREANINV‑FIDEN‑‑EFLGSEMA‑NLLTRIEESELALVIFSVDFTRSHRCLNELAKIKER‑KDQ‑GRLIVIPIFYKVKPSAVKFLEGKFGDNFRAL‑ERNNRH‑‑‑‑‑MLPITQKWKEALESIPGSI‑GMPLAE‑‑Q‑‑‑‑S‑EDNDFINSMV

>F4ISS7

GKELRKGFISFLVPALKKKNINV‑FIDEH‑‑EVRGKDLI‑SLFRRIGESKIALVIFSEGYTESKWCLDELVQIKKC‑VDQ‑KKIIAIPIFYKLDPAVVKGLKGKFGDKFRDL‑IERYHH‑‑‑‑‑EPERYQKWTEALTSVSRTF‑ALCLPE‑‑H‑‑‑‑S‑DEKDFIRSII

>Q9LSX5

GPDVRKGFLSHLHSVFASKGI‑TTFND‑QKID‑RGQTIGPELIQGIREARVSIVVLSKKYASSSWCLDELVEILKCK‑EALGQI‑VMTVFYEVDPSDVKKQSGVFGEAFEKTCQGK‑‑‑N‑‑‑EEV‑K‑IRWRNALAHVAT‑IAGE‑‑‑HSLN‑W‑‑D‑NEAKMIQKIV

>Q9FH20

GEDVRGNFLSHLMKEFESKGI‑VTFKD‑DLIE‑RSQTIGLELKEAVRQSKIFVVIFSKNYASSSWCLDELVEILKCK‑EERRL‑‑‑IPIFYKVNPSDVRNQTGKFGRGFRETCEGK‑‑‑N‑‑‑DET‑Q‑NKWKAALTEAAN‑IAGE‑‑‑DSQS‑W‑‑K‑NEADFLTKIA

>F4JWL7

GEDVRKGFLSHIQKEFERKGI‑FPFVD‑TKMK‑RGSSIGPVLSDAIIVSKIAIVLLSKNYASSTWCLNELVNIMKCR‑EEFGQT‑VMTVFYEVDPSDVRKQTGDFGIAFETTCVGK‑‑‑T‑‑‑EEV‑K‑QSWRQALIDVSN‑IVGE‑‑‑VYRI‑W‑‑S‑KESDLIDKIA

>Q9FL35

GGDVRVTFRSHFLKEFDRKLI‑TAFRD‑NEIE‑RSHSLWPDLEQAIKESRIAVVVFSKNYASSSWCLNELLEIVNCN‑D‑‑‑KI‑IIPVFYGVDPSQVRYQIGEFGKIFEKTCKRQ‑‑‑T‑‑‑EEV‑K‑NQWKKALTHVAN‑MLGF‑‑‑DSSK‑W‑‑D‑DEAKMIEEIA

>Q9XGM3

GADLRRRFVSHLVTALKLNNINV‑FIDDY‑‑EDRGQPLD‑VLLKRIEESKIVLAIFSGNYTESVWCVRELEKIKDC‑TDE‑GTLVAIPIFYKLEPSTVRDLKGKFGDRFRSM‑AKG‑‑‑‑‑‑‑‑DE‑RKKKWKEAFNLIPNIM‑GIIIDK‑‑K‑‑‑‑S‑VESEKVNEIV

>F4IF05

GKDTRKNFVSFLYKALVSKGIRT‑FKDDEELE‑RGRPIPPELRQAIKGSRIAVVVVSVTYPASSWCLEELREILKLE‑KL‑GLLTVIPIFYEINPSDVRRQSGVVSKQFKKH‑EKR‑‑‑Q‑‑SRE‑‑RVKSWREALTKLAS‑LSGE‑‑‑CS‑KNW‑‑E‑DDSKLVDGIT

>Q9SZ66

GFDTRNNFTGHLQKALRLRGIDS‑FIDDR‑‑LRRGDNLT‑ALFDRIEKSKIAIIVFSTNYANSAWCLRELVKILEC‑RNS‑NQQLVVPIFYKVDKSDVEKQRNSFAVPFKLP‑ELT‑‑V‑‑‑‑‑TPEEISSWKAALASASNIL‑GYVVKE‑IS‑‑‑‑T‑SEAKLVDEIA

>Q93ZC0

GGDVRVTFRSHFLKEFDRKLI‑TAFRD‑NEIE‑RSHSLWPDLEQAIKDSRIAVVVFSKNYASSSWCLNELLEIVNCN‑D‑‑‑KI‑IIPVFYGVDPSQVRYQIGDFGRIFEKTCKRQ‑‑‑T‑‑‑EEV‑K‑NQWKKALTLVAN‑MLGF‑‑‑DSAK‑W‑‑D‑DEAKMIEEIA

>F4KIF3

GVELRKNFVSHLEKGLKRKGINA‑FIDTD‑‑EEMGQELS‑VLLERIEGSRIALAIFSPRYTESKWCLKELAKMKER‑TEQ‑KELVVIPIFYKVQPVTVKELKGDFGDKFREL‑VKS‑‑T‑‑‑‑‑DKKTKKEWKEALQYVPFLT‑GIVLDE‑‑K‑‑‑‑S‑DEDEVINIII

>Q9M1P1

GADVRRTFLSHIMESFRRKGI‑DTFID‑NNIE‑RSKSIGPELKEAIKGSKIAIVLLSRKYASSSWCLDELAEIMKCR‑QMVGQI‑VMTIFYEVEPTDIKKQTGEFGKAFTKTCRGK‑‑‑P‑‑‑KEQ‑V‑ERWRKALEDVAT‑IAGY‑‑‑HSHS‑W‑‑R‑NEADMIEKIA

>F4J3L8

GEDVRKDFLSHIQKEFQRQGI‑TPFVD‑NNIK‑RGESIGPELIRAIRGSKIAIILLSKNYASSSWCLDELVEIIKCK‑EEMGQT‑VIVIFYKVDPSLVKKLTGDFGKVFRNTCKGK‑‑‑E‑‑‑REN‑I‑ERWREAFKKVAT‑IAGY‑‑‑DSRK‑W‑‑D‑NESGMIEKIV

>F4JNB8

GVDVRKTFLSHLIEALDGKSI‑NTFID‑HGIE‑RSRTIAPELISAIREARISIVIFSKNYASSTWCLNELVEIHKCF‑NDLGQM‑VIPVFYDVDPSEVRKQTGEFGKVFEKTCEDK‑‑‑P‑‑‑GDQ‑K‑QRWVQALTDIAN‑IAGE‑‑‑DLLN‑G‑‑P‑NEAHMVEKIS

>Q9FI14

GEDVRKGLLSHIQKEFQRNGI‑TPFID‑NEMK‑RGGSIGPELLQAIRGSKIAIILLSRNYGSSKWCLDELVEIMKCR‑EELGQT‑VMTVFYDVDPSDVRKQKGDFGKVFKKTCVGR‑‑‑P‑‑‑EEM‑V‑QRWKQALTSAAN‑ILGE‑‑‑DSRN‑W‑‑E‑NEADMIIKIS

>F4J339

GADVRRTFLSHIMESFRRKGI‑DTFID‑NNIE‑RSKSIGPELKEAIKGSKIAIVLLSRKYASSSWCLDELAEIMKCR‑QMVGQI‑VMTIFYEVDPTDIKKQTGEFGKAFTKTCRGK‑‑‑P‑‑‑KEQ‑V‑ERWRKALEDVAT‑IAGY‑‑‑HSHS‑W‑‑R‑NEADMIEKIS

>Q9M0Q0

VAET‑‑‑LVSDLRSSFSENGIT‑‑MKDDDLEK‑GVSSLGSERSEGIRESKVAVVVISQSYAISAQCLNELQTIVNFH‑DE‑RRISILPIFYGVDYDDVRNQIKELAASFRKL‑GKE‑‑‑Y‑‑PSE‑‑KVQAWMIALIKLIN‑ISRS‑‑‑DS‑RIH‑‑D‑DETTIDMVIT

>Q9SSN5

GHDTRQNFISFLYKELVRRSIRT‑FKDDKELE‑NGQRISSELKRTIEVSRFAVVVVSETYAASSWCLDELVTIMDFE‑KK‑GSITVMPIFYGVEPNHVRWQTGVLAEQFKKH‑GSR‑‑‑E‑‑DHE‑‑KVLKWRQALTNFAQ‑LSGD‑‑‑CS‑G‑‑‑‑D‑DDSKLVDKIA

>Q0WSX8

GDELREIFVNHLELQLRNAGINV‑FIDTK‑‑EQKGRRLQ‑YLFTRIKKSKIALAIFSKRYCESKWCLDELVTMNEQ‑MKE‑KKLVVIPIFYNVRSDDVKNLDGEFSLPFKQL‑KQN‑‑‑‑‑‑‑‑EPERVEGWERALRSVTK‑F‑SRSNSK‑‑Y‑‑‑‑K‑HDTDFVLDIV

>F4KHI3

GKDVRVTFRSHFLKELDRKLI‑SAFRD‑NEIE‑RSHSLWPDLEQAIKDSRIAVVVFSKNYASSSWCLNELLEIVNCN‑D‑‑‑KI‑IIPVFYGVDPSQVRYQIGEFGSIFEKTCKRQ‑‑‑T‑‑‑EEV‑K‑NQWKKALTDVAN‑MLGF‑‑‑DSAK‑W‑‑D‑DEAKMIEEIA

>Q4PT31

GKDTRRTFISFLYKELIEMGIRT‑FKDDVELQ‑SGRRIASDLLAAIENSKIAVVIISKNYSASPWCLQELVMIMDVE‑KK‑GSIIVMPIFYNVEPAHVRRQIEQVAKQFRKH‑EGR‑‑‑E‑‑NYE‑‑TVVSWRQALTNLAS‑ISGH‑‑‑CS‑RDC‑‑E‑DDSKLLDEIT

>F4I3S7

GPDVRKTLLSHMRKQFDFNGI‑TMFDD‑QGIE‑RSEEIAPSLKKAIKESRISIVILSKKYASSSWCLDELVDILKRK‑KAMKQI‑VMTVFYGVEPFEVRNQTGEFGIAFNETCARK‑‑‑T‑‑‑DEE‑R‑QKWSKALNEVAN‑IAGE‑‑‑DFLR‑C‑‑D‑NEAKRIEKIA

>B3H776

GADVRKNFLSHLYDSLRRCGIST‑FMDDVELQ‑RGEYISPELLNAIETSKILIVVLTKDYASSAWCLDELVHIMKSH‑KNNSHM‑VFPIFLYVDPSDIRWQQGSYAKSFSKH‑KNSHPL‑‑‑‑‑‑‑‑KLKDWREALTKVAN‑ISGW‑‑‑‑DIK‑‑‑‑N‑NEAECIADIT

>Q9FFS5

GPDVRKGFLSHLHYHFASKGI‑TTFKD‑QEIE‑KGNTIGPELVNAIRESRVSIVLLSKKYASSSWCLDELVEILKCK‑EDQGQI‑VMTIFYDVDPSSVRKQKGDFGSTFMKTCEGK‑‑‑S‑‑‑EEV‑K‑QRWTKALTHVAN‑IKGE‑‑‑HSLN‑W‑‑A‑NEADMIQKIA

>Q9FJG4

GEDVRRNFLSHLHKELQHNGI‑DAFKD‑GGIK‑RSRSIWPELKQAIWESKIFIVVLSKNYAGSCWCLDELVEIMECR‑EVVGKT‑LVPIFYDVDPSSVRKQTGDFGKAFDKICDVR‑‑‑T‑‑‑EEE‑R‑QRWRQALTNVGN‑IAGE‑‑‑CSSK‑W‑‑D‑NDAKMIEKIV

>F4JU10

GNDLRKGFVSHVVKALKDARVNV‑FVDNDQWERRGHDDH‑LFVRRIHNSKLALVIFSDQYAESQQCLNELTTIHER‑VAE‑GKLMVIPIFYKVNIEEVNNLEGRFGKCFDEM‑VRTQGR‑‑‑‑‑QNHQLHHIVGCLRSIARKP‑SF‑‑‑‑‑‑T‑‑‑‑S‑GDSDLVEAII

>F4JNA9

GVDVRKTFLSHLIEALDRRSI‑NTFMD‑HGIV‑RSCIIADALITAIREARISIVIFSENYASSTWCLNELVEIHKCY‑KKGEQM‑VIPVFYGVDPSHVRKQIGGFGDVFKKTCEDK‑‑‑P‑‑‑EDQ‑K‑QRWVKALTDISN‑LAGE‑‑‑DLRN‑G‑‑P‑TEAFMVKKIA

>Q9C515

GPDVRNTFLSHLRKQFNTNGI‑TMFDD‑QRME‑RSQTLAPTLTQAIRESKIYIVLLSKNYASSSWCLDELLEILNCK‑EKRGQR‑VMTIFYGVNPSDVRKQTGEFGIAFNETCARK‑‑‑T‑‑‑EEE‑R‑RKWSHALTCVGN‑ITGV‑‑‑HVQD‑R‑‑D‑DEANMIEKIA

>F4JU09

GKELRHGFVSHVVKALRIAGVNV‑FIDSN‑‑EMKGRDLQ‑NLFKRIENSKMALVIFSDRFSESDWCLNELVKIDDC‑VKE‑GKLTVIPVFYRVNTDDVKNFKGKFGSCFIET‑VQRQSP‑‑‑‑‑KEEPMERWVNSVKSISSKT‑GF‑‑‑‑‑‑T‑‑‑‑S‑EDSYLVDAIV

>Q9SS05

GEDVRRGFLSHIHKEFQRKGI‑TPFID‑NEIK‑RGESIGLEIIHAIRESKIAIVLLSRNYASSSWCLDELVEIMKCK‑EEFSQI‑VIPIFYRVDPSDVKKLTGNFGNVFKNNCVGK‑‑‑T‑‑‑NEV‑I‑RKWRQALAKMGT‑TTGY‑‑‑DSRN‑W‑‑D‑NEATMIENIA

>F4JYI4

GPDVRKGFLSHLHSLFASKGI‑TTFND‑QNIE‑RGQTIGPELIQGIKEARVSIVVLSKNYASSSWCLDELVEILKCK‑EALGQI‑VMT‑‑‑‑‑‑‑‑‑‑‑‑‑SGVFGKAFEKTCQGK‑‑‑N‑‑‑EEV‑K‑IRWRNALAHVAT‑IAGE‑‑‑HSLN‑W‑‑D‑NEAKMIQKIA

>F4IMF2

GSELRYTFVYYLRTALVKNGINV‑FTDNM‑‑EPKGRNQK‑ILFKRIEESKIALAIFSSRYTESSWCLEELVKMKEC‑MDA‑EKLVIIPIFYIVTPYTIKKQMGDFGDKFRVL‑VDY‑‑V‑‑‑‑‑DDVTEKKWTDALKSVPLIL‑GITYDG‑‑Q‑‑‑‑S‑EEQLLINQIV

>Q9SSN4

GLDTRRNFISFLYKELVRRKIRT‑FKDDKELE‑NGRRISPELKRAIEESKFAVVVVSVNYAASPWCLDELVKIMDFE‑NK‑GSITVMPIFYGVDPCHLRRQIGDVAEQFKKH‑EAR‑‑‑E‑‑DHE‑‑KVASWRRALTSLAS‑ISGE‑‑‑CS‑LKW‑‑E‑DEANLVDEIA

>Q9FKE4

SGIERNSFVSHLSAAFRRRSVSV‑CLGDV‑‑‑‑‑‑‑‑‑‑R‑‑‑KTNEGCKVFVVVFSEDYALSKQCLDTLVEF‑‑LE‑KDDGLV‑IVPVYYGVTESMVKQQTERFGVAFTQH‑Q‑N‑‑‑N‑‑‑YD‑‑QVAKWRDCLIQTAS‑LPGH‑‑‑EL‑L‑‑‑‑Q‑EDSEFVEKIV

>Q9FHF4

GTDVRRNFLSHLLKGL‑HKSV‑NSFRD‑QNME‑RSQSLDPMLKQAIRDSRIALVVFSKNYASSSWCLNELLEIVKCK‑EEFGQM‑VIPIFYCLDPSHVRHQDGDFGKNFEETCGRN‑‑‑T‑‑‑EEE‑K‑IQWEKALTDVAN‑LAGF‑‑‑DSVT‑W‑‑D‑DEAKMIEEIA

>F4KBQ6

GDELREIFVNHLELQLRNAGINV‑FIDTK‑‑EQKGRRLQ‑YLFTRIKKSKIALAIFSKRYCESKWCLDELVTMNEQ‑MKE‑KKLVVIPIFYNVRSDDVKNLDGEFSLPFKQL‑KQN‑‑‑‑‑‑‑‑EPERVE‑‑‑‑‑‑‑‑‑‑‑‑F‑SRSNSK‑‑Y‑‑‑‑K‑HDTDFVLDIV

>Q9FKM9

GADVRKTFLSHMLKEFKRKGI‑VPFID‑NDID‑RSKSIGPELDEAIRGSKIAIVMLSKNYASSSWCLNELVEITKCR‑KDLNQT‑VMTIFYGVDPTDVKKQTGEFGKVFERTCESK‑‑‑T‑‑‑EEQ‑V‑KTWREVLDGAAT‑IAGE‑‑‑HWHI‑W‑‑D‑NEASMIEKIS

>Q9FHF6

GGDVRVTFRSHFLKEFDRKLI‑TAFRD‑NEIE‑RSHSLWPDLEQAIKDSRIAVVVFSKNYASSSWCLNELLEIVNCN‑D‑‑‑KI‑IIPVFYGVDPSQVRYQIGDFGRIFEKTCKRQ‑‑‑T‑‑‑EEV‑K‑NQWKKALTLVAN‑MLGF‑‑‑DSAK‑W‑‑D‑DEAKMIEEIA

>Q9CAD8

GPDVRKTVLSHLRKQFICNGI‑TMFDD‑QRIE‑RGQTISPELTRGIRESRISIVVLSKNYASSSWCLDELLEILKCK‑EDIGQI‑VMTVFYGVDPSDVRKQTGEFGIRFSETWARK‑‑‑T‑‑‑EEE‑K‑QKWSQALNDVGN‑IAGE‑‑‑HFLN‑W‑‑D‑KESKMVETIA

>O23530

GEDVRDSFLSHLLKELRGKAI‑‑TFID‑DEIE‑RSRSIGPELLSAIKESRIAIVIFSKNYASSTWCLNELVEIHKCY‑TNLNQM‑VIPIFFHVDASEVKKQTGEFGKVFEETCKAK‑‑‑S‑‑‑EDE‑K‑QSWKQALAAVAV‑MAGY‑‑‑DLRK‑W‑‑P‑SEAAMIEELA

>F4KIC7

GEDVRKTFVSHLFCEFDRMGIKA‑FRDDLDLQ‑RGKSISPELIDAIKGSRFAIVVVSRNYAASSWCLDELLKIMECN‑KD‑‑‑T‑IVPIFYEVDPSDVRRQRGSFGEDVESH‑DKE‑‑‑‑‑‑‑‑‑‑‑KVGKWKEALKKLAA‑ISGE‑‑‑DS‑R‑‑‑‑N‑DDSKLIKKIV

>Q9SUK4

GEDVRKNFLSHLLKQLNRRSI‑NTFMD‑HVIE‑RSCIIADALISAIREARISIVIFSKNYAASTWCLNELVEIDNCS‑KYFGQK‑VIPVFYDVDPSHVRKQIGEFGKVFKKTCEDK‑‑‑P‑‑‑ADQ‑K‑QRWVKALTDISN‑IAGE‑‑‑DLRN‑G‑‑P‑NDAHMVEKIA

>Q8H1N6

GADVRKSFLSHIMKEFKSKGI‑DIFID‑KDIK‑RGKSIGPELTEAIRGSRVAIVFLSRKYASSSWCLNELALIMKCR‑KELGLT‑VMTLFYDLDPTDVRKQTGDFGMAFKETCKGK‑‑‑T‑‑‑KDE‑I‑GRWRHALEEVAK‑IAGY‑‑‑HSSI‑W‑‑D‑NEADMIGIVT

>F4I594

GSDVRTSFLSHFRKQFNNNGI‑TMFDD‑QRIL‑RGETISPALTQAIRESRISIVLLSKNYASSGWCLDELLEILKCK‑DDMGQI‑VMTVFYGVDPSDVRKQTGEFGIAFNETCACR‑‑‑T‑‑‑EEE‑R‑QKWSQALNYVGN‑IAGE‑‑‑HLLN‑W‑‑D‑NEAKMIEKIA

>Q9SSN2

GVDTRRNFISFLYKEFVRRKIRT‑FKDDKELE‑NGRRISPELKRAIEESKFAVVVVSVNYAASPWCLDELVKIMDFE‑NK‑GSITVMPIFYGVDPCHLRRQIGDVAEQFKKH‑EAR‑‑‑E‑‑DHE‑‑KVASWRRALTSLAS‑ISGD‑‑‑CS‑SKC‑‑E‑DEAKLVDEIA

>F4JT80

GADTRHDFTSHLVKYLRGKGIDV‑FSDAK‑‑LRGGEYIS‑LLFDRIEQSKMSIVVFSEDYANSWWCLEEVGKIMQR‑RKE‑FNHGVLPIFYKVSKSDVSNQTGSFEAVFQSP‑TKI‑‑D‑‑‑‑‑EQ‑KIEELKVALKTASNIR‑GFVYPE‑‑N‑‑‑‑S‑SEPDFLDEIV

>Q9M285

GADVRRTFLSHIKESFRRKGI‑DTFID‑NNIE‑RSKSIGPELKEAIKGSKIAIVLLSRKYASSSWCLDELAEIMKCR‑EMVGQI‑VMTIFYEVEPTDIKKQTGEFGKAFTKTCRGK‑‑‑T‑‑‑KEH‑I‑ERWRKALEDVAT‑IAGY‑‑‑HSHK‑W‑‑C‑DEAEMIEKIS

>Q9SCZ2

GEELRNSFVSHLRSALVRHGVNI‑FIDTN‑‑EEKGKPLH‑VFFQRIEESRIALAIFSVRYTESKWCLNELVKMKEC‑MDK‑GKLLIIPIFYKVKAYEVRYQKGRFGCVFKNL‑‑RN‑‑V‑‑‑‑‑DVHKKNQWSEALSSVADRI‑GFSFDG‑‑K‑‑‑‑S‑DEHNFINGIV

>Q9FKE2

GDELRKTFISHLHKRLQRDGINA‑FIDSD‑‑EAVGEELK‑NLFKRIENSEIALAVLSSRYTESHWCLQELVKMMEC‑SMKEKKLLVIPIFYKLKIDTVKELDGDFGRNLWDL‑WRKGRD‑‑‑‑‑RDSRIVKWNEALKYFLSRN‑ALVFSE‑‑T‑‑‑‑G‑KEEEFVSTIA

>Q9LFN1

GEDVRRDFLSHIQMEFQRMGI‑TPFVD‑NEIK‑RGESIGPELLRAIRGSKIAIILLSRNYASSKWCLDELVEIMKCR‑EEYGQT‑VMAIFYKVDPSDVKNLTGDFGKVFRKTCAGK‑‑‑P‑‑‑KKD‑I‑GRWRQAWEKVAT‑VAGY‑‑‑HSIN‑W‑‑D‑NEAAMIKKIA

>F4HR53

GADTRDNFGDHLYKAL‑KDKVRV‑FRDNEGME‑RGDEISSSLKAGMEDSAASVIVISRNYSGSRWCLDELAMLCKMK‑SSLDRR‑ILPIFYHVDPSHVRKQSDHI‑KDFEEH‑QVRFS‑‑‑‑‑EE‑‑KVQEWREALTLVGN‑LAGY‑‑‑‑VCDK‑‑‑‑SKDDDMIELVV

>V4LSJ3

GPDVRKGFLSHLHDLFARKEI‑TIFKD‑QEIE‑RGQTIGSELVKAIREARLSIVLLSKNYASSSWCLDELVEILKCR‑EVQGQI‑VMPIFYDVDPSHVRKQRGDFGIAFEKTCEGE‑‑‑T‑‑‑EEV‑K‑QRWVEALTCVAT‑IAGE‑‑‑HSRN‑W‑‑T‑DEAEMVEKLS

>V4MFB1

GEDVREEFLSHIKKEFGRKGI‑TPFND‑NGIK‑RGESIGPELIRAIRESKIAIVLLSKNYASSKWCLDELVEIMKCR‑EELGQT‑VMAVFYKVDPSDVKKLLGDFGKVFRKTCAGK‑‑‑R‑‑‑KED‑I‑GRWRQALEKVAT‑IAGY‑‑‑HSNN‑W‑‑D‑DEAAMIEKIA

>V4L7N8

SDETRWSLVPHLSAAFGRKGISV‑LTK‑‑‑‑‑‑‑‑‑‑‑‑H‑‑‑DEFSMSIASVLIFSKNYASSKESLDDFLKTIQRR‑HDKGHV‑VTTVFCGVSRSNVK‑‑‑ANFAKALFEH‑W‑T‑‑‑‑‑‑‑SY‑‑QASQWCNALEEIAS‑LPGH‑‑‑ED‑N‑‑‑‑F‑SDCEFVEKIA

>V4MHR6

GADVRKKLVSHLNDALNEEGIKT‑FHDDRDLQ‑RGDVIWEALEEAINQSRFAIVVISEGYADSHWCLRELSLMVDLE‑KK‑‑RLQLIPIFYEIDPSNLKSRTG‑FSKAFEKH‑EPR‑‑‑‑‑‑GKE‑‑TVKPWRSALATVGN‑ISGW‑‑‑DS‑K‑‑‑RN‑EDSKLVQNVV

>V4KFI3

GADVRRSFLSHIMKEFRSKGI‑SPFVD‑NEIK‑RGEFIGPELKTAIQGSRIALVLLSKNYASSSWCLDELVEIMKCK‑EDLGQT‑VKPIFYEVDPTDIKKQTRDFGKVFNKTCEGK‑‑‑T‑‑‑NEV‑I‑GKWSQALEIVAT‑IAGY‑‑‑HSNK‑W‑‑R‑DEATMIEDIA

>V4KQN6

RPDIRKTLLSHLREQFNLKGI‑TMFDD‑NNIN‑RGEDLDPSLKEAIRESKI‑‑‑‑‑‑‑‑‑‑‑‑‑‑SLQEICFIKLSK‑NAMKQI‑VMTTFYRVETSYVRKQTGEFRIAFNETCARK‑‑‑T‑‑‑DKE‑K‑QKWSKALTGVSN‑IAGE‑‑‑DFKN‑W‑‑D‑NEAYMIKKIA

>V4KGM7

GEDTRGNITKRLYEALNEKEIRV‑FFDNDGMK‑KGEEVDPSLVAGMEDSAASVIILSPKYADSHWCLDELAMLCDLK‑KSLNRL‑MIPIFYMVDPSNVRKQNAHF‑KDFENH‑AKRFS‑‑‑‑‑EE‑‑KIQRWKRAMTLVGN‑LSGF‑‑‑‑VCKE‑‑KDSADDEMIELVV

>V4KFF3

GPDVRRGFLSHLHNHFASKGI‑TTFKD‑QRIE‑RGHTIGPELVQAIRESRVSIVLLSKNYASSSWCLDELVEILKCK‑EASGQI‑VITIFYDVDPSDVRKQRGDFGSTFMKTCEGK‑‑‑A‑‑‑EGE‑K‑KRWIKALAYVAT‑IAGE‑‑‑HSLN‑W‑‑D‑DEAAMVEKIA

>V4KSP7

GEELRENFVSHLTDAFERHEIKF‑FIDKD‑‑EQRGKDLK‑HLFVRMEESSIALAIFSTRYPESTWCMEELVKMKKL‑EEQ‑GKLQVIPIFYKVEAQDVRGQEGKFGENFWML‑ARAS‑‑‑‑‑‑‑SGEQIKEWKEALEYISKKM‑GLSLGD‑‑K‑‑‑‑S‑SEADFVKKIV

>V4M3H5

GPDVRRGFLSHLHDLFARKEI‑TIFKD‑QEIE‑RGHTIGSELVKAIREARLSIVLLSKNYASSSWCLDELVEILKCR‑EAQGQI‑VMPIFYDVDPSSVRKQSGDFGIAFEKTCEGE‑‑‑T‑‑‑EEV‑K‑QRWVEALTCVAT‑IAGE‑‑‑HSRN‑W‑‑T‑DEAAMVEKLS

>V4M3H1

GPDVRKGFLSHLHDLFARKEI‑TIFKD‑QEIE‑RGQTIGSELVKAIREARLSIVLLSKNYASSSWCLDELVEILKCR‑EVQGQI‑VMPIFYDVDPSHVRKQRGDFGIAFEKTCEGE‑‑‑T‑‑‑EEV‑K‑QRWVEALTCVAT‑IAGE‑‑‑HSRN‑W‑‑T‑DEAEMVEKLS

>V4L8Q0

GPDVRRGFLSHLHDLFARKEI‑TIFKD‑QEIE‑RGHTIGSELVKAIREARLSIVLLSKNYASSSWCLDELVEILKCR‑EAQGQI‑VMPIFYDVDPSSVRKQSGDFGIAFEKTCEGE‑‑‑T‑‑‑EEV‑K‑QRWVEALTCVAT‑IAGE‑‑‑HSRN‑W‑‑T‑DEAAMVEKLS

>V4N330

GAELRKNFVSHLKDALERNGINA‑YIDSN‑‑EHAGEDLD‑ILFRRIEESTVALTILSRRYTESHWCLGELVHIMKC‑VDR‑RTLWVIPIFYKLEPGTVKKLDGEFGVQLWNL‑WRK‑‑‑‑‑‑‑‑RDDRILKWDAALQGVAKKI‑ALESEI‑‑S‑‑‑‑R‑DEVAFLDKII

>V4L7H1

RDELRDNFIRYLVWALIDERINV‑FIDRG‑‑EANKREIR‑NFSTMIEDSDIAVVIFSKRYTESEICLNELQKMHEH‑AEQ‑NRLRVIPVFYDVSTSDVKNLEGEFGTHFKEM‑KEK‑‑‑‑‑‑‑‑DPLKFLNWEGSLSSIAC‑‑‑GLTSEE‑‑H‑‑‑‑G‑TGLGLVREIV

>V4KZZ6

GEELRNSFVSHLADDFKRHGIDF‑FVDNS‑‑ELRGKDLK‑TLFERIEESRIALAIFSTRYAESRWCLDELVKMKKR‑ADK‑KKLHVIPIFYKVRARDVKEQEGEFGEHFRKL‑ARAS‑‑‑‑‑‑‑SGNQIKKWKEALERISEKM‑GLPLKD‑‑N‑‑‑‑S‑TEAGFVKEIV

>V4NKA3

GPDVRKGLLSHLHDHFLRKEI‑KMYKD‑DKME‑RCNTIKHELVKAIRESRVLMVLLSKNYASSIWCLDELVEILKCR‑KDKEQI‑VMPIFYDVDPFHVRTQSGDFGSAFEKTCENQ‑‑‑T‑‑‑EEV‑K‑QRWVEALKCVTT‑IAGE‑‑‑HSCN‑W‑‑P‑DEAAMVQKLC

>V4LDJ8

GVELRYNFVSHLDKSLKRNAINA‑FIDTD‑‑EEMGQNLD‑VLLTRIEGSRIALAIFSPRYTESDWCLKELAKMKER‑MEQ‑RKLVVIPIFYKVEPATVKELKGEFGDKFREL‑ARF‑‑I‑‑‑‑‑DKKTKKKWKEALKSVPLLT‑GFVLND‑‑K‑‑‑‑S‑DEDEIIVKVV

>V4L3N2

GEDTRDNFVGLLYDEL‑KKQIRV‑FRDNEGMD‑RGNEIAPSLVAAMEDSAASVVVLSPRYADSRWCLDELAMLCDLR‑SSLKRP‑ILPIFYKVDPSHVRKQSHHF‑EDFEEH‑AKRFT‑‑‑‑‑KE‑‑E‑‑‑‑‑‑‑‑‑‑‑‑‑‑‑‑‑‑‑‑‑‑‑‑‑‑‑‑‑‑SGEDEMIKLVV

>V4MQM0

GADTRNNFTDHLRQCLRRKSIDA‑FFDEK‑‑LRRGQDIS‑VLFERIEQSKMSIVVFSENYANSSWCLDELWKILQC‑REK‑SGHEVIPIFYKVKKSDVENQKGSFGAPFQSP‑MEI‑‑D‑‑‑‑‑EQEKIGTWRESLRTASNIL‑GFVYHE‑‑D‑‑‑‑I‑SETKFLDDIA

>V4L7J0

GEDVRKNFISFLDPALRRANINV‑FIDEN‑‑EFLGAELA‑NLLTRIEESEIALVIFSENYADSDWCLDELAKMKER‑KDQ‑GRLIVIPIFYNLDPSVVKELRGNFGDKFRDL‑KRRHLH‑‑‑‑‑QLERTQKWEEALVSIPDIK‑GMPRAE‑‑Q‑‑‑‑S‑DDNDFINSMV

>V4MS84

GEDVREKFVSHLYEALNEEGIIT‑FHDDRNLE‑KGDFIWEALEEGINQSRFAIVVISEGYAESQWCLRELSFMVELE‑KK‑‑RLELIPVFYEIDPSILKSRFG‑FKKAFEKH‑EVR‑‑‑‑‑‑DQE‑‑TVSRWRSALATVGN‑ISGW‑‑‑DS‑K‑‑‑KN‑EDSKLVQKIV

>V4L7T2

GADLRHGFVSHLIPALKMNGVGV‑FVDQM‑‑EARGQEIG‑SLFKRIEESEIALVIFSERYTESAWCLDELVKIKET‑KEE‑GKLIAIPIFYKVEPSQVRKLMGQFGDNFWRL‑CRS‑‑S‑‑‑‑‑RAGHIMKWKEALESTASTL‑GFVLSE‑‑G‑‑‑‑S‑SESRIVTDIV

>V4M9Q0

GADTRKNFVSFLYKELEAKGIRT‑FKDDKELV‑RGRLISPELLQAIKESRIAVVVVSANYPGSNWCLEELRAILKLE‑AK‑GLLTVMPIFYEVEPSHVGKQIGEVAKQFKKH‑EKR‑‑‑Q‑‑SRE‑‑KVKSWRAALARLAN‑LSGE‑‑‑CS‑KNC‑‑E‑DDAKLVDDFT

>V4KHQ3

GHDVRRTFLSHFLEGLKSKDI‑KTFKD‑NGIK‑RSESINSELIRAIRESRIAVVILSKNYASSSWCLNELQMIMECR‑VSLGQT‑VMTIFYELEPSDVRKQTGDFGKAFKETCVGK‑‑‑T‑‑‑EKE‑K‑QRWREALTQVAV‑IAGE‑‑‑HSFS‑W‑‑P‑SEVDMISKII

>V4LAG4

GPDVRKTFLSHLRKQFSYNGI‑TMFDD‑NGIE‑RGQIIAPSLTQAIRESRIAVVLLSKNYASSSWCLDELLEILNCK‑EARGQK‑VMTVFYGVDPSEVRKQTGDFGKAFHKTCARK‑‑‑T‑‑‑KEE‑R‑RKWSEALTYVGN‑IAGE‑‑‑HFQN‑W‑‑K‑SEAEMIEKIA

>V4LWX4

GEDVRRSFLSHIQKEFERKGI‑TPFND‑NGIK‑RGKSIGPELIRAIRGSKIAIILISKNYASSSWCLDELVEIMKCE‑EELGQT‑VLAIFYKVDPSDVKKLTGYFGEVFKKTCADK‑‑‑S‑‑‑KED‑I‑RRWRQALEKVAK‑IAGY‑‑‑HSIN‑W‑‑D‑DEANMIETIA

>V4JUG9

GPDVRKTLLSHMREQFKRNGI‑TMFDD‑QKIV‑RSATIAPSLTDGIRESRISIVILSKRYASSSWCLDELVEILRCK‑EVMGQI‑VMTIFYGADPSQVRKQTGEFGMVFDETCSSK‑‑‑T‑‑‑GEE‑R‑QKWKEALKDVGN‑IAGE‑‑‑DFLT‑W‑‑D‑NEANMIEKIA

>V4JRJ4

GLDTRRNFISFLYRELIRRHIRT‑FKDDKELE‑TGRRISSELIRAIEESKFAVVVISANYAASTWCLEELVKIMDFA‑NK‑GSLTVMPIFYGVDPCHVRWQIGEVAEQFKKH‑EAR‑‑‑Q‑‑DHE‑‑KLLSWRQALTNLAS‑ISGL‑‑‑CS‑LKW‑‑E‑DDSKLVDKIT

>V4MBH8

GPDVRKRFLSHLHHYFACKGI‑KMYKDNNNME‑RCHTIKRELRQAIKESRVLMVLLSKNYASSSWCLDELVEILKCR‑KDKEHI‑VMPIFYDVDPSHVRTQSGDFGIAFEKPAKTQ‑‑‑T‑‑‑EEL‑K‑QRWVEALKRVTT‑IAGE‑‑‑HSRN‑W‑‑T‑DEAEMVQKLC

>V4M428

GEDVRRGLLSYLLKEFREKVI‑DVFID‑NDIE‑RSKPIGPELKEAIKGSMIAIVLISRNYASSTWCLDELVEIMKCR‑EEFAQT‑VMVVFHEVEPSDVKKQNGYFGSVFAKTCEGK‑‑‑K‑‑‑PEA‑V‑ERWKQALQEVAK‑IAGY‑‑‑HSLK‑F‑‑D‑SDSDMIETIA

>V4NK95

GADVRKRFLSHLHNYFEIKGI‑TTFKD‑QEIE‑RGNSIGPELVKAIRESRVSIVLFSTNYASSSWCLDELVEILKCK‑EASGLI‑VMTIFYDVDPSSVRKQKGDFGNAFMKTCQGK‑‑‑T‑‑‑EEV‑K‑QRWSKALTDVAN‑IEGE‑‑‑HSLN‑W‑‑P‑NEAEMIQKIA

>V4KV35

GSDVRQTFLSHVLEELRRRGI‑TPFID‑NEIK‑RGESIGPELVRAIRESRVAIVVLSRSYASSSWCLDELVEIIKCR‑EDRQQK‑VTPVFYQVDPSDVRNQTGDFGKAFEETCKKK‑‑‑T‑‑‑EEV‑T‑QAWRQALKEVAN‑IAGY‑‑‑HSSN‑W‑‑S‑NEADLINNIA

>V4LRH4

GADLRIGLIGYLKEALIENNIKY‑YIDSE‑‑EPRGAPIE‑ILFERIRESQIALVFFSIRYAESEWCLDELVEIMKN‑MEK‑GKLTVIPVFFKVEPGDVKGQKKEFGVALYGE‑GRR‑‑‑‑‑‑‑‑KRPRMPQWEDALEIIPTRM‑GLEFWE‑‑Q‑‑‑‑S‑EEVVFRNKLI

>V4KP15

SMKDRYSFVSHLSAAFHRRNIST‑FLESS‑‑‑‑‑‑‑‑‑‑Q‑‑‑SAIEGAKVFVVVFSENYAFSPLSLETLAKFLDLR‑RENGPV‑VIPVFYGVTPSIVEQQTEKFGKAFSEH‑R‑S‑‑‑S‑‑EDD‑‑KVERWRNGLVEAAK‑LQGH‑‑‑DS‑E‑‑‑‑Q‑NDSDLVEEIV

>V4MII2

GEDVRRSFLSHIQKEFERKGI‑TPFND‑NGIK‑RGKSIGPELIRAIRGSKIAIILLSKNYASSRWCLDELVEIMKCR‑EELGQT‑VMAIFYKVDPSDVKKQTGYFGEVFKKTCAGK‑‑‑S‑‑‑KED‑I‑RRWRQALEKVAK‑IAGY‑‑‑HSNR‑W‑‑D‑NEAAMIEEIV

>V4N333

N‑DSVSPFISHLIPAIRRK‑VTS‑SF‑‑‑‑‑‑‑‑‑‑‑‑‑‑‑‑‑‑‑‑‑GFTLLLVIFSRDYAYSVSCLEKLVKALESS‑SDENSYVVVPVFYGVSRLAVKQQLATFSDAFTEH‑R‑R‑‑‑S‑‑‑AD‑‑QVTKWRRALKEAAE‑FLGH‑‑‑EY‑D‑‑‑ES‑EESEFLEKIV

>V4MFV5

GKELRKGFVSFLVPALRKENINV‑FIDEI‑‑EIRSIDLQ‑HIFKRIEESSVAVVIFSELYTESKWCLNELVKINER‑MIE‑GKLKVIPIFFNVTVSDVKIHEGDFGKNFRET‑KRKCQG‑‑‑‑‑DSDIIRNWEEALNSIPQKF‑GLASST‑‑Y‑‑‑‑‑‑‑EYDLVHAIV

>V4LX86

GPDIRKTFLTHLRKQFNSNGI‑TMFDD‑QGIE‑RSKFIASELIRAIIESRISIVILSKNYGSSSWCLNELVEILECK‑N‑‑‑‑I‑VMPIFFQV‑‑‑‑‑‑‑‑‑‑‑‑‑‑VHPISCAGK‑‑‑T‑‑‑KEE‑E‑QRWSQALTDVAN‑IAGL‑‑‑HLLN‑W‑‑E‑NEADMIEKIA

>V4LR44

GPDVRKSFLSHLRKQFNYNGI‑TMFDD‑QGIE‑RSQIIAPALTQAIRESRISIVLLSKNYASSGWCLDELLEILNCK‑EDLGQI‑VMTVFYGVNPSDVRKQSGDFGFAFNETCSRK‑‑‑T‑‑‑EEE‑S‑RNWSKALTYVGN‑IAGE‑‑‑HSQN‑W‑‑D‑NEAEMIEKIA

>V4LS85

WEDTRYSFISHLAAAFHRKGISS‑FV‑DP‑‑‑‑‑‑NF‑AR‑‑‑VAMEKSRACVVVFSGKYSSSKPCLEELVNVSERR‑RNNGLA‑VVPVFFPVTKLFVKKQIWNLG‑‑‑‑‑‑‑‑‑‑‑‑‑‑‑‑‑‑H‑‑VRSEWQSALLETAE‑LPGH‑‑‑QL‑D‑‑‑‑K‑SDSEFVQEIV

>V4LBN3

GEDVRRDFLSHIQMEFQRNGI‑TPFID‑NEIK‑RGESIGPELIRAIRGSKIAIILLSRNYASSKWCLDELVEIMKCR‑EELGQT‑VMAIFYKVDPSDVKKLTGDFGRVFRKTCSGK‑‑‑K‑‑‑KED‑T‑ERWRQALAKVAT‑IAGY‑‑‑HSNN‑W‑‑D‑NEVAMIKKIA

>V4KNX1

TTELPDDFMRHLVWGLSELGINI‑FIDRD‑‑EWWGRDLG‑HVFTCIEESTIALAIFSSCYPETEWCLDELVKMKER‑VNE‑NRLFVIPVFYNVSKNDVRNLEGEFGDRFMEM‑RQKYKY‑‑‑‑‑DPFRAQRWETSVTSISR‑‑‑SLTWEA‑‑H‑‑‑‑S‑SNIPLASNIV

>V4ME37

GPDVRVTFLSHLQMQFERNGI‑TTFND‑EGIE‑RSQLIGSELTQAIRESRISIVVLSENYASSNWCLKELVEILKFQ‑ESAGQI‑VMTVFYCVDPCDVKKQMGELGKAFKKTCQGK‑‑‑T‑‑‑ETE‑M‑ESWIQALTHVAN‑IAGE‑‑‑HSLKKF‑‑N‑KSANMIEKIA

>V4KBN9

GEDVRKSFLGHLRKQFNYNGI‑TMFDD‑KGIE‑RSDTISPSLIQAIRQSRISIVILSKNYASSSWCLNELVEILECK‑KAMGLI‑VMTIFYGVDPSHVRKQTGHFGSAFNETCLRK‑‑‑T‑‑‑DEE‑R‑RKWSRALTDVSN‑ILGE‑‑‑DFLN‑W‑‑D‑SEANMIEKIA

>V4KP22

GDELRKSFISHLYSRLRSEGINA‑FIDTD‑‑EGAGQELE‑NLFKRIEESKIALAVLSSRYTESHWCLQELVKIKEC‑SMKENNLFVIPIFYKLETSTVRELTGKFGLNLWDL‑WRVDRN‑‑‑‑‑RDNRIVKWNEALENVLGKK‑ALILTE‑‑T‑‑‑‑G‑KEDDFLSTIV

>V4KUY9

GVELRYNFVSHLDKSLKRNAINA‑FIDTD‑‑EEMGQNLD‑VLLTRIEGSRIALAIFSPRYTESDWCLKELAKMKER‑MEQ‑RKLVVIPIFYKVEPATVKELKGEFGDKFREL‑ARF‑‑I‑‑‑‑‑DKKTKKKWKEALKSVPLLT‑GFVLND‑‑K‑‑‑‑S‑DEDEIIVKVV

>V4LPJ0

GEDVRKTFVSHLFCELDRMGINA‑FRDDLDLE‑KGKSVSPELVDAIRGSRFAIVVVSRNYAASSWCLDELLKIMEC‑‑KDTDQT‑VVPIFYEVDPSVVRRQTESFGKDVESH‑DKE‑‑‑‑‑‑‑‑‑‑‑KVRKWKEALTKLAA‑ISGE‑‑‑DS‑R‑‑‑‑NRDESKLIKKIV

>V4LB82

GKDLRLGFVSHLVRAFKRNKINV‑FMDEF‑‑EDRGKPLD‑SLLKRIEGSRIALAIFSESYTESNWCLKEVEKMNDC‑MEQ‑GNLVVIPIFYKVEPSTVRYLKGDFGDKLWIL‑VKG‑‑‑‑‑‑‑‑DE‑KKKKWEEVLKSIPNLF‑GITVDE‑‑K‑‑‑‑S‑DEGQAVNEIV

>V4MNA0

GDQLRYNFVSHLIDAFERHGISI‑FVDKY‑‑EMRGKDLK‑NLFVRLKESRIALAIFSTRYAESSWCMDELVKMKKF‑ADK‑EKLQVIPIFYKVRARDVRRQTGEFGDNFWKL‑ARAS‑‑‑‑‑‑‑SGDEIKNWKE‑‑‑‑‑‑DKM‑GLSLKD‑‑M‑‑‑‑S‑SEADFVKEIA

>V4KPU4

GEDVRKNFLSHLLKEFENKGI‑VTFRD‑DQIE‑RSHSIGPELVEAIRESKISLVLFSENYASSSWCLDELVEILKCK‑EEQRLK‑VMPIFYKVDPSDVRKQTGKFGMCFWETCYGK‑‑‑T‑‑‑EEK‑Q‑RSWRQALTDAAS‑IVGD‑‑‑HSQD‑W‑‑D‑NEANMITKIA

>V4MFZ2

SHDVDSHFMKTILKELREREVTP‑LTN‑‑‑IR‑R‑EL‑DT‑‑‑ELLDRSRVGILVLSNNYACSSESLDHLVAIMEHW‑KAN‑‑‑‑‑‑PVYFRVTLSNI‑ELEDPFEAVQLQC‑L‑N‑‑‑P‑‑‑AD‑‑RVQNWKEAMAEISS‑LGGN‑‑‑AV‑QNFSHI‑TQVMLAEEVV

>V4LWM1

GSDVRRGFLSHLHNDFALMGI‑KTFND‑QKME‑RGHSL‑‑SLDLAIRESRVLMVLLSKNYASSLWCLNELVEILECR‑KNMVQC‑VLPVFYHVDPSDVRSQSGDFGNGFKKSCQGK‑‑‑N‑‑‑EEE‑K‑QIWSKALTKVAN‑IAGL‑‑‑HCVN‑‑‑‑Y‑DDSEMIKKIA

>V4M356

RPDVRKTLLSHMREQFKRTGI‑TMFDD‑QEIE‑RSAIIAPSLIEAIRESRISIVILSKKYASSSWCLDELVEILECK‑KA‑GQT‑VMTIFYGVHPSDIRKQTGEFGNTFNETCAHK‑‑‑T‑‑‑DQE‑R‑QKWSKALNDVGN‑IAGE‑‑‑DFLK‑W‑‑D‑NEAIMIKKIA

>V4LVY3

GPDVRKTLLSHMREQFKRTGI‑TLFDD‑QEIE‑RSAIIAPSLIEAIRESRISIVILSKKYASSSWCLDELVEILECK‑KA‑GQI‑VMTIFYGVDPSDIRKQAGEFGIAFNETYAHK‑‑‑T‑‑‑DKK‑R‑QKWSKALNDVGN‑IAGE‑‑‑DFLK‑W‑‑D‑NEAIMIKKIA

>V4L7L4

GKEVRGNFASHLKNALIREGINV‑FTDNN‑‑ERMGKALD‑IFFTRIEESKIAIAIISSLYTESKWCLNELVKIHEC‑VKK‑ETLEVFPVFYKVNVDTVKRRKEKFGENFDRL‑VKK‑‑E‑‑‑‑‑HTER‑KKWSRALRFVAGIK‑GEVVDE‑‑K‑‑‑‑S‑DEVQVINKIT

>V4KGQ2

GPDVRKTLLSHLREQFNLKGI‑TMFND‑NKIK‑RGEELDPSLKEAIRESKIWIVILSKKYASSSWCLDELVEILERK‑NAMKQI‑VMTVFYGVEPSDVRKHTGEFGIAFNETCARK‑‑‑T‑‑‑DEE‑R‑QKWSKALTDVSN‑IAGE‑‑‑DFKN‑W‑‑D‑NEANMIKKIA

>V4NGQ8

GKELRNNFVSHLRNALQRHGVNI‑FIDTN‑‑EQKGKPLN‑VLFQRIEESRIALAIFSVKYTESKWCLNELVKMKEC‑MDK‑GKLLIIPIFYKVKAYHVRFQRGRFGYVFKNL‑‑RN‑‑V‑‑‑‑‑DDDKKNQWSEALSSVADRI‑GFCFEG‑‑M‑‑‑‑S‑DENDFINCIV

>V4L370

GEDVRVTFLSHFLKELDRKLI‑IPFKD‑NEME‑RSRSLDPELKQAIKDSRIAVVVISKNYASSSWCLNELLEIVNCK‑EEYGQM‑VIPVFYALDPSHVRKQTGDFGKIFEETCKNS‑‑‑T‑‑‑KEV‑T‑NRWRSALTDVAN‑ILGY‑‑‑HSVS‑W‑‑G‑NEAKMIEEIA

>V4NC85

‑‑‑VRLTMMIHVVRKLKSKGI‑TPFID‑NEIK‑RGQSIGPELILAIRESRVAIVLLSSNYASSSWCLDELVEIIKCK‑EENQQT‑VMTIFYDVDPSDVRKQTGDVGKAFEKTCVGK‑‑‑T‑‑‑KEV‑K‑QRWSQALKDVAS‑IA‑V‑‑‑ESSF‑F‑‑D‑NEADLINKIA

>V4M5V3

SKDTRDNFVSHLCGCLRRKRIKT‑YLD‑‑ELE‑R‑EE‑‑‑SLK‑AIQ‑SRVSVIVFSENFGDSKGLV‑‑‑‑‑‑‑‑‑‑‑‑‑‑‑RF‑VIPVLYHVDPLDIENQTGSFGDAFAKR‑QDK‑‑‑A‑‑‑‑E‑‑QLQEWRDCFTE‑AN‑LPGW‑‑‑‑STN‑Y‑RD‑‑DEMLVNEIA

>V4LJ22

GEDIRKTFVSHFLTELDRKFI‑SAFKD‑NKIK‑KGQTLDPVLKEAIKDSRIAIVIFTENYASSAWCLNEMLKIVKCK‑KKLGQL‑VIPVFYRLDPSHVRKQSGDFGKIFEETCRDK‑‑‑T‑‑‑EDE‑I‑KLWRTALTDVAN‑QVGF‑‑‑DSRN‑W‑‑E‑NDAKLVEDMV

>V4NK81

GPDVRRRFLSHLHKHFESKGI‑MAFKD‑QGIE‑RGHTIGPELVQAIRESRVSIVVLSKNYASSSWCLDELVEILKCR‑EDQGQA‑VMTVFYDVDPSHVRKQSDDFGIAFKKTCQGK‑‑‑T‑‑‑EEE‑K‑QRWSRALTDVAN‑IAGE‑‑‑HCLT‑W‑‑D‑DEAEMIQKIA

>V4LS88

RPDVRKTLLSHMREQFKRTGI‑TMFDD‑QEIE‑RSAIIAPSLIEAIRESRISIVILSKKYASSSWCLDELVEILECK‑KA‑GQT‑VMTIFYG‑‑‑‑‑‑‑‑‑TGEFGNTFNETCAHK‑‑‑T‑‑‑DQE‑R‑QKWSKALNDVGN‑IAGE‑‑‑DFLK‑W‑‑D‑NEAIMIKKIA

>V4L4M8

GEDVRRTFLSHLVGALDCRLVRTVFKD‑SQIE‑RGHSISPALVQAIRESKISIVVLSKNYASSSWCLNELLEILKCR‑EELDQI‑VMTIFYDLDPSHVRNQTGDFGKAFEITCEDK‑‑‑T‑‑‑EDE‑A‑KQWRLALTQVAN‑IHGH‑‑‑‑‑‑‑‑L‑‑L‑VNVHMIEDFV

>V4KZY8

GEELRSSFLSHLIDAFERHGIDF‑FVDEY‑‑ELRGKELK‑NLFVRIQQSRIALAIFSTRYAESSWCMDELVNMKKL‑ADK‑EKLQVIPIFYKVEAQDVREQTGVFGEHFWTL‑ARAS‑‑‑‑‑‑‑SGDQIKKWKEALECISDKM‑GLPLKD‑‑N‑‑‑‑S‑SEADFVKEIV

>V4MEX6

GEDLRLTFVPHLKHHLKESNVNV‑FTDA‑‑‑DAAGEPLK‑NLFNHIRNSRIVIVIFSISYLESKWCLDELVEIRRC‑LKS‑KLDFVIPIFFKVRASHVKEQSGDFGSKFLAL‑QKKHPR‑‑‑‑‑L‑‑RIMRWKRALRFVAKQI‑GLAAKE‑‑S‑‑‑‑S‑IELDFIKNIV

>V4LBM0

GEDVRVTFLSHFLKELDRKLI‑ISFKD‑NEIE‑RSQSLDPELKQAIRTSRIAVVVFSEKYPSSSWCLDELLEIVRCK‑KESGQL‑VIPVFYGLDPSHVRKQTGKFGEAFTKTCQTK‑‑‑T‑‑‑EDE‑T‑KLWRESLIEVAN‑VLGY‑‑‑HSQN‑W‑‑H‑NEAKMIEAIA

>V4LSJ8

GPDVRRGFLSHLHDLFARKEI‑TIFKD‑QEIE‑RGHTIGSELVKAIREARLSIVLLSKNYASSSWCLDELVEILKCR‑EAQGQI‑VMPIFYDVDPSSVRKQSGDFGIAFEKTCEGE‑‑‑T‑‑‑EEV‑K‑QRWVEALTCVAT‑IAGE‑‑‑HSRN‑W‑‑T‑DEAAMVEKLS

>M4D289

GADVRTNFLSHVLKELRSKGI‑DSFID‑NDIE‑RSKLIGPELVEAIRGSRIAIVLLSRNYASSTWCLNELVEIIKCR‑EEFGQT‑VVPLFYELDPTDVKKQTGDFGKVLGKTCRGK‑‑‑E‑‑‑KED‑I‑QRWKRALTEVAQ‑IAGF‑‑‑HSAN‑G‑‑E‑NEAELIEYIA

>M4EG65

GPDVRKTLLSHMREQFNVNGI‑TMYND‑QKMV‑RSEEIAPSLTNGIRESRIAIVILSKKYASSSWCLDELVEILECK‑KTMGQI‑VMTIFYGVEPSDVRKQTGEFGIAFEDTCEHK‑‑‑T‑‑‑KEA‑K‑QKWIKALTDVSN‑IAGE‑‑‑DFLR‑W‑‑A‑NEADMIKQIA

>M4DA90

NQVSEIYLISYIFNELCARGFAP‑LRD‑‑‑MK‑S‑TT‑EN‑‑‑QKLLHSRVGIIIFSMNFAHSRECLDGFVAVMDHL‑KANELV‑LIPVFFKVSVSDVRGQSGSFGKAFTRL‑G‑D‑‑‑S‑‑‑AS‑‑QVLKWRAAMIKLAS‑IIGY‑‑‑AY‑K‑‑‑‑‑‑DEVILAKNIV

>M4F6T0

GDELRYNFVSHLTSALLRDGVNI‑FIDTN‑‑EEKGKSLN‑VLFERIEESRIALALFSVRYTESKWCLNELLKMKEC‑MDK‑GQLLIIPIFYKVQAYEVRFQRGRFGYLFNKL‑‑RH‑‑V‑‑‑‑‑DVDKKKQWSEALNSVADRI‑GFCFDG‑‑K‑‑‑‑S‑DENKFIHSIV

>M4EG76

GPDVRKTFLSHLRKQFNNNGI‑SMFDD‑QGIE‑RGQTIAPELIRAIRESRISIVVLSKNYASSSWCLDELVEIFKCK‑EDKNQI‑VMTVFYGVDPSDVRKQTGDFGKAFKKTCAR‑‑‑‑‑‑‑‑‑‑‑‑‑‑‑‑‑‑‑‑‑‑‑‑‑‑‑IRGE‑‑‑A‑‑‑‑‑‑‑D‑KESEMIEKIA

>M4EFM4

GEDVRKHFISFLDPALRRANINV‑FIDEN‑‑ELLGADLA‑NLFTRIEESEIALVIFSKNYADSDWCLDELAKMKER‑KDQ‑GRLRVIPIFYNLSPSVVKELRQDFGDKFRDM‑QRRHKH‑‑‑‑‑QPERTKKWEEALVSVPDIK‑GMPLSE‑‑Q‑‑‑‑S‑DDNEFINSLV

>M4DZK9

GEELRENFVSHLYKALRQSGINA‑FIDSD‑‑MVLGDKLI‑TLFKTIKESKIALAILSSKYTASQWCLEELVKIMEC‑STNEKNLVVIPIFYKVSTSIVDKLEGEFGVNLLNV‑WRRGGA‑‑‑‑‑RNSRIVKWNAALQDMLSRA‑ALIYDG‑‑S‑‑‑‑M‑EENAFVARIV

>M4ECP6

GQELRYRFVSHLVAAFERDEINF‑FIDKN‑‑ELRGTDLK‑NIFVRIQESRIALVIFSNRYAESSWCMNELAKIKEL‑ADK‑EKLHVVPIFYKLKVGDVRGQTGKFGTKFWNL‑ARVS‑‑‑‑‑‑‑TGDQIKTWKEALECISDKM‑GLAFGD‑‑D‑‑‑‑A‑QSAGLLSAVA

>M4CPF6

GEDVRRDFFSHIQREFERKGI‑TPFID‑NEIK‑RGESIGPELIRAIRGSKIAIILLSRNYASSKWCLDELVEIMKCR‑EEFGQT‑VMAIFHKVDPSDVKKLTGDFGKFFKKTCAGK‑‑‑A‑‑‑KDC‑I‑ERWRQALAKVAT‑IAGY‑‑‑HSSN‑W‑‑D‑NEADMIKKIA

>M4EG77

GPDVRKTFLSHLRKEFNNNGI‑SMFDD‑QGIE‑RGQTIAPELRRAIRESRISIVVLSEYYASSSWCLDELVEILKCK‑KDKNQI‑VMTIFYGVDPSDVRKQTGDFGKVFKKTCARK‑‑‑T‑‑‑EEE‑R‑RKWSQALNRAGK‑IAGE‑‑‑HFLN‑W‑‑H‑NESEMIEKIA

>M4DWG7

GEDVRIGFLSHIQKEFKRKGI‑TPFID‑NEIR‑RGESIGPELIRAIRGSKIAIILLSRNYASSKWCLDELVEIMKCK‑EELGQT‑VIPVFYKVDPSDVKKLRGYFGKVFEKTCEGK‑‑‑S‑‑‑KED‑T‑EKWRHALEKVAT‑IAGY‑‑‑DSRT‑W‑‑D‑NEAAMIEEIA

>M4DHP9

GPDTRRNFISFLYKELVQRNIRT‑FKDDKELE‑SGQRISPELDRAIEESKFAVVVVSANYAASTWCLEELVKIMDVE‑NK‑GSLTVIPVFHGVDPCHVRRQIGQVAVQLEKH‑EMR‑‑‑E‑‑DRE‑‑KVLSWRQALTNLAS‑ISGV‑‑‑CT‑LKW‑‑E‑DDSMMVDEIA

>D1GEG9

GKDVRQTFLSHLIVALDRKLVCTVFKD‑SQIE‑RGHSISPALVQAIRDSRVSIVVLSKNYASSSWCLDELLEILKCR‑EELGQI‑VMTIFYDLDPSDVRYQIGEFGKAFEKTCEKK‑‑‑T‑‑‑ADV‑T‑KQWGLALTEVAN‑IHGH‑‑‑HSRK‑W‑‑D‑SEAHMVDDFV

>M4CIG3

GADVRKKILSHVLKEFKRRGI‑DTFID‑NNIE‑RSKSIGPKLIEAIRGSRVAIVLLSKNYASSTWCLNELVEITKCR‑REFGQT‑VMPVFYEVDPSDVKKQSGEFGKVFQDICNGK‑‑‑K‑‑‑EED‑T‑RTWREALVEVAT‑IAGE‑‑‑HSSN‑W‑‑C‑SEAEMIEKIA

>M4DZ07

GPDVRKTLLSHVRKQLSCNGI‑SMFDD‑QWIE‑RSQTIAPALTQAIRESRISIVVLSKKYASSSWCLDELVEILKCK‑EKMGQI‑VMTVFYGVDPSHVRNQTRDFGIAFDETCQGK‑‑‑T‑‑‑EEK‑M‑RIWRQALTNVGN‑IAGE‑‑‑HFLN‑W‑‑D‑NESMMIEKIA

>M4FGI2

GPDVRRGCLSYLLKEFKEKAI‑DVFID‑NDIE‑RSKLIGPELTEAIRGSLIAIVLISRNYASSTWCLNELVEIMRCW‑DEDKQT‑VEVIFYEVDPSDVKKQKGDFGAVFDKTCAEK‑‑‑S‑‑‑TEE‑V‑ERWRKALHIVAQ‑LAGY‑‑‑HTSN‑F‑‑D‑DDAVMTAKVV

>M4E9N7

‑‑DFSAIYAISLQ‑‑‑‑‑‑‑‑‑‑‑‑KD‑HEIE‑RGHTIGPELVQAIRESRVSVILLSKNYASSSWCLDELVEILNCK‑KASGQI‑VMTIFYQVDPSDVRKQTGDFGIAFKKTCEKK‑‑‑T‑‑‑EED‑K‑KRWMEALAYVAN‑IAGE‑‑‑HSLN‑W‑‑T‑DEAAMVEKFA

>M4EFU7

GPDVRKTFLSHLRKQFACNGI‑SMFND‑QAIE‑RSHTIAPALTQAIRESRISIVVLTKNYASSSWCLDELLEILKCK‑EEMGQI‑VMTIFYGVDPSHVRKQTGDFGKVLKKTCSGK‑‑‑T‑‑‑EEE‑K‑QRWSQALTDVGN‑IAGE‑‑‑HFLN‑W‑‑D‑KESEMIEKIA

>M4E4E3

GQDVRRSFLSHFLEGLKTNGV‑NTFVD‑DGIM‑MSGSINSELVRAIRESRIAVVILSKNYASSSWCLHELQLIMDCR‑ASLGQT‑VMTIFYDVEPSDVRKQTGDFGKAFEETCNGS‑‑‑T‑‑‑EEE‑K‑KTWRQALTQVAL‑IAGE‑‑‑HVTS‑W‑‑A‑SEAQMISKIV

>M4CQG3

GEDVRKGFLSHVVKEFKSKGI‑EAFID‑NEME‑RGKSVGPTLEKAIRQSRVAIVLLSRNYASSSWCLDELVEIMKCR‑EEDKQR‑VITVFYEVDPSDVRKQIGDFGKAFDDTCVGR‑‑‑T‑‑‑EEV‑T‑HVWRQALKEVAD‑IAGY‑‑‑ASSN‑C‑‑G‑SEADLINELA

>M4CAD3

GEDVRKGFLSHVLKEFKSKGI‑NVFID‑NEIK‑RGESVGPELVKAIRHSRVGVVLLSRNYASSSWCLDELVEIMKCR‑EEVGQT‑VMTIFYNVDPSEVRKQTGDFGKAFDETCVGR‑‑‑T‑‑‑EEV‑K‑RAWRQALNDVAS‑IAGY‑‑‑DASN‑C‑‑D‑NEADLINKVA

>M4EFN2

GDELRKNFISHLVEALQRSEINF‑FTDKQ‑‑EKKGEDLS‑NLFNRIEEAKIALAVFSKRYTESRWCLDELVKIKER‑ADL‑GKLKVVPIFYNVTTDNVKYLTEEFGSNLGRH‑QSPH‑‑‑‑‑‑‑EQNKIGKWKEALACISCKL‑GFPFID‑‑N‑‑G‑S‑SESEFIDSIV

>M4F5Q7

GADVRKSFLSHLVKEFGSKGI‑NLFID‑NEIT‑RGEFIGPELKKTIQGSRIAIVLLSKRYASSSWCLDELVEIMKCK‑EELGQT‑VVPVFYEVDPSDVKKQAGEFGKVFKKTCKGK‑‑‑T‑‑‑NEV‑I‑RKWSKALAKVAT‑LAGY‑‑‑HSKN‑W‑‑D‑NEAKMIEDVA

>M4DMS2

GADVRKAFLSHVLKEFRRKGI‑DPFID‑NDME‑RSKSIGSELIEAIRGSRIALVLLSKNYASSTWCLNELVEIIKCR‑EELDHT‑VMVLFYEVDPADVKTQTGDFGKVFRKTCKGK‑‑‑T‑‑‑KEE‑I‑GRWKHALAEVAK‑ITGY‑‑‑HSRN‑W‑‑D‑KEADMIEKIA

>D1GEF8

GEDVRRTFLSHLLKEFRRKGI‑RTFID‑NDIK‑RSQLIGPELVQAIRESRFAVVVLSKRYASSRWCLNELVEIKE‑‑‑‑‑SSKN‑VMPVFYEVNPSDVRNLSGEFGTAFEEACQGK‑‑‑P‑‑‑‑ED‑V‑QRWRQALVYVAN‑IAGE‑‑‑SSQN‑W‑‑D‑NEADMIEKIA

>D1GED5

GEDVRKDFLSHIQMEFQRKGI‑TPFID‑NEIK‑RRDDIGPELIRAIRGSKIAIILLSRNYASSKWCLDELVEIMKCR‑EELGQT‑VMAIFYRVDPSDVKKLAGDFGRVFKKTCAGR‑‑‑T‑‑‑KEN‑I‑ERWRQALAKVAT‑IAGY‑‑‑HSSN‑W‑‑D‑NEAAMIKKIA

>M4EI84

GPDIRRSFLSHLHKHFESKGI‑TMFKD‑HEIE‑RGHTIGPELVQAIRESRVLMVVLSKKYASSSWCLDELVEILKRK‑EDQGKI‑VMTIFYKVDPSCVRKQNGDFGSIFEKTCEGK‑‑‑T‑‑‑KEL‑K‑LRWTKALTDVAN‑IEGD‑‑‑YSLN‑W‑‑H‑DEAEMIGKIA

>M4E4G8

GVDVRKGFLSHVLKELKSKGI‑LPFID‑NEIK‑RGESVGPVLVGAIRQSRVAVVLLSRNYAYSSWCLDELVEIMKCR‑KEDQQK‑VMTIFYEVDPSHVRKQNGDFGKAFDETCVGK‑‑‑T‑‑‑EEV‑K‑QAWKQALKEVAG‑IAGY‑‑‑DFSN‑C‑‑D‑NEADLINKVA

>M4D251

CANTQYSFASHLSMGFHRKGIYA‑SA‑‑‑‑‑‑‑‑‑SE‑TL‑‑‑DVMEGASASVVVFSKNYLSSPSCLDKLVRVLQCR‑RKSGQL‑VVPVFYDVSPSNVEVQEQESV‑‑‑‑‑‑‑‑‑‑‑‑‑‑‑‑‑‑D‑‑RI‑‑‑‑SALQELRE‑FTGY‑‑‑QF‑E‑‑‑‑G‑SECELVEEIV

>M4FCQ3

GADVRKSFLSHFLKELGSKGI‑NLFTE‑KEIP‑RGEFIGPELKKAIQGSRIAIVLLSKRYASSSWCLDELVEIMKCK‑EELGQT‑VMPIFYEVDPSDVKKQAGEFWKVFKETCKGK‑‑‑T‑‑‑NEV‑I‑GKWSKALAKVAT‑LAGY‑‑‑HSNN‑W‑‑D‑NDVKMIEDVA

>M4DZU2

GLDVRKTFLSHFLKELDLRLI‑TAFKD‑SKIE‑RSQAIEPELLQAIRSSRIAVVMFSKNYASSKWCLDELLEIVKCK‑QELEQI‑VIPVFYGLDPSDIRKQLGEFGEAFDKTCKNR‑‑‑T‑‑‑ESK‑I‑QLWRQALTDVAN‑LEGH‑‑‑HSRN‑W‑‑D‑NEAKMIEAIV

>D1GEE4

GEDVRKNFLSHFLKELDRKLI‑KAFKD‑NEIE‑RSHSIAPALVTAIRTSRIAVVVFSPKYASSSWCLDELVEIVRCM‑EELGQL‑VLPIFYGLDPSHVRKQTGKFGEGFAKTCKMK‑‑‑T‑‑‑KAV‑K‑IRWQQALTVVAN‑LLGY‑‑‑HSQN‑F‑‑N‑NEAKMIEVIV

>M4D878

GEDVRKSFLSHLLKELHRKSI‑NTFID‑HGIE‑RSRPIGPELLSAIRESRISIVVFSKNYASSSWCLNELVEIYKSF‑KELNQM‑VIPVFYGLDPSHVRKQTGEFGEAFMVSCQGK‑‑‑T‑‑‑DDE‑K‑QWWIQALAEVAN‑MAGE‑‑‑DSRN‑W‑‑S‑DESNMIERIA

>M4EFN3

HHDAEVLFIDEIIIELQKRAITS‑LRD‑‑‑LT‑D‑TT‑RM‑‑‑PGLHTYGVFLLFISKNYT‑SGESLDKLVTLTEYQ‑KANGLL‑LIPIFYKVTPSEF‑‑PKFFTEERLLQL‑D‑D‑‑‑S‑‑‑IR‑‑RVQKWKEAMNELAL‑SDDC‑‑‑KW‑F‑‑‑‑‑‑NDSILPEEIV

>M4D290

GADVRKTFLSHVLKEFRSKGI‑DLFID‑KDIE‑RSKSIGPALIEAIRGSRIAIVLLSENYASSTWCLNELVEIIKCR‑QEFGQT‑VMPIFYQVNPTDIKKQKGYFGKVFRKTCKGK‑‑‑R‑‑‑KEE‑I‑QRWKHALTQVAQ‑IEGL‑‑‑MPLS‑R‑‑E‑TEAEMIDDIA

>M4CV15

GADTRRKFVSFLYNDLEAKEIRT‑FKDDKELE‑SGRPIPPELIQAIKGSKIAVVVVSATYPASYWCLEELVKILKYE‑RK‑GLLKVLPVFYEVDPSHLRWQIGEVAKQFKKH‑EKR‑‑‑Q‑‑SKE‑‑RVKSWRDALAYLAN‑LSGE‑‑‑CS‑KKW‑‑D‑DDSKLVDGIT

>M4DZK5

SCDGRDTFVSHLSAALKRVNITV‑MEDVPYL‑‑‑‑‑K‑TR‑‑‑LGIERSKICVVVLSEDFASSKHSLTTLAEIIEWR‑SKTGAT‑VVPVFYGVDRSLVEQQIGKYGEAFSKH‑E‑A‑‑‑S‑‑‑KD‑‑RVTEWRNALTEAAS‑IEGL‑‑‑HS‑A‑‑‑‑E‑SDLKLMEDIV

>M4CPK7

GEDVRRKFFSHIQMELERKGI‑TPFID‑NEIR‑RGESIGPELIRAIRGSKIAIILISRNYASSKWCLDELVEIMKCR‑EELGQT‑VMPVFYEVDPSNVKKLTGDFGKVFRKTCAGK‑‑‑T‑‑‑KEC‑I‑KRWRQAFAKVAT‑IAGY‑‑‑HSSN‑WGRD‑NEADMIKKIT

>M4DZU0

GEDVRKSFLSHFLKELDRKLI‑SAFKD‑KKIE‑TSESLDPVLKQAIKKSRIAIVIFSQNYVSSSWCLNELLEIVKCQ‑QELSQI‑VIPVFYDVDPCHVRHQTKEFGEVFKKTCLRR‑‑‑T‑‑‑DDE‑I‑KLWKKALTDVAN‑LVGY‑‑‑HSQN‑W‑‑E‑NEATMIEVIA

>M4ETB5

GPDVRKTFLSHVRKQFNNSGI‑MMFDD‑QGIE‑RSQTLAPSLTQAIINSRISIVVLSKNYASSSWCLDELVKILECK‑RVNGQT‑VMTIFYGVDPSDVRKQAGNFGRAFNDTCVGK‑‑‑T‑‑‑DEE‑R‑QRWTQALTDVSN‑ILGE‑‑‑HFLN‑W‑‑D‑NESNLIEKVT

>D1GEI4

NQDSERYFISYISKELCLRGFTP‑LID‑‑‑LK‑S‑TA‑GV‑‑‑EMLHRSRVGIIIFSNNYASSRQCLDKFVAILDYS‑KANNFV‑LLPVFFKVKVSDIRGQSGSFRRAFSRL‑E‑H‑‑‑S‑‑‑SS‑‑QV‑‑‑‑‑‑‑‑‑LTA‑INKY‑‑‑QY‑K‑‑‑‑‑‑EDVILAKSIV

>M4E4C8

GEDVRKTFVSHLFCELDRMGINA‑FRDDLDLE‑RGKHISSELVDTIRGSRFAVVVVSRNYASSSWCLDELLEIMER‑‑KNTDQT‑IIPVFYEVDPSDVRRQTGSFGEGVESH‑DKK‑‑‑‑‑‑‑‑‑‑‑KVMKWREALTQLAA‑ISGE‑‑‑DS‑R‑‑‑‑NRDESKLIKKIV

>M4EG59

GPDVRKTFLSHLRKQFTFNGI‑TMFDD‑QGIE‑RGQVIAPAITQAIRQSRISIIVLSKNYASSSWCLDELLEILKCK‑EDMGQI‑VMTVFYGVDPSHVRKQTGDFGKAFKETCARK‑‑‑T‑‑‑KEK‑E‑ERWSQALEYVGN‑IEGE‑‑‑HFLN‑W‑‑V‑NEADMIEKIA

>M4DVZ9

GEDVRNSFLSHLME‑LERNLI‑TTFID‑HGID‑RSRPIGSELLLAIKESRIAIVIFSKNYASSTWCLNELVEIHKCF‑KDLNQM‑VIPIFYHVDPSDVRKQTGEFGDRFKEICMDK‑‑‑T‑‑‑EDE‑I‑ERLVRALTDVAN‑LAGQ‑‑‑DSKN‑W‑‑E‑GEAKMIEHIA

>M4DM13

GEDVRKTFLSHFMKELNRKLI‑TAFKD‑NEIE‑RSRSLDPELRQAIKDSRIAVVIFSTNYASSSWCLNELLEIVRCK‑EECAQM‑VIPVFYGLDPSHVRKQTGDFGKIFDKTCQNK‑‑‑T‑‑‑EDE‑I‑ILWREALTDVAN‑ILGY‑‑‑HSVT‑W‑‑D‑NEARMIDEIA

>M4EW13

GEDVRKDFLSHIQKGFERKGI‑RQFND‑NEME‑RGESISFQLVRAIRGSKIAVVLFSKNYASSKWCLDELVEIMKCR‑REFGQI‑VIAVFYKVDPSDIRKQTGDFGKVFRKTCAGK‑‑‑T‑‑‑NEE‑I‑RRWRVALAEVAA‑IAGY‑‑‑HSSN‑W‑‑D‑NEADMVENIA

>D1GEJ1

GADTRNNFTGHLQDKLLGKGIDS‑FIDDR‑‑LRRGDDIT‑ALFDRIEQSKIAIVVFSENYANSVWCLRELVKILQC‑RDR‑NQQLVIPILYKIDKSKLK‑‑‑‑‑‑‑‑‑‑NVR‑KTR‑‑V‑‑‑‑‑TEDEIVSWEAAISTAVDIS‑GYVVDR‑YS‑‑‑‑T‑SEAKLVNDIA

>M4CD72

GGDVRKGFLSHLLKELESKGI‑SPFID‑NNIE‑RGQSIAPELVQAIKESRVALVLLSPNYASSRWCLDELVEIMKCR‑EREQQT‑VITIFYGVDPSDVRNQTGDFGKVFNKTCDGR‑‑‑T‑‑‑EGV‑K‑EAWKKALVDVGN‑IAGY‑‑‑DSSR‑W‑‑D‑NEAKMIEEIA

>M4DZK6

GADVRKNFLSFLTDGLKRACVNY‑YVDTK‑‑ETKGEVLD‑ILLQRIQESRLVLIILSENYMQSNWCIKELRTTTKD‑IKE‑SRRKVIPIFYNVQVADVK‑‑‑‑‑‑‑‑‑‑‑‑‑‑‑‑‑‑‑‑‑‑‑‑‑‑‑‑‑‑DKWKEALMILTRHM‑GMRSDE‑‑Y‑‑‑‑G‑TDCEFIEHIV

>M4EFM2

KEELGDNFVRHLAWALRELGINV‑LMDSY‑‑NRRGDEQQ‑QVFTNIEKSNIVLAIFSKRYSESDRCLNELVKMEEL‑TKE‑GKLVVIPVFYNVKTNEVRRLQGEFGIHFADS‑VKR‑‑‑‑‑‑‑‑EPMMVQSWEEALNFIIK‑‑‑GLSLER‑‑H‑‑‑‑R‑NEFALVAAIV

>M4DNF0

GTDVRKKFISHLNDALTEEGIIT‑FHDDRDLE‑RGNPILKGLEEAMNQSRFAIVVVSEDYATSQWCLRELAFMVELE‑KK‑‑RFDLIPIFYEIDPSALKSRTG‑FNKAFEDH‑EKR‑‑‑‑‑‑DAE‑‑TVRKWRRAVDIVAN‑ISGW‑‑‑DS‑K‑‑‑RS‑DDSKLIQEVV

>M4EFL8

ATELPDDFIKHLVSGLTDLGINI‑FMDRD‑‑DWWGRDLD‑RIFTCIEESTIALVIFSPSYPETEWCLDELVKMKER‑ANN‑NKLLVIPIFFNVSKNDVRNFEGEFGDRFMEL‑RKRYKY‑‑‑‑‑DPFRVQRWETSVMTISR‑‑‑SLTWET‑‑Q‑‑‑‑S‑SSISIAMDII

>M4EQC4

GIDTRT‑FVSHLLLALFKKQFKT‑FR‑EEEMQ‑P‑‑‑‑STQVLEAIENSKIAIVVISKNYTASVSCLDELAKIVEC‑‑EEK‑QVMI‑PVLHEVDPSDVLEQAQNLDGDI‑‑‑‑‑‑N‑‑‑‑‑‑‑‑NNWEVERWRKALASIKQLYSHW‑‑EDS‑SD‑GTDKPPEKMIKGIV

>M4F5S6

GLDVRQTFLSHLLKEFERKGI‑NTFKD‑SQIK‑RGKYISPELKQAIRESRICLIILSKNYASSSWTLGELVEILESR‑KASGKT‑VMTVFYDIDPSHVRKQSGEFGMAFRKTCERK‑‑‑T‑‑‑EHQ‑K‑QRWKQALTNVAS‑ILGE‑‑‑DSHK‑W‑RD‑NEADMISKIA

>M4F4L9

GGDEAKMLERHLQSCFEKYGI‑RTFFD‑RTLE‑IGAVVGPDLIQSLRDSKPIIVVTEKDYNCSNWCLDELVEILKCK‑EASEQN‑VMTIFYDIDPSSVRKQKGDFGSAFKKTCVGK‑‑‑T‑‑‑EEV‑K‑QRWARALTHVAN‑IKGE‑‑‑HSLN‑W‑‑A‑SEAEMIQKIA

>M4DHQ1

GLDTRRNFISFLYKELVRRNIRT‑FKDDKELE‑SGRKISPELERAIQESKFAVVVISANYAASTWCLQELVKIMDFV‑NK‑GSLTVIPVFHGVEPCHVRWQMEKFAVQFEEH‑‑KS‑‑‑E‑‑DRE‑‑KVLSWGHALTNLAN‑ISGH‑‑‑CS‑SEW‑‑E‑DDSMMVDEIA

>M4EE36

GPDVRKGILSHLHIVFERKKI‑TMFKD‑QEME‑RCQQIGSKLIQAIREAKASLVLISKNYASSRCCLDELLEILKCK‑ESSGQI‑VMPIFYDVDPSDVRKQKGDFGITFKTTCQGA‑‑‑T‑‑‑EEK‑K‑QRWIEALTCVAT‑ITGE‑‑‑DSRT‑W‑‑A‑NDAAMLEKIS

>M4EB03

GKDTRRIFISFLYKELIRMSIRT‑FKDDVELK‑SGRRISSDLLLAIEGSKIAVVVVSKKYPASPWCLHELVKIMDVE‑KQ‑GSLTVMPIFYNVEPSHVRRQIEKVAEQFTKH‑EGR‑‑‑E‑‑NHE‑‑TVVSWRQALTNLAS‑ISGH‑‑‑CS‑RDC‑‑D‑DDSKLVDEII

>M4EQC7

GEDTRKTIVSHLYAALDSRGIVT‑FKDDQRLE‑KGDHISDQLHIALKGSSFAVVVLSENYATSRWCLMELQLIMEYM‑KE‑GTLEVFPVFYGVDPSTVRHQLGSF‑‑SLERY‑K‑‑‑‑‑‑‑‑‑VH‑‑KVHKWREALHLIAN‑LSGL‑‑‑DS‑RHC‑‑V‑DEAVMVGEIA

>M4E4E5

GQDVRRTFLSHFLEALKSKGI‑KTFID‑NGII‑RSESINSELIRAIRESRIAVVILSKNYASSSWCLNELQLIMECT‑VSLGQT‑VMTVFYDVEPSDVRKQTGDFGKAFKETCYRK‑‑‑T‑‑‑EEE‑K‑KKWSEALSQVAV‑IAGE‑‑‑HSVS‑W‑‑A‑GEAEMISKIV

>M4DSB6

GEDVRNNFLSHIQKEFKRKGI‑TYFND‑NGIK‑RGESIAPELIRGIRGSKIVIVLLSRNYGSSKWCLEELVEIMKCR‑EELKQT‑VMAIFCKVDPSDVKKLTGDFGKVFRKTCEGQ‑‑‑T‑‑‑KED‑I‑WRWKQVWRR‑‑‑‑‑‑‑‑‑‑‑‑‑‑‑‑‑‑‑D‑DEASMVEEIA

>M4EG66

GPDVRKTLLSHIREQFTRSGI‑TMFDD‑QEIV‑RSATIAPSLTEAIRESRISIVILSKNYASSSWCLNEMVEILECK‑KAMGHI‑VMTIFYGVDPSDVRKQTGEFGIAFNETCASK‑‑‑T‑‑‑KEE‑K‑QRWRQALNEVGN‑IAGE‑‑‑DFLR‑W‑‑S‑NEAKVIKKIA

>M4EFN6

CVDERYSFVSHLSDALRRNVISS‑VF‑GD‑‑‑‑‑‑SE‑AQ‑‑‑GKVERAKVSVVVLP‑‑‑ANRQVCLEKLEKVLNCQ‑RNKEQV‑MIPVLYGDSKLHG‑‑‑‑‑‑‑‑‑‑‑‑‑‑‑‑‑‑‑‑‑‑‑‑‑‑‑‑‑‑‑‑EWLSAM‑NLRG‑LPVF‑‑‑QS‑N‑‑‑‑D‑SDSKLVEKIV

>M4CJ08

GEDVRKTFLSHFLRELERNSI‑VAFKD‑NEME‑RSQSIAPELVQAIRDSRIAVVVFSKNYASSSWCLNELLEILQCN‑EEFGQL‑VIPIFYGLDPSHLRKQTGDFGEAFKKTCLNQ‑‑‑T‑‑‑HEV‑E‑DQWKQALTNVAN‑ILGY‑‑‑HSKN‑C‑‑D‑SEAAMIEEIS

>M4E3J8

GPDVRNGFLSHLYQSLVTSGIYT‑FKDDEELE‑KGESISPELRKAIENSKIHLVVLSESYASSSWCLDELVHMMRRL‑KNNGHL‑VFPVFYKIEPSHVRRQSGPFGESFHKH‑RSRHRE‑‑‑‑‑‑‑‑KLKQWRKALTSIAN‑LKGY‑‑‑‑HSS‑‑‑‑NGNDAELVDQLT

>M4CAD2

GPDVRKGFLSHVRKELKSKGL‑IVFFD‑DEIK‑RGESIDQELVEAIRQSRTAIVLLSPNYTSSSWCLNELVEIIKCR‑EEDRQT‑VLTIFYEVDPSDVRKQTGVFGKLFKKTCVGK‑‑‑T‑‑‑EKV‑K‑KAWKQALEDVAG‑IAGY‑‑‑HSSN‑C‑‑A‑NEADLIKKVA

>M4EI85

GPDVRRKFLSHLHYHFASKGI‑TVFKD‑QEIV‑RGQTIGPELKQAIRESRISMVVLSKNYASSSWCLDELVEILECE‑EACGQK‑VMTIFYDVDPSDVRQQSGDFGRAFDRTCKRQ‑‑‑T‑‑‑EEV‑K‑QIWSKALTDVAE‑IAGV‑‑‑HSLS‑W‑‑D‑DEAKMMQKIV

>M4CI30

GADTRKNFVSFLYKQLETKGIRA‑FKDDNALV‑CGRSIAPVIVQGIKGSTIAVVVISPTYPASFWCLEELVMILKLE‑RE‑KLLTVVPIFYEVEPNDLKRQTGKLVKQFKKH‑EKR‑‑‑H‑‑STE‑‑RVHSWRDALNRLAT‑LSGD‑‑‑CS‑KIS‑‑E‑DDATLVDRVT

>M4EKW4

GFDTRNNFTGHLQKALRLRGIDS‑FIDDK‑‑LRRGDDLT‑ALFDRIEHSKIAIIVFSKNYSNSAWCLRELVKILEC‑RDR‑NQQLVIPILYKVDKSELK‑‑‑‑‑‑‑‑‑‑NVP‑KKS‑‑V‑‑‑‑‑KEEETSTWEAALTTAFNIS‑GYVVNE‑FS‑‑‑‑T‑SEAKLVDEIA

>M4D1Z6

GVDVLENFLSHIVKEFKSNAI‑DLFID‑NNIE‑RSKSIGPALIEAIRGSRVAIVLLSKNYASSTWCLNELVEIMECR‑EEVGQT‑VITIFYQVNPTDIKKQKSYFGKVFRKTCKGK‑‑‑R‑‑‑NEE‑I‑QRWKHALTDVAQ‑IEGY‑‑‑HSIN‑W‑‑K‑NESEMIEYIA

>M4DII4

GEETRHNITKRIYDALNVKEFRV‑FRDNDGLEGGGDETSPNIVEAMKDSAASVVV‑‑‑‑‑‑‑‑‑‑‑‑‑‑‑‑‑‑‑‑‑‑‑‑‑‑‑‑‑‑‑‑‑‑‑‑‑‑‑‑‑‑‑‑‑‑‑‑‑‑‑‑SYALH‑VRNVS‑‑‑‑‑E‑‑‑‑‑‑‑‑‑‑‑‑‑‑‑‑‑‑‑‑‑‑‑‑‑‑‑TPE‑‑‑‑SVDEDMIELVV

>M4EFE8

GEDVRKDFLSHIHMEFQRKGI‑TPFVD‑NEIK‑RGESIGPELVRAIRGSKIAIILISRNYASSKWCLDELVEIMKCR‑EELGQT‑VMAIFYRVDPSDVKKLAGDFGRVFRKTCAGK‑‑‑T‑‑‑KDN‑I‑GRWRQALAKVAT‑VAGY‑‑‑HSSN‑W‑‑D‑NEAAMIKKIA

>M4D879

PEDVYKFFFCRRLSALF‑KDV‑NRYL‑‑‑‑‑‑‑‑‑‑‑‑‑‑‑‑‑‑PIRKA‑‑TNIVFSKSYASSSWCLNELVEIHKCY‑MEVDQT‑VIPIFYGVDPSDVRKQTGEFGKAFGETSKGT‑‑‑T‑‑‑EDE‑K‑QRWMRALAEVAN‑MAGE‑‑‑DLQN‑W‑‑C‑NEANLIDKIA

>M4EZ36

GEDVRKTFLSHLLLSLDRKLI‑TCFKD‑NEIE‑RSQSIGLKLVHAIRDSRIAIVILSKTYASSTWCLNELLEIVKCK‑EDKGQI‑VIPVFYGLDPSHVRKQTGEFGETFQMICKNR‑‑‑S‑‑‑DEL‑P‑DLWKGALTHVAN‑IHGY‑‑‑HSDN‑W‑‑N‑NEAHLIEDIT

>M4D288

GEDVRTNFLSHVLKELKSKAI‑DLFID‑NDIE‑RSKSIGPELIEAIRGSRIAIIFLSKNYASSTWCLNELVEIMTCR‑EEFGQT‑VISLFYEVDPTHVKKQTGDFGKVFKKTCVGK‑‑‑T‑‑‑KDE‑I‑QRWKHALTEVAQ‑IAGF‑‑‑HSSN‑W‑‑E‑TEAKMIEVIA

>M4F6T1

CEDKRYSFISHLSAAFHRRGISS‑YI‑DP‑‑‑‑‑‑DL‑SK‑‑‑GDMEKSKACVVVFSEKYSSSKPCLEELVKVSERR‑YEGGHA‑VVPVFYRATKSSVKKLIWKSS‑‑‑‑‑‑‑‑‑‑‑‑‑‑‑‑‑‑D‑‑LTSERRSALLEVVD‑LPGH‑‑‑ES‑V‑‑‑‑T‑SESDLVEEIV

>M4FEG2

GADVRKSFLSHILKEFRSKGI‑DTFID‑DDIE‑RNKSIGPQLIDAIKGSKIGIILLSKNYASSSWCLNELVEIMKCR‑TELGQT‑VMTIFYEVDPADVKKQRKDFGKSFRKTCKGK‑‑‑T‑‑‑SDE‑I‑ETWKKALEGVAT‑IAGY‑‑‑HSNN‑W‑‑D‑NEAAMIEKIA

>M4F6T9

GEDVRTSLLTHILKEFKSKAI‑YPFID‑DKMK‑RGKIIGPELKKAIQGSRIAVVLLSKNYASSSWCLDELAEIMKCQ‑EELDQM‑VIPILYEVNPSDVKKQRGDFGKVFKKTCEGK‑‑‑T‑‑‑NEV‑I‑EKWSQALSKVAT‑ITGY‑‑‑HSIN‑W‑‑N‑DDAKMIEDIT

>M4D252

GKQLRYGFVSHLEKALRRDGINV‑FVDKN‑‑ETKGKDLS‑SLFSRIEESRIALAIFSSMYTESKWCLNELEKIKEC‑VDL‑GKLVVIPIFYKVDTDDVKNLNGVFGDKFWEL‑AKT‑‑C‑‑‑‑‑NGEKFEKWRQALQNIPQKL‑GFTLGE‑‑T‑‑‑‑S‑DEGDYINQIV

>M4E8F1

VKDTRDNFVSHLCGCLRRKRIKT‑YLD‑‑ELE‑R‑EE‑‑‑SLK‑AIE‑SRVSVIVFSENFGDSKFCLDEVVAILKCK‑KR‑GQI‑VIPVLYHVDRVDIENQTGSFGEAFAKR‑QDK‑‑‑A‑‑‑‑D‑‑QIKEWKDGFTE‑AN‑LPGW‑‑‑‑STS‑H‑RD‑‑EEMLVNGIA

>M4D5A9

GKQLRNGFVSHLEKALRKDGINV‑FIDKN‑‑ETKGKDLS‑ILFSRIEESRIALAIFSTLYTESNWCLNELEKIKEC‑VDL‑GKLVVIPIFYKVETDDVKNLKGVFGDKFWEL‑AKT‑‑C‑‑‑‑‑RGEKLDKWKEALEDVPKKL‑GFTLSE‑‑M‑‑‑‑S‑DEGEYISKIV

>M4E4E4

GEDVRRNFLSHFHKELQLNGI‑DAFKD‑GGIK‑RSRSIWPELKQAIWESRVSIVVLSKNYGGSSWCLDELVEIMECK‑EVSGQT‑VMPIFYGVDPTDVRKQSGDFGKSFDTICHVR‑‑‑T‑‑‑EEE‑R‑QRWKQALTSVAS‑IAGD‑‑‑CSSK‑W‑‑D‑NDAVMIERIV

>D1GEH3

GKDTRADFAERLYTEI‑KREVKI‑FRDNEGME‑RGEEINASLIAGMEDSAASLVLFSPHYADSRWCLDELATLCDLS‑SSLDRP‑MIPIFYKVDPSHVRKQSGDF‑KHFEAH‑AERFS‑‑‑‑‑KE‑‑RIQPWREAMKLVGH‑LPGF‑‑‑‑IYRE‑‑‑‑ENEDALIRLVV

>M4EG67

GPDVRSSYLSHLRKQFERNGI‑ITFND‑QEIE‑RSQTIKPELTRAIQESRISIVVLSQNYASSSWCLNELVEILDCK‑AT‑GQI‑VMTVFYKVNPSDVRKQIGGFGKAFKETCQGK‑‑‑T‑‑‑ETE‑I‑QSWSKALTYVAN‑IEGE‑‑‑HSLN‑W‑‑V‑NEADMIEKIA

>M4DBU9

GEDVRKTFVSHLFCELDRMGINA‑FRDDLDLE‑RGKSISPELVDVIKGSRFAIVVVSRNYAASSWCLDELLKIMEC‑‑KDAEQT‑IIPIFYEVDPSDVRRQHGSFGEDVESH‑DKK‑‑‑‑‑‑‑‑‑‑‑KVKKWKEALTILAA‑ICGE‑‑‑DS‑R‑‑‑‑NRDESKLIKKIV

>M4E4E6

SKDVGRTFLSHFLEGLKSKGI‑KTFQN‑NGIM‑RSEYITTELARAIEESRISVVILSKNYPSSSWCLNELQRIMKCK‑VSLGQI‑VMAIFYDVDPSDVREQTGDFGKVFEETCYGK‑‑‑T‑‑‑DEQ‑K‑KKWRKALSHVAV‑IAGE‑‑‑HSIS‑W‑‑A‑SEAEMISKIV

>D1GED4

GPDTRKNFVSFLYKELVAKEIRT‑FKDDKELE‑RGRLISPELLQAIEGSEIAVVVVSKTYSASNWCLEELVKILKLE‑KK‑GLIKVLPIFYEVDPSHVRWQREEVAKQFKKH‑EKR‑‑‑Q‑‑SRE‑‑KVKSWRDALNYLAE‑LSGE‑‑‑CS‑KNW‑‑E‑DDSKLVDGIT

>D1GEI5

GADTRHSFTCYLLDFLRRKGIDA‑FIDEE‑‑LRRGNDLS‑GLLERIEQSKISIVVFSENYANSAWCLEELAKIMDC‑KRT‑FDQVVLPVFYKVPASDVRYQTGKFGAPFERS‑EEV‑‑S‑‑‑‑‑EH‑RVPAWKEALRAASDIA‑GYVLPE‑‑R‑‑‑‑S‑PECDFVDKIA

>M4FFC5

GEDVRKGFLSHVRKGLESKGI‑IAFVD‑EEIK‑RGESVCTVLVGAIRQSRVAVVLLSPNYASPSWCLDELVEIMKCR‑EEYQQT‑VMTIFYEVDPSDVRKQTGDFGKAFDATCVGK‑‑‑I‑‑‑EEV‑K‑QAWRQALTDVAG‑IAGY‑‑‑HTSN‑C‑‑D‑NEAEMINKVA

>M4DSB7

GEDVRKNFLSHIKKEFKRKTI‑TFFND‑NGIE‑RGESIAPELIRGIRGSKIAIVLLSKNYASSKWCLEELVEIMNCR‑EELGQT‑VMAIFYEVDPSDVKKLNGDFGKVFRKTCEGK‑‑‑S‑‑‑KED‑I‑RRWKQALEKVAT‑IAGY‑‑‑HSCN‑W‑‑V‑DEAAMIEDIS

>M4EBB9

GDELRNNFISHLVDALRRNTINV‑FIDKE‑‑EKKGEDIN‑NLFKRIEESKIAVAVFSRRYTESRWCLDELVKMKER‑ADL‑GKLKIFPIFYNVTTYDVKLREGDFGIHFRRL‑KREYRS‑‑‑‑‑EQHRVGKWKEALACVSGKT‑GLTFND‑‑‑‑‑‑‑K‑SESDFINNIV

>M4CI31

GADTRKNFVSFLYKELETKGIQT‑FKDDKALV‑RGRPIAPELVQAIQGTRIAVVVVSPTYSASYWCLEELVKILKLE‑KK‑GLLVVVPIFYEVDPCQVRRQKGEVAEQFKKH‑KRR‑‑‑Y‑‑SRE‑‑RVRSWRNALTRVTI‑LSGD‑‑‑CS‑KNC‑‑K‑DDATLVDGIT

>M4E9L1

GPDVRRTFLSHLHREFVSKGI‑VAFKD‑KEIE‑RGHTIGPDLVQAIRESRVSIVVLSKNYASSGWCLDELVEILKCK‑EDQGQV‑VMTIFYDVNPSDVRKRCGDFGRAFETTCQGK‑‑‑T‑‑‑EEV‑K‑QRWNKALTDVAD‑IAGE‑‑‑HYLN‑W‑‑E‑DEGEMVENIA

>D1GEG8

GQDVRKTFLSHFLEGLKREGI‑NTFID‑NGIT‑RSESINSELVRAIKEARIAIVILSNNYDSSSWCLNELQLIMECR‑LALGQT‑VMTIFYEVDPSDVRKQTGDFGKAFEETCDGK‑‑‑T‑‑‑EEE‑K‑HRWRQALTQVAV‑IAGE‑‑‑HSVS‑W‑‑A‑SEAEMMLKIV

>M4EG62

GPDVRKTLLSHMREQFKRSGI‑TMFND‑QEIV‑RSQEISPSLTNGIRESKISIVILSKKYASSSWCLDELVEILKCK‑ETMGQI‑VMTIFYGVEPSDVRFQTGDFGIAFNETCADK‑‑‑P‑‑‑DEE‑R‑QKWSKALKDVGN‑IAGE‑‑‑DFQK‑W‑‑D‑NEANMIKKIT

>M4E4G7

GEDVRKGFLSHVRKGLERKGI‑IAFVD‑DKIE‑RGESVGPVLVGAIRQSRVAVVLLSRNYASSSWCLDELVEIMKCR‑KEDQQK‑VMTIFYEVDPSHVRKQTGDFGKAFEKTCMGK‑‑‑T‑‑‑EEV‑K‑QEWRQALEDVAG‑IAGY‑‑‑HSSN‑S‑‑D‑NEAEMIDKVA

>M4E797

GEDTRHTITERVYDALHRKEVRV‑FRDDEGMQ‑RGDEINPSLVAAMEDSAASVVVLSPRYADSHWCLDELATLCDLR‑ASLRRP‑MIPIFYEVDPSHVRKQNDHF‑KDFEVH‑AKRFK‑‑‑‑‑EE‑‑KIQRWRKAMTLVGN‑LSGF‑‑‑‑VCKE‑‑‑‑SVDDEMIGLLV

>M4DZK4

GEELRCGFVSHLVEALQRHGINV‑FIDKL‑‑ESVGQDLS‑NLFARIEESTIALVIFSRRYTESRWCLDELVKIKER‑AAQ‑GLLKVIPIFFKVEPVTVKQLRGAFGDKFRDR‑EWEYRC‑‑‑‑‑DKPRTGRWKEALASVSCKT‑GLTFDR‑‑K‑‑‑‑T‑NESTFVRIIV

>M4D9I4

GPDVRRGFLSHLHNVFAKKEI‑TVFND‑QKIE‑RGHTIGSELVLAIREAEASIVLLSQNYASSSWCLDELVEILKCK‑EASGQI‑VMPIFYDVDPSDVRKQKGGFGIAFEKTCEGE‑‑‑T‑‑‑EEQ‑K‑QRWVDALTYVAT‑IAGE‑‑‑HSRN‑W‑‑T‑DEAVMVEKIS

>M4F229

GTDSRRSFVSHLYEALTKEGIKA‑FHDDRELT‑RGGFIWKELVKAIEESRFAVVVLTEGYATSRWCLEELSLIVDLS‑KK‑‑RLELIPVFLDIDPSELKRRNGSFEKALAKH‑ELR‑‑‑‑‑‑DLE‑‑TVGRWRKALAEVGN‑ISGW‑‑‑DS‑K‑‑‑RS‑EEAVLVQEVV

>M4DLL0

GEQLRHSFVSHLTDAFERHGINF‑IVDKY‑‑EQRGKDLK‑NIFARIEESSIALAIFSTRYPESSWCMDELVKMKKL‑ADK‑GKIQVIPIFYKVSARDVRRQTGKFGDKFWNL‑ARASIT‑‑‑‑‑SGDQIKKWKEALECISGKM‑GLSLKN‑‑K‑‑‑‑R‑YEMDMLQEEA

>M4DII7

SEE‑‑NDFSERLYNAL‑RKEVRV‑FR‑NEGME‑QGDEDNKRLFKAMEDSAASVVVFTQHYADSRSCLDELATLCDLG‑TSLDRP‑ILPVFFKVDPSHVRKQNDHF‑KDFDEH‑KKIFS‑‑‑‑‑KE‑‑EVQRWRKAMELVGN‑LAGY‑‑‑‑VYKL‑R‑‑KDEDDTIKLVV

>M4ET25

GADVRRGFLSHLLKEFKREAI‑DTFVD‑NNIE‑RGKSIGPRFIKSIRGSKIAIVLLSKNYASSTWCLNELAEIMSCR‑KDLGLI‑VMVIFYEVDPSDVKKLTGHFGRVFRKTCAGK‑‑‑I‑‑‑KDD‑I‑VRWRQALAKVAT‑IAGY‑‑‑HSTN‑W‑‑D‑NEAAMIEQIA

>M4DZK7

GAQLRHNFIDHLVNAMKGRGINV‑FIDTD‑‑EQKGKDIK‑ILLKRIEESRVTLAIFSTKYTESSWCLDELATIKKR‑VDL‑G‑‑‑‑‑‑MLEKVLNNLWESLATTLG‑‑‑‑‑‑‑‑HEYRC‑‑‑‑‑EKSKIDEWKKALECVSGKI‑GFTLDE‑‑K‑‑‑‑S‑SESNFIGLII

>M4CD36

GEDVRKNFLSHFHKELKLKGN‑DTFKD‑DGIK‑RSTSIWPELKQAIWESRISIVVLSMNYAGSSWCLNELVEIMECR‑EVSGQT‑LMPIFYEVDPSDVRKQKGEFGKAFEKICAGR‑‑‑T‑‑‑VEE‑T‑QRWRQALTNVGS‑IAGE‑‑‑CSSN‑W‑‑D‑NDAEMIEKIV

>M4CI33

SEDTAKTFVTDLFSSLSEKGITTYYKDEKLEE‑GVSSSGSDLSKCIRDSKLAVVVVSESYPTSVLCLNELQTILNLQ‑DE‑GQLSVLLIFYGVDTSNIRKQTGEYAEPFRKL‑GEE‑‑‑Y‑‑SAE‑‑KVQSWRRTLTKLTG‑ISGL‑‑‑DS‑RFW‑‑S‑REAEMVDLIT

>M4DZX4

GEDVRITFLSHFLKELDKRLI‑IAFKD‑NETQ‑RSLSLGPELKQAIRDSRIAVVIFSNKYASSSWCLNELLEIVKCR‑EECGQM‑VIPVFYRLDPSHVRKQTGDFGNIFEETCKNK‑‑‑T‑‑‑EEV‑I‑IQWRRALTDVAN‑TLGY‑‑‑HSVN‑W‑‑D‑SEAKMIEEIV

>M4F5R1

GKDVRKSFLSHFLKEFGSKGI‑NLFID‑NEIT‑RGEYIGPELKKAIQGSRIAIVLLSKRYASSSWCLDELVEIMKCK‑EELGQT‑VMPVFYEVDPTDVKKQAGDFGKVFKKTCKGK‑‑‑T‑‑‑NEV‑T‑RKWSEALAKVAT‑LAGY‑‑‑HSNN‑W‑‑D‑NEAKMIEDVA

>M4EFN7

GKDLRRGFVGFLVDALKREKINV‑FMDEF‑‑EERGKPLD‑SLLTRIEGSRVAVAIFSENYTESNWCLKEAEKMNEC‑REK‑GNLVVIPIFYKVEPSTVRGLKGDFGYKLWIL‑AKG‑‑‑‑‑‑‑‑DE‑KRKKFDEALESIPNLF‑GITVDD‑‑T‑‑‑‑S‑DECQKINDIV

>M4CAD1

GEDVRKGFLSHVLKEFKSKGI‑NVFID‑NEIK‑RGESVGPELVKAIRHSRVGVVLLSRNYASSSWCLDELVEIMKCR‑EEVGQT‑VMTIFYNVDPSEVRKQTGDFGKAFDETCVGK‑‑‑T‑‑‑EEV‑K‑KAWRQALNDVAG‑IAGY‑‑‑HSSN‑C‑‑G‑NEADLINKVA

>M4CAE6

GVDVRVTFLSHLLKEFDKKLI‑TAFKD‑NEIE‑RSRSLDPELKQAIKDSRIAVVIFSQNYASSSWCLNELLEIVK‑‑‑‑‑CGQM‑VIPVFYRLDPSHVRKQTGDFGKIFEETCKNQ‑‑‑T‑‑‑EEVII‑IQWRRALTDVAN‑TLGY‑‑‑HSVN‑W‑‑G‑NEAAMIEEIA

>M4D2G3

GADVRTNFLSHVLKELRSKGI‑NSFID‑DDME‑RSKLVGLELIEAIRGSRIAIVLLSRNYASSTWCLNELVEIIKCR‑QEFSQT‑VIPLFYEVDPTDVKKQTGDFGKVFRKTCKGK‑‑‑A‑‑‑KED‑I‑QRWKCALTEVAQ‑ITGY‑‑‑HSTN‑W‑‑K‑TEAKMIEDIA

>M4DJB6

GGDTRKNIISHLHKELVRRGIRT‑FKDDETLE‑TGDRFPERLREAINTSRFAIVVISKNYASSRWCLEELRMIMKLQ‑RE‑KNIAVIPVFYEVDISDVRNHRSGF‑‑GLVQH‑H‑‑‑‑‑‑‑‑KDP‑‑KIPFWKDALRGIAN‑TQAT‑‑‑ES‑RKC‑‑K‑DDATLVEGVV

>D1GEB3

GPDTRKIFVGHLYGSLSIRGIFT‑FKDDRRLE‑PGDSITDELCQAIRTSRFAVVVISKNYATSSWCLDELQLIMELV‑EN‑KEIEVFPIFYEVKPSDVRHQQESF‑‑SL‑‑‑‑R‑‑‑‑‑‑‑‑MTE‑‑KVPGWKKALKDIAN‑RKGM‑‑‑ES‑SKF‑‑S‑DDATMIEEIV

>M4CV08

GPDTRKNFVSHLYAALCRKGLYT‑YKDDEEME‑KGGLIPDELIKAIKTSRFFIVVISENFDNSYWCLEELRAIMEVE‑AV‑KRKELIPIFYRVKPGRINRENAAF‑‑SDMKH‑‑‑‑‑‑‑E‑‑ETA‑‑MINEWENTLSQLAN‑RASY‑‑‑IF‑STS‑‑V‑DEATRIEEVV

>M4CQG9

RPDVGKTFISHLQKQFTSNRITM‑FD‑DEGVE‑RGHTI‑PALTQAIRESTISIVVLTKNYASSSWCLDGLLEILKCR‑QA‑KLI‑VMPVFYGVRPNDVQRQTGDFGKGFEKTCRGK‑‑‑T‑‑ILD‑‑K‑GRWSQALNKVCS‑LQGF‑‑‑MFSP‑R‑‑D‑DESELFEKIG

>M4DRY0

GEDLRKNFLSHFLKELQRKGI‑TTFID‑HEIK‑RSKAIGPELVAAIRGSRMAVILLSKNYASSTWCLNELLEIMSCK‑EEIGQT‑VMPVFYEVDPSDVRKQAGDFGNIFEETCLGK‑‑‑S‑‑‑EEV‑R‑QRWSRALTDLAN‑LAGV‑‑‑DSRL‑W‑‑N‑NEADMIEKLA

>M1BK10

GEDTRKNFTDHLYTALINAGIRT‑FRDDDEIR‑RGENIESELQKGIRESKISLVVFSEDYGSSRWCLDELVNILNRR‑KKEGHT‑VLPVFYTVSPEDVQNQTGSFAKAFVNH‑EKRRKEW‑‑‑ME‑‑KMEKWRVALKEVAE‑LEGM‑‑‑CL‑AK‑‑VDGHEAKFIQKII

>M1BKV7

GEDTRRTFTSHLYEGLKNRGIFT‑FQDVKRLD‑HGDSIPEELVKAIKESQISLVVFSKNYGASRWCLNELVKIMECK‑K‑NEQT‑VIPVFYDVDPSHVRNQRESFGEAFSKH‑ESKYKDDE‑GMQ‑‑KVQRWRTALTVAAN‑LKGY‑‑‑DI‑‑‑‑‑RDG‑ESEKIQQIV

>M1C838

GEDTRKTFVGHLYYALKQKGIHT‑FKDDERLE‑RGKSISPELVKAIEESRFAIVVFSKNYASSTWCLDELGKIMECK‑KDFGQT‑VIPIFYDVDPSHVTKQSESFAKSFATH‑EENLKDD‑‑‑VE‑‑KVLCWRDAFRQAGK‑IAGY‑‑‑DL‑PK‑‑YDGHESLCIQHVV

>M1BJT4

GEDTRKTFTDTLYAALVGAGWRT‑FKDDNETE‑RGENIKTELENAIINSRSSIIIISKNYATSTWCLDELVKILEHK‑RTKGHA‑VLPVFYHVDPSEVRDQKKSFAEAFASY‑ERQIKADEGKRE‑‑KVRKWRAALGEVAD‑SGGV‑‑‑‑LVN‑‑‑‑NQKESEFIEEIL

>M1A5Z0

GEDVRKTFVDHLYVALQQKGINT‑FKDSEKLE‑KGNSISPGLMRAIEESRISLIIFSKNYANSRWCLDEVAKIMECK‑NVKGQI‑VIPVFYDVDPSTVRKQKSSFEEAFNNY‑EDCF‑‑‑‑‑‑‑‑‑‑KVQKWRGALEEAAN‑LSGW‑‑‑DL‑PN‑‑SNAHEAIVIKQIV

>M1CNT1

GKDVRKTFLDHLYKAL‑‑‑‑INV‑FRDDDEL‑‑RGEDIS‑SLHEAIEESIISLVVFSKSYASSKWCLNELVKILECK‑ENFGQF‑IYPIFYDVDPSEVRHQTAQIGDSLAKH‑E‑N‑‑‑T‑‑‑‑E‑‑QLWKWRAALTAVAS‑LSGF‑‑‑HLPN‑F‑‑NGHEAKFIEVII

>M1ACW5

GEDTRKNFTSHLYFRLCQVGVNT‑FIDDEELR‑KGEVISVKLEKAIEESRVAIVVFSKNYASSSWCLDELVKILDCR‑ERLNQV‑VLPIFYDVDPSQVRRQTGSFGEALAKH‑KERL‑FG‑‑‑AQ‑‑RVEKWKAALTEAAN‑LSGW‑‑‑NL‑QN‑‑ADGHESKFIEGII

>M1BK09

GEDTRKNFTDHLYTALINAGIRT‑FRDDDEIR‑RGENIESELQKGIRESKISLVVFSEDYGSSRWCLDELVNILNRR‑KKEGHT‑VLPVFYTVSPEDVQNQTGSFAKAFVNH‑EKRRKEW‑‑‑ME‑‑KMEKWRVALKEVAE‑LEGM‑‑‑CL‑AK‑‑VDGHEAKFIQKII

>M1CVI4

GIDTRRNFVSHLYNALEQRGIDV‑FKDDERLE‑TGKSIPNELMKAIEESRFAIVIFSESYASSRWCLEELAHIIKCR‑NELDQI‑VIPIFYDVSPSDVSHQNSPFAESFS‑‑‑‑‑KYKDD‑‑ELE‑‑KVQRWREACKEAGK‑ISGY‑‑‑HL‑QE‑‑KD‑‑EANCIKKVV

>M1C298

GEDTRDNITNNLYNALYSKGIRV‑FRDSEGLT‑QGDEISTGLIEAINDSAAVIAIISPNYASSRWCLEELATIYELG‑‑‑‑‑KL‑VLPLFYGVDPSDVRRQLGPF‑DGFRDL‑ERKFS‑‑‑‑‑PE‑‑KMVRWRNAMERVGG‑VSGW‑‑‑‑VYDN‑G‑‑‑DESQLIQLVV

>M1BF54

GEDTRRNFTSHLYQGLKNRGIFT‑FLDDKRLE‑NGDSISEELGKAIEVSQVAVIVFSRNYAMSRWCLNELVKIMECK‑KENGQI‑VIPIFCDVDPSHVRYQSKSFEEAFAKH‑ESRYKDDE‑GMQ‑‑KVQGWRTALTAAAN‑LKGY‑‑‑VF‑‑‑‑‑PNGSDADCIEHIV

>M1BK04

TK‑TGKSFGNHLHSALSNAGIRA‑FSVDELD‑‑‑IDEKGKELQKTIQESRILIVVLSKDYTSSERCLDELVFILESK‑KLFGRF‑VLPVFYDVDPSEVRKQKGSFEQDFLMY‑EQRYRSEERRLE‑‑KVKEWKASLTEVAD‑LGGM‑‑‑‑VLQ‑‑‑‑NQCESRFIQEIV

>M1BGG4

GEDIRKTFVDHLYLALQQKCINT‑FKDDEKLE‑KGKFISPELESSIEESRIALIIFSKNYANSTWCLDELTKIMECK‑NVKGQI‑VVPVFYDVDPSTVRKQKTIFGEAFSKH‑EARFQ‑‑‑‑‑ED‑‑KVQKWRAALEEAAN‑ISGW‑‑‑DL‑PN‑‑ANGHEARVIEKIA

>M1C837

GEDTRKTFVGHLYYALKQKGIHT‑FKDDERLE‑RGKSISPELVKAIEESRFAIVVFSKNYASSTWCLDELGKIMECK‑KDFGQT‑VIPIFYDVDPSHVTKQSESFAKSFATH‑EENLKDD‑‑‑VE‑‑KVLCWRDAFRQAGK‑IAGY‑‑‑DL‑PK‑‑YDGHESLCIQHVV

>M1B043

GKEISVNFINILHTALTDVGIQT‑FKRHSEAR‑KGKIVGSELQKAVKESRISIIVFTEDYGYSRRCLDELVNILERK‑QSAGHM‑ILPVFYRVDPSHIRKQRESYAKAFHNY‑EEQIMAVERRNE‑‑KIKIWRSSLTEVAN‑MAGI‑‑‑‑VLE‑‑‑‑DG‑ELKFIQEIV

>M1B044

GKEISVNFINILHTALTDVGIQT‑FKRHSEAR‑KGKIVGSELQKAVKESRISIIVFTEDYGYSRRCLDELVNILERK‑QSAGHM‑ILPVFYRVDPSHIRKQRESYAKAFHNY‑EEQIMAVERRNE‑‑KIKIWRSSLTEVAN‑MAGI‑‑‑‑VLE‑‑‑‑DG‑ELKFIQEIV

>M1BZB1

GEDTRDTITGYLYERLTRRGIIT‑FQDNKRLE‑HGDSILEELSKAIKDSQVALVIFSKNYATSRWCLDELVKIMECT‑DENEKA‑IIPVFYGVDATDVRYQSKSFAEAFAKH‑ELKYKDDE‑GMQ‑‑KVQRWRTALTAAAN‑LKGY‑‑‑VF‑‑‑‑‑PNG‑ESDCIDRIV

>M1CV71

GEDTRKTFVSHLYNALIQGRIDV‑FKDDERLE‑TGNSISDELPKAIEESKFAIVIFSESYASSKWCLDELAHIIKCR‑KELKQI‑VIPIFYNVDPSDVRHQTQTFAESFSQH‑EEKYKDD‑‑‑ME‑‑KIQRWRDAFAESGK‑ISGY‑‑‑HL‑QN‑‑KD‑‑EADCIKKVV

>M1CV70

GEDTRKTFVSHLYNALIQGRIDV‑FKDDERLE‑TGNSISDELPKAIEESKFAIVIFSESYASSKWCLDELAHIIKCR‑KELKQI‑VIPIFYNVDPSDVRHQTQTFAESFSQH‑EEKYKDD‑‑‑ME‑‑KIQRWRDAFAESGK‑ISGY‑‑‑HL‑QN‑‑KD‑‑EADCIKKVV

>M1BF53

GDDTRNNFTSHLNKGLENRGIAT‑FLDDERLE‑DGDSISEELMQAIEESQVARIVFSKNYAKSSWCLNEIVKIMECK‑KENRQT‑VIPIFYYVNPSHVRYQSESFAEAFAKH‑ESRYKDDE‑GMQ‑‑KVQGWRNALTAAAD‑LKGH‑‑‑DI‑‑‑‑‑HDGNQSKKIDQIV

>M1AKZ2

GEDTRKTFTGHLFKGLNNRGIFT‑FQDDKRLE‑YGDSIPKELMKAIKESKVALVVFSKNYATSRWCLDELVKIMECK‑K‑NGQT‑VIPIFYDVDPSHVRNQRESFAEAFSQH‑ELKYKDDE‑GMQ‑‑KVQGWRTALTAASN‑LKGY‑‑‑DI‑‑‑‑‑RDG‑ESENIQQIV

>M1BKQ8

GEDTRKTFTGHLYEGLKNRGIST‑FQDDKRLE‑HGDSIPKELLRAIEESQVALIVFSKNYATSRWCLNELVKIMECK‑EENGQT‑VIPIFYDVDPSHVRNQSESFGAAFAEH‑ELKYKDDE‑GMQ‑‑KVQRWRNALTVAAN‑LKGY‑‑‑DI‑‑‑‑‑RDG‑ESENIQQIV

>M1CV72

GEDTRRTFVSHLYNALIQGRIDV‑FKDDERLQ‑TGKSISDELPKAIEESKFAIVIFSESYASSKWCLDELAHIMKCQ‑KELKQI‑VIPIFYNVDPSDVRHQTQTFAESFSQH‑EEKYKDD‑‑‑IE‑‑KIQRWREAFEESGK‑ISGY‑‑‑HL‑QN‑‑KD‑‑EADCIKKVV

>M1C8N7

GEDTRKTFMSHLYEGLKNRGIFT‑FQDDKRLE‑HGDSISEELLKAIEDSQVAILIFSKNYATSRWCLNELVKIMECK‑DENGKT‑VIPIFCDVDPSDVRNQRKIFAEAFAKH‑ESNYKDDE‑GMQ‑‑MVNGWKIALTAAAN‑LKGY‑‑‑DV‑‑‑‑‑CDG‑ESENIQKIV

>M1A5Y9

GEDVRKTFVDHLYVALQQKGINT‑FKDSEKLE‑KGNSISPGLMRAIEESRISLIIFSKNYANSRWCLDEVAKIMECK‑NVKGQI‑VIPVFYDVDPSTVRKQKSSFEEAFNNY‑EDCF‑‑‑‑‑‑‑‑‑‑KVQKWRGALEEAAN‑LSGW‑‑‑DL‑PN‑‑SNAHEAIVIKQIV

>M1CI75

GEDTRNNFTGHLYFRLCQVGVNT‑FIDDEELR‑KGDVISIELEKAIEQSRVAIVVFSKNYASSSWCLDELVKILDCR‑ERLNQL‑VLPIFYDVDPSQVRRQTGSFGEALAKH‑KERS‑IG‑‑‑TE‑‑RMEKWKAALTEAAN‑LSGW‑‑‑DL‑RN‑‑ADGHESKFIESII

>M1APH0

GEDVRKNFVDHLYTALQQRGIHT‑FKDDEKLE‑RGKSISPSLFKAIEESMISIIIFSQNYASSSWCLDELVKITQCM‑KLRGQI‑VLPVFYDVDPSVVRKQKANVGEFFAKH‑ELDFKDD‑‑‑EE‑‑RVKRWRTAMTEAAN‑VSGW‑‑‑DL‑PN‑‑ANGHESKCIEQIV

>M0ZSQ5

‑‑‑‑GNTFADHLYEALAGAGFVT‑LRGGDGNE‑GGEEIKLKLRKGVEESGISIIIFSNDYVSSSLCLDELVMILNCS‑KRRS‑‑‑VLPIFYHVDPSDVRKQKGRIGEAFDRY‑EEA‑‑‑‑‑‑‑‑‑‑‑KVRKWKEALKQVAD‑LGGM‑‑‑‑VLQ‑‑‑‑NQHESKFIQKIL

>M1BA43

GVDTRRTFTGHLYEGLENSAICT‑FQDDKRLE‑IGDSIPEELLKAIEESQVALVIFSKKYATSRWCLNELVKIMECK‑E‑KGLI‑VIPVFYDVYPSVVRKQKKSFKVAFDQH‑ESEYANDE‑GKQ‑‑KVKGWRTALSAAAD‑LKGC‑‑‑NV‑‑‑‑‑HRR‑ESECIKELV

>M1C2N4

GKDTRKTFTSHLYQGLKYKGILT‑FQDDKRLE‑HGDSISEELLKAIKESQVALVVFSKNYATSRWCLNELVQIMECY‑DENGKT‑VIPVFHDVDPSHVRYQSESYAEAFAKH‑ELQFKDDE‑GMQ‑‑KVKRWRTALCEAAD‑LKGH‑‑‑DI‑‑‑‑‑RQG‑ESENIQLIV

>M1D3I2

GDDTRKNFVAHLYKRLQDIGINV‑FKDDVKLE‑RGKFISTELLKAINESRTAIIIFSEDYASSTWCLEELTMIMDCV‑DKKEQ‑‑VYPVFYNVEPSDIRMESSSFAKALEKHNEKRIKDNKDNLE‑‑KVQRWKDALHRAAG‑IAGL‑‑‑DVRK‑‑‑ANGNEADSIDKII

>M1B2A0

GEDTRKSFVDHLYTSLREKGIHT‑FRDDKELS‑RGKSISPELLNAIEKSRFAVVIFSKNYADSSWCLEELTKIVECN‑QQRGQT‑LIPVFYSVDPSVVRKQKESYGDAFAKH‑EENLKGSE‑‑RN‑‑KIQRWRDALKDAAN‑ISGF‑‑‑DV‑QH‑‑EDGHESRCIRQIA

>M1D0W4

GEDTRKTFTSHLYEGLRNRGIFT‑FQDDKRLE‑HGDSIPQELLKAIIESQVALVVFSKNYATSRWCLNELEKIMECK‑E‑NGQM‑VIPVFYDVDPSH‑‑‑‑‑‑‑‑‑‑‑‑‑‑‑‑‑‑‑‑‑‑‑‑‑‑‑‑‑‑KVQGWRTVLTAAAN‑LKGY‑‑‑DI‑‑‑‑‑RDG‑ESENIQQIV

>M1BF44

GEDTRRNFTSHLYQGLDNRGIFT‑FLDDKRLE‑EGDSISEELVKAIEESQVAIIVFSKNYATSGWCLNELVKIMECK‑KENGQT‑VIPVFCYVDPSHVRHQSESFAEAFVKH‑ESRYKDDE‑GMQ‑‑KVQGWRNALTAAAD‑LKGY‑‑‑DI‑‑‑‑‑RDG‑EAEYIQQIV

>M1BA17

GADTRRTFTCHLYEDLKNRGIFT‑FQDDKRLE‑NGDSIPEELLKAIEESQVALVIFSKNYATSRWCLNELVKIMECK‑E‑KGLI‑VIPVFYDVYPSEVRKQTNSFADAFTEH‑ESEYANDE‑GMQ‑‑MVKGWRTALSAAAD‑LKGC‑‑‑NV‑‑‑‑‑HGR‑ESDCIGELV

>M1CV73

GEDTRKTFVSHLYNALIQGRIDV‑FKDDERLE‑TGNSISDELPKAIEESKFAIVIFSESYASSKWCLDELAHIIKCR‑KELKQI‑VIPIFYNVDPSDVRHQTQTFAESFSQH‑EEKYKDD‑‑‑ME‑‑KIQRWRDAFAESGK‑ISGY‑‑‑HL‑QN‑‑KD‑‑EADCIKKVV

>M0ZV73

GEDTRKTFVSHLYDALHRKGIHV‑FKDDERLE‑TGKSISDELLKAIEQSRIAIVIFSKSYASSTWCLKELAHIIKCR‑NELDQN‑VIPIFYDVSPSDVRLQNPPFAEAFSQH‑GEEFKDD‑‑‑AE‑‑KIKNWKDAFVVAGK‑IAGH‑‑‑DL‑KT‑‑KD‑‑EADCIKKLI

>M1BVN8

GEDTRKNFISHLKFRLCQVGICT‑FIDDEEVR‑KGEVISTELEKAIEQSRVSIVVFSKKYASSSWCLEELVKILECR‑ETLKKV‑VLPIFYDVDPSQVRNPIGYFDESLT‑‑‑‑‑RR‑FG‑‑‑AQ‑‑RTEKWKTALTKVAN‑LSGW‑‑‑DS‑RN‑‑VYGHESELIESII

>M0ZR09

GVDSRRTFTGHLYEGLKNRGIFT‑FQDDKRLE‑NGDSIPEELLKAIEESQVALVIFSKNYATSRWCLNELVKIMECK‑E‑NGQI‑VIPVFYDVYPSEVRKQTKSFAEAFAKH‑EKKYNNDE‑GIQ‑‑KVKGWRTALSHAAD‑LKGC‑‑‑NI‑‑‑‑‑HDR‑ESECIRELV

>M1BUY8

REDTGKNFTNHLSTALNQAGFRT‑FEGGDDNK‑SEEDINSELSKAIQDSKMCIIVFSQNYASSSWCLDQLVSILEKK‑MKFACM‑ILPIFYHVDPSNLRKHKGSFGEALNRH‑EEKFKCEKEY‑‑‑‑KLKKWKDALSQAAD‑LAGM‑‑‑‑VLE‑‑‑‑NQHESTFIKKII

>M1BK03

TK‑TGKSFGNHLHSALSNAGIRA‑FSVDELD‑‑‑IDEKGKELQKTIQESRILIVVLSKDYTSSERCLDELVFILESK‑KLFGRF‑VLPVFYDVDPSEVRKQKGSFEQDFLMY‑EQRYRSEERRLE‑‑KVKEWKASLTEVAD‑LGGM‑‑‑‑VLQ‑‑‑‑NQCESRFIQEIV

>M1CI84

GEDTRKNFTGHLYFRLCQVGVNT‑FIDDEELR‑KGDVISIELEKAIEQSRVAIVVFSKNYASSSWCLDELVKILDCK‑ERLNQV‑VLPIFYDVDPSQVRRQTGSFDEALAKH‑KERF‑VG‑‑‑AE‑‑RMEKWKAALTEAAN‑LSGW‑‑‑DL‑RN‑‑ADGHEAKFIENII

>M1B208

GEDTRKTIIGYLYERLTRKGIIA‑FQDDKRLE‑RGDSIPEELLKAIQDSQVALIIFSGNYATSRWCLDELVKIMECT‑DENEKI‑VMPVFYGVEPSHVRNQSDSFAEAFAEH‑ESKYKDDE‑GMQ‑‑KVKEWRTALTAAAN‑LKGY‑‑‑VF‑‑‑‑‑GNG‑ESDYIECIV

>M1BA09

GLDTRNNFTSHLYKALTNRVILT‑FLDDESLE‑SGDTLWIELEKAMEESQVAVIIFSKNYATSSWCLDELVKIMKCK‑D‑NGQT‑VIPVFYDVDPSHVRYQSESFKEAFARH‑ESRYTD‑‑‑‑‑E‑‑KIEGWRTALIAAAN‑IKGH‑‑‑NI‑‑‑‑‑RDR‑EAHFIEDLV

>M0ZR07

GEDTRRNFMSHLYQGLKNRGIVT‑FLDDKRLE‑DGDSISEELMKAIEETQVAVIIFSKNYATSSWCLNELVKIMECK‑KENGHMTVIPVFCNVDASHVRYQSQSFKEAFAKH‑ESRYKDDA‑GMQ‑‑KVQRWRTALTAAAD‑LKGY‑‑‑VF‑‑‑‑‑PSRSDADCIKGIV

>M0ZQS6

GKDTRRNFTSHLYQGLKDRGIFT‑FLDDEMLE‑DGDSISEELVKAIEESQVAIIVFSKNYATSRWCLNELVKIMECK‑KENGQT‑VIPVFCYVDPSHVRNQSKSFAKAFVKH‑KSRYKYDE‑GMH‑‑KVQGWRNALTAAAD‑LKGY‑‑‑DI‑‑‑‑‑RDG‑ESEKIQQIV

>M1AVP4

GEDTRKTFTGHLYEGLKNRGIFT‑FQDDKRLE‑HGDSIPEELLKAIKESQVALVVFSENYAASRWCLNELVKIMEYM‑EENGQI‑VIPVFYNVDPSHVRHQRENFAKAFAKH‑ELKYKDGE‑RME‑‑KVQRWRTALTAATD‑LKGY‑‑‑DI‑‑‑‑‑RQG‑ESENIQQIV

>M1B034

SK‑T‑‑‑FGDHLHTALLNAGIPS‑FRPDDKE‑‑‑LD‑‑‑KKLQNSIQESRILIAIISKDYASSYRCLDELTHMIQTK‑KAFGNF‑LLPVFYDVDPSDVRKQKGSFEEPFFNF‑KKRYKT‑‑‑‑‑‑‑‑KVDQWRAALRQVAD‑LGGM‑‑‑‑VLQ‑‑‑‑NQSESRFIQEIV

>M1BUY5

AHDTGKTFTDHLHRNLFRAGFHV‑FKCEDDDE‑EIDELKLKLKKGIEQSKMSVIVLSQNYASSERCLYELVVILEQR‑RNSGHI‑VLPVFFNVDPSDVRKQKGSFG‑‑‑‑‑‑‑KENFQIESH‑‑‑‑‑KLRDWRNALKEVAD‑LGGM‑‑‑‑PLQ‑‑‑‑NQHEAKFIENIV

>M0ZJY0

GKDTRRNFTSHLYEGLDNRGIFT‑FLDNKRLE‑NGDSIPQELLKAIEESQVALIIFSKNYATSRWCLNELVKIMECK‑KENGET‑VIPIFYDVDPSHVRNQNESFAIAFAEH‑ELKYKDDE‑GMQ‑‑KVQGWRNALSAAAD‑LKGY‑‑‑DI‑‑‑‑‑RHG‑ESENIHKIV

>M0ZR05

GADTRATFTSHLYEGLKNRGIFT‑FQDDKRLE‑QGDSISEELLKAIEESQVALIIFSKNYATSRWCLNELVKIMECK‑EENGQT‑VIPIFYDVDPSNVRYQSESFAEAFAKH‑ESTYKDDE‑GMQ‑‑KVQGWRNALTATGN‑LKGY‑‑‑DI‑‑‑‑‑RGGDQSKEIEQIV

>K4BSV5

GEDVRKNFVDHLYTALQQRGIHT‑FKDDEKLE‑RGKSISPSLFKAIEESMISIIIFSQNYAASSWCLDELVKITQCM‑KLRGQI‑VLPVFYDVDPSVVRKQKANVGEFFARH‑ELDFKDD‑‑‑EE‑‑RVKRWRTAMTEAAN‑VSGW‑‑‑DL‑PN‑‑ANGHESKCIEQVV

>K4CWR7

GEDTRKNFTSHLYFRLCQVGVNT‑YIDDEELR‑KGDVISNELDKAIEQSRISIVVFSKNYASSSWCLDELVKILECR‑AKLNQV‑VLPIFYDVDPSQVRKQSGSFGEAFAKQ‑KQRL‑FG‑‑‑AE‑‑IMEKWKAALTEAAN‑LSGW‑‑‑DL‑RN‑‑ADGHESKFIESII

>K4AWK5

GEDTRKNFTSHLYQGLENRGILT‑FVDDKRLE‑DGDSISEELVKAIEESQVAVMVFSKNYATSRWCLNELVKIMECK‑KEIGHI‑VIPVFYYVDPSHVRYQSESFAEAFAKH‑ESRYKDDE‑GMQ‑‑KVQGWRNALAAAAN‑LKGY‑‑‑DI‑‑‑‑‑RDW‑ESDFIQHIV

>K4BNN9

GKDVRKTFVDHLYVALQQKGINT‑FKDDDKLE‑KGDSISPGLARAIEESRIALIIFSKNYAESSWCLDEVVKIMECK‑KVKKQI‑VIPIFYDVDPSTVRKQKSSFEEAFNKY‑EDCI‑‑‑‑‑‑‑‑‑‑KVQKWRGALEEAAN‑LSGW‑‑‑DL‑PN‑‑SNAHEAIVIKQIV

>K4DHG3

GVDIRKTFVSHLYNALVQRGINV‑FKDDERLE‑TGKSISDELLKAIEESKFAIVIFSESYASSKWCLNELAHIIKCR‑KELDLT‑LIPIFFDVNPSDVSHQTQSFAESFSKH‑EEQYKDD‑‑‑ME‑‑KIHRWRDAFAVSGE‑IKGH‑‑‑HL‑QN‑‑QD‑‑EADCIKKVV

>K4D5R5

GKDTRRNFTSHLYERLDNRGIIT‑FLDNKRLE‑NGDSLSKELVKAIEESQVAVIIFSKNYAESRWCLNELVKIMECN‑K‑DGQL‑VIPVFYDVDSSHVRNQSESFADAFTKH‑KLRYENDE‑GIQ‑‑KMQRWRTALRDAAD‑LKGY‑‑‑DI‑‑‑‑‑RDW‑ESECIGDLV

>K4B1L1

TK‑IGKSFGNHLHSALSNAGIRA‑FSVDELD‑‑‑IDEKGKELQKTIQESRILIVVLSKDYTSSERCLDELVFILESK‑KLFGRF‑VLPVFYDVDPSEVRKQKGCFEQDFLMY‑EERFKSEERRLE‑‑KVKKWKTSLTEVAD‑LGGM‑‑‑‑VLQ‑‑‑‑NQCESRFIQEIV

>K4B4A8

AKDTGITFADHLYEALAGAGFVT‑LRGGDGDE‑GGEETKLKLQKGVEESGISIIILSNDYVSSSLCLDELVMILNCS‑KRRS‑‑‑VLPIFYHVDPSDVRKQKGRIGEEFDRH‑EEA‑‑‑‑‑‑‑‑‑‑‑KVKKWKEALKQVAD‑LGGM‑‑‑‑VLQ‑‑‑‑NQ‑‑SKFIQKIL

>K4AT76

GEDTRKTFTSHLYQALKNKGILT‑FQDDKRLE‑DGDSISKELSKAIKESQVALVVFSKNYATSRWCLNELVKIMECY‑DEDGKT‑VIPVFYDVDPSHVRYQSESFAEAFAKH‑ELQFKDDE‑GMQ‑‑KVKRWRTALCEAAD‑LKGH‑‑‑DI‑‑‑‑‑RQR‑ESENIQRII

>K4DHH3

GEDTRRTFVSHLYNALEQRGIHA‑FKDDERLE‑AGQSISAELLKAIEDARFAVVVFSKSYASSRRCLEELAHIIKCK‑MELEQV‑VIPVFYDVSPSNVRHQNSPFADSFFQH‑EVEYKDD‑‑‑ME‑‑KVQRWRGAFAEAGK‑LSGY‑‑‑HL‑LN‑‑KD‑‑EAACIKKLV

>K4DHH5

GEDTRRTFVSHLYNALEQRGIHA‑FKDDERLE‑AGQSISAELLRAIEDARFAVVVFSKSYASSRWCLEELAHIIKCK‑NELEQN‑VIPVFYDVSPADVRHQNSPFADSFFQH‑EVKYKDD‑‑‑ME‑‑KVQRWRGAFAEAGN‑ISGY‑‑‑HL‑LN‑‑KD‑‑EAECVKKLV

>Q6T3R3

GEDTRKTFTGHLYEGLRNRGINT‑FQDDKRLE‑HGDSIPKELLRAIEDSQVALIIFSKNYATSRWCLNELVKIMECK‑EENGQT‑VIPIFYNVDPSHVRYQTESFGAAFAKH‑ESKYKDDE‑GMQ‑‑KVQRWRTALTAAAN‑LKGY‑‑‑DI‑‑‑‑‑RNG‑ESENIQQIV

>K4BWI8

GEDTRNNITNSLYNALYSKGIRV‑FRDSEGLT‑QGDEISTGLIEAINDSAAVIAIISPNYASSRWCLEELATIYELG‑‑‑‑‑KL‑VLPLFYGVNPSDVRRQLGPF‑DGFRDL‑ERKFS‑‑‑‑‑PE‑‑KMARWRNAMERVGG‑VSGW‑‑‑‑VYDN‑G‑‑‑DESQLIQLVV

>K4D5U0

GEDTRKTFTGHLYEGLKNRGIFT‑FQDDKRLE‑HGDSIPKELLKAIKDSQVALVVFSRNYATSRWCLNELEKIMECK‑EKNGQI‑VVPIFYDVDPSHVRYQSESFAEAFVKH‑EVRYKGDE‑GMQ‑‑KVQGWRNALTAAAD‑LKGY‑‑‑DI‑‑‑‑‑RDG‑EAEYIQQIV

>K4AUB8

GKDTRKTFVGHLNYALKQKGIHT‑FKDDERLE‑RGKTISPELVKAIEESRFAIVVFSKNYASSTWCLDELVKIMECK‑KELGQT‑VIPIFYDVDPSHVTKQSETFAKSFAIH‑EENLKDD‑‑‑VE‑‑KVLCWRDAFRQAGK‑IAGY‑‑‑DL‑PN‑‑YDGYESNCIQHVV

>K4BWI9

GEDTRHGFTGKLYNELVRNGVRT‑FIDNEDLD‑RGEEISKKLVAAIEDSAASIAVISENYAESKWCLEELAKIWDCK‑‑‑‑‑KL‑LLPVFYEVDPSNVRKQKGTF‑KHFDEH‑EILLEA‑‑‑APE‑‑KVSRWRDALTKAAN‑TSGW‑‑‑‑‑‑DS‑R‑‑WEEADLIQSLV

>K4CI42

GEDTRRTFTSHLYEGLKNRGIFT‑FQDVKRLD‑HGDSIPEELVKAIKESQVSLVVFSKNYGASRWSLNELVEIMERK‑K‑NGQT‑VIPVFYDVDPSHVRNQTESFGEAFSKH‑ESKYKDDE‑AMK‑‑KVKRWRTALTVVAN‑LKGY‑‑‑DI‑‑‑‑‑RDG‑ESEKIQLIV

>K4B1L0

GEDTRKNFTDHLYTALINAGIRT‑FRDDDEIR‑RGENIESELQKGIRESKISLIVFSKDYASSRWCLDELVNILDRR‑KKEGHT‑VLPVFYTVSPEDVQNQSGSFAEAFVNH‑EKRRKEW‑‑‑ME‑‑KMEKWRLALKEVAE‑LEGM‑‑‑CL‑AK‑‑VDGHEAKFIQKII

>K4BA75

GEDTRKSFVDHLYTTLHDKGIHA‑FRDDKELS‑RGKSISPELVKAIEKSRFAVVIFSKNYADSSWCLEELTKIVECN‑KQRGQT‑LIPVFYSVDPSVVRKQKGSYGDAFAKH‑EENLKGSE‑‑SY‑‑KIQRWRDALKDAAN‑ISGF‑‑‑DV‑QH‑‑EDGHESRCIRQIA

>K4CQG0

GEDTRRTFVSHLYKALEQSGIRI‑FKDDERLE‑RGKPIFDELLKAIEESKIAIVIFSKSYASSRWCLEELAHIIKCR‑NELELI‑VIPVFYDVTPSDVRHQNPPFADSFLQY‑‑‑‑MKDD‑‑‑ME‑‑KVQRWRAAFVEAGK‑ISGY‑‑‑HL‑LN‑‑KH‑‑EAKFNKKLV

>K4B5G6

SN‑T‑‑‑FGDHLHTALLTAGIPS‑FRPD‑‑‑‑‑‑‑D‑‑‑KKLQNAIQESRILIAIISKDYASSHRCLDELTHMIQTR‑KSLGNF‑LLPVFYDVDPSDVRKQKGTFEQPFFNF‑EKRYKT‑‑‑‑‑‑‑‑KVDQWRADLREVAD‑LGGM‑‑‑‑VLQ‑‑‑‑NQSESRFIQEIV

>K4CFH6

GEDTRRTFVSHLYNALEQRGIHA‑FKDDERLE‑AGQSIFAELLKAIEDARFAVVIFSKSYASSRWCLEELVHIIKCK‑NELEQA‑VIPVFYDVSPADVRHQNSPSVDSFFQH‑EVKYKDD‑‑‑ME‑‑KVQRWRGAFAEAGN‑IS‑‑‑‑‑‑‑‑‑‑‑‑‑D‑‑EAECVKKLV

>K4DHG1

GADVRKTFVSHLHNALIQVGINV‑FI‑DERIE‑TGTSIPHELPKAIKESKFAIVIFSKSYAWSKWCLNELAEIIKCR‑KELDQI‑VIPIFYNVDPSDVSHQTQSFAEAFSKH‑EEKYEDE‑‑‑‑‑‑‑KIQRWRGALAKSGK‑IKGH‑‑‑HL‑QN‑‑KT‑‑EADCIKEVV

>M4DA47

GEELRSGFVSHLVEALQRHGINV‑FIDKL‑‑ETIGQDLS‑NLFARIEESTIALVIFSRRYTESRWCLDELSQVVEI‑ASK‑NLLQVIPIFFKVEPVTVKHLRGAFGDKFRDR‑EWEYRC‑‑‑‑‑DKPRTNRWKEALASVSSKI‑GLTFDR‑‑K‑‑‑‑S‑NESMFVRIIV

>M1BUA6

GEDTRKNFTGHLYFRLCQVGVNT‑FIDDEELR‑KGDVISNKLDKAIEQSR‑‑‑‑‑‑‑‑‑‑‑‑‑‑‑‑‑‑‑‑‑‑‑‑‑‑‑‑ERLKQV‑VLPIFYDVDPSQVRKQTGSFGEALAKH‑KERS‑FG‑‑‑VQ‑‑RVENWKAALTEAAN‑LSGW‑‑‑DL‑RN‑‑ADGHESKFIGSII

>G7ZVL3

GEDTHKTFKSHLNSALRRLDIKT‑YIE‑DNLV‑RGDEISQPLLKAIDE‑‑‑‑‑‑‑‑‑‑‑‑‑‑‑‑‑‑‑‑‑‑‑‑ILECR‑KNKGQM‑ILPVFYEVDPFHVRHQLGSYAEAFIKH‑EQRFGST‑‑‑MN‑‑VLQKWRDALGEAAN‑HSG‑‑‑‑‑‑‑‑‑‑‑‑‑‑TEAELVEEIA

>M4FAM9

GDQLRYGFVSHLIDAFERYGIMF‑FIDKH‑‑EQRGKDLT‑NLFVRMKESKIALVIFSSRYAESSWCMDELVNIKKR‑AEK‑GKLEVIPIFYKVRAKDVRAQAGKFGDKFWAL‑AKVS‑‑‑‑‑‑‑SGDQIKKWKDALECISNKM‑GLSLRD‑‑K‑‑‑‑S‑SEADFIKGIV

>C6TIX3

‑EETRHSFTGTLYHALAR‑‑FKT‑YMENGKLR‑RGDKIATAILTAMEASRISIVVFS‑‑FASSTCCLDQLVHIHRCNTKN‑‑QL‑ILPIFYDVD‑SDVRDQLNTFGQAMLQH‑QHRFGKS‑‑‑‑D‑‑KVQ‑WSSVLSHVAN‑LTAFCFSST‑‑‑‑G‑D‑QEYQFVEEIV

>M4DHQ0

GADTRKNFVSFLYRDLVAKEIRT‑FKDDKELE‑RG‑‑‑‑‑‑‑‑‑‑‑‑‑‑‑‑‑‑‑‑‑‑‑‑‑‑‑‑‑‑‑‑‑QLVKILKLE‑KQ‑GLIKVLPIFYDVDPCDVRRQTGAVKKHFEKH‑KKR‑‑‑L‑‑SRE‑‑KVKSWRDALNYLAE‑LSGE‑‑‑CS‑QNW‑‑E‑DDSKLVERIT

>V4MJX9

GEVVRKSFLSHLLKELDRKSI‑NAFID‑DGIE‑RSRPIGPELLSAIRESRISIVVFSKSYASST‑C‑‑‑‑‑‑‑‑‑‑‑‑‑‑‑‑‑‑‑‑‑‑‑‑‑‑VDPSEVRKHTGEFGKVFKETCDGK‑‑‑T‑‑‑EDQ‑K‑QRWMQALVDVAN‑MAGE‑‑‑DLRN‑W‑‑C‑NEASMIEKIA

>D7KPJ7

R‑DTSHNFTDPLYEALVKKELRV‑WNDD‑‑LE‑RGDELRPSLVEAIEDSAASVIILSTNYANSSWCLDELALLCDLR‑SSLKRP‑MIPIFYGVNPSDVRKQSGHF‑EDFNDG‑EDTAM‑‑‑‑‑EE‑‑SYEKYPWICLHVGTLIHLL‑‑‑‑ICKSWKKNEKVDELIGLVV

>B9HZC5

GEDTRKNFTDHLYTALVQAGIHT‑LRDDDEIG‑RGENIN‑‑‑‑‑‑‑‑‑‑‑‑‑‑‑‑‑‑‑‑‑‑‑‑‑‑‑‑‑‑‑‑‑‑‑‑‑‑‑‑NTDCI‑ILPVFYDVDPFEVRNQTGSFAAAFVDH‑DKRFKKE‑‑‑ME‑‑QVNGWRIAL‑‑‑‑‑‑‑‑‑‑‑‑‑‑‑‑‑‑‑‑‑‑‑YEAQLVQS‑‑

>G7JKN4

GEDIRHGFFGHLVIAFPRKQINA‑FVDE‑KLK‑RGDDMSHSLVEAIEGSPISL‑‑‑‑‑‑‑‑‑‑‑‑‑‑‑‑‑‑‑‑‑‑CK‑EKYGQI‑VIPVFYGVDPTNVRHQKKSYENAFAEL‑EKRCNSS‑‑‑‑‑‑‑KVQIWRHALNTSAN‑LSGI‑‑‑KS‑‑‑‑‑‑‑‑NDAELLEEII

>G7KJR8

GSDTRYGFTENLYRALCHKGIHT‑FIDDRELQ‑GGDEITPSLFKAIEESRIFIPVLSINY‑‑‑‑‑‑‑‑‑‑‑‑‑‑‑‑‑‑‑ENRRL‑VLPIFYDVEPSHVRHHKGSYGKALDDH‑IKKFQNNKDNME‑‑RLQKWKMALTQTSN‑FSGH‑‑‑HF‑NP‑G‑NGYEYKYIKKIV

>O65506 (0‑318)

SVDVPKSFLSRIRKELRRKGF‑EPLID‑NETE‑RCVSIGPELRNAISVSRIVIVVLSRNYALSPWCLDELVEIMKCK‑EELGQR‑VVTIFYNLDPIDVLKQTGDFGDNFRKTCERK‑‑‑N‑‑‑KED‑I‑DRWIKALEQVAT‑IDGY‑‑‑RSRD‑W‑‑D‑DEKAMVKKIA

>O65506 (319‑1607)

CEDTQYSFASHLSMDFRRKGISA‑FV‑‑‑‑‑‑‑‑‑YE‑TL‑‑‑DVIERVSASVLVFSKSCVSSTSCLDMLVRVFQCR‑RKTGQL‑VVPVYYGISSSDVVVQEHKSV‑‑‑‑‑‑‑‑‑‑‑‑‑‑‑‑‑‑D‑‑RIREWSSALQELRE‑LPGH‑‑‑HN‑E‑‑‑‑E‑SESELVEEIV

>M4FEN8 (0‑354)

EEEISRGFFGYFKKEFERKGI‑KLFID‑‑‑‑‑‑RRESFGPGLIEAIRRSRIAIVILSKHYASSTLRLDELVEIMKCR‑E‑‑‑‑‑‑‑‑‑‑‑‑ELGAADVKKQSGYFGSVFEKACVGR‑‑‑S‑‑‑VED‑V‑EKWKRALNELSF‑IFGY‑‑‑LSGN‑W‑‑K‑SEDDMMEEVA

>M4FEN8 (355‑1330)

GVDVRKSFLRHMLMVLRNKGI‑TLFTD‑IEIE‑TGTSIAHELKEAIHRSRISIILISNKYVSRSWEMEEIIE‑‑‑‑‑‑‑KVANN‑LSDLLYEDVPS‑‑‑‑‑‑GDFDGIWGPSGIGK‑‑‑T‑‑‑TTA‑R‑ALYRKLSSNFTH‑TAFM‑‑‑ESIK‑A‑‑K‑NEAEMIENVA

>F4JNL1 (0‑278)

GKAQRKTLVSFIKSKLEESEINV‑FMDEY‑‑EIRGRPIT‑TLFERIRESSIALVIFSDKYPESRWCLDELVEIKKQ‑MET‑GSIVPFPIFYKVKAESVKNQTGHFRNVLLKT‑ERKILE‑‑‑‑‑TEDMI‑‑WRQALVSVGGRM‑GFSYNH‑‑‑‑‑‑‑K‑CDNDFVNDIV

>F4JNL1 (279‑635)

EKHLGKFLVSSLKEELESNQILV‑YV‑ED‑‑ETK‑‑‑‑‑‑‑‑‑SRIKESGVAVVFFSKKYPNSEKCLDELVEIKKL‑MDA‑GKIDPLPVFYSLKDEPVKNLKGYFLNRLLKI‑ERKILD‑‑‑‑‑TEAKI‑‑WRDALSSIASRP‑GLSYEL‑‑‑‑‑‑‑S‑TDDVFVSDIV

>F4JT82

WEDTRHSFVSHLSAEFQRKGVSV‑FAEDD‑‑‑‑‑‑AE‑SD‑‑‑AAIAKARVSVVIFSENFASSKGCLNEFLKVSKCR‑RSKGLV‑VVPVFYGLTNSIVKKHCLELKKMY‑‑‑‑‑‑P‑‑‑‑‑‑‑DD‑‑KVDEWRNALWDIAD‑LGGH‑‑‑VS‑H‑‑‑‑K‑SDSELVEKIV

>V4LWJ7 (0‑268)

GKGQRDKLVSFLKKQLERSDINF‑FMDEN‑‑EVRGMPLT‑TLFERIRESSVALVFFSDKYPESCWCLDELVEIKKQ‑MEK‑GSLVPFPIFYKVKAETIKRQTGCFGNSLLRT‑ERKILE‑‑‑‑‑TEAVI‑‑WRQALVSVGGRM‑GFSYKH‑‑‑‑‑‑‑S‑SDEAFVSDLV

>V4LWJ7 (269‑600)

EKQLRENLVSFLKTELESNRISV‑CV‑ED‑‑EMK‑‑‑‑‑‑‑‑‑KRIKESKVAIIIFSAKYPESQHCLDELVEIKKL‑MDT‑GEIDPFPIFYKLKAESVKVIKGWFRNRLLKI‑ERKILD‑‑‑‑‑TEARI‑‑WREALASLVSRP‑GLSYQH‑‑‑‑‑‑‑S‑SDSLFVTDVV

>Q56XU3 (0‑206)

GEELRKSFLGFLLKAMRDAKINV‑FTDEI‑‑EVRGRDIQ‑NLLSRIEESRVAIAILSKRYTESSWCLDELVKMKER‑IDQ‑DELVVIPIFYRLDATNCKRLEGPFGDNFRNL‑ERDYRS‑‑‑‑‑EPERIKKWKEALISIPQKI‑GLTSAG‑‑H‑‑‑‑R‑D‑SELVDSIV

>Q56XU3 (207‑419)

NDELGDNFIKHLVWALRDSGINV‑FKDSF‑‑KLIGGQKQ‑EVFMSIENSNIALAIFSKRYSESYRCLNELVKMEEL‑AKE‑GKLVVIPVFYSVKTNEVRRLEGEFGIHFRNT‑KER‑‑‑‑‑‑‑‑EPMMVESWEKSL‑‑‑‑KRI‑GLSLEA‑‑H‑‑‑‑M‑NEFALVGAIV

>D7MKB0 (0‑266)

GRDLRYGFVSHLEKILKDHKIEV‑FVDSG‑‑EDRGEHLE‑NLLTRIEESRIALAIFSENYTESEWCLRELAKIKDC‑VDQ‑KRLVAIPIYYKVDRYSVKYLTEKFGDAFREL‑AKD‑‑‑‑‑‑‑‑DL‑RKKEWKQALQSIVNRI‑GFMVDE‑‑K‑‑‑‑S‑NENEILNEIV

>D7MKB0 (267‑495)

GDQLRNNFVGYLRHALRISKINV‑FIDNE‑‑EQRGEDLN‑TLFKRIEESQIAIVVFSSRYTESKWCLDELVKIKER‑VDQ‑GLL‑‑‑‑‑‑‑‑‑‑‑‑‑‑‑‑‑KGEFGDHFRDI‑EYMYKS‑‑‑‑‑DEPMIKQWKEAIVFVSHKF‑ALTLDE‑‑K‑‑‑‑S‑SEIDFVETIV

>G7J7A7 (0‑730)

GEDTRLGFTDHLYAALVRKSIIT‑FRDDEELA‑RGEVISQKLLLAIEESLSAVLIISKNYANSAWCLDELVKILESK‑RLLGQ‑‑VFPVFYGVDPSDVRNQRGSFAEAFKKH‑EEKFSES‑‑‑KE‑‑KVQKWRDALREVAN‑LSGW‑‑‑DS‑‑‑‑‑KD‑HETKLIEEVI

>G7J7A7 (731‑1664)

GEDTRLGFTDHLYASLVRKSIIT‑FRDDEELA‑RGEVISQKLLHAIEESLSAIVIISKNYADSAWCLDELVKILESK‑RLLGQ‑‑VFPIFYGVDPSDVRNQRGSFAEAFKKH‑EEKFSES‑‑‑KE‑‑KVQRWRDALREVAN‑FSGW‑‑‑DS‑‑‑‑‑KD‑HETKLIEEVI

>D7MRD9 (0‑469)

GADVRKTFLSHFLKELDLKSI‑KPFKD‑SEIE‑RSHSIAPELIQAIRGSRIAVVVFSENYATSKWCLDELVEILKCK‑EELGQI‑VIPIFYDLDPFHVRKQLGKFGEAFKNTCLNK‑‑‑T‑‑‑KNE‑I‑QLWRQALNDVAN‑LLGY‑‑‑HSHT‑C‑‑N‑NEPKMIEDIV

>D7MRD9 (470‑1750)

GADVRKTFLSHFLKELDLKSI‑KPFKD‑SEIE‑RSHSIAPELIQAIRGSRIAVVVFSENYATSKWCLDELVEILKCK‑EELGQI‑VIPIFYALDPFHVRKQLGKFGEAFKKTCLNK‑‑‑T‑‑‑EDE‑R‑QLWRQALTDVAN‑LLGY‑‑‑HSHT‑C‑‑N‑SEAKMIEDIV

>G7JSC4 (0‑355)

‑QDAAS‑FATGIYTALRKSRFHV‑FWDDE‑‑‑‑KGD‑‑GPSILNVIEDCKVAVIVFSRNYVNSRSCLQEFEKITECL‑TS‑G‑I‑VLPVLYGLNSGTVE‑‑‑‑‑‑‑ETFHDF‑D‑R‑KET‑‑SEEKDKFMSWVAAVT‑KAT‑‑TGV‑‑IDF‑‑‑‑‑AD‑YGREYVVDVV

>G7JSC4 (356‑1864)

GEDSRAKFMSHIFSSLQNAGIHT‑FRDDDQIQ‑RGDQISVSLLRAIGQSRISIIILSTNYANSRWCMLELVKIMEIG‑RTRGLV‑VLPVFYEVDPSEVRHQEGQFGKSFEDL‑‑‑‑‑‑‑‑‑‑SVD‑‑EKSNWKRDLIDIGG‑IAGF‑‑‑VL‑‑‑‑‑KDSNESADIKNIV

>U7DX85

GKDTRNNFTSHLYYNLAQRGIDV‑YMDDREFE‑RGKTIEPALWKPFEESRFSVIIFSRDYASSPWCLDELVKIVQCM‑KEMGQT‑VLPVFYDVDPSEVTDRKRKYEEAFGEH‑EQNFKEN‑‑‑LE‑‑KVRNWKDCLSTVAN‑LSGW‑‑‑DV‑R‑‑‑‑NRNESESIKIIV

>F4J910

GEDIQESLMVHVDKEFQRKGI‑TPFND‑NEIK‑RGESISPELVLAIRGSRIALILLSRNYASSSWCLDELAEIIKCR‑EEFGQT‑VMVVFYKVDPSDIKKLTGDFGSVFRKTCAGK‑‑‑T‑‑‑NED‑T‑RRWIQALAKVAT‑LAGY‑‑‑VSNN‑W‑‑D‑NEAVMIEKIA

>G7JSA9 (0‑542)

‑EDSRS‑FVLSIYTALSKPGVVV‑FWEDQ‑‑‑‑WGDS‑QSSALNVIEDCEIAVIIFSKNYTKSRWCLQELEKITQCR‑TD‑G‑I‑FLSVFYDVYDLWVRR‑‑D‑FG‑‑‑EDF‑D‑REKET‑‑SEDEDKFMTWVAAVTNEAS‑‑KEL‑‑YSLRH‑‑‑SH‑HESELIKIV‑

>G7JSA9 (543‑1714)

GKDCCTKFISHLYTSLQNAGIYT‑FRDDDEIQ‑RGDRISMSLLKAIGRSRISIVVLSTTYANSRWCMLELVKIMEIG‑RTMDLI‑VVPVFYEVDPSEVRHQKGKFGKAFEEL‑‑‑‑‑‑‑‑‑‑SVD‑‑EKSDWRRDLSDIGG‑IAGI‑‑‑VL‑‑‑‑‑IDSNESEDIKNIV

>U7E2T9

GKETRNNFSSHLYSNLKQRGIDV‑YMDDRELE‑RGKAIEPALWKAIEESRISVVIFSRDYASSPWCLDELVKIVQCM‑KEMGHT‑VLPVFYDVDPSDVAERKRKYEKAFVEH‑EQNFKEN‑‑‑ME‑‑KVRNWKDCLSTVAN‑LSGW‑‑‑DV‑R‑‑‑‑HRNESESIRIIA

>Q9FKE3 (0‑265)

GS‑‑RMGFIYHLIMALEKKNINV‑FVGFN‑‑GCICEPVE‑RLSNRIE‑SIIVLVIFTSRYTESKWCLMKLVDINKC‑AEK‑DHLVAIPIFYKLDPSTVRGLSGQFGDAFRDL‑RESM‑‑‑‑‑‑‑‑‑‑‑‑EKWKEALKSISDRP‑GIRVDK‑‑S‑‑‑‑S‑PKAKRIEIV‑

>Q9FKE3 (266‑546)

GDQLRNNFVGYLVDALRRSEINV‑FIDNQ‑‑EQRGEDLN‑TLFKRIEDSGIAIVVFSSRYTESKWCLEELVKIKER‑VHQ‑GLLKVLPIFYKVTPTNVKRPKGEFGDHFRDK‑EYMYES‑‑‑‑‑DEPMIKRWKEAIVFISHRF‑ALTLDE‑‑KSWTCS‑SEIDFVETIV

>F4JT78 (0‑382)

KVDVRRSFLAHLLKELDRRLI‑NTFTD‑HGME‑RNLPIDAELLSAIAESRISIVIFSKNYASSTWCLDELVEIHTCY‑KELAQI‑VVPVFFNVHPSQVKKQTGEFGKVFGKTCKGK‑‑‑P‑‑‑ENR‑K‑LRWMQALAAVAN‑IAGY‑‑‑DLQN‑W‑‑P‑DEAVMIEMVA

>F4JT78 (383‑1309)

NLDVETSFIEAISKELHKQGFIP‑LTN‑‑‑LG‑R‑EL‑DE‑‑‑EMLYGSRVGIMILSSSYVSSRQSLDHLVAVMEHW‑KTTDLV‑IIPIYFKVRLSDICGLKGRFEAAFLQL‑H‑M‑‑‑S‑‑‑ED‑‑RVQKWKAAMSEIVS‑IGGH‑‑‑EW‑K‑‑‑‑‑‑SQFILAEEVV

>M4EA32

GEDVRVRFRSHFLKELNRKLI‑TPFKD‑DEIV‑KGRSIGHELINAIRGSRISVVAFSDNYASSSWCLDELVEIIKCR‑EELGQI‑LIPIFYDVDPSHVKKQTERFGVIFEKTCQGR‑‑‑K‑‑‑EEE‑K‑LRWRRALTHAAT‑IAGE‑‑‑DSRN‑W‑‑S‑DEAKMIEKIV

>D7MIU3

GPDVRRGFLSHLHNHFTSKGI‑TTFKD‑QEIE‑RGQTIGPELVQAIRESRISVVVLSKSYGSSSWCLDELVEILRCK‑EDQGQI‑VMTIFYEIDTSDVRKQSGDFGRDFKRTCEGK‑‑‑T‑‑‑EEV‑K‑QRWIQALAHVAT‑IAGE‑‑‑HLLN‑W‑‑D‑NEAAMVQKFA

>G7JTC9 (0‑340)

‑QDRDH‑FIWHLNTVLSKAGTAL‑FGVEE‑‑‑‑RQ‑‑‑‑EELDAIVGYCKLAIVVFSSNYNKSISCVQELEKITECR‑‑S‑D‑V‑VFPVFFGVYPERSEG‑‑G‑FDNAFHDF‑D‑R‑‑‑I‑‑KDK‑DKFLSWVVGVT‑KAT‑‑EGP‑‑SDLYR‑‑‑EH‑YVQDYIRDIG

>G7JTC9 (341‑1673)

GEDSRAKFMSHLFSSLQNEGIHA‑FKDDNEIQ‑RGDQISISLLRAIGQSRISIIVLSTNYANSRWCMLELEKIMEIG‑RTKGLI‑VVPVFYEVAPSEVRDQKGRFGKAFKKL‑‑‑‑‑‑‑‑‑‑SMD‑‑EKSNWRRDLFDIGG‑IAGF‑‑‑VL‑‑‑‑‑LGSNESADIKNIV

>G7JSB5 (0‑365)

‑HDKGY‑FLSSLEEALLEAGINV‑FGDIK‑‑‑‑RQ‑‑‑‑VSVLNVIQDCKVAVVLFSKNYTNSSSCIQELEKITQCR‑‑S‑D‑V‑VLPVFYGVGP‑‑‑HG‑‑D‑FGDTFHDF‑D‑R‑EEI‑‑KEE‑DKLMTWVAAIT‑KAN‑‑KGS‑‑RDLYR‑‑‑EH‑SITDYIKDIV

>G7JSB5 (366‑1684)

GEDNRAKFMSHLYSSLQNAGIYV‑FRDDDEIQ‑RGDHISISLLRAIEQSRTCIVVLSTNYANSRWCMLELEKIMEIG‑RNRGLV‑VVPVFYEVAPSEVRHQEGQFGKSFDDL‑‑‑‑‑‑‑‑‑‑SVD‑‑EKSNWKRELFDIGG‑IAGF‑‑‑VL‑‑‑‑‑IDSNESADIKNIV

>G7JSB4 (0‑1121)

GEDSRAKFISHLHSSLENAGIHV‑FKDDFKIQ‑RGDQISISLFRAIGQSRICIVVLSKNYANSRWCMLELENIMEIG‑RNRGLV‑VVPVFYEVDPSEVRHQKGHFGKGFDDL‑‑‑‑‑‑‑‑‑‑SVD‑‑EKSNWRRELFDICG‑ISGF‑‑‑VL‑‑‑‑‑INSNESADVNSIV

>G7JSB4 (1122‑2026)

GEDVRSNFKDLMYKSLIQMGIDT‑FLDDDNLN‑KGDEISPHLFSAVRESKVVVVILSKNYTHSRWCLQELEKITQCRTKD‑G‑V‑VLPVFYDVHPSRIL‑QE‑DYGEAFHDF‑D‑RMKET‑‑SEDEDKM‑SWNEASKYAA‑‑LAGP‑‑‑N‑‑Q‑‑‑‑‑‑NRGEHITHVV

>K4B1L4

GQEISMNFINILHTALTDVGIQT‑FKRHSESR‑NGKTVGSELQKAVKESRISIVVFTEDYGYSRRCLDELVSILERK‑QIAGHM‑ILPVFYRVDPSHVRKQRGSFAKAFHNY‑EEQIMVVERRNE‑‑KIKIWRSSLTDVAN‑MAGI‑‑‑‑VLE‑‑‑‑DG‑ELKFIQEIV

>G7JSB9 (0‑379)

‑QDKHS‑FVFSIYNALRKAGVDV‑FWENE‑‑‑‑RGD‑‑EPSVLNVIRDCKVFVIVFSRDYFNSRSCLHEFKKITECR‑KD‑D‑M‑VLPVFYGVDLGSWER‑‑G‑FGETLHDC‑D‑K‑KKT‑‑KEE‑DKFMTWVASIS‑KAT‑‑IGQ‑‑SDL‑‑‑‑‑ED‑NSSIYIDDVV

>G7JSB9 (380‑1831)

GEDSRAKFISHLYSSLQNAGIYV‑FKDDDEIQ‑RGDQISISLLRAIGHSRIFIVVLSTNYANSRWCMLELEKIMEIG‑RTGGLV‑VVPVFYEVDPSEVRRREGQFGKAFEKL‑‑‑‑‑‑‑‑‑‑SVD‑‑EKSNWKRALFDIGS‑IAGF‑‑‑VL‑‑‑‑‑IDSNESADIKNIV

>M4D5A8

CEETQYTFASHLSVDFRRKRIAA‑FV‑‑‑‑‑‑‑‑‑CL‑NP‑‑‑DVAEGASASVVVFSKSYSSSASCLDKLVTVLRCR‑RNTGQMVVVPVFYGISPSDVAVRVHGSA‑‑‑‑‑‑‑‑‑‑‑‑‑‑‑‑‑‑D‑‑RIREWSNALRELRE‑LPSH‑‑‑QC‑E‑‑‑‑E‑DEGQVVEEIV

>D7KPJ9

GEDTREIFAGPLYKAL‑KEKVRV‑FLDNDGME‑RGDEIGSSLQAGMEDSAASVIVLSRNYANSRWCLNELAMLCKLK‑SSLDRR‑MLPIFYKVDPSHVRKQSDHI‑ADFKRH‑EERFD‑‑‑‑‑KE‑‑KVQEWRDAMKLVGN‑LAGY‑‑‑‑VCVE‑‑‑‑SNEDEMIELVV

>U7E0X1

KKKNRSHWNKKKVVRALERGIDV‑YMDDRELE‑RGKAIEPALWKAIEESRISVVIFSRDYASSPWCLDELVKIVQCM‑KEMGHT‑VLPVFYDVDPSDVAERKRKYEKAFVEH‑EQNFKEN‑‑‑ME‑‑KVRNWKDCLSTVAN‑LSGW‑‑‑DV‑R‑‑‑‑HRNESESIRIIA

>F4HR54

GFDTRTNFCERLYIALNEKQVRV‑FRDNEGME‑KGDKIDPSLFEAIEDSAASVIILSTNYANSSWCLDELALLCDLR‑SSLKRP‑MIPIFYGVNPEDVRKQSGEF‑KDFEEK‑AKSFD‑‑‑‑‑EE‑‑TIQRWKRAMNLVGN‑IPGY‑‑‑‑VCTA‑‑INEKVDDMIDLVV

>G7JSD3 (0‑465)

GDDGSAKFVSHLHSSLQNAGISV‑FR‑GDEIQ‑QGDDISISLLRAIRHSRISIVVLSINYANSRWCMFELEKIMEIG‑RTGGLV‑VVPVLYEVDPSEVRHQEGQFGKALEDL‑‑‑‑‑‑‑‑‑‑SVD‑‑EKSNWRRDLIDIGG‑KDGF‑‑‑IV‑‑‑‑‑TDSNESADIKNIV

>G7JSD3 (466‑1898)

GEDNRPRFISHLHSSLHSAGIYA‑FKDDDGIQ‑RGDQISVSLGKAIEQSRISIVVLSTNYANSRWCMLELEKIMEVG‑RMNGRV‑VVPVFYDVDPSEVRHQKGRFGKAFEEL‑‑‑‑‑‑‑‑‑‑SVD‑‑EYSNWRRQLFDIGG‑IAGF‑‑‑VL‑‑‑‑‑VGSNESAAVKNIV

>G7JDC6

GEDCRAKFISHLYISLQNSGLYV‑FKDDDGIQ‑RGDQISVALIQAVGQSKISIVVLSKNFANSKWCMTELERIVEIS‑RTKGMV‑LVPVFYEVDPSEVRHQTGEFGKAFECL‑‑‑‑‑‑‑‑‑‑SVD‑‑EKRNWKAALHEVGS‑IAGV‑‑‑VI‑‑‑‑‑LKSDESEDIKKIV

>M4DB38

EKQLKMNLVSSLKTEFESNEISV‑YI‑ED‑‑ETK‑‑‑‑‑‑‑‑‑ERIKESKVAIVVFSDKYPESPQCLDELVEIKKL‑MDA‑GEITPFPIFYKLKAQSVKQLKGCFRNRLLKI‑EHKILD‑‑‑‑‑TEARI‑‑WRQAISSISSRP‑GLSNEN‑‑‑‑‑‑‑S‑SDPVFFTDVV

>K7KXJ4

GLDTRNNFAALLLQALHRNGIDA‑FNDNVHVM‑KGEFIEYELYKAIDGSRNFIVVFSKNYASSTWCLRELARICKN‑IETSRR‑‑ILPIFYVVDPLKVQKQSGCYEKAFLDH‑EERFRGAKER‑E‑‑QVWRWRKALKQVSH‑LPCL‑‑‑HI‑Q‑‑‑‑NELQQAEIEEIL

>K4D5R6 (0‑675)

GGDTRKNFTSHLYKALTNRGISA‑FLDEETLE‑HGDSISEQIVKVIEESQVAVVIFSKNYAKSKWCLNELVKIMECK‑K‑NGQL‑VIPVFYDVDPSEVRYIRGTFAEAFAKH‑NIRYKDEG‑GIH‑‑KVIKWMVAASNASY‑LEGC‑‑‑DI‑‑‑‑‑RER‑ESDCILDLV

>K4D5R6 (676‑2871)

GEDTRRTFMSHLYQGLKNRGIFT‑FQGDERLE‑LGDSI‑QELLKGIEESQVALIVFSKNYATSVWCLNELVKIMECK‑E‑NGQT‑VIPVFYDVDPSHVRNQRESLEEAFAKH‑ESMYKDDE‑GMQ‑‑KVKRWRNALTAAAD‑LKGY‑‑‑DI‑‑‑‑‑RDG‑ESENIQQIV

>D7M939 (0‑224)

GKDERYGFLTHLKQKLIDGNVNV‑FTDD‑‑‑NVTGQPLQ‑NLFGHIRKSRIVIVIFSKNYAESDWCLDELVEIKKC‑FET‑ELKAVIPIFHKVKVSSVKKQSGKFGEKFLAL‑QKKIKR‑‑‑‑‑INSRIKRWKKALKIVTEIA‑GL‑‑‑D‑‑K‑‑‑‑N‑SELAFVEKVV

>D7M939 (225‑506)

GKAQRKTLVSFIKSKLEESEINV‑FMDEY‑‑EIRGRPIT‑TLFERIRESSIALIIFSDKYPESRWCLDELVEIKKQ‑MDT‑GSIVPFPIFYKVKAESVKYQTGHFRNVLLKT‑ERKILE‑‑‑‑‑TEDMI‑‑WRQALVSVGGRM‑GFSYNH‑‑‑‑‑‑‑K‑CDNDFVNDIV

>D7M939 (507‑879)

EKHLGKFLVNSLKEELESNQILV‑YA‑ED‑‑ETK‑‑‑‑‑‑‑‑‑SRIKESGVAVVVFSNKYPKSEKCLDELVEIKKL‑MDA‑GKIDPLPVFYSLKVEPVKKLKGCFLNRLLKI‑ERKILD‑‑‑‑‑TEAKI‑‑WRDALSSIASRP‑GLSYEL‑‑‑‑‑‑‑S‑TDDVFVSDIV

>G7L5T5 (0‑352)

DKDTSESLASYLYTALTVAGIVV‑YKDEDKLL‑NHDQITSSVLHAIAGSRLSIIVFSKLYAVSTCCRQELEKIMECR‑RTTCQI‑VVPVFYDADPSGVFHQEDLLGEASKYL‑‑‑‑‑‑‑‑‑‑‑‑‑‑‑KILKKDKLIHEVCN‑ISGF‑‑‑AV‑‑‑‑‑‑HSNESEDIMKIV

>G7L5T5 (353‑853)

GDDTHAKFISHLYTALENAGIYV‑FRGDDEIQ‑RGDQVSVSLLQAIGQSRISIIVLSRNYANSRWCMLELENIMGNS‑RTQGMV‑VVPVFYKIDPTEVRNQSGRFGEDFESL‑‑‑‑‑‑‑‑‑‑SVD‑‑TFSNWRRALAEVRG‑TTGV‑‑‑VI‑‑‑‑‑INSNESEDITKIV

>G7L5T5 (854‑2084)

GNDTRAKFISHLYTALENAGIYV‑FRDDDEIQ‑RGDQISASLLQAIEQSKISIVVLSRSYADSRWCMLELENIMGNS‑RTQGMV‑VVPVFYEIDPSEVRNQSGKFGEDFESL‑‑‑‑‑‑‑‑‑‑SVD‑‑TLSNWKTALAEVGG‑TAGV‑‑‑VI‑‑‑‑‑INSNESEDIRKIV

>K7KXM9 (0‑301)

GLDTLYGFTGNLYNALYDRGIYT‑FIDDQERS‑RGDEIAPALSKAIQESRIAITVLSENYAFSSFRLNELVTILDCK‑S‑EGLL‑VIPVFYNVDPSDVRHQKGSYGEAMTYH‑QKRF‑‑‑KANKE‑‑KLQKWRMALHQVAD‑LSGY‑‑‑HF‑KD‑G‑DSYEYKFIGSIV

>K7KXM9 (302‑599)

GEDTRYSFTGNLCRALHDSGIHT‑FVDDEELQ‑RGDEITSELEKEIEDSRFFIIVLSQNYASSSFCLNVLAYILECV‑KRKRLL‑VLPIFYKVDPSNIGYHRGSFGEALANH‑EMKFKAKEHNME‑‑KLEKWKMALHETAN‑FSGY‑‑‑HF‑KQ‑G‑DGYEYEFITRIV

>K7KXM9 (600‑1238)

GSDTLHGFTGYLYEALHDSGIHT‑FIDED‑LK‑RGEEITPAIVKAIEESRIAIIVLSINYASSSSCLDELATILDCL‑KRKRLL‑VLPVFYNVDHSQVRLQEGSYGEALVKH‑EESL‑‑‑KHSME‑‑KLEKWKMALHQVAN‑LSDI‑‑‑KI‑KH‑G‑ARYEYDFIGEIV

>K7KXP0

GEDTRHGFTGHLYKALCDKGIRA‑FMEEVDLK‑RGEEITRTLEEAIKGSRIAITVLSENYASSSFCLNVLAYILECV‑KRKRLL‑VLPIFYKVDPSNIRYHRGSFGEALANH‑EMKFKAKEHNME‑‑KLEKWKMALHETAN‑FSGY‑‑‑HF‑KQ‑G‑DGYEYEFITRIV

>K7KXP2 (0‑381)

GEDTLNNFTVFLFDALSQNGIDA‑FKDDTHLQ‑KGESIAPEVLQAIEESLLFLVVFSKNYASSTRCLRELAHICNCTDEASPS‑‑VLPVFYDVDPSEVRKQSGYYGIAFAEH‑ERRFREDIEKME‑‑EVLRWREALTQVAN‑ISGW‑‑‑DI‑R‑‑‑‑NKSHPAMIKEIV

>K7KXP2 (382‑743)

GEDTRYSFTGNLCRALRDSGIHT‑FVDDDELQ‑RGDEITSELEKEIEDSRFFIIVLSQNYASSSFCLNVLAYILECV‑KRKRLL‑VLPIFYKVDPSSIRFHGGSFGEALANH‑EMKFKAKEHNME‑‑KLEKWKMALHETAN‑FSGY‑‑‑HF‑KQ‑G‑DGYEYEFITRIV

>K7KXP2 (744‑1382)

GSDTLHGFTGYLYKALHDRGIHT‑FIDED‑LK‑RGEEITPEIVKAIEESRIAIIVLSINYASSSFCLDELATILDCL‑ERKRLL‑VLPVFYNVDHYQV‑‑LGGSYVEALVKH‑GKSL‑‑‑KHSME‑‑KLEKWEMALYEVAD‑LSDF‑‑‑KI‑KH‑G‑ARYEYDFIGEIV

>105922849

GKDTRNNFTSHLYSNLAQRGIDV‑YMDDSELE‑RGKTIETALWKAVEESRFSVIIFSRDYASSPWCLDELVKIVQCM‑KEMGQT‑VLPVFYDVDPSEVAKRKGQYEKAFVEH‑EQNFKEN‑‑‑LE‑‑KVRNWKDCLSTVAN‑LSGW‑‑‑DI‑R‑‑‑‑NRNESESIKIIV

>947078674

G‑GTSNPFVDPLCRALRDKGISI‑FRSED‑‑‑‑‑‑GETRPA‑IEEIEKSKMVIVVFCQNYAFSTESLDELVKIRE‑V‑DNRRKQ‑VWTIFYIVEPSDVRKQRNSYKDAMNGH‑EMTYGKS‑‑‑‑E‑‑KVKAWREALTRVCD‑LSGI‑‑‑H‑CK‑‑‑‑D‑FEAEL‑QKIV

>947059181

GSDTRYGFTGHLYKALCDRKVRT‑FIDDEELQ‑RGEQITPSLLKAIEESRIFIPVFSKNYASSTFCLDELVHIFACV‑KEKSRL‑VLPVFYEVDPSHVRHQRGSYKEALNSH‑KKRFNDDQE‑‑‑‑‑KLQKWRNALSQAAN‑LSGY‑‑‑HF‑KQ‑GE‑EYEYDFIAKIV

>947107202

GEDTRNNFTAFLFDSLSQNGIHA‑FKDDTHFP‑KGESIAPELLQAIEESRLFLVVFSKNYASSTWCLRELAHICNCTIEASPS‑‑VLPIFYDVDPSEVRKQSGYFGIDFAEH‑EERFREDKEEME‑‑EVQRWREALTQVAH‑LSGW‑‑‑DI‑R‑‑‑‑NKSQPAMIKEIV

>731431476

GEDTRTIFTDHLFVNLGGRGINT‑FRDDQ‑LE‑RGEEIKSELLKTIEESRISVVVFSRNYAHSKWCLDELAKIMECR‑EEMEQI‑VLPVFYHVDPSDVRKQTGSFGEAFSIH‑ERNVDE‑‑‑‑‑K‑‑KVQRWRVFLTEASN‑LSGF‑‑‑HV‑N‑‑‑‑DGYESMHIEEIT

>297742842

GEDTRYDFTDHLYNALVGKGIIT‑FRDEK‑LK‑RGEKIAPKLLNAIEKSRSSIVVFSKTYADSRWCLDELAKIIECS‑RKYRQI‑VFPIFYHVDPSDVRKQTGRFGEAFTKY‑EEN‑‑‑‑‑‑‑KN‑‑KVQSWREALTEAGN‑LSGW‑‑‑HV‑‑‑‑‑NEGYESEHIKKIT

>298204577

GEDTRKNFTDHLHEALRRNGIHA‑FIDDQ‑LR‑RGEQISSALLRAIEESRFSIIIFSEHYASSSWCLDELTKILECV‑KVGGHT‑AFPVFYNVDPSHVRKQTGSYGVAFTKH‑EQVYRDN‑‑‑ME‑‑KVSKWREALTAVSG‑LSGW‑‑‑DS‑R‑‑‑‑NEHESEFIKEIV

>731431111

GKDTRNGFTAHLYEALCNKKI‑RTFMDADKIV‑KGEEISASLVTAMDKSMFCIVVLSKNYASSTWCLDELVQILKCK‑NAKKRT‑VLPIFYNVNPSDVREQKGSFAKAFAKLEEKF‑‑‑K‑‑‑EEM‑EVKMWKQALTEVAN‑VSGW‑‑‑DARD‑‑‑‑R‑HEPTLIKEIV

>225464430

GEDTRKNFSDHLYTTLIANGIHT‑FRDSEELD‑KGGDIASELSRVIQKSRIFIIIFSRNYATSKWCLNELVKITERM‑TQKEST‑IHPVFYHVNPSEVRHQSGSYGEAFSNY‑EKDDLEK‑‑‑EN‑‑IV‑KWRAALTQVGN‑LSGW‑‑‑HVDN‑‑‑‑‑‑YESEVLIGIT

>731436401

GEDTRNNFTAHLYHALCQKGINT‑FIDDDKLE‑RGQVISPALVAAIENSMYSIVVLSKNYASSRWCLQELVKIVECM‑KSKRQM‑VFPIFYDVDPSEVRRHRGTFGEALAKH‑EEN‑SEN‑‑‑ME‑‑RVESWKDALTQVAN‑LSGW‑‑‑DS‑R‑‑‑‑NKNEHLLIKGIV

>731430917

AEDTRYKFTDHLYAALRNRSIRT‑FRDDK‑LK‑RGEEIAPELLKVIEESRLSIVVFSENYASSRWCLDELVKIMECR‑QKIRQI‑VVPIFYHVDPSDLRKQKGSFGKAFASY‑ERHGRDS‑‑‑KE‑‑KIQRWRAALTEASN‑LSGW‑‑‑RL‑F‑‑‑‑EGYESDHIKKII

>731435262

GADTRYNFTDHLYKALDRRGIRT‑FRDDT‑LR‑RGEAIDPELLKAIEGSRSSVIVFSENYAHSRSCLDELVKIMECQ‑KDLGHT‑VIPIFYHVDPSHVRKQEGSFGAAFAGY‑EENWKD‑‑‑‑‑‑‑‑KIPRWRTALTEAAN‑LSGW‑‑‑HL‑Q‑‑‑‑DGYESDNIKKIT

>731431697

GEDTRNNFTDHLYTALVQRGINT‑FRDDK‑LR‑RGEEIAPELLKAIEESRSSIVVFSKTYAHSRWCLDELAKIMECR‑REYRQI‑VLPIFYHVDPADVRKQTGSFGEAFTSY‑EENWKN‑‑‑‑‑‑‑‑KAQRWREALTEAGY‑IAGW‑‑‑PI‑N‑‑‑‑KGYESRPIEEII

>731390030

GGDTRKNFTDHLYTTLTASGIQT‑FRDDEELE‑KGGDIASDLLRAIEESRFFIIIFSKNYAYSRWCLNELVKIIERK‑SQKESM‑VLPIFYHVDPSDVRNQRGSFGDALAYH‑E‑R‑‑‑D‑‑NQEK‑KIQKWRIALREAAN‑LSGC‑‑‑HV‑N‑‑‑‑DQYETEVVKEIV

>731410927

GEDTRKSFTDHLHSALCQYGINT‑FIDDQ‑FR‑RGEQISSALLRAIEESRFSIIVFSEHYASSSWCLDELTKILECV‑KVGGHT‑AFPVFYNVDPSHVRKQTGSYGVAFTKH‑EQVYRDN‑‑‑ME‑‑KVLKWREALTVASG‑LSGW‑‑‑DS‑R‑‑‑‑DRHESKVIKEII

>731431211

GEDTRNNFTSHLYKDLDKANIKT‑FKDDEELR‑KGGEIAPELLKAIEESRIAIIVFSKTYAHSKWCLDELVKIMECQ‑KEKGQI‑VYPVFYHVRPCEVRNQYGTYGEEFKKH‑E‑S‑‑‑N‑‑DEEK‑KIGEWRTALRKAGD‑LSGF‑‑‑SL‑R‑‑‑‑DRSEAEFIEEII

>147802475

GEDTRRSFTDHLYAALVEKGVRT‑FRDDEELE‑RGKEIAPELLKAIEESRISVVVFSKNYARSGWCMDELVKIIECM‑KAKGQT‑VLPVFYDVDPTHVRKQTGSFMEAFASH‑GED‑TEV‑‑‑IE‑‑RAKRWRAALTQAAN‑LSGW‑‑‑HL‑Q‑‑‑‑NGYESKLIKKII

>731431012

GEDTRTGFTDHLYAALVDKGIRT‑FRDSEELR‑RGEEIEGELLKAIHESRIFIIIFSEDYANSKWCLKELAEISKC‑‑KAKGRK‑VFPVFYHVDPSEVRNQSGYYGEAFAAY‑E‑N‑‑‑D‑‑NQDS‑‑IQVWRTALKEAGH‑IIGY‑‑‑HI‑D‑‑‑‑KEPEADVVKTIT

>9758205

GEDVRKSFLSHLLKKLHRKSI‑NTFID‑NNIE‑RSHAIAPDLLSAINNSMISIVVFSKKYASSTWCLNELVEIHKCY‑KELTQI‑VIPIFYEVDPSDVRKQTREFGEFFKVTCVGK‑‑‑T‑‑‑EDV‑K‑QQWIEALEEVAS‑IAGH‑‑‑DSKN‑W‑‑P‑NEANMIEHIA

>12324938

GPDVRKTFLSHLRKQFICNGT‑TMFDD‑QAIE‑RGQTISPELTRGIRESRISIVVLSKNYASSSWCLDELLEILKCK‑EDIGQI‑VMTVFYGVDPSDVRKQTGDILKVFKKTCSGK‑‑‑T‑‑‑EEK‑R‑RRWSQALNDVGN‑IAGE‑‑‑HFLN‑W‑‑D‑NESKMMEKIA

>685339467

GTDVRKGFLSHLYKALTDNGIHT‑FRDDAELQ‑RGNFISPALLGAIEQSRFAVVVLSENYATSRWCLQELVHITKCE‑KK‑‑QMELIPVFFGVDPSHVKRQSGNFAKAFAEH‑DKR‑‑‑‑‑‑NKD‑‑AVESWRKAMATVGF‑ISGW‑‑‑DS‑R‑‑‑WN‑EESKLIEELV

>685356609

GEDVRKNFLSHLLKEFENKGI‑LTFRD‑DQIE‑RSHSIGPELVEAIRESKISVVLFSENYASSSWCLDELVEILKCK‑EEQGLK‑VMPIFYKVDPSEVRKQTGKFGMGFLKTCHGK‑‑‑T‑‑‑EEQ‑Q‑QSWRQALTDAAS‑IVGD‑‑‑HPQD‑W‑‑D‑NEADMITTIA

>685355951

GQGLRQTFLSHLCRQLNENGITV‑FT‑NQDLV‑RGEPV‑PSLVQRIRESRISIVVLSQKYASSSWCLNELVEILRCR‑ET‑GHI‑VMTIFYRVDPSHVRNQTGDFGNIFVQTCAGK‑‑‑T‑‑EEE‑‑R‑RMWSQALTDVGN‑IAGE‑‑‑DSRN‑W‑‑D‑NESKMIEKIV

>971588095

GEDTRKTFTSHLYEGLKNRGIFT‑FQDDKRLE‑QGDSISKELLKAIEESQVALIVFSRNYATSRWCLNELVKVMECK‑EENGQT‑VIPIFYDVDPSHVRYQSESFAEVFAKH‑ESRYKDDE‑GMQ‑‑KVQGWRTALTAAAN‑LKGY‑‑‑DI‑‑‑‑‑RDG‑ESEKIQQIV

>460396445

GEDVRKTFVDHLYLALEQKCIYT‑FKDDEKLE‑KGKFISPELESSIEESRIALIIFSKNYADSTWCLDELTKIMECK‑NVKGQI‑VVPVFYDVDPSTVRKQKMIFGEAFSKH‑EARFQ‑‑‑‑‑ED‑‑KVQKWRAALEEAAN‑ISGW‑‑‑DM‑PN‑‑SNGHEARVIEKIA

>723668142

GEDTRKTFTDELYKALVDEGYRT‑FRDDNEIE‑RGEDIKSELDKAIHSSKSSIIILSKNYATSSWCLDELVMILENK‑RKRGHA‑ILPVFYYVDPLDVGKQMGSFATAFATH‑EQRIMEDDEGKE‑‑KIKRWRAALREVAD‑QSGM‑‑‑‑VLQ‑‑‑‑SSTELRFIEKLV

**Aligned *A. thaliana* sequences from Pfam used to build profile HMM after alignment pruning and exclusion.**

>Q9CAE0

GPDVRKTLLSNLREHFQGKGITMFDDE‑KIKRGGDLSPSLKRAIKTSKISIVILSQKYASSSWCLDELLEIMKRKKAM‑KQIVMTVFYGVEPSDVRKQTGDFGIAFNKT‑‑‑‑CV‑NKTDKE‑RKEWSKALTDVSNIAGEDFKKW‑DNEAN‑‑MIKKIA

>Q9FGT2

GPDVRKTFLSHLRKQFSYNGISMFNDQ‑SIERSQTIVPALTGAIKESRISIVVLSKNYASSRWCLDELLEILKCREDI‑GQIVMTVFYGVDPSDVRKQTGEFGIAFNKT‑‑‑‑CE‑GKTNEE‑TQKWSKALNDVGNIAGEHFFNW‑DNEAK‑‑MIEKIA

>Q9CAD8

GPDVRKTVLSHLRKQFICNGITMFDDQ‑RIERGQTISPELTRGIRESRISIVVLSKNYASSSWCLDELLEILKCKEDI‑GQIVMTVFYGVDPSDVRKQTGEFGIRFSET‑‑‑‑WA‑RKTEEE‑KQKWSQALNDVGNIAGEHFLNW‑DKESK‑‑MVETIA

>Q9CAK1

GPDVRKTLLSHIRLQFNRNGITMFDDQ‑KIVRSATIGPSLVEAIKESRISIVILSKKYASSSWCLDELVEILECKKAM‑GQIVMTIFYGVDPSDVRKQIGKFGIAFNET‑‑‑‑CA‑RKTEEE‑RQKWSKALNQVSNIAGEDFLRW‑DNEAI‑‑MIEKIA

>Q9CAK0

GPNVRKTLLSHMRKQFNFNGITMFDDQ‑GIERSEEIVPSLKKAIKESRISIVILSKKYALSRWCLDELVEILKCKEVM‑GHIVMTIFYGVEPSDVRKQTGEFGFHFNET‑‑‑‑CA‑HRTDED‑KQNWSKALKDVGNIAGEDFLRW‑DNEAK‑‑MIEKIA

>F4I3S8

GPDVRKTLLSHMRKQFDFNGITMFDDQ‑GIERSEEIAPSLKKAIKESRISIVILSKKYASSSWCLDELVDILKRKKAM‑KQIVMTVFYGVEPFEVRNQTGEFGIAFNET‑‑‑‑CA‑RKTDEE‑RQKWSKALNEVANIAGEDFLRC‑DNEAK‑‑RIEKIA

>F4I3S7

GPDVRKTLLSHMRKQFDFNGITMFDDQ‑GIERSEEIAPSLKKAIKESRISIVILSKKYASSSWCLDELVDILKRKKAM‑KQIVMTVFYGVEPFEVRNQTGEFGIAFNET‑‑‑‑CA‑RKTDEE‑RQKWSKALNEVANIAGEDFLRC‑DNEAK‑‑RIEKIA

>Q9C7X0

GPDVRIKFLSHLRQQFVYNGITMFDDN‑GIERSQIIAPALKKAIGESRVAIVLLSKNYASSSWCLDELLEILKCKEYI‑GQIVMTVFYEVDPSHVRKQTGDFGIAFKET‑‑‑‑CA‑HKTEEE‑RSKWSQALTYVGNIAGEDFIHW‑KDEAK‑‑MIEKIA

>F4I552

GPDVRIKFLSHLRQQFIYNGITMFDDN‑GIERSQIIAPALKKAIGESRIAILLLSKNYASSSWSLDELLEILKCKEDI‑GQIVMTVFYEVDPSDVRNQTGDFGIAFKET‑‑‑‑CA‑HKTEEE‑RQKWTQALTYVGNIAGEDFKHW‑PNEAK‑‑MIEKIA

>Q9C7W9

GPDVRIKFLSHLRQQFIYNGITMFDDN‑GIERSQIIAPALKKAIGESRIAILLLSKNYASSSWSLDELLEILKCKEDI‑GQIVMTVFYEVDPSDVRNQTGDFGIAFKET‑‑‑‑CA‑HKTEEE‑RQKWTQALTYVGNIAGEDFKHW‑PNEAK‑‑MIEKIA

>Q9FXA6

GPDVRKTFLSHLRKQFNYNGITMFDDQ‑RIERSQIIAPALTEAIRESRIAIVLLSKNYASSSWCLDELLEILDCKEQL‑GQIVMTVFYGVHPSDVRKQTGDFGIAFNET‑‑‑‑CA‑RKTEEQ‑RQKWSQALTYVGNIAGEHFQNW‑DNEAK‑‑MIEKIA

>Q9C515

GPDVRNTFLSHLRKQFNTNGITMFDDQ‑RMERSQTLAPTLTQAIRESKIYIVLLSKNYASSSWCLDELLEILNCKEKR‑GQRVMTIFYGVNPSDVRKQTGEFGIAFNET‑‑‑‑CA‑RKTEEE‑RRKWSHALTCVGNITGVHVQDR‑DDEAN‑‑MIEKIA

>Q9ZVX6

GPDVRKSFLSHFRKQFICNGITMFDDQ‑KIVRSQTIAPSLTQGIRESKISIVILSKNYASSTWCLNELLEILKCREDI‑GQIVMTVFYGVDPSDVRKQTGEFGTVFNKT‑‑‑‑CA‑RRTEKE‑RRNWSQALNVVGNIAGEHFLNW‑DNEAE‑‑MIEKIA

>F4I594

GSDVRTSFLSHFRKQFNNNGITMFDDQ‑RILRGETISPALTQAIRESRISIVLLSKNYASSGWCLDELLEILKCKDDM‑GQIVMTVFYGVDPSDVRKQTGEFGIAFNET‑‑‑‑CA‑CRTEEE‑RQKWSQALNYVGNIAGEHLLNW‑DNEAK‑‑MIEKIA

>Q9FMB7

GGDIRKTFLSHLRKQFNSNGITMFDDQ‑GIERSQTIAPALIQAIRESRISIVVLSKNYASSSWCLNELVEILKCKD‑‑‑‑‑VVMPIFYEVDPSDVRKQTGDFGKAFKNS‑‑‑‑CK‑SKTKEE‑RQRWIQALIFVGNIAGEHSLKW‑ENEAD‑‑MIEKIA

>Q9FFS6

GSDVRRKFLSHLRFHFAIKGIVAFKDQ‑EIERGQRIGPELVQAIRESRVSLVVLSKNYPSSSWCLDELVEILKCKEDQ‑EQIVMPIFYEIDPSDVRKQSGDFGKAFGKT‑‑‑‑CV‑GKTKEV‑KQRWTNALTEAANIGGEHSLNW‑TDEAE‑‑MIEKIV

>F4JYI5

GPDVRKGFLSHLHSLFASKGITTFNDQ‑NIERGQTIGPELIQGIKEARVSIVVLSKNYASSSWCLDELVEILKCKEAL‑GQIVMT‑‑‑‑‑‑‑‑‑‑‑‑‑SGVFGKAFEKT‑‑‑‑CQ‑GKNEEV‑KIRWRNALAHVATIAGEHSLNW‑DNEAK‑‑MIQKIA

>F4JYI4

GPDVRKGFLSHLHSLFASKGITTFNDQ‑NIERGQTIGPELIQGIKEARVSIVVLSKNYASSSWCLDELVEILKCKEAL‑GQIVMT‑‑‑‑‑‑‑‑‑‑‑‑‑SGVFGKAFEKT‑‑‑‑CQ‑GKNEEV‑KIRWRNALAHVATIAGEHSLNW‑DNEAK‑‑MIQKIA

>Q9LSX5

GPDVRKGFLSHLHSVFASKGITTFNDQ‑KIDRGQTIGPELIQGIREARVSIVVLSKKYASSSWCLDELVEILKCKEAL‑GQIVMTVFYEVDPSDVKKQSGVFGEAFEKT‑‑‑‑CQ‑GKNEEV‑KIRWRNALAHVATIAGEHSLNW‑DNEAK‑‑MIQKIV

>Q9FFS5

GPDVRKGFLSHLHYHFASKGITTFKDQ‑EIEKGNTIGPELVNAIRESRVSIVLLSKKYASSSWCLDELVEILKCKEDQ‑GQIVMTIFYDVDPSSVRKQKGDFGSTFMKT‑‑‑‑CE‑GKSEEV‑KQRWTKALTHVANIKGEHSLNW‑ANEAD‑‑MIQKIA

>Q9FKR7

GPDVRSGFLSHLHNHFESKGITPFKDQ‑EIERGHTIGPELIQAIRESRVSIVVLSEKYASSCWCLDELVEILKCKEAS‑GQVVMTIFYKVDPSDVRKQRGDFGSTFKKT‑‑‑‑CE‑GKTWIV‑KQRWIKALEYIATVAGEHSLSW‑ANEAE‑‑LIQKIA

>O82500

GEDVRNNFLSHLLKEFESKGIVTFRDD‑HIKRSHTIGHELRAAIRESKISVVLFSENYASSSWCLDELIEIMKCKEEQ‑GLKVMPVFYKVDPSDIRKQTGKFGMSFLET‑‑‑‑CC‑GKTEER‑QHNWRRALTDAANILGDHPQNW‑DNEAY‑‑KITTIS

>F4JWL8

GKDVRRTFLSHLLKEFRRKGIRTFIDN‑DIKRSQMISSELVRAIRESRIAVVVLSRTYASSSWCLNELVEIKK‑‑‑‑V‑SQMIMPVFYEVDPSDVRKRTGEFGKAFEEA‑‑‑‑CE‑RQPDEEVKQKWREALVYIANIAGESSQNW‑DNEAD‑‑LIDKIA

>Q9FJG4

GEDVRRNFLSHLHKELQHNGIDAFKDG‑GIKRSRSIWPELKQAIWESKIFIVVLSKNYAGSCWCLDELVEIMECREVV‑GKTLVPIFYDVDPSSVRKQTGDFGKAFDKI‑‑‑‑CD‑VRTEEE‑RQRWRQALTNVGNIAGECSSKW‑DNDAK‑‑MIEKIV

>Q9FH20

GEDVRGNFLSHLMKEFESKGIVTFKDD‑LIERSQTIGLELKEAVRQSKIFVVIFSKNYASSSWCLDELVEILKCKEE‑‑‑RRLIPIFYKVNPSDVRNQTGKFGRGFRET‑‑‑‑CE‑GKNDET‑QNKWKAALTEAANIAGEDSQSW‑KNEAD‑‑FLTKIA

>F4JWM0

GEDVRKGFLSHIQKEFKSKGIVPFIDD‑EMKRGESIGPGLFQAIRESKIAIVLLSKNYASSSWCLNELVEIMNCREEI‑GQTVMTVFYQVDPSDVRKQTGDFGKAFKKT‑‑‑‑CV‑GKTQEV‑KQRWSRALMDVANILGQDSRKW‑DKEAD‑‑MIVKVA

>F4JWL7

GEDVRKGFLSHIQKEFERKGIFPFVDT‑KMKRGSSIGPVLSDAIIVSKIAIVLLSKNYASSTWCLNELVNIMKCREEF‑GQTVMTVFYEVDPSDVRKQTGDFGIAFETT‑‑‑‑CV‑GKTEEV‑KQSWRQALIDVSNIVGEVYRIW‑SKESD‑‑LIDKIA

>F4IFF6

GADVRKSFLSHILKEFKRKGIDTFIDN‑NIERSKSIGPELIEAIKGSKIAVVLLSKDYASSSWCLNELVEIMKCRKML‑DQTVMTIFYEVDPTDVKKQTGDFGKVFKKT‑‑‑‑CM‑GKTNAV‑SRKWIEALSEVATIAGEHSINW‑DTEAA‑‑MIEKIS

>F4J3L8

GEDVRKDFLSHIQKEFQRQGITPFVDN‑NIKRGESIGPELIRAIRGSKIAIILLSKNYASSSWCLDELVEIIKCKEEM‑GQTVIVIFYKVDPSLVKKLTGDFGKVFRNT‑‑‑‑CK‑GKEREN‑IERWREAFKKVATIAGYDSRKW‑DNESG‑‑MIEKIV

>Q9LFN1

GEDVRRDFLSHIQMEFQRMGITPFVDN‑EIKRGESIGPELLRAIRGSKIAIILLSRNYASSKWCLDELVEIMKCREEY‑GQTVMAIFYKVDPSDVKNLTGDFGKVFRKT‑‑‑‑CA‑GKPKKD‑IGRWRQAWEKVATVAGYHSINW‑DNEAA‑‑MIKKIA

>F4I270

GDDVRRNFLSHIQKEFRRKGITPFIDN‑EIRRGESIGPELIKAIRESKIAIVLLSRNYASSKWCLEELVEIMKCKKEF‑GLTVFAIFYEVDPSHVKKLTGEFGAVFQKT‑‑‑‑CK‑GRTKEN‑IMRWRQAFEEVATIAGYDSRNW‑ENEAA‑‑MIEEIA

>F4IBL4

GEDVRRGFLSHIHKEFQRKGITPFIDN‑EIKRGESIGLEIIHAIRESKIAIVLLSRNYASSSWCLDELVEIMKCKEEF‑SQIVIPIFYRVDPSDVKKLTGNFGNVFKNN‑‑‑‑CV‑GKTNEV‑IRKWRQALAKMGTTTGYDSRNW‑DNEAT‑‑MIENIA

>Q9SS05

GEDVRRGFLSHIHKEFQRKGITPFIDN‑EIKRGESIGLEIIHAIRESKIAIVLLSRNYASSSWCLDELVEIMKCKEEF‑SQIVIPIFYRVDPSDVKKLTGNFGNVFKNN‑‑‑‑CV‑GKTNEV‑IRKWRQALAKMGTTTGYDSRNW‑DNEAT‑‑MIENIA

>Q9FKM9

GADVRKTFLSHMLKEFKRKGIVPFIDN‑DIDRSKSIGPELDEAIRGSKIAIVMLSKNYASSSWCLNELVEITKCRKDL‑NQTVMTIFYGVDPTDVKKQTGEFGKVFERT‑‑‑‑CE‑SKTEEQ‑VKTWREVLDGAATIAGEHWHIW‑DNEAS‑‑MIEKIS

>Q9FI14

GEDVRKGLLSHIQKEFQRNGITPFIDN‑EMKRGGSIGPELLQAIRGSKIAIILLSRNYGSSKWCLDELVEIMKCREEL‑GQTVMTVFYDVDPSDVRKQKGDFGKVFKKT‑‑‑‑CV‑GRPEEM‑VQRWKQALTSAANILGEDSRNW‑ENEAD‑‑MIIKIS

>F4J361

GADVRKTILSHILESFRRKGIDPFIDN‑NIERSKSIGHELKEAIKGSKIAIVLLSKNYASSSWCLDELAEIMKCRELL‑GQIVMTIFYEVDPTDIKKQTGEFGKAFTKT‑‑‑‑CK‑GKTKEY‑VERWRKALEDVATIAGEHSRNW‑RNEAD‑‑MIEKIA

>F4J359

GADVRRTFLSHIMESFRRKGIDTFIDN‑NIERSKSIGPELKEAIKGSKIAIVLLSRKYASSSWCLDELAEIMKCRQMV‑GQIVMTIFYEVEPTDIKKQTGEFGKAFTKT‑‑‑‑CR‑GKPKEQ‑VERWRKALEDVATIAGYHSHSW‑RNEAD‑‑MIEKIA

>Q9M1P1

GADVRRTFLSHIMESFRRKGIDTFIDN‑NIERSKSIGPELKEAIKGSKIAIVLLSRKYASSSWCLDELAEIMKCRQMV‑GQIVMTIFYEVEPTDIKKQTGEFGKAFTKT‑‑‑‑CR‑GKPKEQ‑VERWRKALEDVATIAGYHSHSW‑RNEAD‑‑MIEKIA

>F4J339

GADVRRTFLSHIMESFRRKGIDTFIDN‑NIERSKSIGPELKEAIKGSKIAIVLLSRKYASSSWCLDELAEIMKCRQMV‑GQIVMTIFYEVDPTDIKKQTGEFGKAFTKT‑‑‑‑CR‑GKPKEQ‑VERWRKALEDVATIAGYHSHSW‑RNEAD‑‑MIEKIS

>Q9M285

GADVRRTFLSHIKESFRRKGIDTFIDN‑NIERSKSIGPELKEAIKGSKIAIVLLSRKYASSSWCLDELAEIMKCREMV‑GQIVMTIFYEVEPTDIKKQTGEFGKAFTKT‑‑‑‑CR‑GKTKEH‑IERWRKALEDVATIAGYHSHKW‑CDEAE‑‑MIEKIS

>Q8H1N6

GADVRKSFLSHIMKEFKSKGIDIFIDK‑DIKRGKSIGPELTEAIRGSRVAIVFLSRKYASSSWCLNELALIMKCRKEL‑GLTVMTLFYDLDPTDVRKQTGDFGMAFKET‑‑‑‑CK‑GKTKDE‑IGRWRHALEEVAKIAGYHSSIW‑DNEAD‑‑MIGIVT

>F4J910

GEAEESSFNEALMKEFQRKGITPFNDN‑EIKRGESISPELVLAIRGSRIALILLSRNYASSSWCLDELAEIIKCREEF‑GQTVMVVFYKVDPSDIKKLTGDFGSVFRKT‑‑‑‑CA‑GKTNED‑TRRWIQALAKVATLAGYVSNNW‑DNEAV‑‑MIEKIA

>Q8LPH7

GEDVRVTFRSHFLKELDRKLITAFRDN‑EIERSHSLWPDLEQAIKESRIAVVVFSKNYASSSWCLNELLEIVNCND‑‑‑‑KIVIPVFYHVDPSQVRHQIGDFGKIFENT‑‑‑‑CK‑RQTDEEVKNQWKKALTLVANMLGFDSAKW‑NDEAK‑‑MIEEIA

>F4I9F1

GEDVRVTFRSHFLKELDRKLITAFRDN‑EIERSHSLWPDLEQAIKESRIAVVVFSKNYASSSWCLNELLEIVNCND‑‑‑‑KIVIPVFYHVDPSQVRHQIGDFGKIFENT‑‑‑‑CK‑RQTDEEVKNQWKKALTLVANMLGFDSAKW‑NDEAK‑‑MIEEIA

>F4KG41

GGDVRVTFRSHFLKELDRKLITAFRDN‑EIERSHSLWPDLEQAIKDSRIAVVIFSKNYASSSWCLNELLEIVNCND‑‑‑‑KIVIPVFYGVDPSQVRHQIGDFGKIFEKT‑‑‑‑CK‑RQT‑EQVKNQWKKALTDVANMLGFDSATW‑DDEAK‑‑MIEEIA

>Q9FL35

GGDVRVTFRSHFLKEFDRKLITAFRDN‑EIERSHSLWPDLEQAIKESRIAVVVFSKNYASSSWCLNELLEIVNCND‑‑‑‑KIIIPVFYGVDPSQVRYQIGEFGKIFEKT‑‑‑‑CK‑RQT‑EEVKNQWKKALTHVANMLGFDSSKW‑DDEAK‑‑MIEEIA

>Q93ZC0

GGDVRVTFRSHFLKEFDRKLITAFRDN‑EIERSHSLWPDLEQAIKDSRIAVVVFSKNYASSSWCLNELLEIVNCND‑‑‑‑KIIIPVFYGVDPSQVRYQIGDFGRIFEKT‑‑‑‑CK‑RQT‑EEVKNQWKKALTLVANMLGFDSAKW‑DDEAK‑‑MIEEIA

>Q9FHF6

GGDVRVTFRSHFLKEFDRKLITAFRDN‑EIERSHSLWPDLEQAIKDSRIAVVVFSKNYASSSWCLNELLEIVNCND‑‑‑‑KIIIPVFYGVDPSQVRYQIGDFGRIFEKT‑‑‑‑CK‑RQT‑EEVKNQWKKALTLVANMLGFDSAKW‑DDEAK‑‑MIEEIA

>F4KHI3

GKDVRVTFRSHFLKELDRKLISAFRDN‑EIERSHSLWPDLEQAIKDSRIAVVVFSKNYASSSWCLNELLEIVNCND‑‑‑‑KIIIPVFYGVDPSQVRYQIGEFGSIFEKT‑‑‑‑CK‑RQT‑EEVKNQWKKALTDVANMLGFDSAKW‑DDEAK‑‑MIEEIA

>Q9FHF4

GTDVRRNFLSHLLKGL‑HKSVNSFRDQ‑NMERSQSLDPMLKQAIRDSRIALVVFSKNYASSSWCLNELLEIVKCKEEF‑GQMVIPIFYCLDPSHVRHQDGDFGKNFEET‑‑‑‑CG‑RNT‑EEEKIQWEKALTDVANLAGFDSVTW‑DDEAK‑‑MIEEIA

>F4KHH8

GEDVRNTFLSHFLKELDRKLIISFKDN‑EIERSQSLDPELKHGIRNSRIAVVVFSKTYASSSWCLNELLEIVKCKKEF‑GQLVIPIFYNLDPSHVRKQTGDFGKIFEKT‑‑‑‑CR‑NKT‑VDEKIRWKEALTDVANILGYHIVTW‑DNEAS‑‑MIEEIA

>Q9FHG0

GEDVRKTFLSHFLRELERKSIITFKDN‑EMERSQSIAPELVEAIKDSRIAVIVFSKNYASSSWCLNELLEIMRCNKYL‑GQQVIPVFYYLDPSHLRKQSGEFGEAFKKT‑‑‑‑CQ‑NQT‑EEVKNQWKQALTDVSNILGYHSKNC‑NSEAT‑‑MIEEIS

>Q9SW60

GEDIRVTFLTHFLKELDRKMIIAFKDN‑EIERGNSIGTELIQAIKDSRIAVVVFSKKYSSSSWCLNELVEIVNCKE‑‑‑‑‑IVIPVFYDLDPSDVRKQEGEFGESFKET‑‑‑‑CK‑NRTDYEIQ‑RWGQALTNVANIAGYHTRKP‑NNEAK‑‑LIEEIT

>Q9FNJ2

GEDVRVTFLSHFLKELDRKLISVFKDN‑DIQRSQSLDPELKLAIRDSRIAIVVFSKNYAASSWCLDELLEIVKCKEEF‑GQIVIPVFYGLDPCHVRKQSGEFGIVFENT‑‑‑‑CQ‑TKTDDEIQ‑KWRRALTDVANILGFHSSNW‑DNEAT‑‑MVEDIA

>O81430

VEDIRQTFLSHFLKDLDRKLIIAFKDN‑EIERSQSLNPDLKRPIRDSRIAIVIFSKNYASSSWCLNELLEIVRCKEDSNRLVVIPVFYGLDPSHVRKQIGNFGKIFKKT‑‑‑‑CQ‑NRTEDEINLR‑RRALIDVANTLGYHSTIC‑‑‑KAN‑‑MTKEIT

>Q9FT77

GEDVRHSLVSHLRKELDRKFINTFNDN‑RIERSRKITPELLLAIENSRISLVVFSKNYASSTWCLDELVKIQECYEKL‑DQMVIPIFYKVDPSHVRKQTGEFGMVFGET‑‑‑‑CK‑GR‑TENEKRKWMRALAEVAHLAGEDLRNW‑RSEAE‑‑MLENIA

>F4JNB2

GVDVRKTFLSNLLEAFDRRSINTFMDH‑GIERSRTIAPELISAIREARISIVIFSKNYASSTWCLDELVEIHNRLNDW‑GQLVISVFYDVDPSEVRKQTGEFGDVFKKT‑‑‑‑CE‑DK‑EEDQKQRWMQALVDITNIAGEDLRNG‑PSEAA‑‑MVVKIA

>O23536

GVDVRKTFLSHLIEALDRRSINTFMDH‑GIVRSCIIADELITAIREARISIVIFSENYASSTWCLNELVEIHKCHKDL‑DQMVIPVFYGVDPSHVRKQIGGFGDVFKKT‑‑‑‑CE‑DK‑PEDQKQRWVKALTDISNLAGEDLRNG‑PSEAA‑‑MVVKIA

>F4JNB7

GVDVRKTFLSHLIEALDGKSINTFIDH‑GIERSRTIAPELISAIREARISIVIFSKNYASSTWCLNELVEIHKCFNDL‑GQMVIPVFYDVDPSEVRKQTGEFGKVFEKT‑‑‑‑CEKDKQPGDQKQRWVQALTDIANIAGEDLLNG‑PNEAH‑‑MVEKIS

>F4JNB8

GVDVRKTFLSHLIEALDGKSINTFIDH‑GIERSRTIAPELISAIREARISIVIFSKNYASSTWCLNELVEIHKCFNDL‑GQMVIPVFYDVDPSEVRKQTGEFGKVFEKT‑‑‑‑CEKDKQPGDQKQRWVQALTDIANIAGEDLLNG‑PNEAH‑‑MVEKIS

>Q9SUK4

GEDVRKNFLSHLLKQLNRRSINTFMDH‑VIERSCIIADALISAIREARISIVIFSKNYAASTWCLNELVEIDNCSKYF‑GQKVIPVFYDVDPSHVRKQIGEFGKVFKKT‑‑‑‑CE‑DK‑PADQKQRWVKALTDISNIAGEDLRNG‑PNDAH‑‑MVEKIA

>F4JNA9

GVDVRKTFLSHLIEALDRRSINTFMDH‑GIVRSCIIADALITAIREARISIVIFSENYASSTWCLNELVEIHKCYKKG‑EQMVIPVFYGVDPSHVRKQIGGFGDVFKKT‑‑‑‑CE‑DK‑PEDQKQRWVKALTDISNLAGEDLRNG‑PTEAF‑‑MVKKIA

>O23530

GEDVRDSFLSHLLKELRGKAI‑TFIDD‑EIERSRSIGPELLSAIKESRIAIVIFSKNYASSTWCLNELVEIHKCYTNL‑NQMVIPIFFHVDASEVKKQTGEFGKVFEET‑‑‑‑CK‑AK‑SEDEKQSWKQALAAVAVMAGYDLRKW‑PSEAA‑‑MIEELA

>F4JT78

KVDVRRSFLAHLLKELDRRLINTFTDH‑GMERNLPIDAELLSAIAESRISIVIFSKNYASSTWCLDELVEIHTCYKEL‑AQIVVPVFFNVHPSQVKKQTGEFGKVFGKT‑‑‑‑CK‑GK‑PENRKLRWMQALAAVANIAGYDLQNW‑PDEAV‑‑MIEMVA

>F4KDB8

GEDVRKSFLSHLLKKLHRKSINTFIDN‑NIERSHAIAPDLLSAINNSMISIVVFSKKYASSTWCLNELVEIHKCYKEL‑TQIVIPIFYEVDPSDVRKQTREFGEFFKVT‑‑‑‑CV‑GK‑TEDVKQQWIEALEEVASIAGHDSKNW‑PNEAN‑‑MIEHIA

>Q0WPW2

GEDVRKSFLSHLLKKLHRKSINTFIDN‑NIERSHAIAPDLLSAINNSMISIVVFSKKYASSTWCLNELVEIHKCYKEL‑TQIVIPIFYEVDPSDVRKQTREFGEFFKVT‑‑‑‑CV‑GK‑TEDVKQQWIEALEEVASIAGHDSKNW‑PNEAN‑‑MIEHIA

>O65506

SVDVPKSFLSRIRKELRRKGFEPLIDN‑ETERCVSIGPELRNAISVSRIVIVVLSRNYALSPWCLDELVEIMKCKEEL‑GQRVVTIFYNLDPIDVLKQTGDFGDNFRKTRKGKCE‑RKNKED‑IDRWIKALEQVATIDGYRSRDW‑DDEKA‑‑MVKKIA

>F4IF04

GKDTRKNFVSFLYKALVSKGIRTFKDDEELERGRPIPPELRQAIKGSRIAVVVVSVTYPASSWCLEELREILKLE‑KLGLLTVIPIFYEINPSDVRRQSGVVSKQFKKHEK‑‑‑‑R‑QSRER‑VKSWREALTKLASLSGECSKNWREDDSK‑‑LVDGIT

>F4IF05

GKDTRKNFVSFLYKALVSKGIRTFKDDEELERGRPIPPELRQAIKGSRIAVVVVSVTYPASSWCLEELREILKLE‑KLGLLTVIPIFYEINPSDVRRQSGVVSKQFKKHEK‑‑‑‑R‑QSRER‑VKSWREALTKLASLSGECSKNW‑EDDSK‑‑LVDGIT

>Q9SSN3

GHDTRHNFISFLYKELVRRSIRTFKDDKELENGQRFSPELKSPIEVSRFAVVVVSENYAASSWCLDELVTIMDFE‑KKGSITVMPIFYGVEPNHVRWQTGVLAEQFKKHAS‑‑‑‑R‑EDPEK‑VLKWRQALTNFAQLSGDCSG‑‑‑DDDSK‑‑LVDKIA

>Q9SSN5

GHDTRQNFISFLYKELVRRSIRTFKDDKELENGQRISSELKRTIEVSRFAVVVVSETYAASSWCLDELVTIMDFE‑KKGSITVMPIFYGVEPNHVRWQTGVLAEQFKKHGS‑‑‑‑R‑EDHEK‑VLKWRQALTNFAQLSGDCSG‑‑‑DDDSK‑‑LVDKIA

>Q9SSN1

GLDTRRSFISFLYKELIRRNIRTFKDDKELKNGRRITPELIRAIEGSRFAVVVVSVNYAASRWCLEELVKIMDFE‑NMGSLKVMPIFYGVDPCHVRRQIGEVAEQFKKHEG‑‑‑‑R‑EDHEK‑VLSWRQALTNLASISGDCSWKW‑EDDSK‑‑MVEEIT

>Q9SSN4

GLDTRRNFISFLYKELVRRKIRTFKDDKELENGRRISPELKRAIEESKFAVVVVSVNYAASPWCLDELVKIMDFE‑NKGSITVMPIFYGVDPCHLRRQIGDVAEQFKKHEA‑‑‑‑REEDHEK‑VASWRRALTSLASISGECSLKW‑EDEAN‑‑LVDEIA

>Q9SSN2

GVDTRRNFISFLYKEFVRRKIRTFKDDKELENGRRISPELKRAIEESKFAVVVVSVNYAASPWCLDELVKIMDFE‑NKGSITVMPIFYGVDPCHLRRQIGDVAEQFKKHEA‑‑‑‑REEDHEK‑VASWRRALTSLASISGDCSSKC‑EDEAK‑‑LVDEIA

>Q9SSN6

GPDTRRKFISFLYKELVGRDIRTFKDDKELENGQMISPELILAIEDSRFAVVVVSVNYAASSWCLDELVKIMDIQKNKGSITVMPIFYGVNPCHLRRQIGDVAEQFKKHEA‑‑‑‑REKDLEK‑VLKWRQALAALADISGDCSG‑‑‑EDDSK‑‑LVDVIA

>Q4PT31

GKDTRRTFISFLYKELIEMGIRTFKDDVELQSGRRIASDLLAAIENSKIAVVIISKNYSASPWCLQELVMIMDVE‑KKGSIIVMPIFYNVEPAHVRRQIEQVAKQFRKHEG‑‑‑‑R‑ENYET‑VVSWRQALTNLASISGHCSRDC‑EDDSK‑‑LLDEIT

>F4KFY5

GEDTRHSIVSHLYEALTSRGIATFKDDKRLELGDHISEELQRAIEGSDFVVVVLSENYPTSRWCLMELQSIMELQ‑MEGRLGVFPVFYRVEPSAVRYQLGSF‑‑DLEGYQR‑‑‑‑DPQMADM‑VPKWRQALKLIADLSGVASGQC‑IDEAT‑‑MVRKIV

>F4I901

GEDTRKTIVSHLYAALDSRGIVTFKDDQRLEIGDHISDELHRALGSSSFAVVVLSENYATSRWCLLELQLIMELM‑KEGRLEVFPIFYGVDPSVVRHQLGSF‑‑SLVKYQ‑‑‑‑‑GLEMVDK‑VLRWREALNLIANLSGVVSSHC‑VDEAI‑‑MVGEIA

>Q0WQ93

GVDTRQTIVSHLYVALRNNGVLTFKDDRKLEIGDTIADGLVKAIQTSWFAVVILSENYATSTWCLEELRLIMQLH‑SEEQIKVLPIFYGVKPSDVRYQEGSFATAFQRYEA‑‑‑‑DPEMEEK‑VSKWRRALTQVANLSGKHSRNC‑VDEAD‑‑MIAEVV

>F4IEZ7

GVDTRQTIVSHLYVALRNNGVLTFKDDRKLEIGDTIADGLVKAIQTSWFAVVILSENYATSTWCLEELRLIMQLH‑SEEQIKVLPIFYGVKPSDVRYQEGSFATAFQRYEA‑‑‑‑DPEMEEK‑VSKWRRALTQVANLSGKHSRNC‑VDEAD‑‑MIAEVV

>Q9M0P9

GADTRNNIVSYLHKALVDVGIRTFKDDKELEEGDIISEKLVNAIQTSWFAVVVLSEKYVTSSWCLEELRHIMELS‑IQDDIIVVPIFYKVEPSDVRYQKNSFEVKLQHYR‑‑‑‑‑DP‑‑‑EK‑ILKWKGALTQVGNMSGKHFQTC‑SDEAT‑‑NIAEIV

>Q9FKB9

GEDTRRTIVSHLYAALGAKGIITFKDDQDLEVGDHISSHLRRAIEGSKFAVVVLSERYTTSRWCLMELQLIMELY‑NLGKLKVLPLFYEVDPSDVRHQRGSF‑‑GLERYQ‑‑‑‑‑GPEFADI‑VQRWRVALCMVANLSGMVSRYC‑ADEAM‑‑MLEEIV

>F4IF00

GEDTRKNIVSHLHKQLVDKGVVTFKDDKKLELGDSISEEISRAIQNSTYALVILSENYASSSWCLDELRMVMDLH‑LKNKIKVVPIFYGVDPSHVRHQTGSF‑‑TFDKYQ‑‑‑‑‑DSKMPNK‑VTTWREALTQIASLAGKDFETC‑EDEAS‑‑MIEEIV

>F4KIC7

GEDVRKTFVSHLFCEFDRMGIKAFRDDLDLQRGKSISPELIDAIKGSRFAIVVVSRNYAASSWCLDELLKIMECN‑‑‑‑KDTIVPIFYEVDPSDVRRQRGSFGEDVESHS‑‑‑‑‑‑‑‑DKEK‑VGKWKEALKKLAAISGEDSRNW‑‑DDSK‑‑LIKKIV

>B3H776

GADVRKNFLSHLYDSLRRCGISTFMDDVELQRGEYISPELLNAIETSKILIVVLTKDYASSAWCLDELVHIMKSHKNNPSHMVFPIFLYVDPSDIRWQQGSYAKSFSKHKN‑‑‑‑‑SHPLNK‑LKDWREALTKVANISGWDIKN‑‑RNEAE‑‑CIADIT

>Q9FKB8

GLDTRRTFVSHLRRSLDRKGIKTFEDENESLRGELDSSAVYQTIGESKVAVVLISVNYASSPLCLDSLLKILKFHQSG‑SLVLIPIFYEVDPMDVRKQIGKLYEAFSLHE‑‑‑‑‑‑RENPEK‑VQTWRQALSQLVSIGGQYSEIW‑DGDAE‑‑LIHQIT

>F4K382

GLDTRRTFVSHLRRSLDRKGIKTFEDENESLRGELDSSAVYQTIGESKVAVVLISVNYASSPLCLDSLLKILKFHQSG‑SLVLIPIFYEVDPMDVRKQIGKLYEAFSLHE‑‑‑‑‑‑RENPEK‑VQTWRQALSQLVSIGGQYSEIW‑DGDAE‑‑LIHQIT

>F4HR52

‑RDARHKFTERLYEVLVKEQVRVW‑NNDDVERGNELGASLVEAMEDSVALVVVLSPNYAKSHWCLEELAMLCDLKSSL‑GRLVLPIFYEVEPCMLRKQNGPYEMDFEEHSKRF‑‑‑SE‑‑EK‑IQRWRRALNIIGNIPGFVYSKD‑‑KDDD‑‑MIELVV

>F4HR53

GADTRDNFGDHLYKAL‑KDKVRVFRDNEGMERGDEISSSLKAGMEDSAASVIVISRNYSGSRWCLDELAMLCKMKSSL‑DRRILPIFYHVDPSHVRKQSDHIKKDFEEHQVRF‑‑‑SEEKEK‑VQEWREALTLVGNLAGYVCDKD‑‑KDDD‑‑MIELVV

>F4HR54

GFDTRTNFCERLYIALEKQNVRVFRDNEGMEKGDKIDPSLFEAIEDSAASVIILSTNYANSSWCLDELALLCDLRSSL‑KRPMIPIFYGVNPEDVRKQSGEFRKDFEEKAKSF‑‑‑DE‑‑ET‑IQRWKRAMNLVGNIPGYVCTAK‑EKVDD‑‑MIDLVV

>Q9M0Q0

‑‑‑VEATLVSDLRSSFSENGI‑TMKDD‑DLEKGVSLGSERSEGIRESKVAVVVISQSYAISAQCLNELQTIVNFHDER‑RISILPIFYGVDYDDVRNQIKELAASFRKLGK‑‑‑‑‑EYPSEK‑VQAWMIALIKLINISRSDSRIHKDKQIN‑‑LKATIS

>Q9SSN9

REDTGRTFVSHLYRSLDQKEIRTYKENQQAGDGKRISSEVKQAINESRIAVVVISENYVSSVLCLDVLAKII‑‑‑ERLPLIKIETVFYEVDPGDLTRPTGKFADDFRRHEA‑‑‑‑‑RENRRT‑VNRWRDALDQLVSISNFCSRNW‑EDDSK‑‑MIDDLA

>Q9SCZ3

‑EDTVRSFVSHLCAAFRRRGISSFIEN‑GSD‑‑‑‑SESNGFSKLETSRASVVVFSEKYSSSKSCMEELVKVSERRRKN‑CLAVVPVFYPVTKS‑MKKQIWNLG‑‑‑‑‑‑‑‑‑‑‑‑DVRSD‑‑‑‑‑‑WPSALLETVDLPGHELYDT‑QSDSD‑‑FVEEIV

>F4JT82

‑EDTIRSFVSHLSAEFQRKGVSVFAED‑SASDDRAEESD‑‑AAIAKARVSVVIFSENFASSKGCLNEFLKVSKCRRSK‑GLVVVPVFYGLTNS‑VKKHCLELK‑‑‑‑‑‑‑‑‑‑‑‑KMYPDDK‑VDEWRNALWDIADLGGHVSSHK‑RSDSE‑‑LVEKIV

>O65506

GEDTLQSFASHLSMDFRRKGISAFV‑‑‑‑‑‑‑‑‑‑NYSETLDVIERVSASVLVFSKSCVSSTSCLDMLVRVFQCRRKT‑GQLVVPVYYGISSSDVQEH‑‑‑‑‑‑‑‑‑‑‑‑‑‑‑‑‑‑‑KSVDR‑IREWSSALQELRELPGHHNREE‑CSESE‑‑LVEEIV

>Q9FKE4

G‑‑‑TRSFVSHLSAAFRRRSVSVCLGD‑CTD‑VVPRKTN‑‑‑‑‑EGCKVFVVVFSEDYALSKQCLDTLVEFLE‑RKDD‑GLVIVPVYYGVTES‑VKQQTERFGVAFTQHQ‑‑‑‑‑NNYSYDQ‑VAKWRDCLIQTASLPGHELNLQ‑QEDSE‑‑FVEKIV

>F4JT79

‑LDVDESFIEAISKELHKQGFIPLT‑‑‑‑‑‑‑NLGRENLDEEMLYGSRVGIMILSSSYVSSRQSLDHLVAVMEHWKTT‑DLVIIPIYFKVRLSDLQLH‑‑‑‑‑‑‑‑‑‑‑‑‑‑‑‑‑MSLQEDR‑VQKWKAAMSEIVSIGGHEWT‑‑‑‑KGSQ‑‑FIEEVV

>F4JT78

‑LDVDESFIEAISKELHKQGFIPLT‑‑‑‑‑‑‑NLGRENLDEEMLYGSRVGIMILSSSYVSSRQSLDHLVAVMEHWKTT‑DLVIIPIYFKVRLSDLQLH‑‑‑‑‑‑‑‑‑‑‑‑‑‑‑‑‑MSLQEDR‑VQKWKAAMSEIVSIGGHEWT‑‑‑‑KGSQ‑‑FIEEVV

>F4KD49

‑NDSSVSFISYLMAGFGCRGIKQFL‑‑‑‑‑‑‑‑‑‑‑‑‑‑‑‑‑‑‑‑‑‑GIYLVILSRDYASSVLCLENLELCCDDK‑‑‑‑SYEVVPVFYGVSRSDVRQQSGPFSDAFTKLER‑‑‑‑‑SNPADH‑VTKWRRMFAKIAELKGHEYDEE‑SEESE‑‑FVEEIV

>F4I819

GDELRNSFVGFLVKAMRLEKINVFTDEV‑ELRG‑TNLNYLFRRIEESRVAVAIFSERYTESCWCLDELVKMKEQMEQG‑KLVVVPVFYRLNATACKRFMGAFGDNLRNLEWEY‑‑‑RSEPER‑IQKWKEALSSVFSNIGLTSDI‑RSNE‑‑SKFVDSIV

>F4I818

GDELRNSFVGFLVKAMRLEKINVFTDEV‑ELRG‑TNLNYLFRRIEESRVAVAIFSERYTESCWCLDELVKMKEQMEQG‑KLVVVPVFYRLNATACKRFMGAFGDNLRNLEWEY‑‑‑RSEPER‑IQKWKEALSSVFSNIGLTSDI‑RSNE‑‑SKFVDSIV

>F4I820

GDELRNSFVGFLVKAMRLEKINVFTDEV‑ELRG‑TNLNYLFRRIEESRVAVAIFSERYTESCWCLDELVKMKEQMEQG‑KLVVVPVFYRLNATACKRFMGAFGDNLRNLEWEY‑‑‑RSEPER‑IQKWKEALSSVFSNIGLTSDI‑RSNE‑‑SKFVDSIV

>Q9SYC9

GDELRNSFVGFLVKAMRLEKINVFTDEV‑ELRG‑TNLNYLFRRIEESRVAVAIFSERYTESCWCLDELVKMKEQMEQG‑KLVVVPVFYRLNATACKRFMGAFGDNLRNLEWEY‑‑‑RSEPER‑IQKWKEALSSVFSNIGLTSDI‑RRYNLINKNMDHTS

>Q56XU3

GEELRKSFLGFLLKAMRDAKINVFTDEI‑EVRG‑RDIQNLLSRIEESRVAIAILSKRYTESSWCLDELVKMKERIDQD‑ELVVIPIFYRLDATNCKRLEGPFGDNFRNLERDY‑‑‑RSEPER‑IKKWKEALISIPQKIGLTSAG‑HRDE‑‑SELVDSIV

>Q6NPD9

GEKLRDGFLGFLVDALLKENVNVFIDDH‑ELRG‑RDLDHLFSRIEESRVALTIFSKNFTNSRWCLDELAKIKECVDQE‑SLTVIPIFFKMKTDDVKKLKGNFGDNFRDLKLTH‑‑‑RGEPET‑YRRWKDAILYVSKKTGLSSSR‑YSRQ‑‑NDLVNTIV

>A8MR18

GKDLRKGFMSFLKPALKKEKINVFIDEQ‑EERG‑KYLISLFDTIGESKIALVIFSEGYCESHWCMDELVKIKEYMDQN‑RLIIIPIFYRLDLDVVKDLTGKFGDNFWDLVDKY‑‑‑QPEPKK‑LHKWTEALFSVCELFSLILPK‑HSDISDRDFVKSIV

>Q9C5Q9

GKDLRKGFMSFLKPALKKEKINVFIDEQ‑EERG‑KYLISLFDTIGESKIALVIFSEGYCESHWCMDELVKIKEYMDQN‑RLIIIPIFYRLDLDVVKDLTGKFGDNFWDLVDKY‑‑‑QPEPKK‑LHKWTEALFSVCELFSLILPK‑HSDISDRDFVKSIV

>F4ISS7

GKELRKGFISFLVPALKKKNINVFIDEH‑EVRG‑KDLISLFRRIGESKIALVIFSEGYTESKWCLDELVQIKKCVDQK‑KIIAIPIFYKLDPAVVKGLKGKFGDKFRDLIERY‑‑‑HHEPER‑YQKWTEALTSVSRTFALCLPE‑HSDKSEKDFIRSII

>O80617

GEQLRRSFVSHLIDAFERNEINFFVDKY‑EQRG‑KDLKNLFLRIQESKIALAIFSTRYTESSWCLDELVKIKKLADKK‑KLHVIPIFYKVKVEDVRKQTGEFGDNFWTLAKV‑‑‑‑‑SSGDQ‑IKKWKEALECIPNKMGLSLGD‑KSSE‑‑ADFIKEVV

>Q9SKM4

GEQLRRSFVSHLIDAFERNEINFFVDKY‑EQRG‑KDLKNLFLRIQESKIALAIFSTRYTESSWCMDELVKIKKLADKR‑KLHVIPIFYKVKVEDVRKQTGEFGDNFWTLAKV‑‑‑‑‑SSGDQ‑IKKWKEALECIPNKMGLSLGD‑KSDE‑‑ADFIKEVV

>F4HT77

GEEIRHGFISHLADALERYGIMFIIDKD‑EQRG‑NDLTSLLLRIKESKVALVIFSSRFAESRFCMDEIVKMKECVDER‑KLLVIPIFYKVRARDVSGRTGDFGKKFWALAQK‑‑‑‑‑SRGCQ‑IKEWMEALECISNKMGLSLGD‑GRSE‑‑ADFIKEIV

>Q9FVT6

GKDIRHGFVSHLKDALKRKNINFFIDTH‑EQKG‑RDLNHLFKRIEEATIALVILSPRYGESKWCLEELTTIMDQEEKG‑QMIVIPIFYKVRTEDVEKQTGEFGHMFWSCDEE‑‑‑‑‑ASLEE‑MEKWQVALKAVCNKIGLTLDL‑KRSE‑‑AKFIKKVL

>Q9FHE5

EEDVSKGLINFLEPVLQNKNINVFIDEE‑EVRG‑KGLKNLFKRIQDSKISLAIFS‑‑‑‑ESKCDFNDLLKNNESADE‑‑‑‑‑AIPIFYKVDAT‑‑‑‑‑‑GDLAD‑‑‑‑‑‑‑‑‑‑‑‑‑‑‑‑‑‑‑‑‑‑‑‑‑‑‑‑‑‑‑‑‑‑‑‑‑‑LQ‑NSVKCKKDLINSAV

>Q9FHE9

GKDLRNGFLSFLEPAMREANINVFIDKD‑EVVG‑TDLVNLFVRIQESRVAVVIFSKDYTSSEWCLDELAEIKDCINQG‑GLNAIPIFYKLAPSSVLELKGGFGDTFRVLKEKY‑‑‑KNDPER‑TQKWQEALESIPKLKGLRLAE‑KSDRNEREFMNEMI

>Q9FHE8

GADVRKHFISFLVPALREANINVFIDEN‑EFLG‑SEMANLLTRIEESELALVIFSVDFTRSHRCLNELAKIKERKDQG‑RLIVIPIFYKVKPSAVKFLEGKFGDNFRALERNN‑‑‑RHMLPI‑TQKWKEALESIPGSIGMPLAE‑QSERTDNDFINSMV

>F4KD45

GAELRHKFISHLLKALERERINVFIDTR‑ETMG‑TGLENLFQRIQESKIAIVVISSRYTESQWCLNELVKIKECVEAG‑TLVVFPVFYKVDVKIVRFLTGSFGEKLETLVL‑‑‑‑‑RHSE‑R‑YEPWKQALEFVTSKTGKRVEE‑NSDEGA‑‑EVEQIV

>Q9FHF0

GEDLRLGFVSHLVEALENDNIKVFIDNY‑ADKG‑EPLETLLTKIHDSKIALAIFSGKYTESTWCLRELAMIKDCVEKG‑KLVAIPIFYKVDPSTVRGVRGQFGDAFRDLEE‑‑‑‑‑RDVI‑K‑KKEWKQALKWIPGLIGITVHD‑KSPESE‑‑ILNEIV

>Q9XGM3

GADLRRRFVSHLVTALKLNNINVFIDDY‑EDRG‑QPLDVLLKRIEESKIVLAIFSGNYTESVWCVRELEKIKDCTDEG‑TLVAIPIFYKLEPSTVRDLKGKFGDRFRSMAK‑‑‑‑‑GDE‑‑R‑KKKWKEAFNLIPNIMGIIIDK‑KSVESE‑‑KVNEIV

>Q9SCZ2

GEELRNSFVSHLRSALVRHGVNIFIDTN‑EEKG‑KPLHVFFQRIEESRIALAIFSVRYTESKWCLNELVKMKECMDKG‑KLLIIPIFYKVKAYEVRYQKGRFGCVFKNLRN‑‑‑‑‑VDVH‑K‑KNQWSEALSSVADRIGFSFDG‑KSDEHN‑‑FINGIV

>O49471

GDELRNNFVSHLDKALRGKQINVFIDEA‑VEKG‑ENLDNLFKEIEKSRIALAIISQKYTESKWCLNELVKMKEL‑‑EG‑KLVTIPIFYNVEPATVRYQKEAFGAALTKTQE‑‑‑‑‑NDSDGQ‑MKKWKEALTYVSLLVGFPFNS‑KSKEKETTLIDKIV

>O65507

GKQLRNGFVSHLEKALRRDGINVFIDRN‑ETKG‑RDLSNLFSRIQESRIALAIFSSMYTESYWCLDELVKIKDCVDLG‑TLVVIPIFYMVDTDDVKNLKGAFGYTFWKLAK‑‑‑‑‑TCNGEK‑LDKWKQALKDVPKKLGFTLSE‑MSDEGE‑‑SINQIV

>F4KIF3

GVELRKNFVSHLEKGLKRKGINAFIDTD‑EEMG‑QELSVLLERIEGSRIALAIFSPRYTESKWCLKELAKMKERTEQK‑ELVVIPIFYKVQPVTVKELKGDFGDKFRELVK‑‑‑‑‑STDKKT‑KKEWKEALQYVPFLTGIVLDE‑KSDEDE‑‑VINIII

>F4IMF2

GSELRYTFVYYLRTALVKNGINVFTDNM‑EPKG‑RNQKILFKRIEESKIALAIFSSRYTESSWCLEELVKMKECMDAE‑KLVIIPIFYIVTPYTIKKQMGDFGDKFRVLVD‑‑‑‑‑YVDDVT‑EKKWTDALKSVPLILGITYDG‑QSEEQL‑‑LINQIV

>O48573

GADLRNGFISHLAGALTSAGITYYIDTE‑EVPS‑EDLTVLFKRIEESEIALSIFSSNYAESKWCLDELVKIMEQVKKG‑KLRIMPVFFNVKPEEVREQNGEFGLKLYGEGK‑‑‑‑‑SKRP‑N‑IPNWENALRSVPSKIGLNLAN‑FRNEKE‑‑LLDKII

>Q9FKE2

GDELRKTFISHLHKRLQRDGINAFIDSD‑EAVG‑EELKNLFKRIENSEIALAVLSSRYTESHWCLQELVKMMECSMKGKKLLVIPIFYKLKIDTVKELDGDFGRNLWDLWRKPC‑GRDRDSR‑IVKWNEALKYFLSRNALVFSE‑TGKEEE‑‑FVSTIA

>F4JT81

GADTRHDFTSHLVKYLRGKGIDVFSDAK‑LRGG‑EYISLLFDRIEQSKMSIVVFSEDYANSWWCLEEVGKIMQRRKEF‑NHGVLPIFYKVSKSDVSNQTGSFEAVFQSPTKIF‑‑‑G‑DEQK‑IEELKVALKTASNIRGFVYPE‑NSSEPD‑‑FLDEIV

>F4JT80

GADTRHDFTSHLVKYLRGKGIDVFSDAK‑LRGG‑EYISLLFDRIEQSKMSIVVFSEDYANSWWCLEEVGKIMQRRKEF‑NHGVLPIFYKVSKSDVSNQTGSFEAVFQSPTKIF‑‑‑G‑DEQK‑IEELKVALKTASNIRGFVYPE‑NSSEPD‑‑FLDEIV

>Q9SZ66

GFDTRNNFTGHLQKALRLRGIDSFIDDR‑LRRG‑DNLTALFDRIEKSKIAIIVFSTNYANSAWCLRELVKILECRNSN‑QQLVVPIFYKVDKSDVEKQRNSFAVPFKLPELTF‑‑‑GVTPEE‑ISSWKAALASASNILGYVVKE‑STSEAK‑‑LVDEIA

>F4JU10

GNDLRKGFVSHVVKALKDARVNVFVDNDHERRGPHDDHLFVRRIHNSKLALVIFSDQYAESQQCLNELTTIHERVAEG‑KLMVIPIFYKVNIEEVNNLEGRFGKCFDEMVRTQ‑‑‑RQNHQL‑THHIVGCLRSIARKPSFTSGY‑YSNDSD‑‑LVEAII

>F4JU09

GKELRHGFVSHVVKALRIAGVNVFIDSN‑EMKG‑RDLQNLFKRIENSKMALVIFSDRFSESDWCLNELVKIDDCVKEG‑KLTVIPVFYRVNTDDVKNFKGKFGSCFIETVQRQ‑‑‑PKEEPM‑AERWVNSVKSISSKTGFTSEV‑HRIDSY‑‑LVDAIV

>Q0WSX8

GDELREIFVNHLELQLRNAGINVFIDTK‑EQKG‑‑RLQYLFTRIKKSKIALAIFSKRYCESKWCLDELVTMNEQMKEK‑KLVVIPIFYNVRSDDVKRLDGEFSLPFKQLKQNH‑‑‑AGEPER‑VEGWERALRSVTKRIGFSRSNSYKHDTDFVL‑‑DIV

>F4KBQ6

GDELREIFVNHLELQLRNAGINVFIDTK‑EQKG‑‑RLQYLFTRIKKSKIALAIFSKRYCESKWCLDELVTMNEQMKEK‑KLVVIPIFYNVRSDDVKRLDGEFSLPFKQLKQNH‑‑‑AGEPER‑VE‑‑‑‑‑‑‑‑‑‑‑‑‑GFSRSNSYKHDTDFVL‑‑DIV

>Q56XU3

NDELGDNFIKHLVWALRDSGINVFKDSF‑KLIG‑GQKQEVFMSIENSNIALAIFSKRYSESYRCLNELVKMEELAKEG‑KLVVIPVFYSVKTNEVRRLEGEFGIHFRNTKERF‑‑‑AMEPMM‑VESWEKSLKSVTGRIGLSLE‑‑‑AHMNEFALVGAIV

>Q9FLA7

GNESRDNFIKYLVWGLRDERVNVFVDRA‑EANR‑RDIRNISTKIEESNIAVVIFSKRYTESEMCLNELQKMYEHVEQS‑NLKVIPVFYDVSISGVKNLEDEFGNHFEELREKY‑‑‑ANDPLK‑ILKWEDSLSSIVERTGLTSED‑HGTGLG‑‑LVRAIV

>F4JNL1

GKAQRKTLVSFIKSKLEESEINVFMDEY‑EIRG‑RPITTLFERIRESSIALVIFSDKYPESRWCLDELVEIKKQMETG‑SIVPFPIFYKVKAESVKNQTGHFRNVLLKTEEDVKKILETEDM‑IWGWRQALVSVGGRMGFS‑‑‑‑YNHKCDNDFVNDIV

>F4JU08

ESDQSDGFIRHVERALNDEGFNVFIDSD‑ERRG‑RGMEHIFRAIDNSNVALVIFSDRYTASELCLHEAVRIYDRRREG‑KLVLIPVFYRVSEDDVNMFNGRFGESFLETLTIQ‑‑‑FRDHPF‑AEHWMRNVNFICTDTGFTSAD‑SSNDTS‑‑LVEEIV

>Q9FKE3

GS‑‑RMGFIYHLIMALEKKNINVFVGFN‑‑‑‑GCEPVERLSNRIE‑SIIVLVIFTSRYTESKWCLMKLVDINKCAEKD‑HLVAIPIFYKLDPSTVRGLSGQFGDAFRDLREST‑‑‑‑‑‑‑‑L‑MEKWKEALKSISDRPGIRVDK‑SSPKAK‑‑‑‑‑RIE

>F4JNL2

GKDERNGLLTLLKQKLIDGNVNVFTDD‑‑KLTG‑QPLQNLFGHIRKSRIAIVIFSKNYAESGWCLDELVEIKKCFETEALKAVIPIFHRVKVSSVKKQSGKFGEKFLALQNYLAE‑KRINSR‑IKRWKKALKIVTEIAGLTHDK‑NSPELA‑‑FVEKVV

>F4IUF0

SKDTRDNFVSHLCGCLRRKRIKTFLDELP‑‑‑‑‑ERYEESLKAIEVSKISVIVFSENFGDSRWCLDEVVAILKCKEKF‑GQIVIPVLYHVDPLDIENQTGSFGDAFAKR‑‑‑‑‑‑‑RDKAEQ‑LQEWKDSFTEAINLPGWSTAYL‑SDEEM‑‑LVNGIA

>Q9SUS7

GSDVRYNFFSFLKDALIKNGINVVTDE‑‑APRGKPIDENLLKLIKDSRIAVVIFSENYPESTWCLDELVEIEKQMDLK‑MLDSCPIFFEVETCHVKLARSTFNYNLLQLEHDESKAWEDAEKRFEGWRKALISVASRLGLTYKKG‑SNQAT‑‑FVNEIV

>F4JNL1

GKHLGKFLVSSLKEELESNQILVYVDETK‑‑‑‑‑‑‑‑‑‑‑‑SRIKESGVAVVFFSKKYPNSEKCLDELVEIKKLMDAG‑KIDPLPVFYSLKDEPVKNLKGYFLNRLLKIENEVIK‑LDTEAK‑IWGWRDALSSIASRPGLSYEL‑‑STDDV‑‑FVSDIV

**Aligned *G. max* sequences from Pfam used to build profile HMM after alignment pruning and exclusion.**

>K7MH68

GEDTRYG‑FTGNLYNVLRERG‑‑IHTFI‑DDE‑‑‑E‑‑LQKGDEITTALE‑‑‑EAIEKSKI‑FIIVLS‑‑‑‑‑‑‑‑‑‑‑‑‑‑‑‑‑‑‑‑‑‑‑‑‑‑‑‑‑‑‑‑‑‑‑‑‑‑‑‑‑‑‑‑‑‑‑‑‑‑‑‑‑‑‑‑‑‑‑‑‑‑‑‑‑‑‑‑‑ENYAYSSFCLNELTHILNF‑TE‑‑‑GKNDPLVLPVFYKVNP‑‑SYVRH‑‑HRGS‑YGEALANH‑EK‑‑KLNSN‑‑‑‑‑‑‑NM‑‑‑‑‑‑‑‑‑‑‑‑‑‑‑‑‑‑‑‑‑‑‑‑‑‑‑‑‑‑‑‑‑‑‑‑‑‑‑‑‑‑‑‑‑‑‑‑‑‑‑‑‑‑‑‑‑‑‑‑‑‑‑‑‑‑‑‑‑‑‑‑‑‑‑‑‑‑‑‑‑‑‑‑‑‑‑‑‑‑‑‑‑‑‑‑‑‑‑‑‑‑‑‑‑‑‑‑‑‑‑‑‑‑‑‑‑‑‑‑‑‑‑‑‑‑‑‑‑‑‑‑‑‑‑‑‑‑‑‑‑‑‑‑‑‑‑‑‑‑‑‑‑‑‑‑‑‑‑‑‑‑‑‑‑‑‑‑‑‑‑‑‑‑‑‑‑‑‑‑‑‑‑‑‑‑‑‑‑‑‑‑‑‑‑‑‑‑‑‑‑‑‑‑‑‑‑‑‑‑‑‑‑‑‑‑‑‑‑‑‑‑‑‑‑‑‑‑‑‑‑‑‑‑‑‑‑‑‑‑‑‑‑‑‑EKLETWKMALRQV‑SNIS‑‑‑‑‑‑‑‑‑‑‑‑‑‑‑‑‑‑‑‑‑‑‑‑‑‑‑‑‑‑‑‑‑‑‑‑GH‑HLQHD‑G‑‑NK‑‑‑‑‑‑‑‑‑YE‑YKFIKEIV

>I1MND4

GEDTRYG‑FTGYLYNVLRERG‑‑IHTFI‑DDD‑‑‑E‑‑PQEGDEITTALE‑‑‑AAIEKSKI‑FIIVLS‑‑‑‑‑‑‑‑‑‑‑‑‑‑‑‑‑‑‑‑‑‑‑‑‑‑‑‑‑‑‑‑‑‑‑‑‑‑‑‑‑‑‑‑‑‑‑‑‑‑‑‑‑‑‑‑‑‑‑‑‑‑‑‑‑‑‑‑‑ENYASSSFCLNSLTHILNF‑TK‑‑‑ENNDVLVLPVFYRVNP‑‑SDVRH‑‑HRGS‑FGEALANH‑EK‑‑KSNSN‑‑‑‑‑‑‑NM‑‑‑‑‑‑‑‑‑‑‑‑‑‑‑‑‑‑‑‑‑‑‑‑‑‑‑‑‑‑‑‑‑‑‑‑‑‑‑‑‑‑‑‑‑‑‑‑‑‑‑‑‑‑‑‑‑‑‑‑‑‑‑‑‑‑‑‑‑‑‑‑‑‑‑‑‑‑‑‑‑‑‑‑‑‑‑‑‑‑‑‑‑‑‑‑‑‑‑‑‑‑‑‑‑‑‑‑‑‑‑‑‑‑‑‑‑‑‑‑‑‑‑‑‑‑‑‑‑‑‑‑‑‑‑‑‑‑‑‑‑‑‑‑‑‑‑‑‑‑‑‑‑‑‑‑‑‑‑‑‑‑‑‑‑‑‑‑‑‑‑‑‑‑‑‑‑‑‑‑‑‑‑‑‑‑‑‑‑‑‑‑‑‑‑‑‑‑‑‑‑‑‑‑‑‑‑‑‑‑‑‑‑‑‑‑‑‑‑‑‑‑‑‑‑‑‑‑‑‑‑‑‑‑‑‑‑‑‑‑‑‑‑‑‑EKLETWKMALHQV‑SNIS‑‑‑‑‑‑‑‑‑‑‑‑‑‑‑‑‑‑‑‑‑‑‑‑‑‑‑‑‑‑‑‑‑‑‑‑GH‑HFQHD‑G‑‑NK‑‑‑‑‑‑‑‑‑YE‑YKFIKEIV

>I1MNC5

GEDTRYS‑FTGNLYNVLRERG‑‑IHTFI‑DDD‑‑‑E‑‑FQKGDQITSALE‑‑‑EAIEKSKI‑FIIVLS‑‑‑‑‑‑‑‑‑‑‑‑‑‑‑‑‑‑‑‑‑‑‑‑‑‑‑‑‑‑‑‑‑‑‑‑‑‑‑‑‑‑‑‑‑‑‑‑‑‑‑‑‑‑‑‑‑‑‑‑‑‑‑‑‑‑‑‑‑ENYASSSFCLNELTHILNF‑TK‑‑‑GKNDLLVLPVFYIVDP‑‑SDVRH‑‑HRGS‑FGEALANH‑EK‑‑KLNSD‑‑‑‑‑‑‑NM‑‑‑‑‑‑‑‑‑‑‑‑‑‑‑‑‑‑‑‑‑‑‑‑‑‑‑‑‑‑‑‑‑‑‑‑‑‑‑‑‑‑‑‑‑‑‑‑‑‑‑‑‑‑‑‑‑‑‑‑‑‑‑‑‑‑‑‑‑‑‑‑‑‑‑‑‑‑‑‑‑‑‑‑‑‑‑‑‑‑‑‑‑‑‑‑‑‑‑‑‑‑‑‑‑‑‑‑‑‑‑‑‑‑‑‑‑‑‑‑‑‑‑‑‑‑‑‑‑‑‑‑‑‑‑‑‑‑‑‑‑‑‑‑‑‑‑‑‑‑‑‑‑‑‑‑‑‑‑‑‑‑‑‑‑‑‑‑‑‑‑‑‑‑‑‑‑‑‑‑‑‑‑‑‑‑‑‑‑‑‑‑‑‑‑‑‑‑‑‑‑‑‑‑‑‑‑‑‑‑‑‑‑‑‑‑‑‑‑‑‑‑‑‑‑‑‑‑‑‑‑‑‑‑‑‑‑‑‑‑‑‑‑‑‑ENLETWKMALHQV‑SNIS‑‑‑‑‑‑‑‑‑‑‑‑‑‑‑‑‑‑‑‑‑‑‑‑‑‑‑‑‑‑‑‑‑‑‑‑GH‑HFQHD‑G‑‑NK‑‑‑‑‑‑‑‑‑YE‑YKFIKEIV

>K7MH83

GEDTRYG‑FTGNLYKVLQERG‑‑IHTFI‑DDE‑‑‑E‑‑LQEGDQITTALE‑‑‑EAIEKSKI‑FIIVLS‑‑‑‑‑‑‑‑‑‑‑‑‑‑‑‑‑‑‑‑‑‑‑‑‑‑‑‑‑‑‑‑‑‑‑‑‑‑‑‑‑‑‑‑‑‑‑‑‑‑‑‑‑‑‑‑‑‑‑‑‑‑‑‑‑‑‑‑‑ENYASSSFCLNELTHILNF‑TK‑‑‑ENNDVLVLPVFYKVDP‑‑SDVRH‑‑HRGS‑FGEALANH‑EK‑‑NLNSN‑‑‑‑‑‑‑NM‑‑‑‑‑‑‑‑‑‑‑‑‑‑‑‑‑‑‑‑‑‑‑‑‑‑‑‑‑‑‑‑‑‑‑‑‑‑‑‑‑‑‑‑‑‑‑‑‑‑‑‑‑‑‑‑‑‑‑‑‑‑‑‑‑‑‑‑‑‑‑‑‑‑‑‑‑‑‑‑‑‑‑‑‑‑‑‑‑‑‑‑‑‑‑‑‑‑‑‑‑‑‑‑‑‑‑‑‑‑‑‑‑‑‑‑‑‑‑‑‑‑‑‑‑‑‑‑‑‑‑‑‑‑‑‑‑‑‑‑‑‑‑‑‑‑‑‑‑‑‑‑‑‑‑‑‑‑‑‑‑‑‑‑‑‑‑‑‑‑‑‑‑‑‑‑‑‑‑‑‑‑‑‑‑‑‑‑‑‑‑‑‑‑‑‑‑‑‑‑‑‑‑‑‑‑‑‑‑‑‑‑‑‑‑‑‑‑‑‑‑‑‑‑‑‑‑‑‑‑‑‑‑‑‑‑‑‑‑‑‑‑‑‑‑EKLQIWKKALHQV‑SNIS‑‑‑‑‑‑‑‑‑‑‑‑‑‑‑‑‑‑‑‑‑‑‑‑‑‑‑‑‑‑‑‑‑‑‑‑GY‑HFQDD‑G‑‑NK‑‑‑‑‑‑‑‑‑YE‑YKFIKEIV

>K7MH74

GEDTRYC‑FTGNLYNVLRERG‑‑IHTFI‑DDD‑‑‑E‑‑LQKGDQITSALQ‑‑‑EAIEKSKI‑FIIVLS‑‑‑‑‑‑‑‑‑‑‑‑‑‑‑‑‑‑‑‑‑‑‑‑‑‑‑‑‑‑‑‑‑‑‑‑‑‑‑‑‑‑‑‑‑‑‑‑‑‑‑‑‑‑‑‑‑‑‑‑‑‑‑‑‑‑‑‑‑ENYASSSFCLNELTHILNF‑TK‑‑‑GKNDLLVLPVFYIVDP‑‑SDVRH‑‑HRGS‑FGEALANH‑EK‑‑KLNST‑‑‑‑‑‑‑NM‑‑‑‑‑‑‑‑‑‑‑‑‑‑‑‑‑‑‑‑‑‑‑‑‑‑‑‑‑‑‑‑‑‑‑‑‑‑‑‑‑‑‑‑‑‑‑‑‑‑‑‑‑‑‑‑‑‑‑‑‑‑‑‑‑‑‑‑‑‑‑‑‑‑‑‑‑‑‑‑‑‑‑‑‑‑‑‑‑‑‑‑‑‑‑‑‑‑‑‑‑‑‑‑‑‑‑‑‑‑‑‑‑‑‑‑‑‑‑‑‑‑‑‑‑‑‑‑‑‑‑‑‑‑‑‑‑‑‑‑‑‑‑‑‑‑‑‑‑‑‑‑‑‑‑‑‑‑‑‑‑‑‑‑‑‑‑‑‑‑‑‑‑‑‑‑‑‑‑‑‑‑‑‑‑‑‑‑‑‑‑‑‑‑‑‑‑‑‑‑‑‑‑‑‑‑‑‑‑‑‑‑‑‑‑‑‑‑‑‑‑‑‑‑‑‑‑‑‑‑‑‑‑‑‑‑‑‑‑‑‑‑‑‑‑ENLETWKIALHQV‑SNIS‑‑‑‑‑‑‑‑‑‑‑‑‑‑‑‑‑‑‑‑‑‑‑‑‑‑‑‑‑‑‑‑‑‑‑‑GY‑HFQHD‑G‑‑DK‑‑‑‑‑‑‑‑‑YE‑YKFIKEIV

>K7MH86
[truncated: 401,565 more chars]
